# Enantioselective Inverse-Electron Demand Aza-Diels–Alder Reaction: ipso,α-Selectivity of Silyl Dienol Ethers

**DOI:** 10.1021/acscatal.1c03390

**Published:** 2021-09-15

**Authors:** Víctor Laina-Martín, Jorge Humbrías-Martín, Rubén Mas-Ballesté, Jose A. Fernández-Salas, José Alemán

**Affiliations:** † Departamento de Química Orgánica (módulo 1), Universidad Autónoma de Madrid, Cantoblanco, 28049 Madrid, Spain; ‡ Departamento de Química Inorgánica (módulo 7), Universidad Autónoma de Madrid, Cantoblanco, 28049 Madrid, Spain; § Institute for Advanced Research in Chemical Sciences (IAdChem), Universidad Autónoma de Madrid, 28049 Madrid, Spain

**Keywords:** organocatalysis, Diels−Alder, hydrogen-bonding activation, benzofuran derivatives, bifunctional catalysis, squaramide

## Abstract

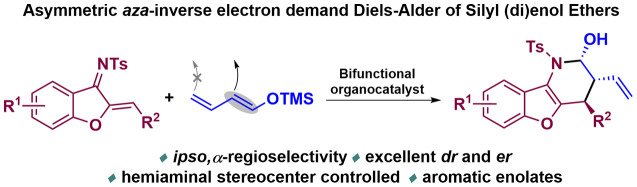

A highly efficient enantioselective inverse-electron-demand aza-Diels–Alder reaction between aza-sulfonyl-1-aza-1,3-butadienes and silyl (di)enol ethers has been developed. The presented methodology allows the synthesis of benzofuran-fused 2-piperidinol derivatives with three contiguous stereocenters in a highly selective manner, as even the hemiaminal center is completely stereocontrolled. Density functional theory (DFT) calculations support that the hydrogen-bond donor-based bifunctional organocatalyst selectively triggers the reaction through the ipso,α-position of the dienophile, in contrast to the reactivity observed for dienolates in situ generated from β,γ-unsaturated derivatives. Moreover, the calculations have clarified the mechanism of the reaction and the ability of the hydrogen-bond donor core to hydrolyze selectively the *E* isomer of the dienol ether. Furthermore, to demonstrate the applicability of silyl enol ethers as nucleophiles in the asymmetric synthesis of interesting benzofuran-fused derivatives, the catalytic system has also been implemented for the highly efficient installation of an aromatic ring in the piperidine adducts.

## Introduction

**Scheme 1:**
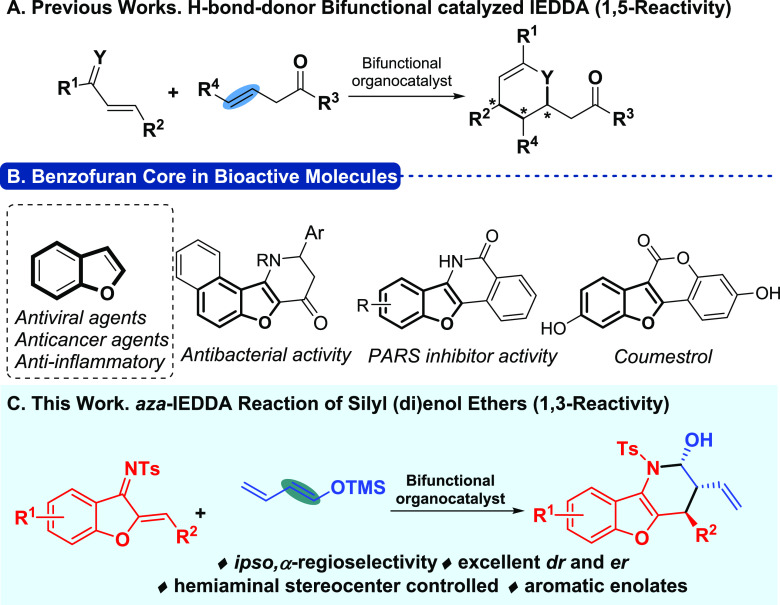
Organocatalyzed Hetero-IEDDA Reaction: Previous Works (Equation A) and Present Work (Equation C)

The development of efficient and practical strategies for the stereoselective construction of C–C bonds still stands as a crucial ongoing objective and preserves a preferred position in the organic chemistry research.^1^ Especially interesting are those synthetic tools able to generate structural diversity in a single operation. Among these exceptional types of reactions, Diels–Alder (DA) cycloaddition is recognized as one of the most useful and employed reactions in modern organic chemistry.^2^ In this regard, the inverse-electron-demand Diels–Alder (IEDDA) reaction has emerged as a very interesting variant that permits the synthesis of optically active six-membered heterocycles, as it incorporates the functionality into the ring, allowing also the construction of C-heteroatom bonds.^3^ Apart from a large number of Lewis-acid catalytic systems,^3a−3e^ organocatalytic alternatives,^3a−3e,4^ which are continuously evolving, have also been harnessed to develop new asymmetric IEDDA reactions. Focusing on hetero-IEDDA metal-free systems, numerous studies have been reported over the past decades, involving a highest occupied molecular orbital (HOMO)-raising activation of electron-deficient dienophiles by aminocatalysis, being one of the preferred approaches for such a challenging goal.^3a−3e,4a^ This strategy involves *in situ* generation of an enamine that reacts with electron-poor dienes such as α,β-unsaturated systems to generate a variety of oxygen- and nitrogen-based heterocycles.^3a−3e,4a,5^ This approach has been later extended to dienamine as dienophile species when formed from the corresponding α,β-unsaturated aldehyde.^3a−3e,4a^ In this context, the majority of these interesting studies showed how the dienamine reacted through the terminal β,γ-alkene (or reactivity 1,5).^6,7^ Following an analogous strategy, the activation of dienolates by the hydrogen-bond donor-based bifunctional catalytic asymmetric IEDDA reaction has been much less studied.^8^ Thus, Huang^9^ and Fang^10^ reported the synthesis of dihydropyrans with α,β-unsaturated amides and ketones, respectively. Very recently, Albrecht and co-workers have described a very efficient aza-IEDDA reaction following a similar strategy.^11^ Nevertheless, all of these examples show how the reaction takes place through the β,γ-alkene of the in situ generated dienolate (1,5-reactivity, Scheme 1A).^9−11^

In this context, benzofuran derivatives have been identified as recognizable relevant cores in heterocyclic ring systems due to their diverse biological activity (Scheme 1B).^12^ As can be observed in Scheme 1C, a switch of the reactive position of the silyl dienol ether (1,3 vs 1,5-reactivity) would lead to a different family of products through an attractive and alternative synthetic approach. Moreover, in this latter strategy, in which a hemiaminal is formed, an additional oxidation/reduction operation step is usually required to obtain the corresponding lactams/piperidines since the diastereoselectivity of the mentioned hemiaminal stereocenter is usually low.^5a−5c,7^ The thermodynamic control of the different quasi-barrierless steps involved in the preparation of a hemiaminal has for long limited its asymmetric synthesis,^13^ although their enantioselective synthesis could be of crucial interest for the preparation of chiral oxazolidines.^13b^

Taking all of these and our previous experience^14^ into consideration, we envisioned that the reaction of silyl dienol ethers in the presence of a H-bond donor-based bifunctional organocatalyst would lead to a new asymmetric aza-IEDDA reaction with the alternative ipso,α-selectivity of the dienolate (1,3-selectivity). Herein, we describe a regioselective aromative aza-IEDDA reaction of silyl dienol ethers and α,β-unsaturated imines **1**. Benzofuran-fused 2-piperidinol derivatives with three contiguous stereocenters are obtained with excellent enantioselectivities and complete diastereoselectivity, controlling even the hemiaminal stereocenter (Scheme 1C). In addition, this methodology allows the synthesis of the corresponding DA-cycloadducts bearing an aromatic ring, using phenylacetaldehyde-derived silyl enol ether as the phenyl installation remains as a scope limitation for previously reported non-vinylogous enamine-based organocatalytic methods.^5a−5c,7^

## Results and Discussion

Based on our experience in bifunctional catalysis and activation of silicon reagents,^14^ we began our investigations by studying the reaction of diene **1a** as a model substrate with trimethyl silyl dienolate derivative **2a** as dienophile and in the presence of different thiourea and squaramide bifunctional organocatalysts (Table 1). First, we examined commercially available catalyst **3a** in tetrahydrofuran (THF), and, to our delight, the desired aza-IEDDA adduct was obtained in good yield with complete regio- and diastereoselectivity (entry 1). We then evaluated different solvents (entries 1–5), and THF was considered the optimal one. The hydrogen-bond donor unit of the catalyst was then studied, and we observed how the squaramide core (**3e**) led to a better result when compared with the analogous cinchonidine-thiourea-based catalyst **3b** (entries 6 and 7). Different squaramide-based organocatalysts were subsequently tested (entries 7–10). Catalysts **3c** and **3d** still led to the desired product with complete regio- and diastereoselectivity, but the enantiomeric excesses (ee) were slightly diminished (entries 8 and 9), while **3f** showed worse reactivity, leading to a lower yield of product **4a** (entry 10). At 40 °C, the aza-IEDDA adduct was obtained with a slightly higher yield, maintaining the excellent enantioselectivity observed when performed at room temperature (rt) (entry 11 vs 7). Thus, we tested the reaction with an increased amount of the nucleophile and the desired cycloadduct was obtained with 67% yield (entry 12). In addition, under the same reaction conditions, trimethyl(styryloxy)silane **2b** showed a very high reactivity, leading to the phenyl-bearing aza-IEDDA adduct with very good yield and enantioselectivity (entry 13).

**Table 1:**
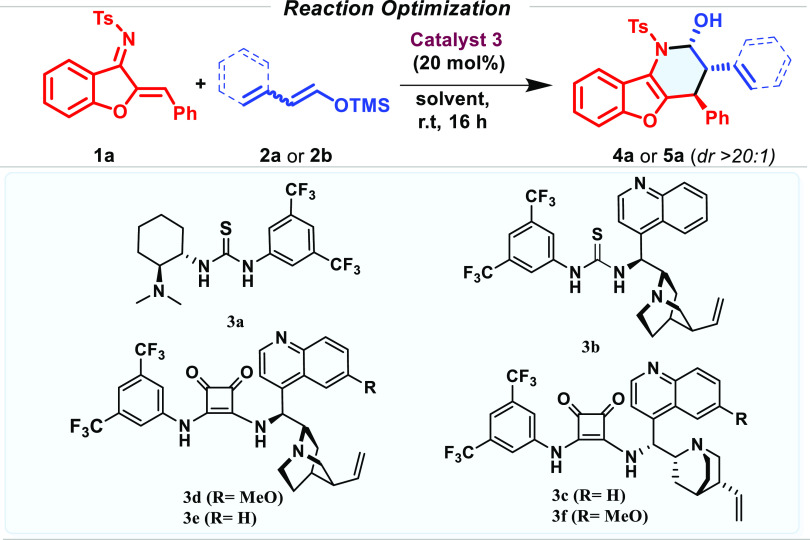
Optimization of the Reaction Conditions

entry^a^	organocatalyst	solvent	yield (%)	er^e^
1	**3a**	THF	56-**4a**	88:12
2	**3a**	DCM	50-**4a**	87:13
3	**3a**	toluene	28-**4a**	69:31
4	**3a**	DCE	54-**4a**	82:18
5	**3a**	Et_2_O	44-**4a**	76:24
6	**3b**	THF	48-**4a**	92:8
7	**3e**	THF	42-**4a**	96:4
8	**3c**	THF	41-**4a**	9:91
9	**3d**	THF	41-**4a**	93:7
10	**3f**	THF	29-**4a**	3:97
11^b^	**3e**	THF	56-**4a**	97:3
**12**^c^	**3e**	**THF**	**67-4a**	**97:3**
**13**^d^	**3e**	**THF**	**78-5a**	**94:6**

a Standard reaction conditions: 0.05 mmol of **1a**, 0.15 mmol of **2a** (mixture of isomers, 70(*E*):30(*Z*)), and 0.01 mmol of 3 in 0.3 mL of solvent.

b Reaction was carried out at 40 °C.

c Reaction was carried out in the presence of 0.3 mmol of (buta-1,3-dien-1-yloxy) trimethylsilane (mixture of isomers, 70(*E*):30(*Z*)).

d Reaction was carried out in the presence of 0.3 mmol of trimethyl(styryloxy)silane **2b** (mixture of isomers, 65(*Z*):35(*E*)).

e Enantiomeric ratio determined by supercritical fluid chromatography (SFC).

Once the reaction conditions had been optimized (entries 12 and 13, Table 1), we studied the scope of the reaction considering differently substituted aza-sulfonyl-1-aza-1,3-butadienes (**1**) with **2a** (Table 2) and **2b** (Table 3). The aza-IEDDA reaction embraced a variety of aromatics with complete regio- and diastereoselectivity observed in all cases. The reaction proceeded smoothly with differently substituted halogenated aromatic rings in *para* (**4b** and **4c**), *meta* (**4g**), and *ortho* (**4h** and **4i**) positions, leading to the aza-IEDDA adducts with excellent enantioselectivities. Substitution on the core aromatic ring was examined in the aza-IEDDA reaction. The presence of a bromine atom at the 8- and 7-positions of the phenyl ring (**4l** and **4m**) was also very well tolerated, keeping the high efficiency of the system. Aza-sulfonyl-1-aza-1,3-butadienes bearing an electron-poor aromatic ring (*p*-NO_2_, *p*-CF_3_, and *p*-CN), led to the desired products **4d**, **4e**, and **4f** with excellent enantioselectivities, maintaining the same reactivity level. Dienes bearing an electron-rich aromatic ring (**1l**) did not show enough electrophilicity, and no reactivity under the optimized reaction conditions was observed. However, the presence of a methoxy group at the *meta* position of the aromatic ring was well tolerated, leading to an excellent enantioselectivity of the corresponding cycloadduct (**4j**). In addition, the protocol also enabled access to the corresponding hemiaminal bearing a sterically encumbered naphthyl derivative (**4k**). Differently substituted silyl dienol derivatives were subjected to the optimized reaction conditions. Gratifyingly, methyl substituents at positions C3 and C4 of the silyl dienol ether led to the desired aza-IEDDA adduct with complete enantiocontrol (**4n** and **4o**). The presence of a substituent at the C2 position was, however, not tolerated, and the reaction did not take place.

**Table 2:**
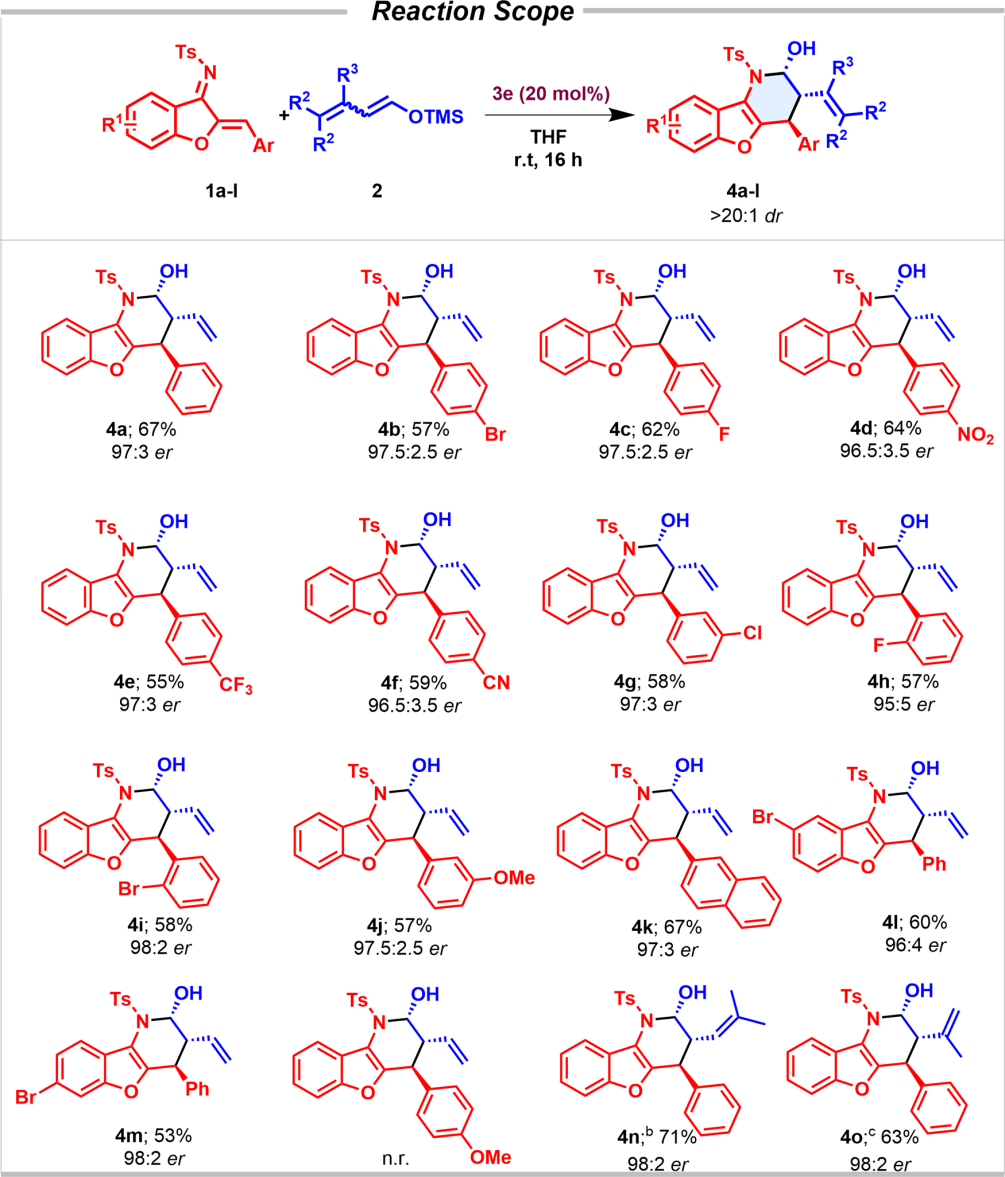
ipso,α-Selective Asymmetric Aza-IEDDA between Diene 1 and Silyl Dienol Ether **2**^a^^c^

a All of the reactions were performed on a 0.05 mmol scale of **1** in 0.3 mL of THF at rt. Diastereomeric ratios were determined by ^1^H NMR of the crude mixture. Enantiomeric excesses were determined by SFC chromatography.

b Reaction was carried out in the presence of 0.3 mmol of **2c** (mixture of isomers, 95(*E*):5(*Z*)).

c Reaction was carried out in the presence of 0.3 mmol of mixture of **2d** (isomers, 95(*E*):5(*Z*)).

**Table 3:**
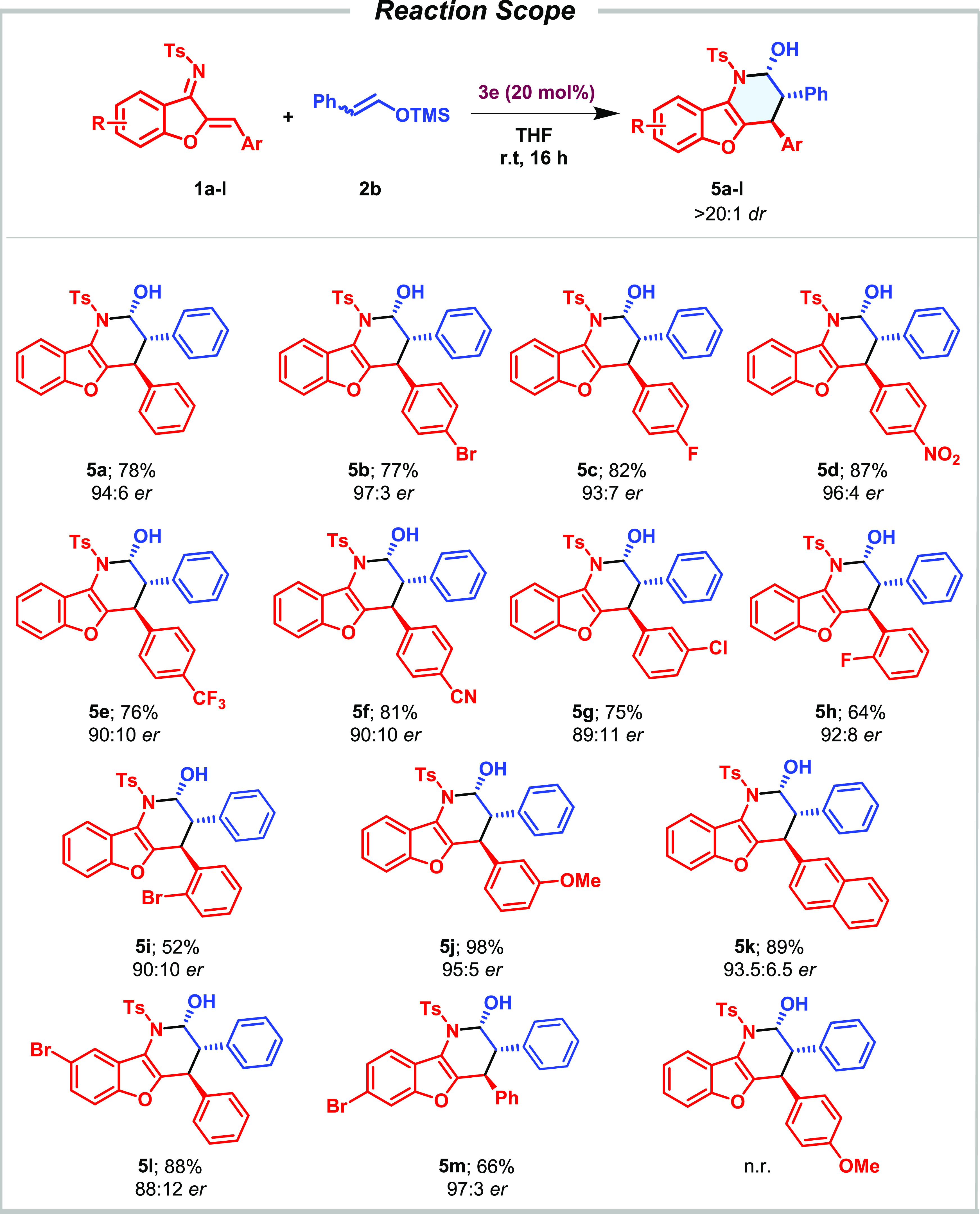
Enantioselective Aza-IEDDA between Diene **1** and Trimethyl(styryloxy)silane **2b**^a^

a All of the reactions were performed on a 0.05 mmol scale of **1** in 0.3 mL of THF at rt. Diastereomeric ratios were determined by ^1^H NMR of the crude mixture. Enantiomeric excesses were determined by SFC chromatography.

With the idea to expand the scope of the presented aza-IEDDA reaction and considering what has been reported in the literature to date and its limitation in the introduction of an aromatic ring,^5a−5c,7^ we studied the scope of the reaction in the presence of trimethyl(styryloxy)silane **2b**. This strategy represents a straightforward alternative to access the corresponding aza-IEDDA adducts bearing an aromatic ring in the α position of the activating group. The system allowed the synthesis of the desired phenyl-bearing cycloadducts from differently substituted dienes (**1a**–**k**) with similar levels of enantioselectivity (up to 94% ee) and a slightly increased reactivity (Table 3). The reaction tolerated substituted halogenated aromatic rings in *para* (**5b** and **5c**), *meta* (**5g**), and *ortho* (**5h** and **5i**) positions. Halogen substitution of the benzofuran core led to the desired adduct with a slightly diminished enantioselectivity when allocated at the 8-position of the aromatic ring core (**5l**), while a bromine atom at position 7 afforded the cycloadduct with excellent results (**5m**). Deactivating groups on the aromatic ring of the α,β-unsaturated imine led to the corresponding tricyclic compounds with very good yields and high enantioselectivities (**5d**–**f**). Naphthyl- and 3-methoxy-substituted aromatic rings were well tolerated, leading to the final cycloadducts with excellent yields and enantioselectivities (**5j** and **5k**). As observed before, more electron-rich dienes (*p*-MeO aryl substitution) resulted to be unreactive under these reaction conditions.

**Scheme 2:**
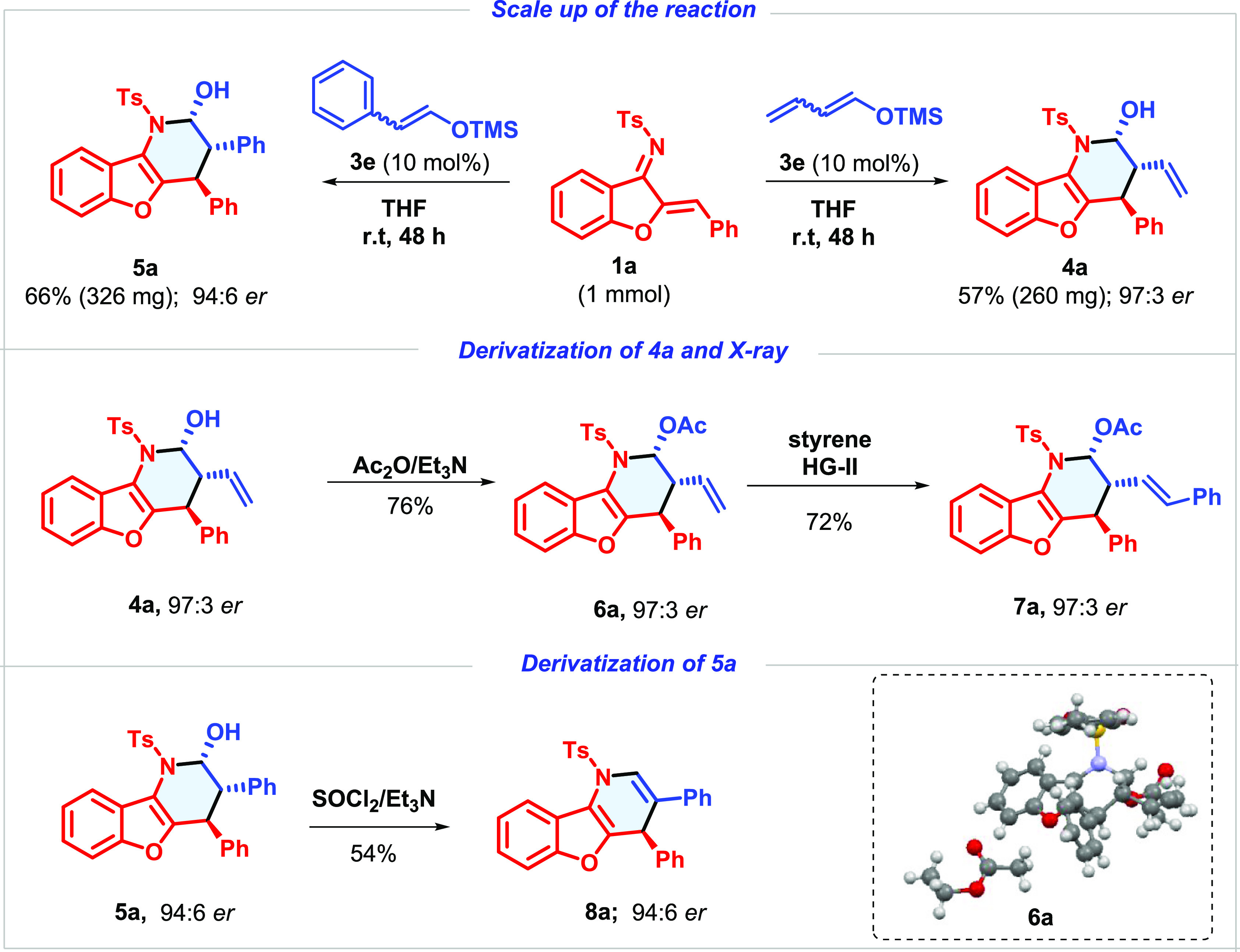
Scale-Up of the Reaction and Useful Transformations

To our delight, the reaction proceeded efficiently starting from 1 mmol (20 times scale-up) of **1a** in the presence of 10 mol % catalyst and both silylated enolates, leading to **4a** and **5a** with good results (top, Scheme 2). We next investigated the synthetic versatility of the multifunctional piperidine derivatives. Straightforward acetylation of the hemiaminal hydroxyl group led to the OH-protected product. The absolute configuration of the asymmetric centers of **6a** was unequivocally assigned as (2*R*,3*R*,4*S*) by X-ray crystallographic analysis (see bottom right, Scheme 2 and Supporting Information (SI)).^15^ The same stereochemical outcome was assumed for all of the compounds included in Tables 2 and 3. Moreover, taking advantage of the enantioselective formation of the vinyl-bearing adducts **4**, tetrahydrobenzofuran derivative **7a** was obtained by straightforward alkene metathesis with styrene without erosion in the enantiomeric excess (middle, Scheme 2). The attempt of direct chlorination over hemiaminal **5a** led to a synthetically interesting acridine derivative **8a**, maintaining the enantiomeric excess of the remaining benzylic stereocenter (bottom, Scheme 2).

## Mechanistic Proposal

**Scheme 3:**
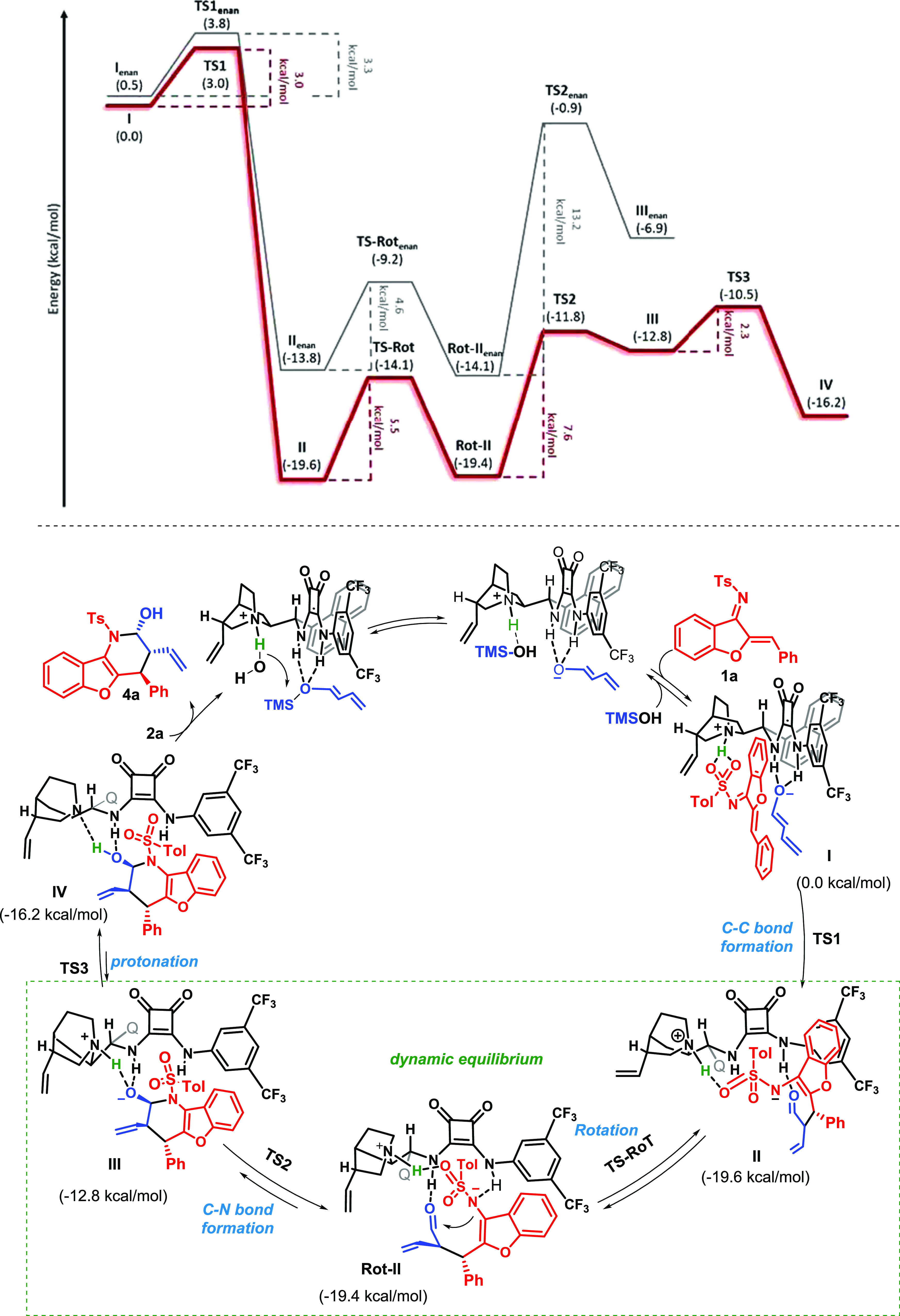
DFT M062x/6-311G* Reaction Energy Profile (Top) for Both Enantiomers (Major in Red and Minor in Gray) and Proposed Catalytic Cycle (Bottom) (Q = Quinoline)

Next, we carried out a series of density functional theory (DFT) calculations at the M06-2X/6311G*^16^ level to define a plausible mechanism for the reaction. We calculated the full experimental system considering the model substrate **1a**, nucleophile **2a**, and the catalyst **3e**. Based on previous reports,^14d,17^ the reaction begins with the hydrolysis of the isomer *E* of dienolate **2a** in the presence of catalyst **3e** and residual water present in the solvent (250 ppm, determined by Karl-Fischer titration; see Table S1 in the SI to see more details of the effect of the presence of water in the reaction). In this context, the initial process calculated in this work consists of the full catalyst interacting with the molecule of water through a hydrogen bonding with the nitrogen atom of the quinuclidine fragment of catalyst **3e**. At the same time, another hydrogen bond between the N–H of the squaramide moiety and the oxygen atom of the dienolate is present in the starting structure. The overall hydrolysis process leads to the formation of a negatively charged enolate that coordinates to the squaramide moiety of the catalyst, and at the same time, the tertiary amine of the catalyst forms the corresponding tertiary ammonium cation that interacts through hydrogen bonding with the TMSOH generated. This reversible hydrolysis reaction shows the highest barrier of the process (14.2 kcal/mol, see Scheme 3 and SI), in which reactants and products are isoenergetic (see the SI for the energy profile). The reaction proceeds then through a reversible exchange between the TMSOH and the electrophile **1a** since the tertiary ammonium fragment of the catalyst acts as a hydrogen-bond donor.

Once the dienolate is hydrolyzed and the two substrates are disposed of in the effective orientation, the reaction follows a stepwise mechanism as illustrated in Scheme 3 (red line energy profile). First, the formation of the C–C bond between the C_2_ of the dienolate **2a** (1,3-reactivity) and the benzylidenic carbon of imine **1** takes place in an exergonic process (−19.6 kcal/mol), leading to the formation of intermediate **II** with a very low kinetic barrier of 3 kcal/mol.^18^ This intermediate **II** can easily rotate with a barrier of 5.5 kcal/mol to form the isoenergetic intermediate **Rot-II**, which is correctly oriented to generate the final experimentally observed product. Subsequently, the formation of the C_aldehyde_–N_sulfonamide_ bond takes place, giving rise to intermediate **III** in an endergonic process (6.6 kcal/mol). Then, the protonation step of the corresponding cyclic intermediate **III** by the ammonium moiety of the catalyst will result in the formation of the final product **IV** through an exergonic process. An analysis of the structure of cyclic intermediate **III** reveals that the O atom of the alkoxide directly interacts with the ammonium moiety of the catalyst by a hydrogen bond (*d*_O–H_ = 1.78 Å). Such a N–H···O preorganization disposes the whole system to a protonation step without major structure rearrangement (activation barrier of 2.3 kcal/mol (**TS3**)) (see Scheme 3). Although **IV** is slightly less favorable than **Rot-II**, the isolated uncomplexed final product has been found to be significantly more stable (5.4 kcal/mol, see the SI), which can account for the driving force of the overall reaction.

**Figure 1:**
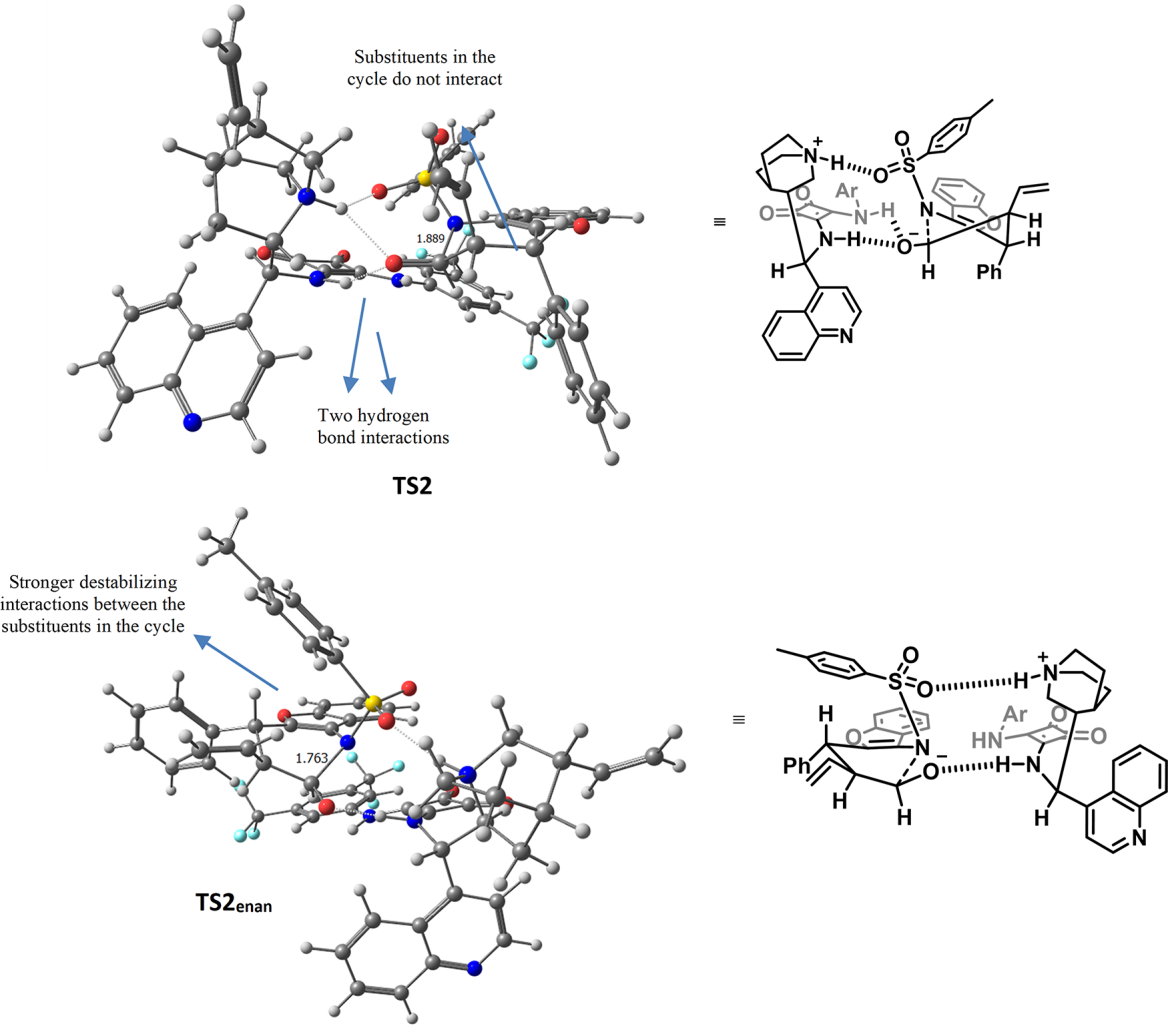
Comparison of the TS for each enantiomer: major (top) and minor (bottom).

Once the mechanistic pathway for the major enantiomer had been described, we focused on revealing where the enantioinduction of the process lies. The energy profile for the minor enantiomer (gray line, reaction profile, Scheme 3) shows similar kinetic barriers for the formation of the C–C bond and the rotation step. However, the C–N bond formation for the minor enantiomer is a more endergonic process than that in the case of the major enantiomer and has the highest energy barrier (13.2 kcal/mol). This difference in energy comes from the number of hydrogen-bond interactions that each transition state (TS) possesses and the steric hindrance present in the conformation in which the cycle is formed. In the case of the major enantiomer (**TS2**), the generated alkoxide is stabilized by the formation of two simultaneous hydrogen bonds with the squaramide moiety and the protonated quinuclidine fragment of the catalyst (see Figure 1). Meanwhile, in the case of the minor enantiomer (**TS2**_**enan**_), the alkoxide is just stabilized by one hydrogen-bond interaction; in addition, a more steric hindered cycle is being formed (substituents present gauche and *pseudo*-1,3-diaxial type interactions), which forces the TS to have a shorter distance between the C and N atoms (more product-like TS). As the energy of the barrier for **TS2** was very high (13.2 kcal/mol) and considered enough to explain the enantioinduction observed, the calculation of the protonation step from **III**_**enan**_ to **IV**_**enan**_ was not carried out.

**Scheme 4:**
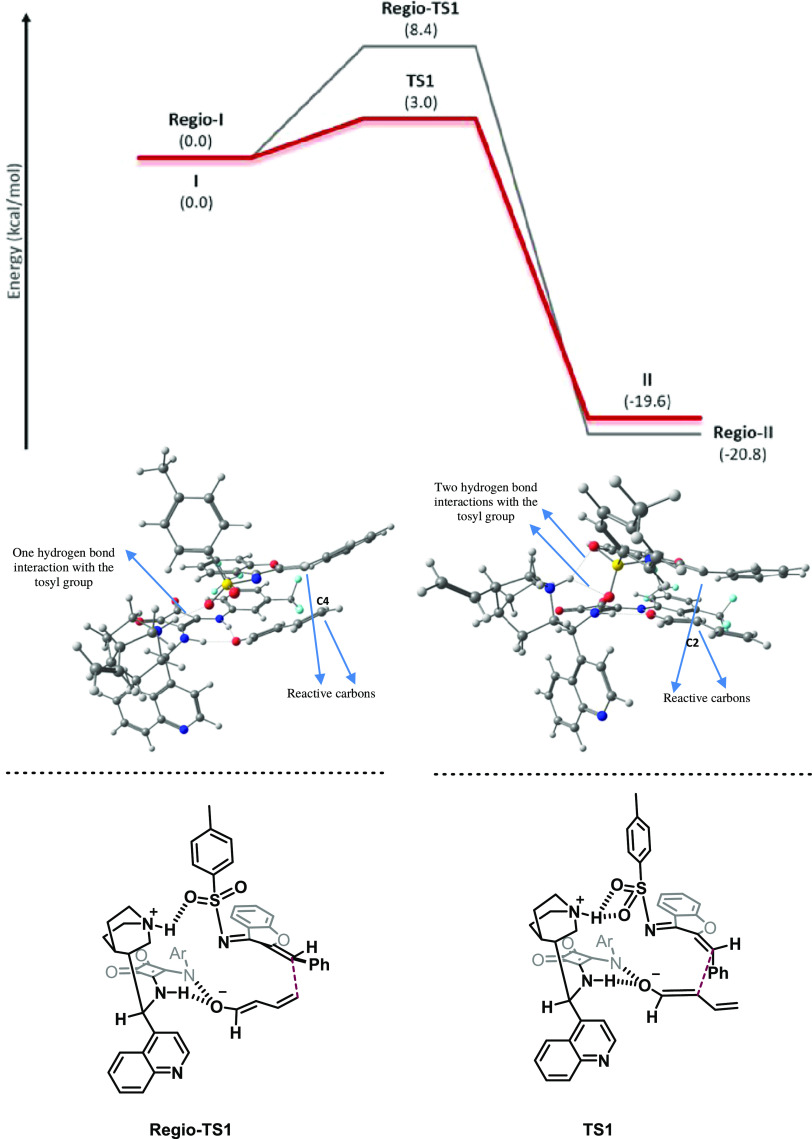
DFT M062x/6-311G* Reaction Energy Profile for the Regioselectivity Analysis in C–C Bond Formation: Reaction through the C2 of the Dienolate **2a** (Red) and Reaction through the C4 of the Dienolate **2a** (Gray) (Top) The TS structure for the gray pathway (bottom left) presents one hydrogen-bond interaction, while the transition state structure for the red pathway (bottom right) presents two.

In addition, we study the regioselectivity of the process, considering the possibility of generating the C–C bond between the C4 of the dienolate and the benzylidenic carbon of the electrophile (Scheme 4). We found that while the process from **I** to **II** shows an activation barrier of 3.0 kcal/mol, the TS calculated for this alternative pathway is 8.4 kcal/mol. Such a difference occurs due to the higher structural tension imposed by the catalyst during the C–C bond formation in **Regio-TS1**. Besides, the tosyl group of the imine is coordinated with an extra hydrogen bond to the catalyst, which stabilizes **TS1** against **Regio-TS1**. Therefore, the preference to activate the C2 carbon of the dienolate vs the C4 atom is the result of kinetic control.

**Scheme 5:**
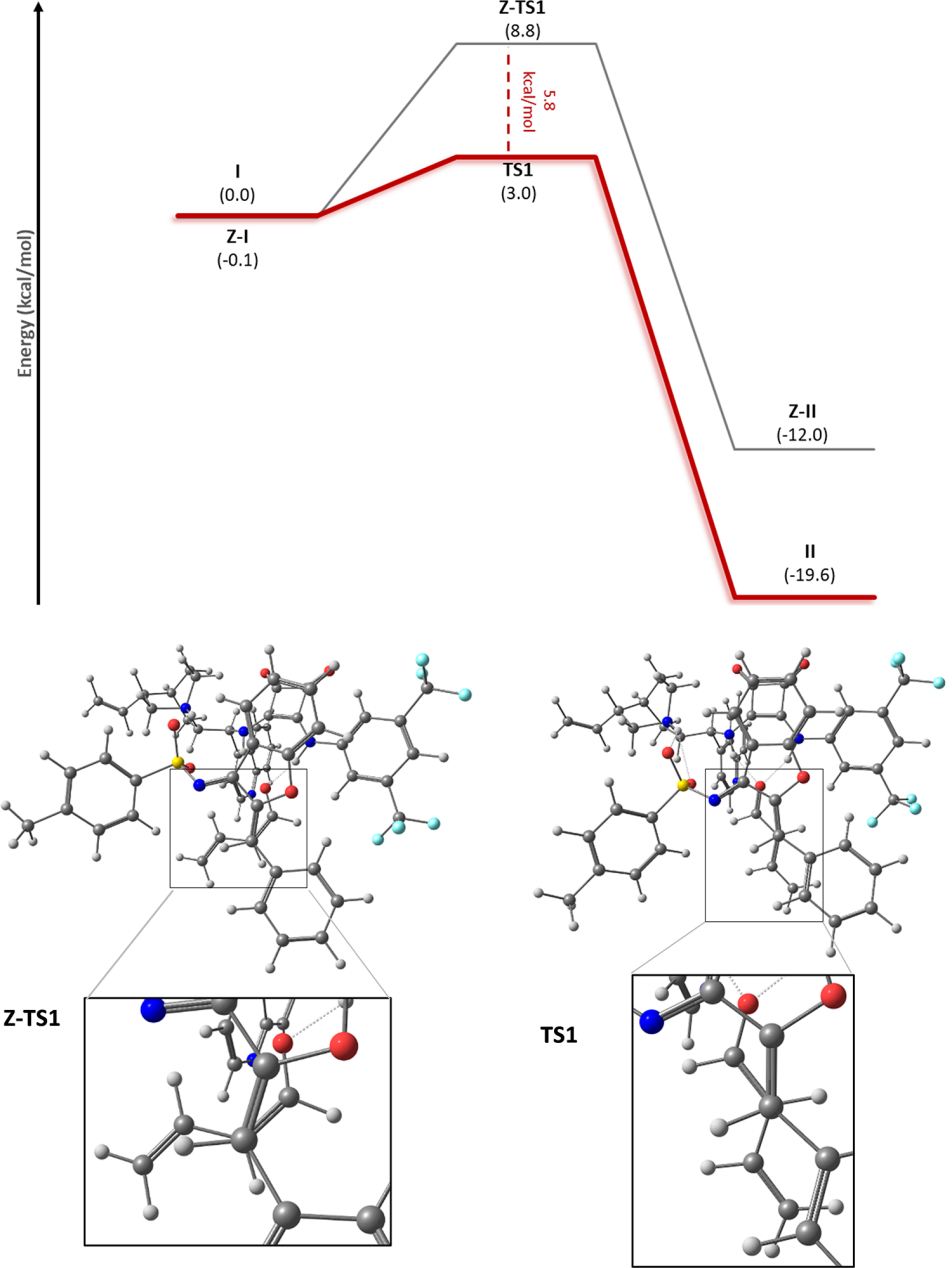
DFT M062x/6-311G* Reaction Energy Profile Showing the Hydrolysis Step and C–C Bond Formation with **1a** of Dienolate (*E*)-**2a** (Red) and Dienolate (*Z*)-**2a** (Gray); Bottom Left: Eclipsed Newman’s Projection of the New C–C Bond; Bottom Right: Staggered Newman’s Projection of the New C–C Bond

Furthermore, dienolate **2a** was used as a mixture of both isomers *E* and *Z*, out of which just (*E*)-**2a** reacted to give the final product, while (*Z*)-**2a** was observed untouched in the reaction crude. With the aim of better understanding this observation, we compared by DFT calculations the hydrolysis of (*Z*)-**2a** and (*E*)-**2a** with catalyst **3e**. The same energy profile was found in both cases (see the SI) with a difference of 1.4 kcal/mol between both energy barriers. Although the hydrolysis is less favorable for (*Z*)-**2a** than for (*E*)-**2a**, this difference in energy is not high enough to explain the lack of reactivity of (*Z*)-**2a**. Thus, we wondered if the formation of the C–C bond could be influenced by the geometry of the dienolate, and thus, we carried out DFT calculations using (*Z*)-**2a**. Again, a similar energy profile was found with the exception that the barrier for (*Z*)-**2a** (**TS1-*Z***) is 5.8 kcal/mol higher than that for (*E*)-**2a** (**TS1**). We assume that this difference in energy may come from the steric hindrance provoked by the conformation in which the new C–C bond is formed and may cause the lower reactivity of the *Z*-enolate (see Scheme 5, ***Z*-TS1** and **TS1**).

**Scheme 6:**
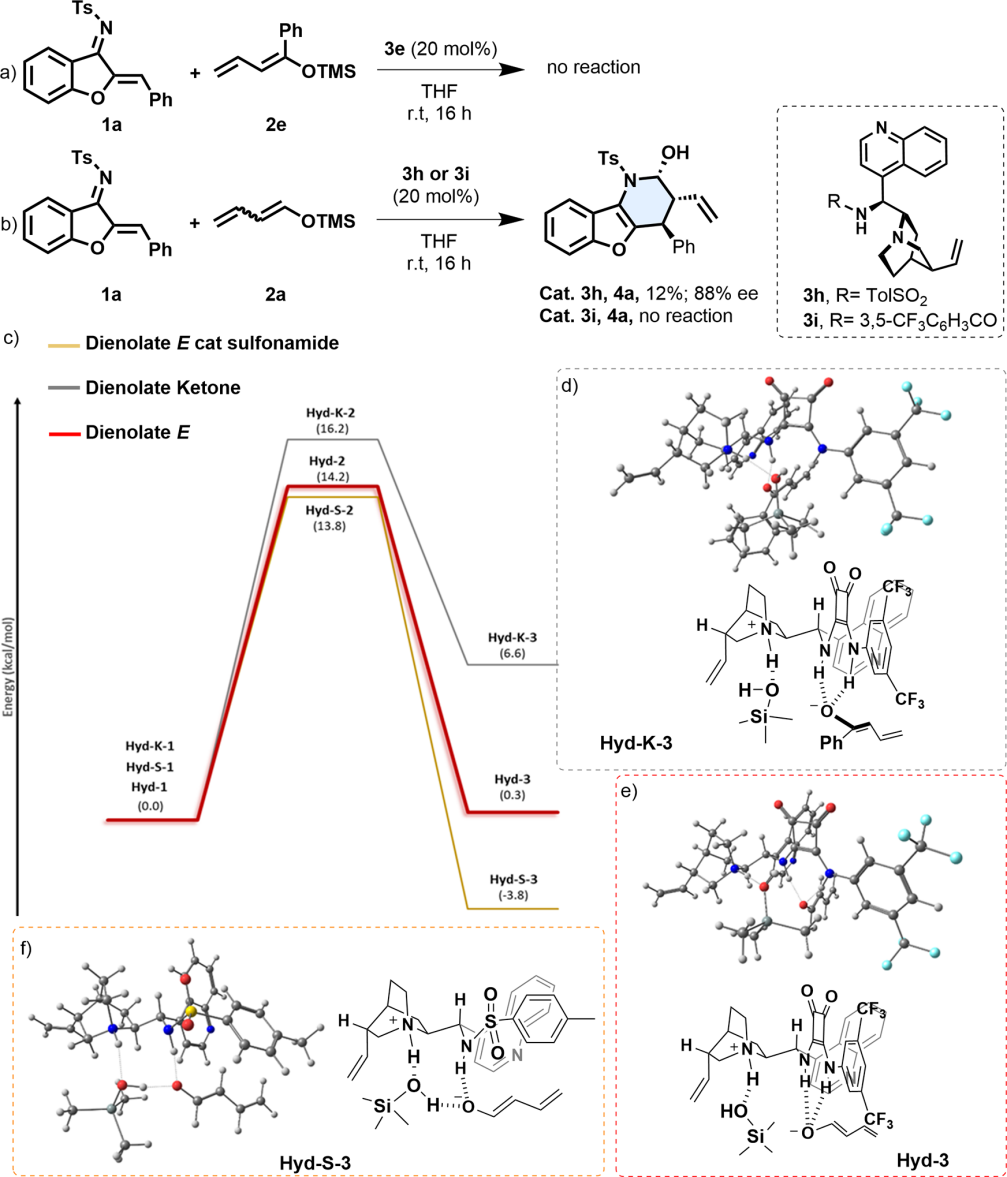
Mechanistic Proofs (a, b) and DFT M062x/6-311G* Reaction Energy Profile (c) Showing the Hydrolysis Step for (*E*)-**2a** and **2e** in the Presence of **3e** and for Dienolate (*E*)-**2a** in the Presence of **3i**; (d): **Hyd-K-3**; (e): **Hyd-3**; (f): **Hyd-S-3**

Aiming to expand the applicability and to better understand this methodology, we carried out a reaction with a ketone-derived dienolate (**2e**) (top, Scheme 6a). Interestingly, no hydrolysis of the corresponding silyl dienolate was observed in this case, and therefore no reaction was observed. When calculating the energy profile for the hydrolysis step for ketone dienolate derivative **2e** in the presence of **3e**, we found that it is an endergonic process, with a higher barrier (16.2 kcal/mol, see SI) than that for the case of the aldehyde-derived dienolate **2a** (compare gray and red energy profile lines). The endergonicity of the process might occur from the intrinsic lower stability of the ketone dienolate (compared to the analogous aldehyde dienolate) and because the squaramide moiety stabilizes the negatively charged dienolate with just one hydrogen-bond interaction, while for dienolate **2a**, it gets stabilized by two (see **Hyd-K-3** and **Hyd -3**, d and e, Scheme 6).

Lastly, regarding the role of the squaramide moiety in the hydrolysis and stabilization of the dienolates, and the correct disposition of the substrates to induce the stereoselectivity, we decided to study if other hydrogen-bond donor groups could afford similar results. This study will give a better understanding of the catalysts with the ability to activate the silyl dienol ether, thus triggering the aza-Diels–Alder reaction. In this manner, we synthesized the sulfonamide (**3h**) and amide (**3i**) analogues of catalyst **3e** and performed the reaction under the optimized reaction conditions (see top, Scheme 6b). In both cases, the major product observed was crotonaldehyde, which comes from the hydrolysis of dienolate **2a**. However, just in the reaction catalyzed by the sulfonamide catalyst **3h**, product **4a** was isolated in low yield (12%) and with lower enantioselectivity (94:6). Therefore, we decided to study by DFT calculations the hydrolysis step for dienolate (*E*)-**2a** and sulfonamide catalyst **3h**. The energy profile shows a similar energy barrier (13.8 kcal/mol) to catalyst **3e** (14.2 kcal/mol), but the process is exergonic in this case. An analysis of the structure of **Hyd-S-3** shows that the negatively charged dienolate is coordinated to the sulfonamide subunit of the catalyst as well as to the TMSOH byproduct. The distance *d*_O–H_ between the H of the OH of the TMSOH and the O of the dienolate is 1.63 Å, which suggests that a direct protonation of the dienolate is possible (before the C–C bond-forming event), giving rise to crotonaldehyde, which is the major product observed in the reaction.

## Conclusions

In conclusion, we have described an asymmetric aza-IEDDA reaction of aza-sulfonyl-1-aza-1,3-butadienes and silyl dienol ethers. The bifunctional organocatalyst triggers the reaction, enabling an ipso,α-regioselectivity of the dienophile as an alternative for the selectivity observed in the literature for β,γ-unsaturated systems. The reaction proceeded with complete diastereoselectivity and excellent enantioselectivities, featuring a reasonable substrate scope and functional group compatibility and facile scalability. In addition, the catalytic system has been implemented for the installation of an aromatic ring in the created fused piperidine ring with a similar efficiency, which has led to the desired phenyl-bearing adducts with good to excellent enantioselectivities. Furthermore, the DFT calculations carried out have demonstrated how the organocatalyst is able to activate the organosilyl species, directing the C–C bond-forming event through the 1,3 position (instead of 1,5) of the dienolate and enabling the asymmetric synthesis of these interesting benzofused derivatives.

## Supporting Information Available

Experiment details; spectroscopic data and DFT calculations; and copies of ^1^H and ^13^C spectra of all new compounds (PDF)
X-ray data for compound **6a** are included (CIF)

## Supplementary Material

cs1c03390_si_001.pdf

cs1c03390_si_002.cif

## References

[ref1] aFarina V.; Reeves J. T.; Senanayake C. H.; Song J. J. Asymmetric Synthesis of Active Pharmaceutical Ingredients. Chem. Rev. 2006, 106, 2734–2793. 10.1021/cr040700c. 16836298

[ref2] aBuonora P.; Olsen J.-C.; Oh T. Recent developments in imino Diels–Alder reactions. Tetrahedron 2001, 57, 6099–6138. 10.1016/S0040-4020(01)00438-0.

[ref3] aPng Z. M.; Zeng H.; Ye Q.; Xu J. Inverse-Electron-Demand Diels–Alder Reactions: Principles and Applications. Chem. – Asian J. 2017, 12, 2142–2159. 10.1002/asia.201700442. 28497539

[ref4] aLi J.-L.; Liu T.-Y.; Chen Y.-C. Aminocatalytic Asymmetric Diels–Alder Reactions via HOMO Activation. Acc. Chem. Res. 2012, 45, 1491–1500. 10.1021/ar3000822. 22716926

[ref5] aRong Z.-Q.; Wang M.; Chow C. H. E.; Zhao Y. A Catalyst-Enabled Diastereodivergent Aza-Diels–Alder Reaction: Complementarity of N-Heterocyclic Carbenes and Chiral Amines. Chem. – Eur. J. 2016, 22, 9483–9487. 10.1002/chem.201601626. 27219298

[ref6] aGu J.; Ma C.; Li Q.-Z.; Du W.; Chen Y.-C. β,γ-Regioselective Inverse-Electron-Demand Aza-Diels–Alder Reactions with α,β-Unsaturated Aldehydes via Dienamine Catalysis. Org. Lett. 2014, 16, 3986–3989. 10.1021/ol501814p. 25046480

[ref7] Han B.; He Z.-Q.; Li J.-L.; Li R.; Jiang K.; Liu T.-Y.; Chen Y.-C. Organocatalytic Regio- and Stereoselective Inverse-Electron-Demand Aza-Diels–Alder Reaction of α,β-Unsaturated Aldehydes and N-Tosyl-1-aza-1,3-butadienes. Angew. Chem., Int. Ed. 2009, 48, 5474–5477. 10.1002/anie.200902216. 19554588

[ref8] aMao Z.; Lin A.; Shi Y.; Mao H.; Li W.; Cheng Y.; Zhu C. Chiral Tertiary Amine Thiourea-Catalyzed Asymmetric Inverse-Electron-Demand Diels–Alder Reaction of Chromone Heterodienes Using 3-Vinylindoles as Dienophiles. J. Org. Chem. 2013, 78, 10233–10239. 10.1021/jo401592w. 24007305

[ref9] Qin J.; Zhang Y.; Liu C.; Zhou J.; Zhan R.; Chen W.; Huang H. Asymmetric Inverse-Electron-Demand Diels–Alder Reaction of β,γ-Unsaturated Amides through Dienolate Catalysis. Org. Lett. 2019, 21, 7337–7341. 10.1021/acs.orglett.9b02629. 31465234

[ref10] Li X.; Kong X.; Yang S.; Meng M.; Zhan X.; Zeng M.; Fang X. Bifunctional Thiourea-Catalyzed Asymmetric Inverse-Electron-Demand Diels–Alder Reaction of Allyl Ketones and Vinyl 1,2-Diketones via Dienolate Intermediate. Org. Lett. 2019, 21, 1979–1983. 10.1021/acs.orglett.9b00035. 30865466

[ref11] Frankowski S.; Skrzynska A.; Sieron L.; Albrecht L. Deconjugated-Ketone-Derived Dienolates in Remote, Stereocontrolled, Aromative aza-Diels-Alder Cycloaddition. Adv. Synth. Catal. 2020, 362, 2658–2665. 10.1002/adsc.202000197.

[ref12] aHiremathad A.; Patil M. R.; Chethana K. R.; Chand K.; Santos M. A.; Keri R. S. Benzofuran: an emerging scaffold for antimicrobial agents. RSC Adv. 2015, 5, 96809–96828. 10.1039/C5RA20658H.

[ref13] aRan G.-Y.; Gong M.; Yue J.-F.; Yang X.-X.; Zhou S.-L.; Du W.; Chen Y.-C. Asymmetric Cascade Assembly of 1,2-Diaza-1,3-dienes and α,β-Unsaturated Aldehydes via Dienamine Activation. Org. Lett. 2017, 19, 1874–1877. 10.1021/acs.orglett.7b00636. 28350461

[ref14] aEsteban F.; Ciesĺik W.; Arpa E. M.; Guerrero-Corella A.; Díaz-Tendero S.; Perles J.; Fernández-Salas J. A.; Fraile A.; Alemán J. Intramolecular Hydrogen Bond Activation: Thiourea-Organocatalyzed Enantioselective 1,3-Dipolar Cycloaddition of Salicylaldehyde-Derived Azomethine Ylides with Nitroalkenes. ACS Catal. 2018, 8, 1884–1890. 10.1021/acscatal.7b03553. 29527400 PMC5839603

[ref15] CCDC 2035518 (**6a**). The crystallographic data can be obtained free of charge from The Cambridge Crystallographic Data Centre.

[ref16] aZhao Y.; Truhlar D. G. The M06 suite of density functionals for main group thermochemistry, thermochemical kinetics, noncovalent interactions, excited states, and transition elements: two new functionals and systematic testing of four M06-class functionals and 12 other functionals. Theor. Chem. Acc. 2008, 120, 215–241. 10.1007/s00214-007-0310-x.

[ref17] Frías M.; Mas-Ballesté R.; Arias S.; Alvarado C.; Alemán J. Asymmetric Synthesis of Rauhut–Currier type Products by a Regioselective Mukaiyama Reaction under Bifunctional Catalysis. J. Am. Chem. Soc. 2017, 139, 672–679. 10.1021/jacs.6b07851. 28004935

[ref18] In addition, other starting structures that could afford different diastereoisomers of the final product were analyzed. In all cases the most stable intermediate corresponds to species II, confirming that the experimentally observed product comes from the thermodynamically preferred pathway (see SI).

