# Supplementary material for: Enantioselective Inverse-Electron Demand Aza-Diels–Alder Reaction: ipso,α-Selectivity of Silyl Dienol Ethers
Source: ACS Catal. 2021 Sep 15;11(19):12133–45. doi: 10.1021/acscatal.1c03390 (PMC8491166; doi:10.1021/acscatal.1c03390)
Supplement: Supplementary file 1 — cs1c03390_si_001.pdf [file cs1c03390_si_001.pdf]

Enantioselective Inverse-Electron Demand aza-Diels-Alder Reaction: *ipso,α*-Selectivity of Silyl Dienol Ethers

Víctor Laina-Martín,[a] Jorge Humbrías-Martín,[a] Rubén Mas-Ballesté,[b,c] Jose A. Fernández-Salas,[a,b]* and José Alemán[a,b]*

a. Departamento de Química Orgánica (Módulo 1), Facultad de Ciencias, Universidad Autónoma de Madrid, 28049-Madrid, Spain. e-mail: j.fernandez@uam.es; e-mail: jose.aleman@uam.es; webpage: www.uam.es/jose.aleman

b. Departamento de Química Inorgánica (Módulo 7), Facultad de Ciencias, Universidad Autónoma de Madrid, 28049-Madrid, Spain.

c. Institute for Advanced Research in Chemical Sciences (IAdChem), Facultad de Ciencias, Universidad Autónoma de Madrid, 28049-Madrid, Spain.

Contenido

1. General methods and starting materials 2

2. Optimization table of Reaction Conditions 3

3. General procedure A: Synthesis and characterization data of benzofuran-3(*2H*)-one derivatives 1 5

4. Synthesis and characterization data of trimethyl(styryloxy) silane **2b** 11

5. General procedure B: Enantioselective *aza*-IEDDA reaction. Asymmetric synthesis of cyclic hemiaminals **4** and **5** 12

6. General procedure C: Synthesis of **6a** and **7a** 30

7. General procedure D: Synthesis of **8a** 32

8. References 33

9. NMR spectra and SFC chromatograms 35

10. Single Crystal X-Ray Structure of compound **6a** 105

11. Computational studies 122

11.1. Hydrolysis of the nucleophile 122

11.2. Hydrolysis of (*Z*)-**2a** 124

11.3. Origin of the diastereoselectivity 125

11.5. Coordinates 126

1. General methods and starting materials

All dry solvents were dried using activated 4Å molecular sieves and stored under nitrogen. 4Å molecular sieves, 1.6-2.5 mm of particle size, were activated by microwave (700W) (3 x 60 sec) and subsequent cycles of vacuum/nitrogen. THF water content was determined as 250 ppm by Karl-Fisher titration. Catalyst **3a** was acquired from commercial sources and catalysts **3b**, **3c**, **3d**, **3e**, **3f**, **3g**, **3h** and **3i** were synthesized following a procedure described in the literature.[1a-b] (Buta-1,3-dien-1-yloxy)trimethylsilane **2a** was acquired from commercial sources as a 70:30 *E:Z* mixture. Silyl dienol ethers **2b** and **2c** were synthesized following a procedure described in the literature.[1c] For thin layer chromatography (TLC), silica gel plates with fluorescence indicator 254 nm were used and compounds were visualized by irradiation with UV light and/or by treatment with a solution of potassium permanganate in water followed by heating. Celite® 512 medium was used for filtrations. Flash column chromatography was performed using Geduran® Silica Gel 60 (0.040-0.063 nm). Cyclohexane and ethyl acetate for flash chromatography were acquired from commercial sources and were used without previous purification. Optical rotation was recorded in cells with 10 cm path length; the specific solvents and concentrations (in g/100 mL) are indicated. NMR spectra were acquired on a *Bruker Avance 300 MHz spectrometer*, running at 300, 75 and 282 MHz for ^{1}H, ^{13}C and ^{19}F, respectively. Chemical shifts (δ) are reported in ppm relative to residual solvent signals ($CDCl_3$, 7.26 ppm for ^{1}H NMR and 77.2 ppm for ^{13}C NMR respectively; and CD_2Cl_2, 53.8 ppm for ^{13}C NMR). ^{13}C and ^{19}F spectra were acquired on a broad band decoupled mode. The following abbreviations are used to describe peak patterns when appropriate: s (singlet), d (doublet), t (triplet), q (quartet), quint (quintet), m (multiplet), bs (broad singlet). Electrospray ionization has been used for measuring the exact mass (indicated for each case): MS (ESI) (Electrospray ionization mass spectroscopy) was acquired with an *Agilent Technologies 6120 Quadrupole LC/MS*. In this technique, *MassWorks software ver. 4.0.0.0 (Cerno Bioscience*) was used for the formula identification. *MassWorks* is a MS calibration software which calibrates for isotope profile as well as for mass accuracy, allowing highly accurate comparisons between calibrated and theoretical spectra.[2]

Enantiomeric excesses were determined in a Supercritical Fluid Chromatography (SFC) with chiral columns. The chromatograms were acquired with an *Agilent Technologies 1260 Infinity* with a *SFC module* and a UV-vis detector. The chiral columns used were: Chiralpak IA, IB-3, IC, ID-3, IG-3 (see in each case).

2. Optimization of the Reaction Conditions

Table S1.

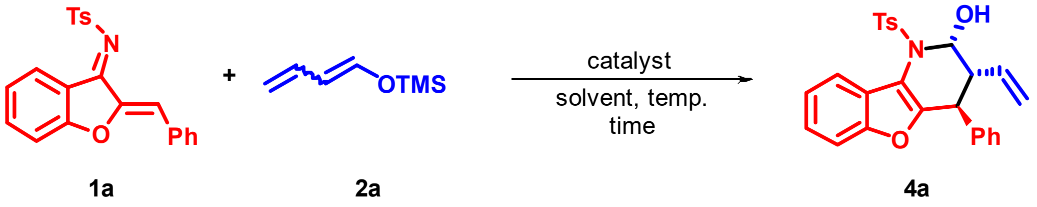

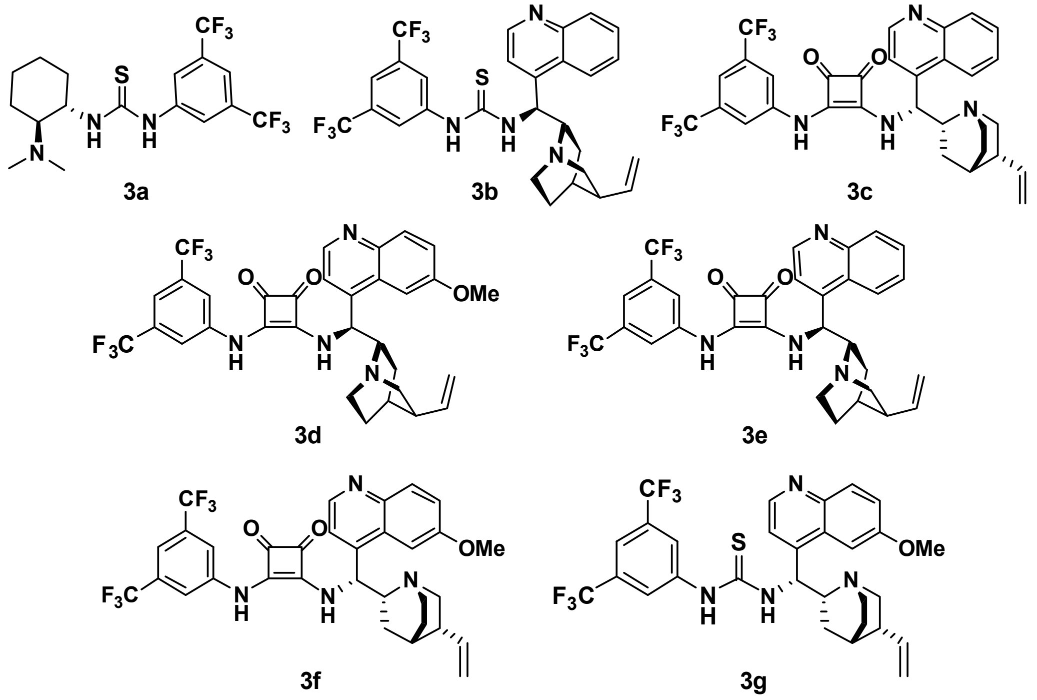

Entry[a]	*Catalyst*	*Solvent*	*T* (°C)	*t* (h)	*Solvent* (mL)	*H_2O* (equiv.)	*Yield* (%)	*dr*	*ee*
1	**3a**	THF	25	16	0.3	3	56	>20:1	76
2	**3a**	DCM	25	16	0.3	3	50	>20:1	75
3	**3a**	Toluene	25	16	0.3	3	28	>20:1	38
4	**3a**	DCE	25	16	0.3	3	54	>20:1	64
5	**3a**	Et_2O	25	16	0.3	3	44	>20:1	52
6	**3g**	THF	25	16	0.3	3	51	>20:1	-82
7	**3b**	THF	25	16	0.3	3	48	>20:1	84

8	**3f**	THF	25	16	0.3	3	29	>20:1	-94
9	**3e**	THF	25	16	0.3	3	42	>20:1	92
10	**3e**	THF	25	64	0.3	3	45	>20:1	92
11	**3d**	THF	25	16	0.3	3	41	>20:1	86
12	**3c**	THF	25	16	0.3	3	41	>20:1	-82
13	**3e**	THF	25	16	0.3	0	42	>20:1	92
14	**3e**	THF	25	40	0.3	0	40	>20:1	94
15	**3e**	THF	25	16	0.3	6	56	>20:1	63
16	**3e**	THF	25	16	0.1	0	43	>20:1	90
17	**3e**	THF	25	16	0.5	0	32	>20:1	91
18	**3e**	MTBE	25	16	0.3	0	33	>20:1	65
19	**3e**	Dioxane	25	16	0.3	0	10	>20:1	nd
***20**[b]*	**3e**	**THF**	**25**	**16**	**0.3**	**0**	**67**	**>20:1**	**94**
21	**3e**	THF	40	16	0.3	0	56	>20:1	93
22[b]	**3e**	THF	40	16	0.3	0	51	>20:1	93
***23**[c]*	**3e**	**THF**	**25**	**16**	**0.3**	**0**	**78**	**>20:1**	**88**

[a] Standard conditions: 0.05 mmol of *N*-((*E*)-2-((*Z*)-benzylidene)benzofuran-3(*2H*)-ylidene)-4-methylbenzenesulfonamide, 0.15 mmol of a 70:30 *E:Z* mixture of buta-1,3-dien-1-yloxy)trimethylsilane and 0.01 mmol of catalyst in 0.3 mL of solvent were used. Diastereomeric ratio (*dr*) was measured by ^{1}H-NMR of the crude mixture and enantiomeric excesses were measured by SFC chromatography. [b] 0.30 mmol of a 70:30 *E:Z* mixture of buta-1,3-dien-1-yloxy)trimethylsilane were used. [c] 0.30 mmol of a 65:35 *Z:E* mixture of trimethyl(styryloxy) silane were used.

3. General procedure A: Synthesis and characterization data of benzofuran-3(2*H*)-one derivatives 1

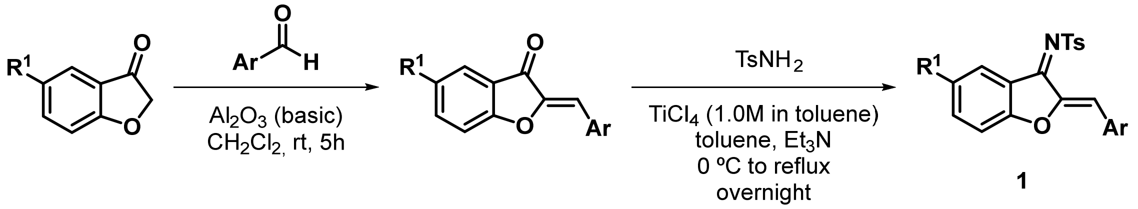

They were prepared following a modified procedure described in the literature:[3] A round bottom flask was charged with a magnetic stirrer and the corresponding benzofuran-3(2*H*)-one (7 mmol, 1.0 equiv.) was dissolved in CH_2Cl_2 (30 mL). Then, the corresponding aromatic aldehyde (8.4 mmol, 1.2 equiv.) and Al_2O_3 (activated basic, 21.4 g, 210 mmol, 30 equiv.) were sequentially added and the reaction mixture was stirred for 5 hours at room temperature. Finally, the reaction mixture was filtered through Celite® and the filtrate was concentrated under reduced pressure. The final products were used for the next step without further purification.

A two-neck round bottom flask was charged with a magnetic stirrer, the corresponding enone (3.0 mmol, 1.0 equiv.) and *p*-toluenesulfonamide (514 mg, 3.0 mmol, 1.0 equiv.) under nitrogen atmosphere. Then, toluene (30 mL) was added and the reaction mixture was cooled to 0°C. Triethylamine (0.9 mL, 6 mmol, 2.0 equiv.) and $TiCl_4$ (1.0M in toluene, 3.0 mL, 3 mmol, 1.0 equiv.) were added dropwise. Finally, the reaction mixture was stirred overnight at reflux. The solution was then cooled to room temperature. The crude mixture was diluted with ethyl acetate, washed with brine (3 x 30 mL), dried over $MgSO_4$, filtered and the solvent was evaporated under reduced pressure. The crude mixture was purified by column chromatography using silica gel and the eluent indicated in each case.

***N*-((*E*)-2-((*Z*)-Benzylidene)benzofuran-3(2*H*)-ylidene)-4-methylbenzenesulfonamide (1a)**[4]

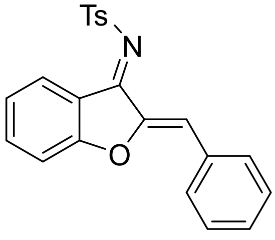

Following general procedure A, benzofuran-3(2*H*)-one (940 mg, 7 mmol) and benzaldehyde (0.9 mL, 8.4 mmol), gave **1a** (81% yield) as a yellow solid. Eluent: cyclohexane: ethyl acetate from 99:1 to 85:15. The ^{1}H-NMR is in accordance with the literature.

^{1}H NMR: δ 8.79 (d, *J* = 8.0 Hz, 1H), 8.08 – 7.95 (m, 2H), 7.90 (dd, *J* = 7.4, 2.0 Hz, 2H), 7.75 – 7.63 (m, 1H), 7.51 – 7.28 (m, 7H), 7.12 (s, 1H), 2.48 (s, 3H) ppm.

N-((E)-2-((Z)-4-Bromobenzylidene)benzofuran-3(2H)-ylidene)-4-methylbenzenesulfonamide (1b)[4]

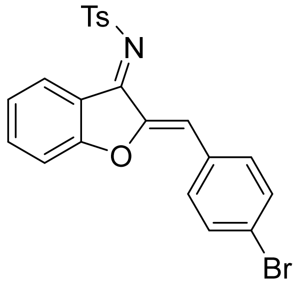

Following general procedure A, benzofuran-3(*2H*)-one (940 mg, 7 mmol) and 4-bromobenzaldehyde (1.55 g, 8.4 mmol), gave **1b** (74% yield) as a yellow solid. Eluent: cyclohexane: ethyl acetate from 99:1 to 85:15. The ^{1}H-NMR is in accordance with the literature.

^{1}H NMR: δ 8.78 (d, *J* = 8.1 Hz, 1H), 7.99 (d, *J* = 8.3 Hz, 2H), 7.78 – 7.66 (m, 3H), 7.60 – 7.54 (m, 2H), 7.41 – 7.36 (m, 2H), 7.35 – 7.29 (m, 2H), 7.29 – 7.25 (m, 1H), 7.02 (s, 1H), 2.48 (s, 3H) ppm.

N-((E)-2-((Z)-4-Fluorobenzylidene)benzofuran-3(2H)-ylidene)-4-methylbenzenesulfonamide (1c)

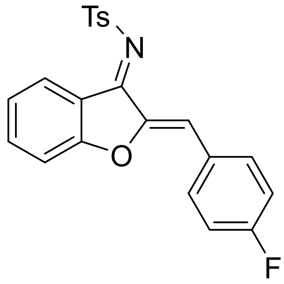

Following general procedure A, benzofuran-3(*2H*)-one (940 mg, 7 mmol) and 4-fluorobenzaldehyde (0.9 mL, 8.4 mmol), gave **1c** (67% yield) as a yellow solid. Eluent: cyclohexane: ethyl acetate from 98:2 to 85:15.

^{1}H NMR: δ 8.77 (d, *J* = 7.5 Hz, 1H), 7.99 (d, *J* = 8.3 Hz, 2H), 7.90 – 7.83 (m, 2H), 7.71 – 7.63 (m, 1H), 7.37 (d, *J* = 7.8 Hz, 2H), 7.33 – 7.23 (m, 2H), 7.15 – 7.03 (m, 3H), 2.46 (s, 3H) ppm.

^{13}C NMR: δ 165.1, 164.8, 163.8 (d, *J* = 253.4 Hz), 149.4 (d, *J* = 2.5 Hz), 143.6, 139.1, 137.8, 133.9 (d, *J* = 8.5 Hz, 2C), 131.2, 129.6 (2C), 128.9 (d, *J* = 3.4 Hz), 127.2 (2C), 124.0, 118.5, 116.4 (d, *J* = 21.8 Hz, 2C), 114.5, 112.4, 21.8 ppm.

^{19}F NMR: δ -108.11 ppm.

HRMS (ESI$^+$): calculated for $C_{22}H_{17}SFO_3N$ [M+H]$^+$: 394.0908; found: 394.0918.

4-Methyl-N-((E)-2-((Z)-4-nitrobenzylidene)benzofuran-3(2H)-ylidene)benzenesulfonamide (1d)

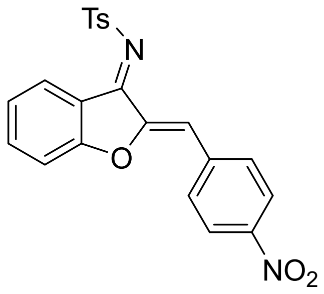

Following general procedure A, benzofuran-3(*2H*)-one (940 mg, 7 mmol) and 4-nitrobenzaldehyde (1.27 g, 8.4 mmol), gave **1d** (69% yield) as a yellow solid. Eluent: cyclohexane: ethyl acetate from 98:2 to 85:15.

^{1}H NMR: δ 8.78 (d, *J* = 8.0 Hz, 1H), 8.25 (d, *J* = 8.9 Hz, 2H), 8.04 – 7.96 (m, 4H), 7.72 (t, *J* = 7.2 Hz, 1H), 7.43 – 7.28 (m, 4H), 7.03 (s, 1H), 2.49 (s, 3H) ppm.

^{13}C NMR: δ 164.9, 164.8, 151.9, 147.8, 144.0, 138.7, 138.6, 138.3, 131.9 (2C), 131.6, 129.8 (2C), 127.3 (2C), 124.6, 124.2 (2C), 118.0, 112.5, 111.4, 21.8 ppm.

HRMS (ESI$^+$): calculated for $C_{22}H_{17}SO_5N_2$ [M+H]$^+$: 421.0853; found: 421.0815.

4-Methyl-*N*-((*E*)-2-((*Z*)-4-(trifluoromethyl)benzylidene)benzofuran-3(*2H*)-ylidene) benzenesulfonamide (1e)[5]

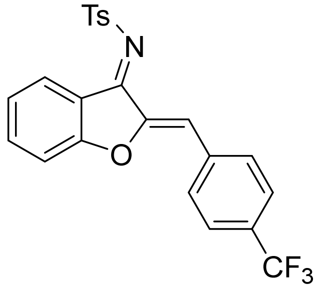

Following general procedure A, benzofuran-3(*2H*)-one (940 mg, 7 mmol) and 4-(trifluoromethyl)benzaldehyde (1.1 mL, 8.4 mmol), gave **1e** (74% yield) as a yellow solid. Eluent: cyclohexane: ethyl acetate from 99:1 to 85:15. The ^{1}H-NMR is in accordance with the literature.

^{1}H NMR: δ 8.79 (d, *J* = 8.0 Hz, 1H), 8.16 – 7.94 (m, 4H), 7.77 – 7.63 (m, 3H), 7.53 – 7.22 (m, 4H), 7.06 (s, 1H), 2.47 (s, 3H) ppm.

***N*-((*E*)-2-((*Z*)-4-Cyanobenzylidene)benzofuran-3(*2H*)-ylidene)-4-methylbenzenesulfonamide (1f)**

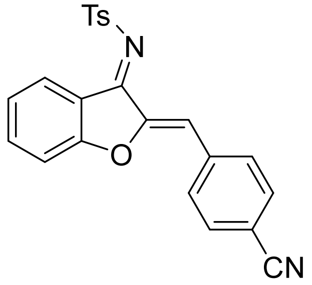

Following general procedure A, benzofuran-3(*2H*)-one (940 mg, 7 mmol) and 4-cyanobenzaldehyde (1.11 g, 8.4 mmol), gave **1f** (72% yield) as a yellow solid. Eluent: cyclohexane: ethyl acetate from 99:1 to 85:15.

^{1}H NMR: δ 8.78 (d, *J* = 8.1 Hz, 1H), 7.97 (dd, *J* = 11.3, 8.4 Hz, 4H), 7.72 – 7.66 (m, 3H), 7.42 – 7.28 (m, 4H), 7.00 (s, 1H), 2.48 (s, 3H) ppm.

^{13}C NMR: δ 164.9, 151.6, 143.9, 138.7, 138.3, 136.9 (2C), 132.6 (2C), 131.7 (2C), 131.5, 129.7 (2C), 127.3 (2C), 124.5, 118.7, 118.1, 112.9, 112.5, 112.1, 21.8 ppm.

HRMS (ESI$^+$): calculated for $C_{23}H_{17}SO_3N_2$ [M+H]$^+$: 401.0954; found: 401.1010.

N-((E)-2-((Z)-3-Chlorobenzylidene)benzofuran-3(2H)-ylidene)-4-methylbenzenesulfonamide (1g)[5]

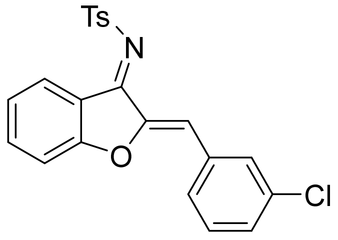

Following general procedure A, benzofuran-3(*2H*)-one (940 mg, 7 mmol) and 3-chlorobenzaldehyde (1.0 mL, 8.4 mmol), gave **1g** (74% yield) as a yellow solid. Eluent: cyclohexane: ethyl acetate from 99:1 to 85:15. The ^{1}H-NMR is in accordance with the literature.

^{1}H NMR: δ 8.76 (d, *J* = 8.0 Hz, 1H), 8.04 – 7.94 (m, 2H), 7.85 (brs, 1H), 7.73 – 7.62 (m, 2H), 7.44 – 7.22 (m, 6H), 6.95 (s, 1H), 2.46 (s, 3H) ppm.

N-((E)-2-((Z)-2-Fluorobenzylidene)benzofuran-3(2H)-ylidene)-4-methylbenzenesulfonamide (1h)

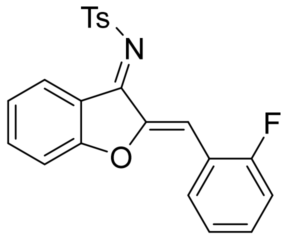

Following general procedure A, benzofuran-3(*2H*)-one (940 mg, 7 mmol) and 2-fluorobenzaldehyde (0.9 mL, 8.4 mmol), gave **1h** (65% yield) as a yellow solid. Eluent: cyclohexane: ethyl acetate from 99:1 to 85:15.

^{1}H NMR: δ 8.78 (d, *J* = 8.0 Hz, 1H), 8.33 – 8.25 (m, 1H), 7.99 (d, *J* = 8.2 Hz, 2H), 7.72 – 7.58 (m, 1H), 7.41 – 7.16 (m, 7H), 7.06 (t, *J* = 9.3 Hz, 1H), 2.45 (s, 3H) ppm.

^{13}C NMR: δ 165.0, 164.8, 161.5 (d, *J* = 255.0 Hz), 150.6, 143.6, 138.9, 137.8, 132.0, 131.9, 131.3, 129.6 (2C), 127.2 (2C), 124.8 (d, *J* = 3.8 Hz), 124.0, 120.8 (d, *J* = 11.4 Hz), 118.3, 115.7 (d, *J* = 21.8 Hz), 112.4, 106.3, 21.5 ppm.

^{19}F NMR: δ -113.7 ppm.

HRMS (ESI$^+$): calculated for $C_{22}H_{17}SFO_3N$ $[M+H]^+$: 394.0908; found: 394.0926.

N-((E)-2-((Z)-2-Bromobenzylidene)benzofuran-3(2H)-ylidene)-4-methylbenzenesulfonamide (1i)[4]

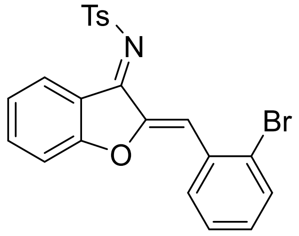

Following general procedure A, benzofuran-3(*2H*)-one (940 mg, 7 mmol) and 2-bromobenzaldehyde (1.55 g, 8.4 mmol), gave **1m** (62% yield) as a dark yellow solid. Eluent: cyclohexane: ethyl acetate from 99:1 to 80:20. The ^{1}H-NMR is in accordance with the literature.

^{1}H NMR: δ 8.78 (d, *J* = 8.1 Hz, 1H), 7.99 (d, *J* = 8.3 Hz, 2H), 7.78 – 7.66 (m, 3H), 7.60 – 7.54 (m, 2H), 7.41 – 7.36 (m, 2H), 7.35 – 7.29 (m, 2H), 7.29 – 7.25 (m, 1H), 7.02 (s, 1H), 2.48 (s, 3H) ppm.

N-((E)-2-((Z)-3-Methoxybenzylidene)benzofuran-3(2H)-ylidene)-4-methyl benzenesulfonamide (1j)

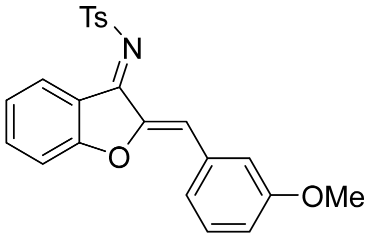

Following general procedure A, benzofuran-3(*2H*)-one (940 mg, 7 mmol) and 3-methoxybenzaldehyde (1.0 mL, 8.4 mmol), gave **1i** (71% yield) as a yellow solid. Eluent: cyclohexane: ethyl acetate from 98:2 to 80:20.

^{1}H NMR: δ 8.78 (d, *J* = 7.4 Hz, 1H), 8.00 (d, *J* = 8.3 Hz, 2H), 7.73 – 7.60 (m, 1H), 7.49 – 7.23 (m, 7H), 7.07 (s, 1H), 6.97 – 6.92 (m, 1H), 3.84 (s, 3H), 2.46 (s, 3H) ppm.

^{13}C NMR: δ 165.3, 164.9, 160.0, 149.9, 143.5, 139.2, 137.8, 133.7, 131.3, 130.0, 129.6 (2C), 127.2 (2C), 124.6, 124.0, 118.5, 116.7, 116.4, 115.7, 112.5, 55.5, 21.8 ppm.

HRMS (ESI^{+}): calculated for $C_{23}H_{20}SO_4N$ $[M+H]^+$: 406.1108; found: 406.1125.

4-Methyl-N-((*2Z,3E*)-2-(naphthalen-2-ylmethylene)benzofuran-3(*2H*)-ylidene) benzenesulfonamide (1k)[4]

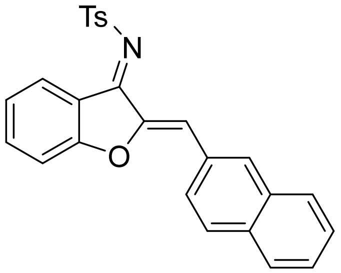

Following general procedure A, benzofuran-3(*2H*)-one (940 mg, 7 mmol) and 2-naphthaldehyde (1.31 g, 8.4 mmol), gave **1j** (78% yield) as a yellow solid. Eluent: cyclohexane: ethyl acetate from 99:1 to 85:15. The ^{1}H-NMR is in accordance with the literature.

^{1}H NMR: δ 8.81 (d, *J* = 8.1 Hz, 1H), 8.30 (s, 1H), 8.09 – 8.00 (m, 3H), 7.92 – 7.79 (m, 3H), 7.70 (t, *J* = 7.6 Hz, 1H), 7.58 – 7.47 (m, 2H), 7.39 (m, 3H), 7.34 – 7.26 (m, 2H), 2.49 (s, 3H) ppm.

***N*-((*E*)-2-((*Z*)-Benzylidene)-5-bromobenzofuran-3(*2H*)-ylidene)-4-methylbenzenesulfonamide (1l)[6]**

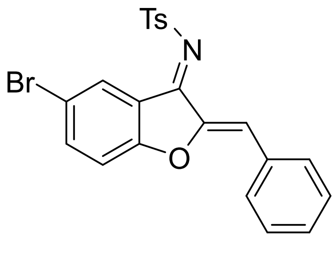

Following general procedure A, 5-bromobenzofuran-3(*2H*)-one (1.48 g, 7 mmol) and benzaldehyde (0.9 mL, 8.4 mmol), gave **1k** (77% yield) as a yellow solid. Eluent: cyclohexane: ethyl acetate from 99:1 to 85:15. The ^{1}H-NMR is in accordance with the literature.

^{1}H NMR: δ 8.91 (s, 1H), 7.99 (d, *J* = 8.3 Hz, 2H), 7.93 – 7.81 (m, 2H), 7.77 (dd, *J* = 8.8, 2.1 Hz, 1H), 7.51 – 7.34 (m, 5H), 7.23 (d, *J* = 8.7 Hz, 1H), 7.15 (s, 1H), 2.48 (s, 3H) ppm.

***N*-((*E*)-2-((*Z*)-benzylidene)-6-bromobenzofuran-3(*2H*)-ylidene)-4-methylbenzenesulfonamide (1m)**

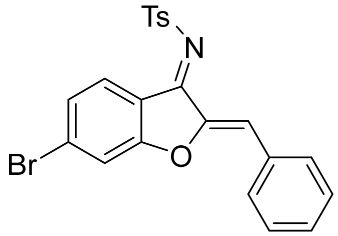

Following general procedure A, 6-bromobenzofuran-3(*2H*)-one (1.49 g, 7 mmol) and benzaldehyde (0.9 mL, 8.4 mmol), gave **1m** (54% yield) as a dark yellow solid. Eluent: cyclohexane: ethyl acetate from 98:2 to 80:20.

^{1}H NMR: δ 8.65 (d, *J* = 8.6 Hz, 1H), 7.99 (d, *J* = 8.3 Hz, 2H), 7.90 – 7.81 (m, 2H), 7.55 (d, *J* = 1.6 Hz, 1H), 7.47 – 7.35 (m, 6H), 7.12 (s, 1H), 2.48 (s, 3H) ppm.

^{13}C NMR: δ 164.8, 164.0, 149.7, 143.7, 138.9, 132.5, 132.3, 132.1, 131.9 (2C), 130.7, 129.7 (2C), 129.2 (2C), 127.6, 127.2 (2C), 117.6, 116.5, 116.1, 21.8 ppm.

***N*-((*E*)-2-((*Z*)-4-Methoxybenzylidene)benzofuran-3(*2H*)-ylidene)-4-methyl benzenesulfonamide (1n)[4]**

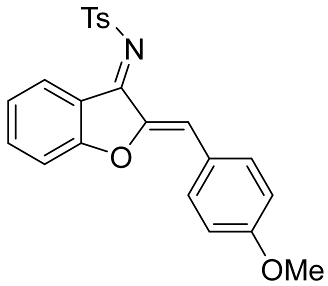

Following general procedure A, benzofuran-3(*2H*)-one (940 mg, 7 mmol) and 4-methoxybenzaldehyde (1.0 mL, 8.4 mmol), gave **1l** (58% yield) as a yellow solid. Eluent: cyclohexane: ethyl acetate from 98:2 to 80:20. The ^{1}H-NMR is in accordance with the literature.

^{1}H NMR: δ 8.77 (d, *J* = 8.1 Hz, 1H), 8.00 (d, *J* = 8.2 Hz, 2H), 7.88 (d, *J* = 8.8 Hz, 2H), 7.67 (t, *J* = 7.7 Hz, 1H), 7.37 (d, *J* = 8.1 Hz, 2H), 7.32 (d, *J* = 8.7 Hz, 2H), 7.14 (s, 1H), 6.97 (d, *J* = 8.8 Hz, 2H), 3.88 (s, 3H), 2.47 (s, 3H) ppm.

4. Synthesis and characterization data of trimethyl(styryloxy) silane 2b

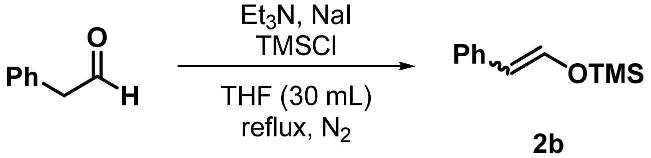

It was prepared following a modified procedure described in the literature:[7] To a previously oven-dried round bottom flask charged with a condenser, a magnetic stirrer and anhydrous sodium iodide (105 mg, 0.7 mmol, 0.08 equiv.) were added underand three cycles vacuum-nitrogen were perfmormed. Then, anhydrous THF (30 mL), Et_3N (1.4 mL, 10 mmol, 1.1 equiv.) and TMSCl (1.3 mL, 10 mmol, 1.1 equiv.) were sequentially added and the mixture was heated to 70 °C. Later, phenylacetaldehyde (1.0 mL, 9 mmol, 1.0 equiv.) was added under nitrogen atmosphere and the reaction mixture was refluxed for 5 hours. Finally, the solution was cooled to room temperature and the crude mixture was diluted with pentane, washed with water (3 x 30 mL), dried over $MgSO_4$, filtered and the solvent was evaporated under reduced pressure. Purification by Kugelrohr distillation (b.p. 110-115 °C, 25 mmHg) gave **2d** as a colorless liquid (78% yield, 65:35 *Z:E* mixture). The ^{1}H-NMR is in accordance with the literature.[8]

^{1}H-NMR: δ 7.62 (d, *J* = 7.1 Hz, 2H, *Z*), 7.41 – 7.09 (m, 8H, *Z* and *E*), 6.97 (d, *J* = 12.4 Hz, 1H, *E*), 6.41 (d, *J* = 6.6 Hz, 1H, *Z*), 6.03 (d, *J* = 12.4 Hz, 1H, *E*), 5.34 (d, *J* = 6.6 Hz, 1H, *Z*), 0.28 (s, 9H, *Z*), 0.27 (s, 9H, *E*) ppm.

5. General procedure B: Enantioselective *aza*-IEDDA reaction. Asymmetric synthesis of cyclic hemiaminals 4 and 5

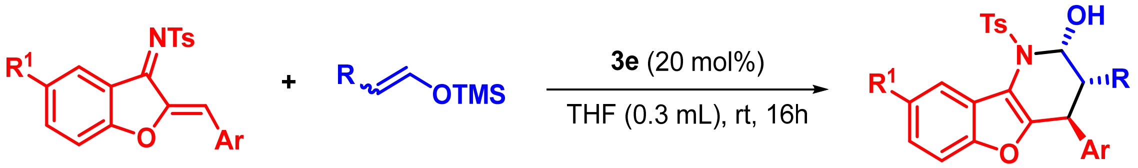

Catalyst **3e** (6.3 mg, 0.01 mmol, 0.2 equiv.) and the corresponding benzofuran-3(*2H*)-one derivative **1** (0.05 mmol, 1.0 eq.) were dissolved in THF (0.3 mL) in a vial. Then, the corresponding silyl (di)enol ether **2** (0.3 mmol, 6.0 equiv. of an *E:Z* mixture) was added and the reaction was stirred overnight at room temperature. After that, the mixture was concentrated *in vacuo.* Finally, the crude mixture was purified by flash column chromatography using silica gel and eluting with the solvent indicated in each case.

(*2R,3R,4S*)-4-Phenyl-1-tosyl-3-vinyl-1,2,3,4-tetrahydrobenzofuro[3,2-*b*]pyridin-2-ol (4a)

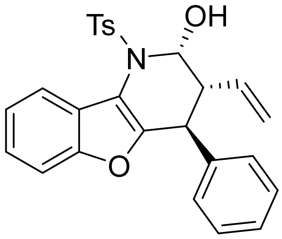

Following general procedure B, *N*-((*E*)-2-((*Z*)-benzylidene) benzofuran-3(*2H*)-ylidene)-4-methylbenzenesulfonamide **1a** (18.8 mg, 0.05 mmol) and silyl dienol ether **2a** (53 µL, 0.3 mmol) after 16h at room temperature, gave **4a** (67% yield, >20:1 *dr*) as a colorless oil. Eluent: cyclohexane: ethyl acetate from 97:3 to 85:15. **$[\alpha]^{25}_{D}$** = +251.3 (*c* 0.75, $CHCl_3$).

^{1}H NMR: δ 8.24 – 8.16 (m, 1H), 7.62 – 7.54 (m, 2H), 7.35 – 7.28 (m, 5H), 7.20 – 7.09 (m, 3H), 6.58 – 6.51 (m, 2H), 5.78 – 5.62 (m, 2H), 4.98 (dd, *J* = 10.2, 1.5 Hz, 1H), 4.61 – 4.54 (m, 1H), 3.94 (d, *J* = 11.0 Hz, 1H), 2.73 (brs, 1H), 2.44 (s, 3H), 1.94 – 1.84 (m, 1H) ppm.

^{13}C NMR: δ 154.5, 148.6, 144.8, 138.4, 135.1, 134.1, 130.1 (2C), 128.6 (2C), 128.4 (2C), 128.2 (2C), 127.5, 124.8, 124.6, 123.3, 122.5, 119.3, 115.8, 111.5, 84.0, 49.0, 41.8, 21.8 ppm.

HRMS (ESI$^+$): calculated for $C_{26}H_{27}SO_4N_2$ $[M+NH_4]^+$: 463.1686; found: 463.1691.

The enantiomeric excess was determined by SFC using a Chiralpak IA column [CO_2/MeOH from 95:5 to 60:40 in 8 min, flow rate 3.0 mL/min], τ_{minor} = 4.65 min, τ_{major} = 5.07 min (97:3 *er*).

The reaction was also performed on a 1.0 mmol scale. In this case, and following general procedure B, catalyst **3e** (63 mg, 0.1 mmol), *N*-((*E*)-2-((*Z*)-benzylidene) benzofuran-3(*2H*)-ylidene)-4-methylbenzenesulfonamide **1a** (380 mg, 1.0 mmol) and silyl dienol ether **2a** (1 mL, 6.0 mmol) after 48h at room temperature, gave **4a** (260 mg, 57% yield, >20:1 *dr*) as a yellowish

solid. Eluent: cyclohexane: ethyl acetate from 97:3 to 85:15. The enantiomeric excess was determined by SFC using a Chiralpak IA column [CO_2/MeOH from 95:5 to 60:40 in 8 min, flow rate 3.0 mL/min], τ_{minor} = 4.63 min, τ_{major} = 5.03 min (96.5:3.5 *er*).

(*2R,3R,4S*)-4-(4-Bromophenyl)-1-tosyl-3-vinyl-1,2,3,4-tetrahydrobenzofuro[3,2-*b*]pyridin-2-ol (4b)

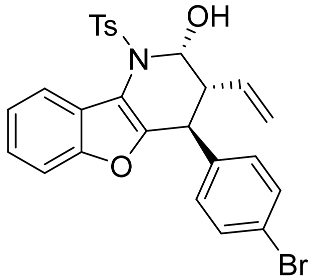

Following general procedure B, *N*-((*E*)-2-((*Z*)-4-bromobenzylidene) benzofuran-3(*2H*)-ylidene)-4-methylbenzenesulfonamide **1b** (22.7 mg, 0.05 mmol) and silyl dienol ether **2a** (53 µL, 0.3 mmol) after 16h at room temperature, gave **4b** (57% yield, >20:1 *dr*) as a colorless oil. Eluent: cyclohexane: ethyl acetate from 97:3 to 85:15. **$[\alpha]^{25}_D$** = +279.2 (*c* 0.66, $CHCl_3$).

^{1}H NMR: δ 8.24 – 8.13 (m, 1H), 7.57 (d, *J* = 7.9 Hz, 2H), 7.39 – 7.18 (m, 8H), 6.43 (d, *J* = 8.4 Hz, 1H), 5.78 – 5.57 (m, 2H), 5.00 (d, *J* = 10.0 Hz, 1H), 4.57 (d, *J* = 17.1 Hz, 1H), 3.90 (d, *J* = 11.0 Hz, 1H), 2.69 (brs, 1H), 2.44 (s, 3H), 1.82 (t, *J* = 10.0 Hz, 1H) ppm.

^{13}C NMR: δ 154.6, 147.8, 144.9, 137.5, 134.7, 134.1, 131.6 (2C), 130.3 (2C), 130.1 (2C), 128.2 (2C), 125.0, 124.5, 123.4, 122.6, 121.3, 119.7, 116.1, 111.5, 83.8, 49.0, 41.3, 21.8 ppm.

HRMS (ESI$^+$): calculated for $C_{26}H_{26}SBrO_4N_2$ $[M+NH_4]^+$: 541.0791; found: 541.0830.

The enantiomeric excess was determined by SFC using a Chiralpak IB-3 column [CO_2/MeOH from 95:5 to 60:40 in 8 min, flow rate 2.0 mL/min], τ_{minor} = 5.20 min, τ_{major} = 5.84 min (97.5:2.5 *er*).

(*2R,3R,4S*)-4-(4-Fluorophenyl)-1-tosyl-3-vinyl-1,2,3,4-tetrahydrobenzofuro[3,2-*b*]pyridin-2-ol (4c)

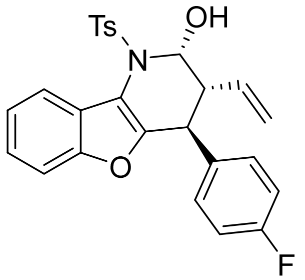

Following general procedure B, *N*-((*E*)-2-((*Z*)-4-fluorobenzylidene) benzofuran-3(*2H*)-ylidene)-4-methylbenzenesulfonamide **1c** (19.7 mg, 0.05 mmol) and silyl dienol ether **2a** (53 µL, 0.3 mmol) after 16h at room temperature, gave **4c** (62% yield, >20:1 *dr*) as a colorless oil. Eluent: cyclohexane: ethyl acetate from 97:3 to 85:15. **$[\alpha]^{25}_D$** = +252.8 (*c* 0.94, $CHCl_3$).

^{1}H NMR: δ 8.22 – 8.16 (m, 1H), 7.58 (d, *J* = 8.3 Hz, 2H), 7.36 – 7.28 (m, 5H), 6.82 (t, *J* = 8.6 Hz, 2H), 6.54 – 6.47 (m, 2H), 5.75 – 5.61 (m, 2H), 5.00 (dd, *J* = 10.2, 1.4 Hz, 1H), 4.56 (d, *J* = 17.1 Hz, 1H), 3.93 (d, *J* = 11.0 Hz, 1H), 2.67 (brs, 1H), 2.45 (s, 3H), 1.86 – 1.76 (m, 1H) ppm.

^{13}C NMR: δ 162.1 (d, *J* = 245.9 Hz), 154.5, 148.2, 144.9, 134.9, 134.1, 130.2 (d, *J* = 7.9 Hz, 2C), 130.1 (2C), 130.0, 128.2 (2C), 124.9, 124.6, 123.4, 122.5, 119.5, 116.0, 115.3 (d, *J* = 21.4 Hz, 2C), 111.5, 83.9, 49.2, 41.0, 21.8 ppm.

^{19}F NMR: δ -115.2 ppm.

HRMS (ESI$^+$): calculated for $C_{26}H_{26}SFO_4N_2$ $[M+NH_4]^+$: 481.1592; found: 481.1559.

The enantiomeric excess was determined by SFC using a Chiralpak IA column [CO_2/MeOH from 95:5 to 60:40 in 8 min, flow rate 3.0 mL/min], τ_{minor} = 4.34 min, τ_{major} = 4.85 min (97.5:2.5 *er*).

(*2R,3R,4S*)-4-(4-Nitrophenyl)-1-tosyl-3-vinyl-1,2,3,4-tetrahydrobenzofuro[3,2-*b*]pyridin-2-ol (4d)

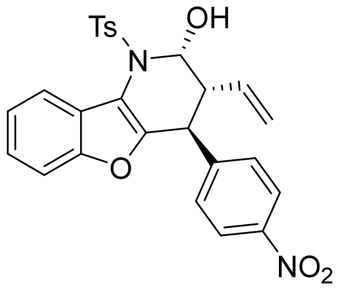

Following general procedure B, 4-methyl-*N*-((*E*)-2-((*Z*)-4-nitrobenzylidene) benzofuran-3(*2H*)-ylidene)benzenesulfonamide **1d** (21.0 mg, 0.05 mmol) and silyl dienol ether **2a** (53 μL, 0.3 mmol) after 16h at room temperature, gave **4d** (64% yield, >20:1 *dr*) as a yellow oil. Eluent: cyclohexane: ethyl acetate from 97:3 to 80:20. **$[\alpha]^{25}_D$** = -14.5 (*c* 0.72, $CHCl_3$).

^{1}H NMR: δ 8.26 – 8.15 (m, 1H), 8.00 (d, *J* = 8.7 Hz, 2H), 7.59 (d, *J* = 8.3 Hz, 2H), 7.39 – 7.28 (m, 5H), 6.72 (d, *J* = 8.7 Hz, 2H), 5.81 – 5.59 (m, 2H), 5.01 (d, *J* = 10.1 Hz, 1H), 4.52 (d, *J* = 17.1 Hz, 1H), 4.07 (d, *J* = 11.0 Hz, 1H), 2.84 (brs, 1H), 2.47 (s, 3H), 1.88 – 1.77 (m, 1H) ppm.

^{13}C NMR: δ 154.6, 147.4, 146.7, 146.4, 145.2, 134.3, 134.0, 130.1 (2C), 129.5 (2C), 128.2 (2C), 125.3, 124.3, 123.7 (2C), 123.6, 122.7, 120.1, 116.6, 111.5, 83.6, 49.2, 41.7, 21.9 ppm.

HRMS (ESI$^+$): calculated for $C_{26}H_{26}SO_6N_3$ $[M+NH_4]^+$: 508.1537; found: 508.1565.

The enantiomeric excess was determined by SFC using a Chiralpak IA column [CO_2/MeOH from 95:5 to 60:40 in 8 min, flow rate 3.0 mL/min], τ_{minor} = 5.15 min, τ_{major} = 5.85 min (96.5:3.5 *er*).

(*2R,3R,4S*)-1-Tosyl-4-(4-(trifluoromethyl)phenyl)-3-vinyl-1,2,3,4-tetrahydrobenzofuro[3,2-*b*]pyridin-2-ol (4e)

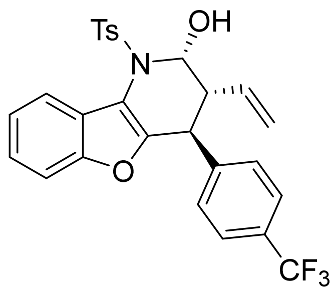

Following general procedure B, 4-methyl-*N*-((*E*)-2-((*Z*)-4-(trifluoromethyl)benzylidene) benzofuran-3(*2H*)-ylidene)benzenesulfonamide **1e** (22.2 mg, 0.05 mmol) and silyl dienol ether **2a** (53 µL, 0.3 mmol) after 16h at room temperature, gave **4e** (55% yield, >20:1 *dr*) as a yellow oil. Eluent: cyclohexane: ethyl acetate from 97:3 to 85:15. $[\alpha]^{25}_D$ = +230.0 (*c* 0.81, $CHCl_3$).

^{1}H NMR: δ 8.24 – 8.16 (m, 1H), 7.58 (d, *J* = 8.2 Hz, 2H), 7.38 (d, *J* = 8.1 Hz, 2H), 7.34 – 7.29 (m, 5H), 6.67 (d, *J* = 8.2 Hz, 2H), 5.78 – 5.62 (m, 2H), 5.01 (dd, *J* = 10.2, 1.4 Hz, 1H), 4.55 (d, *J* = 17.1 Hz, 1H), 4.01 (d, *J* = 11.1 Hz, 1H), 2.69 (brs, 1H), 2.45 (s, 3H), 1.85 (t, *J* = 10.2 Hz, 1H) ppm.

^{1}H NMR: δ 154.6, 147.5, 145.0, 142.7, 134.5, 134.0, 130.1 (2C), 129.8 (q, *J* = 32.5 Hz), 129.0 (2C), 128.2 (2C), 125.3 (q, *J* = 3.8 Hz, 2C), 125.1, 124.4, 124.2 (q, *J* = 272.2 Hz), 123.5, 122.6, 119.8, 116.3, 111.5, 83.8, 49.1, 41.6, 21.8 ppm.

^{19}F NMR: δ -62.6 ppm.

HRMS (ESI$^+$): calculated for $C_{27}H_{26}SF_3O_4N_2$ $[M+NH_4]^+$: 531.1560; found: 531.1536.

The enantiomeric excess was determined by SFC using a Chiralpak IA column [CO_2/MeOH from 95:5 to 60:40 in 8 min, flow rate 3.0 mL/min], τ_{minor} = 3.70 min, τ_{major} = 4.14 min (97:3 *er*).

4-((*2R,3R,4S*)-2-Hydroxy-1-tosyl-3-vinyl-1,2,3,4-tetrahydrobenzofuro[3,2-*b*]pyridin-4-yl)benzonitrile (4f)

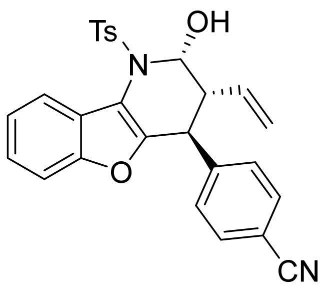

Following general procedure B, *N*-((*E*)-2-((*Z*)-4-cyanobenzylidene) benzofuran-3(*2H*)-ylidene)-4-methylbenzenesulfonamide **1f** (20.0 mg, 0.05 mmol) and silyl dienol ether **2a** (53 µL, 0.3 mmol) after 16h at room temperature, gave **4f** (59% yield, >20:1 *dr*) as a yellow oil. Eluent: cyclohexane: ethyl acetate from 97:3 to 85:15. $[\alpha]^{25}_D$ = +141.8 (*c* 0.96, $CHCl_3$).

^{1}H NMR: δ 8.23 – 8.17 (m, 1H), 7.58 (d, *J* = 8.3 Hz, 2H), 7.54 – 7.36 (m, 3H), 7.35 – 7.28 (m, 4H), 6.67 (d, *J* = 8.3 Hz, 2H), 5.79 – 5.60 (m, 2H), 5.01 (d, *J* = 10.0 Hz, 1H), 4.53 (d, *J* = 17.1 Hz, 1H), 4.01 (d, *J* = 11.0 Hz, 1H), 2.83 (brs, 1H), 2.46 (s, 3H), 1.82 (t, *J* = 10.0 Hz, 1H) ppm.

^{13}C NMR: δ 154.6, 146.9, 145.0, 144.3, 134.4, 134.1, 132.2 (2C), 130.1 (2C), 129.5 (2C), 128.2 (2C), 127.3, 125.2, 124.3, 123.5, 122.7, 120.0, 118.7, 116.6, 111.5, 83.7, 49.1, 41.9, 21.8 ppm.

HRMS (ESI+): calculated for $C_{27}H_{26}SO_4N_3$ $[M+NH_4]^+$: 488.1639; found: 488.1633.

The enantiomeric excess was determined by SFC using a Chiralpak IC column [CO_2/MeOH from 95:5 to 60:40 in 8 min, flow rate 3.0 mL/min], τ_{major} = 4.87 min, τ_{minor} = 5.38 min (96.5:3.5 *er*).

(*2R,3R,4S*)-4-(3-Chlorophenyl)-1-tosyl-3-vinyl-1,2,3,4-tetrahydrobenzofuro[3,2-*b*]pyridin-2-ol (4g)

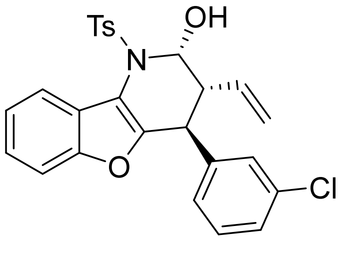

Following general procedure B, *N*-((*E*)-2-((*Z*)-3-chlorobenzylidene) benzofuran-3(*2H*)-ylidene)-4-methylbenzenesulfonamide **1g** (20.3 mg, 0.05 mmol) and silyl dienol ether **2a** (53 µL, 0.3 mmol) after 16h at room temperature, gave **4g** (58% yield, >20:1 *dr*) as a colorless oil. Eluent: cyclohexane: ethyl acetate from 98:2 to 85:15. $[\alpha]^{25}_D$ = +268.3 (*c* 0.82, $CHCl_3$).

^{1}H NMR: δ 8.26 – 8.14 (m, 1H), 7.58 (d, *J* = 8.3 Hz, 2H), 7.40 – 7.29 (m, 5H), 7.20 – 7.05 (m, 2H), 6.67 (dt, *J* = 7.2, 1.6 Hz, 1H), 6.45 – 6.36 (m, 1H), 5.76 – 5.58 (m, 2H), 5.00 (d, *J* = 10.2 Hz, 1H), 4.56 (dt, *J* = 17.1, 1.1 Hz, 1H), 3.92 (d, *J* = 11.0 Hz, 1H), 2.76 (brs, 1H), 2.45 (s, 3H), 1.79 – 1.68 (m, 1H) ppm.

^{13}C NMR: δ 154.6, 147.7, 145.2, 140.5, 134.7, 134.5, 134.0, 130.3 (2C), 129.6, 128.0 (2C), 127.9 (2C), 127.8, 125.0, 124.6, 123.4, 122.7, 119.7, 116.2, 111.6, 83.8, 48.9, 41.5, 21.9 ppm.

HRMS (ESI+): calculated for $C_{26}H_{26}SO_4N_2Cl$ $[M+NH_4]^+$: 497.1296; found: 497.1368.

The enantiomeric excess was determined by SFC using a Chiralpak IA column [CO_2/MeOH from 95:5 to 60:40 in 8 min, flow rate 3.0 mL/min], τ_{minor} = 4.90 min, τ_{major} = 5.32 min (97:3 *er*).

(*2R,3R,4S*)-4-(2-Fluorophenyl)-1-tosyl-3-vinyl-1,2,3,4-tetrahydrobenzofuro[3,2-*b*]pyridin-2-ol (4h)

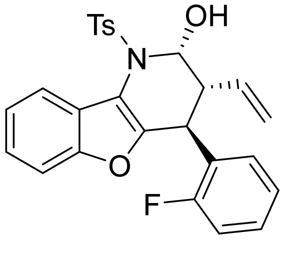

Following general procedure B, *N*-((*E*)-2-((*Z*)-2-fluorobenzylidene) benzofuran-3(*2H*)-ylidene)-4-methylbenzenesulfonamide **1h** (19.7 mg, 0.05 mmol) and silyl dienol ether **2a** (53 µL, 0.3 mmol) after 16h at room temperature, gave **4h** (57% yield, >20:1 *dr*) as a yellow oil. Eluent: cyclohexane: ethyl acetate from 97:3 to 85:15. $[\alpha]^{25}_D$ = +164.9 (*c* 0.53, $CHCl_3$).

^{1}H NMR: δ 8.23 – 8.17 (m, 1H), 7.58 (d, *J* = 8.3 Hz, 2H), 7.36 – 7.28 (m, 5H), 7.21 – 7.13 (m, 1H), 7.01 – 6.93 (m, 1H), 6.85 (td, *J* = 7.5, 1.3 Hz, 1H), 6.19 (t, *J* = 7.5 Hz, 1H), 5.82 – 5.68 (m, 2H), 4.97

(dd, *J* = 10.2, 1.5 Hz, 1H), 4.58 (d, *J* = 17.1 Hz, 1H), 4.41 (d, *J* = 11.2 Hz, 1H), 2.64 (brs, 1H), 2.43 (s, 3H), 1.88 (t, *J* = 10.2 Hz, 1H) ppm.

^{13}C NMR: δ 161.3 (d, *J* = 247.3 Hz), 154.3, 147.4, 144.6, 134.8, 134.0, 129.9 (2C), 129.0 (d, *J* = 3.4 Hz), 128.9 (d, *J* = 8.3 Hz), 128.0 (2C), 125.3 (d, *J* = 13.9 Hz), 124.6, 124.4, 123.8 (d, *J* = 3.6 Hz), 123.2, 122.3, 118.9, 116.0, 115.5 (d, *J* = 22.4 Hz), 111.3, 83.7, 48.4, 34.2, 21.6 ppm.

^{19}F NMR: δ -117.4 ppm.

HRMS (ESI$^+$): calculated for $C_{26}H_{26}SFO_4N_2$ $[M+NH_4]^+$: 481.1592; found: 481.1584.

The enantiomeric excess was determined by SFC using a Chiralpak IC column [CO_2/MeOH from 95:5 to 60:40 in 8 min, flow rate 3.0 mL/min], τ_{major} = 3.69 min, τ_{minor} = 4.13 min (95:5 *er*).

(*2R,3R,4S*)-4-(2-Bromophenyl)-1-tosyl-3-vinyl-1,2,3,4-tetrahydrobenzofuro[3,2-*b*]pyridin-2-ol (4i)

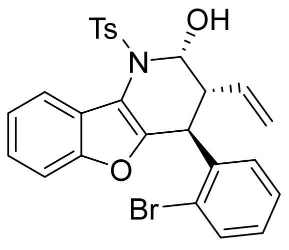

Following general procedure B, *N*-((*E*)-2-((*Z*)-2-bromobenzylidene)benzofuran-3(*2H*)-ylidene)-4-methylbenzene sulfonamide **1i** (22.7 mg, 0.05 mmol) and silyl dienol ether **2a** (53 µL, 0.3 mmol) after 16h at room temperature, gave **4i** (58% yield, >20:1 *dr*) as a yellow oil. Eluent: cyclohexane: ethyl acetate from 97:3 to 83:17. **$[\alpha]^{25}_D$** = +180.3 (*c* 0.38, $CHCl_3$).

^{1}H NMR: δ 8.23 – 8.18 (m, 1H), 7.60 (d, *J* = 8.1 Hz, 2H), 7.51 (d, *J* = 8.3 Hz, 1H), 7.36 – 7.28 (m, 5H), 7.04 (t, *J* = 7.5 Hz, 1H), 6.96 – 6.88 (m, 1H), 5.95 – 5.78 (m, 2H), 5.73 (s, 1H), 4.96 (d, *J* = 10.0 Hz, 1H), 4.74 (d, *J* = 10.0 Hz, 1H), 4.55 (d, *J* = 17.2 Hz, 1H), 2.74 (s, 1H), 2.45 (s, 3H), 1.88 (t, *J* = 9.9 Hz, 1H) ppm.

^{13}C NMR: δ 154.1, 147.7, 144.5, 138.8, 134.2, 133.8, 132.7, 132.6, 129.7 (2C), 128.5, 128.2, 127.9 (2C), 127.0, 124.5, 124.1, 123.0, 122.1, 118.7, 111.2, 83.7, 31.8, 22.5, 21.4, 14.0 ppm.

HRMS (ESI$^+$): calculated for $C_{26}H_{26}BrSO_4N_2$ $[M+NH_4]^+$: 541.0791; found: 591.0811.

The enantiomeric excess was determined by SFC using a Chiralpak IA column [CO_2/MeOH from 95:5 to 60:40 in 8 min, flow rate 3.0 mL/min], τ_{minor} = 5.33 min, τ_{major} = 5.67 min (98:2 *er*).

(*2R,3R,4S*)-4-(3-Methoxyphenyl)-1-tosyl-3-vinyl-1,2,3,4-tetrahydrobenzofuro[3,2-*b*]pyridin-2-ol (4j)

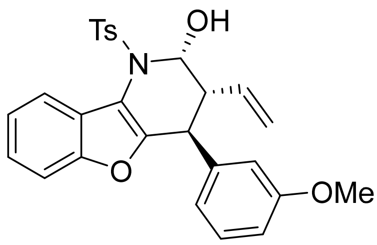

Following general procedure B, *N*-((*E*)-2-((*Z*)-3-methoxybenzylidene) benzofuran-3(*2H*)-ylidene)-4-methylbenzenesulfonamide **1i** (20.3 mg, 0.05 mmol) and silyl dienol ether **2a** (53 µL, 0.3 mmol) after 16h at room temperature, gave **4i** (57% yield, >20:1 *dr*) as a yellow oil. Eluent: cyclohexane: ethyl acetate from 97:3 to 80:20. **[α]$^{25}_{D}$** = +233.9 (*c* 0.85, $CHCl_3$).

^{1}H NMR: 8.22 – 8.16 (m, 1H), 7.57 (d, *J* = 8.3 Hz, 2H), 7.35 – 7.28 (m, 5H), 7.12 – 7.02 (m, 1H), 6.72 (ddd, *J* = 8.3, 2.6, 1.0 Hz, 1H), 6.22 – 6.16 (m, 1H), 6.13 – 6.11 (m, 1H), 5.76 – 5.62 (m, 2H), 5.02 – 4.96 (m, 1H), 4.66 – 4.58 (m, 1H), 3.91 (d, *J* = 11.1 Hz, 1H), 3.73 (s, 3H), 2.67 – 2.64 (m, 1H), 2.43 (s, 3H), 1.88 (t, *J* = 9.9 Hz, 1H) ppm.

^{13}C NMR: δ 159.7, 154.6, 148.5, 145.0, 139.9, 135.1, 134.0, 130.2 (2C), 129.3, 128.1 (2C), 124.8, 124.6, 123.3, 122.5, 121.2, 119.2, 115.7, 115.3, 111.8, 111.6, 84.0, 55.3, 48.7, 41.7, 21.7 ppm.

HRMS (ESI$^+$): calculated for $C_{27}H_{29}SO_5N_2$ $[M+NH_4]^+$: 493.1792; found: 493.1791.

The enantiomeric excess was determined by SFC using a Chiralpak IC column [CO_2/MeOH from 95:5 to 60:40 in 8 min, flow rate 3.0 mL/min], τ_{major} = 4.15 min, τ_{minor} = 4.70 min (97:3 *er*).

(*2R,3R,4S*)-4-(Naphthalen-2-yl)-1-tosyl-3-vinyl-1,2,3,4-tetrahydrobenzofuro[3,2-*b*]pyridin-2-ol (4k)

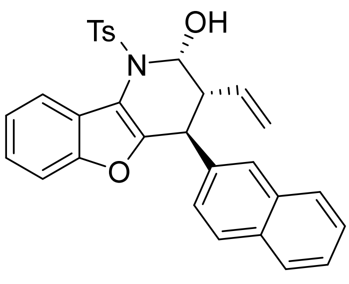

Following general procedure B, 4-methyl-*N*-((*2Z,3E*)-2-(naphthalen-2-ylmethylene) benzofuran-3(*2H*)-ylidene)benzenesulfonamide **1k** (21.3 mg, 0.05 mmol) and silyl dienol ether **2a** (53 µL, 0.3 mmol) after 16h at room temperature, gave **4k** (67% yield, >20:1 *dr*) as a yellow oil. Eluent: cyclohexane: ethyl acetate from 98:2 to 85:15. **[α]$^{25}_{D}$** = +281.2 (*c* 1.36, $CHCl_3$).

^{1}H NMR: δ 8.23 (d, *J* = 7.3 Hz, 1H), 7.79 – 7.74 (m, 1H), 7.88 – 7.61 (m, 3H), 7.57 (d, *J* = 8.5 Hz, 1H), 7.48 – 7.41 (m, 2H), 7.38 – 7.27 (m, 5H), 7.22 (brs, 1H), 6.50 (dd, *J* = 8.5, 1.8 Hz, 1H), 5.81 – 5.67 (m, 2H), 4.94 (d, *J* = 10.3 Hz, 1H), 4.59 – 4.50 (m, 1H), 4.12 (d, *J* = 11.1 Hz, 1H), 2.79 – 2.71 (m, 1H), 2.46 (s, 3H), 1.99 (d, *J* = 11.1 Hz, 1H) ppm.

^{13}C NMR: δ 154.6, 148.6, 144.9, 135.8, 135.0, 134.2, 133.4, 132.8, 130.2 (2C), 128.2 (2C), 128.1, 127.9, 127.8, 127.7, 126.3, 126.1, 126.0, 124.8, 124.6, 123.3, 122.5, 119.4, 115.9, 111.5, 84.0, 48.9, 41.9, 21.9 ppm.

HRMS (ESI$^+$): calculated for $C_{30}H_{29}SO_4N_2$ $[M+NH_4]^+$: 513.1843; found: 513.1858.

The enantiomeric excess was determined by SFC using a Chiralpak IA column [CO_2/MeOH from 95:5 to 60:40 in 8 min, flow rate 3.0 mL/min], τ_{minor} = 5.93 min, τ_{major} = 6.48 min (97:3 *er*).

(*2R,3R,4S*)-8-Bromo-4-phenyl-1-tosyl-3-vinyl-1,2,3,4-tetrahydrobenzofuro[3,2-*b*]pyridin-2-ol (4l)

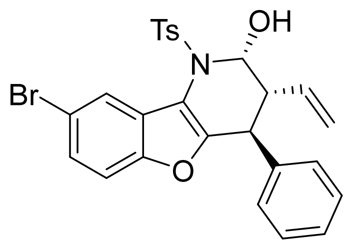

Following general procedure B, *N*-((*E*)-2-((*Z*)-benzylidene)-5-bromobenzofuran-3(*2H*)-ylidene)-4-methylbenzenesulfonamide **1l** (22.7 mg, 0.05 mmol) and silyl dienol ether **2a** (53 µL, 0.3 mmol) after 16h at room temperature, gave **4l** (60% yield, >20:1 *dr*) as a colorless oil. Eluent: cyclohexane: ethyl acetate from 97:3 to 85:15. **[α]^{25}$_D$** = +253.8 (*c* 1.33, $CHCl_3$).

^{1}H NMR: δ 8.35 – 8.30 (m, 1H), 7.59 (d, *J* = 8.8 Hz, 2H), 7.40 – 7.30 (m, 3H), 7.22 – 7.11 (m, 4H), 6.57 – 6.49 (m, 2H), 5.76 – 5.60 (m, 2H), 4.98 (d, *J* = 10.0 Hz, 1H), 4.57 (d, *J* = 17.1 Hz, 1H), 3.93 (d, *J* = 11.0 Hz, 1H), 2.86 (brs, 1H), 2.46 (s, 3H), 1.87 (t, *J* = 10.0 Hz, 1H) ppm.

^{13}C NMR: δ 153.3, 150.0, 145.1, 138.0, 134.8, 133.9, 130.1 (2C), 128.6 (2C), 128.5 (2C), 128.2 (2C), 127.8, 127.6, 126.5, 125.1, 119.4, 116.6, 115.3, 113.0, 83.8, 48.9, 41.7, 21.8 ppm.

HRMS (ESI$^+$): calculated for $C_{26}H_{26}SBrO_4N_2$ $[M+NH_4]^+$: 541.0791; found: 541.0808.

The enantiomeric excess was determined by SFC using a Chiralpak IA column [CO_2/MeOH from 95:5 to 60:40 in 8 min, flow rate 3.0 mL/min], τ_{minor} = 5.00 min, τ_{major} = 5.52 min (96:4 *er*).

(*2R,3R,4S*)-7-Bromo-4-phenyl-1-tosyl-3-vinyl-1,2,3,4-tetrahydrobenzofuro[3,2-*b*]pyridin-2-ol (4m)

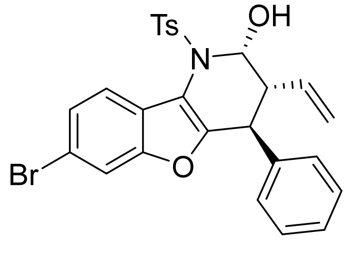

Following general procedure B, *N*-((*E*)-2-((*Z*)-benzylidene) 6-bromobenzofuran-3(*2H*)-ylidene)-4-methylbenzenesulfonamide **1m** (22.7 mg, 0.05 mmol) and silyl dienol ether **2a** (53 µL, 0.3 mmol) after 16h at room temperature, gave **4m** (53% yield, >20:1 *dr*) as a yellow oil. Eluent: cyclohexane: ethyl acetate from 97:3 to 83:17. **[α]^{25}$_D$** = +160.9 (*c* 0.31, $CHCl_3$).

^{1}H NMR: δ 8.06 (d, *J* = 8.5 Hz, 1H), 7.56 (d, *J* = 8.2 Hz, 2H), 7.50 – 7.39 (m, 2H), 7.32 (d, *J* = 8.2 Hz, 2H), 7.22 – 7.10 (m, 3H), 6.56 – 6.48 (m, 2H), 5.76 – 5.61 (m, 2H), 4.99 (dd, *J* = 10.1, 1.5 Hz, 1H), 4.57 (dt, *J* = 17.3, 1.1 Hz, 1H), 3.91 (d, *J* = 11.0 Hz, 1H), 2.72 (brs, 1H), 2.45 (s, 3H), 1.86 (t, *J* = 10.1 Hz, 1H) ppm.

^{13}C NMR: δ 154.7, 149.2, 145.0, 138.0, 134.9, 133.9, 130.1 (2C), 128.6 (2C), 128.5 (2C), 128.1 (2C), 127.6, 126.7, 123.7, 123.5, 119.4, 118.2, 115.8, 115.0, 83.9, 48.9, 41.7, 21.8 ppm.

HRMS (ESI$^+$): calculated for $C_{26}H_{26}BrSO_4N_2$ $[M+NH_4]^+$: 541.0791; found: 591.0780.

The enantiomeric excess was determined by SFC using a Chiralpak IA column [CO_2/MeOH from 95:5 to 60:40 in 8 min, flow rate 3.0 mL/min], τ_{minor} = 5.22 min, τ_{major} = 5.88 min (98:2 *er*).

(*2R,3R,4S*)-3-(2-Methylprop-1-en-1-yl)-4-phenyl-1-tosyl-1,2,3,4-tetrahydrobenzofuro[3,2-*b*] pyridin-2-ol (4n)

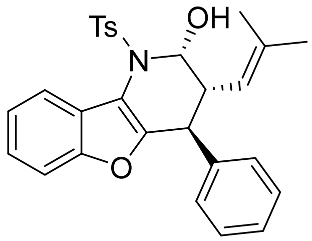

Following general procedure B, *N*-((*E*)-2-((*Z*)-benzylidene) benzofuran-3(*2H*)-ylidene)-4-methylbenzenesulfonamide **1a** (18.8 mg, 0.05 mmol) and silyl dienol ether **2c** (61 μL, 0.3 mmol) after 16h at room temperature, gave **4n** (71% yield, >20:1 *dr*) as a colorless oil. Eluent: cyclohexane: ethyl acetate from 97:3 to 83:17. **$[\alpha]^{25}_D$** = +162.3 (*c* 0.75, $CHCl_3$).

^{1}H NMR: δ 8.24 – 8.19 (m, 1H), 7.58 (d, *J* = 8.3 Hz, 2H), 7.37 – 7.24 (m, 5H), 7.17 – 7.05 (m, 3H), 6.57 – 6.51 (m, 2H), 5.63 (t, *J* = 2.6 Hz, 1H), 5.16 – 5.10 (m, 1H), 3.79 (d, *J* = 10.7 Hz, 1H), 2.68 (t, *J* = 2.6 Hz, 1H), 2.41 (s, 3H), 2.11 (t, *J* = 10.3 Hz, 1H), 1.58 (d, *J* = 1.4 Hz, 3H), 0.66 (d, *J* = 1.4 Hz, 3H) ppm.

^{13}C NMR: δ 154.5, 148.8, 144.6, 138.9, 136.8, 134.4, 130.1 (2C), 128.7 (2C), 128.2 (2C), 128.1 (2C), 127.1, 124.7, 124.7, 123.2, 122.4, 120.6, 116.1, 111.5, 83.6, 44.1, 42.6, 25.9, 21.7, 17.5 ppm.

HRMS (ESI$^+$): calculated for $C_{28}H_{31}SO_4N_2$ $[M+NH_4]^+$: 491.1999; found: 491.2013.

The enantiomeric excess was determined by SFC using a Chiralpak IA column [CO_2/MeOH from 95:5 to 60:40 in 8 min, flow rate 3.0 mL/min], τ_{minor} = 4.80 min, τ_{major} = 5.34 min (98:2 *er*).

(*2R,3S,4S*)-4-Phenyl-3-(prop-1-en-2-yl)-1-tosyl-1,2,3,4-tetrahydrobenzofuro[3,2-*b*]pyridin-2-ol (4o)

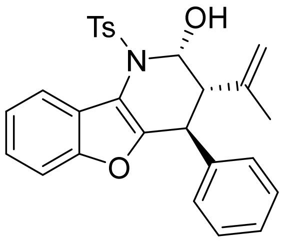

Following general procedure B, *N*-((*E*)-2-((*Z*)-benzylidene) benzofuran-3(*2H*)-ylidene)-4-methylbenzenesulfonamide **1a** (18.8 mg, 0.05 mmol) and silyl dienol ether **2d** (76 µL, 0.3 mmol) after 16h at room temperature, gave **4o** (63% yield, >20:1 *dr*) as a yellow oil. Eluent: cyclohexane: ethyl acetate from 97:3 to 83:17. **$[\alpha]^{25}_D$** = +149.2 (*c* 0.61, $CHCl_3$).

^{1}H NMR: δ 8.23 – 8.17 (m, 1H), 7.59 (d, *J* = 8.3 Hz, 2H), 7.35 – 7.27 (m, 5H), 7.20 – 7.09 (m, 3H), 6.59 – 6.54 (m, 2H), 5.77 (t, *J* = 2.5 Hz, 1H), 4.85 – 4.81 (m, 1H), 4.62 (s, 1H), 4.19 (d, *J* = 11.8 Hz, 1H), 2.68 (dd, *J* = 3.2, 1.7 Hz, 1H), 2.46 (s, 3H), 2.02 (d, *J* = 11.8 Hz, 1H), 1.64 (s, 3H) ppm.

^{13}C NMR: δ 154.6, 149.1, 144.8, 141.3, 138.5, 134.1, 130.1 (2C), 128.5 (2C), 128.4 (2C), 128.3 (2C), 127.4, 124.7, 124.6, 123.3, 122.5, 116.1, 115.4, 111.5, 83.8, 50.7, 40.1, 22.1, 21.8 ppm.

HRMS (ESI$^+$): calculated for $C_{27}H_{29}SO_4N_2$ $[M+NH_4]^+$: 477.1843; found: 477.1826.

The enantiomeric excess was determined by SFC using a Chiralpak IA column [CO_2/MeOH from 95:5 to 60:40 in 8 min, flow rate 3.0 mL/min], τ_{minor} = 4.75 min, τ_{major} = 5.24 min (98:2 *er*).

(*2R,3S,4S*)-3,4-Diphenyl-1-tosyl-1,2,3,4-tetrahydrobenzofuro[3,2-*b*]pyridin-2-ol (5a)

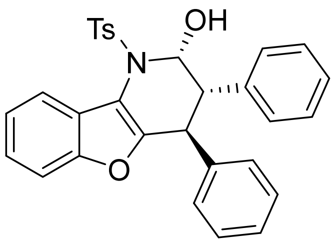

Following general procedure B, *N*-((*E*)-2-((*Z*)-benzylidene) benzofuran-3(*2H*)-ylidene)-4-methylbenzenesulfonamide **1a** (18.8 mg, 0.05 mmol) and silyl enol ether **2b** (62 µL, 0.3 mmol) after 16h at room temperature, gave **5a** (78% yield, >20:1 *dr*) as a colorless oil. Eluent: cyclohexane: ethyl acetate from 97:3 to 85:15. **$[\alpha]^{25}_D$** = +269.5 (*c* 1.32, $CHCl_3$).

^{1}H NMR: δ 8.28 – 8.21 (m, 1H), 7.66 (d, *J* = 8.3 Hz, 2H), 7.42 – 7.28 (m, 6H), 7.23 – 7.18 (m, 3H), 7.11 – 6.95 (m, 5H), 6.41 (d, *J* = 6.9 Hz, 1H), 5.82 (t, *J* = 2.6 Hz, 1H), 4.41 (d, *J* = 11.6 Hz, 1H), 2.82 (brs, 1H), 2.52 – 2.44 (m, 4H) ppm.

^{13}C NMR: δ 154.6, 149.1, 144.9, 138.4, 137.4, 134.2, 130.1 (2C), 129.4 (2C), 128.4 (2C), 128.3 (2C), 128.2 (2C), 128.1 (2C), 127.7, 127.3, 124.8, 124.7, 123.3, 122.5, 116.0, 111.6, 84.1, 50.9, 42.6, 21.8 ppm.

HRMS (ESI$^+$): calculated for $C_{30}H_{29}SO_4N_2$ $[M+NH_4]^+$: 513.1843; found: 513.1855.

The enantiomeric excess was determined by SFC using a Chiralpak IA column [CO_2/MeOH from 95:5 to 60:40 in 8 min, flow rate 3.0 mL/min], τ_{minor} = 5.72 min, τ_{major} = 6.13 min (94:6 *er*).

The reaction was also performed on a 1.0 mmol scale. In this case, and following general procedure B, catalyst **3e** (63 mg, 0.1 mmol), *N*-((*E*)-2-((*Z*)-benzylidene) benzofuran-3(*2H*)-ylidene)-4-methylbenzenesulfonamide **1a** (380 mg, 1.0 mmol) and silyl dienol ether **2d** (1.3 mL, 6.0 mmol) after 48h at room temperature, gave **5a** (326mg, 66% yield, >20:1 *dr*) as a yellowish solid. Eluent: cyclohexane: ethyl acetate from 97:3 to 85:15. The enantiomeric excess was determined by SFC using a Chiralpak IA column [CO_2/MeOH from 95:5 to 60:40 in 8 min, flow rate 3.0 mL/min], τ_{minor} = 5.72 min, τ_{major} = 6.28 min (93.5:6.5 *er*).

(*2R,3S,4S*)-4-(4-Bromophenyl)-3-phenyl-1-tosyl-1,2,3,4-tetrahydrobenzofuro[3,2-*b*]pyridin-2-ol (5b)

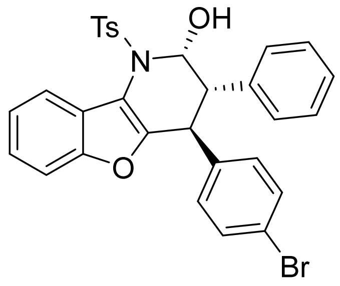

Following general procedure B, *N*-((*E*)-2-((*Z*)-4-bromobenzylidene) benzofuran-3(*2H*)-ylidene)-4-methylbenzenesulfonamide **1b** (22.7 mg, 0.05 mmol) and silyl enol ether **2b** (62 µL, 0.3 mmol) after 16h at room temperature, gave **5b** (77% yield, >20:1 *dr*) as a colorless oil. Eluent: cyclohexane: ethyl acetate from 98:2 to 85:15. **$[\alpha]^{25}_D$** = +53.6 (*c* 1.35, $CHCl_3$).

^{1}H NMR: δ 8.26 – 8.20 (m, 1H), 7.64 (d, *J* = 8.3 Hz, 2H), 7.38 (d, *J* = 8.1 Hz, 2H), 7.36 – 7.30 (m, 3H), 7.24 – 7.19 (m, 3H), 7.11 (d, *J* = 8.4 Hz, 2H), 7.01 (dd, *J* = 6.7, 2.9 Hz, 2H), 6.27 (d, *J* = 8.4 Hz, 2H), 5.80 (t, *J* = 2.6 Hz, 1H), 4.37 (d, *J* = 11.7 Hz, 1H), 2.80 (brs, 1H), 2.50 (s, 3H), 2.40 (d, *J* = 11.7 Hz, 1H) ppm.

^{13}C NMR: δ 154.6, 148.3, 145.0, 137.5, 137.0, 134.2, 131.4 (2C), 130.1 (2C), 130.0 (2C), 129.4 (2C), 128.6 (2C), 128.3 (2C), 127.9, 125.0, 124.6, 123.4, 122.6, 121.2, 116.3, 111.6, 84.0, 50.8, 42.1, 21.9 ppm.

HRMS (ESI$^+$): calculated for $C_{30}H_{28}SO_4N_2Br$ $[M+NH_4]^+$: 591.0948; found: 591.0921.

The enantiomeric excess was determined by SFC using a Chiralpak IC column [CO_2/MeOH from 95:5 to 60:40 in 8 min, flow rate 3.0 mL/min], τ_{major} = 4.82 min, τ_{minor} = 5.87 min (97:3 *er*).

(*2R,3S,4S*)-4-(4-Fluorophenyl)-3-phenyl-1-tosyl-1,2,3,4-tetrahydrobenzofuro[3,2-*b*]pyridin-2-ol (5c)

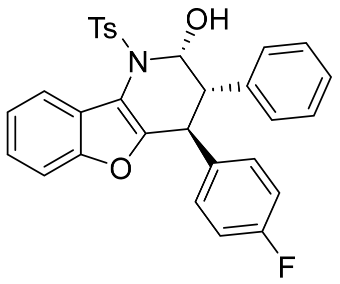

Following general procedure B, *N*-((*E*)-2-((*Z*)-4-fluorobenzylidene) benzofuran-3(*2H*)-ylidene)-4-methylbenzenesulfonamide **1c** (19.7 mg, 0.05 mmol) and silyl enol ether **2b** (62 µL, 0.3 mmol) after 16h at room temperature, gave **5d** (82% yield, >20:1 *dr*) as a colorless oil. Eluent: cyclohexane: ethyl acetate from 97:3 to 85:15. $[\alpha]^{25}_{D}$ = +190.5 (*c* 0.88, $CHCl_3$).

^{1}H NMR: δ 8.27 – 8.21 (m, 1H), 7.65 (d, *J* = 8.3 Hz, 2H), 7.38 (d, *J* = 8.5 Hz, 2H), 7.35 – 7.31 (m, 3H), 7.24 – 7.18 (m, 3H), 7.03 – 6.98 (m, 2H), 6.68 (t, *J* = 8.7 Hz, 2H), 6.39 – 6.31 (m, 2H), 5.81 (dd, *J* = 3.3, 2.1 Hz, 1H), 4.39 (d, *J* = 11.7 Hz, 1H), 2.79 (dd, *J* = 3.3, 1.8 Hz, 1H), 2.49 (s, 3H), 2.40 (d, *J* = 11.7 Hz, 1H) ppm.

^{13}C NMR: δ 161.8 (d, *J* = 245.7 Hz), 154.4, 148.5, 144.8, 137.0, 134.0, 133.9 (d, *J* = 3.2 Hz), 130.0 (2C), 129.6 (d, *J* = 8.0 Hz, 2C), 129.2 (2C), 128.4 (2C), 128.2 (2C), 127.6, 124.8, 124.5, 123.2, 122.4, 116.0, 115.0 (d, *J* = 21.4 Hz, 2C), 111.4, 83.8, 50.9, 41.8, 21.7 ppm.

^{19}F NMR: δ -115.3 ppm.

HRMS (ESI$^+$): calculated for $C_{30}H_{28}SFO_4N_2$ $[M+NH_4]^+$: 531.1748; found: 531.1743.

The enantiomeric excess was determined by SFC using a Chiralpak IC column [CO_2/MeOH from 95:5 to 60:40 in 8 min, flow rate 3.0 mL/min], τ_{major} = 4.08 min, τ_{minor} = 5.00 min (93:7 *er*).

(*2R,3S,4S*)-4-(4-Nitrophenyl)-3-phenyl-1-tosyl-1,2,3,4-tetrahydrobenzofuro[3,2-*b*]pyridin-2-ol (5d)

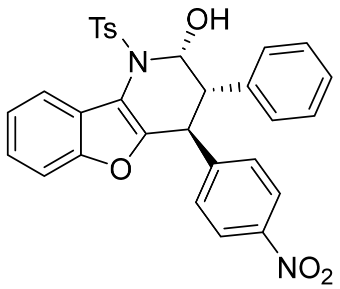

Following general procedure B, 4-methyl-*N*-((*E*)-2-((*Z*)-4-nitrobenzylidene) benzofuran-3(*2H*)-ylidene)benzenesulfonamide **1d** (21.0 mg, 0.05 mmol) and silyl enol ether **2b** (62 µL, 0.3 mmol) after 16h at room temperature, gave **5d** (87% yield, >20:1 *dr*) as a yellow oil. Eluent: cyclohexane: ethyl acetate from 97:3 to 80:20. $[\alpha]^{25}_{D}$ = +296.0 (*c* 1.11, $CHCl_3$).

^{1}H NMR: δ 8.30 – 8.20 (m, 1H), 7.84 (d, *J* = 8.6 Hz, 2H), 7.65 (d, *J* = 8.2 Hz, 2H), 7.40 (d, *J* = 8.2 Hz, 2H), 7.36 – 7.32 (m, 3H), 7.27 – 7.20 (m, 3H), 7.00 (dd, *J* = 6.5, 3.0 Hz, 2H), 6.55 (d, *J* = 9.0 Hz, 2H), 5.82 (s, 1H), 4.52 (d, *J* = 11.6 Hz, 1H), 3.01 (brs, 1H), 2.51 (s, 3H), 2.41 (d, *J* = 11.6 Hz, 1H) ppm.

^{13}C NMR: δ 154.7, 147.3, 147.2, 146.4, 145.2, 136.5, 134.2, 130.2 (2C), 129.3 (2C), 129.2 (2C), 128.7 (2C), 128.3 (2C), 128.2, 125.3, 124.4, 123.6, 123.5 (2C), 122.7, 116.8, 111.6, 83.8, 50.9, 42.7, 21.9 ppm.

HRMS (ESI$^+$): calculated for $C_{30}H_{28}SO_6N_3$ $[M+NH_4]^+$: 558.1693; found: 558.1667.

The enantiomeric excess was determined by SFC using a Chiralpak IC column [CO_2/MeOH from 95:5 to 60:40 in 8 min, flow rate 3.0 mL/min], τ_{major} = 5.89 min, τ_{minor} = 7.24 min (96:4 *er*).

(*2R,3S,4S*)-3-Phenyl-1-tosyl-4-(4-(trifluoromethyl)phenyl)-1,2,3,4-tetrahydrobenzofuro[3,2-*b*]pyridin-2-ol (5e)

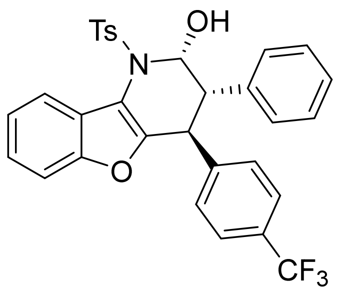

Following general procedure B, 4-methyl-*N*-((*E*)-2-((*Z*)-4-(trifluoromethyl)benzylidene) benzofuran-3(*2H*)-ylidene)benzenesulfonamide **1e** (22.2 mg, 0.05 mmol) and silyl enol ether **2b** (62 µL, 0.3 mmol) after 16h at room temperature, gave **5e** (76% yield, >20:1 *dr*) as a yellow oil. Eluent: cyclohexane: ethyl acetate from 98:2 to 85:15. **$[\alpha]^{25}_D$** = +216.9 (*c* 0.84, $CHCl_3$).

^{1}H NMR: δ 8.27 – 8.23 (m, 1H), 7.66 (d, *J* = 8.3 Hz, 2H), 7.39 (d, *J* = 7.9 Hz, 2H), 7.36 – 7.33 (m, 3H), 7.25 – 7.21 (m, 5H), 7.05 – 6.98 (m, 2H), 6.51 (d, *J* = 8.0 Hz, 2H), 5.82 (t, *J* = 2.6 Hz, 1H), 4.48 (d, *J* = 11.7 Hz, 1H), 2.79 (brs, 1H), 2.49 (s, 3H), 2.48 – 2.42 (m, 1H) ppm.

^{13}C NMR: δ 154.6, 148.0, 145.1, 142.8, 136.8, 134.2, 130.2 (2C), 129.8 (q, *J* = 32.3 Hz), 129.4 (2C), 128.7 (2C), 128.6 (2C), 128.3 (2C), 128.0, 125.2 (q, *J* = 4.0 Hz, 2C), 125.1, 124.5, 124.1 (q, *J* = 273.0 Hz), 123.5, 122.6, 116.5, 83.9, 50.8, 42.5, 21.8 ppm.

^{19}F NMR: δ -62.6 ppm.

HRMS (ESI$^+$): calculated for $C_{31}H_{28}SF_3O_4N_2$ $[M+NH_4]^+$: 581.1716; found: 581.1722.

The enantiomeric excess was determined by SFC using a Chiralpak IC column [CO_2/MeOH from 95:5 to 60:40 in 8 min, flow rate 3.0 mL/min], τ_{major} = 3.24 min, τ_{minor} = 4.06 min (90:10 *er*).

4-((*2R,3S,4S*)-2-Hydroxy-3-phenyl-1-tosyl-1,2,3,4-tetrahydrobenzofuro[3,2-*b*]pyridin-4-yl)benzonitrile (5f)

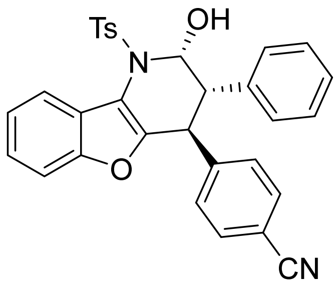

Following general procedure B, *N*-((*E*)-2-((*Z*)-4-cyanobenzylidene) benzofuran-3(*2H*)-ylidene)-4-methylbenzenesulfonamide **1f** (20.0 mg, 0.05 mmol) and silyl enol ether **2b** (62 µL, 0.3 mmol) after 16h at room temperature, gave **5f** (81% yield, >20:1 *dr*) as a yellow oil. Eluent: cyclohexane: ethyl acetate from 97:3 to 85:15. **[α]$^{25}_{D}$** = +183.4 (*c* 1.82, $CHCl_3$).

^{1}H NMR: δ 8.30 – 8.20 (m, 1H), 7.65 (d, *J* = 8.3 Hz, 2H), 7.41 – 7.33 (m, 5H), 7.25 – 7.19 (m, 5H), 7.01 – 6.96 (m, 2H), 6.50 (d, *J* = 8.3 Hz, 2H), 5.82 (t, *J* = 2.4 Hz, 1H), 4.46 (d, *J* = 11.6 Hz, 1H), 2.89 (brs, 1H), 2.50 (s, 3H), 2.39 (d, *J* = 12.1 Hz, 1H) ppm.

^{13}C NMR: δ 154.7, 147.4, 145.1, 144.4, 136.6, 134.2, 132.1 (2C), 130.2 (2C), 129.3 (2C), 129.2, 129.1 (2C), 128.7 (2C), 128.3 (2C), 128.1, 125.3, 124.4, 123.6, 122.7, 118.7, 116.8, 111.6, 83.8, 50.8, 42.9, 21.9 ppm.

HRMS (ESI$^+$): calculated for $C_{31}H_{28}SO_4N_3$ $[M+NH_4]^+$: 538.1795; found: 538.1797.

The enantiomeric excess was determined by SFC using a Chiralpak IC column [CO_2/MeOH from 95:5 to 60:40 in 8 min, flow rate 3.0 mL/min], τ_{major} = 5.62 min, τ_{minor} = 6.91 min (90:10 *er*).

(*2R,3S,4S*)-4-(3-Chlorophenyl)-3-phenyl-1-tosyl-1,2,3,4-tetrahydrobenzofuro[3,2-*b*]pyridin-2-ol (5g)

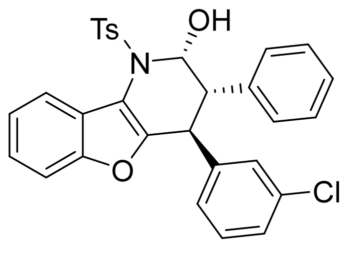

Following general procedure B, *N*-((*E*)-2-((*Z*)-3-chlorobenzylidene) benzofuran-3(*2H*)-ylidene)-4-methylbenzenesulfonamide **1g** (20.3 mg, 0.05 mmol) and silyl enol ether **2b** (62 µL, 0.3 mmol) after 16h at room temperature, gave **5g** (75% yield, >20:1 *dr*) as a colorless oil. Eluent: cyclohexane: ethyl acetate from 98:2 to 85:15. **[α]$^{25}_{D}$** = +191.4 (*c* 0.94, $CHCl_3$).

^{1}H NMR: δ 8.30 – 8.22 (m, 1H), 7.66 (d, *J* = 8.3 Hz, 2H), 7.43 (d, *J* = 8.1 Hz, 2H), 7.38 – 7.32 (m, 3H), 7.24 – 7.19 (m, 3H), 7.07 – 6.98 (m, 3H), 6.92 (t, *J* = 7.8 Hz, 1H), 6.44 (dt, *J* = 7.7, 1.4 Hz, 1H), 6.34 (t, *J* = 1.9 Hz, 1H), 5.81 – 5.79 (m, 1H), 4.39 (d, *J* = 11.7 Hz, 1H), 2.77 (dd, *J* = 3.2, 1.9 Hz, 1H), 2.50 (s, 3H), 2.33 (dt, *J* = 11.7, 1.9 Hz, 1H) ppm.

^{13}C NMR: δ 154.7, 148.2, 145.4, 140.5, 136.9, 134.3, 134.1, 130.4 (2C), 129.5, 129.4 (2C), 128.5 (2C), 128.2 (2C), 127.9, 127.6, 127.6, 127.4, 125.1, 124.6, 123.4, 122.7, 116.4, 111.6, 83.9, 50.7, 42.3, 22.0 ppm.

HRMS (ESI$^+$): calculated for $C_{30}H_{28}SO_4N_2Cl$ $[M+NH_4]^+$: 547.1453; found: 547.1479.

The enantiomeric excess was determined by SFC using a Chiralpak IB-3 column [CO_2/MeOH from 95:5 to 70:30 in 8 min, flow rate 2.0 mL/min], τ_{major} = 3.63 min, τ_{minor} = 5.61 min (89:11 *er*).

(*2R,3S,4S*)-4-(2-Fluorophenyl)-3-phenyl-1-tosyl-1,2,3,4-tetrahydrobenzofuro[3,2-*b*]pyridin-2-ol (5h)

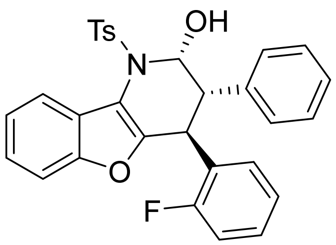

Following general procedure B, *N*-((*E*)-2-((*Z*)-2-fluorobenzylidene) benzofuran-3(*2H*)-ylidene)-4-methylbenzenesulfonamide **1h** (19.7 mg, 0.05 mmol) and silyl enol ether **2b** (62 µL, 0.3 mmol) after 16h at room temperature, gave **5h** (64% yield, >20:1 *dr*) as a colorless oil. Eluent: cyclohexane: ethyl acetate from 97:3 to 85:15. **$[\alpha]^{25}_D$** = +105.2 (*c* 1.42, $CHCl_3$).

^{1}H NMR: δ 8.27 – 8.21 (m, 1H), 7.67 (d, *J* = 8.3 Hz, 2H), 7.39 (d, *J* = 8.6 Hz, 2H), 7.35 – 7.30 (m, 3H), 7.23 – 7.17 (m, 3H), 7.10 – 7.04 (m, 3H), 6.83 – 6.73 (m, 2H), 6.19 (t, *J* = 7.8 Hz, 1H), 5.83 (brs, 1H), 4.86 (d, *J* = 11.9 Hz, 1H), 2.69 (brs, 1H), 2.59 (d, *J* = 11.9 Hz, 1H), 2.50 (s, 3H) ppm.

^{13}C NMR: δ 161.3 (d, *J* = 247.9 Hz), 154.5, 148.2, 144.9, 137.0, 134.4, 130.2 (2C), 129.7, 129.3 (2C), 129.0 (d, *J* = 8.4 Hz), 128.4 (2C), 128.3 (2C), 127.8, 125.1 (d, *J* = 14.0 Hz), 124.8, 124.6, 124.0 (d, *J* = 3.6 Hz), 123.4, 122.6, 115.9, 115.7 (d, *J* = 22.6 Hz), 111.6, 84.1, 49.4, 35.1, 21.8 ppm.

^{19}F NMR: δ -117.4 ppm.

HRMS (ESI$^+$): calculated for $C_{30}H_{28}SFO_4N_2$ $[M+NH_4]^+$: 531.1748; found: 531.1743.

The enantiomeric excess was determined by SFC using a Chiralpak IC column [CO_2/MeOH from 95:5 to 60:40 in 8 min, flow rate 3.0 mL/min], τ_{major} = 4.22 min, τ_{minor} = 4.92 min (92:8 *er*).

(*2R,3S,4S*)-4-(2-Bromophenyl)-3-phenyl-1-tosyl-1,2,3,4-tetrahydrobenzofuro[3,2-*b*]pyridin-2-ol (5i)

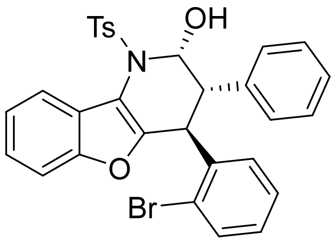

Following general procedure B, *N*-((*E*)-2-((*Z*)-2-bromobenzylidene)benzofuran-3(*2H*)-ylidene)-4-methylbenzene sulfonamide **1i** (22.7 mg, 0.05 mmol) and silyl dienol ether **2b** (62 µL, 0.3 mmol) after 16h at room temperature, gave **5i** (52% yield, >20:1 *dr*) as a yellow oil. Eluent: cyclohexane: ethyl acetate from 97:3 to 83:17. **[α]$^{25}_{D}$** = +98.3 (*c* 0.65, $CHCl_3$).

^{1}H NMR: δ 8.23 (d, *J* = 7.3 Hz, 1H), 7.67 (d, *J* = 8.3 Hz, 2H), 7.45 – 7.28 (m, 5H), 7.23 – 7.16 (m, 4H), 7.13 – 7.07 (m, 2H), 6.98 – 6.88 (m, 2H), 5.93 (d, *J* = 7.1 Hz, 1H), 5.87 (brs, 1H), 5.17 (d, *J* = 11.8 Hz, 1H), 2.86 (brs, 1H), 2.59 (d, *J* = 11.8 Hz, 1H), 2.53 (s, 3H) ppm.

^{13}C NMR: δ 154.6, 148.8, 144.9, 137.9, 134.3, 133.2, 130.2 (2C), 129.9, 129.7 (2C), 128.8, 128.5 (2C), 128.3 (2C), 127.8, 127.4, 127.2, 126.3, 124.9, 124.5, 123.3, 122.5, 115.6, 111.6, 84.3, 50.5, 40.6, 21.9 ppm.

HRMS (ESI$^+$): calculated for $C_{30}H_{28}BrSO_4N_2$ $[M+NH_4]^+$: 591.0948; found: 591.0958.

The enantiomeric excess was determined by SFC using a Chiralpak IC column [CO_2/MeOH from 95:5 to 60:40 in 8 min, flow rate 3.0 mL/min], τ_{major} = 4.74 min, τ_{minor} = 5.38 min (90:10 *er*).

(*2R,3S,4S*)-4-(3-Methoxyphenyl)-3-phenyl-1-tosyl-1,2,3,4-tetrahydrobenzofuro[3,2-*b*]pyridin-2-ol (5j)

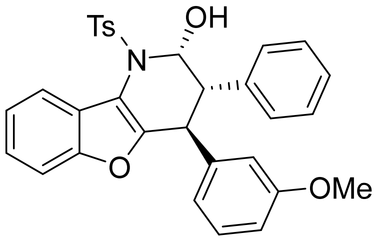

Following general procedure B, *N*-((*E*)-2-((*Z*)-3-methoxybenzylidene) benzofuran-3(*2H*)-ylidene)-4-methylbenzenesulfonamide **1j** (20.3 mg, 0.05 mmol) and silyl enol ether **2b** (62 µL, 0.3 mmol) after 16h at room temperature, gave **5j** (98% yield, >20:1 *dr*) as a yellow oil. Eluent: cyclohexane: ethyl acetate from 97:3 to 80:20. **[α]$^{25}_{D}$** = +234.0 (*c* 2.12, $CHCl_3$).

^{1}H NMR: δ 8.27 – 8.21 (m, 1H), 7.65 (d, *J* = 8.3 Hz, 2H), 7.42 – 7.29 (m, 5H), 7.21 (dd, *J* = 5.0, 1.9 Hz, 3H), 7.07 – 7.01 (m, 2H), 6.92 (t, *J* = 7.9 Hz, 1H), 6.61 (dd, *J* = 8.3, 2.6 Hz, 1H), 6.04 (d, *J* = 7.6 Hz, 1H), 5.99 – 5.96 (m, 1H), 5.81 (s, 1H), 4.39 (d, *J* = 11.7 Hz, 1H), 3.59 (s, 3H), 2.91 (brs, 1H), 2.50 – 2.40 (m, 4H) ppm.

^{13}C NMR: δ 159.5, 154.6, 148.9, 145.1, 139.8, 137.4, 134.1, 130.2 (2C), 129.5 (2C), 129.1, 128.4 (2C), 128.2 (2C), 127.6, 124.8, 124.7, 123.3, 122.5, 120.9, 116.0, 114.8, 112.0, 111.6, 84.1, 55.1, 50.7, 42.5, 21.8 ppm.

HRMS (ESI$^+$): calculated for $C_{31}H_{31}SO_5N_2$ $[M+NH_4]^+$: 543.1948; found: 543.1950.

The enantiomeric excess was determined by SFC using a Chiralpak IB-3 column [CO_2/MeOH from 95:5 to 60:40 in 8 min, flow rate 2.0 mL/min], τ_{major} = 3.72 min, τ_{minor} = 5.17 min (95:5 *er*).

(*2R,3S,4S*)-4-(Naphthalen-2-yl)-3-phenyl-1-tosyl-1,2,3,4-tetrahydrobenzofuro[3,2-*b*]pyridin-2-ol (5k)

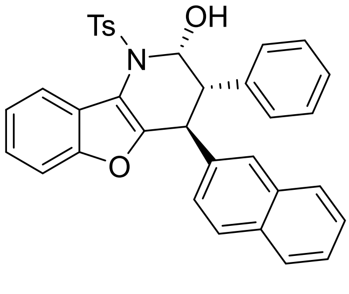

Following general procedure B, 4-methyl-*N*-((*2Z,3E*)-2-(naphthalen-2-ylmethylene) benzofuran-3(*2H*)-ylidene)benzenesulfonamide **1k** (21.3 mg, 0.05 mmol) and silyl enol ether **2b** (62 µL, 0.3 mmol) after 16h at room temperature, gave **5j** (89% yield, >20:1 *dr*) as a yellow oil. Eluent: cyclohexane: ethyl acetate from 98:2 to 85:15. **[α]^{25}$_{D}$** = +410.1 (*c* 0.98, $CHCl_3$).

^{1}H NMR: δ 8.27 (d, *J* = 7.1 Hz, 1H), 7.71 (d, *J* = 8.3 Hz, 2H), 7.69 – 7.65 (m, 1H), 7.55 – 7.50 (m, 1H), 7.48 – 7.30 (m, 8H), 7.21 – 7.15 (m, 3H), 7.09 – 7.01 (m, 3H), 6.42 (dd, *J* = 8.5, 1.8 Hz, 1H), 5.88 – 5.83 (m, 1H), 4.59 (d, *J* = 11.7 Hz, 1H), 2.71 (brs, 1H), 2.60 (d, *J* = 11.7 Hz, 1H), 2.50 (s, 3H) ppm.

^{13}C NMR: δ 154.6, 149.1, 145.0, 137.3, 135.8, 134.3, 133.3, 132.7, 130.2 (2C), 129.4 (2C), 128.5 (2C), 128.4 (2C), 127.9, 127.7 (3C), 127.6, 126.1, 126.0, 125.9, 124.9, 124.7, 123.3, 122.6, 116.1, 111.6, 84.1, 50.8, 42.6, 21.9 ppm.

HRMS (ESI$^+$): calculated for $C_{34}H_{31}SO_4N_2$ $[M+NH_4]^+$: 563.1999; found: 563.1978.

The enantiomeric excess was determined by SFC using a Chiralpak IC column [CO_2/MeOH from 95:5 to 60:40 in 8 min, flow rate 3.0 mL/min], τ_{major} = 5.45 min, τ_{minor} = 6.40 min (93.5:6.5 *er*).

(*2R,3S,4S*)-8-Bromo-3,4-diphenyl-1-tosyl-1,2,3,4-tetrahydrobenzofuro[3,2-*b*]pyridin-2-ol (5l)

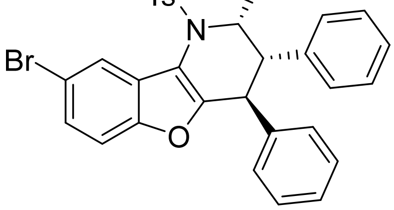

Following general procedure B, *N*-((*E*)-2-((*Z*)-benzylidene)-5-bromobenzofuran-3(*2H*)-ylidene)-4-methylbenzenesulfonamide **1l** (22.7 mg, 0.05 mmol) and silyl enol ether **2b** (62 µL, 0.3 mmol) after 16h at room temperature, gave **5l** (88% yield, >20:1 *dr*) as a yellow oil. Eluent: cyclohexane: ethyl acetate from 97:3 to 85:15. **[α]^{25}$_{D}$** = +299.2 (*c* 1.07, $CHCl_3$).

^{1}H NMR: δ 8.36 (t, *J* = 2.1 Hz, 1H), 7.66 (d, *J* = 8.3 Hz, 2H), 7.44 – 7.35 (m, 3H), 7.20 (dd, *J* = 5.6, 3.1 Hz, 4H), 7.07 – 6.96 (m, 6H), 6.43 – 6.35 (m, 2H), 5.80 (d, *J* = 2.1 Hz, 1H), 4.39 (d, *J* = 11.7 Hz, 1H), 2.51 – 2.43 (m, 4H) ppm.

^{1}H NMR: δ 153.3, 150.5, 145.1, 137.9, 137.1, 133.9, 130.2 (2C), 129.4 (2C), 128.5 (2C), 128.4 (4C), 128.3 (2C), 127.8, 127.7, 127.4, 126.6, 125.1, 116.6, 115.5, 113.0, 84.0, 50.8, 42.5, 21.9 ppm.

HRMS (ESI$^+$): calculated for $C_{30}H_{28}SO_4N_2Br$ [M+NH$_4$]$^+$: 591.0948; found: 591.1001.

The enantiomeric excess was determined by SFC using a Chiralpak IC column [CO_2/MeOH from 95:5 to 60:40 in 8 min, flow rate 3.0 mL/min], τ_{major} = 4.86 min, τ_{minor} = 5.54 min (88:12 *er*).

(*2R,3S,4S*)-7-Bromo-3,4-diphenyl-1-tosyl-1,2,3,4-tetrahydrobenzofuro[3,2-*b*]pyridin-2-ol (5m)

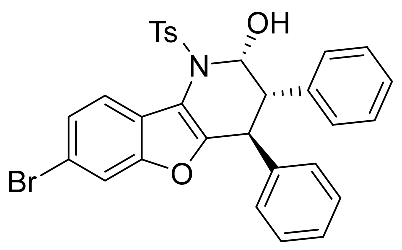

Following general procedure B, *N*-((*E*)-2-((*Z*)-benzylidene)-6-bromobenzofuran-3(*2H*)-ylidene)-4-methylbenzene sulfonamide **1m** (22.7 mg, 0.05 mmol) and silyl dienol ether **2b** (62 µL, 0.3 mmol) after 16h at room temperature, gave **5m** (66% yield, >20:1 *dr*) as a colorless oil. Eluent: cyclohexane: ethyl acetate from 97:3 to 83:17. **[α]^{25}$_D$** = +156.1 (*c* 0.41, $CHCl_3$).

^{1}H NMR: δ 8.10 (d, *J* = 8.5 Hz, 1H), 7.63 (d, *J* = 8.2 Hz, 2H), 7.51 (d, *J* = 1.6 Hz, 1H), 7.44 (dd, *J* = 8.5, 1.6 Hz, 1H), 7.39 (d, *J* = 8.2 Hz, 2H), 7.23 – 7.18 (m, 3H), 7.12 – 6.95 (m, 5H), 6.37 (d, *J* = 7.4 Hz, 2H), 5.80 (brs, 1H), 4.37 (d, *J* = 11.7 Hz, 1H), 2.81 (brs, 1H), 2.49 (s, 3H), 2.45 (d, *J* = 11.7 Hz, 1H) ppm.

^{13}C NMR: δ 154.8, 149.7, 145.1, 138.0, 137.1, 134.0, 130.2 (2C), 129.4 (2C), 128.5 (2C), 128.4 (2C), 128.3 (4C), 127.8, 127.4, 126.7, 123.8, 123.6, 118.2, 116.0, 115.0, 84.0, 50.8, 42.5, 21.8 ppm.

HRMS (ESI$^+$): calculated for $C_{30}H_{28}BrSO_4N_2$ [M+NH$_4$]$^+$: 591.0948; found: 591.0966.

The enantiomeric excess was determined by SFC using a Chiralpak IB-3 column [CO_2/MeOH from 95:5 to 70:30 in 8 min, flow rate 3.0 mL/min], τ_{major} = 3.98 min, τ_{minor} = 5.75 min (97:3 *er*).

6. General procedure C: Synthesis of 6a and 7a

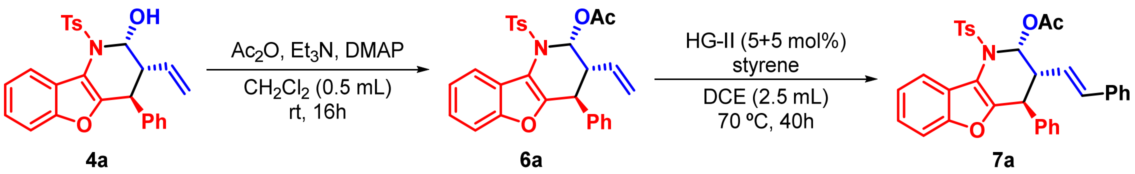

Hemiaminal **4a** (44.6 mg, 0.1 mmol, 1.0 equiv.) and 4-(dimethylamino)pyridine (1.2 mg, 0.01 mmol, 0.1 equiv.) were dissolved in DCM (0.5 mL). Then, acetic anhydride (19 µL, 0.2 mmol, 2.0 equiv.) and triethylamine (28 µL, 0.2 mmol, 2.0 equiv.) were added and the reaction was stirred overnight at room temperature. After that, the mixture was concentrated *in vacuo.* Finally, the crude mixture was purified by flash column chromatography using silica gel to afford product **6a**.

A previously oven-dried vial was charged with a magnetic stirrer, Hoveyda-Grubbs 2nd Generation Catalyst™ (1.6 mg, 0.0025 mmol, 0.05 equiv.) and **6** (24.4 mg, 0.05 mmol, 1.0 equiv.). Then, DCE (2.5 mL) was added and the reaction was purged with nitrogen for 10 minutes. Styrene (12 µL, 0.1 mmol, 2 equiv.) was added and the reaction mixture was stirred for 16 hours at 70 °C. The reaction was cooled to room temperature and Hoveyda-Grubbs 2nd Generation Catalyst™ (1.6 mg, 0.0025 mmol, 0.05 equiv.) was added. The reaction was purged again with nitrogen for 10 minutes and styrene (12 µL, 0.1 mmol, 2 equiv.) was added. The reaction mixture was stirred for 24 hours at 70 °C. Finally, the solvent was evaporated under reduced pressure and the crude mixture was purified by column chromatography, affording product **7a**.

(2R,3R,4S)-4-Phenyl-1-tosyl-3-vinyl-1,2,3,4-tetrahydrobenzofuro[3,2-*b*]pyridin-2-yl acetate (6a)

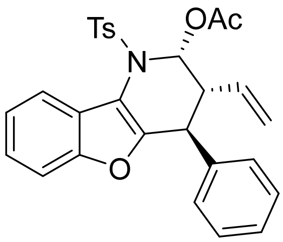

Following general procedure C, (*2R*,*3R*,*4S*)-4-phenyl-1-tosyl-3-vinyl-1,2,3,4-tetrahydrobenzofuro[3,2-*b*]pyridin-2-ol **4a** after 16h at room temperature, gave **6a** (76% yield, >20:1 *dr*) as a white solid. Eluent: cyclohexane: ethyl acetate from 97:3 to 85:15. $[\alpha]^{25}_{D}$ = +191.1 (*c* 0.95, $CHCl_3$).

^{1}H NMR: δ 8.19 – 8.13 (m, 1H), 7.64 (d, *J* = 8.3 Hz, 2H), 7.37 – 7.29 (m, 5H), 7.23 – 7.12 (m, 3H), 6.81 (d, *J* = 2.5 Hz, 1H), 6.60 – 6.54 (m, 2H), 5.47 (ddd, *J* = 17.2, 10.4, 8.2 Hz, 1H), 5.03 (d, *J* = 10.4 Hz, 1H), 4.70 (d, *J* = 17.2 Hz, 1H), 3.92 (d, *J* = 11.0 Hz, 1H), 2.46 (s, 3H), 2.23 – 2.13 (m, 1H), 2.03 (s, 3H) ppm.

^{13}**C NMR:** δ 168.8, 154.4, 148.1, 144.9, 138.1, 134.1, 133.4, 130.1 (2C), 128.6 (2C), 128.5 (2C), 128.3 (2C), 127.7, 124.9, 124.2, 123.3, 122.6, 120.3, 116.7, 111.5, 81.0, 47.8, 42.2, 21.8, 20.9 ppm.

HRMS (ESI$^+$): calculated for $C_{28}H_{29}SO_5N_2$ $[M+NH_4]^+$: 505.1792; found: 505.1812.

The enantiomeric excess was determined by SFC using a Chiralpak IA column [CO_2/MeOH from 95:5 to 60:40 in 8 min, flow rate 3.0 mL/min], τ_{minor} = 3.58 min, τ_{major} = 3.81 min (97:3 *er*).

(*2R*,*3R*,*4S*)-4-Phenyl-3-((*E*)-styryl)-1-tosyl-1,2,3,4-tetrahydrobenzofuro[3,2-*b*]pyridin-2-yl acetate (7a)

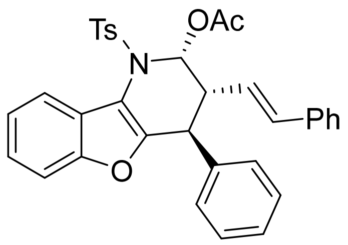

Following general procedure C, (*2R*,*3R*,*4S*)-4-phenyl-1-tosyl-3-vinyl-1,2,3,4-tetrahydrobenzofuro[3,2-*b*]pyridin-2-yl acetate **6a** after 40h at 70 °C, gave **7a** (72% yield, >20:1 *dr*) as a yellowish solid. Eluent: cyclohexane: ethyl acetate from 98:2 to 85:15. $[\alpha]^{25}_D$ = +215.0 (*c* 0.25, $CHCl_3$).

^{1}**H NMR:** δ 8.18 – 8.13 (m, 1H), 7.68 (d, *J* = 8.3 Hz, 2H), 7.38 – 7.30 (m, 5H), 7.26 (m, 3H), 7.21 – 7.11 (m, 5H), 6.86 (d, *J* = 2.5 Hz, 1H), 6.61 – 6.56 (m, 2H), 5.95 (d, *J* = 16.0 Hz, 1H), 5.78 (dd, *J* = 16.0, 8.1 Hz, 1H), 4.00 (d, *J* = 11.0 Hz, 1H), 2.48 (s, 3H), 2.36 (ddd, *J* = 10.8, 8.1, 2.5 Hz, 1H), 2.05 (s, 3H) ppm.

^{13}**C NMR:** δ 168.8, 154.4, 148.1, 145.0, 138.1, 136.4, 134.9, 134.3, 130.1 (2C), 128.7 (2C), 128.6 (2C), 128.5 (2C), 128.4 (2C), 128.1, 127.7, 126.5 (2C), 125.0, 124.6, 124.3, 123.4, 122.6, 116.8, 111.5, 81.2, 47.3, 42.7, 21.8, 21.0 ppm.

HRMS (ESI$^+$): calculated for $C_{34}H_{33}SO_5N_2$ $[M+NH_4]^+$: 581.2105; found: 581.2123.

The enantiomeric excess was determined by SFC using a Chiralpak IC column [CO_2/MeOH from 95:5 to 60:40 in 8 min, flow rate 3.0 mL/min], τ_{major} = 5.52 min, τ_{minor} = 5.80 min (97:3 *er*).

7. General procedure D: Synthesis of 8a

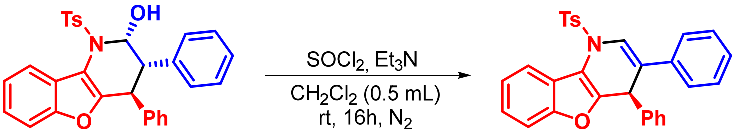

5a **8a**

Hemiaminal **5a** (24.8 mg, 0.05 mmol, 1.0 equiv.) was dissolved in DCM (0.5 mL) under nitrogen atmosphere in a vial. Then, triethylamine (21 µL, 0.15 mmol, 3.0 equiv.) and thionyl chloride (11 µL, 0.15 mmol, 3.0 equiv.) were added and the reaction was stirred overnight at room temperature. After that, the mixture was concentrated *in vacuo.* Finally, the crude mixture was purified by flash column chromatography using silica gel to afford product **8a**.

(*R*)-3,4-Diphenyl-1-tosyl-1,4-dihydrobenzofuro[3,2-*b*]pyridine (8a)

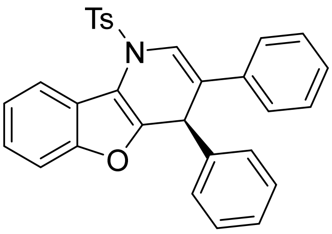

Following general procedure D, (*2R,3S,4S*)-3,4-diphenyl-1-tosyl-1,2,3,4-tetrahydrobenzofuro[3,2-*b*]pyridin-2-ol **5a** after 16h, gave **8a** (54% yield) as a yellow oil. Eluent: cyclohexane: ethyl acetate from 97:3 to 81:19. **$[\alpha]^{25}_D$** = +13.4 (*c* 0.52, $CHCl_3$).

^{1}H NMR: δ 8.43 – 8.35 (m, 1H), 7.58 (d, *J* = 8.1 Hz, 2H), 7.39 – 7.29 (m, 4H), 7.25 – 7.21 (m, 7H), 7.08 – 7.01 (m, 1H), 6.93 (t, *J* = 7.5 Hz, 2H), 6.48 (d, *J* = 7.5 Hz, 2H), 5.21 (s, 1H), 2.44 (s, 3H) ppm.

^{13}C NMR (CD_2Cl_2): δ 154.4, 147.0, 145.5, 132.5, 130.4 (2C), 129.2, 128.9 (2C), 128.6 (2C), 128.4 (2C), 128.2 (2C), 127.7, 127.4, 127.2, 127.0, 126.7 (2C), 126.0, 125.1, 124.5, 123.7, 122.9, 114.8, 111.6, 43.6, 21.8 ppm.

HRMS (ESI$^+$): calculated for $C_{30}H_{27}SO_3N_2$ $[M+NH_4]^+$: 495.1737; found: 495.1721.

The enantiomeric excess was determined by SFC using a Chiralpak IC column [CO_2/MeOH from 95:5 to 60:40 in 8 min, flow rate 3.0 mL/min], τ_{major} = 5.34 min, τ_{minor} = 5.79 min (97:3 *er*).

8. References

1. (a) Cassani, C.; Martín-Rapún, R.; Arceo, E.; Bravo F.; Melchiorre, P. Synthesis of 9-amino(9-deoxy)epi cinchona alkaloids, general chiral organocatalysts for the stereoselective functionalization of carbonyl compounds. *Nat. Protoc.* **2013**, *8*, 325-344. (b) Sladojevich, F.; Fuentes de Arriba, Á. L.; Ortín, I.; Yang, T.; Ferrali, A.; Paton R. S.; Dixon. D. J. Mechanistic Investigations into the Enantioselective Conia-Ene Reaction Catalyzed by Cinchona-Derived Amino Urea Pre-Catalysts and Cu^{I}. *Chem. Eur. J.* **2013**, *19*, 14286-14295. (c) Frias, M.; Mas-Balleste ́, R.; Arias, S.; Alvarado, C.; Alemán, J. Asymmetric Synthesis of Rauhut−Currier type Products by a Regioselective Mukaiyama Reaction under Bifunctional Catalysis. *J. Am. Chem. Soc.*, **2017**, *139*, 672−679.

2. (a) Wang Y.; Gu, M. The Concept of Spectral Accuracy for MS. *Anal. Chem.* **2010**, *82*, 7055-7062. (b) Wang, Y. Methods for Operating MS Instrument Systems, United States Patent No. 6,983,213, 2006. (c) Ochiaia, N.; Sasamoto, K.; MacNamara, K. Characterization of sulfur compounds in whisky by full evaporation dynamic head space and selectable one-dimensional/two-dimensional retention timelocked gas chromatography–mass spectrometry with simultaneous element-specific detection. *J. Chromatogr. A* **2012**, *1270*, 296-304. (d) Ho, H.; Lee, R.; Chen, C.; Wang, S.; Li, Z.; Lee, M. dentification of new minor metabolites of penicillin G in human serum by multiple-stage tandem mass spectrometry. *Rapid Commun. Mass Spectrom*. **2011**, *25*, 25-32.

3. Gu, Z.; Xie, J.; Jiang, G.-F.; Zhou, Y. Catalytic Asymmetric Conjugate Addition of Tritylthiol to Azadienes with a Bifunctional Organocatalyst. *Asian J. Org. Chem.* **2018**, *7*, 1561-1564.

4. Rong, Z.; Wang, M.; Chow, C. H. E.; Zhao, Y. A Catalyst-Enabled Diastereodivergent Aza-Diels–Alder Reaction: Complementarity of N-Heterocyclic Carbenes and Chiral Amine. *Chem. Eur. J.* **2016**, *22*, 9483-9487.

5. Xie, H.; Sun, L.; Wu, B.; Zhou, Y. Copper-Catalyzed Alkynylation/Cyclization/Isomerization Cascade for Synthesis of 1,2-Dihydrobenzofuro[3,2-b]pyridines and Benzofuro[3,2-b]pyridines. *J. Org. Chem.* **2019**, *84*, 15498-15507.

6. Zeng, R.; Shan, C.; Liu, M.; Jiang, K.; Ye, Y.; Liu, T.; Chen, Y. [4 + 1 + 1] Annulations of α-Bromo Carbonyls and 1-Azadienes toward Fused Benzoazaheterocycles. *Org. Lett.* **2019**, *21*, 2312-2316.

7. Tobisu, M.; Takahira, T.; Morioka, T.; Chatani, N. Nickel-Catalyzed Alkylative Cross-Coupling of Anisoles with Grignard Reagents via C−O Bond Activation. *J. Am. Chem. Soc.* **2016**, *138*, 6711-6714.

8. (a) Aggarwal, V. K.; Sheldon, C. G.; Macdonald, G. J.; Martin, W. P. A New Method for the Preparation of Silyl Enol Ethers from Carbonyl Compounds and (Trimethylsilyl)diazomethane in a Regiospecific and Highly Stereoselective Manner. *J. Am. Chem. Soc.* **2002**, *124*, 10300-10301. (b) Dias, E. L.; Brookhart, M.; White, P. S. Rhodium(I)-Catalyzed Homologation of Aromatic Aldehydes with Trimethylsilyldiazomethane. *J. Am. Chem. Soc.* **2001**, *123*, 2442-2443.

9. NMR spectra and SFC chromatograms

N-((*E*)-2-((*Z*)-4-Fluorobenzylidene)benzofuran-3(2*H*)-ylidene)-4-methylbenzenesulfonamide (1c)

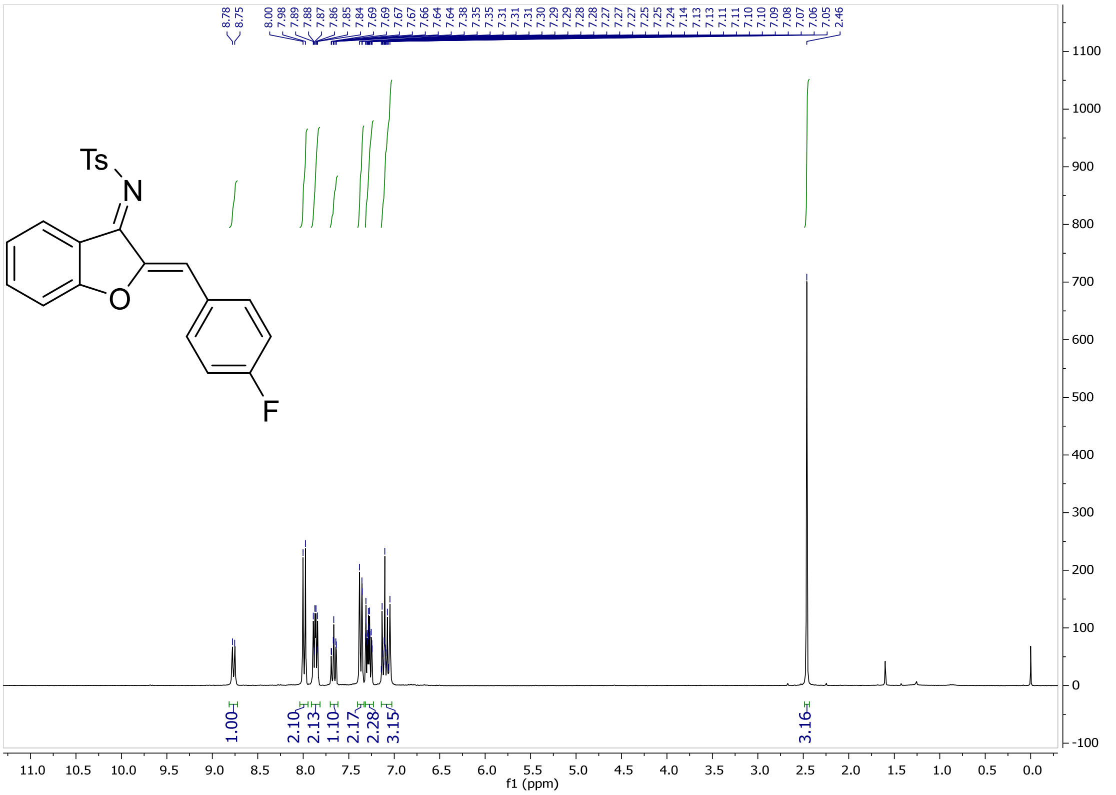

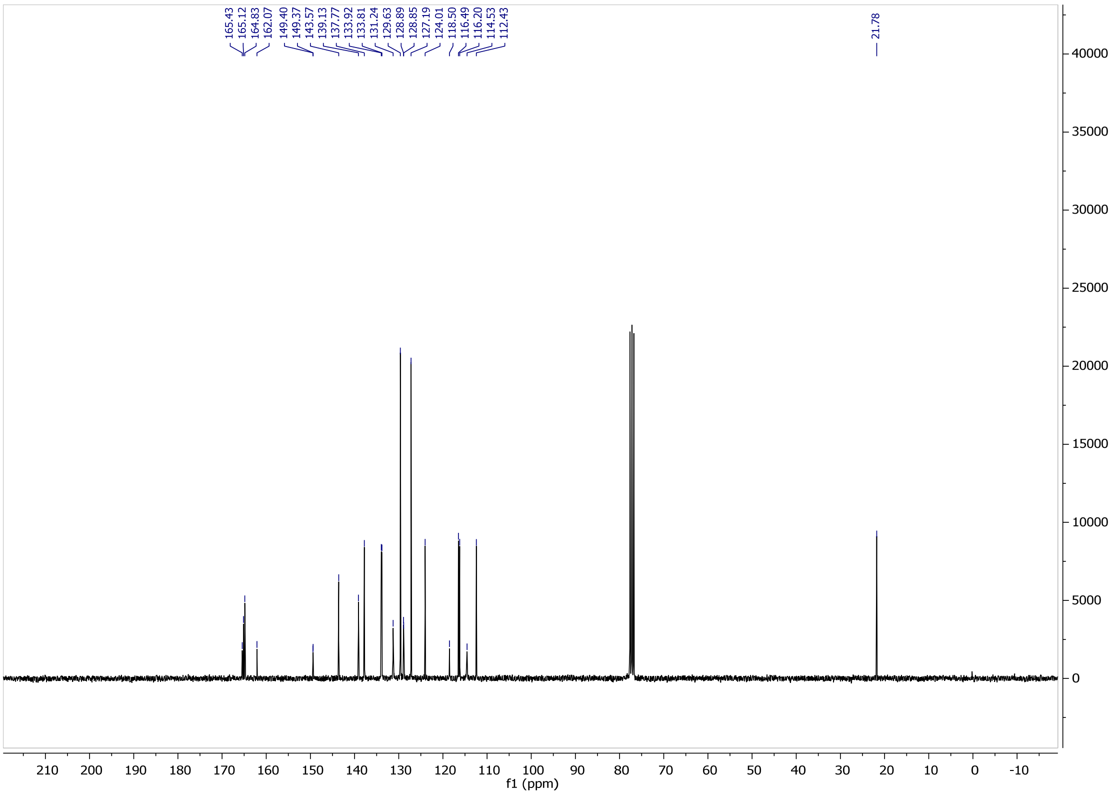

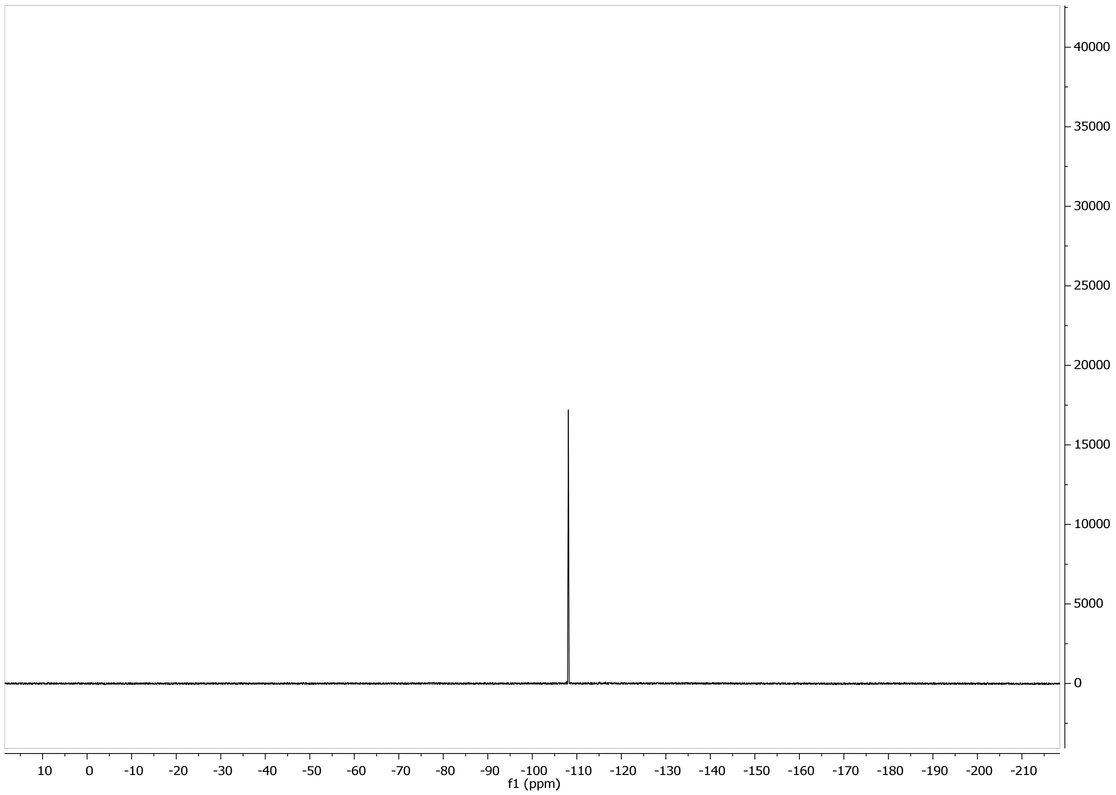

40000
35000
30000
25000
20000
15000
10000
5000
0
10
0
-10
-20
-30
-40
-50
-60
-70
-80
-90
-100
-110
-120
-130
-140
-150
-160
-170
-180
-190
-200
-210
f1 (ppm)

4-Methyl-*N*-((*E*)-2-((*Z*)-4-nitrobenzylidene)benzofuran-3(2*H*)-ylidene)benzenesulfonamide (1d)

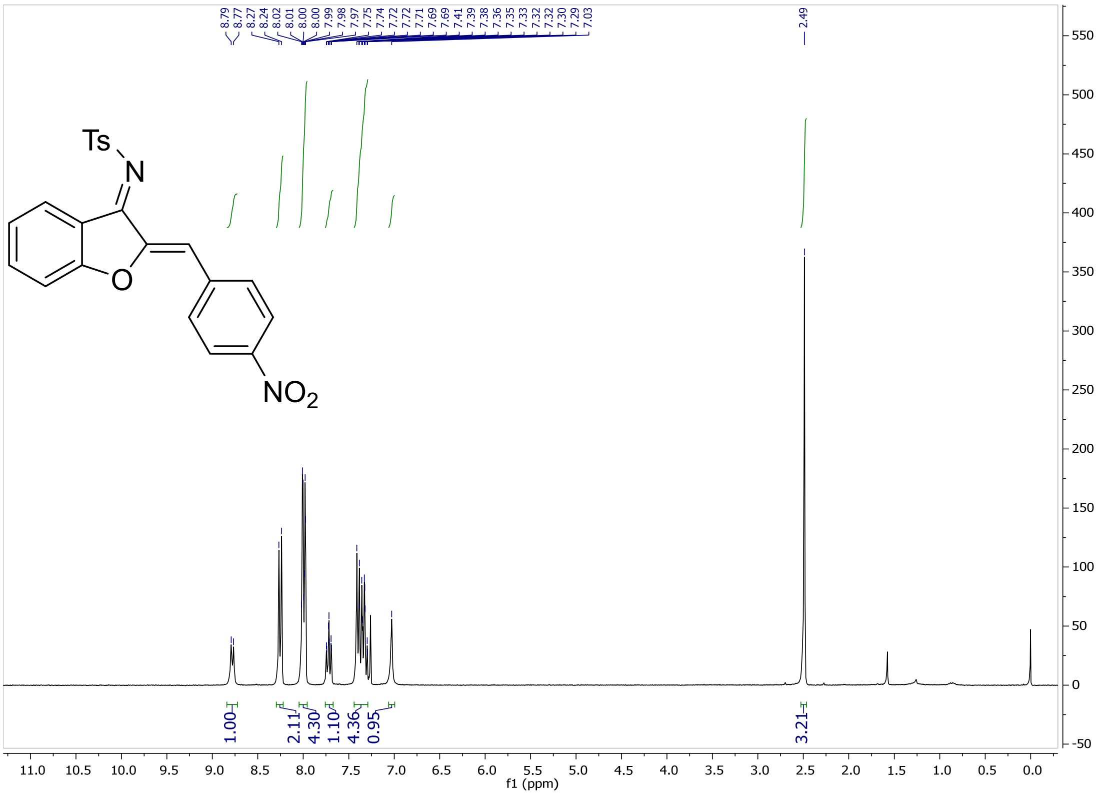

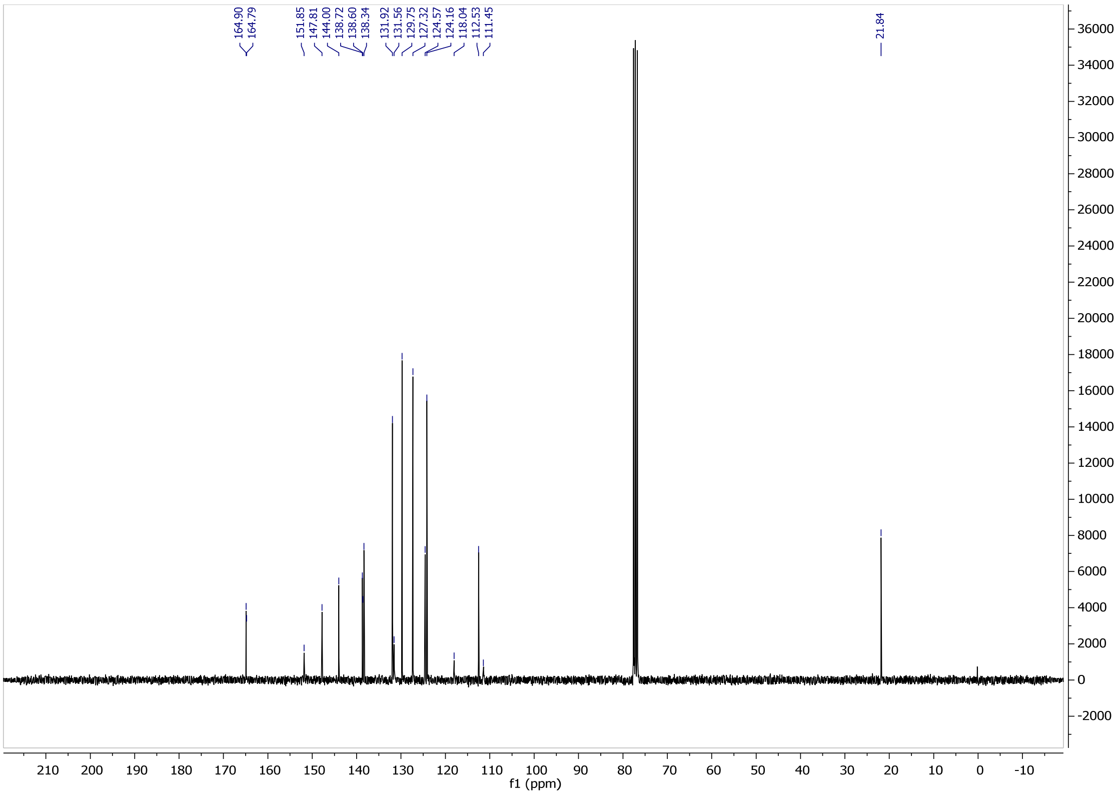

N-((E)-2-((Z)-4-Cyanobenzylidene)benzofuran-3(2H)-ylidene)-4-methylbenzenesulfonamide (1f)

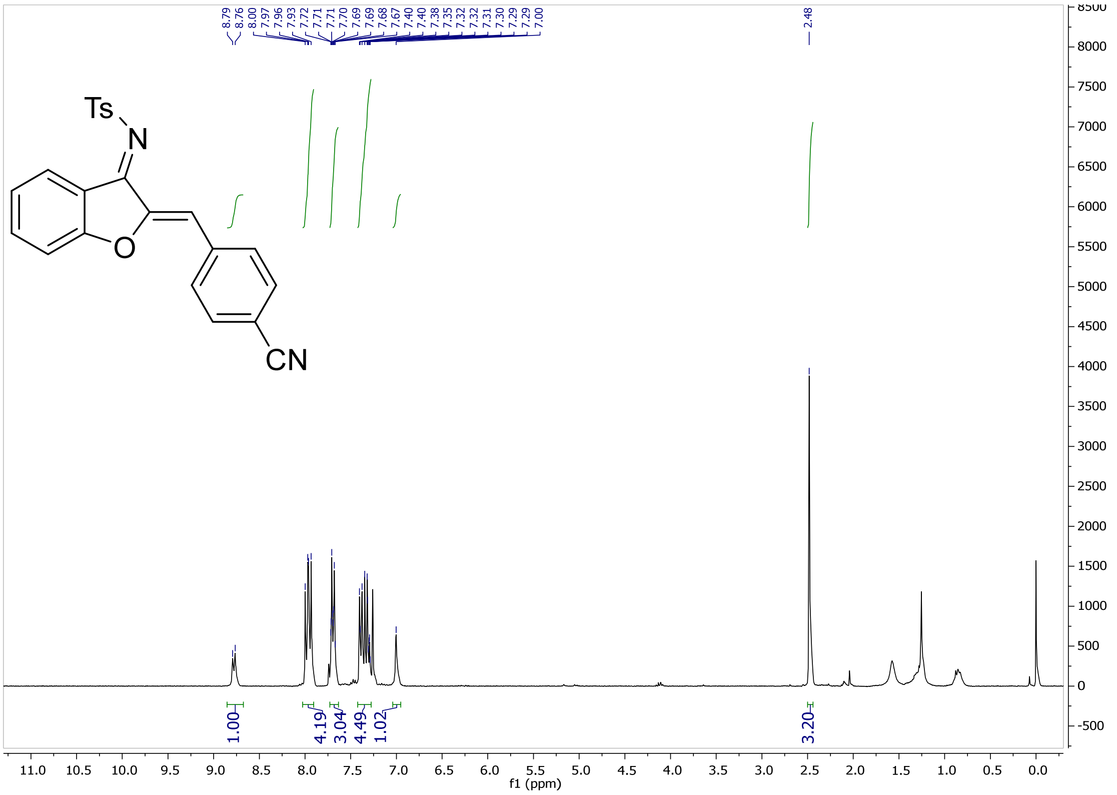

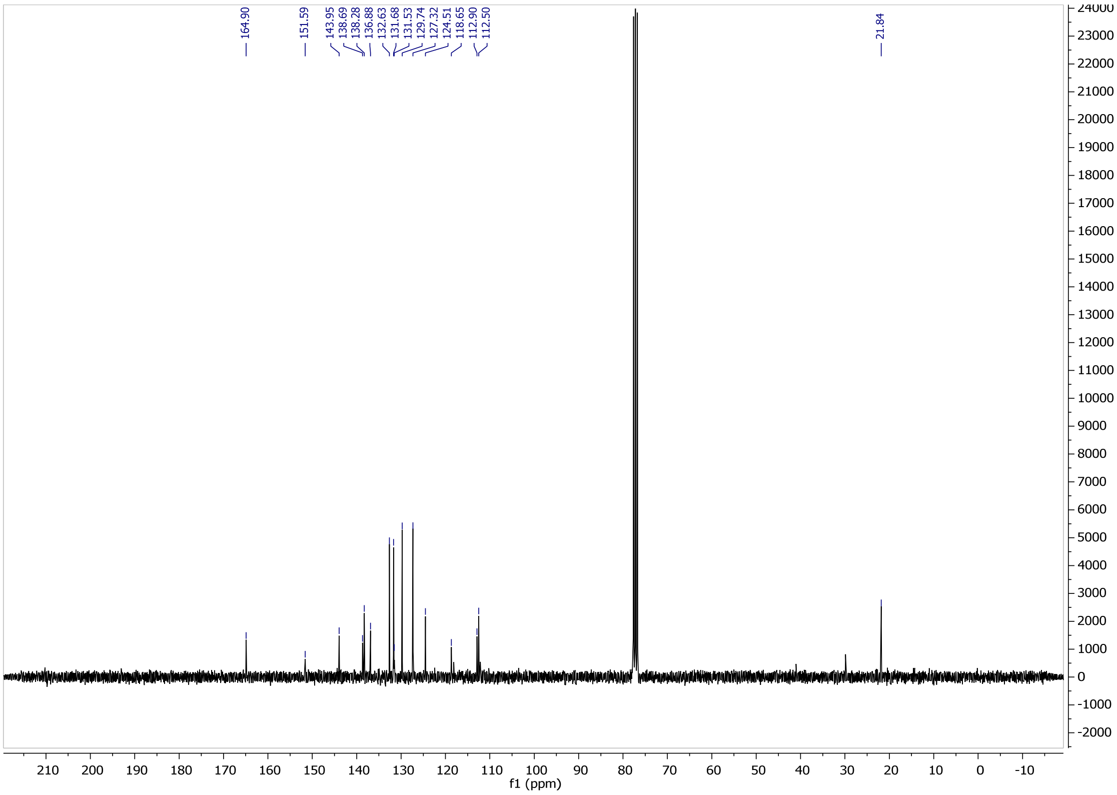

N-((E)-2-((Z)-2-Fluorobenzylidene)benzofuran-3(2H)-ylidene)-4-methylbenzenesulfonamide (1h)

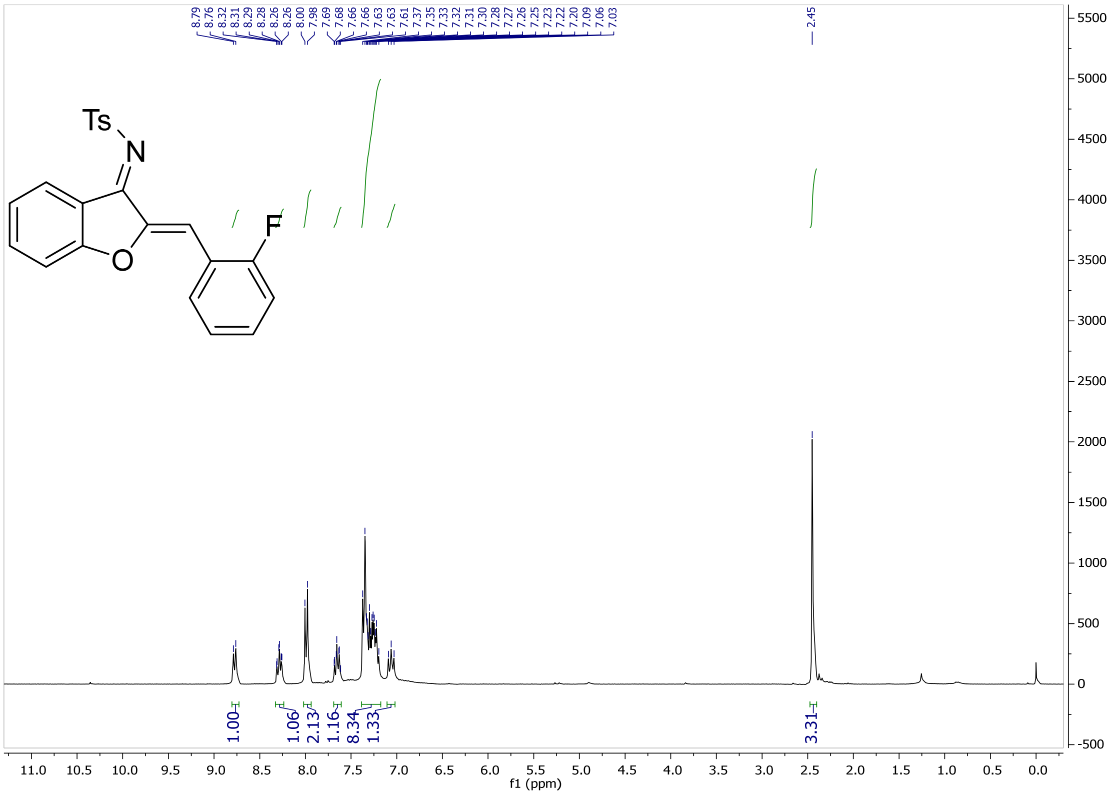

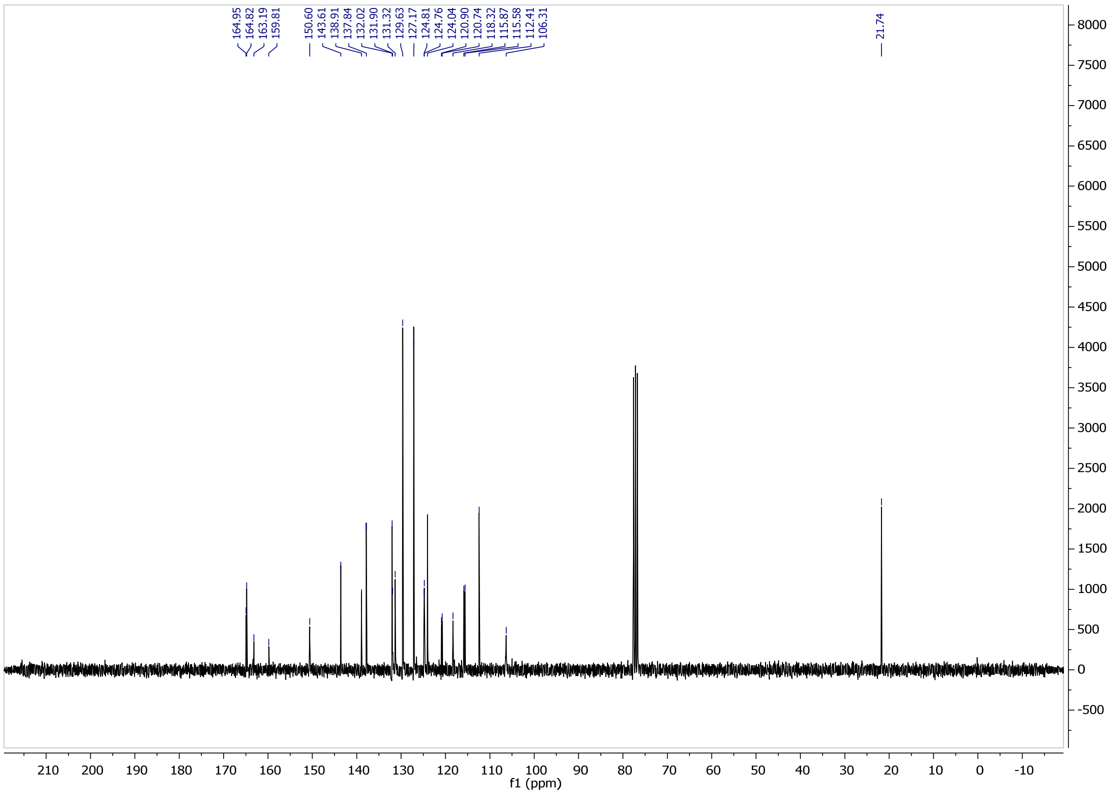

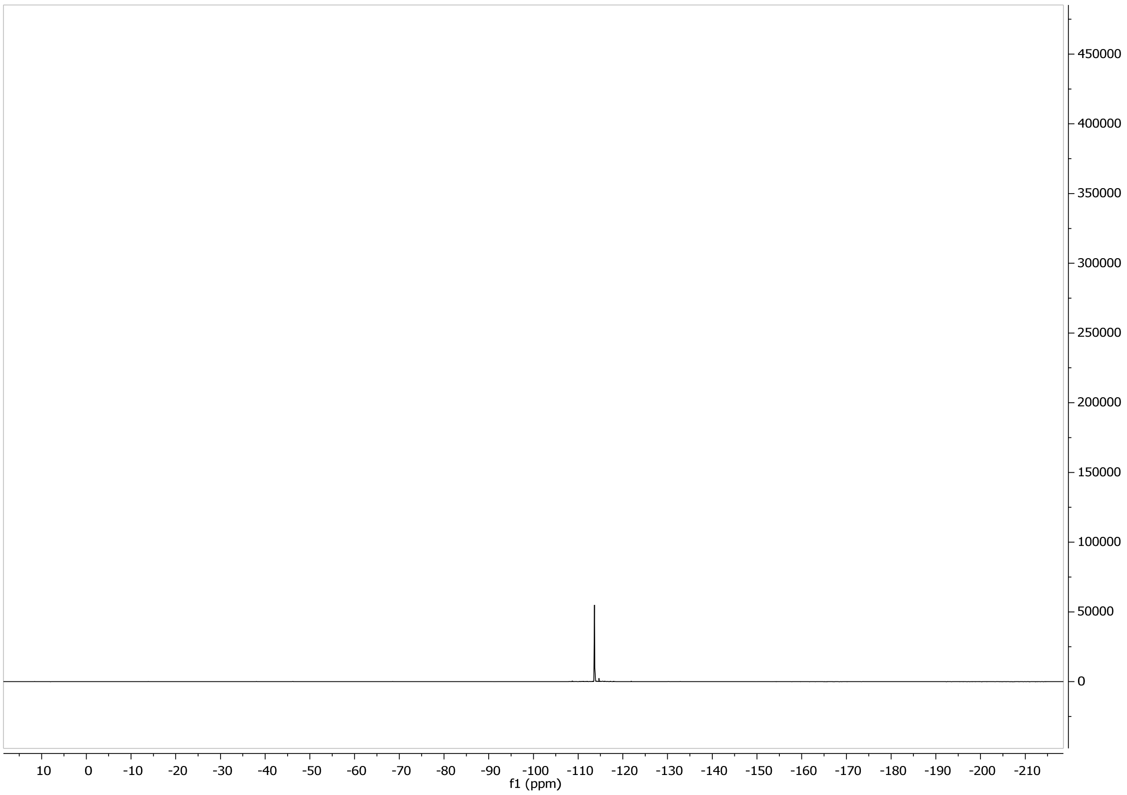
450000
400000
350000
300000
250000
200000
150000
100000
50000
0
10 0 -10 -20 -30 -40 -50 -60 -70 -80 -90 -100 -110 -120 -130 -140 -150 -160 -170 -180 -190 -200 -210
f1 (ppm)

_N_-((_E_)-2-((_Z_)-3-Methoxybenzylidene)benzofuran-3(2_H_)-ylidene)-4-methyl benzenesulfonamide (1i)

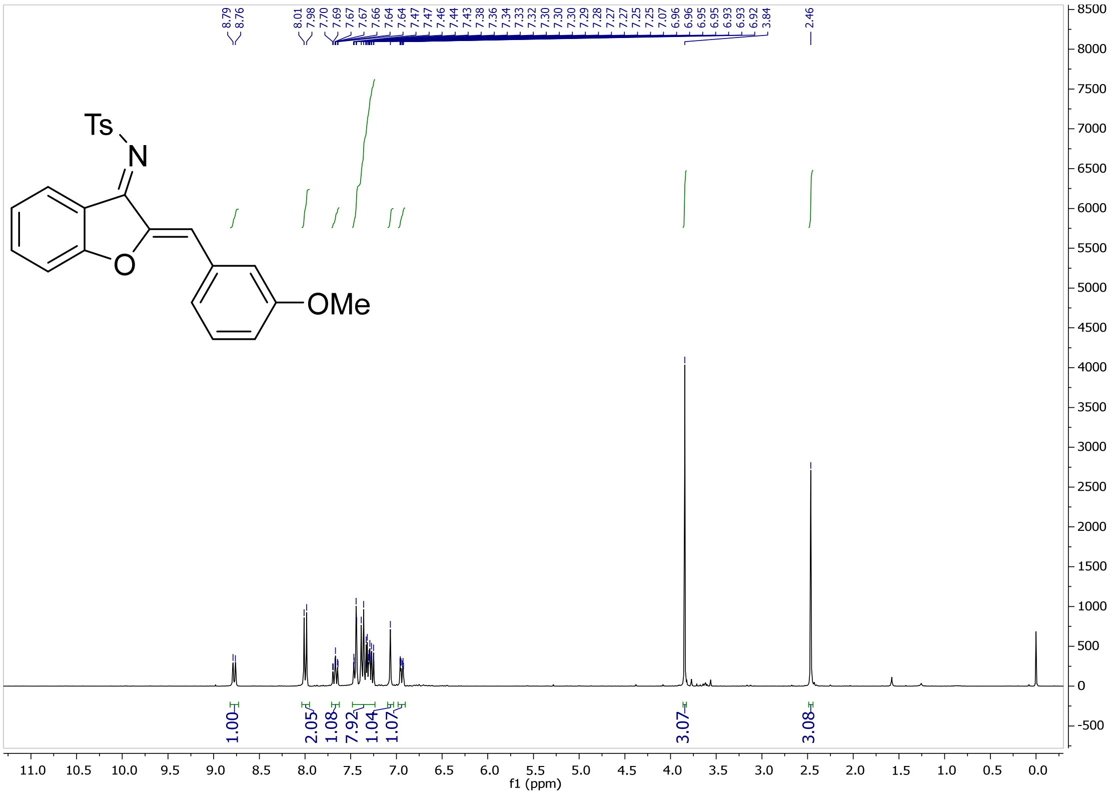

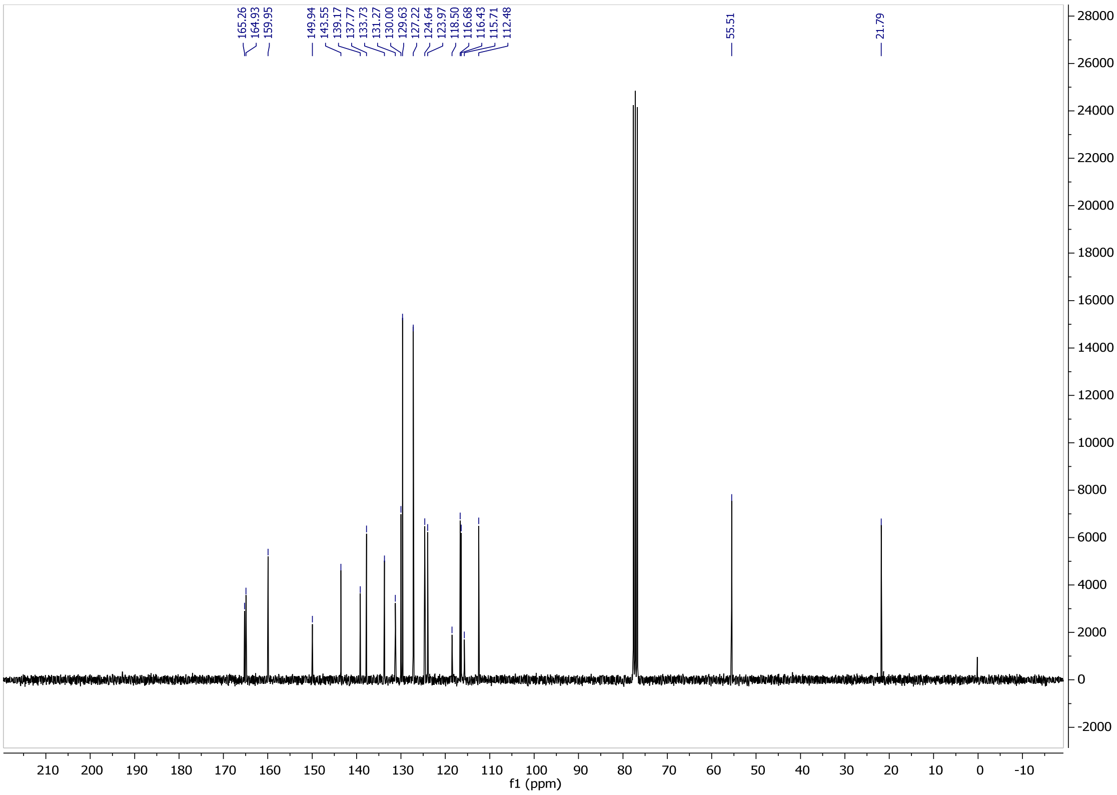

_N_-((_E_)-2-((_Z_)-benzylidene)-6-bromobenzofuran-3(_2H_)-ylidene)-4-methylbenzenesulfonamide (1m)

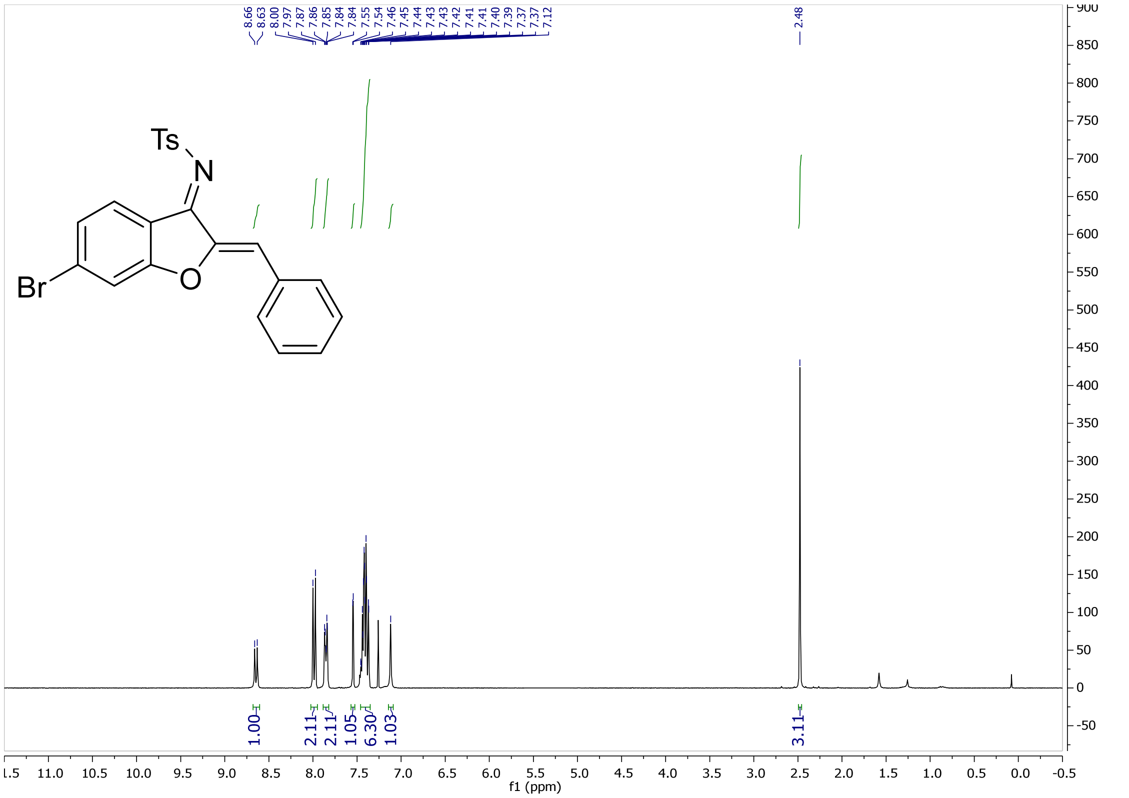

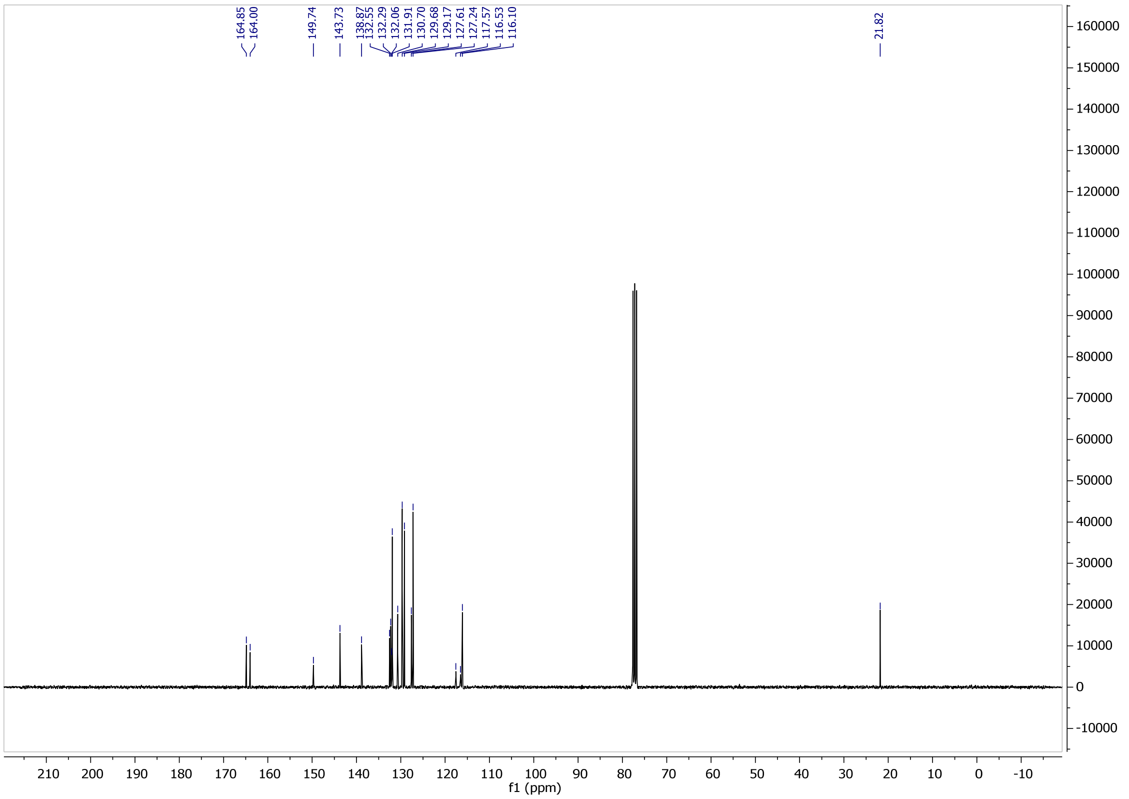

(*2R,3R,4S*)-4-Phenyl-1-tosyl-3-vinyl-1,2,3,4-tetrahydrobenzofuro[3,2-*b*]pyridin-2-ol (4a)

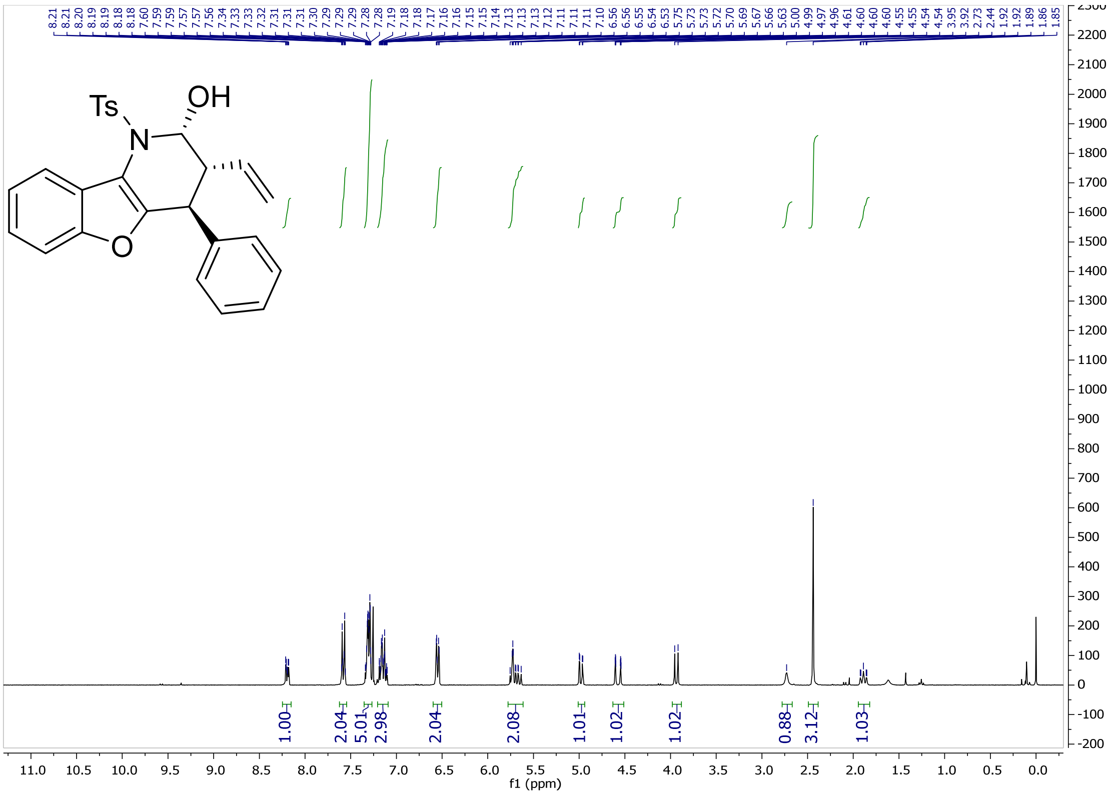

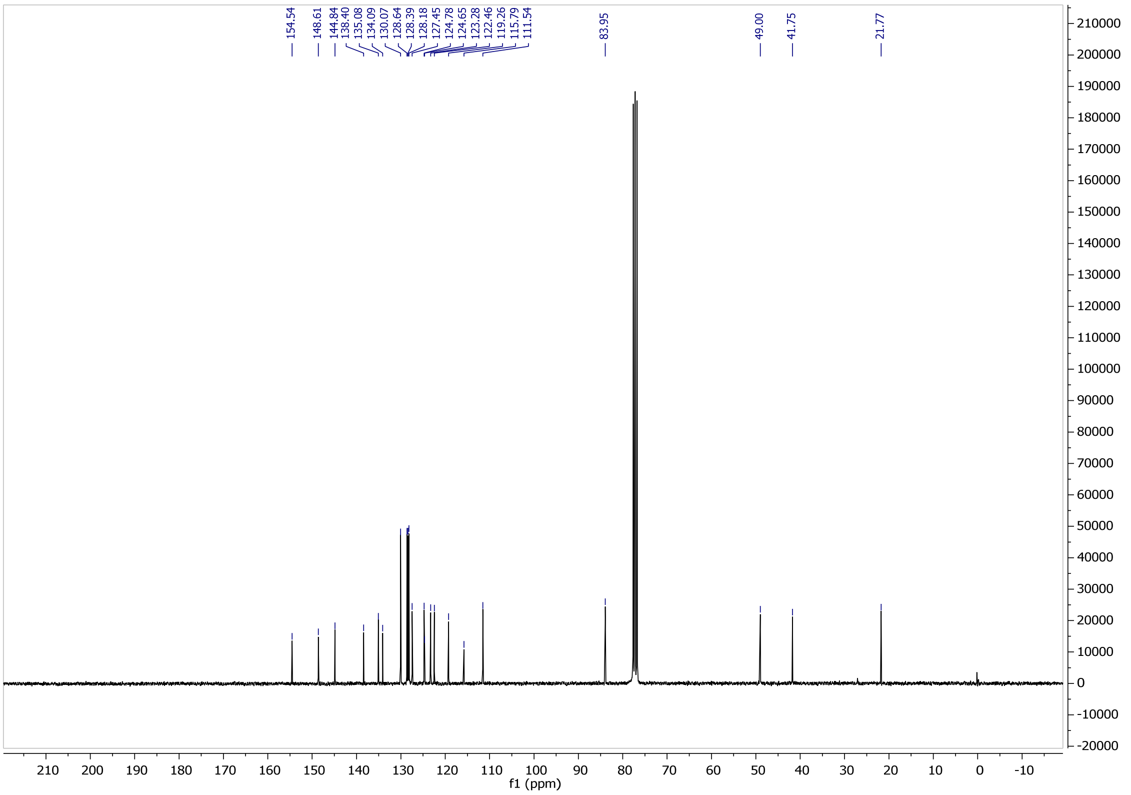

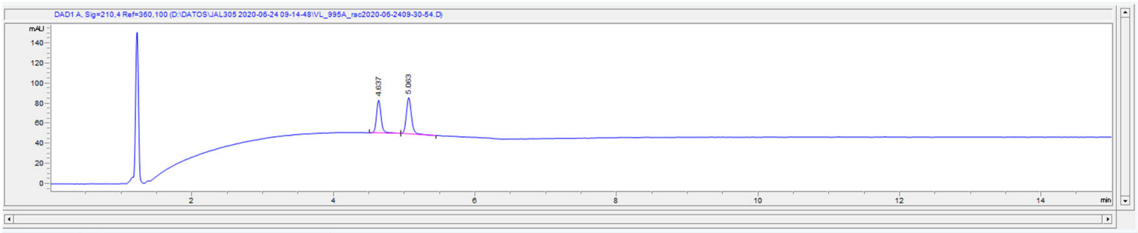

File Information

LC-File	VL_995A_rac2020-06-2409-30-54.D
File Path	D:\DATOS\JAL305 2020-06-24 09-14-48
Date	24-Jun-20, 09:34:20
Sample	VL_995A_rac
Sample Info	VL_995A_rac
Barcode	
Operator	SYSTEM

#	Time	Type	Area	Height	Width	Area%	Symmetry
1	4.637	BB	145.5	32.6	0.0681	44.956	0.832
2	5.063	BB	178.2	35.8	0.078	55.044	0.81

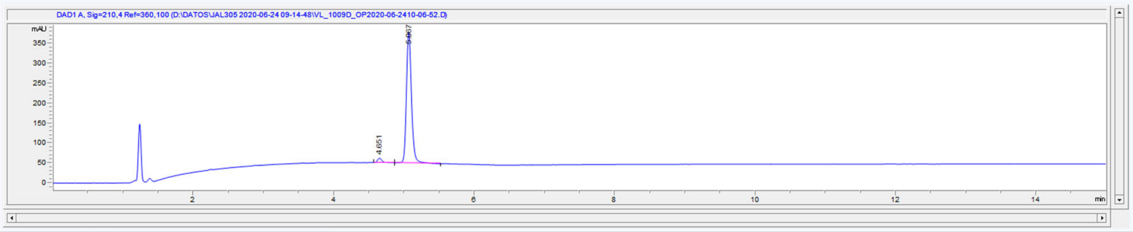

File Information

LC-File	VL_1009D_OP2020-06-2410-06-52.D
File Path	D:\DATOS\JAL305 2020-06-24 09-14-48
Date	24-Jun-20, 10:10:15
Sample	VL_1009D_OP
Sample Info	VL_1009D_OP
Barcode	
Operator	SYSTEM

#	Time	Type	Area	Height	Width	Area%	Symmetry
1	4.651	BB	49.3	11	0.0685	2.917	0.765
2	5.067	BB	1641.6	328.7	0.0783	97.083	0.786

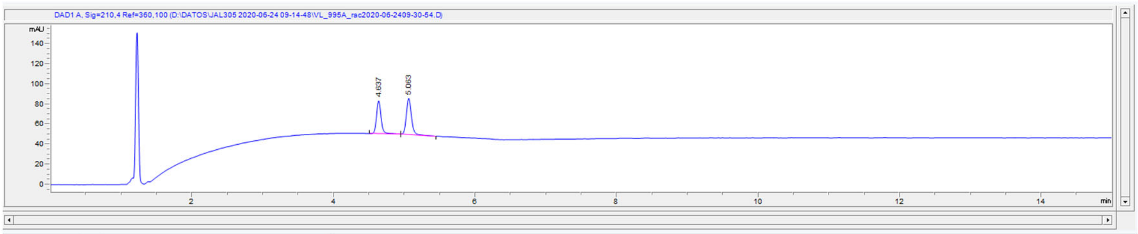

File Information

LC-File	VL_995A_rac2020-06-2409-30-54.D
File Path	D:\DATOS\JAL305 2020-06-24 09-14-48
Date	24-Jun-20, 09:34:20
Sample	VL_995A_rac
Sample Info	VL_995A_rac
Barcode	
Operator	SYSTEM

#	Time	Type	Area	Height	Width	Area%	Symmetry
1	4.637	BB	145.5	32.6	0.0681	44.956	0.832
2	5.063	BB	178.2	35.8	0.078	55.044	0.81

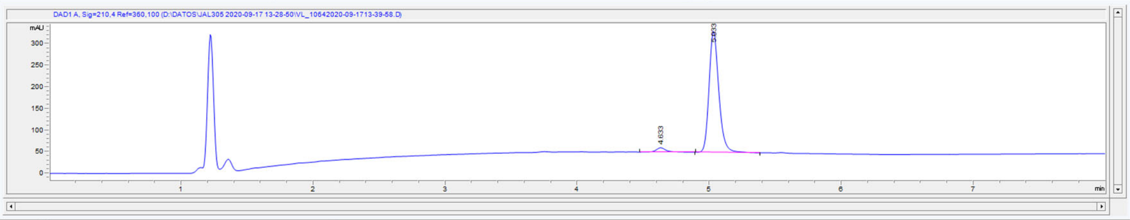

File Information

LC-File	VL_10642020-09-1713-39-58.D
File Path	D:\DATOS\JAL305 2020-09-17 13-28-50
Date	17-Sep-20, 13:43:29
Sample	VL_1064
Sample Info	VL_1064
Barcode	
Operator	SYSTEM
Method	IA-gradiente5_40_MeOH_MS.M
Reference	D:\DATOS\JAL305 2020-09-17 13-28-50\no Sample Name2020-09-:

#	Time	Type	Area	Height	Width	Area%	Symmetry
1	4.633	BB	52.5	10	0.079	3.521	0.792
2	5.033	BB	1439.8	280.5	0.0779	96.479	0.769

(*2R,3R,4S*)-4-(4-Bromophenyl)-1-tosyl-3-vinyl-1,2,3,4-tetrahydrobenzofuro[3,2-*b*]pyridin-2-ol (4b)

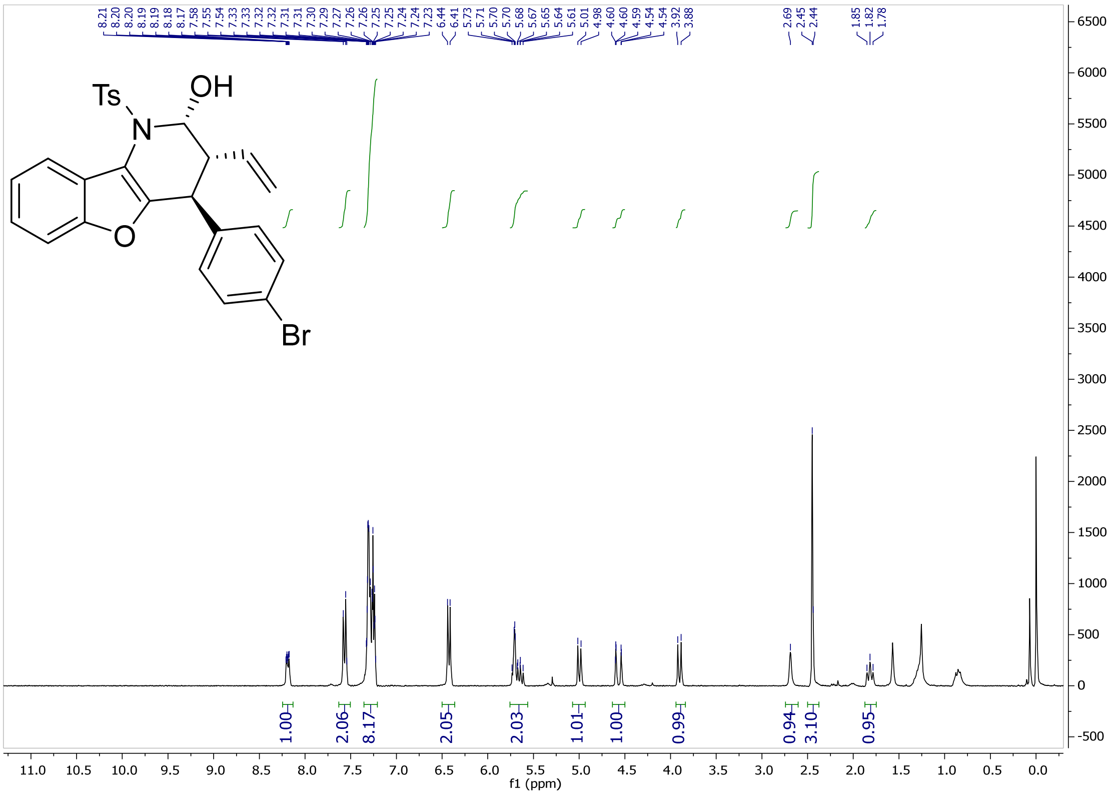

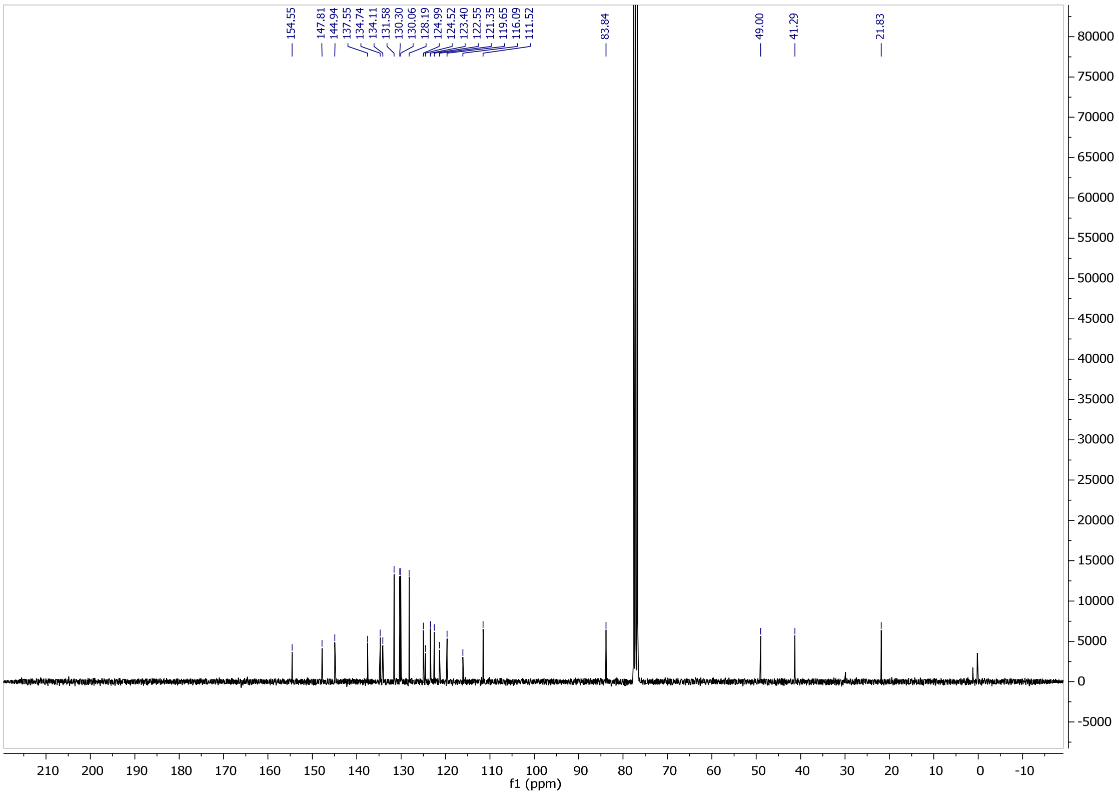

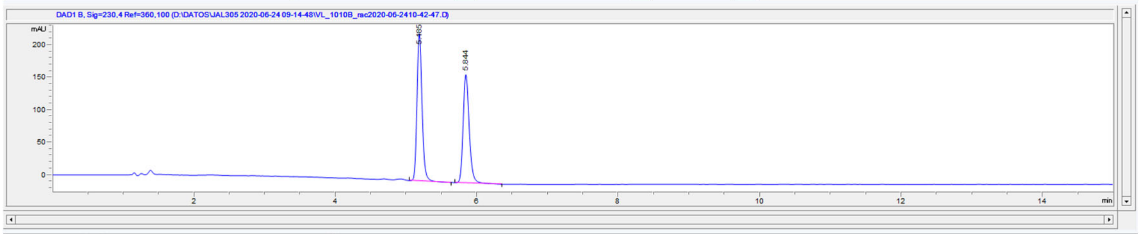

File Information

LC-File	VL_1010B_rac2020-06-2410-42-47.D
File Path	D:\DATOS\JAL305 2020-06-24 09-14-48
Date	24-Jun-20, 10:46:13
Sample	VL_1010B_rac
Sample Info	VL_1010B_rac
Barcode	
Operator	SYSTEM

#	Time	Type	Area	Height	Width	Area%	Symmetry
1	5.185	BB	1137.6	229	0.076	53.112	0.806
2	5.844	BB	1004.3	167.7	0.0919	46.888	0.761

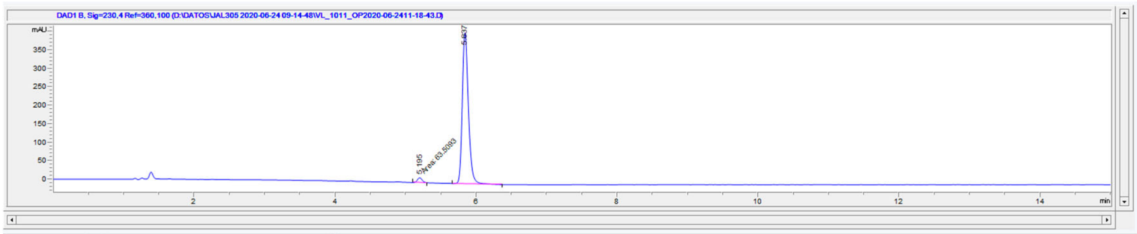

File Information

LC-File	VL_1011_OP2020-06-2411-18-43.D
File Path	D:\DATOS\JAL305 2020-06-24 09-14-48
Date	24-Jun-20, 11:22:15
Sample	VL_1011_OP
Sample Info	VL_1011_OP
Barcode	
Operator	SYSTEM

#	Time	Type	Area	Height	Width	Area%	Symmetry
1	5.195	MM	63.5	13.2	0.0799	2.536	0.894
2	5.837	BB	2440.9	407.5	0.0919	97.464	0.734

(*2R,3R,4S*)-4-(4-Fluorophenyl)-1-tosyl-3-vinyl-1,2,3,4-tetrahydrobenzofuro[3,2-*b*]pyridin-2-ol (4c)

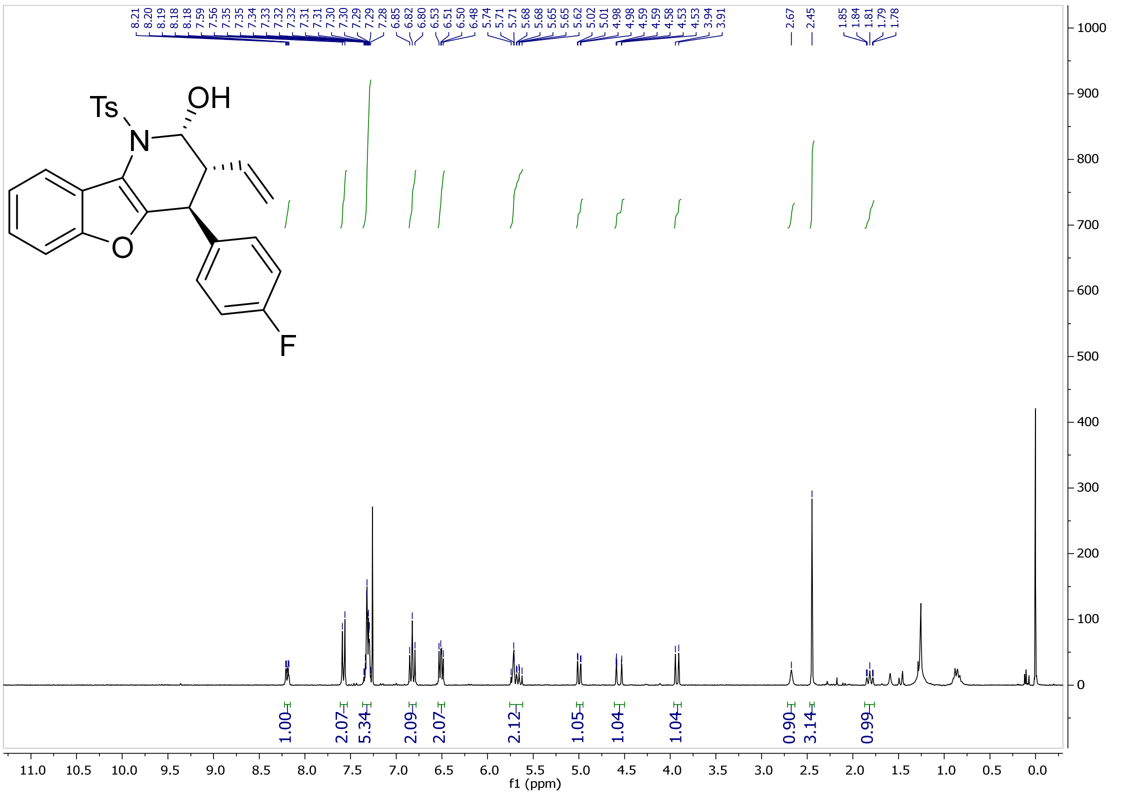

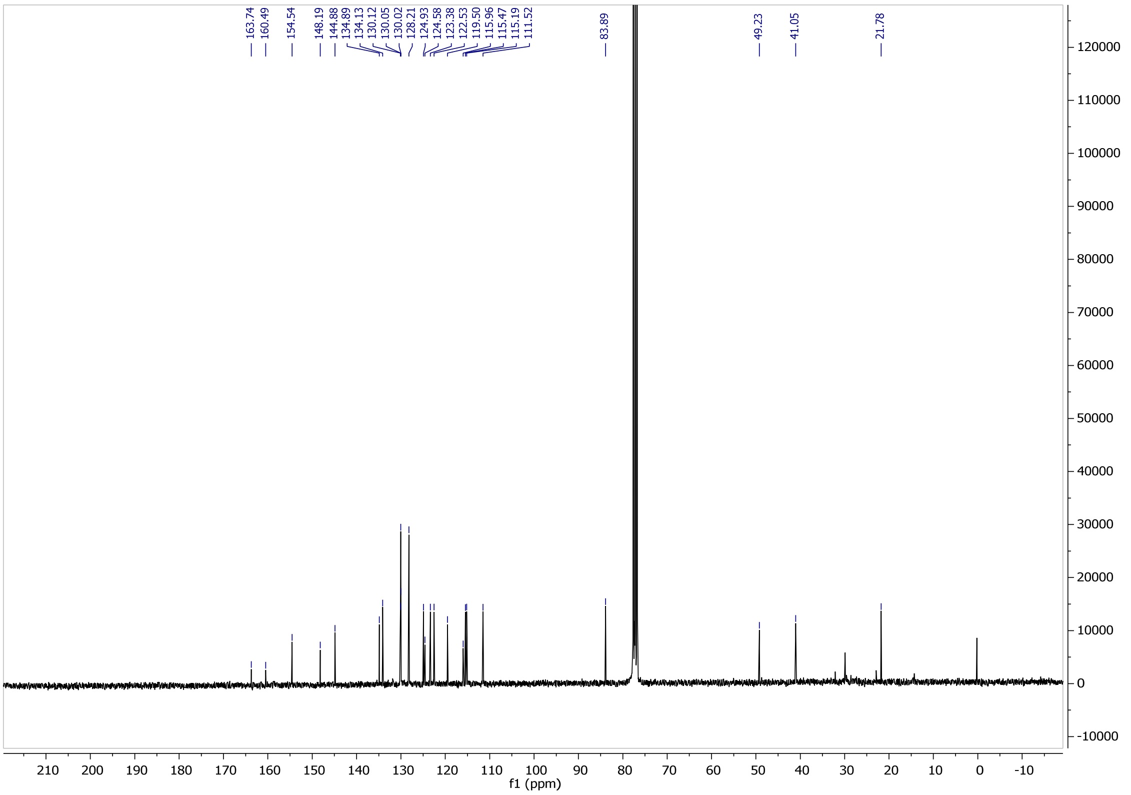

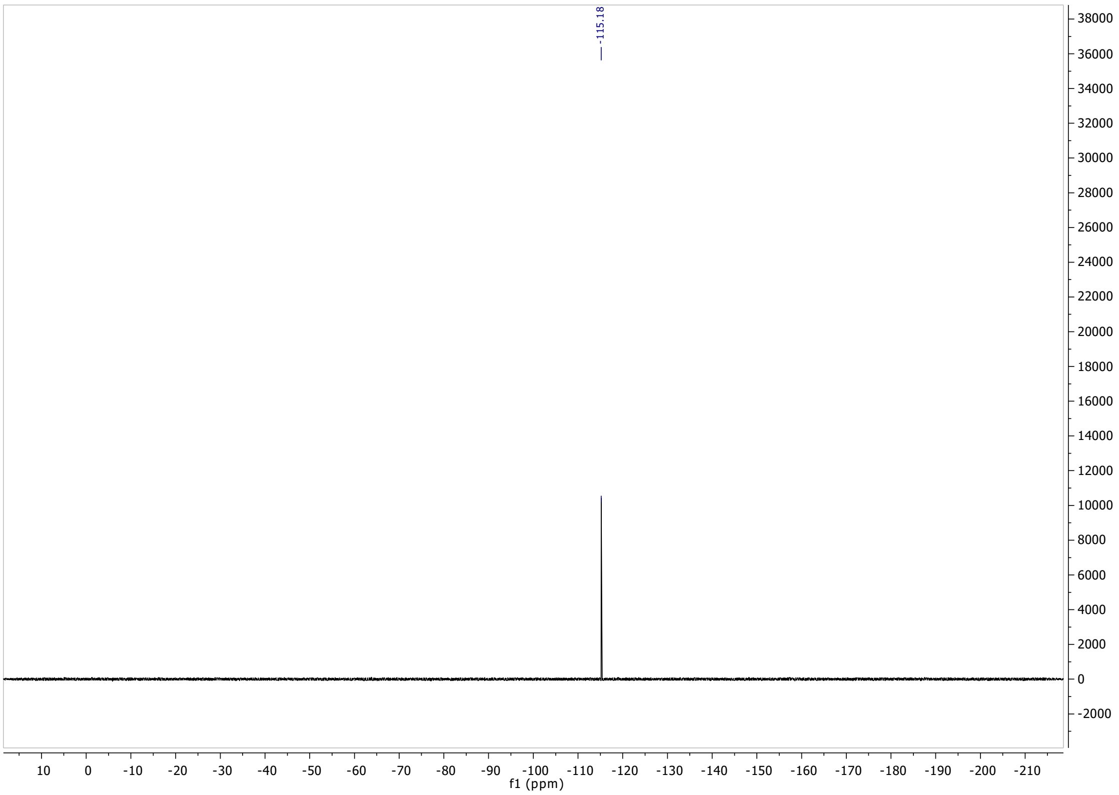

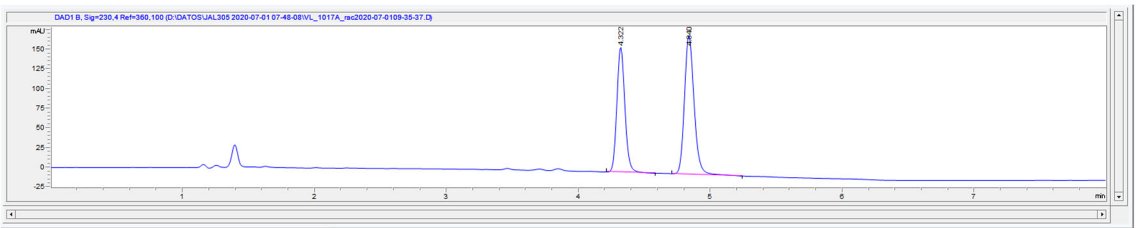

File Information

LC-File	VL_1017A_rac2020-07-0109-35-37.D
File Path	D:\DATOS\JAL305 2020-07-01 07-48-08
Date	01-Jul-20, 09:38:54
Sample	VL_1017A_rac
Sample Info	VL_1017A_rac
Barcode	
Operator	SYSTEM

#	Time	Type	Area	Height	Width	Area%	Symmetry
1	4.322	BB	681.5	160	0.0657	43.074	0.851
2	4.84	BB	900.6	177.5	0.0772	56.926	0.792

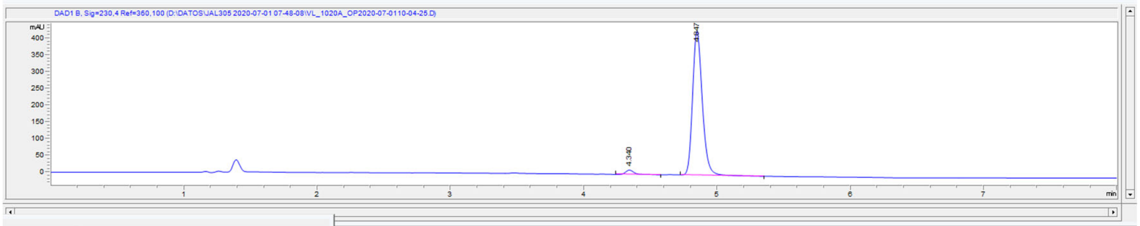

File Information

LC-File	VL_1020A_OP2020-07-0110-04-25.D
File Path	D:\DATOS\JAL305 2020-07-01 07-48-08
Date	01-Jul-20, 10:07:53
Sample	VL_1020A_OP
Sample Info	VL_1020A_OP
Barcode	
Operator	SYSTEM

#	Time	Type	Area	Height	Width	Area%	Symmetry
1	4.34	BB	61.3	13.4	0.0693	2.732	0.72
2	4.847	BB	2183.6	430.9	0.0771	97.268	0.76

(*2R,3R,4S*)-4-(4-Nitrophenyl)-1-tosyl-3-vinyl-1,2,3,4-tetrahydrobenzofuro[3,2-*b*]pyridin-2-ol (4d)

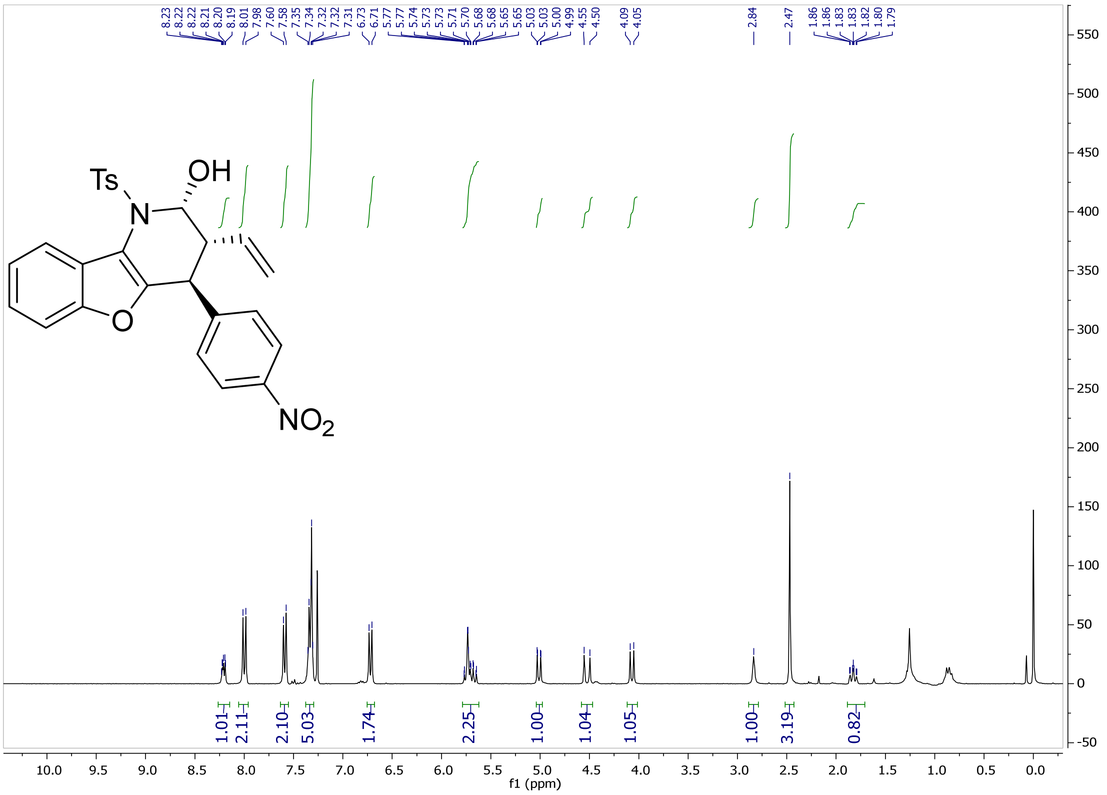

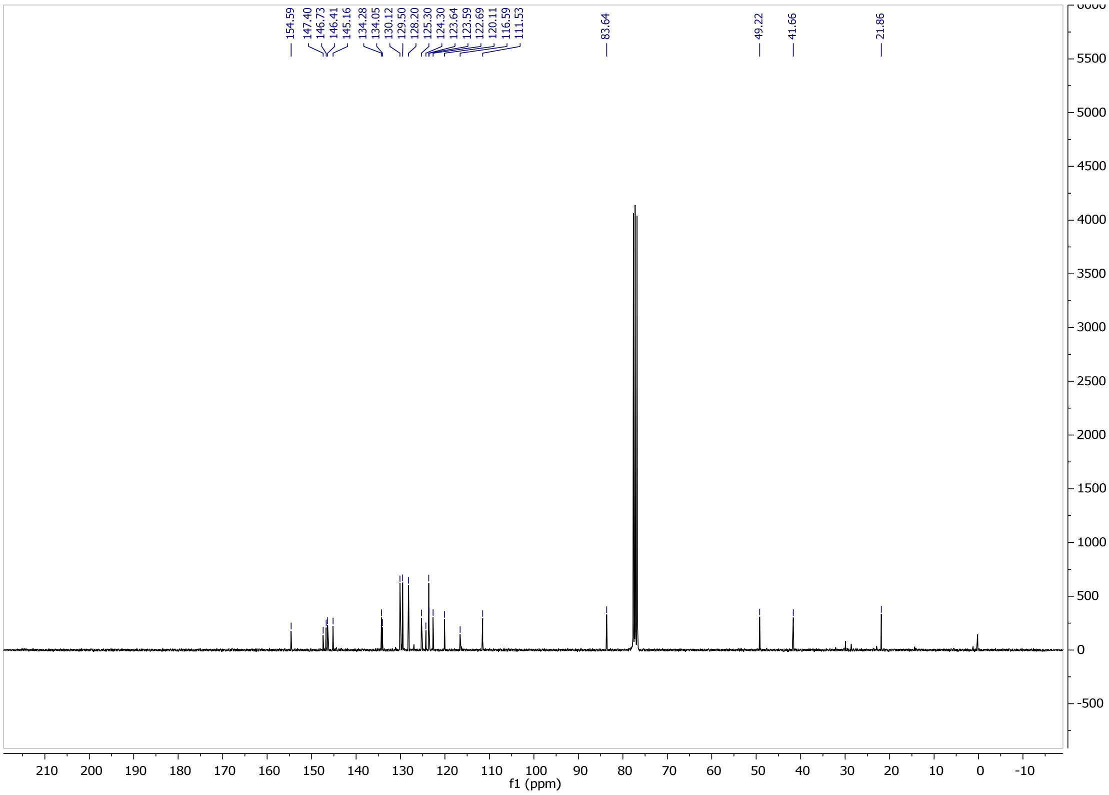

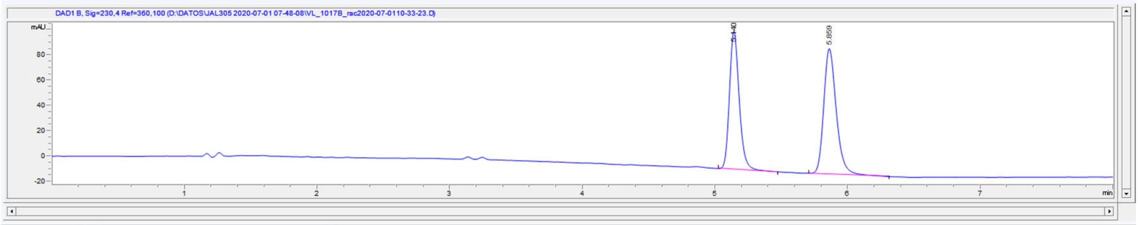

File Information

LC-File	VL_1017B_rac2020-07-0110-33-23.D
File Path	D:\DATOS\JAL305 2020-07-01 07-48-08
Date	01-Jul-20, 10:36:53
Sample	VL_1017B_rac
Sample Info	VL_1017B_rac
Barcode	
Operator	SYSTEM

#	Time	Type	Area	Height	Width	Area%	Symmetry
1	5.14	BB	559.2	109.8	0.0774	46.079	0.774
2	5.859	BB	654.4	99.5	0.1007	53.921	0.736

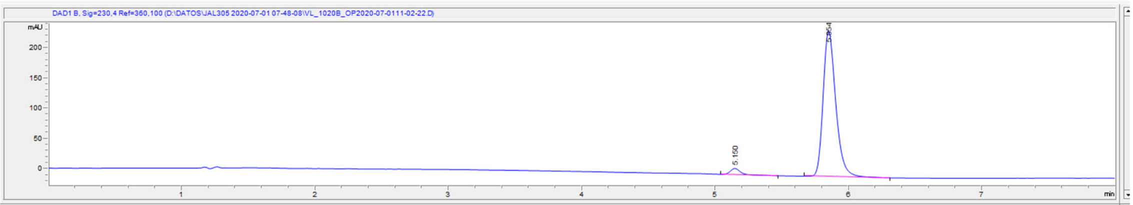

File Information

LC-File	VL_1020B_OP2020-07-0111-02-22.D
File Path	D:\DATOS\JAL305 2020-07-01 07-48-08
Date	01-Jul-20, 11:05:47
Sample	VL_1020B_OP
Sample Info	VL_1020B_OP
Barcode	
Operator	SYSTEM

#	Time	Type	Area	Height	Width	Area%	Symmetry
1	5.15	BB	54.5	10.3	0.082	3.316	0.728
2	5.854	BB	1587.8	241.1	0.1008	96.684	0.709

(*2R,3R,4S*)-1-Tosyl-4-(4-(trifluoromethyl)phenyl)-3-vinyl-1,2,3,4-tetrahydrobenzofuro[3,2-*b*]pyridin-2-ol (4e)

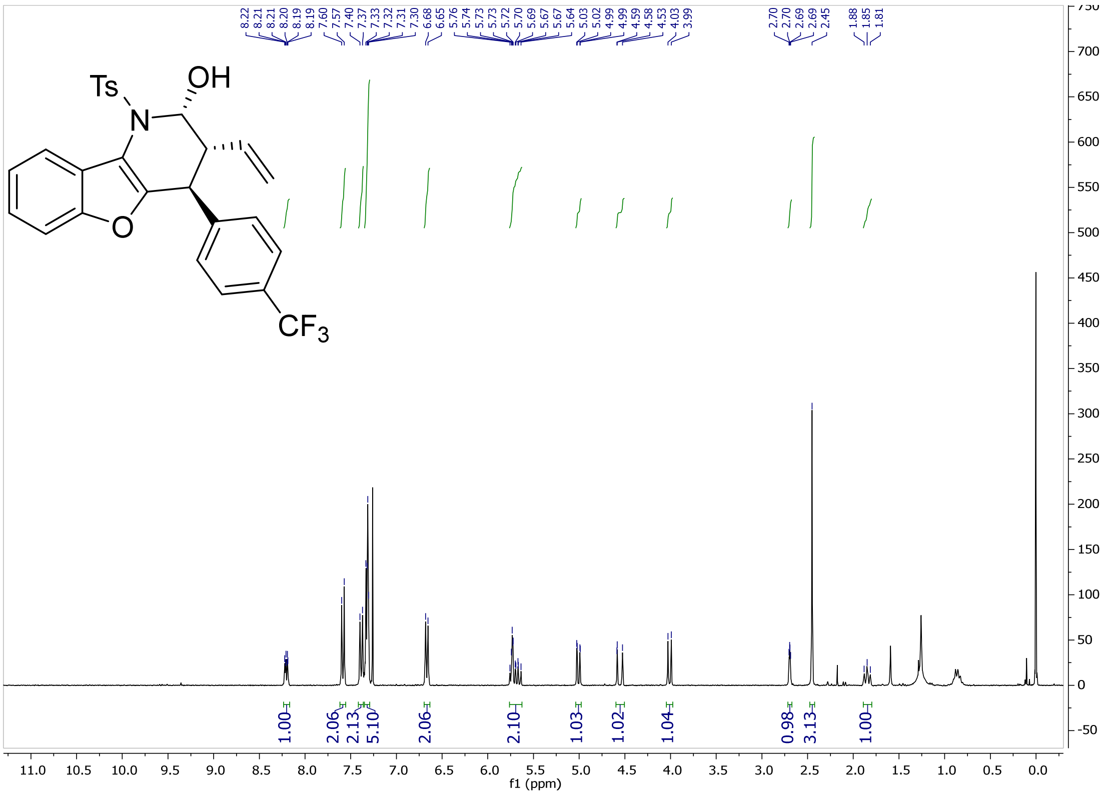

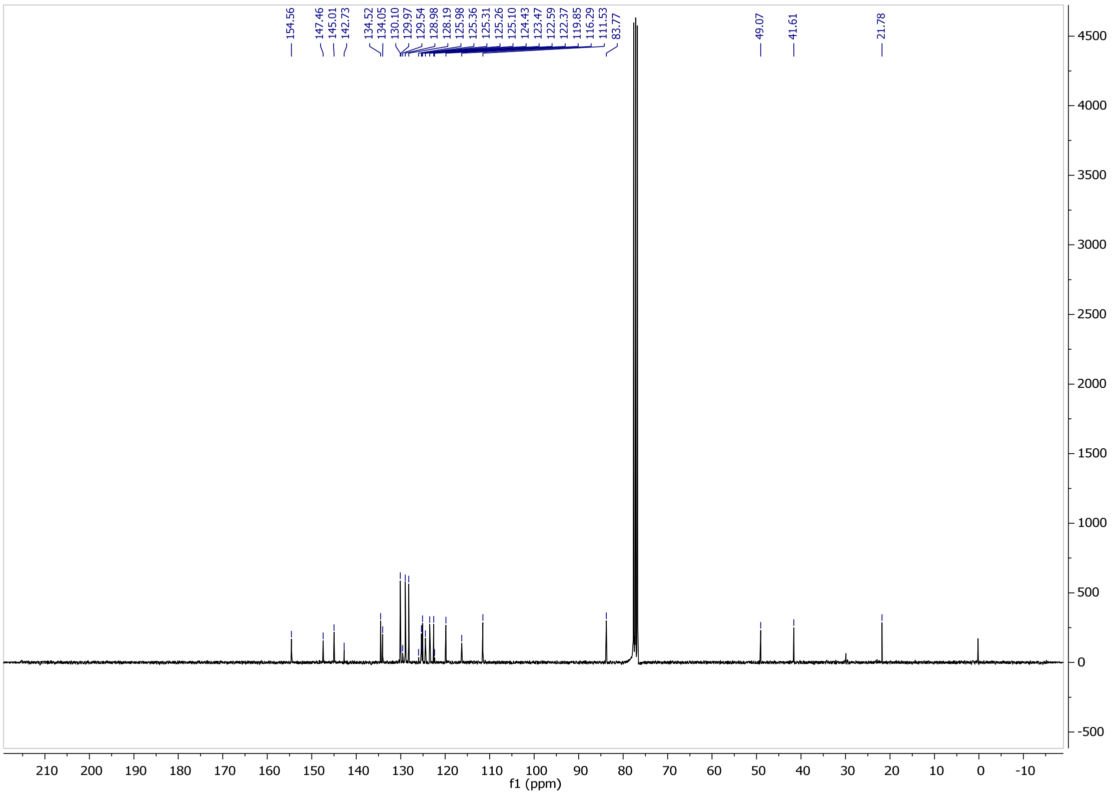

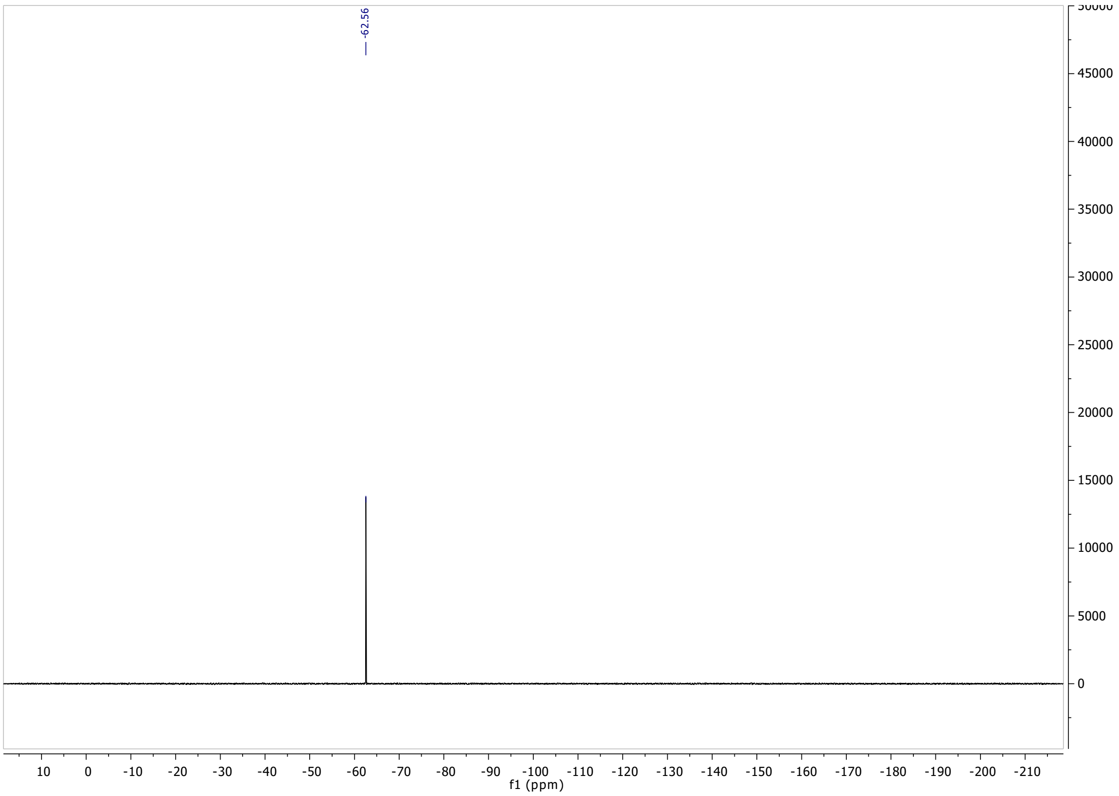

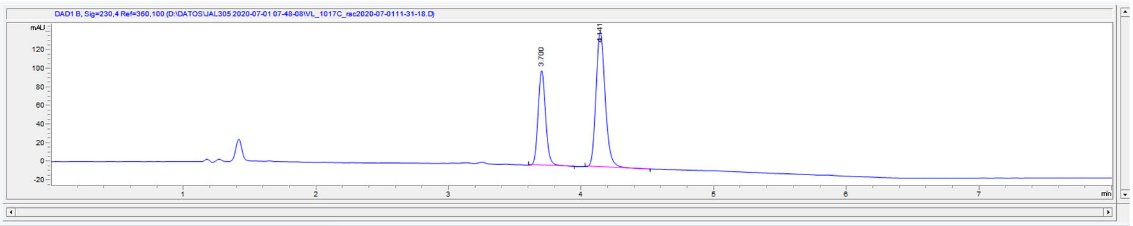

File Information

LC-File	VL_1017C_rac2020-07-0111-31-18.D
File Path	D:\DATOS\JAL305 2020-07-01 07-48-08
Date	01-Jul-20, 11:34:45
Sample	VL_1017C_rac
Sample Info	VL_1017C_rac
Barcode	
Operator	SYSTEM

#	Time	Type	Area	Height	Width	Area%	Symmetry
1	3.7	BB	406.6	102.8	0.0622	36.426	0.842
2	4.141	BB	709.6	146.4	0.0745	63.574	0.779

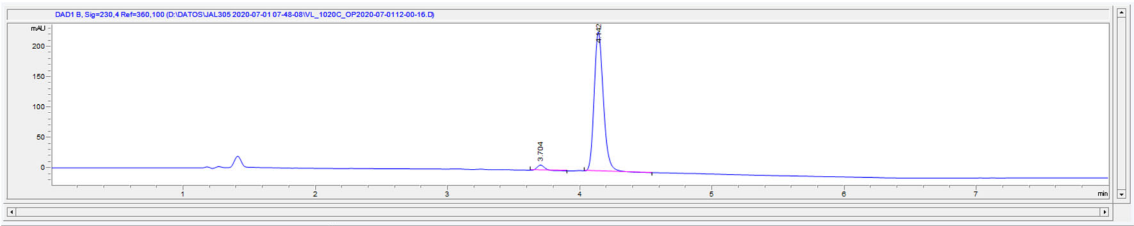

File Information

LC-File	VL_1020C_OP2020-07-0112-00-16.D
File Path	D:\DATOS\JAL305 2020-07-01 07-48-08
Date	01-Jul-20, 12:03:42
Sample	VL_1020C_OP
Sample Info	VL_1020C_OP
Barcode	
Operator	SYSTEM

#	Time	Type	Area	Height	Width	Area%	Symmetry
1	3.704	BB	36.8	8.8	0.0647	3.188	0.76
2	4.142	BB	1117.2	230.8	0.0745	96.812	0.781

4-((*2R,3R,4S*)-2-Hydroxy-1-tosyl-3-vinyl-1,2,3,4-tetrahydrobenzofuro[3,2-*b*]pyridin-4-yl)benzonitrile (4f)

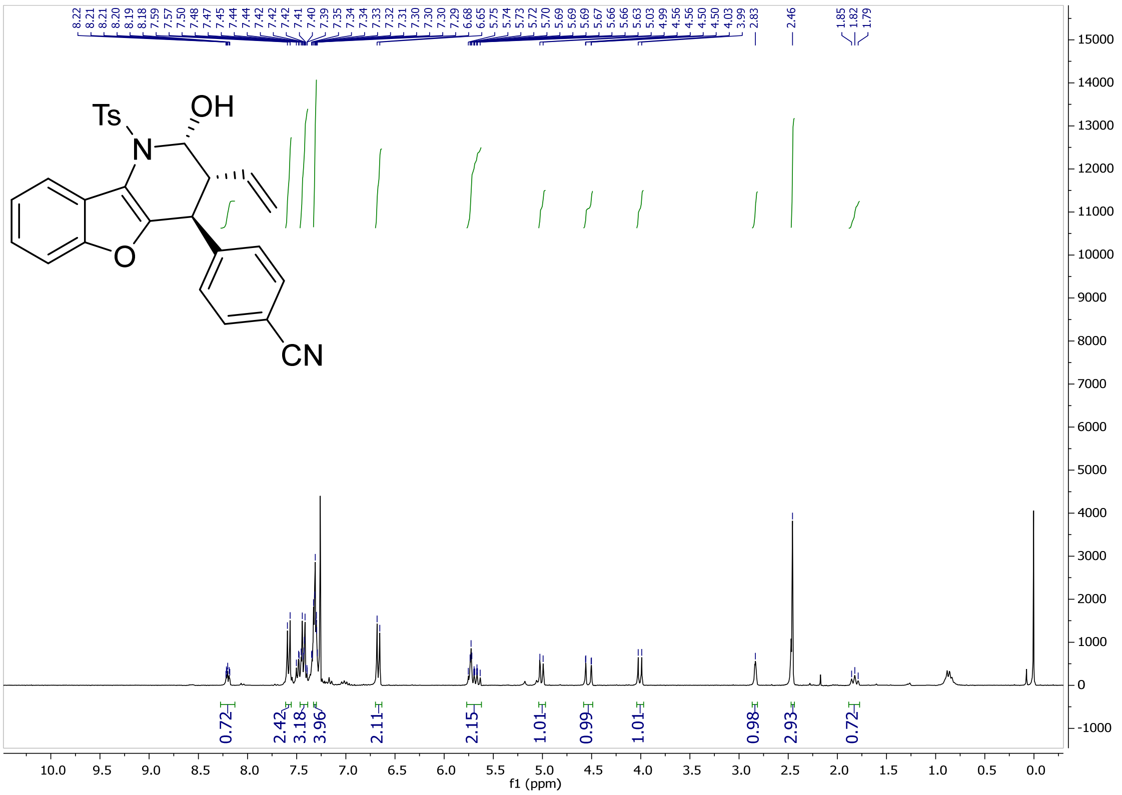

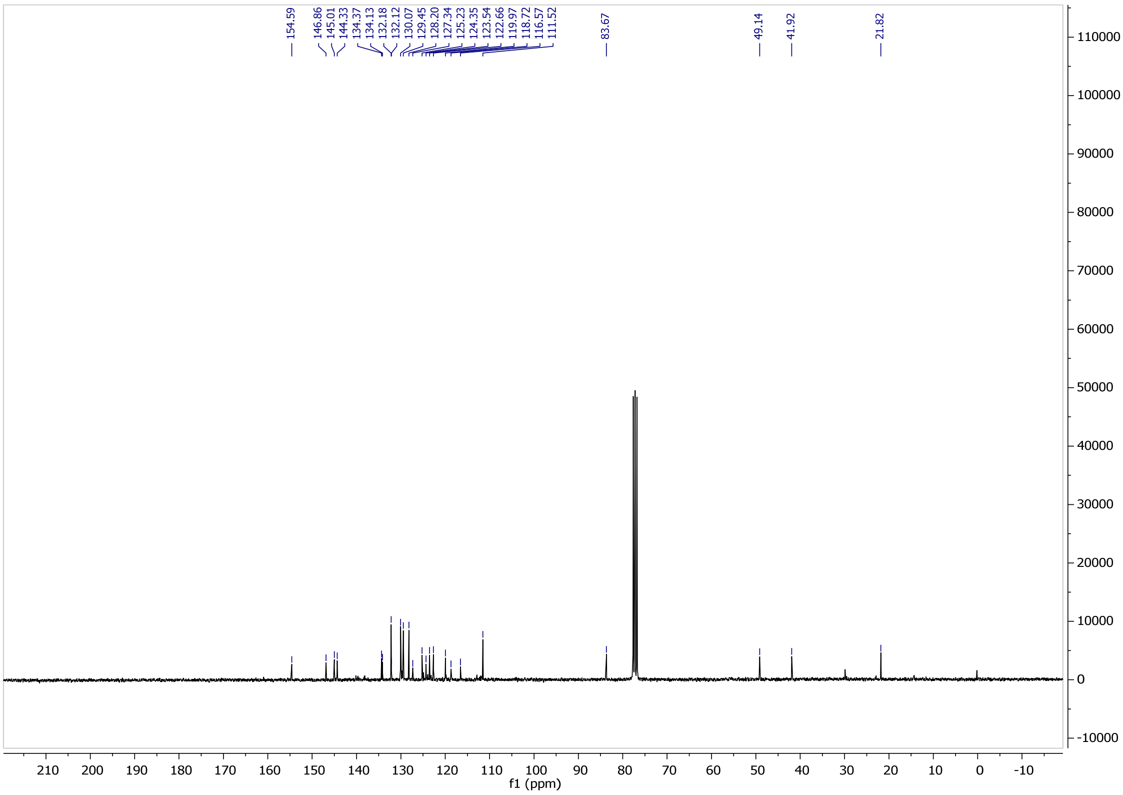

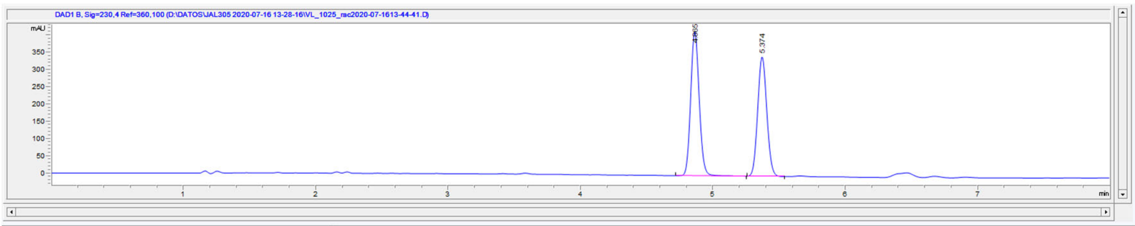

File Information

LC-File	VL_1025_rac2020-07-1613-44-41.D
File Path	D:\DATOS\JAL305 2020-07-16 13-28-16
Date	16-Jul-20, 13:48:00
Sample	VL_1025_rac
Sample Info	VL_1025_rac
Barcode	

#	Time	Type	Area	Height	Width	Area%	Symmetry
1	4.865	BB	1866.3	416.7	0.0703	52.988	0.871
2	5.374	BB	1655.9	345.4	0.0759	47.012	0.899

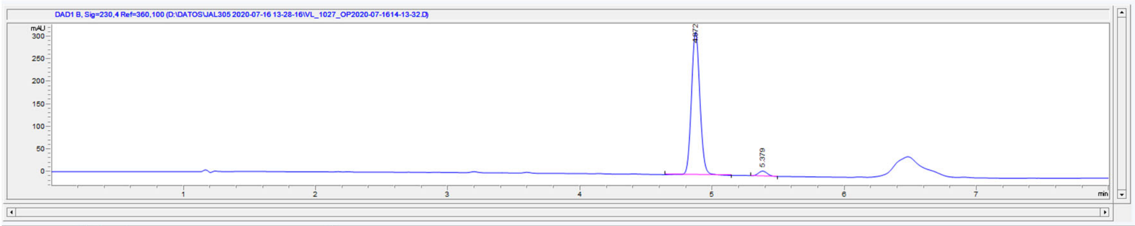

File Information

LC-File	VL_1027_OP2020-07-1614-13-32.D
File Path	D:\DATOS\JAL305 2020-07-16 13-28-16
Date	16-Jul-20, 14:16:57
Sample	VL_1027_OP
Sample Info	VL_1027_OP
Barcode	

#	Time	Type	Area	Height	Width	Area%	Symmetry
1	4.872	BB	1422.5	318.6	0.0681	96.585	0.888
2	5.379	BB	50.3	11	0.0715	3.415	0.87

(*2R,3R,4S*)-4-(3-Chlorophenyl)-1-tosyl-3-vinyl-1,2,3,4-tetrahydrobenzofuro[3,2-*b*]pyridin-2-ol (4g)

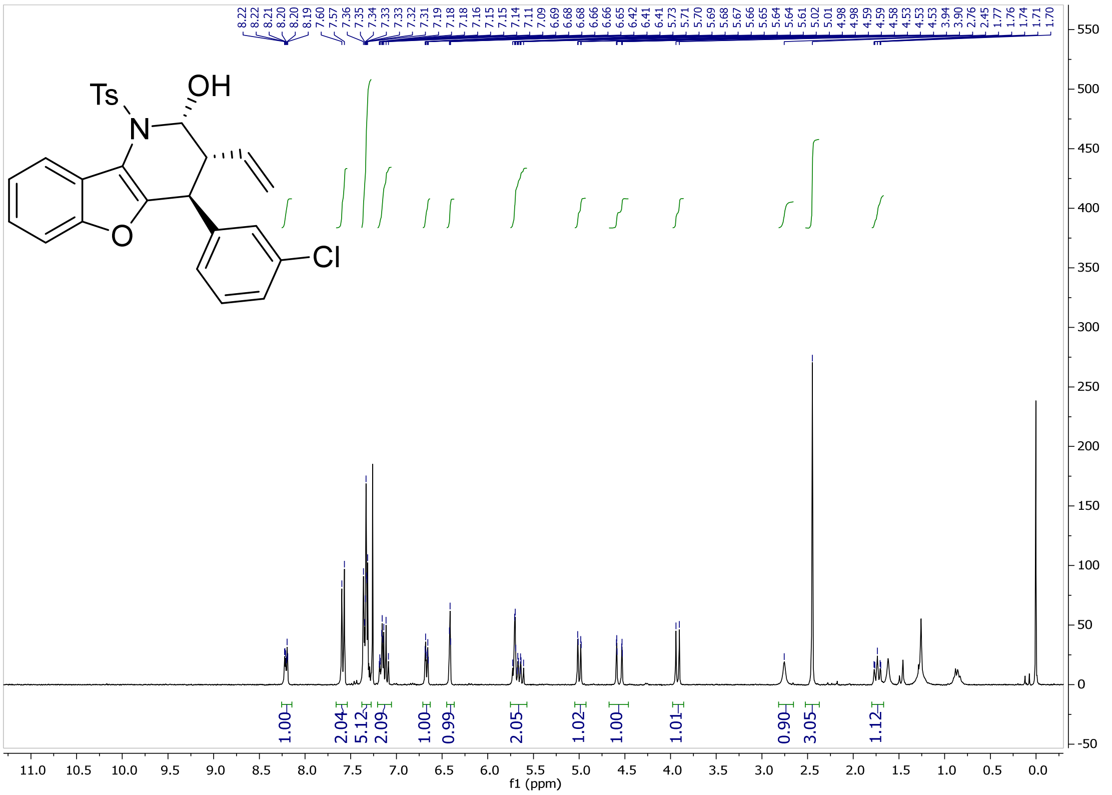

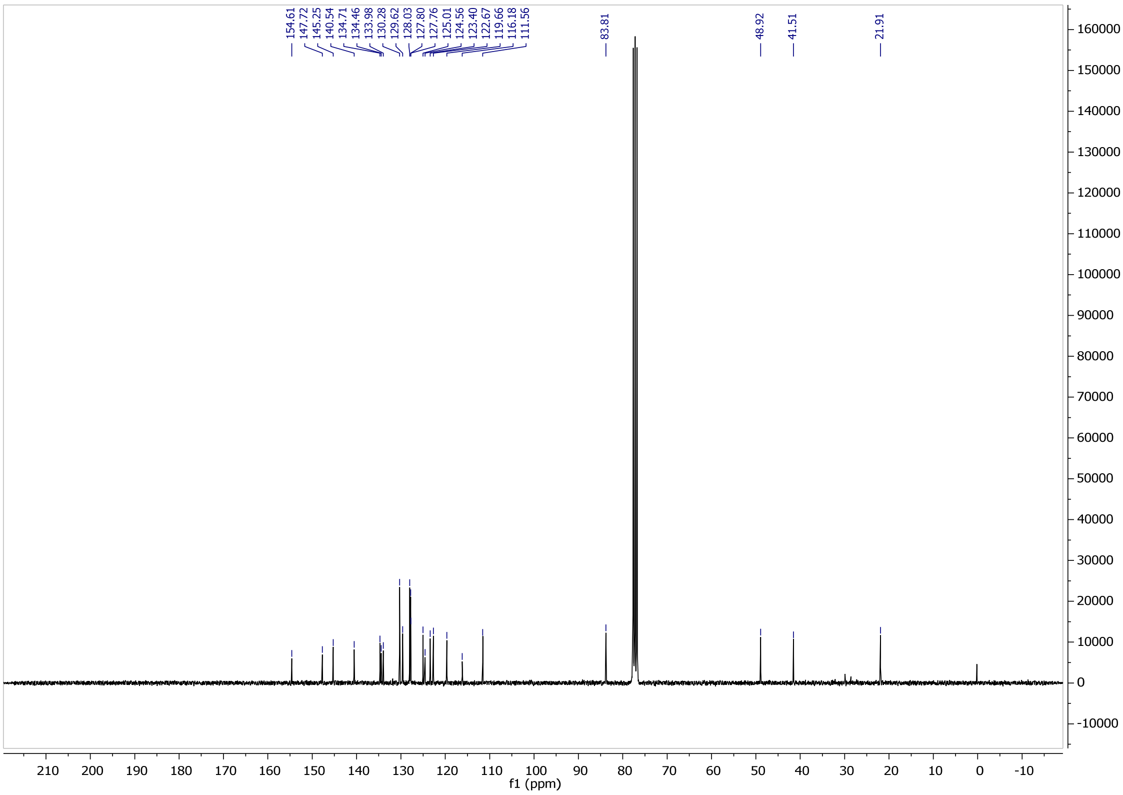

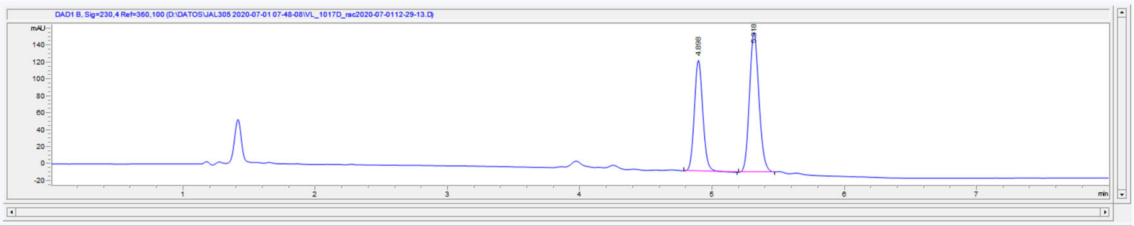

File Information

LC-File	VL_1017D_rac2020-07-0112-29-13.D
File Path	D:\DATOS\JAL305 2020-07-01 07-48-08
Date	01-Jul-20, 12:32:38
Sample	VL_1017D_rac
Sample Info	VL_1017D_rac
Barcode	
Operator	SYSTEM

#	Time	Type	Area	Height	Width	Area%	Symmetry
1	4.898	BB	590.6	131.8	0.0703	41.634	0.858
2	5.318	BB	827.9	165.8	0.0763	58.366	0.846

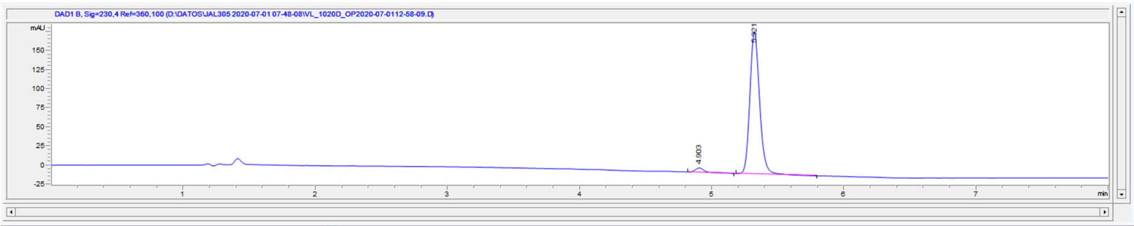

File Information

LC-File	VL_1020D_OP2020-07-0112-58-09.D
File Path	D:\DATOS\JAL305 2020-07-01 07-48-08
Date	01-Jul-20, 13:01:38
Sample	VL_1020D_OP
Sample Info	VL_1020D_OP
Barcode	
Operator	SYSTEM

#	Time	Type	Area	Height	Width	Area%	Symmetry
1	4.903	BB	28.2	5.7	0.0758	2.860	0.661
2	5.321	BB	956.4	187.1	0.0796	97.140	0.806

(*2R,3R,4S*)-4-(2-Fluorophenyl)-1-tosyl-3-vinyl-1,2,3,4-tetrahydrobenzofuro[3,2-*b*]pyridin-2-ol (4h)

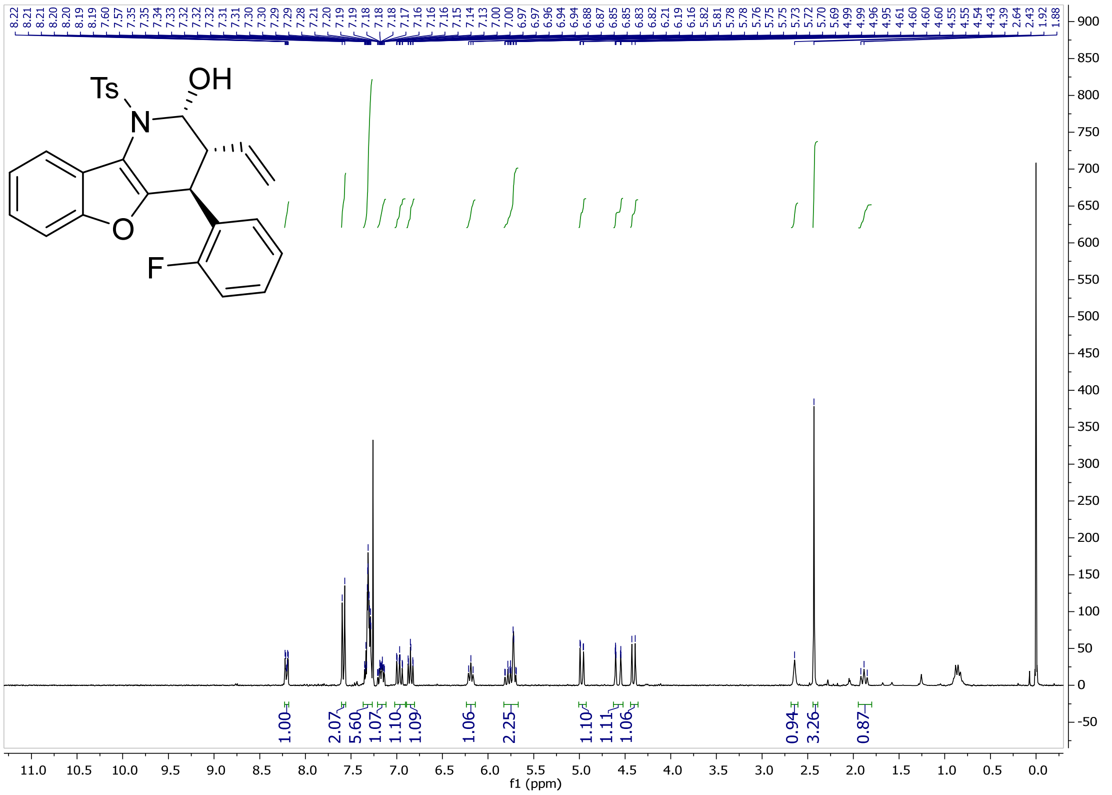

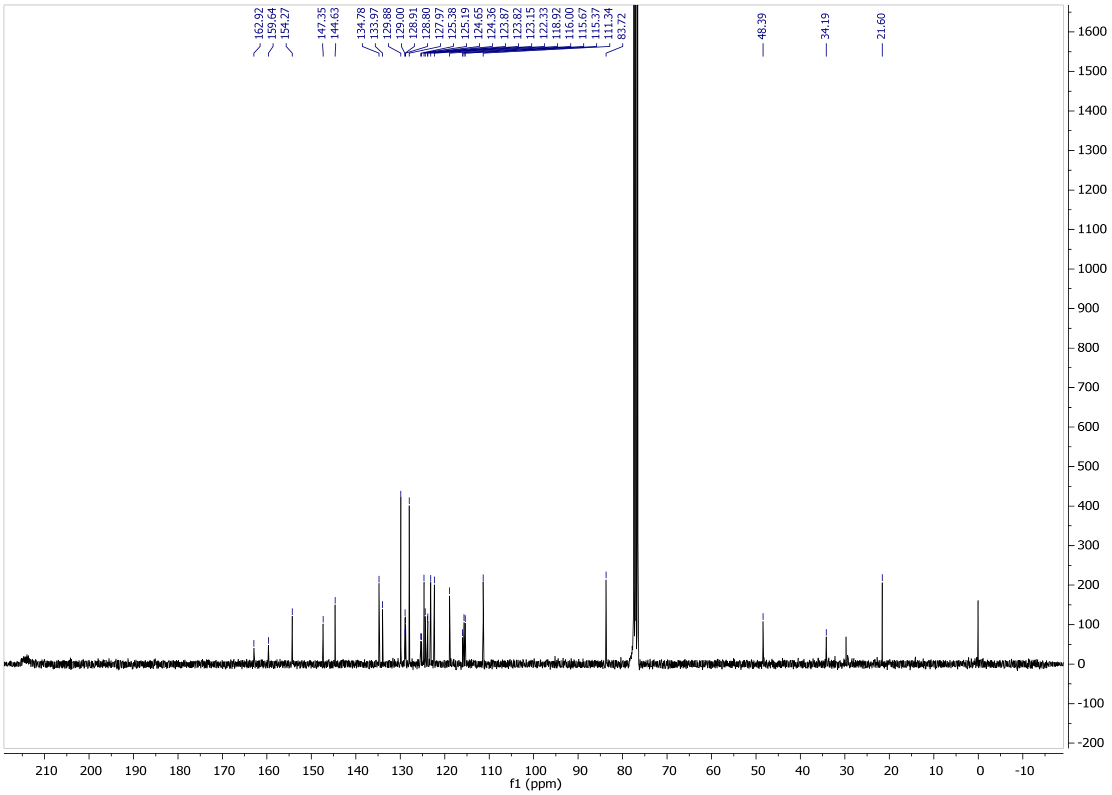

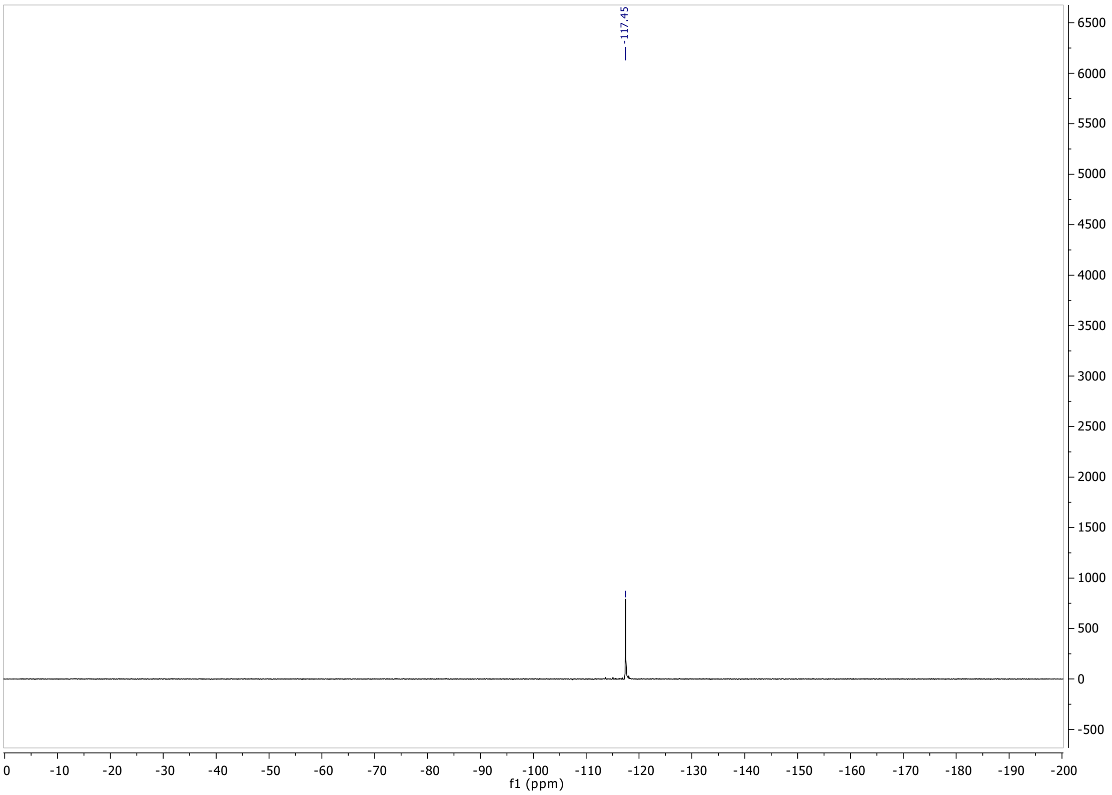

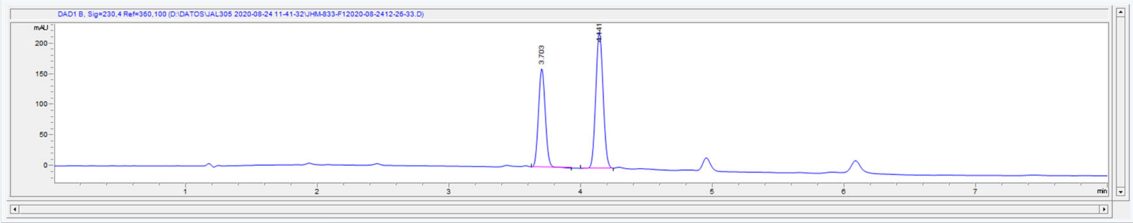

File Information

LC-File	JHM-833-F12020-08-2412-26-33.D
File Path	D:\DATOS\JAL305 2020-08-24 11-41-32
Date	24-Aug-20, 12:30:05
Sample	JHM-833-F1
Sample Info	JHM-833-F1
Barcode	
Operator	SYSTEM
Method	IC-gradiente5_40_MeOH_MS.M
Reference	D:\DATOS\JAL305 2020-08-24 11-41-32\no Sample Name2020-08-[illegible]

#	Time	Type	Area	Height	Width	Area%	Symmetry
1	3.703	BB	583.7	162	0.056	40.382	0.895
2	4.141	BB	861.7	223.4	0.059	59.618	0.922

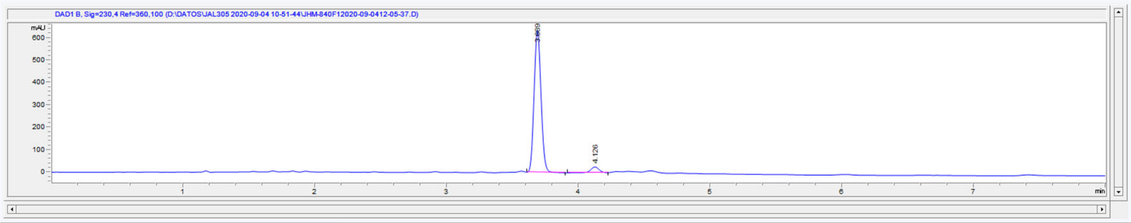

File Information

LC-File	JHM-840F12020-09-0412-05-37.D
File Path	D:\DATOS\JAL305 2020-09-04 10-51-44
Date	04-Sep-20, 12:08:59
Sample	JHM-840F1
Sample Info	JHM-840F1
Barcode	
Operator	SYSTEM
Method	IC-gradiente5_40_MeOH_MS.M
Reference	D:\DATOS\JAL305 2020-09-04 10-51-44\no Sample Name2020-09-[illegible]

#	Time	Type	Area	Height	Width	Area%	Symmetry
1	3.689	BB	2298.5	631.1	0.0565	95.384	0.891
2	4.126	BB	111.2	25.4	0.067	4.616	1.04

(*2R,3R,4S*)-4-(2-Bromophenyl)-1-tosyl-3-vinyl-1,2,3,4-tetrahydrobenzofuro[3,2-*b*]pyridin-2-ol (4i)

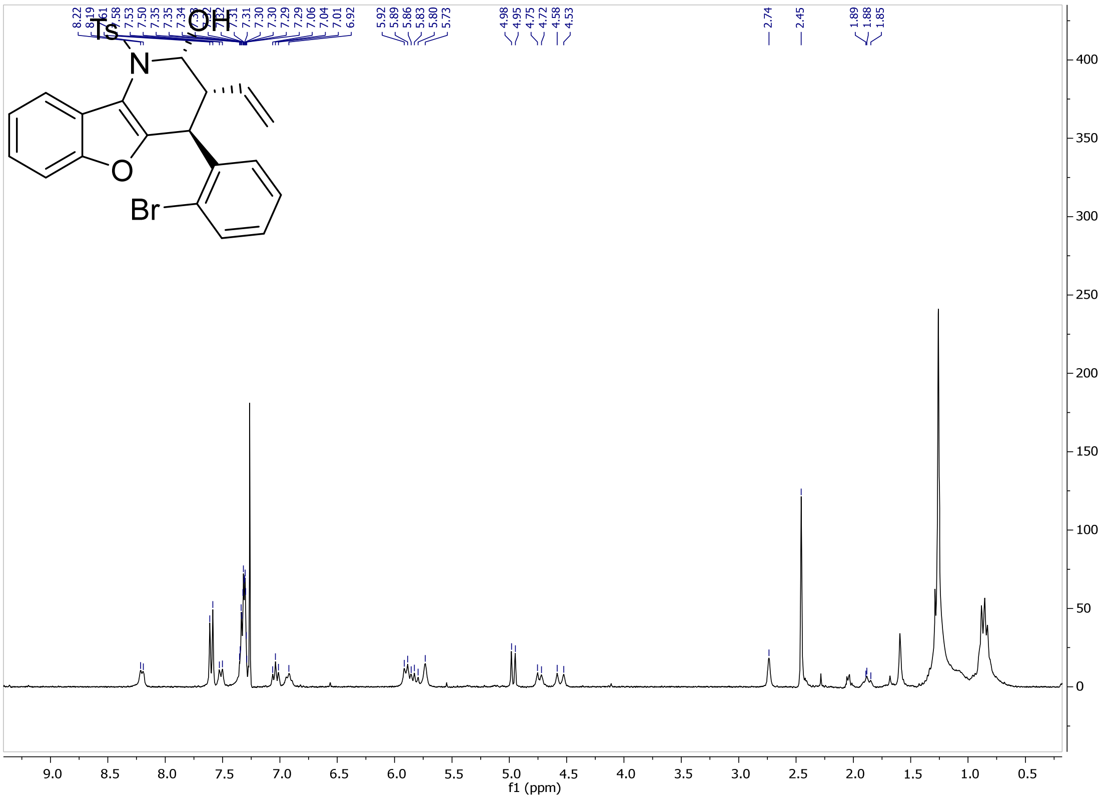

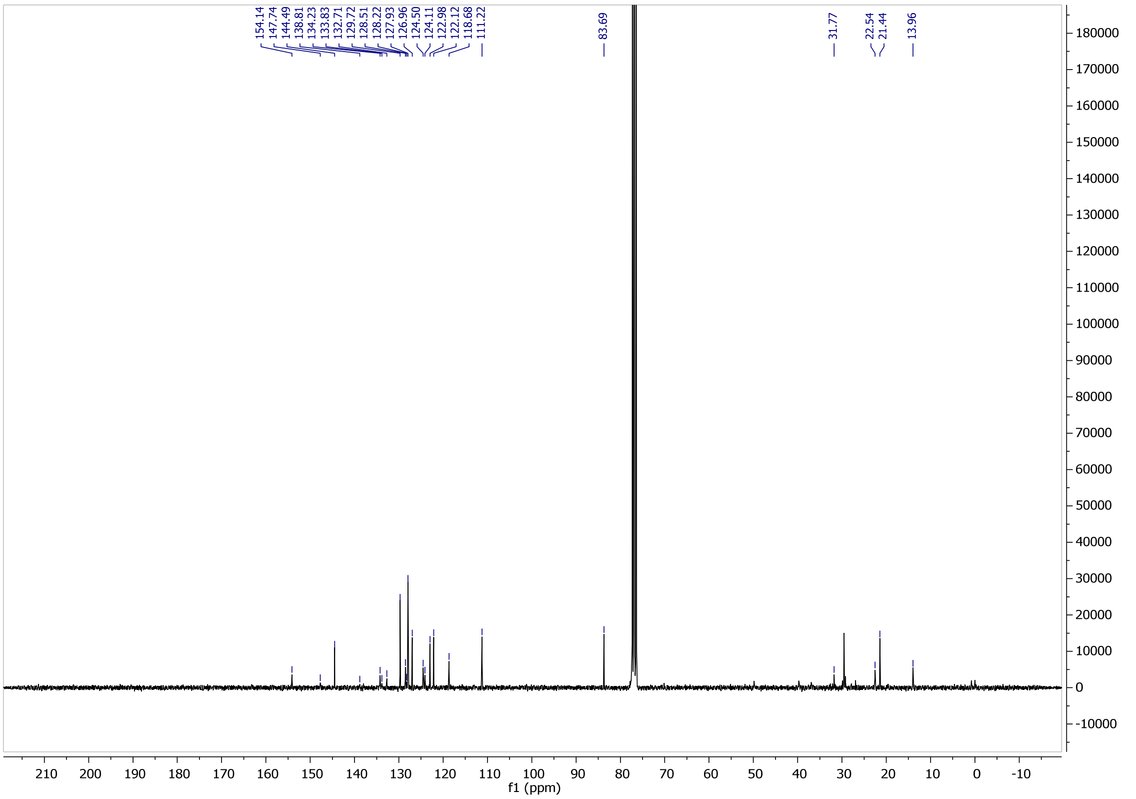

DAD1 A, Sig=210,4 Ref=360,100 (D:\DATOS\JAL305 2021-03-01 10-52-03\VL_1179A2021-03-0115-28-10.D)

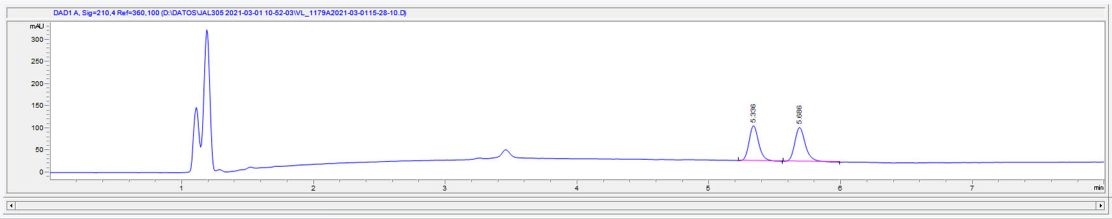

File Information

LC-File	VL_1179A2021-03-0115-28-10.D
File Path	D:\DATOS\JAL305 2021-03-01 10-52-03
Date	01-Mar-21, 15:31:26
Sample	VL_1179A
Sample Info	VL_1179A
Barcode	
Operator	SYSTEM
Method	IA-gradiente5_40_MeOH_MS.M
Reference	D:\DATOS\JAL305 2021-03-01 10-52-03\no Sample Name2021-03-(
Analysis Time	8 min

#	Time	Type	Area	Height	Width	Area%	Symmetry
1	5.336	BB	401.7	79	0.0774	48.028	0.806
2	5.686	BB	434.7	76.7	0.0862	51.972	0.758

DAD1 A, Sig=210,4 Ref=360,100 (D:\DATOS\JAL305 2021-03-01 10-52-03\VL_1179B2021-03-0115-56-58.D)

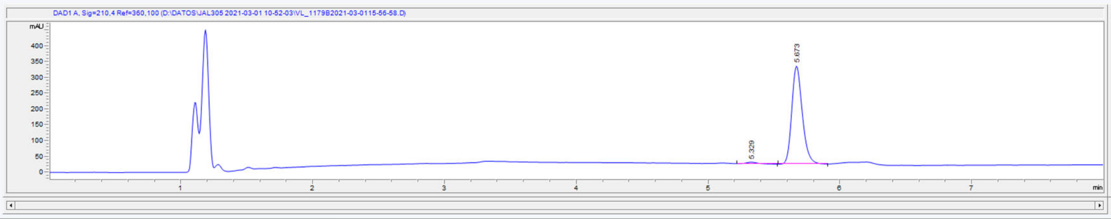

File Information

LC-File	VL_1179B2021-03-0115-56-58.D
File Path	D:\DATOS\JAL305 2021-03-01 10-52-03
Date	01-Mar-21, 16:00:18
Sample	VL_1179B
Sample Info	VL_1179B
Barcode	
Operator	SYSTEM
Method	IA-gradiente5_40_MeOH_MS.M
Reference	D:\DATOS\JAL305 2021-03-01 10-52-03\no Sample Name2021-03-(
Analysis Time	7.993 min

#	Time	Type	Area	Height	Width	Area%	Symmetry
1	5.329	BB	31.5	5.2	0.088	1.795	0.677
2	5.673	BB	1721.7	309.9	0.0848	98.205	0.766

(*2R,3R,4S*)-4-(3-Methoxyphenyl)-1-tosyl-3-vinyl-1,2,3,4-tetrahydrobenzofuro[3,2-*b*]pyridin-2-ol (4j)

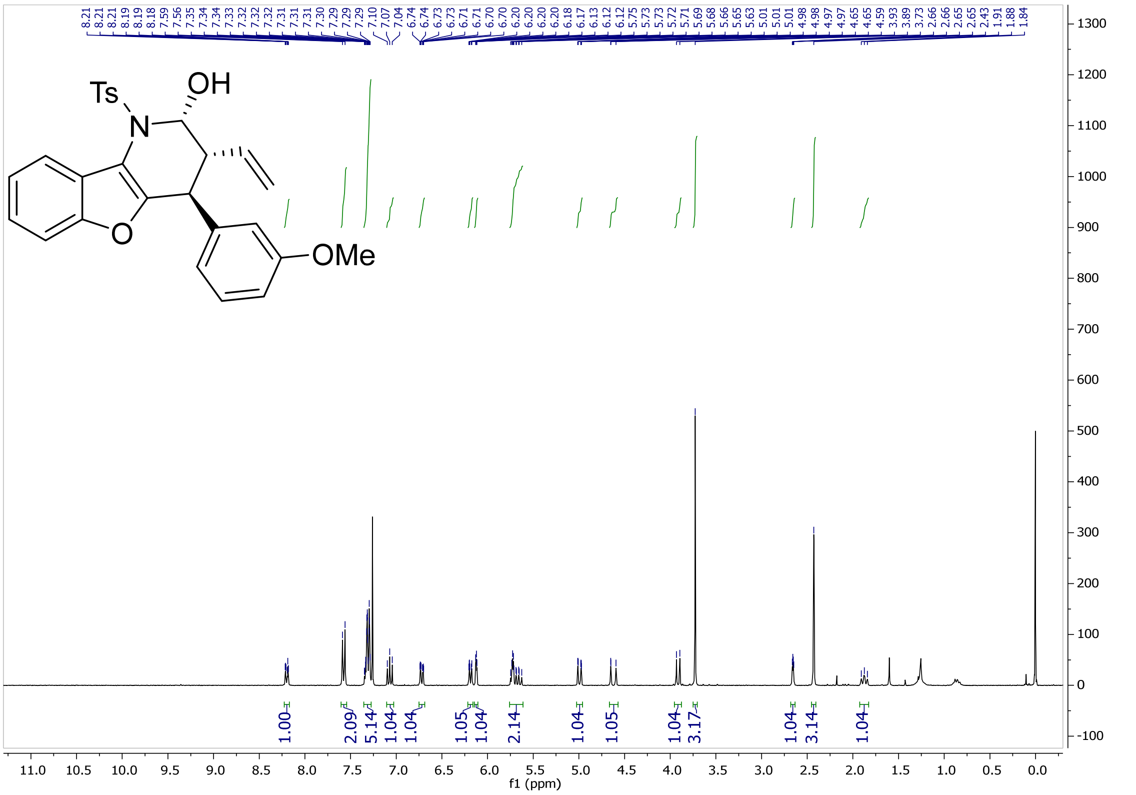

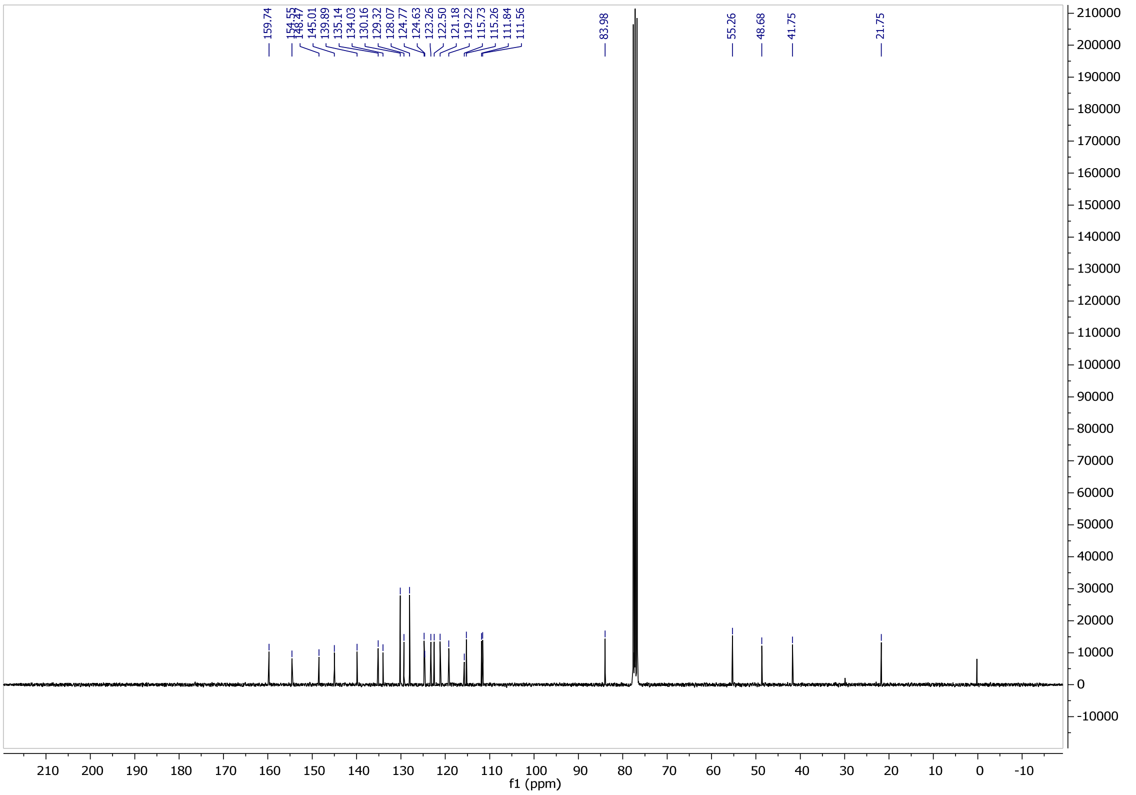

DAD1 B, Sig=230,4 Ref=360,100 (D:\DATOS\JAL305 2020-07-16 13-28-16\VL_1026_rac2020-07-1614-42-27.D)

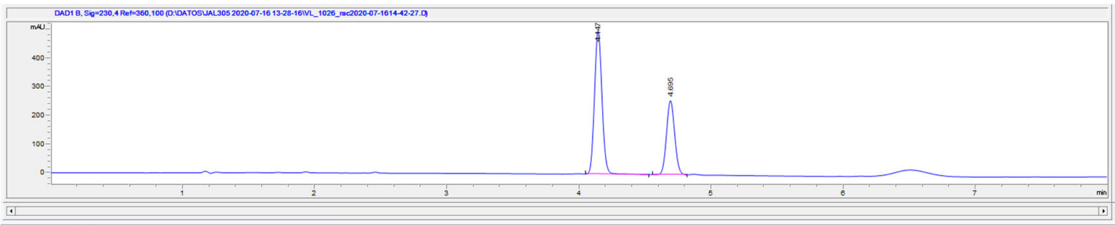

File Information

LC-File	VL_1026_rac2020-07-1614-42-27.D
File Path	D:\DATOS\JAL305 2020-07-16 13-28-16
Date	16-Jul-20, 14:45:53
Sample	VL_1026_rac
Sample Info	VL_1026_rac
Barcode	

#	Time	Type	Area	Height	Width	Area%	Symmetry
1	4.147	BB	1973	500.7	0.06	63.993	0.878
2	4.695	BB	1110.2	259.3	0.0659	36.007	0.908

DAD1 B, Sig=230,4 Ref=360,100 (D:\DATOS\JAL305 2020-07-16 13-28-16\VL_1028_OP2020-07-1615-11-23.D)

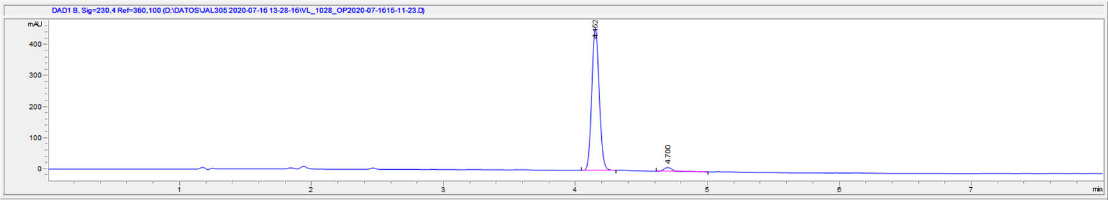

File Information

LC-File	VL_1028_OP2020-07-1615-11-23.D
File Path	D:\DATOS\JAL305 2020-07-16 13-28-16
Date	16-Jul-20, 15:14:52
Sample	VL_1028_OP
Sample Info	VL_1028_OP
Barcode	

#	Time	Type	Area	Height	Width	Area%	Symmetry
1	4.152	BB	1794.4	459.1	0.0616	96.819	0.899
2	4.7	BB	59	12.3	0.0737	3.181	0.68

(*2R,3R,4S*)-4-(Naphthalen-2-yl)-1-tosyl-3-vinyl-1,2,3,4-tetrahydrobenzofuro[3,2-*b*]pyridin-2-ol (4k)

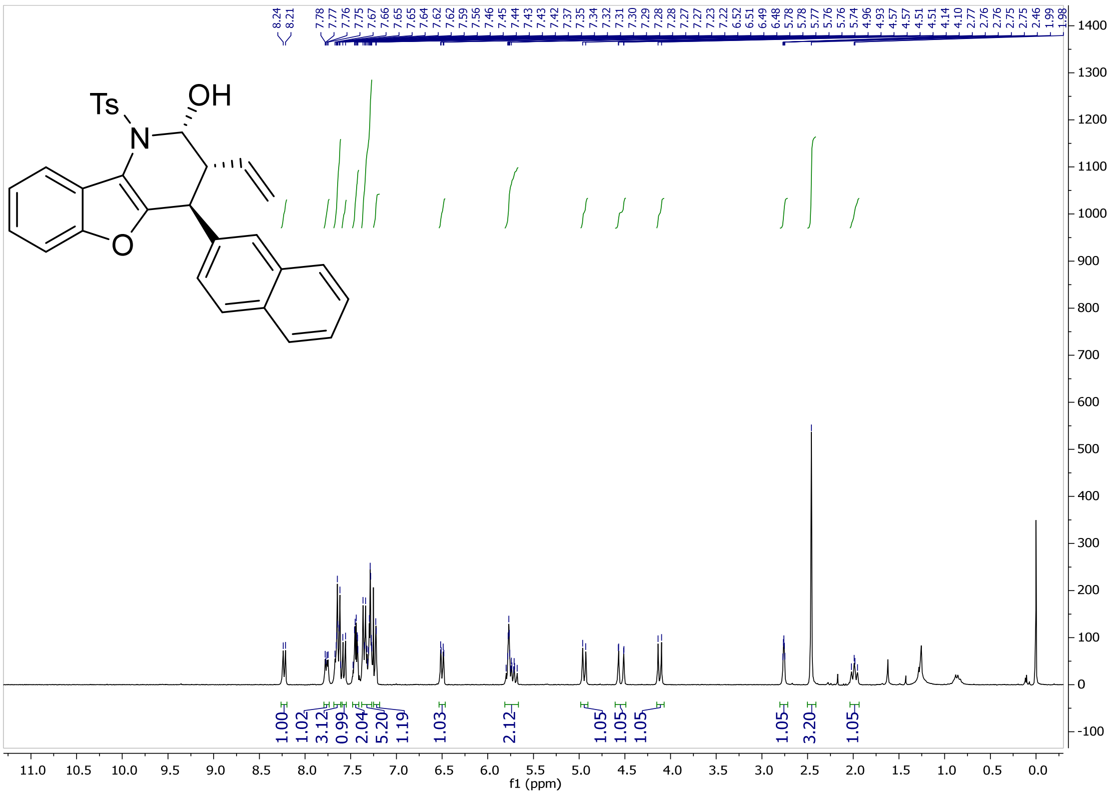

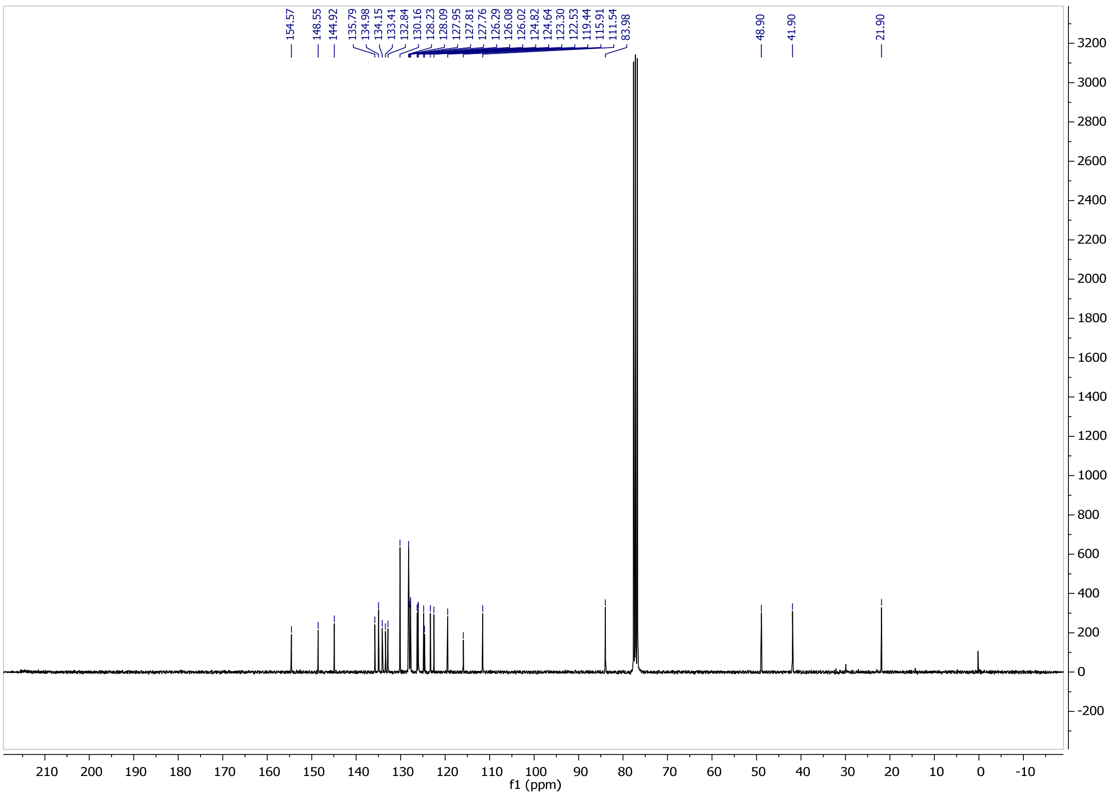

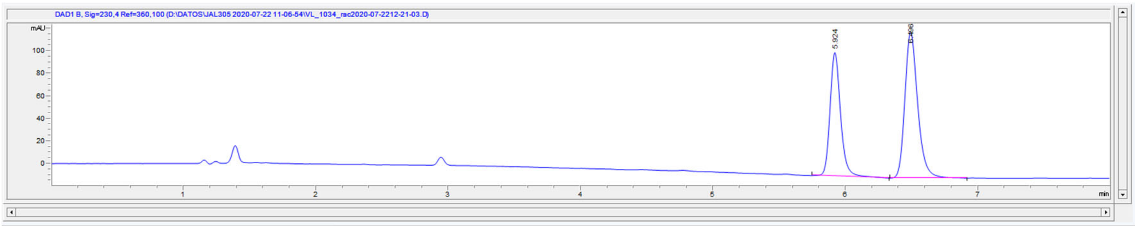

File Information

LC-File	VL_1034_rac2020-07-2212-21-03.D
File Path	D:\DATOS\JAL305 2020-07-22 11-06-54
Date	22-Jul-20, 12:24:28
Sample	VL_1034_rac
Sample Info	VL_1034_rac
Barcode	
Operator	SYSTEM
Method	IA-gradiente5_40_MeOH_MS.M
Reference	D:\DATOS\JAL305 2020-07-22 11-06-54\no Sample Name2020-07-:

#	Time	Type	Area	Height	Width	Area%	Symmetry
1	5.924	BB	616.8	110.3	0.0852	41.271	0.786
2	6.496	BB	877.7	129.9	0.1028	58.729	0.752

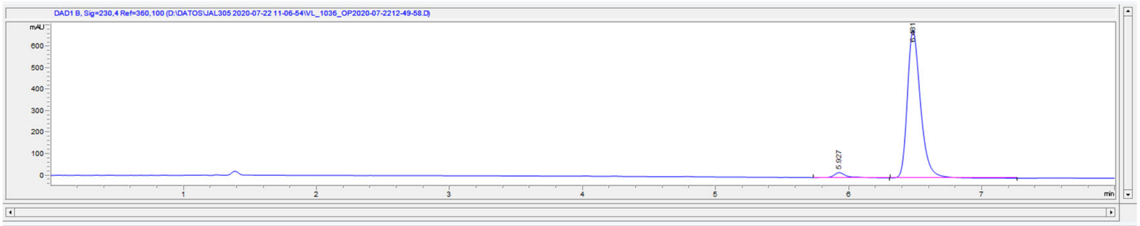

File Information

LC-File	VL_1036_OP2020-07-2212-49-58.D
File Path	D:\DATOS\JAL305 2020-07-22 11-06-54
Date	22-Jul-20, 12:53:24
Sample	VL_1036_OP
Sample Info	VL_1036_OP
Barcode	
Operator	SYSTEM
Method	IA-gradiente5_40_MeOH_MS.M
Reference	D:\DATOS\JAL305 2020-07-22 11-06-54\no Sample Name2020-07-:

#	Time	Type	Area	Height	Width	Area%	Symmetry
1	5.927	BB	152.6	23.6	0.0953	3.143	0.643
2	6.481	BB	4702.1	685.7	0.106	96.857	0.689

(*2R,3R,4S*)-8-Bromo-4-phenyl-1-tosyl-3-vinyl-1,2,3,4-tetrahydrobenzofuro[3,2-*b*]pyridin-2-ol (4l)

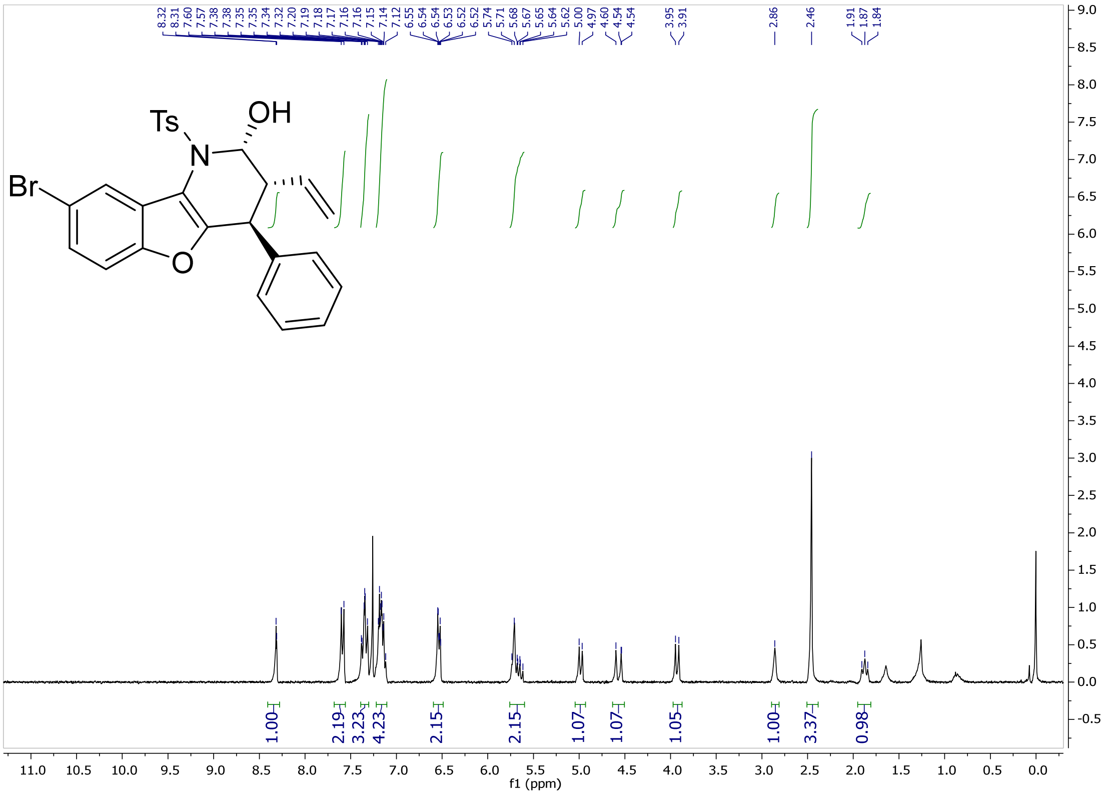

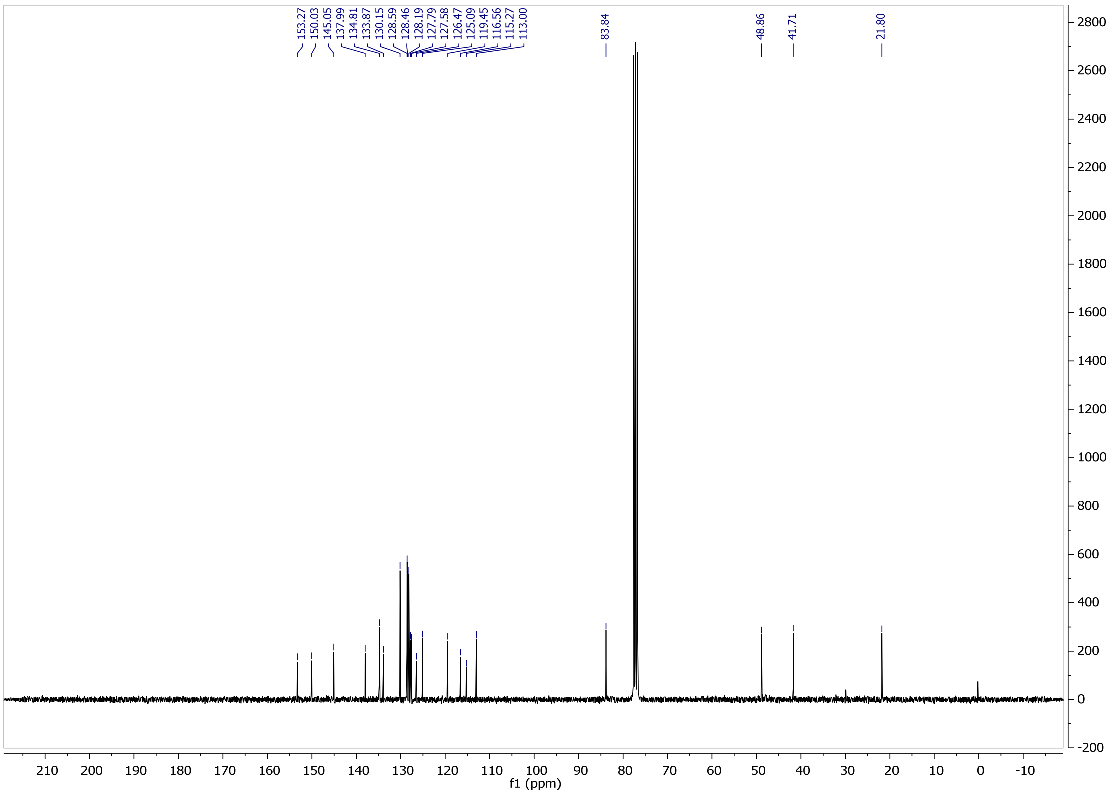

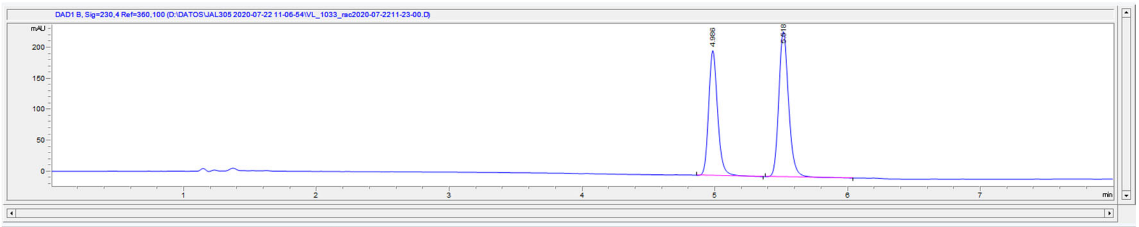

File Information

LC-File	VL_1033_rac2020-07-2211-23-00.D
File Path	D:\DATOS\JAL305 2020-07-22 11-06-54
Date	22-Jul-20, 11:26:33
Sample	VL_1033_rac
Sample Info	VL_1033_rac
Barcode	
Operator	SYSTEM
Method	IA-gradiente5_40_MeOH_MS.M
Reference	D:\DATOS\JAL305 2020-07-22 11-06-54\no Sample Name2020-07-2
Analysis Time	7.993 min

#	Time	Type	Area	Height	Width	Area%	Symmetry
1	4.986	BB	948.7	201.4	0.0729	44.011	0.791
2	5.518	BB	1207	234.2	0.0801	55.989	0.795

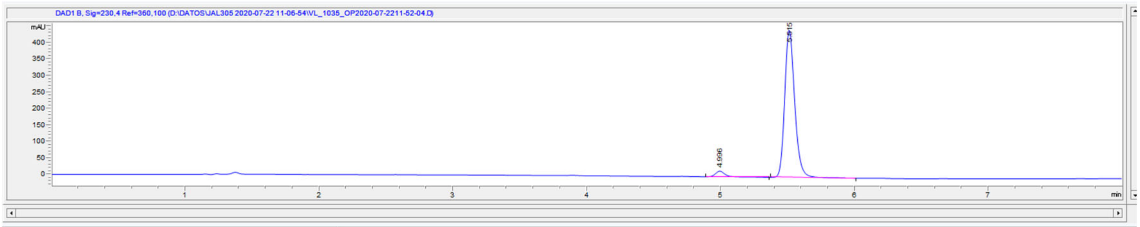

File Information

LC-File	VL_1035_OP2020-07-2211-52-04.D
File Path	D:\DATOS\JAL305 2020-07-22 11-06-54
Date	22-Jul-20, 11:55:32
Sample	VL_1035_OP
Sample Info	VL_1035_OP
Barcode	
Operator	SYSTEM
Method	IA-gradiente5_40_MeOH_MS.M
Reference	D:\DATOS\JAL305 2020-07-22 11-06-54\no Sample Name2020-07-2

#	Time	Type	Area	Height	Width	Area%	Symmetry
1	4.996	BB	92.3	17.5	0.0774	3.823	0.682
2	5.515	BB	2322.3	447.4	0.0806	96.177	0.765

(*2R,3R,4S*)-7-Bromo-4-phenyl-1-tosyl-3-vinyl-1,2,3,4-tetrahydrobenzofuro[3,2-*b*]pyridin-2-ol (4m)

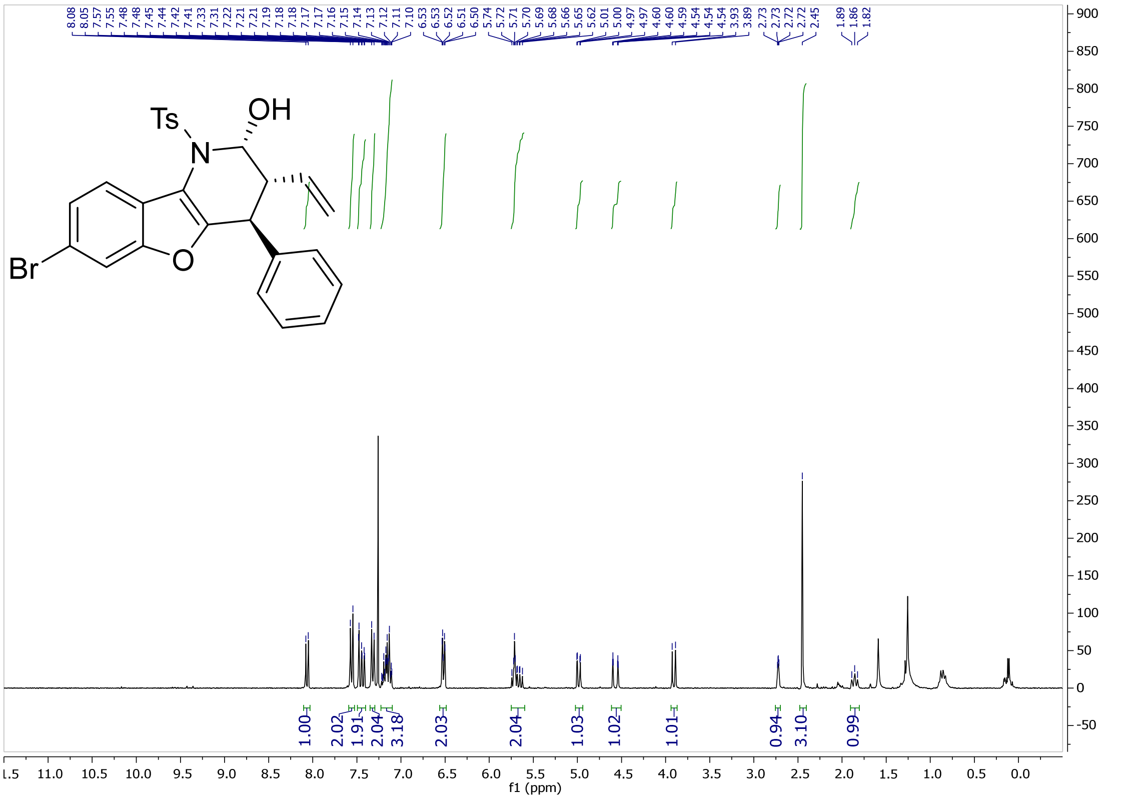

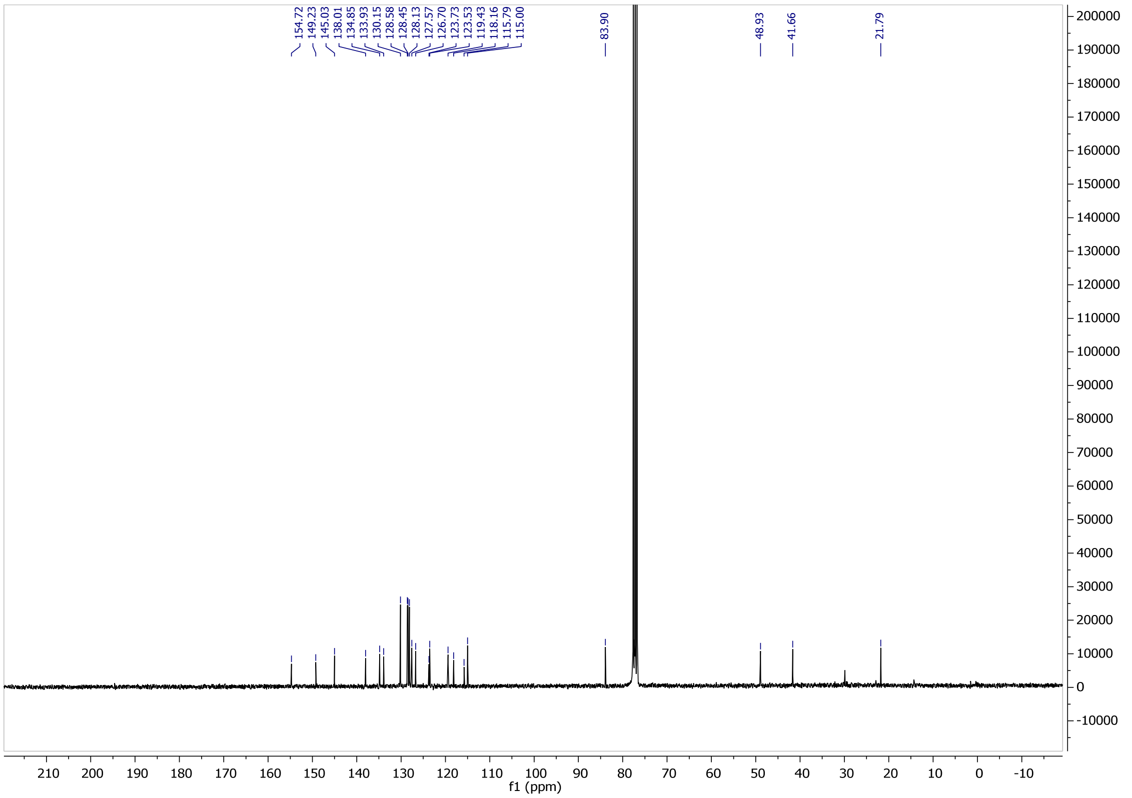

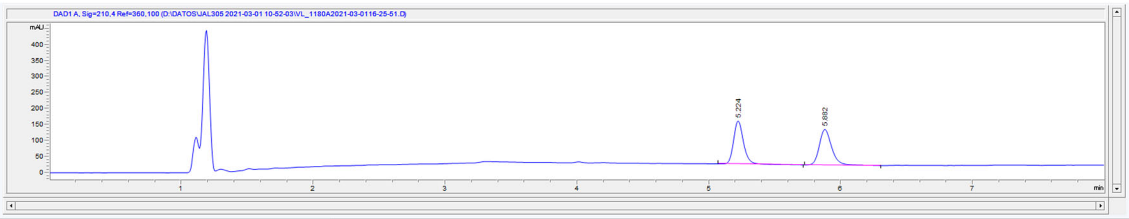

File Information

LC-File	VL_1180A2021-03-0116-25-51.D
File Path	D:\DATOS\JAL305 2021-03-01 10-52-03
Date	01-Mar-21, 16:29:17
Sample	VL_1180A
Sample Info	VL_1180A
Barcode	
Operator	SYSTEM
Method	IA-gradiente5_40_MeOH_MS.M
Reference	D:\DATOS\JAL305 2021-03-01 10-52-03\no Sample Name2021-03-(
Analysis Time	7.993 min

#	Time	Type	Area	Height	Width	Area%	Symmetry
1	5.224	BB	707.5	134.5	0.0794	49.468	0.778
2	5.882	BB	722.7	110.9	0.1001	50.532	0.756

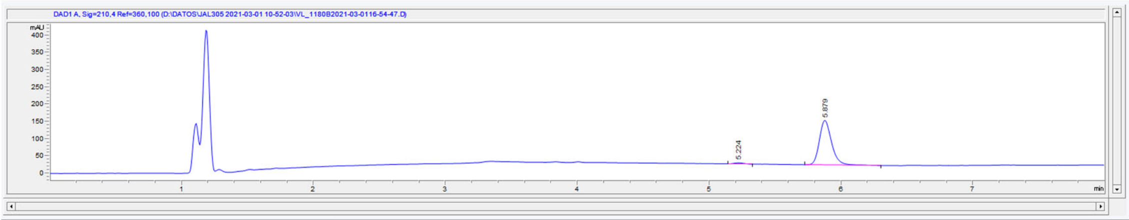

File Information

LC-File	VL_1180B2021-03-0116-54-47.D
File Path	D:\DATOS\JAL305 2021-03-01 10-52-03
Date	01-Mar-21, 16:58:15
Sample	VL_1180B
Sample Info	VL_1180B
Barcode	
Operator	SYSTEM
Method	IA-gradiente5_40_MeOH_MS.M
Reference	D:\DATOS\JAL305 2021-03-01 10-52-03\no Sample Name2021-03-(
Analysis Time	7.993 min

#	Time	Type	Area	Height	Width	Area%	Symmetry
1	5.224	BB	16.6	3.2	0.082	1.951	0.808
2	5.879	BB	833	128.4	0.0997	98.049	0.745

(*2R,3R,4S*)-3-(2-Methylprop-1-en-1-yl)-4-phenyl-1-tosyl-1,2,3,4-tetrahydrobenzofuro[3,2-*b*] pyridin-2-ol (4n)

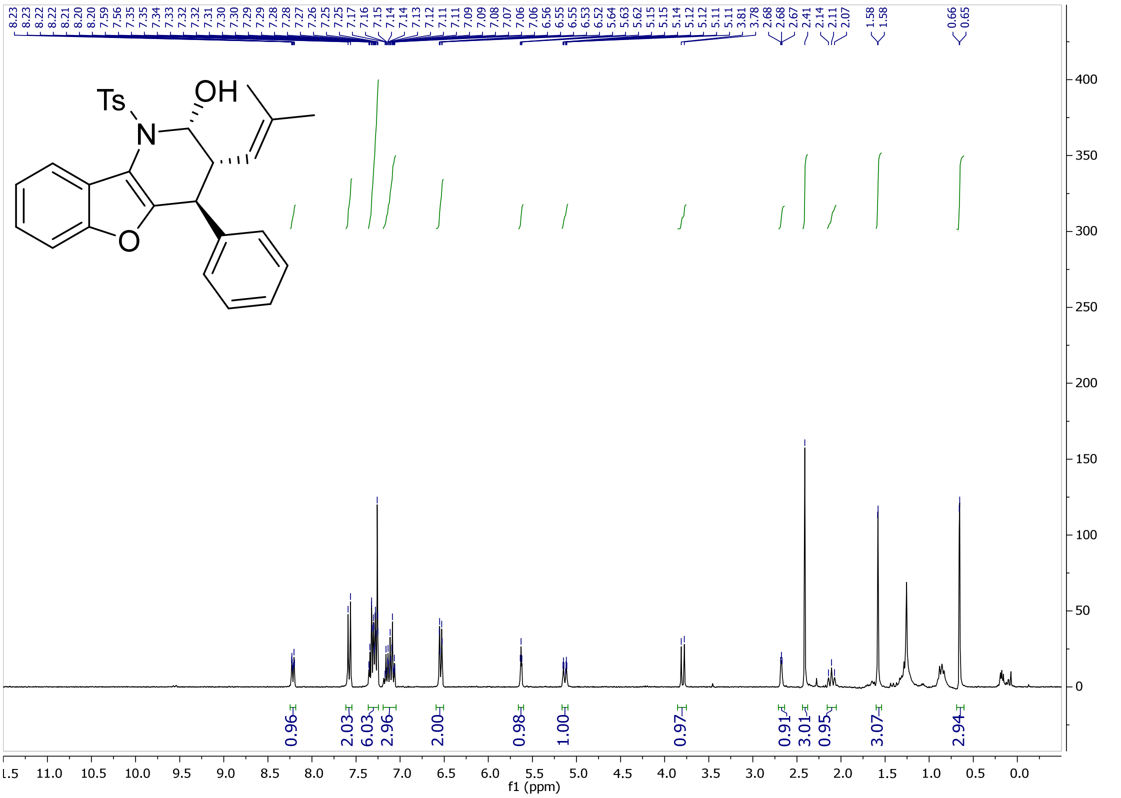

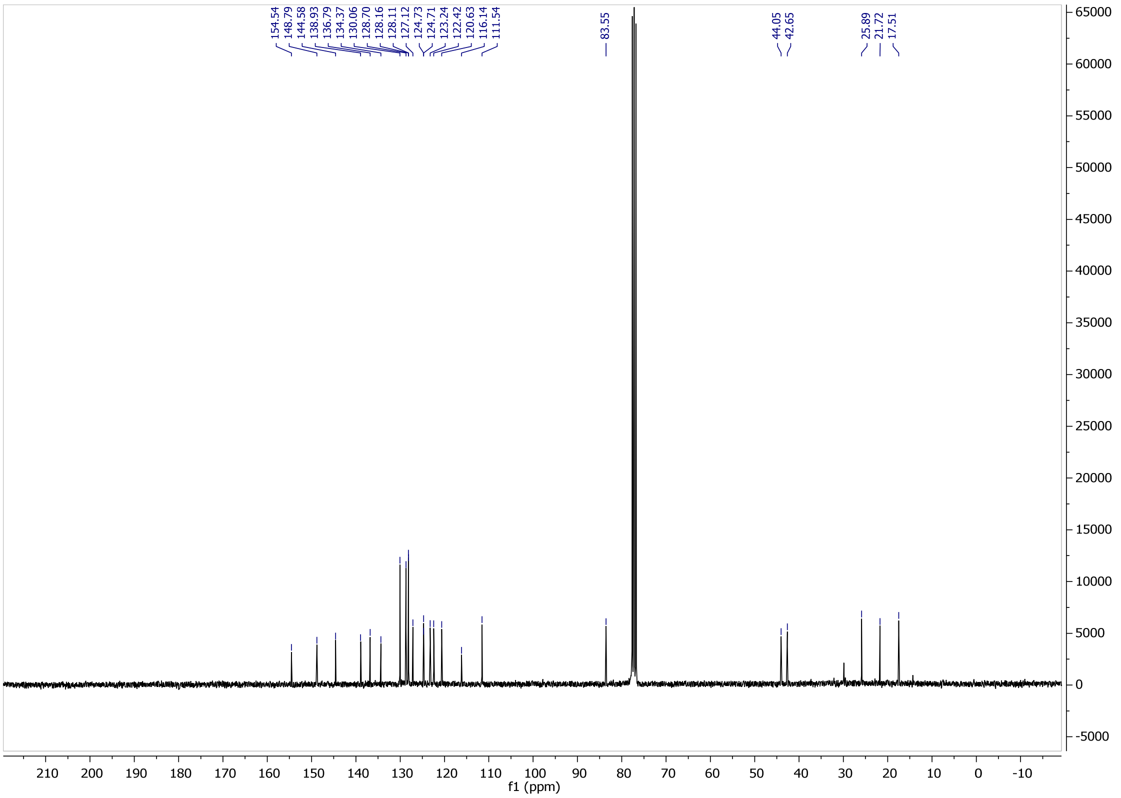

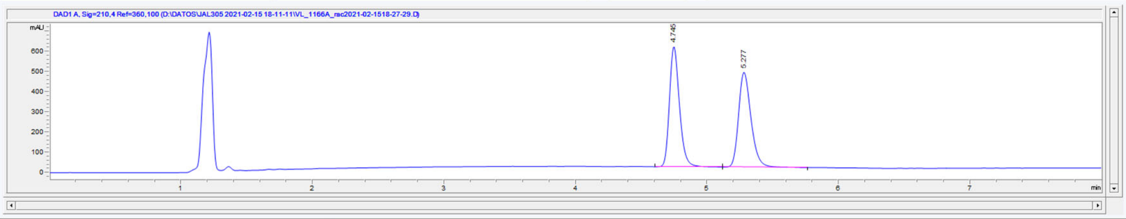

File Information

LC-File	VL_1166A_rac2021-02-1518-27-29.D
File Path	D:\DATOS\JAL305 2021-02-15 18-11-11
Date	15-Feb-21, 18:30:58
Sample	VL_1166A_rac
Sample Info	VL_1166A_rac
Barcode	
Operator	SYSTEM
Method	IA-gradiente5_40_MeOH_MS.M
Reference	D:\DATOS\JAL305 2021-02-15 18-11-11\no Sample Name2021-02-[illegible]
Analysis Time	8 min

#	Time	Type	Area	Height	Width	Area%	Symmetry
1	4.745	BB	3171.7	599.9	0.0817	50.444	0.745
2	5.277	BB	3115.9	473.3	0.1008	49.556	0.717

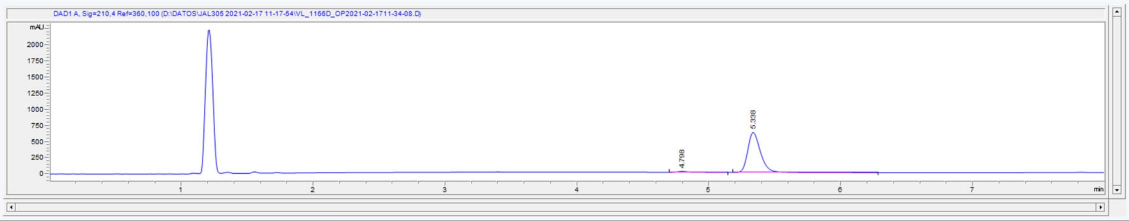

File Information

LC-File	VL_1166D_OP2021-02-1711-34-08.D
File Path	D:\DATOS\JAL305 2021-02-17 11-17-54
Date	17-Feb-21, 11:37:36
Sample	VL_1166D_OP
Sample Info	VL_1166D_OP
Barcode	
Operator	SYSTEM
Method	IA-gradiente5_40_MeOH_MS.M
Reference	D:\DATOS\JAL305 2021-02-17 11-17-54\no Sample Name2021-02-[illegible]
Analysis Time	7.993 min

#	Time	Type	Area	Height	Width	Area%	Symmetry
1	4.798	BB	80.8	12.8	0.0955	1.905	0.557
2	5.338	BB	4161.6	617.5	0.1046	98.095	0.683

(2*R*,3*S*,4*S*)-4-Phenyl-3-(prop-1-en-2-yl)-1-tosyl-1,2,3,4-tetrahydrobenzofuro[3,2-*b*]pyridin-2-ol (4o)

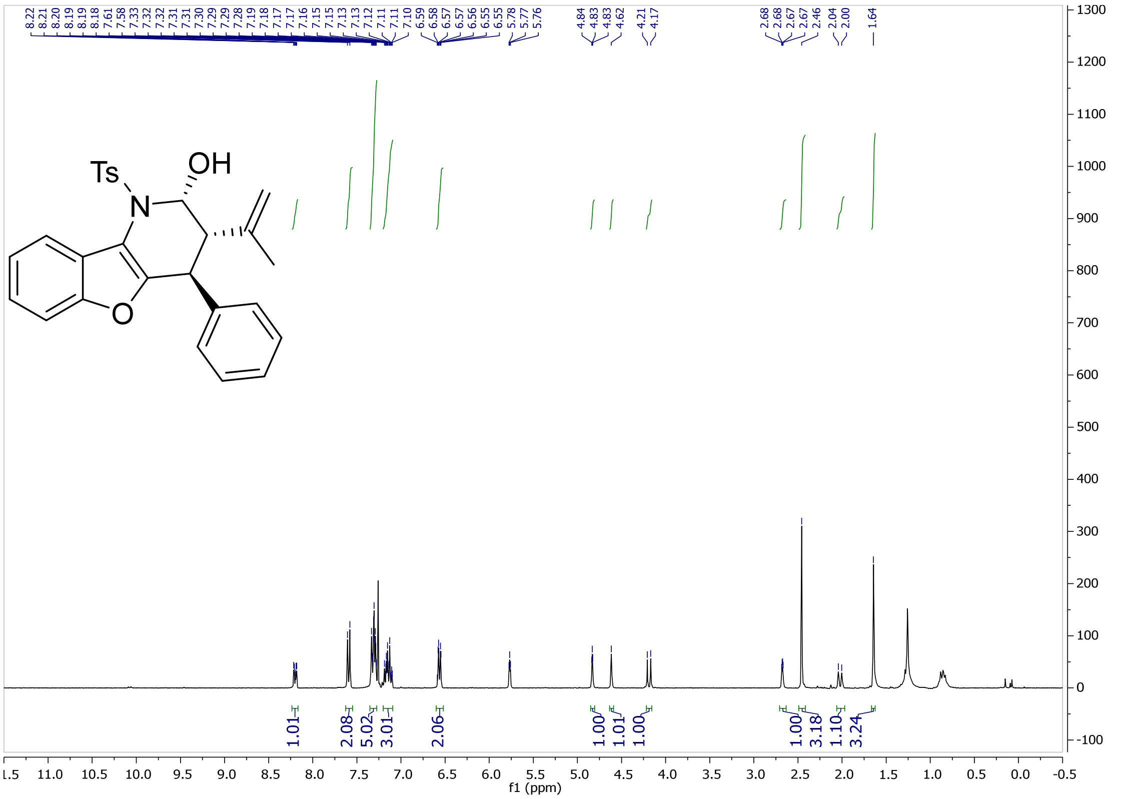

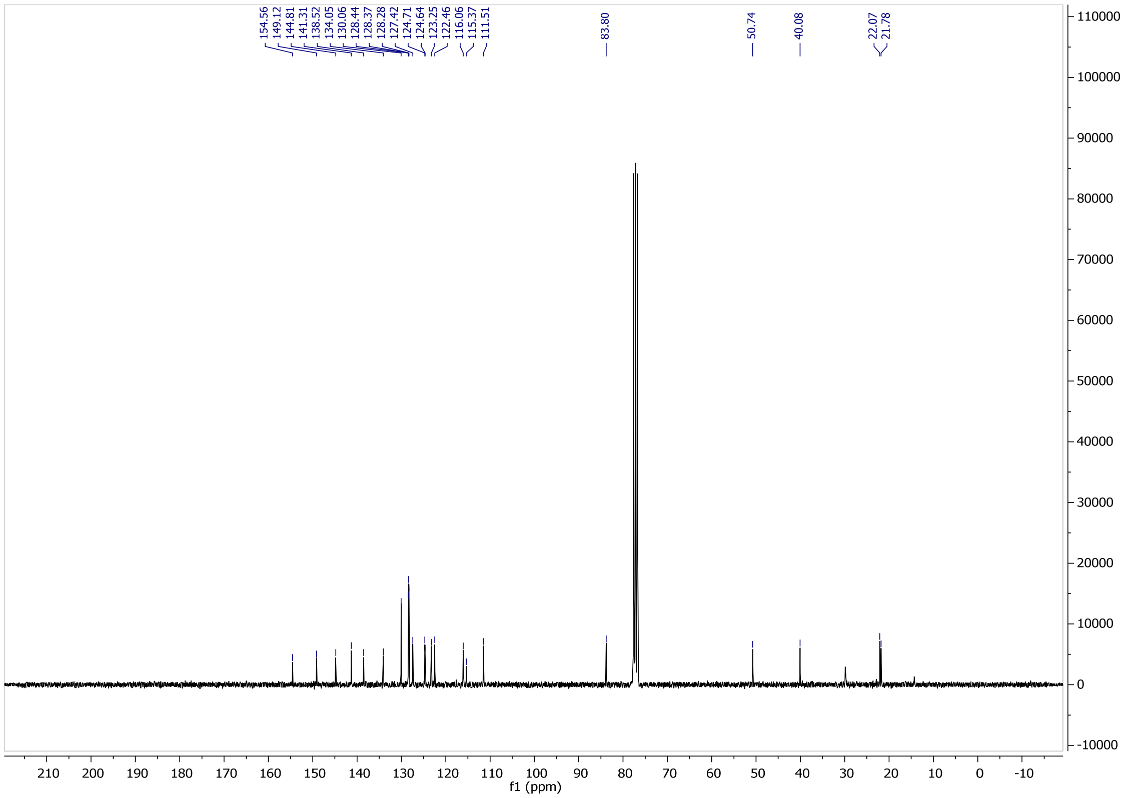

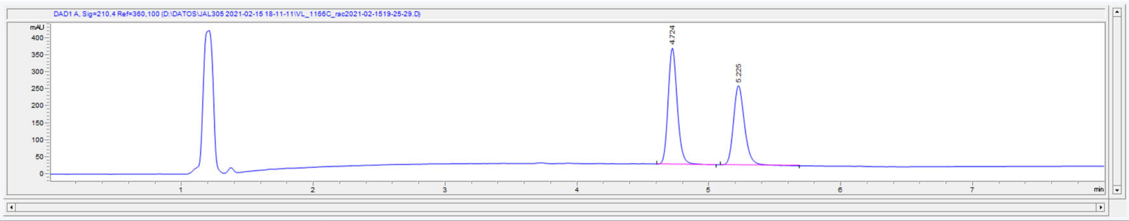

File Information

LC-File	VL_1166C_rac2021-02-1519-25-29.D
File Path	D:\DATOS\JAL305 2021-02-15 18-11-11
Date	15-Feb-21, 19:29:00
Sample	VL_1166C_rac
Sample Info	VL_1166C_rac
Barcode	
Operator	SYSTEM
Method	IA-gradiente5_40_MeOH_MS.M
Reference	D:\DATOS\JAL305 2021-02-15 18-11-11\no Sample Name2021-02-:
Analysis Time	7.993 min

#	Time	Type	Area	Height	Width	Area%	Symmetry
1	4.724	BB	1642.6	341.1	0.0742	54.861	0.802
2	5.225	BB	1351.6	233	0.0897	45.139	0.767

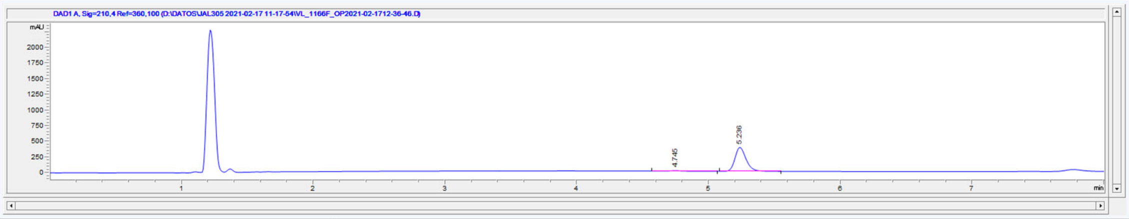

File Information

LC-File	VL_1166F_OP2021-02-1712-36-46.D
File Path	D:\DATOS\JAL305 2021-02-17 11-17-54
Date	17-Feb-21, 12:40:18
Sample	VL_1166F_OP
Sample Info	VL_1166F_OP
Barcode	
Operator	SYSTEM
Method	IA-gradiente5_40_MeOH_MS.M
Reference	D:\DATOS\JAL305 2021-02-17 11-17-54\no Sample Name2021-02-:
Analysis Time	8 min

#	Time	Type	Area	Height	Width	Area%	Symmetry
1	4.745	BB	50.9	8.3	0.0895	2.261	0.722
2	5.236	BB	2201.7	379.1	0.0898	97.739	0.744

(*2R,3S,4S*)-3,4-Diphenyl-1-tosyl-1,2,3,4-tetrahydrobenzofuro[3,2-*b*]pyridin-2-ol (5a)

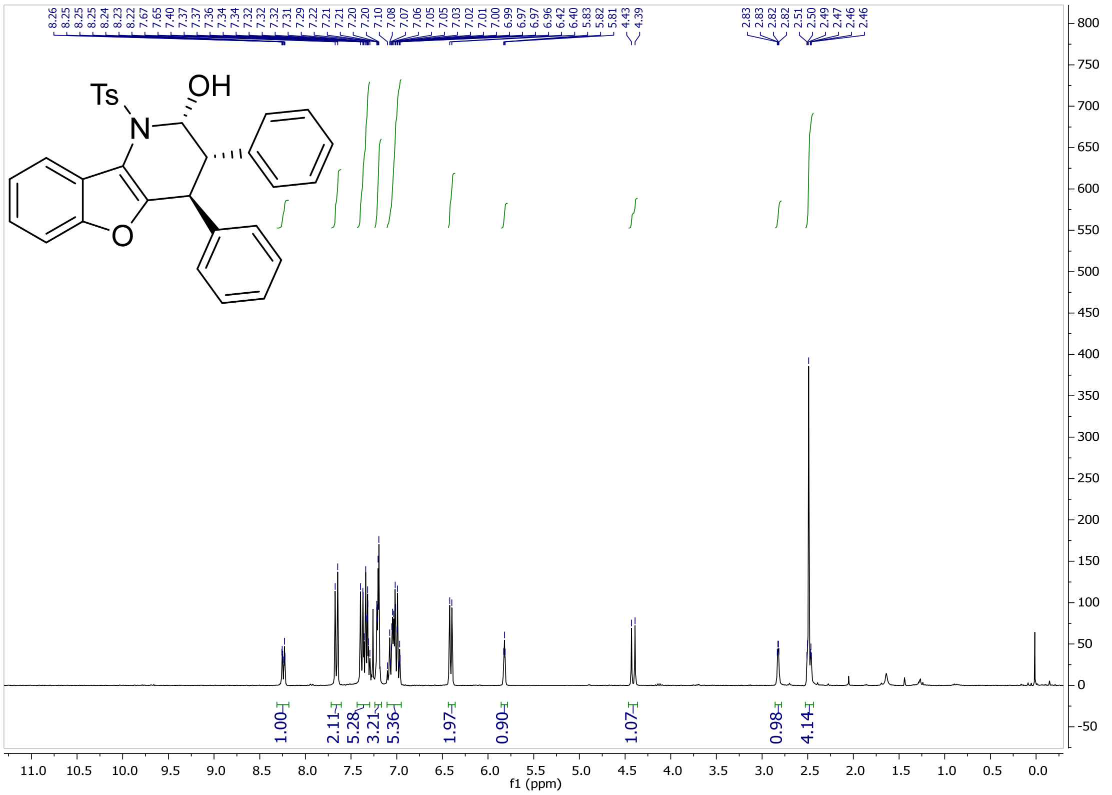

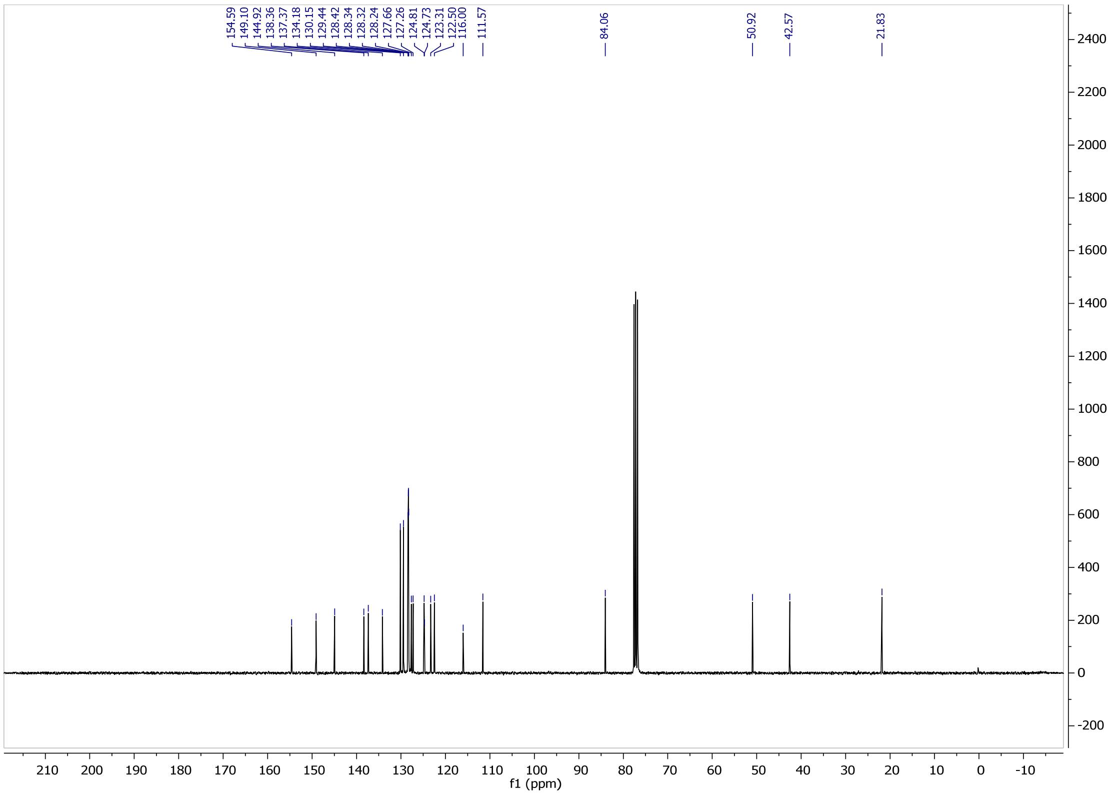

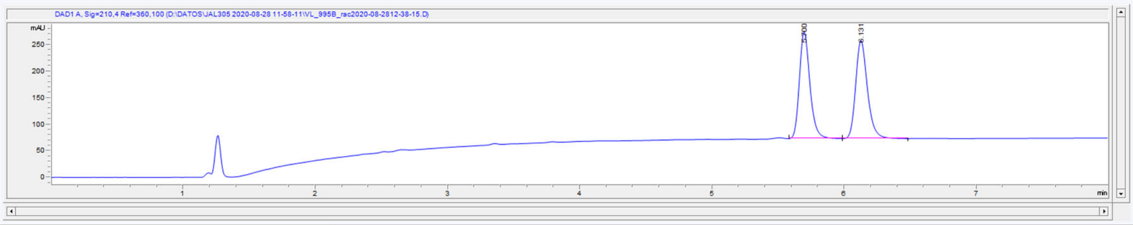

File Information

LC-File	VL_995B_rac2020-08-2812-38-15.D
File Path	D:\DATOS\JAL305 2020-08-28 11-58-11
Date	28-Aug-20, 12:41:51
Sample	VL_995B_rac
Sample Info	VL_995B_rac
Barcode	
Operator	SYSTEM
Method	IA-gradiente5_40_MeOH_MS.M
Reference	D:\DATOS\JAL305 2020-08-28 11-58-11\no Sample Name2020-08-2

#	Time	Type	Area	Height	Width	Area%	Symmetry
1	5.7	BB	1116.3	201.9	0.0845	49.408	0.767
2	6.131	BB	1143	185.1	0.094	50.592	0.757

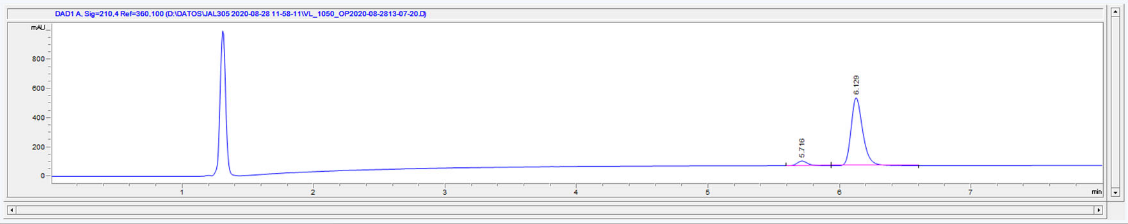

File Information

LC-File	VL_1050_OP2020-08-2813-07-20.D
File Path	D:\DATOS\JAL305 2020-08-28 11-58-11
Date	28-Aug-20, 13:10:50
Sample	VL_1050_OP
Sample Info	VL_1050_OP
Barcode	
Operator	SYSTEM
Method	IA-gradiente5_40_MeOH_MS.M
Reference	D:\DATOS\JAL305 2020-08-28 11-58-11\no Sample Name2020-08-2

#	Time	Type	Area	Height	Width	Area%	Symmetry
1	5.716	BB	180	32.4	0.0849	5.903	0.786
2	6.129	BB	2869.8	464.4	0.0941	94.097	0.724

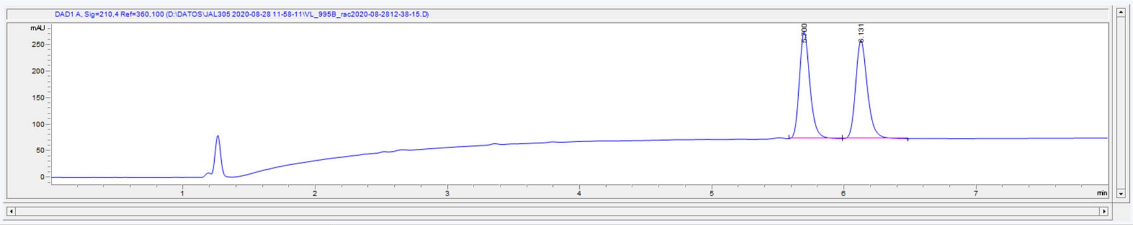

File Information

LC-File	VL_995B_rac2020-08-2812-38-15.D
File Path	D:\DATOS\JAL305 2020-08-28 11-58-11
Date	28-Aug-20, 12:41:51
Sample	VL_995B_rac
Sample Info	VL_995B_rac
Barcode	
Operator	SYSTEM
Method	IA-gradiente5_40_MeOH_MS.M
Reference	D:\DATOS\JAL305 2020-08-28 11-58-11\no Sample Name2020-08-2

#	Time	Type	Area	Height	Width	Area%	Symmetry
1	5.7	BB	1116.3	201.9	0.0845	49.408	0.767
2	6.131	BB	1143	185.1	0.094	50.592	0.757

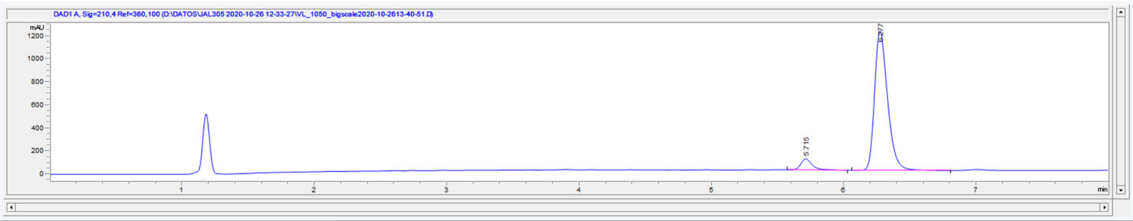

File Information

LC-File	VL_1050_bigscale2020-10-2613-40-51.D
File Path	D:\DATOS\JAL305 2020-10-26 12-33-27
Date	26-Oct-20, 13:44:09
Sample	VL_1050_bigscale
Sample Info	VL_1050_bigscale
Barcode	
Operator	SYSTEM
Method	IA-gradiente5_40_MeOH_MS.M
Reference	D:\DATOS\JAL305 2020-10-26 12-33-27\no Sample Name2020-10-2

#	Time	Type	Area	Height	Width	Area%	Symmetry
1	5.715	BB	574.4	94.6	0.0908	6.514	0.71
2	6.277	BB	8243.5	1208.6	0.1056	93.486	0.679

(*2R,3S,4S*)-4-(4-Bromophenyl)-3-phenyl-1-tosyl-1,2,3,4-tetrahydrobenzofuro[3,2-*b*]pyridin-2-ol (5b)

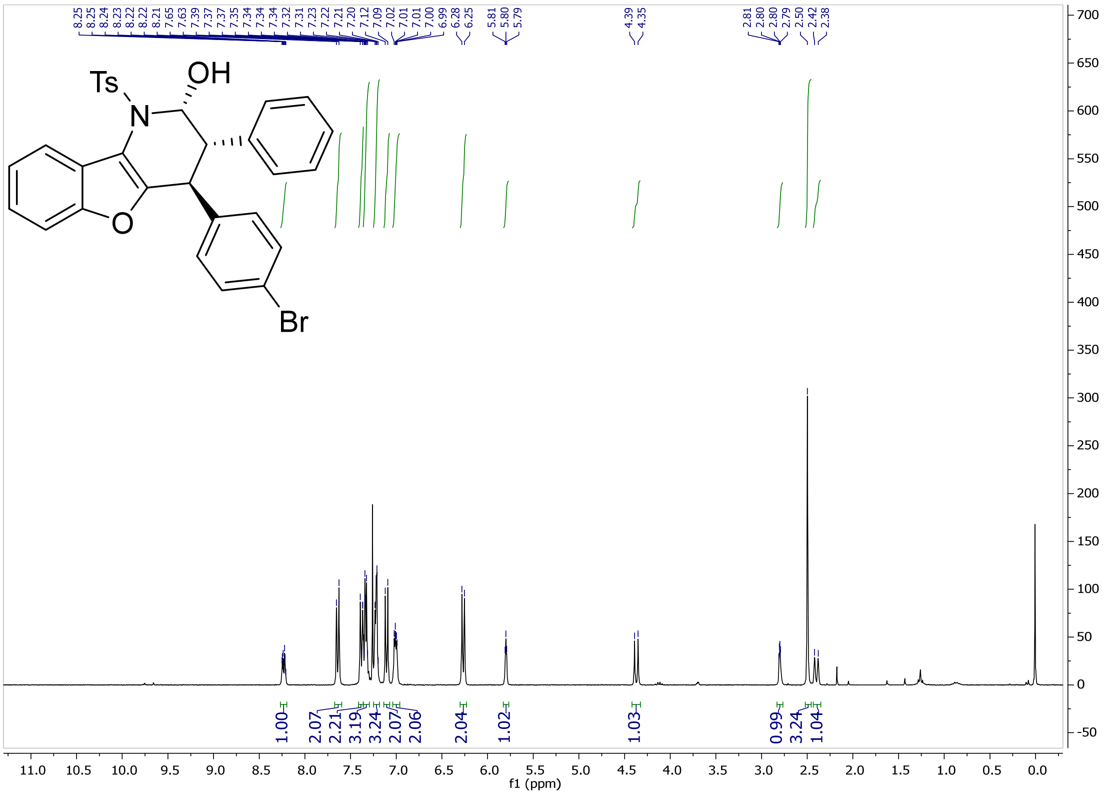

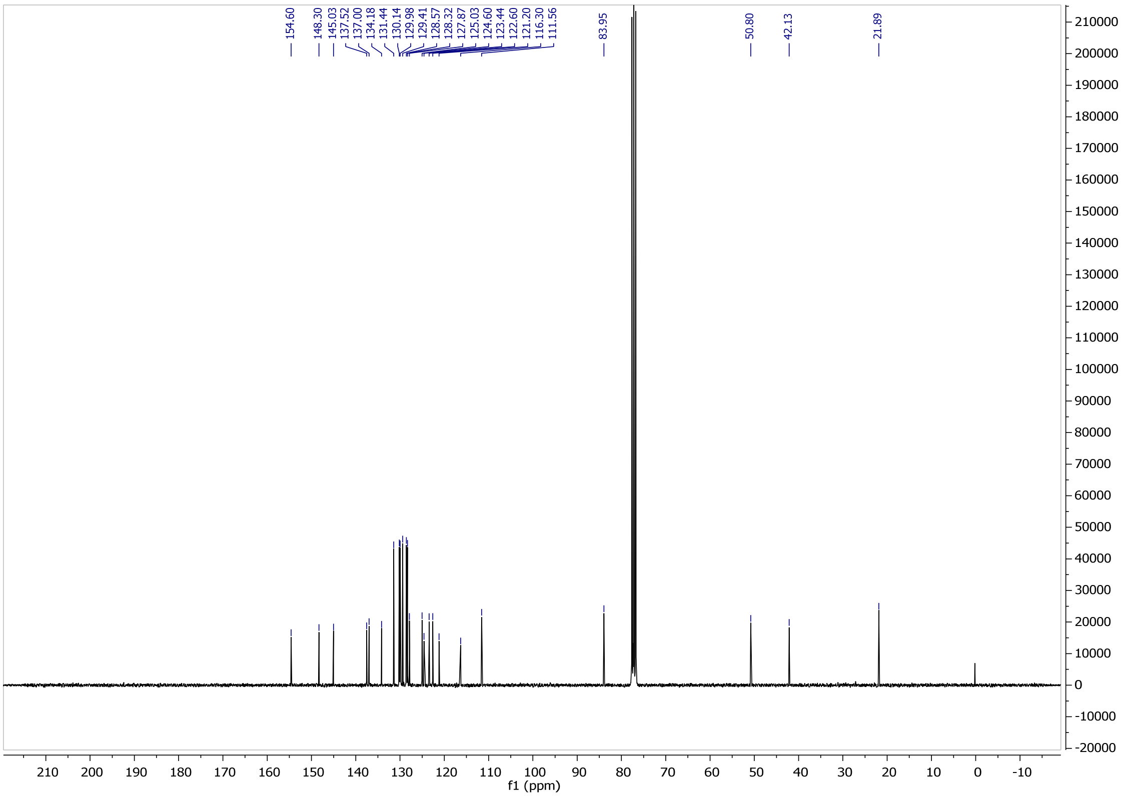

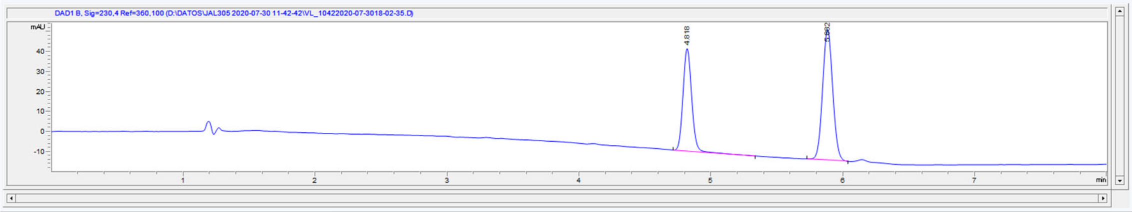

File Information	
LC-File	VL_10422020-07-3018-02-35.D
File Path	D:\DATOS\JAL305 2020-07-30 11-42-42
Date	30-Jul-20, 18:05:06
Sample	VL_1042
Sample Info	VL_1042
Barcode	
Operator	SYSTEM
Method	IC-gradiente5_40_MeOH_NO_MS.M
[illegible]	[illegible]

#	Time	Type	Area	Height	Width	Area%	Symmetry
1	4.818	BB	233.3	51.2	0.0692	40.263	0.859
2	5.882	BB	346.2	65.1	0.084	59.737	0.92

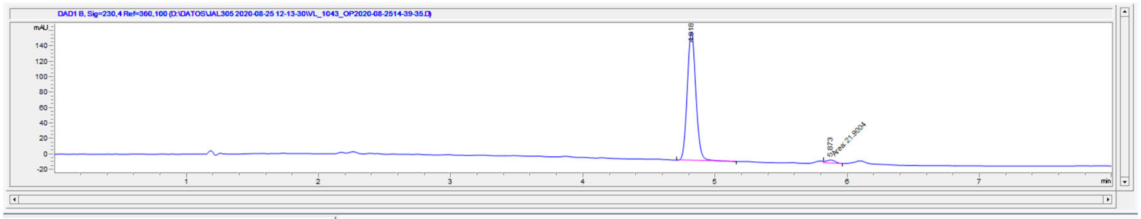

File Information	
LC-File	VL_1043_OP2020-08-2514-39-35.D
File Path	D:\DATOS\JAL305 2020-08-25 12-13-30
Date	25-Aug-20, 14:42:04
Sample	VL_1043_OP
Sample Info	VL_1043_OP
Barcode	
Operator	SYSTEM
Method	IC-gradiente5_40_MeOH_NO_MS.M
[illegible]	[illegible]

#	Time	Type	Area	Height	Width	Area%	Symmetry
1	4.818	BB	765.4	169.2	0.0688	97.218	0.861
2	5.873	FM	21.9	4.6	0.0802	2.782	0.958

(*2R,3S,4S*)-4-(4-Fluorophenyl)-3-phenyl-1-tosyl-1,2,3,4-tetrahydrobenzofuro[3,2-*b*]pyridin-2-ol (5c)

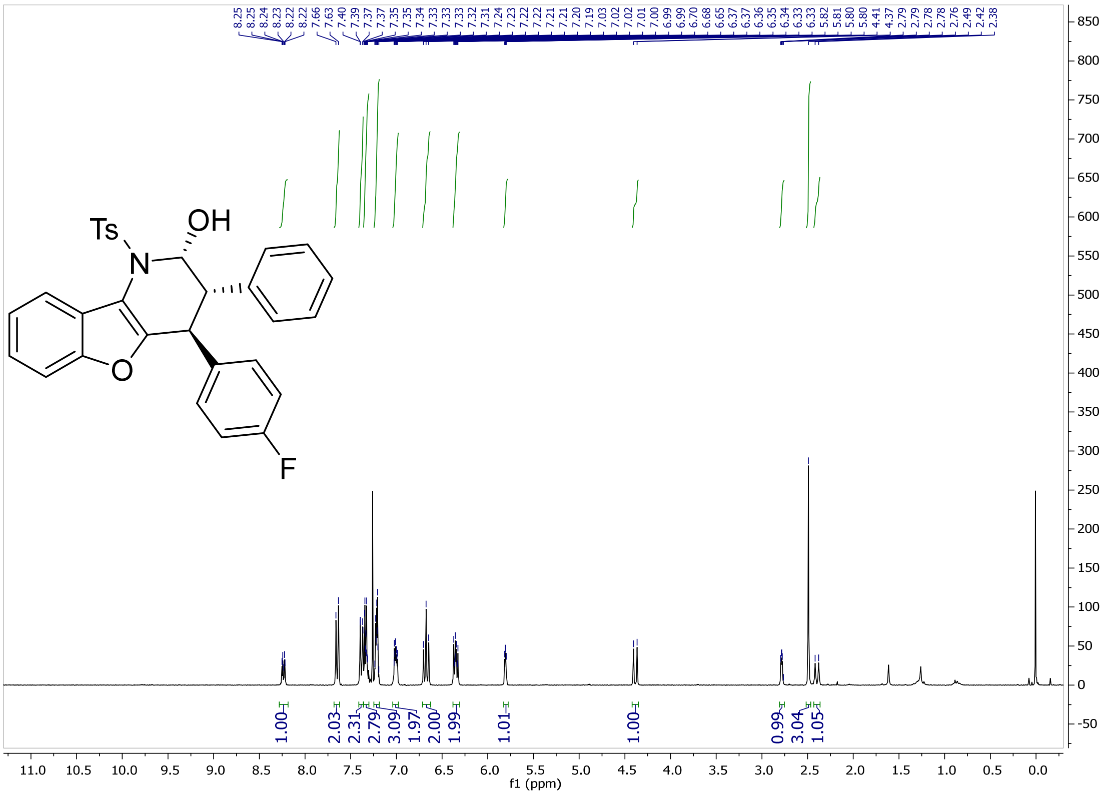

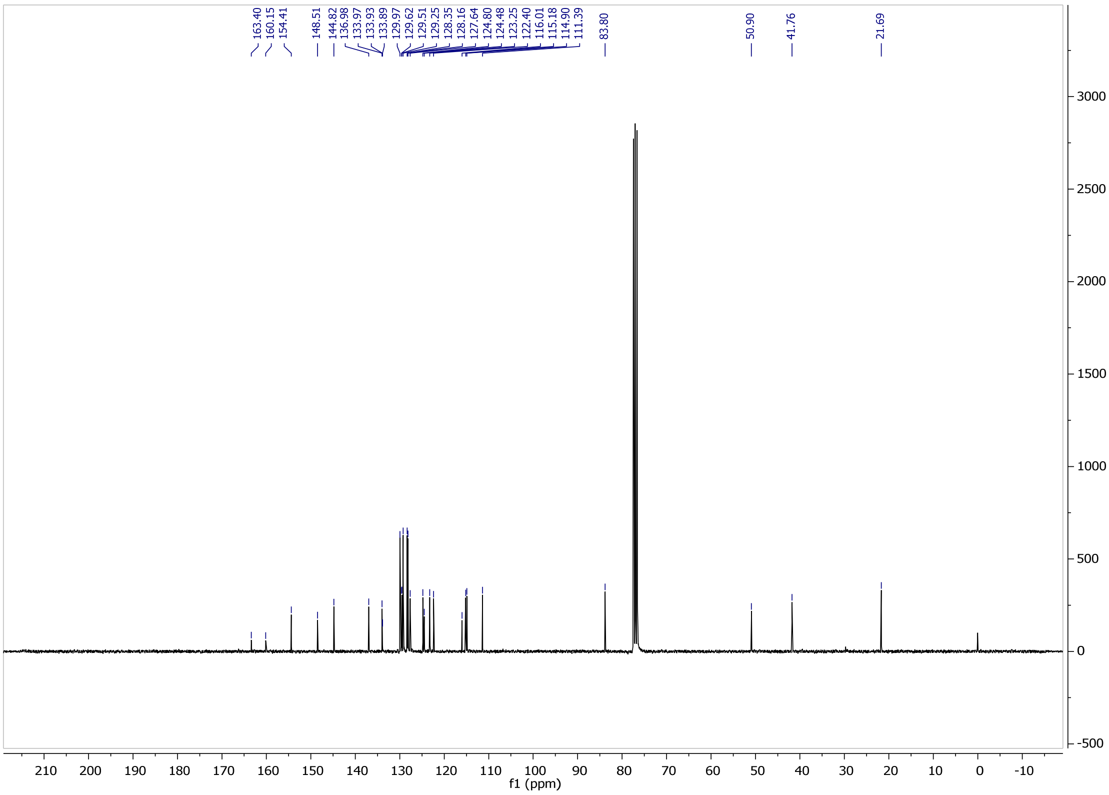

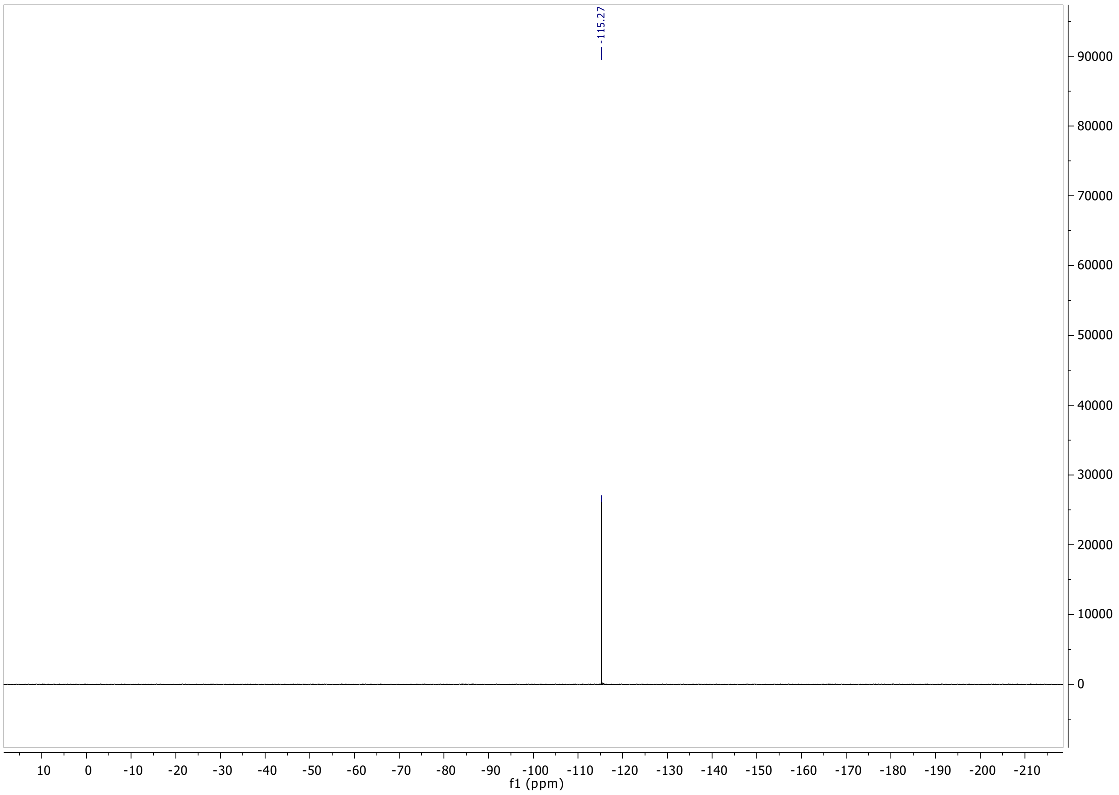

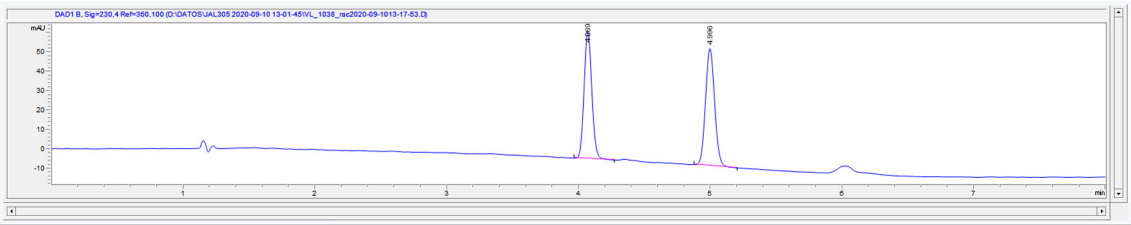

File Information

LC-File	VL_1038_rac2020-09-1013-17-53.D
File Path	D:\DATOS\JAL305 2020-09-10 13-01-45
Date	10-Sep-20, 13:21:24
Sample	VL_1038_rac
Sample Info	VL_1038_rac
Barcode	
Operator	SYSTEM
Method	IC-gradiente5_40_MeOH_MS.M
Reference	D:\DATOS\JAL305 2020-09-10 13-01-45\no Sample Name2020-09-:

#	Time	Type	Area	Height	Width	Area%	Symmetry
1	4.069	BB	269.6	65.8	0.0638	48.043	0.874
2	4.996	BB	291.6	60.7	0.074	51.957	0.907

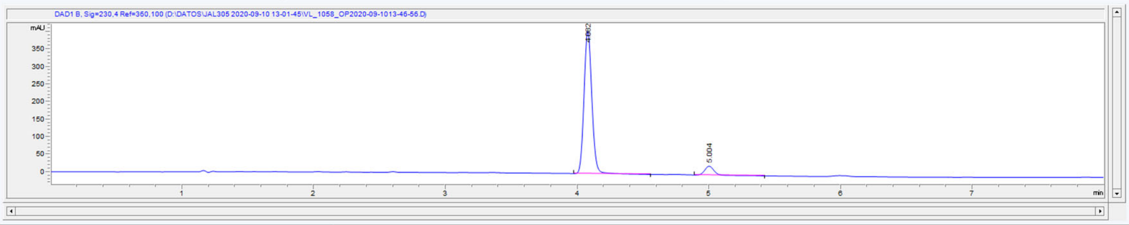

File Information

LC-File	VL_1058_OP2020-09-1013-46-56.D
File Path	D:\DATOS\JAL305 2020-09-10 13-01-45
Date	10-Sep-20, 13:50:24
Sample	VL_1058_OP
Sample Info	VL_1058_OP
Barcode	
Operator	SYSTEM
Method	IC-gradiente5_40_MeOH_MS.M
Reference	D:\DATOS\JAL305 2020-09-10 13-01-45\no Sample Name2020-09-:

#	Time	Type	Area	Height	Width	Area%	Symmetry
1	4.082	BB	1687.5	407.6	0.0643	93.092	0.849
2	5.004	BB	125.2	25.6	0.0751	6.908	0.851

(*2R,3S,4S*)-4-(4-Nitrophenyl)-3-phenyl-1-tosyl-1,2,3,4-tetrahydrobenzofuro[3,2-*b*]pyridin-2-ol (5d)

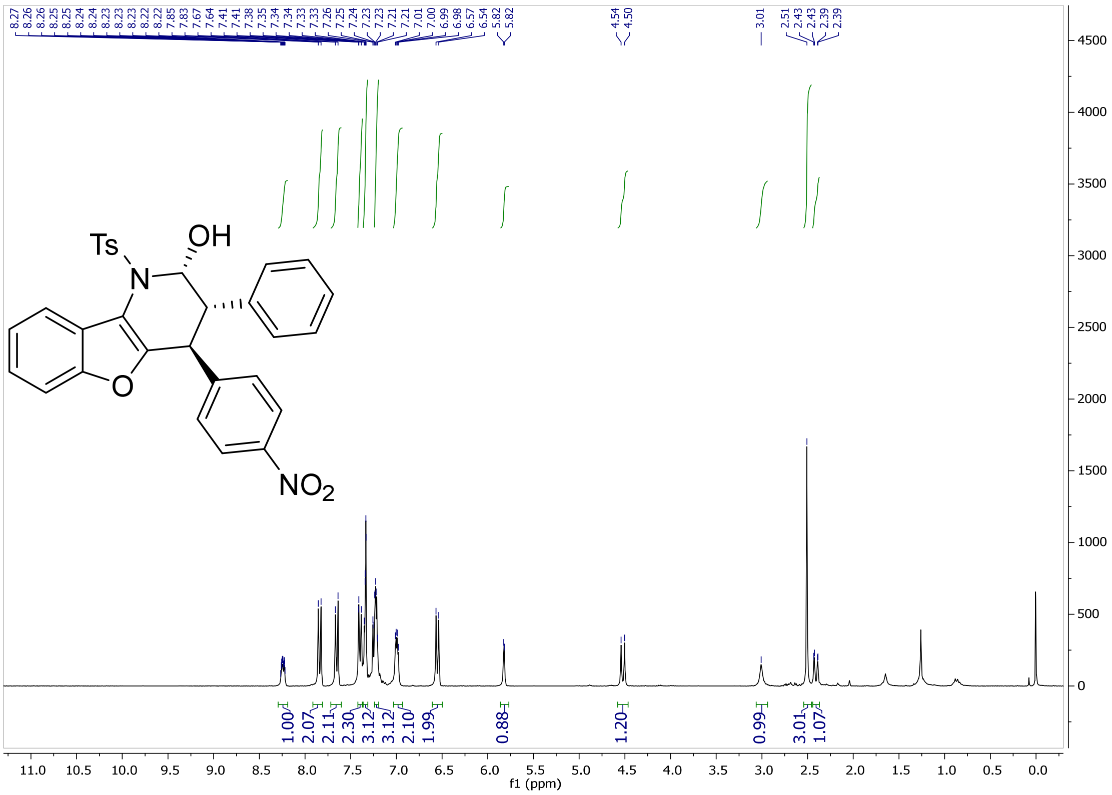

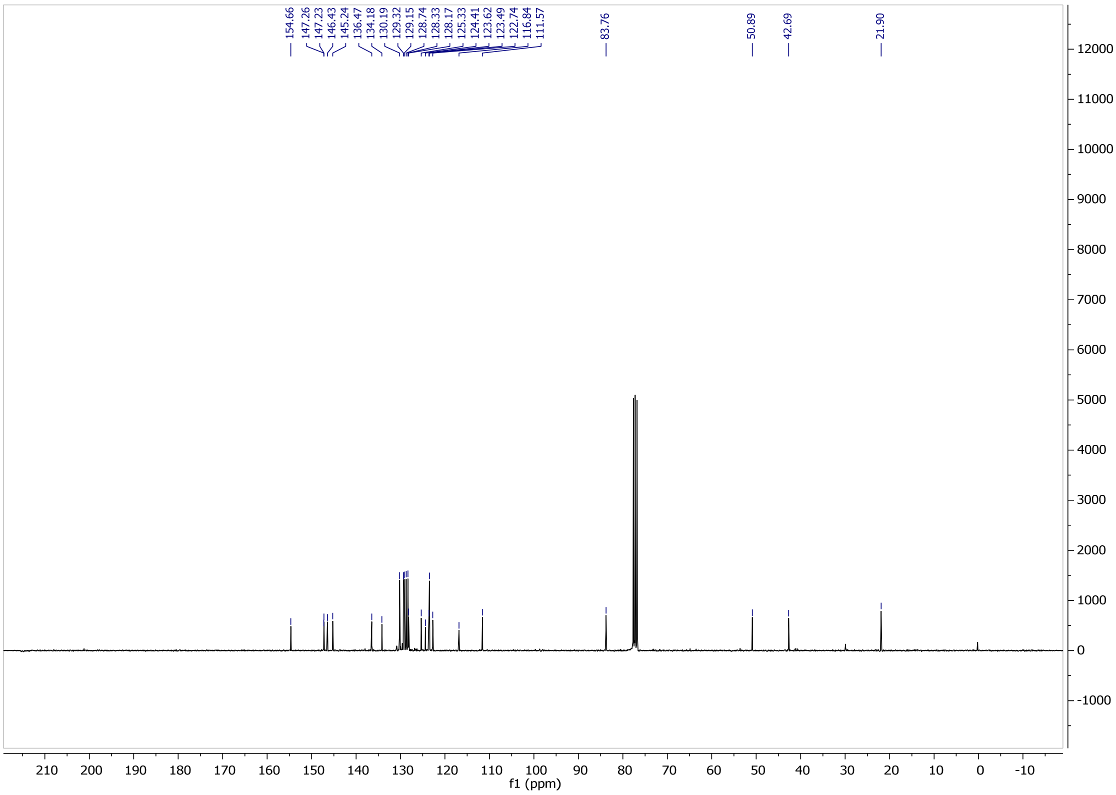

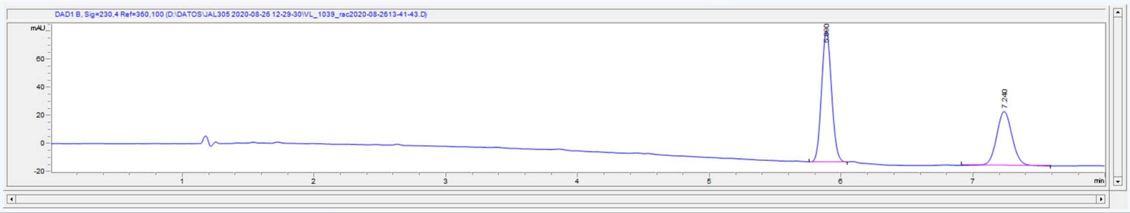

File Information

LC-File	VL_1039_rac2020-08-2613-41-43.D
File Path	D:\DATOS\JAL305 2020-08-26 12-29-30
Date	26-Aug-20, 13:44:23
Sample	VL_1039_rac
Sample Info	VL_1039_rac
Barcode	
Operator	SYSTEM
Method	IC-gradiente5_40_MeOH_NO_MS.M
[illegible]	[illegible]

#	Time	Type	Area	Height	Width	Area%	Symmetry
1	5.89	BB	491.9	93.6	0.0813	60.868	0.892
2	7.24	BB	316.2	38.5	0.1258	39.132	0.916

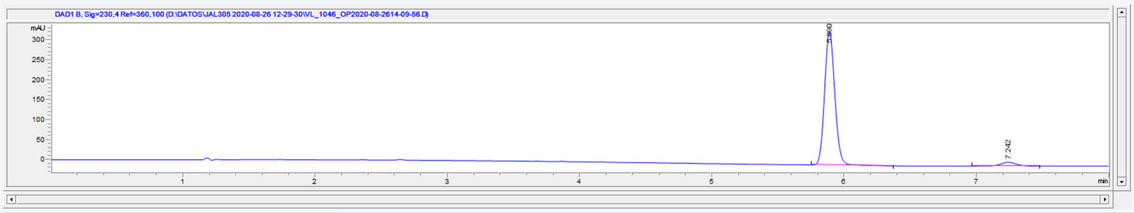

File Information

LC-File	VL_1046_OP2020-08-2614-09-56.D
File Path	D:\DATOS\JAL305 2020-08-26 12-29-30
Date	26-Aug-20, 14:12:25
Sample	VL_1046_OP
Sample Info	VL_1046_OP
Barcode	
Operator	SYSTEM
Method	IC-gradiente5_40_MeOH_NO_MS.M
[illegible]	[illegible]

#	Time	Type	Area	Height	Width	Area%	Symmetry
1	5.89	BB	1804.1	336	0.0826	95.890	0.858
2	7.242	BB	77.3	9.5	0.1268	4.110	0.939

(*2R,3S,4S*)-3-Phenyl-1-tosyl-4-(4-(trifluoromethyl)phenyl)-1,2,3,4-tetrahydrobenzofuro[3,2-*b*]pyridin-2-ol (5e)

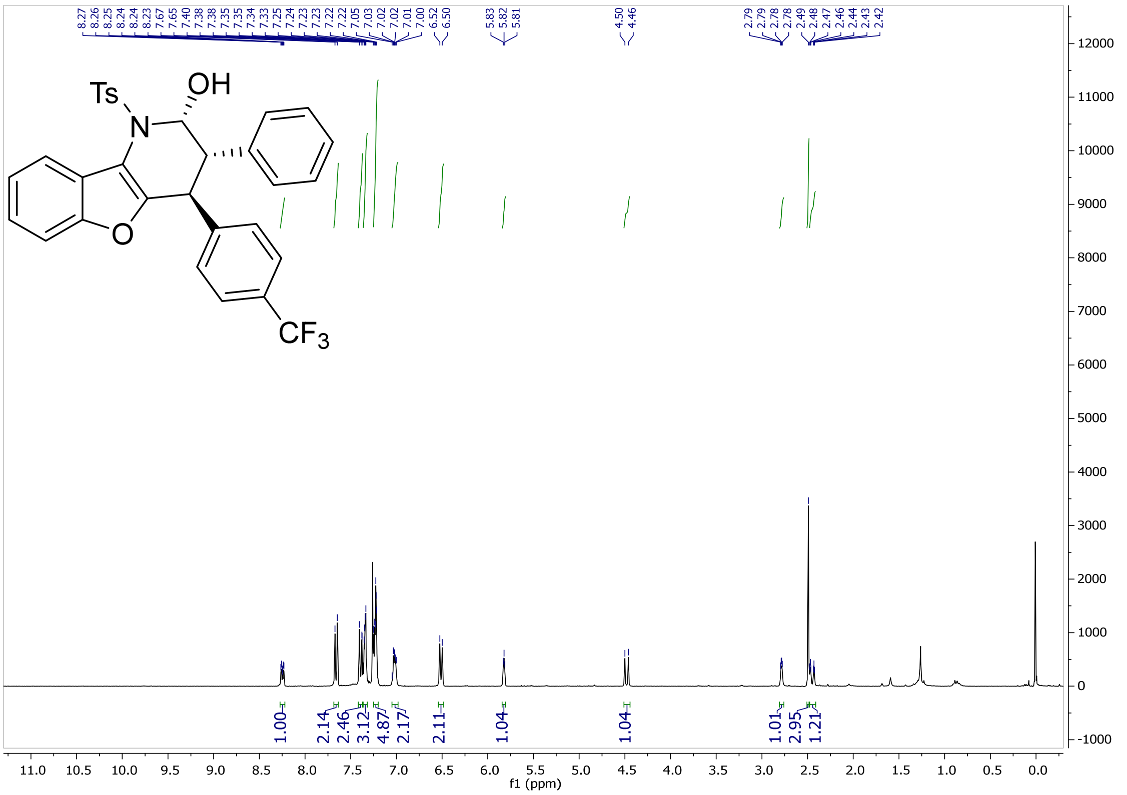

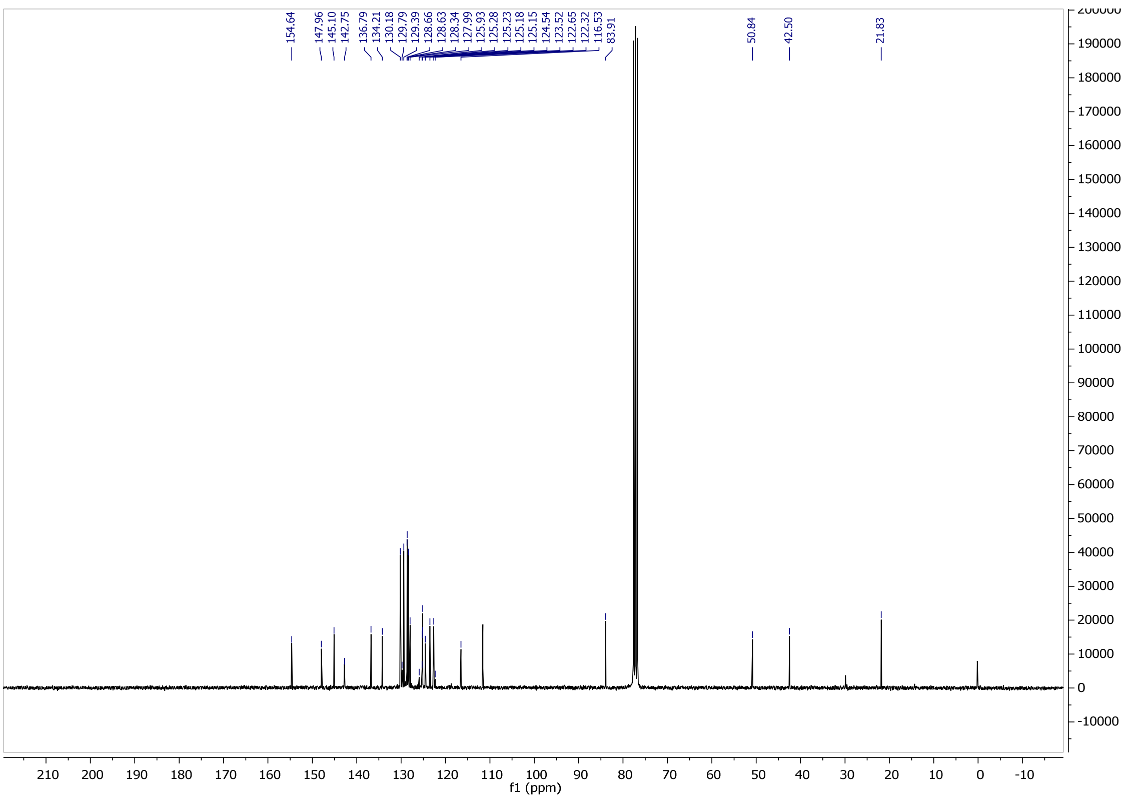

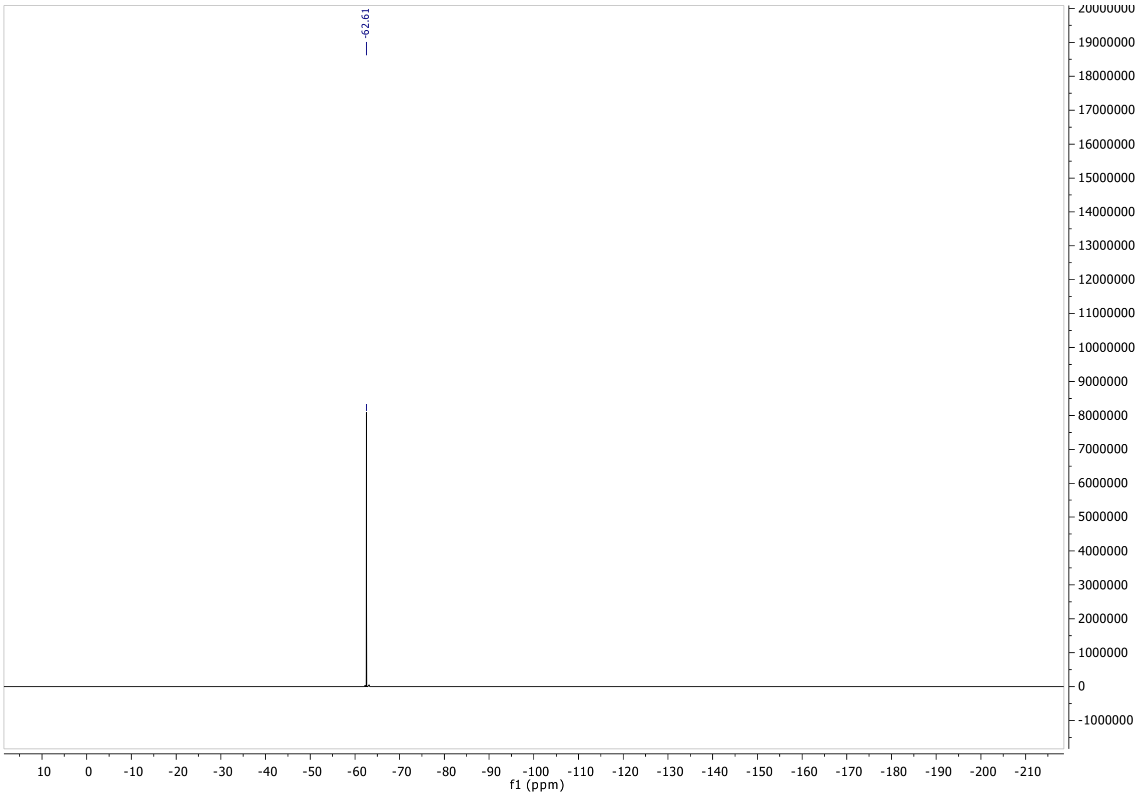

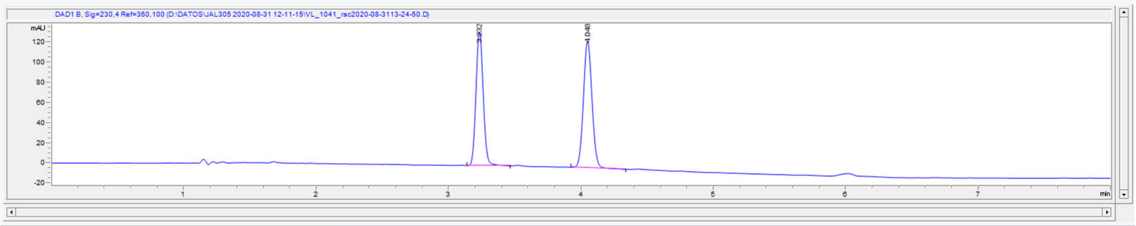

File Information

LC-File	VL_1041_rac2020-08-3113-24-50.D
File Path	D:\DATOS\JAL305 2020-08-31 12-11-15
Date	31-Aug-20, 13:28:14
Sample	VL_1041_rac
Sample Info	VL_1041_rac
Barcode	
Operator	SYSTEM
Method	IC-gradiente5_40_MeOH_MS.M
Reference	D:\DATOS\JAL305 2020-08-31 12-11-15\no Sample Name2020-08-:

#	Time	Type	Area	Height	Width	Area%	Symmetry
1	3.232	BB	507	133.9	0.0602	47.182	0.878
2	4.048	BB	567.6	126	0.0706	52.818	0.906

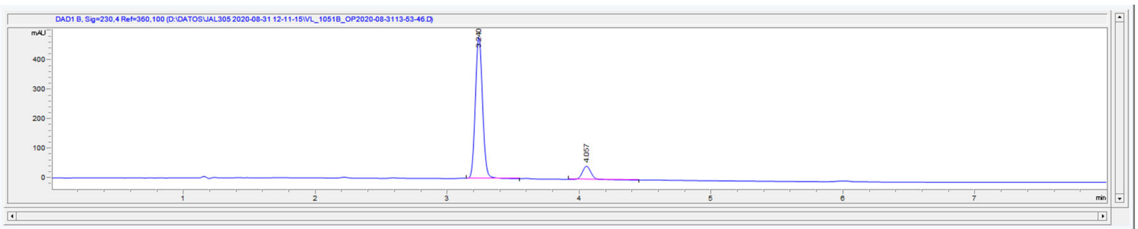

File Information

LC-File	VL_1051B_OP2020-08-3113-53-46.D
File Path	D:\DATOS\JAL305 2020-08-31 12-11-15
Date	31-Aug-20, 13:57:12
Sample	VL_1051B_OP
Sample Info	VL_1051B_OP
Barcode	
Operator	SYSTEM
Method	IC-gradiente5_40_MeOH_MS.M
Reference	D:\DATOS\JAL305 2020-08-31 12-11-15\no Sample Name2020-08-:

#	Time	Type	Area	Height	Width	Area%	Symmetry
1	3.24	BB	1818.1	483.2	0.0599	89.828	0.878
2	4.057	BB	205.9	45.4	0.0709	10.172	0.895

4-((*2R,3S,4S*)-2-Hydroxy-3-phenyl-1-tosyl-1,2,3,4-tetrahydrobenzofuro[3,2-*b*]pyridin-4-yl)benzonitrile (5f)

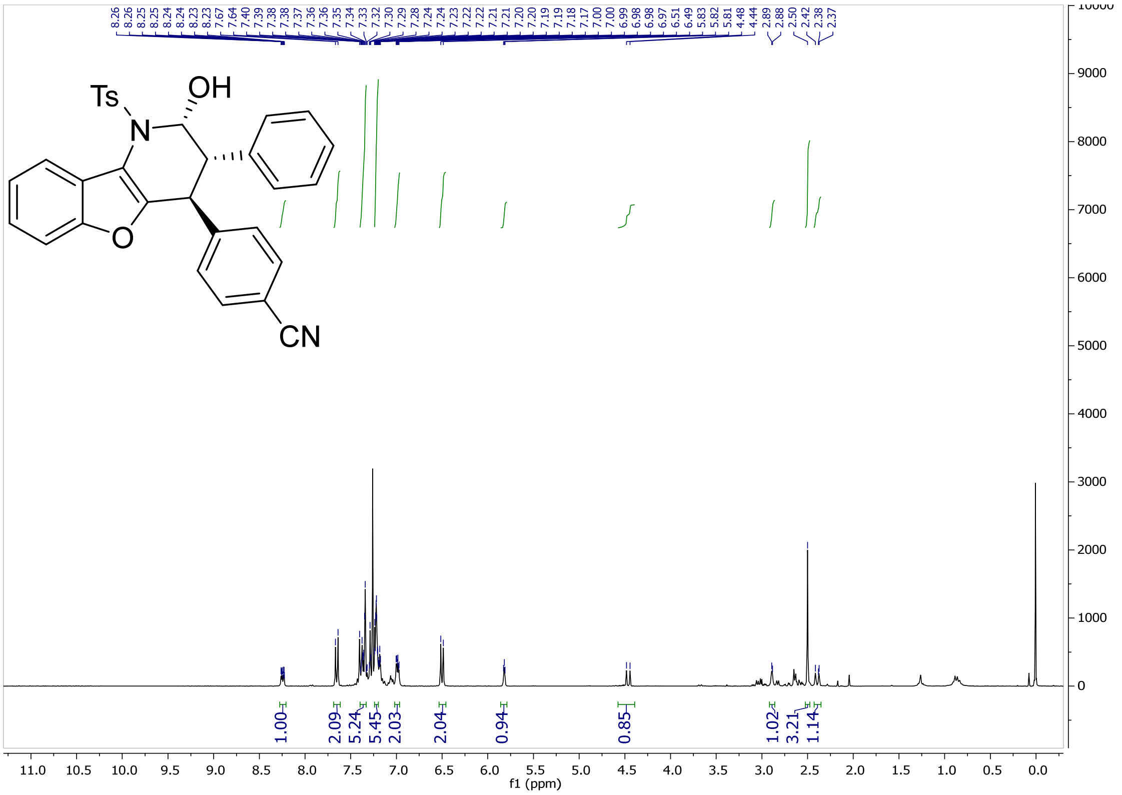

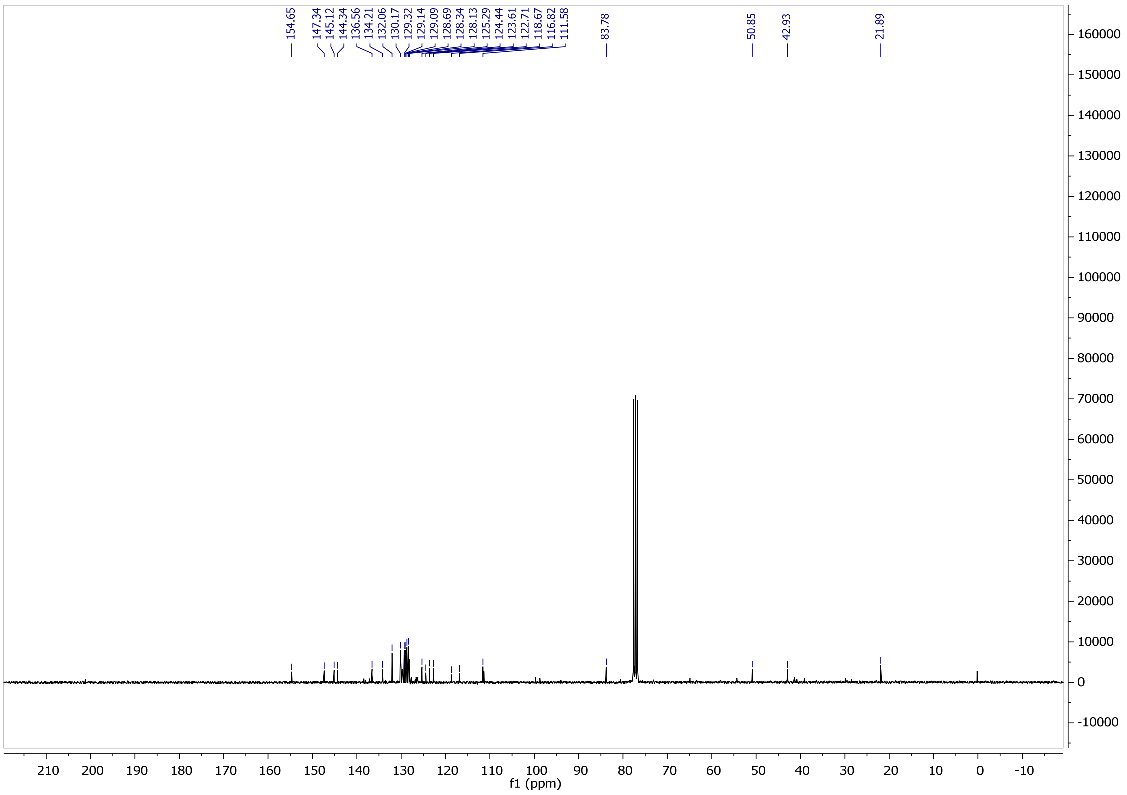

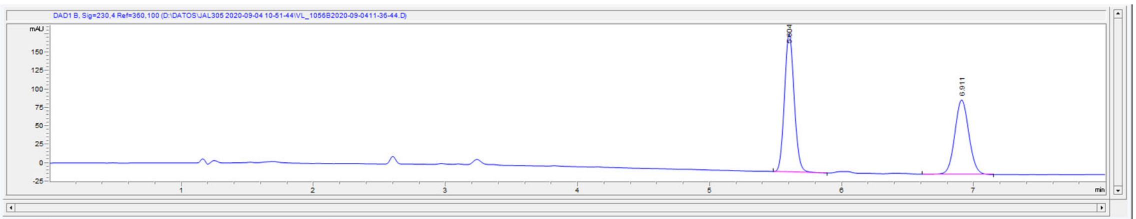

File Information

LC-File	VL_1056B2020-09-0411-36-44.D
File Path	D:\DATOS\JAL305 2020-09-04 10-51-44
Date	04-Sep-20, 11:40:06
Sample	VL_1056B
Sample Info	VL_1056B
Barcode	
Operator	SYSTEM
Method	IC-gradiente5_40_MeOH_MS.M
Reference	D:\DATOS\JAL305 2020-09-04 10-51-44\no Sample Name2020-09-(

#	Time	Type	Area	Height	Width	Area%	Symmetry
1	5.604	BB	969	189.4	0.0777	56.120	0.875
2	6.911	BB	757.7	102.2	0.1145	43.880	0.914

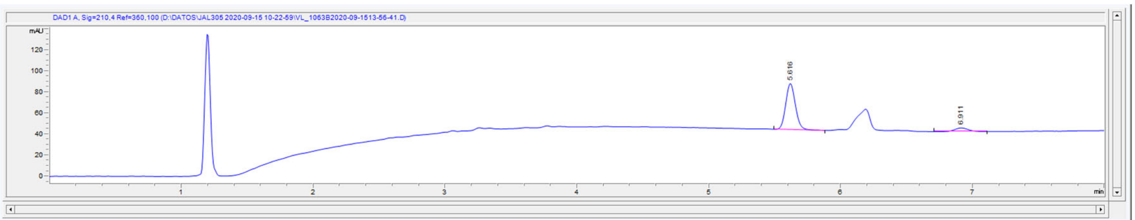

File Information

LC-File	VL_1063B2020-09-1513-56-41.D
File Path	D:\DATOS\JAL305 2020-09-15 10-22-59
Date	15-Sep-20, 14:00:06
Sample	VL_1063B
Sample Info	VL_1063B
Barcode	
Operator	SYSTEM
Method	IC-gradiente5_40_MeOH_MS.M
Reference	D:\DATOS\JAL305 2020-09-15 10-22-59\no Sample Name2020-09-:

#	Time	Type	Area	Height	Width	Area%	Symmetry
1	5.616	BB	223.9	43.7	0.0797	89.762	0.854
2	6.911	BB	25.5	3.5	0.1106	10.238	0.913

(*2R,3S,4S*)-4-(3-Chlorophenyl)-3-phenyl-1-tosyl-1,2,3,4-tetrahydrobenzofuro[3,2-*b*]pyridin-2-ol (5g)

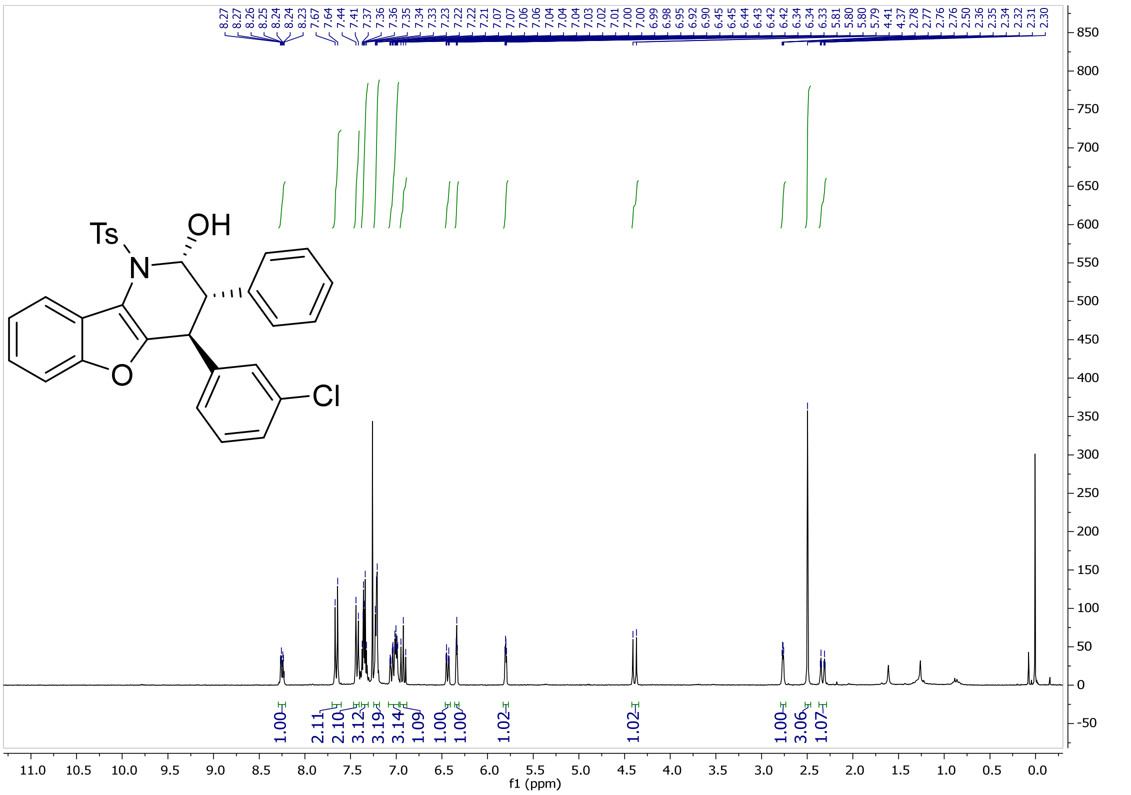

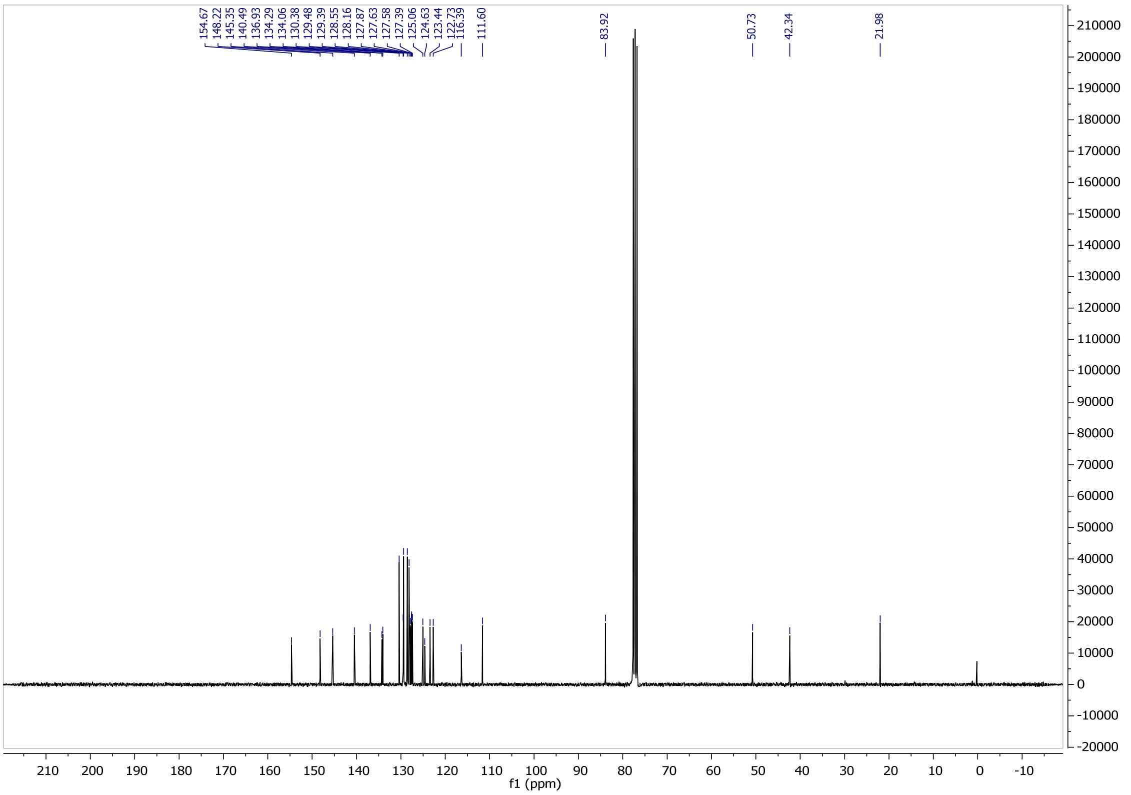

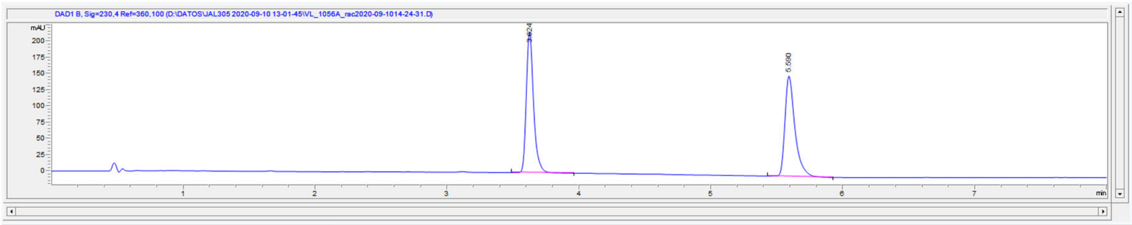

File Information

LC-File	VL_1056A_rac2020-09-1014-24-31.D
File Path	D:\DATOS\JAL305 2020-09-10 13-01-45
Date	10-Sep-20, 14:28:00
Sample	VL_1056A_rac
Sample Info	VL_1056A_rac
Barcode	
Operator	SYSTEM
Method	IB-gradiente5_30_MeOH_MS.M
Reference	D:\DATOS\JAL305 2020-09-10 13-01-45\no Sample Name2020-09-:

#	Time	Type	Area	Height	Width	Area%	Symmetry
1	3.624	BB	835.8	219.1	0.0585	50.491	0.749
2	5.59	BB	819.5	155.7	0.0794	49.509	0.621

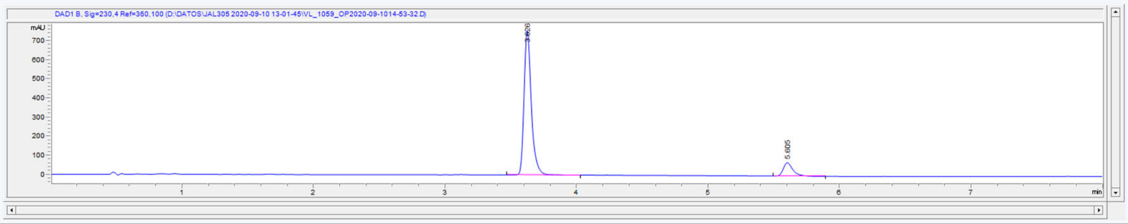

File Information

LC-File	VL_1059_OP2020-09-1014-53-32.D
File Path	D:\DATOS\JAL305 2020-09-10 13-01-45
Date	10-Sep-20, 14:56:40
Sample	VL_1059_OP
Sample Info	VL_1059_OP
Barcode	
Operator	SYSTEM
Method	IB-gradiente5_30_MeOH_MS.M
Reference	D:\DATOS\JAL305 2020-09-10 13-01-45\no Sample Name2020-09-:

#	Time	Type	Area	Height	Width	Area%	Symmetry
1	3.626	BB	2891.4	753	0.0588	89.121	0.721
2	5.605	BB	353	69.8	0.075	10.879	0.67

(*2R,3S,4S*)-4-(2-Fluorophenyl)-3-phenyl-1-tosyl-1,2,3,4-tetrahydrobenzofuro[3,2-*b*]pyridin-2-ol (5h)

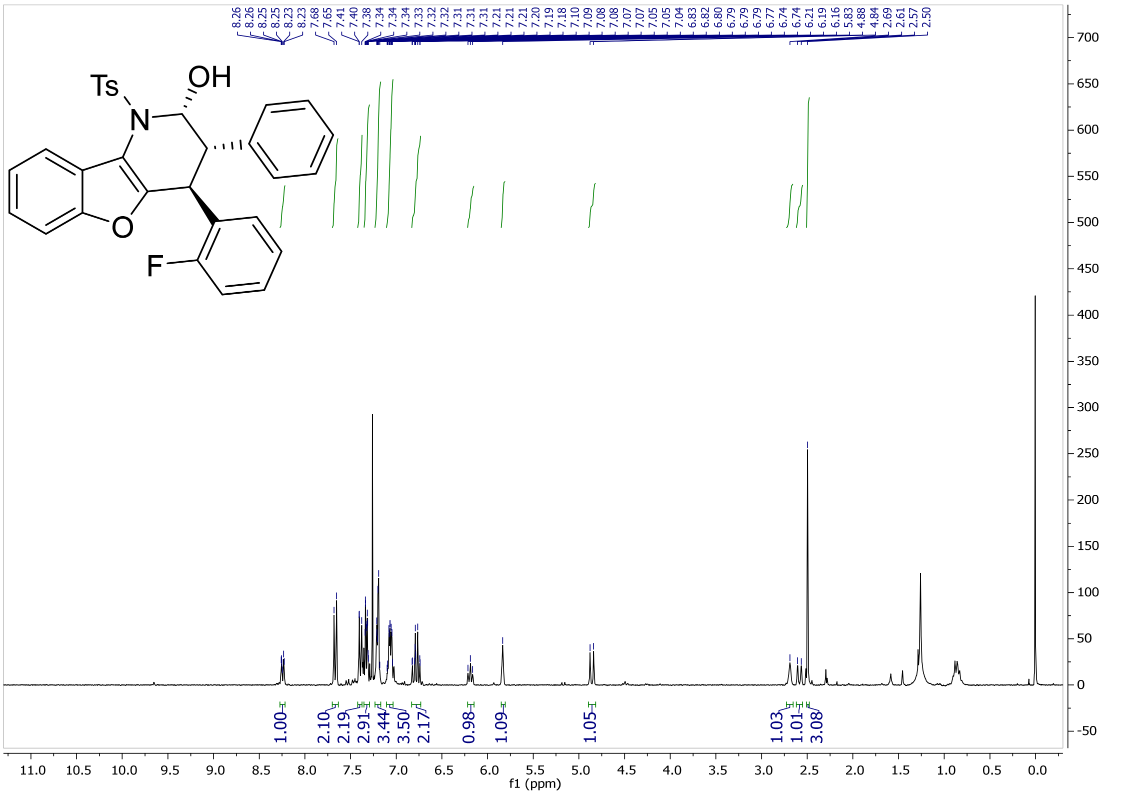

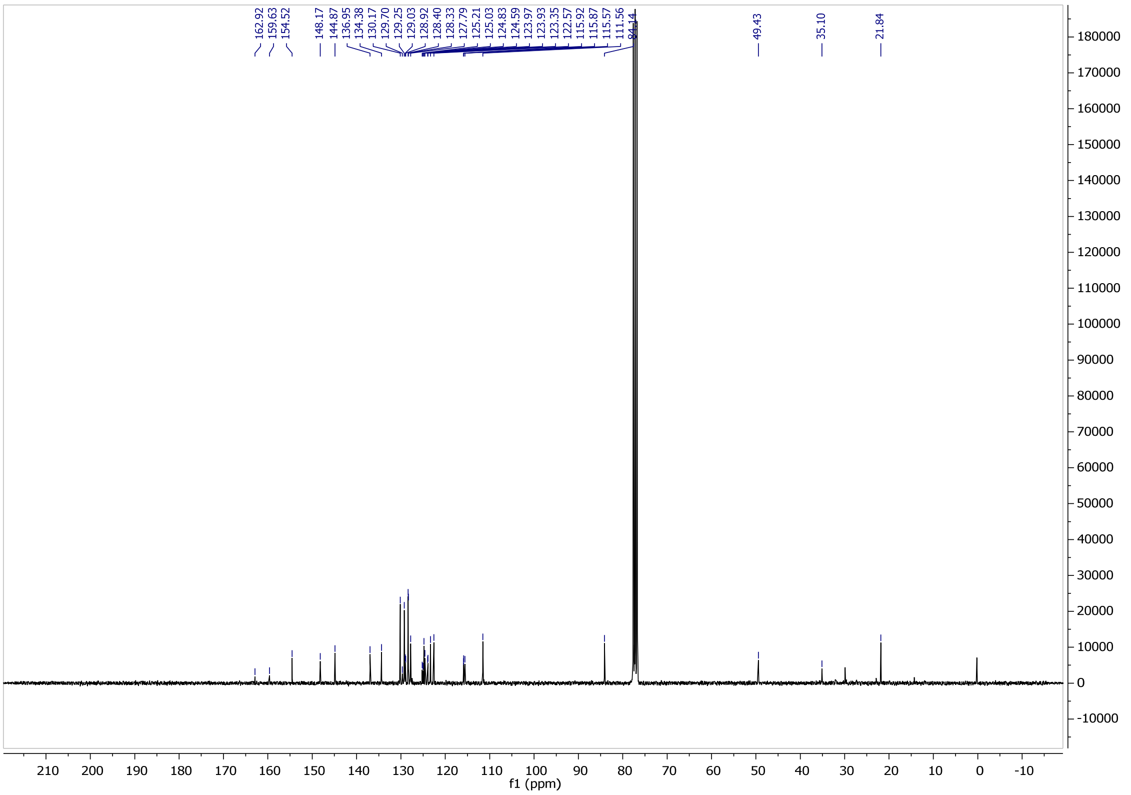

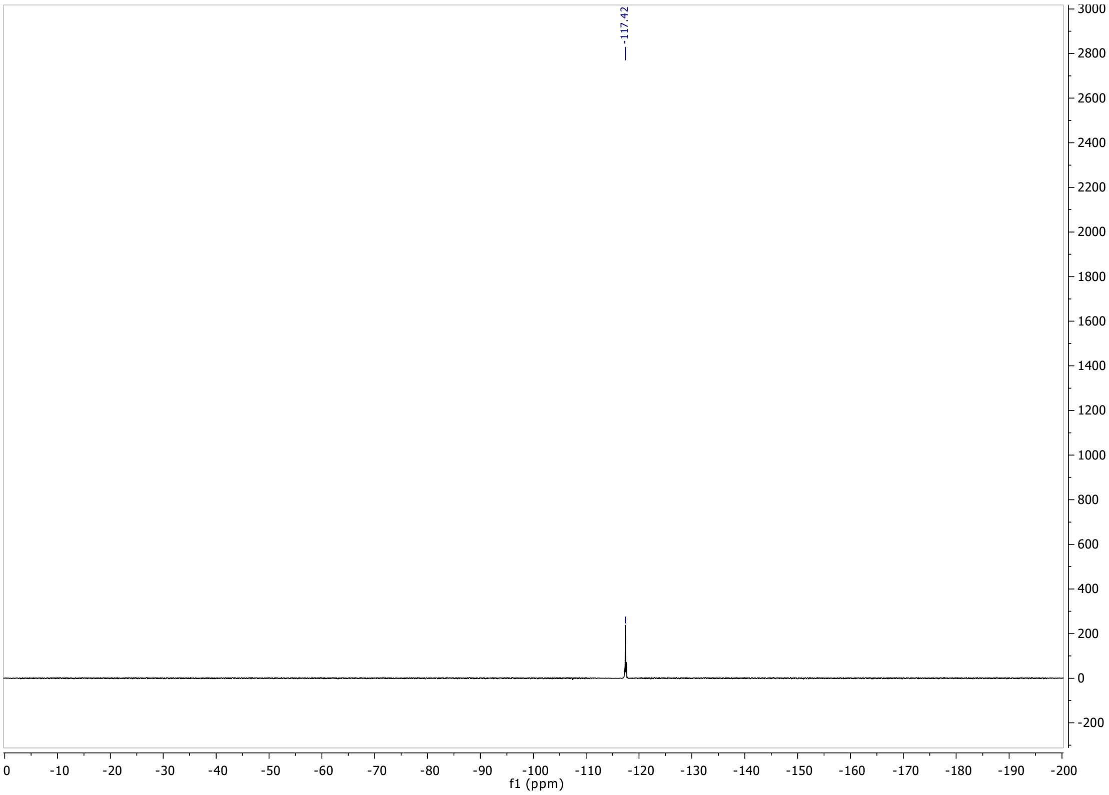

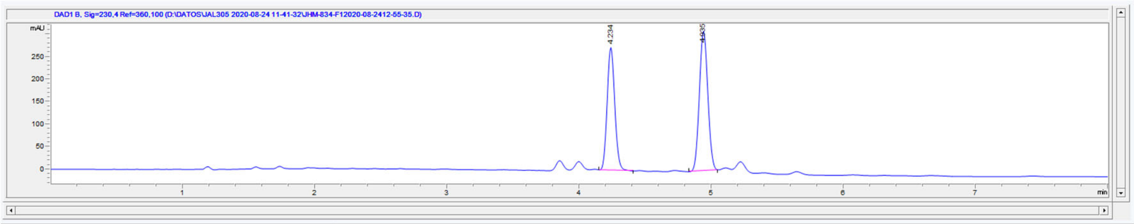

File Information

LC-File	JHM-834-F12020-08-2412-55-35.D
File Path	D:\DATOS\JAL305 2020-08-24 11-41-32
Date	24-Aug-20, 12:58:59
Sample	JHM-834-F1
Sample Info	JHM-834-F1
Barcode	
Operator	SYSTEM
Method	IC-gradiente5_40_MeOH_MS.M
Reference	D:\DATOS\JAL305 2020-08-24 11-41-32\no Sample Name2020-08-2

#	Time	Type	Area	Height	Width	Area%	Symmetry
1	4.234	BB	1087.1	271.4	0.0627	44.241	0.881
2	4.935	BB	1370.1	308.9	0.0697	55.759	0.929

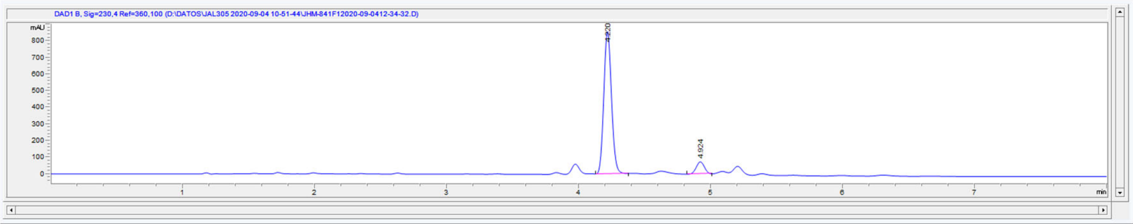

File Information

LC-File	JHM-841F12020-09-0412-34-32.D
File Path	D:\DATOS\JAL305 2020-09-04 10-51-44
Date	04-Sep-20, 12:37:57
Sample	JHM-841F1
Sample Info	JHM-841F1
Barcode	
Operator	SYSTEM
Method	IC-gradiente5_40_MeOH_MS.M
Reference	D:\DATOS\JAL305 2020-09-04 10-51-44\no Sample Name2020-09-(

#	Time	Type	Area	Height	Width	Area%	Symmetry
1	4.22	BB	3474.5	861.4	0.0631	91.715	0.88
2	4.924	BB	313.9	73.3	0.0679	8.285	0.988

(*2R,3S,4S*)-4-(2-Bromophenyl)-3-phenyl-1-tosyl-1,2,3,4-tetrahydrobenzofuro[3,2-*b*]pyridin-2-ol (5i)

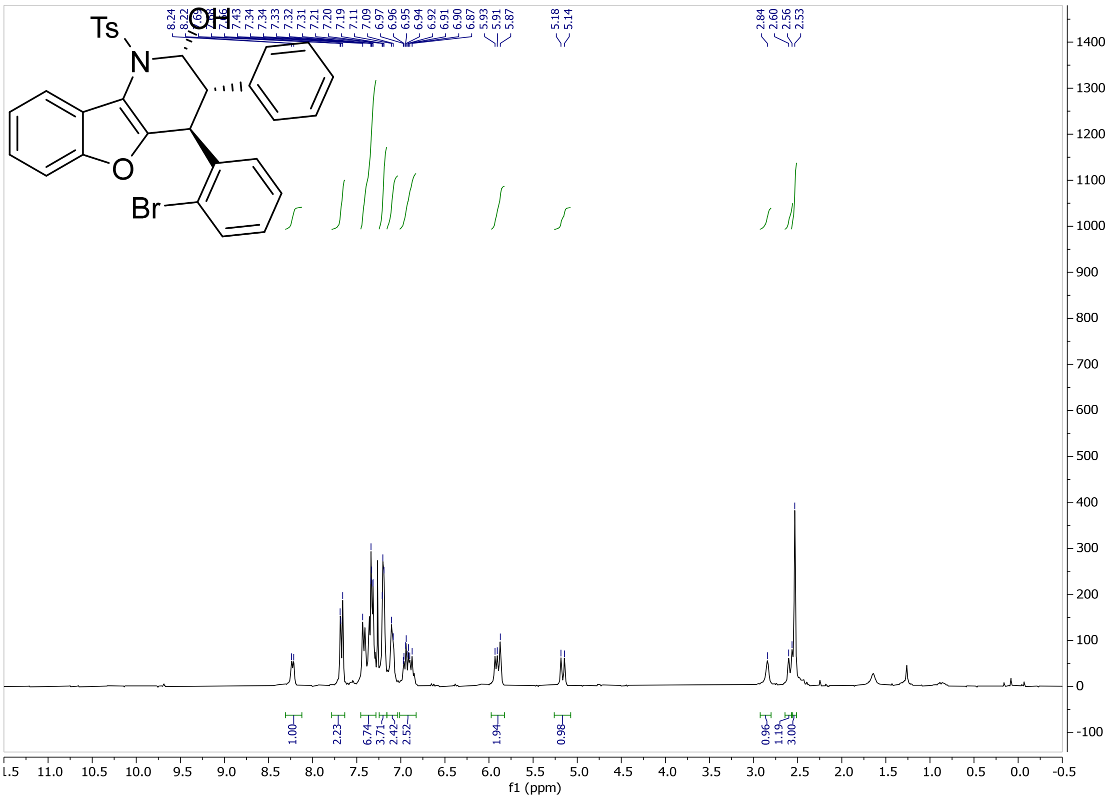

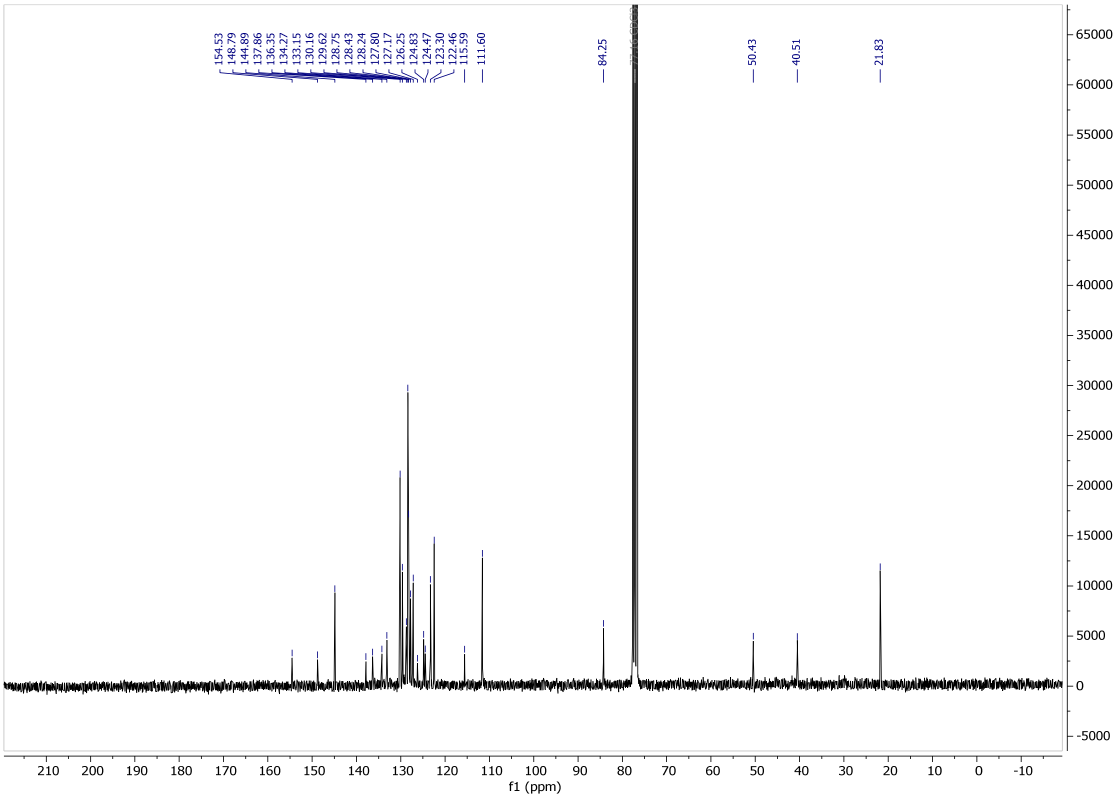

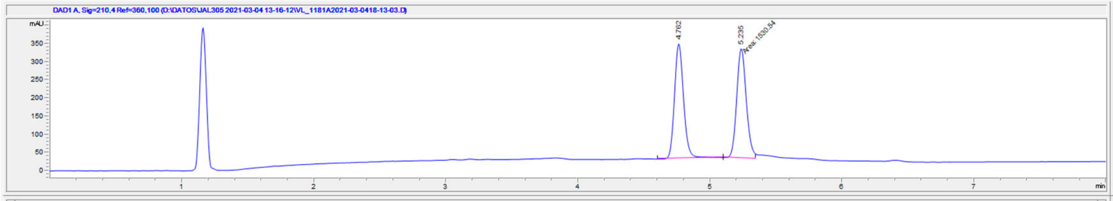

File Information

LC-File	VL_1181A2021-03-0418-13-03.D
File Path	D:\DATOS\JAL305 2021-03-04 13-16-12
Date	04-Mar-21, 18:16:28
Sample	VL_1181A
Sample Info	VL_1181A
Barcode	
Operator	SYSTEM
Method	IC-gradiente5_40_MeOH_MS.M
Reference	D:\DATOS\JAL305 2021-03-04 13-16-12\no Sample Name2021-03-(
Analysis Time	8 min

#	Time	Type	Area	Height	Width	Area%	Symmetry
1	4.762	BB	1527.4	317.7	0.0741	49.948	0.819
2	5.235	MF	1530.5	303.5	0.084	50.052	0.87

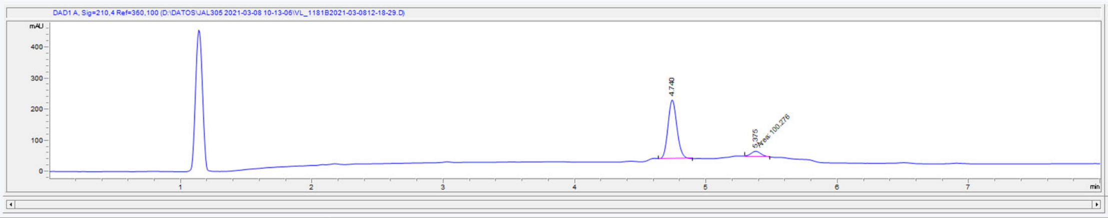

File Information

LC-File	VL_1181B2021-03-0812-18-29.D
File Path	D:\DATOS\JAL305 2021-03-08 10-13-06
Date	08-Mar-21, 12:21:56
Sample	VL_1181B
Sample Info	VL_1181B
Barcode	
Operator	SYSTEM
Method	IC-gradiente5_40_MeOH_MS.M
Reference	D:\DATOS\JAL305 2021-03-08 10-13-06\no Sample Name2021-03-(
Analysis Time	8 min

#	Time	Type	Area	Height	Width	Area%	Symmetry
1	4.74	BB	896.3	189.7	0.0731	89.938	0.845
2	5.375	MM	100.3	18	0.0928	10.062	0.867

(*2R,3S,4S*)-4-(3-Methoxyphenyl)-3-phenyl-1-tosyl-1,2,3,4-tetrahydrobenzofuro[3,2-*b*]pyridin-2-ol (5j)

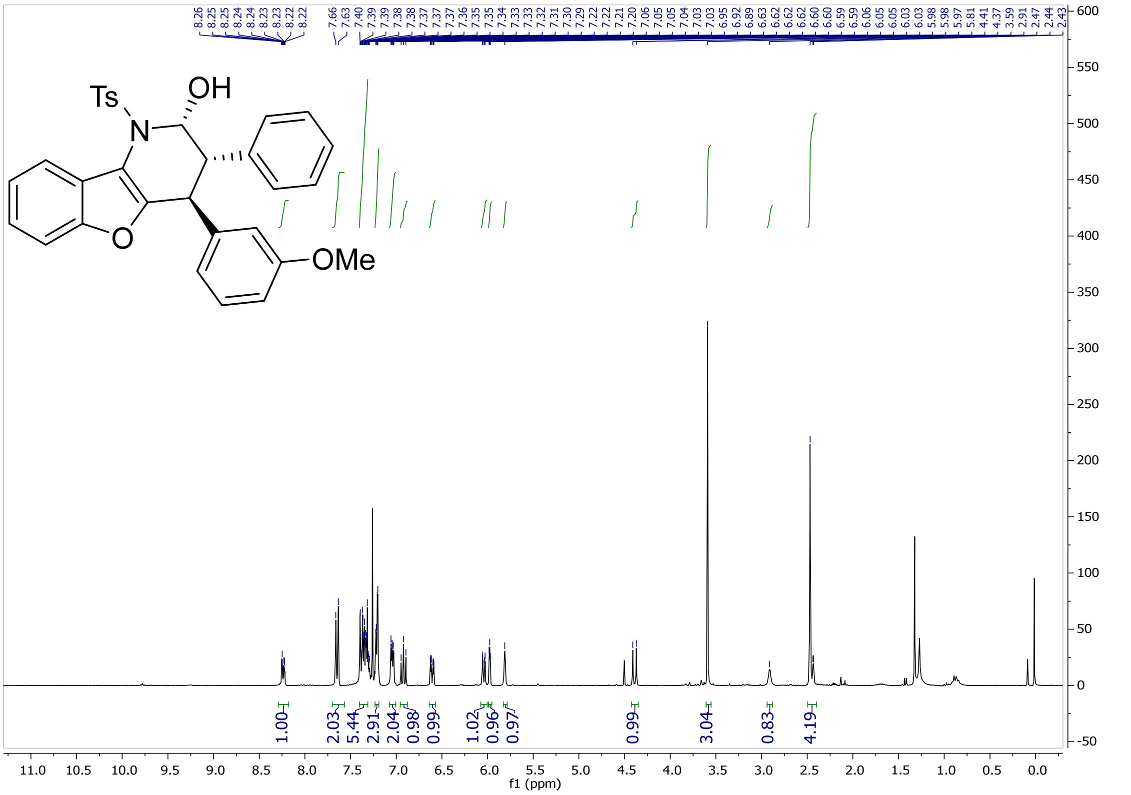

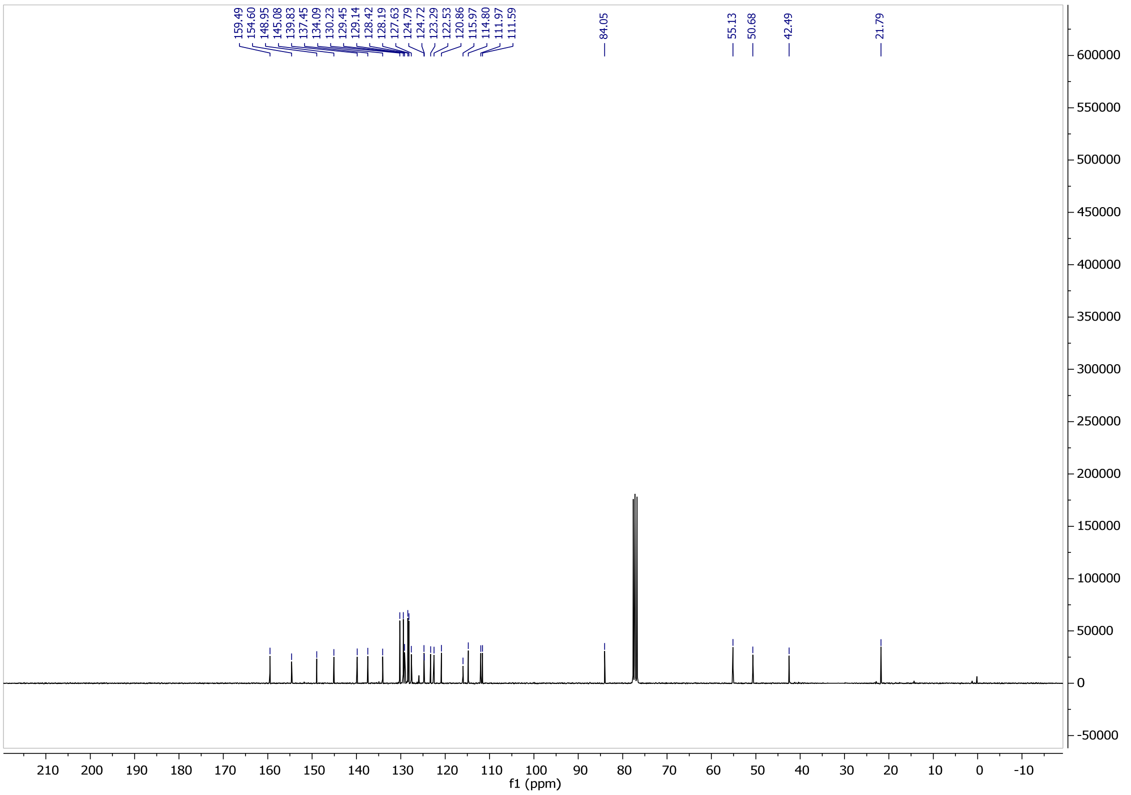

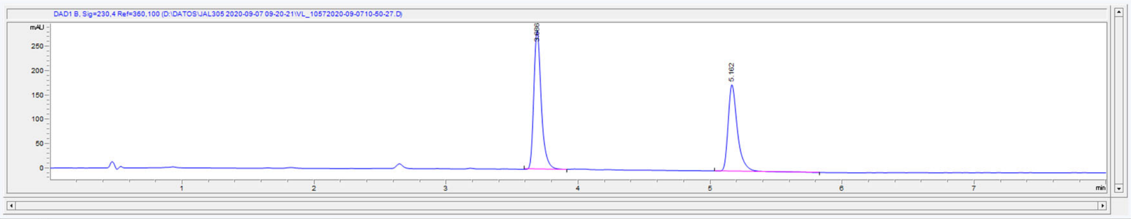

File Information

LC-File	VL_10572020-09-0710-50-27.D
File Path	D:\DATOS\JAL305 2020-09-07 09-20-21
Date	07-Sep-20, 10:53:52
Sample	VL_1057
Sample Info	VL_1057
Barcode	
Operator	SYSTEM
Method	IB-gradiente5_30_MeOH_MS.M
Reference	D:\DATOS\JAL305 2020-09-07 09-20-21\no Sample Name2020-09-(

#	Time	Type	Area	Height	Width	Area%	Symmetry
1	3.686	BB	1113.3	285.4	0.0595	55.956	0.722
2	5.162	BB	876.3	177.8	0.0755	44.044	0.631

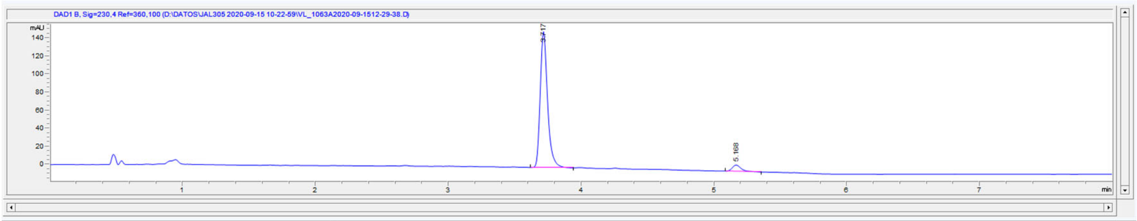

File Information

LC-File	VL_1063A2020-09-1512-29-38.D
File Path	D:\DATOS\JAL305 2020-09-15 10-22-59
Date	15-Sep-20, 12:32:59
Sample	VL_1063A
Sample Info	VL_1063A
Barcode	
Operator	SYSTEM
Method	IB-gradiente5_30_MeOH_MS.M
Reference	D:\DATOS\JAL305 2020-09-15 10-22-59\no Sample Name2020-09-:

#	Time	Type	Area	Height	Width	Area%	Symmetry
1	3.717	BB	577.7	150.6	0.0588	94.650	0.743
2	5.168	BB	32.7	7.1	0.07	5.350	0.701

(*2R,3S,4S*)-4-(Naphthalen-2-yl)-3-phenyl-1-tosyl-1,2,3,4-tetrahydrobenzofuro[3,2-*b*]pyridin-2-ol (5k)

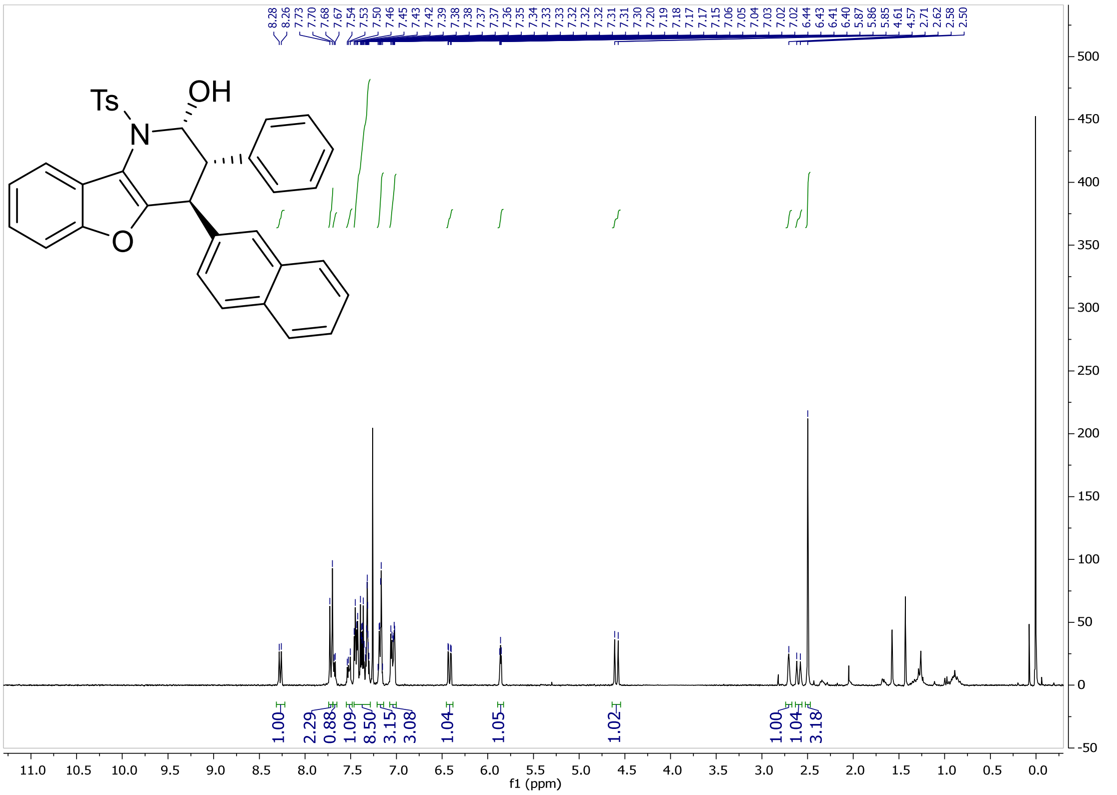

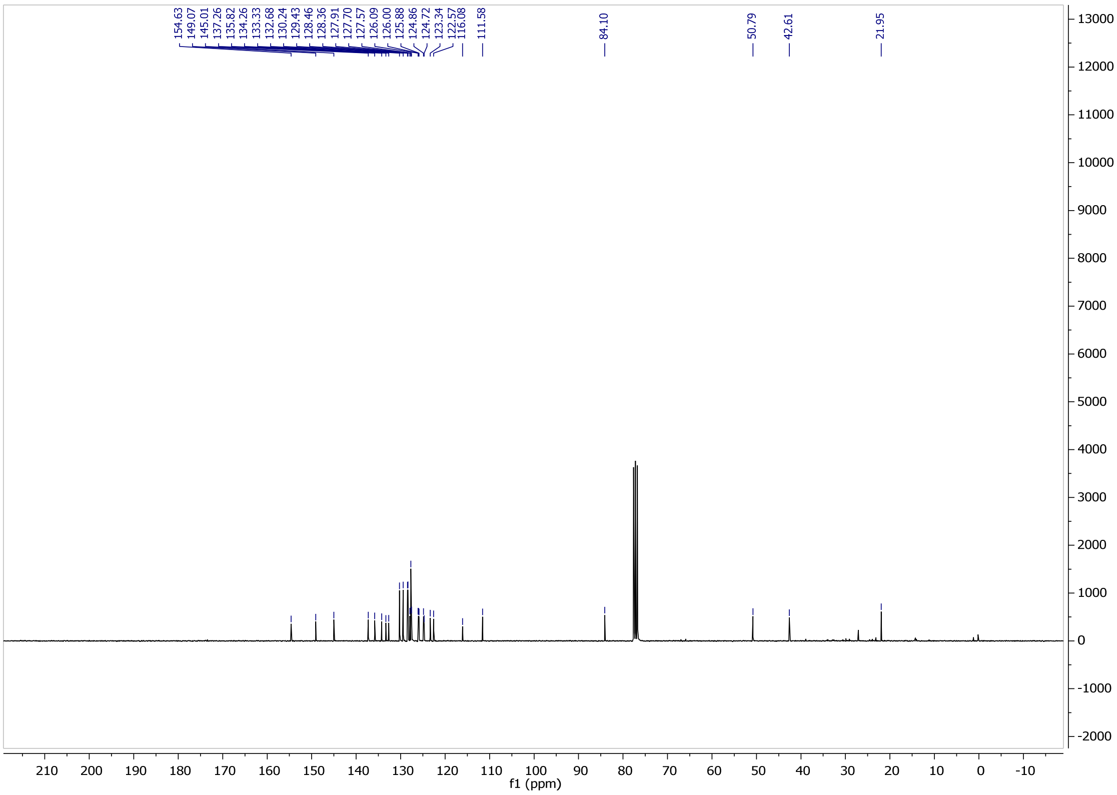

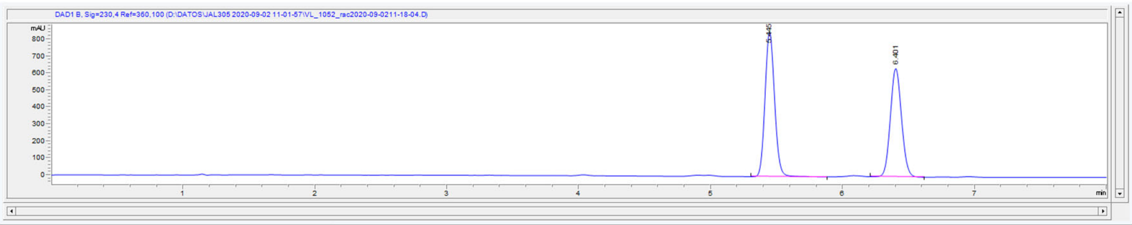

File Information

LC-File	VL_1052_rac2020-09-0211-18-04.D
File Path	D:\DATOS\JAL305 2020-09-02 11-01-57
Date	02-Sep-20, 11:21:28
Sample	VL_1052_rac
Sample Info	VL_1052
Barcode	
Operator	SYSTEM
Method	IC-gradiente5_40_MeOH_MS.M
Reference	D:\DATOS\JAL305 2020-09-02 11-01-57\no Sample Name2020-09-(

#	Time	Type	Area	Height	Width	Area%	Symmetry
1	5.445	BB	4310.1	855	0.0788	53.388	0.859
2	6.401	BB	3763	640.1	0.0926	46.612	0.905

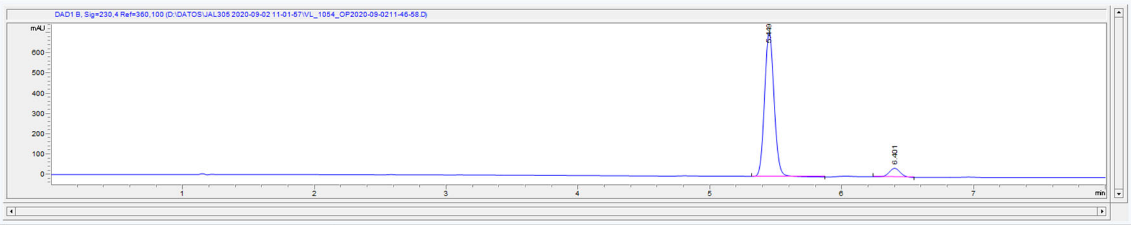

File Information

LC-File	VL_1054_OP2020-09-0211-46-58.D
File Path	D:\DATOS\JAL305 2020-09-02 11-01-57
Date	02-Sep-20, 11:50:19
Sample	VL_1054_OP
Sample Info	VL_1053
Barcode	
Operator	SYSTEM
Method	IC-gradiente5_40_MeOH_MS.M
Reference	D:\DATOS\JAL305 2020-09-02 11-01-57\no Sample Name2020-09-(

#	Time	Type	Area	Height	Width	Area%	Symmetry
1	5.449	BB	3583.2	717.6	0.0783	93.415	0.875
2	6.401	BB	252.6	43.6	0.0916	6.585	0.938

(*2R,3S,4S*)-8-Bromo-3,4-diphenyl-1-tosyl-1,2,3,4-tetrahydrobenzofuro[3,2-*b*]pyridin-2-ol (5l)

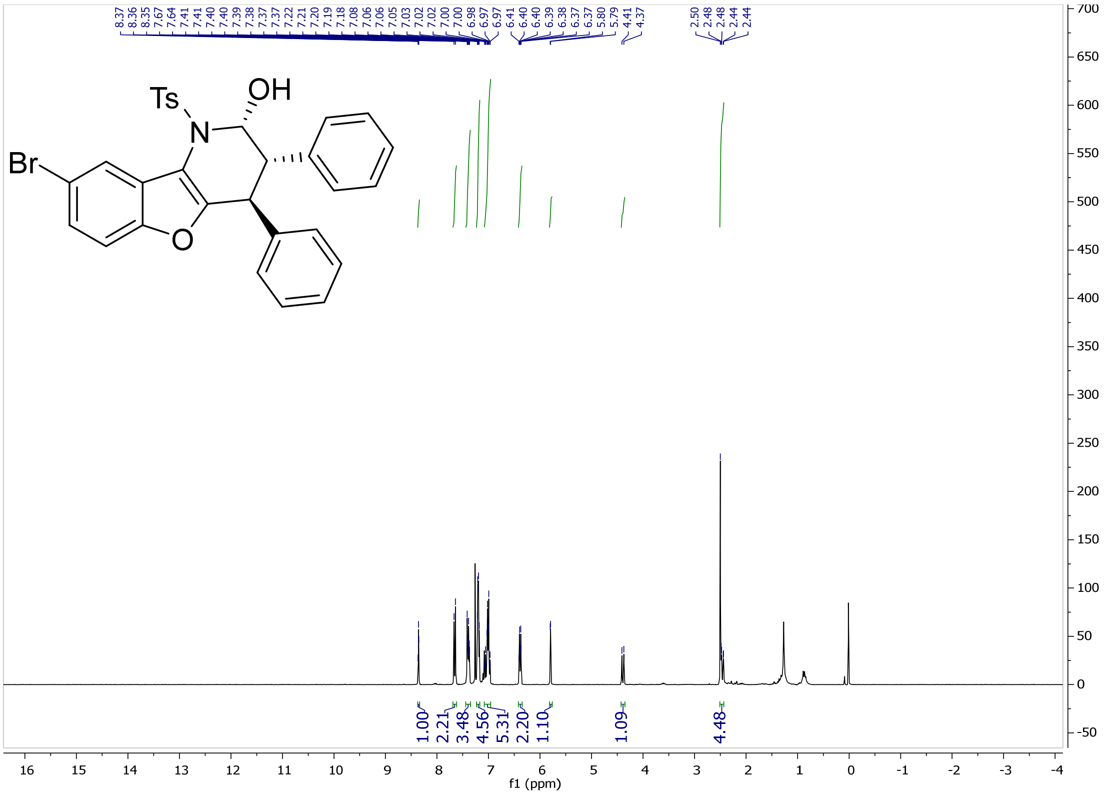

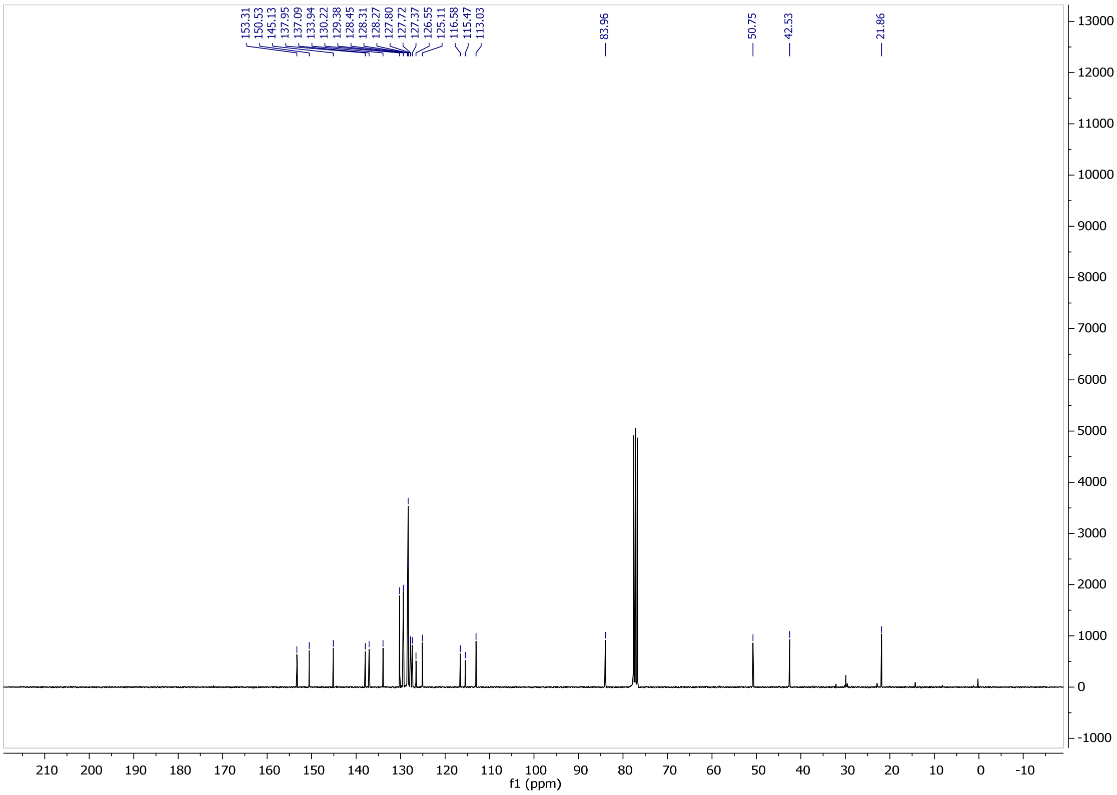

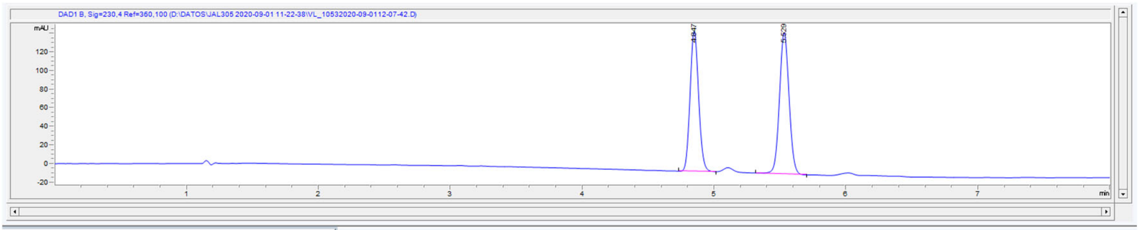

File Information

LC-File	VL_10532020-09-0112-07-42.D
File Path	D:\DATOS\JAL305 2020-09-01 11-22-38
Date	01-Sep-20, 12:11:14
Sample	VL_1053
Sample Info	VL_1053
Barcode	
Operator	SYSTEM
Method	IC-gradiente5_40_MeOH_MS.M
Reference	D:\DATOS\JAL305 2020-09-01 11-22-38\no Sample Name2020-09-(

#	Time	Type	Area	Height	Width	Area%	Symmetry
1	4.847	BB	675.8	150.4	0.0704	46.944	0.879
2	5.529	BB	763.8	151.6	0.0768	53.056	0.941

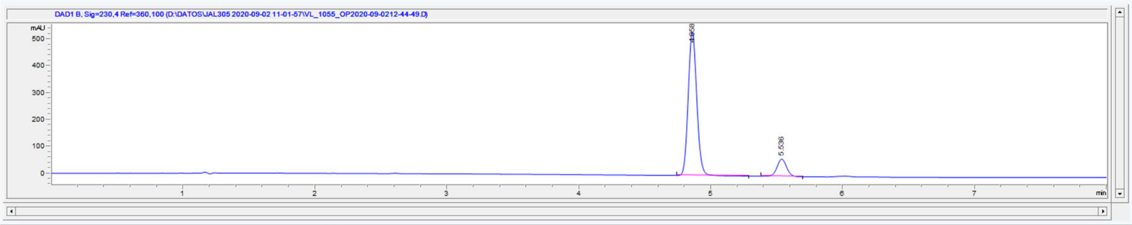

File Information

LC-File	VL_1055_OP2020-09-0212-44-49.D
File Path	D:\DATOS\JAL305 2020-09-02 11-01-57
Date	02-Sep-20, 12:48:17
Sample	VL_1055_OP
Sample Info	VL_1053
Barcode	
Operator	SYSTEM
Method	IC-gradiente5_40_MeOH_MS.M
Reference	D:\DATOS\JAL305 2020-09-02 11-01-57\no Sample Name2020-09-(

#	Time	Type	Area	Height	Width	Area%	Symmetry
1	4.858	BB	2423	538.8	0.0685	88.195	0.866
2	5.536	BB	324.3	64.8	0.0764	11.805	0.947

(*2R,3S,4S*)-7-Bromo-3,4-diphenyl-1-tosyl-1,2,3,4-tetrahydrobenzofuro[3,2-*b*]pyridin-2-ol (5m)

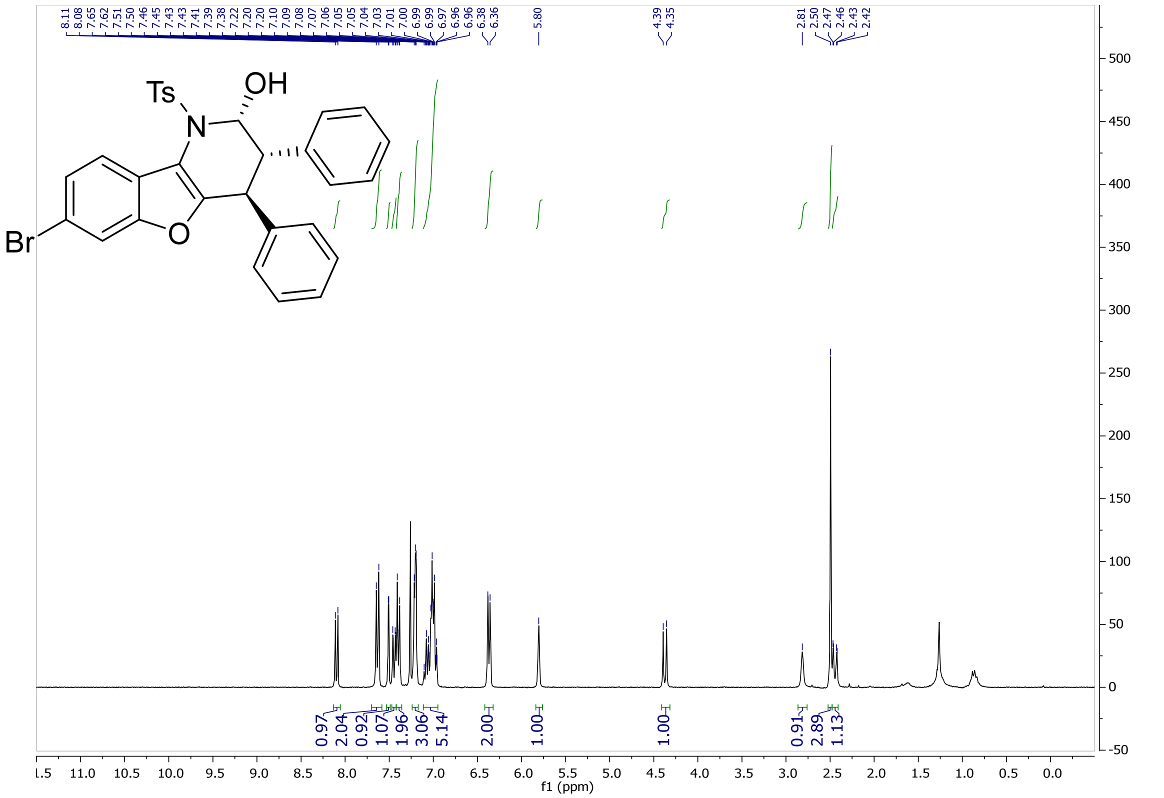

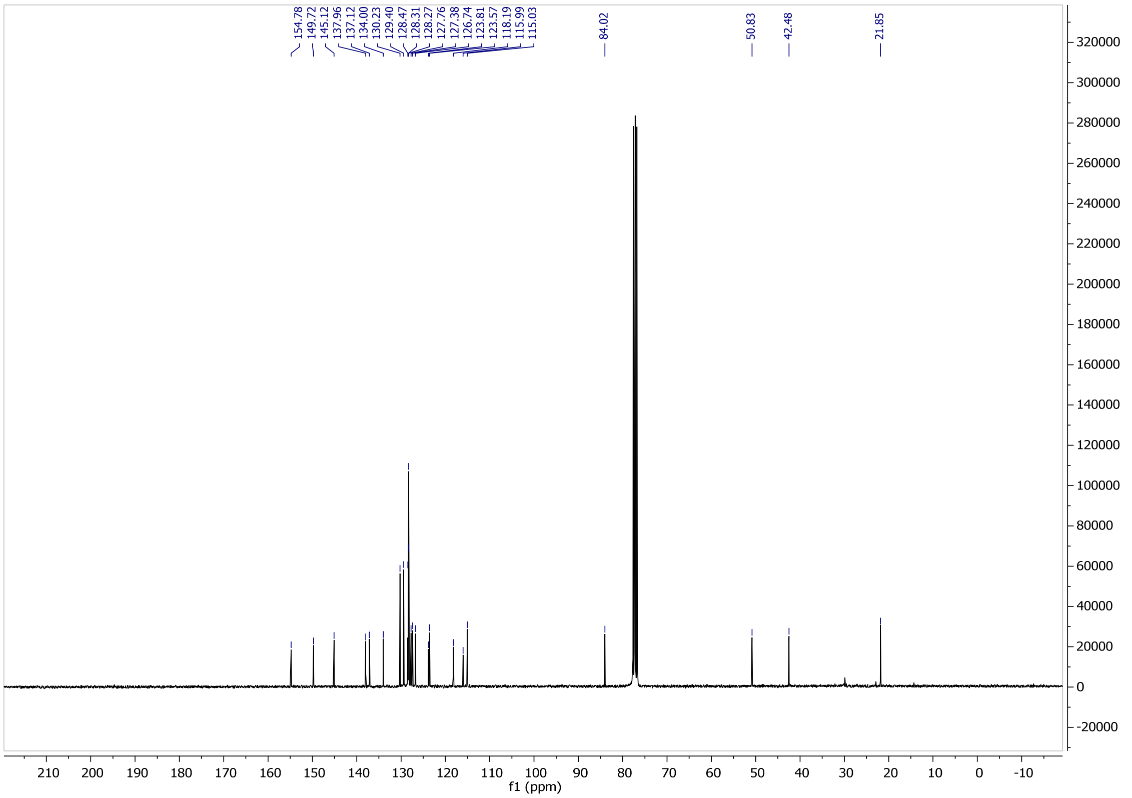

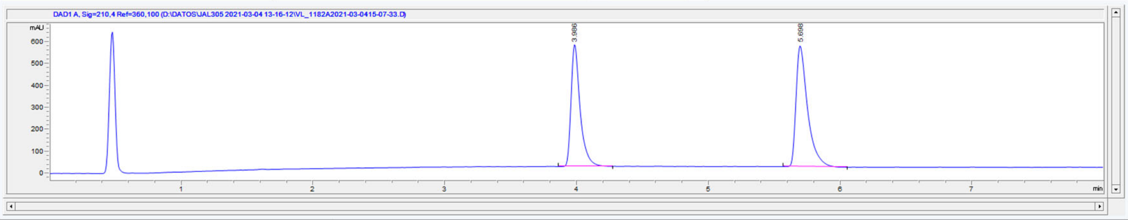

File Information

LC-File	VL_1182A2021-03-0415-07-33.D
File Path	D:\DATOS\JAL305 2021-03-04 13-16-12
Date	04-Mar-21, 15:10:57
Sample	VL_1182A
Sample Info	VL_1182A
Barcode	
Operator	SYSTEM
Method	IB-gradiente5_30_MeOH_MS.M
Reference	D:\DATOS\JAL305 2021-03-04 13-16-12\no Sample Name2021-03-(
Analysis Time	7.993 min

#	Time	Type	Area	Height	Width	Area%	Symmetry
1	3.986	BB	2673.9	560.6	0.0717	44.096	0.597
2	5.698	BB	3389.8	554.9	0.0913	55.904	0.5

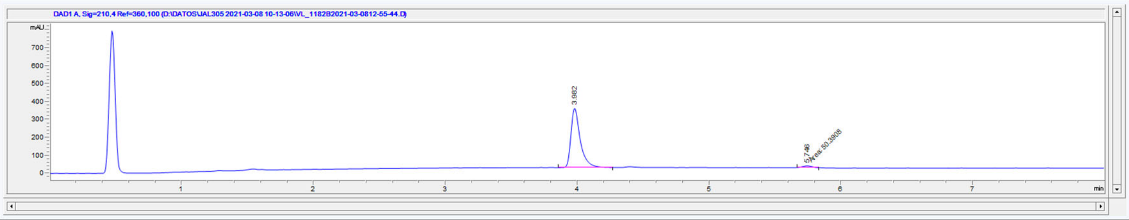

File Information

LC-File	VL_1182B2021-03-0812-55-44.D
File Path	D:\DATOS\JAL305 2021-03-08 10-13-06
Date	08-Mar-21, 12:59:09
Sample	VL_1182B
Sample Info	VL_1182B
Barcode	
Operator	SYSTEM
Method	IB-gradiente5_30_MeOH_MS.M
Reference	D:\DATOS\JAL305 2021-03-08 10-13-06\no Sample Name2021-03-(
Analysis Time	7.993 min

#	Time	Type	Area	Height	Width	Area%	Symmetry
1	3.982	BB	1626.1	331.3	0.0733	96.994	0.6
2	5.746	MM	50.4	10.3	0.0819	3.006	0.971

(2R,3R,4S)-4-Phenyl-1-tosyl-3-vinyl-1,2,3,4-tetrahydrobenzofuro[3,2-*b*]pyridin-2-yl acetate (6a)

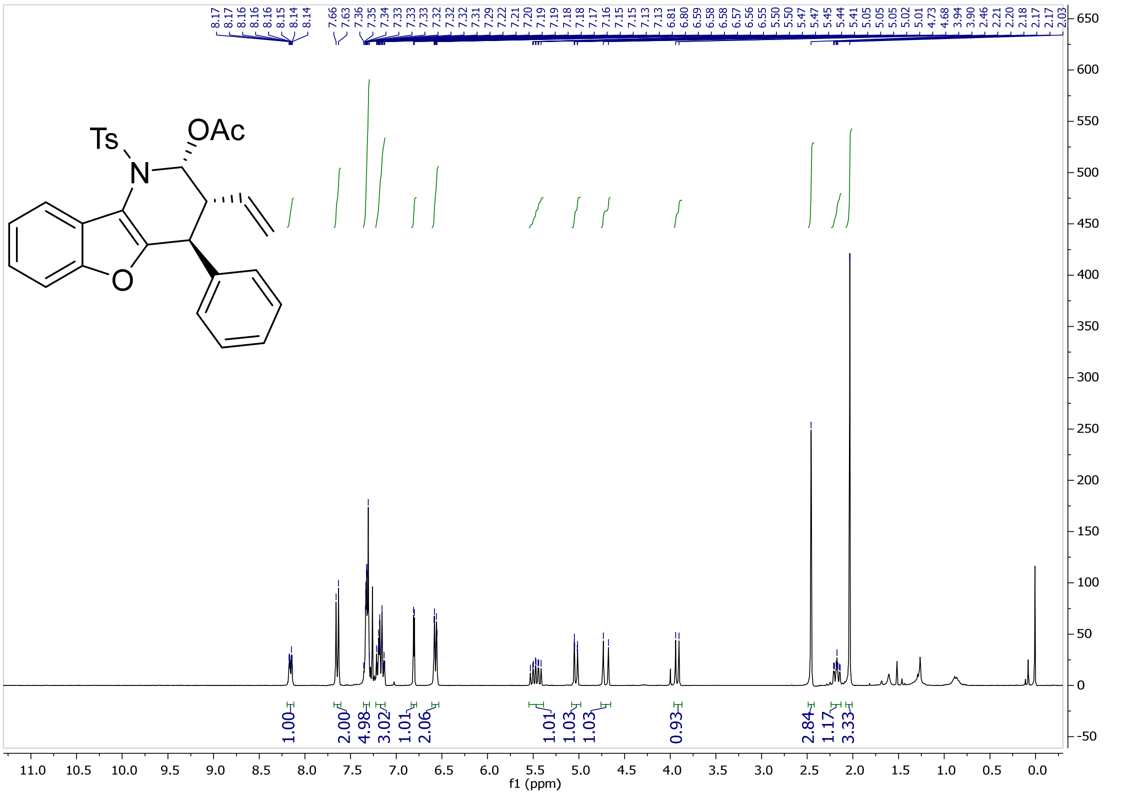

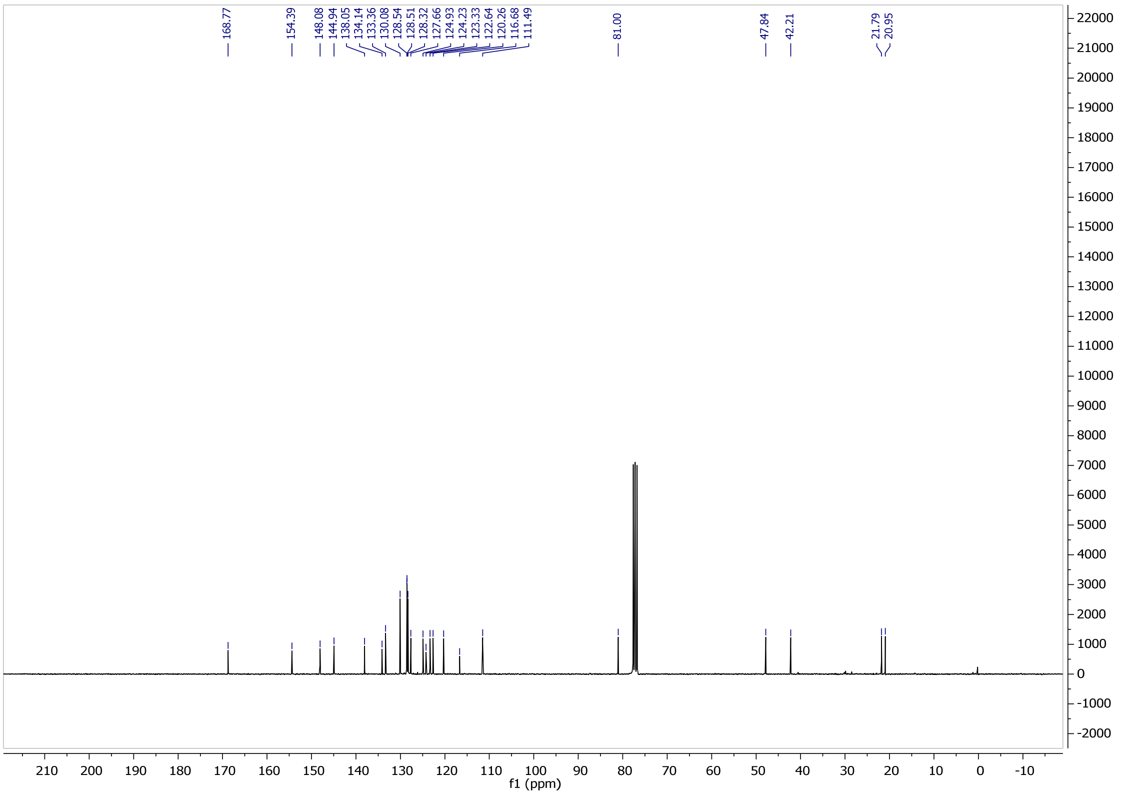

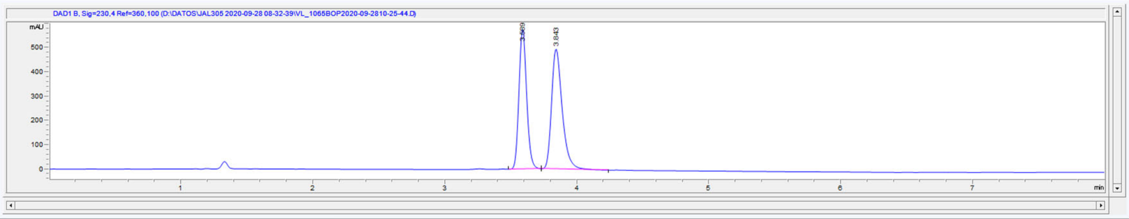

File Information

LC-File	VL_1065BOP2020-09-2810-25-44.D
File Path	D:\DATOS\JAL305 2020-09-28 08-32-39
Date	28-Sep-20, 10:28:10
Sample	VL_1065BOP
Sample Info	VL_1065BOP
Barcode	
Operator	SYSTEM
Method	IA-gradiente5_40_MeOH_NO_MS.M
Reference	D:\DATOS\JAL305 2020-09-28 08-32-39\no Sample Name2020-09-:

#	Time	Type	Area	Height	Width	Area%	Symmetry
1	3.589	BB	2322.8	574.3	0.0632	45.711	0.843
2	3.843	BB	2758.6	492.4	0.0854	54.289	0.679

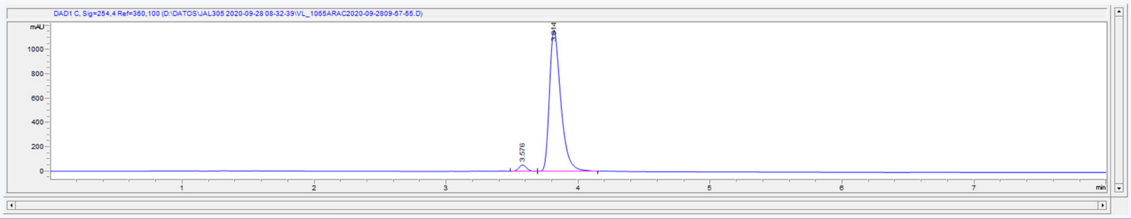

File Information

LC-File	VL_1065ARAC2020-09-2809-57-55.D
File Path	D:\DATOS\JAL305 2020-09-28 08-32-39
Date	28-Sep-20, 10:00:13
Sample	VL_1065ARAC
Sample Info	VL_1065ARAC
Barcode	
Operator	SYSTEM
Method	IA-gradiente5_40_MeOH_NO_MS.M
Reference	D:\DATOS\JAL305 2020-09-28 08-32-39\no Sample Name2020-09-:

#	Time	Type	Area	Height	Width	Area%	Symmetry
1	3.576	BB	207.5	51.5	0.063	2.918	0.867
2	3.814	BB	6903.5	1160.8	0.0914	97.082	0.617

(*2R*,*3R*,*4S*)-4-Phenyl-3-((*E*)-styryl)-1-tosyl-1,2,3,4-tetrahydrobenzofuro[3,2-*b*]pyridin-2-yl acetate (7a)

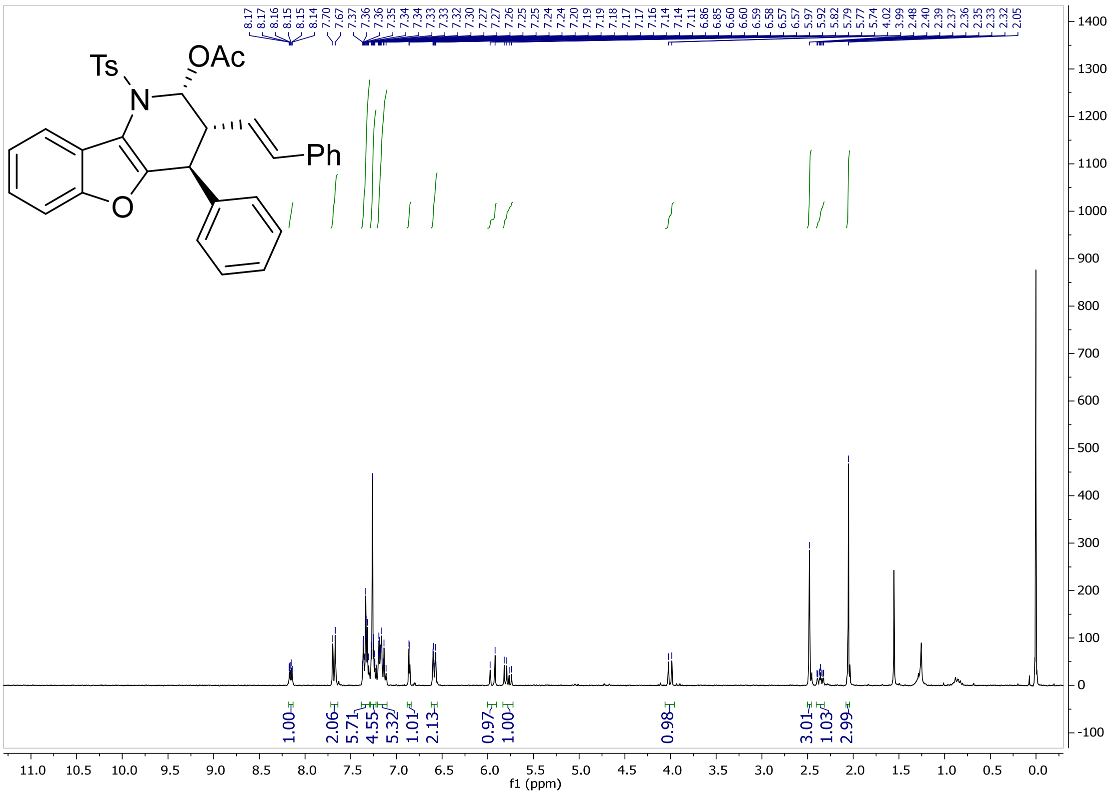

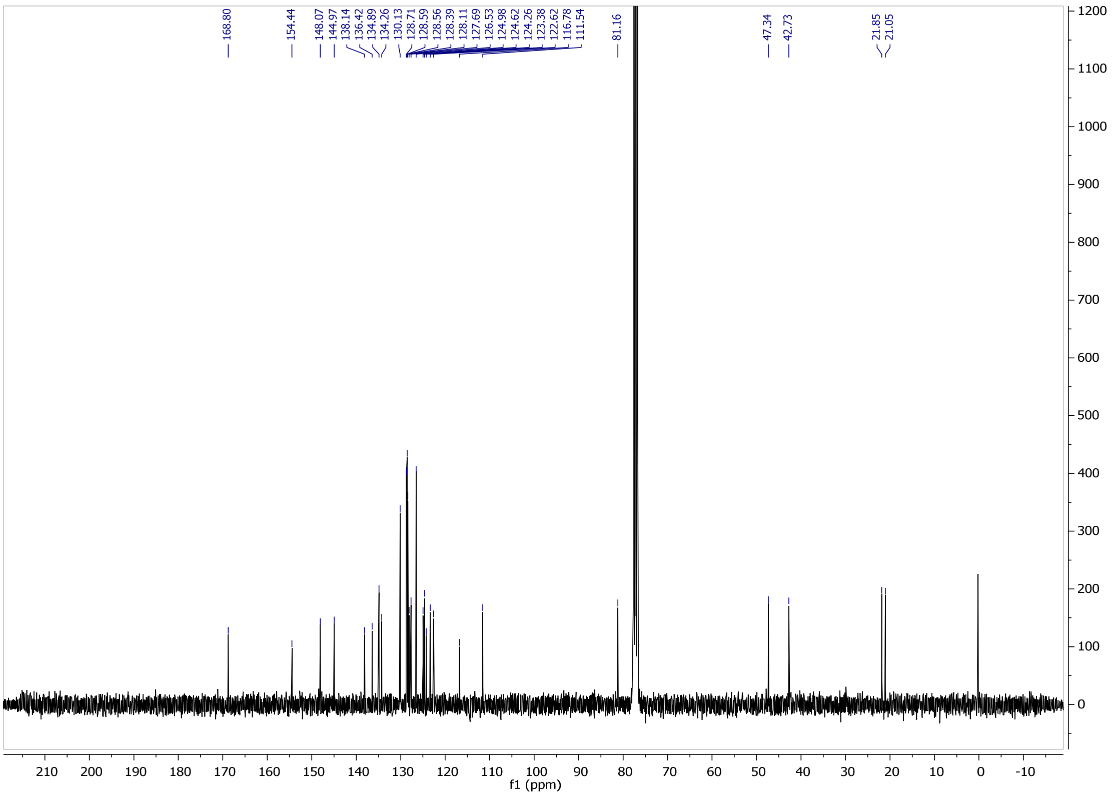

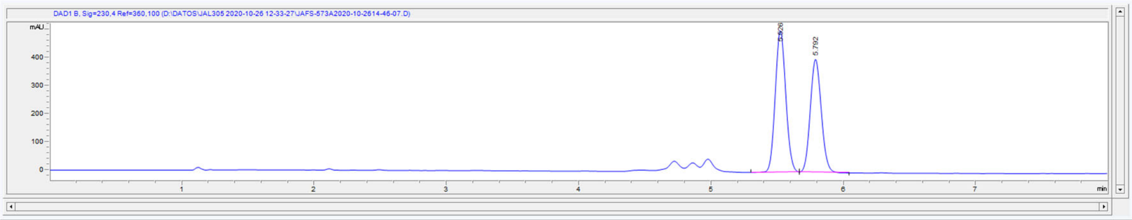

File Information

LC-File	JAFS-573A2020-10-2614-46-07.D
File Path	D:\DATOS\JAL305 2020-10-26 12-33-27
Date	26-Oct-20, 14:49:38
Sample	JAFS-573A
Sample Info	JAFS-573A
Barcode	
Operator	SYSTEM
Method	IC-gradiente5_40_MeOH_MS.M
Reference	D:\DATOS\JAL305 2020-10-26 12-33-27\no Sample Name2020-10-[illegible]

#	Time	Type	Area	Height	Width	Area%	Symmetry
1	5.526	BB	2775.8	504.2	0.0862	54.489	0.907
2	5.792	BB	2318.5	402.1	0.0893	45.511	0.883

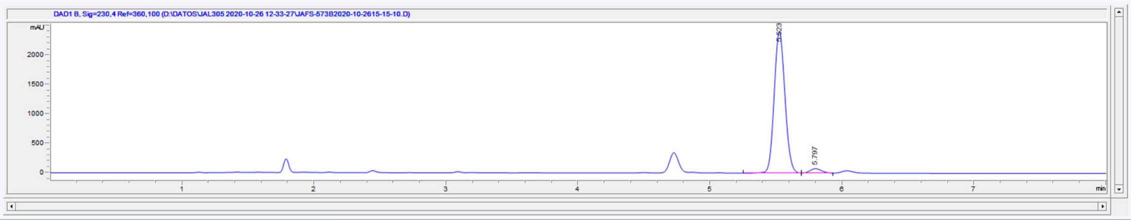

File Information

LC-File	JAFS-573B2020-10-2615-15-10.D
File Path	D:\DATOS\JAL305 2020-10-26 12-33-27
Date	26-Oct-20, 15:18:39
Sample	JAFS-573B
Sample Info	JAFS-573B
Barcode	
Operator	SYSTEM
Method	IC-gradiente5_40_MeOH_MS.M
Reference	D:\DATOS\JAL305 2020-10-26 12-33-27\no Sample Name2020-10-[illegible]

#	Time	Type	Area	Height	Width	Area%	Symmetry
1	5.523	BB	13607.1	2415.5	0.0877	96.808	0.884
2	5.797	BB	448.6	75.6	0.0913	3.192	0.821

(*R*)-3,4-Diphenyl-1-tosyl-1,4-dihydrobenzofuro[3,2-*b*]pyridine (8a)

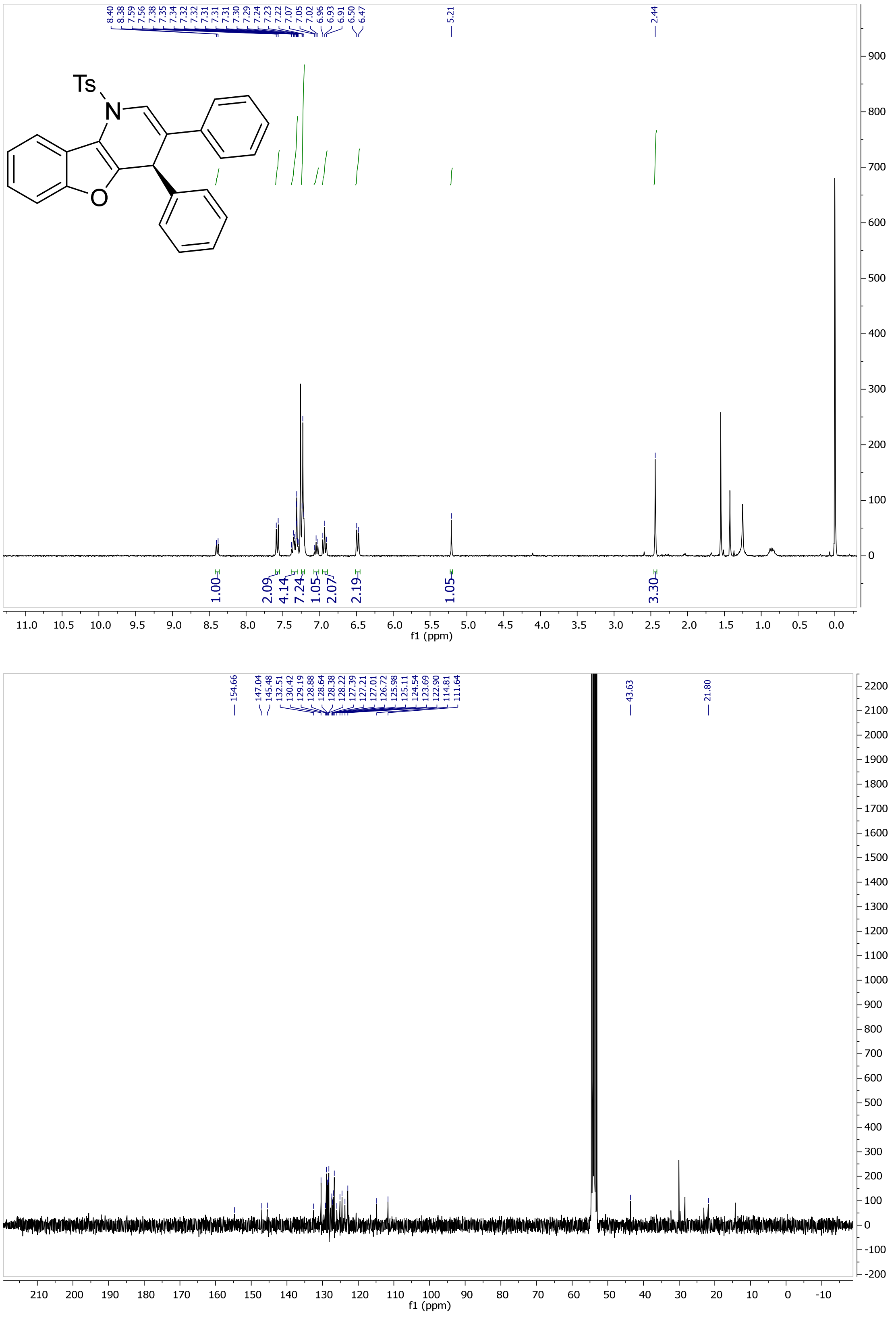

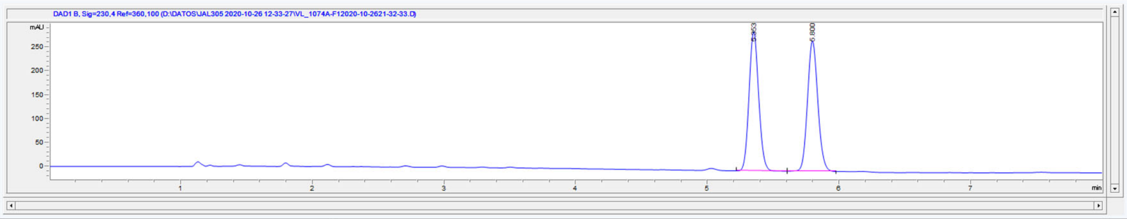

File Information

LC-File	VL_1074A-F12020-10-2621-32-33.D
File Path	D:\DATOS\JAL305 2020-10-26 12-33-27
Date	26-Oct-20, 21:36:01
Sample	VL_1074A-F1
Sample Info	VL_1074A-F1
Barcode	
Operator	SYSTEM
Method	IC-gradiente5_40_MeOH_MS.M
Reference	D:\DATOS\JAL305 2020-10-26 12-33-27\no Sample Name2020-10-2

#	Time	Type	Area	Height	Width	Area%	Symmetry
1	5.353	BB	1490.9	294.1	0.0772	49.917	0.887
2	5.8	BB	1495.9	275.3	0.0854	50.083	0.885

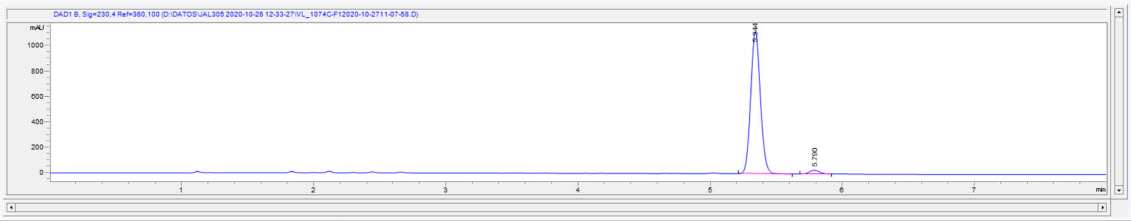

File Information

LC-File	VL_1074C-F12020-10-2711-07-58.D
File Path	D:\DATOS\JAL305 2020-10-26 12-33-27
Date	27-Oct-20, 11:11:19
Sample	VL_1074C-F1
Sample Info	VL_1074C-F1
Barcode	
Operator	SYSTEM
Method	IC-gradiente5_40_MeOH_MS.M
Reference	D:\DATOS\JAL305 2020-10-26 12-33-27\no Sample Name2020-10-2

#	Time	Type	Area	Height	Width	Area%	Symmetry
1	5.344	BB	5693.8	1122.1	0.0792	97.401	0.894
2	5.79	BB	151.9	29.2	0.0808	2.599	0.922

10. Single Crystal X-Ray Structure of compound 6a

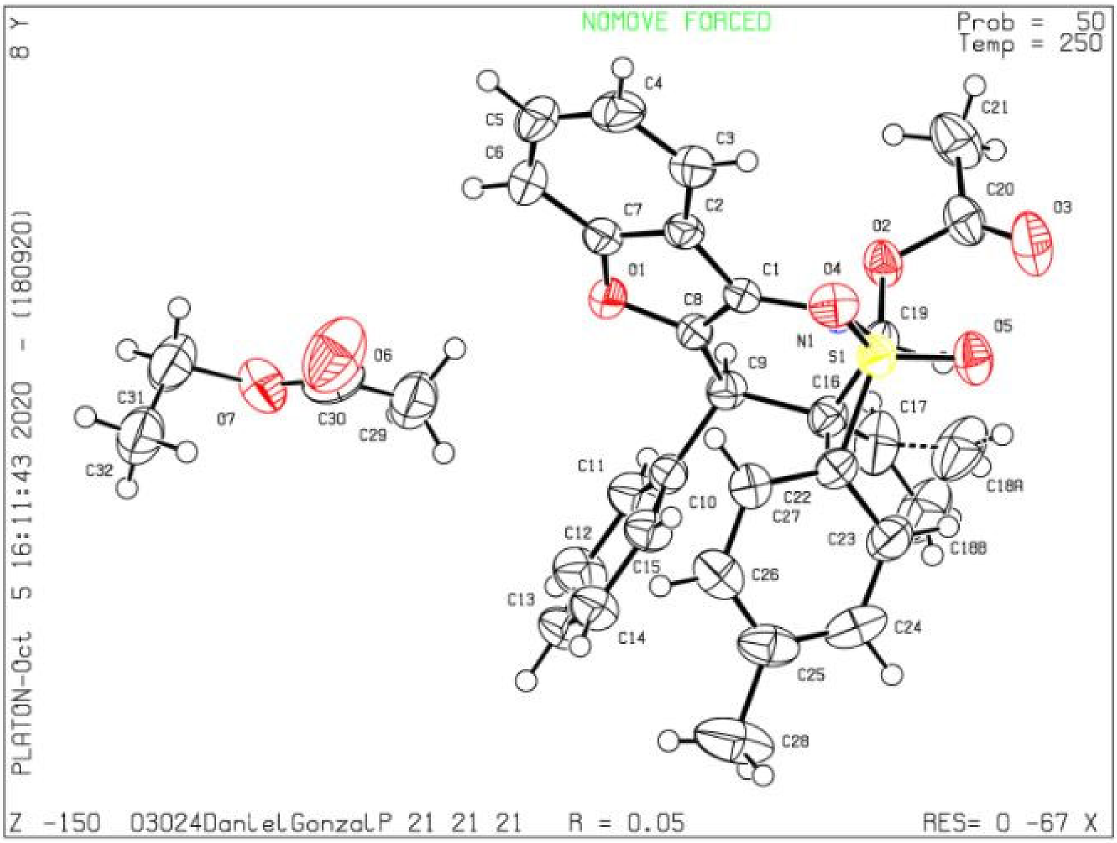

A clear colorless needle-like specimen of $C_{31}H_{31}NO_{6.50}S$, approximate dimensions 0.094 mm x 0.174 mm x 0.298 mm, was used for the X-ray crystallographic analysis. The X-ray intensity data were measured.

The total exposure time was 12.14 hours. The frames were integrated with the Bruker SAINT software package using a narrow-frame algorithm. The integration of the data using an orthorhombic unit cell yielded a total of 36993 reflections to a maximum θ angle of 25.34° (0.83 Å resolution), of which 5339 were independent (average redundancy 6.929, completeness = 99.5%, R_{int} = 4.70%, R_{sig} = 2.71%) and 4722 (88.44%) were greater than $2\sigma(F^2)$. The final cell constants of a = 10.5571(2) Å, b = 12.9667(4) Å, c = 21.3679(6) Å, volume = 2925.07(13) Å^3, are based upon the refinement of the XYZ-centroids of 8114 reflections above 20 σ(I) with 4.940° < 2θ < 43.55°. Data were corrected for absorption effects using the multi-scan method (SADABS). The ratio of minimum to maximum apparent transmission was 0.895. The calculated minimum and maximum transmission coefficients (based on crystal size) are 0.9550 and 0.9860.

The structure was solved and refined using the Bruker SHELXTL Software Package, using the space group P 21 21 21, with Z = 4 for the formula unit, $C_{31}H_{31}NO_{6.50}S$. The final anisotropic full-

matrix least-squares refinement on F^2 with 372 variables converged at R1 = 4.83%, for the observed data and wR2 = 14.44% for all data. The goodness-of-fit was 0.999. The largest peak in the final difference electron density synthesis was 0.619 $e^-/Å^3$ and the largest hole was -0.250 $e^-/Å^3$ with an RMS deviation of 0.061 $e^-/Å^3$. On the basis of the final model, the calculated density was 1.257 g/cm^3 and F(000), 1168 e^-.

Table S2. Sample and crystal data.

**Identification code**	03024DanielGonzalez	
**Chemical formula**	$C_{31}H_{31}NO_{6.50}S$	
**Formula weight**	553.63 g/mol	
**Temperature**	250(2) K	
**Wavelength**	0.71073 Å	
**Crystal size**	0.094 x 0.174 x 0.298 mm	
**Crystal habit**	clear colourless needle	
**Crystal system**	orthorhombic	
**Space group**	P 21 21 21	
**Unit cell dimensions**	a = 10.5571(2) Å	α = 90°
	b = 12.9667(4) Å	β = 90°
	c = 21.3679(6) Å	γ = 90°
**Volume**	2925.07(13) Å^3	
**Z**	4	
**Density (calculated)**	1.257 g/cm^3	
**Absorption coefficient**	0.156 mm^{-1}	
**F(000)**	1168	

Table S3. Data collection and structure refinement.

**Theta range for data collection**	3.13 to 25.34°	
**Index ranges**	-12<=h<=12, -15<=k<=15, -25<=l<=25	
**Reflections collected**	36993	
**Independent reflections**	5339 [R(int) = 0.0470]	
**Coverage of independent reflections**	99.5%	
**Absorption correction**	multi-scan	
**Max. and min. transmission**	0.9860 and 0.9550	
**Structure solution technique**	direct methods	
**Structure solution program**	SHELXS-97 (Sheldrick 2008)	
**Refinement method**	Full-matrix least-squares on F^2	
**Refinement program**	SHELXL-2014/7 (Sheldrick, 2014)	
**Function minimized**	$\Sigma w(F_o^2 - F_c^2)^2$	
**Data / restraints / parameters**	5339 / 2 / 372	
**Goodness-of-fit on F^2**	0.999	
**Final R indices**	4722 data; $I>2\sigma(I)$	R1 = 0.0483, wR2 = 0.1353
	all data	R1 = 0.0567, wR2 = 0.1444
**Weighting scheme**	$w=1/[\sigma^2(F_o^2)+(0.0997P)^2+0.9066P]$ where $P=(F_o^2+2F_c^2)/3$	
**Absolute structure parameter**	0.1(0)	
**Largest diff. peak and hole**	0.619 and -0.250 eÅ^{-3}	
**R.M.S. deviation from mean**	0.061 eÅ^{-3}	

Table S4. Atomic coordinates and equivalent isotropic atomic displacement parameters (Å²).

U(eq) is defined as one third of the trace of the orthogonalized U_{ij} tensor.

	x/a	y/b	z/c	U(eq)
C1	0.4844(3)	0.4984(3)	0.76757(17)	0.0310(8)
C2	0.6072(4)	0.5229(3)	0.79305(18)	0.0345(8)
C3	0.6552(4)	0.5650(3)	0.8484(2)	0.0427(10)
C4	0.7854(5)	0.5664(4)	0.8562(2)	0.0528(12)
C5	0.8669(4)	0.5271(4)	0.8106(3)	0.0569(12)
C6	0.8211(4)	0.4856(4)	0.7561(2)	0.0500(11)
C7	0.6908(4)	0.4841(3)	0.74914(18)	0.0385(9)
C8	0.5021(3)	0.4479(3)	0.71345(17)	0.0333(8)
C9	0.4091(4)	0.3926(3)	0.67409(17)	0.0344(8)
C10	0.4168(4)	0.4208(3)	0.60532(18)	0.0349(8)
C11	0.4261(5)	0.3461(3)	0.5596(2)	0.0492(11)
C12	0.4347(5)	0.3737(5)	0.4967(2)	0.0588(13)
C13	0.4314(5)	0.4752(4)	0.4800(2)	0.0534(12)
C14	0.4230(5)	0.5498(4)	0.5240(2)	0.0529(11)
C15	0.4150(4)	0.5228(3)	0.58732(19)	0.0426(10)
C16	0.2744(4)	0.4149(3)	0.70081(18)	0.0364(9)
C17	0.1812(5)	0.3331(5)	0.6815(3)	0.0618(14)
C18A	0.0762(18)	0.311(2)	0.7037(14)	0.077(3)
C18B	0.0757(7)	0.3421(8)	0.6581(4)	0.077(3)
C19	0.2739(3)	0.4272(3)	0.77183(19)	0.0346(8)
C20	0.2454(5)	0.2947(3)	0.8478(2)	0.0438(10)
C21	0.2992(6)	0.1944(4)	0.8704(3)	0.0633(14)

	x/a	y/b	z/c	U(eq)
C22	0.2732(4)	0.6828(3)	0.73898(19)	0.0352(9)
C23	0.1546(4)	0.6742(4)	0.7119(2)	0.0505(11)
C24	0.1336(5)	0.7184(4)	0.6537(3)	0.0638(14)
C25	0.2303(6)	0.7699(4)	0.6218(2)	0.0569(13)
C26	0.3458(5)	0.7781(3)	0.6508(2)	0.0497(11)
C27	0.3694(4)	0.7351(3)	0.70862(19)	0.0383(9)
C28	0.2045(8)	0.8152(5)	0.5591(3)	0.092(2)
N1	0.3613(3)	0.5060(2)	0.79500(14)	0.0321(7)
O1	0.6282(2)	0.4382(2)	0.70004(13)	0.0400(7)
O2	0.3140(3)	0.3310(2)	0.79881(13)	0.0401(6)
O3	0.1567(4)	0.3373(3)	0.86909(19)	0.0682(10)
O4	0.4024(3)	0.6764(2)	0.84129(14)	0.0439(7)
O5	0.1848(3)	0.6054(2)	0.84167(14)	0.0479(7)
S1	0.30261(9)	0.62260(7)	0.81083(4)	0.0344(3)
C29	0.7684(7)	0.4006(8)	0.5514(4)	0.0805(18)
C30	0.8880(8)	0.4519(6)	0.5477(4)	0.067(2)
C31	0.0906(7)	0.4593(7)	0.4993(4)	0.072(2)
C32	0.0882(7)	0.5527(8)	0.4642(4)	0.0805(18)
O6	0.9191(6)	0.5241(6)	0.5821(3)	0.100(2)
O7	0.9593(5)	0.4156(4)	0.5076(2)	0.0581(12)

Table S5. Bond lengths (Å).

C1-C8	1.342(5)	C1-N1	1.429(5)
C1-C2	1.441(5)	C2-C7	1.383(6)
C2-C3	1.398(6)	C3-C4	1.384(7)
C3-H3	0.94	C4-C5	1.397(8)
C4-H4	0.94	C5-C6	1.370(7)
C5-H5	0.94	C6-C7	1.384(6)
C6-H6	0.94	C7-O1	1.376(5)
C8-O1	1.368(4)	C8-C9	1.478(5)
C9-C10	1.516(5)	C9-C16	1.560(5)
C9-H9	0.99	C10-C15	1.378(6)
C10-C11	1.379(6)	C11-C12	1.394(7)
C11-H11	0.94	C12-C13	1.364(8)
C12-H12	0.94	C13-C14	1.352(7)
C13-H13	0.94	C14-C15	1.400(6)
C14-H14	0.94	C15-H15	0.94
C16-C17	1.505(6)	C16-C19	1.526(5)
C16-H16	0.99	C17-C18B	1.226(9)
C17-C18A	1.24(2)	C17-H17A	0.94
C17-H17B	0.94	C18A-H18A	0.94
C18A-H18B	0.94	C18B-H18C	0.94
C18B-H18D	0.94	C19-O2	1.437(5)
C19-N1	1.464(5)	C19-H19	0.99
C20-O3	1.179(6)	C20-O2	1.358(5)
C20-C21	1.499(7)	C21-H21A	0.97
C21-H21B	0.97	C21-H21C	0.97
C22-C27	1.382(6)	C22-C23	1.384(6)

C22-S1	1.750(4)	C23-C24	1.388(7)
C23-H23	0.94	C24-C25	1.397(8)
C24-H24	0.94	C25-C26	1.372(7)
C25-C28	1.488(7)	C26-C27	1.379(6)
C26-H26	0.94	C27-H27	0.94
C28-H28A	0.97	C28-H28B	0.97
C28-H28C	0.97	N1-S1	1.668(3)
O4-S1	1.421(3)	O5-S1	1.425(3)
C29-C30	1.430(11)	C29-H29A	0.97
C29-H29B	0.97	C29-H29C	0.97
C30-O7	1.234(9)	C30-O6	1.235(10)
C31-C32	1.425(12)	C31-O7	1.508(9)
C31-H31A	0.98	C31-H31B	0.98
C32-H32A	0.97	C32-H32B	0.97
C32-H32C	0.97		

Table S6. Bond angles (°).

C8-C1-N1	120.9(3)	C8-C1-C2	108.0(3)
N1-C1-C2	130.4(3)	C7-C2-C3	119.0(4)
C7-C2-C1	103.7(3)	C3-C2-C1	137.1(4)
C4-C3-C2	117.8(4)	C4-C3-H3	121.1
C2-C3-H3	121.1	C3-C4-C5	121.6(4)
C3-C4-H4	119.2	C5-C4-H4	119.2
C6-C5-C4	121.2(4)	C6-C5-H5	119.4
C4-C5-H5	119.4	C5-C6-C7	116.6(4)
C5-C6-H6	121.7	C7-C6-H6	121.7
O1-C7-C2	111.6(3)	O1-C7-C6	124.5(4)
C2-C7-C6	123.8(4)	C1-C8-O1	111.1(3)
C1-C8-C9	129.4(3)	O1-C8-C9	118.8(3)
C8-C9-C10	113.5(3)	C8-C9-C16	107.9(3)
C10-C9-C16	111.0(3)	C8-C9-H9	108.1
C10-C9-H9	108.1	C16-C9-H9	108.1
C15-C10-C11	118.5(4)	C15-C10-C9	120.1(4)
C11-C10-C9	121.4(4)	C10-C11-C12	120.5(4)
C10-C11-H11	119.7	C12-C11-H11	119.7
C13-C12-C11	119.9(4)	C13-C12-H12	120.1
C11-C12-H12	120.1	C14-C13-C12	120.6(4)
C14-C13-H13	119.7	C12-C13-H13	119.7
C13-C14-C15	119.9(5)	C13-C14-H14	120.1
C15-C14-H14	120.1	C10-C15-C14	120.6(4)
C10-C15-H15	119.7	C14-C15-H15	119.7
C17-C16-C19	110.2(3)	C17-C16-C9	111.4(3)
C19-C16-C9	112.7(3)	C17-C16-H16	107.4

C19-C16-H16	107.4	C9-C16-H16	107.4
C18B-C17-C16	129.7(7)	C18A-C17-C16	130.0(13)
C18A-C17-H17A	115.0	C16-C17-H17A	115.0
C18B-C17-H17B	115.2	C16-C17-H17B	115.2
C17-C18A-H18A	120.0	C17-C18A-H18B	120.0
H18A-C18A-H18B	120.0	C17-C18B-H18C	120.0
C17-C18B-H18D	120.0	H18C-C18B-H18D	120.0
O2-C19-N1	106.5(3)	O2-C19-C16	107.9(3)
N1-C19-C16	114.0(3)	O2-C19-H19	109.4
N1-C19-H19	109.4	C16-C19-H19	109.4
O3-C20-O2	124.0(4)	O3-C20-C21	125.7(4)
O2-C20-C21	110.3(4)	C20-C21-H21A	109.5
C20-C21-H21B	109.5	H21A-C21-H21B	109.5
C20-C21-H21C	109.5	H21A-C21-H21C	109.5
H21B-C21-H21C	109.5	C27-C22-C23	120.6(4)
C27-C22-S1	120.0(3)	C23-C22-S1	119.4(3)
C22-C23-C24	119.0(5)	C22-C23-H23	120.5
C24-C23-H23	120.5	C23-C24-C25	121.2(5)
C23-C24-H24	119.4	C25-C24-H24	119.4
C26-C25-C24	117.9(4)	C26-C25-C28	122.6(6)
C24-C25-C28	119.6(6)	C25-C26-C27	122.2(5)
C25-C26-H26	118.9	C27-C26-H26	118.9
C26-C27-C22	119.1(4)	C26-C27-H27	120.4
C22-C27-H27	120.4	C25-C28-H28A	109.5
C25-C28-H28B	109.5	H28A-C28-H28B	109.5
C25-C28-H28C	109.5	H28A-C28-H28C	109.5

H28B-C28-H28C	109.5	C1-N1-C19	112.7(3)
C1-N1-S1	118.9(2)	C19-N1-S1	117.9(2)
C8-O1-C7	105.6(3)	C20-O2-C19	116.9(3)
O4-S1-O5	120.81(19)	O4-S1-N1	105.20(17)
O5-S1-N1	106.01(17)	O4-S1-C22	108.32(18)
O5-S1-C22	108.71(19)	N1-S1-C22	106.99(16)
C30-C29-H29A	109.5	C30-C29-H29B	109.5
H29A-C29-H29B	109.5	C30-C29-H29C	109.5
H29A-C29-H29C	109.5	H29B-C29-H29C	109.5
O7-C30-O6	122.7(8)	O7-C30-C29	113.6(7)
O6-C30-C29	123.7(8)	C32-C31-O7	111.4(6)
C32-C31-H31A	109.4	O7-C31-H31A	109.4
C32-C31-H31B	109.4	O7-C31-H31B	109.4
H31A-C31-H31B	108.0	C31-C32-H32A	109.5
C31-C32-H32B	109.5	H32A-C32-H32B	109.5
C31-C32-H32C	109.5	H32A-C32-H32C	109.5
H32B-C32-H32C	109.5	C30-O7-C31	119.9(6)

Table S7. Torsion angles (°).

C8-C1-C2-C7	0.9(4)	N1-C1-C2-C7	170.5(4)
C8-C1-C2-C3	-173.3(4)	N1-C1-C2-C3	-3.7(7)
C7-C2-C3-C4	0.5(6)	C1-C2-C3-C4	174.0(4)

C2-C3-C4-C5	-0.1(7)	C3-C4-C5-C6	0.1(8)
C4-C5-C6-C7	-0.5(7)	C3-C2-C7-O1	175.1(3)
C1-C2-C7-O1	-0.4(4)	C3-C2-C7-C6	-1.0(6)
C1-C2-C7-C6	-176.4(4)	C5-C6-C7-O1	-174.6(4)
C5-C6-C7-C2	1.0(7)	N1-C1-C8-O1	-171.9(3)
C2-C1-C8-O1	-1.1(4)	N1-C1-C8-C9	-1.0(6)
C2-C1-C8-C9	169.8(4)	C1-C8-C9-C10	131.7(4)
O1-C8-C9-C10	-58.0(5)	C1-C8-C9-C16	8.2(5)
O1-C8-C9-C16	178.5(3)	C8-C9-C10-C15	-51.1(5)
C16-C9-C10-C15	70.6(5)	C8-C9-C10-C11	128.6(4)
C16-C9-C10-C11	-109.6(4)	C15-C10-C11-C12	0.6(7)
C9-C10-C11-C12	-179.2(4)	C10-C11-C12-C13	-1.3(8)
C11-C12-C13-C14	1.7(8)	C12-C13-C14-C15	-1.3(8)
C11-C10-C15-C14	-0.2(7)	C9-C10-C15-C14	179.5(4)
C13-C14-C15-C10	0.6(7)	C8-C9-C16-C17	-157.8(4)
C10-C9-C16-C17	77.2(5)	C8-C9-C16-C19	-33.4(4)
C10-C9-C16-C19	-158.4(3)	C19-C16-C17-C18B	103.8(8)
C9-C16-C17-C18B	-130.3(7)	C19-C16-C17-C18A	36.(2)
C9-C16-C17-C18A	162.(2)	C17-C16-C19-O2	62.3(4)
C9-C16-C19-O2	-62.8(4)	C17-C16-C19-N1	-179.5(4)
C9-C16-C19-N1	55.4(4)	C27-C22-C23-C24	0.3(7)

S1-C22-C23-C24	-177.2(4)	C22-C23-C24-C25	0.9(7)
C23-C24-C25-C26	-1.9(8)	C23-C24-C25-C28	179.3(5)
C24-C25-C26-C27	1.9(7)	C28-C25-C26-C27	-179.4(5)
C25-C26-C27-C22	-0.7(6)	C23-C22-C27-C26	-0.4(6)
S1-C22-C27-C26	177.1(3)	C8-C1-N1-C19	20.1(5)
C2-C1-N1-C19	-148.4(4)	C8-C1-N1-S1	-124.0(3)
C2-C1-N1-S1	67.5(5)	O2-C19-N1-C1	72.0(4)
C16-C19-N1-C1	-46.9(4)	O2-C19-N1-S1	-143.5(2)
C16-C19-N1-S1	97.6(3)	C1-C8-O1-C7	0.9(4)
C9-C8-O1-C7	-171.1(3)	C2-C7-O1-C8	-0.3(4)
C6-C7-O1-C8	175.8(4)	O3-C20-O2-C19	-0.4(6)
C21-C20-O2-C19	-180.0(4)	N1-C19-O2-C20	102.8(4)
C16-C19-O2-C20	-134.3(3)	C1-N1-S1-O4	-46.7(3)
C19-N1-S1-O4	171.0(3)	C1-N1-S1-O5	-175.8(3)
C19-N1-S1-O5	41.9(3)	C1-N1-S1-C22	68.3(3)
C19-N1-S1-C22	-74.0(3)	C27-C22-S1-O4	27.8(4)
C23-C22-S1-O4	-154.6(3)	C27-C22-S1-O5	160.8(3)
C23-C22-S1-O5	-21.6(4)	C27-C22-S1-N1	-85.2(3)
C23-C22-S1-N1	92.4(4)	O6-C30-O7-C31	0.3(11)
C29-C30-O7-C31	-178.1(7)	C32-C31-O7-C30	-79.3(9)

Table S8. Anisotropic atomic displacement parameters (Å^2).

The anisotropic atomic displacement factor exponent takes the form: $-2\pi^2[h^2 a^{*2} U_{11} + ... + 2 h k a^* b^* U_{12}]$

	U_{11}	U_{22}	U_{33}	U_{23}	U_{13}	U_{12}
C1	0.0323(19)	0.0314(19)	0.0293(18)	0.0047(15)	-0.0002(15)	0.0006(15)
C2	0.0348(19)	0.0335(18)	0.0352(19)	0.0081(16)	-0.0030(16)	-0.0014(16)
C3	0.045(2)	0.039(2)	0.043(2)	0.0022(18)	-0.0055(19)	-0.0057(18)
C4	0.051(3)	0.055(3)	0.053(3)	0.007(2)	-0.020(2)	-0.012(2)
C5	0.031(2)	0.075(3)	0.065(3)	0.014(3)	-0.007(2)	-0.008(2)
C6	0.032(2)	0.066(3)	0.052(3)	0.007(2)	0.0039(19)	0.003(2)
C7	0.036(2)	0.045(2)	0.035(2)	0.0095(16)	-0.0017(17)	-0.0030(18)
C8	0.0313(18)	0.037(2)	0.0313(18)	0.0049(15)	0.0036(15)	0.0072(16)
C9	0.0358(19)	0.0328(19)	0.0347(19)	-0.0029(15)	-0.0006(16)	0.0051(16)
C10	0.0309(19)	0.041(2)	0.033(2)	-0.0024(16)	-0.0003(15)	0.0013(16)
C11	0.066(3)	0.042(2)	0.040(2)	-0.0075(18)	-0.002(2)	0.006(2)
C12	0.075(3)	0.067(3)	0.034(2)	-0.015(2)	0.000(2)	0.003(3)
C13	0.053(3)	0.077(3)	0.030(2)	0.005(2)	-0.002(2)	-0.002(3)
C14	0.056(3)	0.055(3)	0.048(3)	0.012(2)	-0.001(2)	-0.001(2)
C15	0.053(3)	0.039(2)	0.036(2)	-0.0034(17)	-0.0026(19)	0.003(2)
C16	0.033(2)	0.0393(19)	0.037(2)	-0.0036(16)	-0.0013(16)	0.0006(15)
C17	0.048(3)	0.074(3)	0.064(3)	-0.032(3)	0.009(2)	-0.015(2)
C18A	0.044(3)	0.103(6)	0.085(6)	-0.039(5)	-0.014(4)	-0.008(4)

	U_{11}	U_{22}	U_{33}	U_{23}	U_{13}	U_{12}
C18B	0.044(3)	0.103(6)	0.085(6)	-0.039(5)	-0.014(4)	-0.008(4)
C19	0.031(2)	0.0312(18)	0.041(2)	-0.0017(16)	0.0028(16)	-0.0009(15)
C20	0.051(2)	0.039(2)	0.041(2)	-0.0034(18)	0.008(2)	-0.014(2)
C21	0.083(4)	0.048(3)	0.059(3)	0.013(2)	0.016(3)	-0.006(3)
C22	0.036(2)	0.0274(17)	0.042(2)	-0.0028(16)	-0.0012(17)	0.0046(16)
C23	0.038(2)	0.054(3)	0.060(3)	0.005(2)	-0.005(2)	0.002(2)
C24	0.056(3)	0.063(3)	0.072(3)	-0.002(3)	-0.028(3)	0.014(3)
C25	0.083(4)	0.041(2)	0.046(3)	0.000(2)	-0.012(3)	0.017(2)
C26	0.071(3)	0.035(2)	0.044(2)	-0.0014(18)	0.008(2)	0.003(2)
C27	0.043(2)	0.0323(19)	0.040(2)	-0.0061(16)	0.0026(18)	-0.0001(17)
C28	0.148(7)	0.072(4)	0.055(3)	0.009(3)	-0.020(4)	0.033(4)
N1	0.0306(15)	0.0322(16)	0.0337(16)	-0.0022(13)	0.0045(13)	-0.0007(13)
O1	0.0309(13)	0.0525(16)	0.0366(15)	0.0008(12)	0.0029(12)	0.0048(12)
O2	0.0409(15)	0.0328(13)	0.0465(16)	0.0055(12)	0.0102(13)	-0.0027(12)
O3	0.065(2)	0.067(2)	0.073(2)	0.0051(19)	0.0335(19)	-0.0020(19)
O4	0.0508(17)	0.0381(15)	0.0429(15)	-0.0069(13)	-0.0063(13)	0.0005(13)
O5	0.0478(17)	0.0468(17)	0.0490(16)	-0.0044(14)	0.0184(14)	0.0033(14)
S1	0.0368(5)	0.0332(4)	0.0333(5)	-0.0033(4)	0.0039(4)	0.0014(4)
C29	0.047(3)	0.132(6)	0.063(3)	-0.007(3)	0.005(2)	0.010(3)
C30	0.067(5)	0.055(4)	0.080(5)	0.001(4)	-0.018(4)	0.012(4)

	U_{11}	U_{22}	U_{33}	U_{23}	U_{13}	U_{12}
C31	0.041(3)	0.085(5)	0.090(6)	0.006(5)	0.004(4)	0.008(4)
C32	0.047(3)	0.132(6)	0.063(3)	-0.007(3)	0.005(2)	0.010(3)
O6	0.073(4)	0.115(5)	0.111(5)	-0.048(4)	0.000(4)	0.014(4)
O7	0.067(3)	0.061(3)	0.046(2)	-0.002(2)	0.010(2)	-0.009(2)

Table S9. Hydrogen atomic coordinates and isotropic atomic displacement parameters ($Å^2$).

	x/a	y/b	z/c	U(eq)
H3	0.6009	0.5916	0.8794	0.051
H4	0.8197	0.5944	0.8930	0.063
H5	0.9549	0.5291	0.8174	0.068
H6	0.8755	0.4594	0.7251	0.06
H9	0.4256	0.3178	0.6781	0.041
H11	0.4267	0.2761	0.5709	0.059
H12	0.4428	0.3224	0.4658	0.071
H13	0.4350	0.4934	0.4374	0.064
H14	0.4224	0.6196	0.5121	0.063
H15	0.4084	0.5748	0.6178	0.051
H16	0.2453	0.4810	0.6826	0.044
H17A	0.2065	0.2926	0.6472	0.074
H17B	0.2078	0.2649	0.6883	0.074
H18A	0.0443	0.3479	0.7381	0.093
H18B	0.0284	0.2568	0.6862	0.093
H18C	0.0426	0.4081	0.6498	0.093
H18D	0.0278	0.2831	0.6484	0.093
H19	0.1870	0.4433	0.7862	0.041

	x/a	y/b	z/c	U(eq)
H21A	0.2368	0.1402	0.8653	0.095
H21B	0.3215	0.2005	0.9143	0.095
H21C	0.3744	0.1777	0.8463	0.095
H23	0.0891	0.6390	0.7327	0.061
H24	0.0529	0.7137	0.6354	0.077
H26	0.4112	0.8142	0.6305	0.06
H27	0.4497	0.7412	0.7272	0.046
H28A	0.2840	0.8313	0.5386	0.137
H28B	0.1551	0.8778	0.5640	0.137
H28C	0.1575	0.7661	0.5340	0.137
H29A	0.7560	0.3746	0.5936	0.097
H29B	0.7668	0.3437	0.5220	0.097
H29C	0.7012	0.4488	0.5413	0.097
H31A	1.1280	0.4726	0.5405	0.087
H31B	1.1439	0.4087	0.4778	0.087
H32A	1.0303	0.6010	0.4837	0.097
H32B	1.0602	0.5383	0.4219	0.097
H32C	1.1726	0.5823	0.4631	0.097

Table S10. Hydrogen bond distances (Å) and angles (°).

	Donor-H	Acceptor-H	Donor-Acceptor	Angle
C9-H9···O4	0.99	2.61	3.454(5)	142.8
C19-H19···O5	0.99	2.41	2.907(5)	110.1
C21-H21A···O6	0.97	2.50	3.349(8)	146.4

	Donor-H	Acceptor-H	Donor-Acceptor	Angle
C32-H32A···O6	0.97	2.61	3.110(11)	112.6

11. Computational studies

11.1. Hydrolysis of the nucleophile

Since the analysis of the Potential Energy Surface (PES) indicated that the reaction needs of a previous step consisting of the hydrolysis of the silylated dienolate, we included in the initial system a molecule of water. This molecule of water required for the hydrolysis might come from the residual amount present in the solvent. Based on previous reports describing very related systems,[1] the initial system calculated in this work consists of the full catalyst interacting with the molecule of water through a Hydrogen bonding with the N atom of the quinuclidine fragment of the catalyst through. At the same time, another Hydrogen bonding between the N-H of the squaramide moiety and the O atom of the dienolate is present in the starting structure. Our results show that the hydrolysis of nucleophile **3e** into the corresponding negatively charged dienolate presents an asynchronous Transition State (TS). The overall process can be described as a molecule of water being deprotonated by the catalyst while the resulting hydroxide attacks the Silicon atom of the dienolate. Thus, a pentavalent Silicon species **Hyd-2** is formed (found as a pseudo minimum in the PES), which immediately undergoes the breaking of a O-Si bond (Scheme S1). The complete hydrolysis process takes place with a barrier of 15 kcal/mol where the reagents and products are isoenergetic. The hydrolysis leads to the formation of a negatively charged enolate that coordinates to the squaramide moiety of the catalyst, and at the same time the tertiary amine of the catalyst forms the corresponding tertiary ammonium cation that interacts through Hydrogen bonding with the TMSOH generated (Scheme S1).

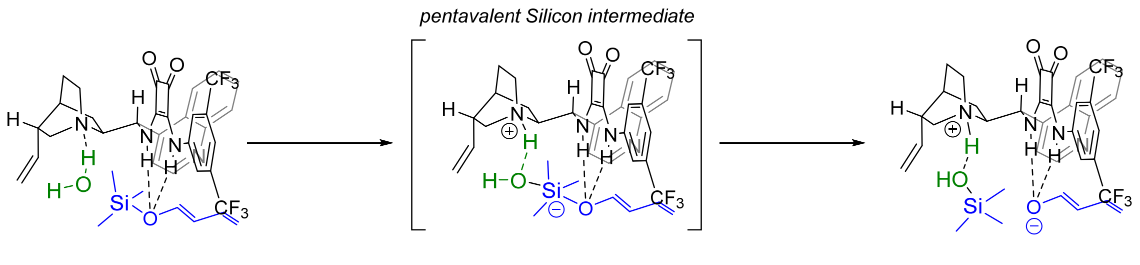

[1] (a) Humbrías-Martín, J.; Pérez-Aguilar, M. C.; Mas-Ballesté, R.; Litta, A. D.; Lattanzi, A.; Sala, G. D.; Fernández-Salas, J. A.; Alemán, J. *Adv. Synth. Catal.,* **2019**, *361*, 4790. (b) Frías, M.; Mas-Ballesté, R.; Arias, S.; Alvarado, C.; Alemán, J. *J. Am. Chem. Soc.* **2017***, 139*, 2, 672

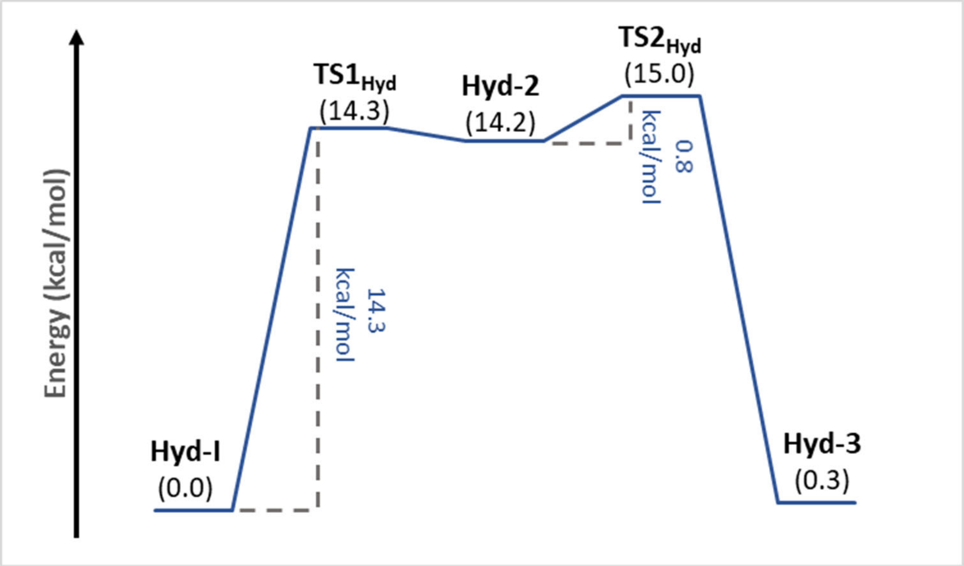

Scheme S1.

We could not find the **TS2$_{Hyd}$** but instead we had to estimate its energy from the electronic energy. In this manner, we carried out different structure optimizations from **Hyd-2** to **Hyd-3** by changing and fixing the distance between the Si-O$_{enolate}$ fixed (Table S10 and Figure S1).

Table S11.

**Entry**	**Distance Si-O$_{enolate}$ (Å)**	**Electronic Energy (Hartrees)**	**ΔΔE=ΔE-ΔE$_{Hyd-1}$ (kcal/mol)**
**Hyd-2**	1.79849	-2881.47191904	14.2
**2**[a]	1.90000	-2881.47030630	14.2
**3**[a]	1.92000	-2881.46974590	14.5
**4**[a]	1.94000	-2881.46914320	14.9
**5**[a]	1.94400	-2881.46901779	15.0
**6**[a]	1.94600	-2881.46894863	15.0
**7**[a]	1.94800	-2881.46888685	15.1
**8**[a]	1.95000	-2881.48376546	5.7
**Hyd-3**	3.58783	-2881.49054139	0.3

[a] ΔE was calculated as: ΔE = Electronic energy x 627.51

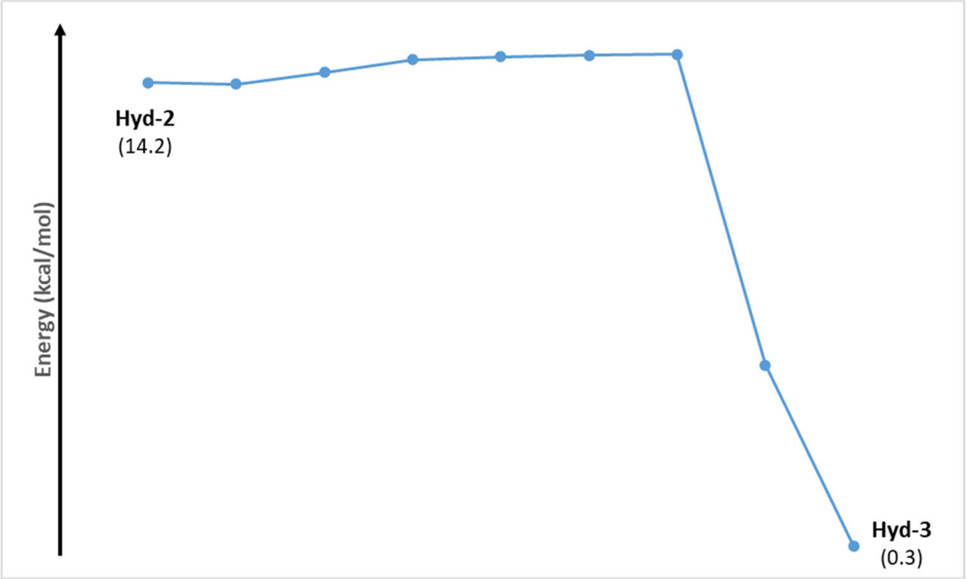

Figure S1

Therefore, as the difference in energy between the pentavalent Silicon species and the two TS is in both cases lower than 1 kcal/mol, we have considered the **ΔE** of this pseudominimun as the TS for all the hydrolysis processes.

11.2. Hydrolysis of (*Z)*-2a

(*Z*)-2a gave a very similar energy profile as for the **(*E*)-2a** with a slightly higher energy barrier (15.6 kcal/mol). Although, the hydrolysis is less favorable for the **(*Z*)-2a** than for (E)-2a, the difference in energy (1.4 kcal/mol) is not high enough to explain the lack of reactivity of the (Z)-2a.

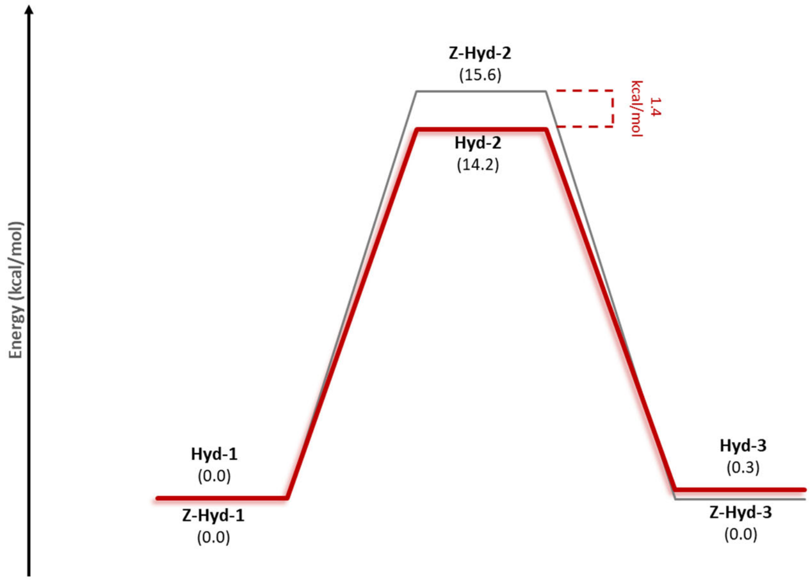

11.3. Origin of the diastereoselectivity

As mentioned, in the manuscript, in the C-C bond step (first step of the reaction pathway) two stereogenic centers are formed. As a result, 4 possible diastereoisomers can be formed (Figure S4), where one of them is the enantiomer of the experimentally observed product.

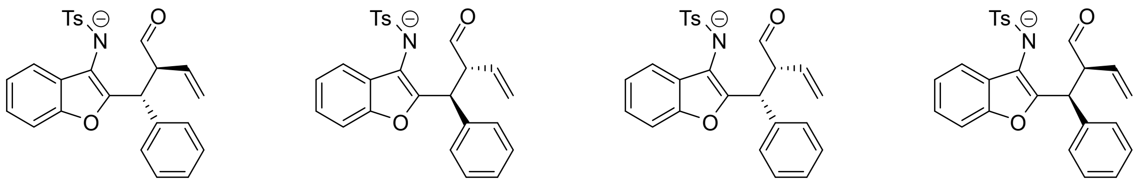

Figure S2

The initial approximation **DiastA-I** for the formation of diastereoisomer A is more stable than **I** by 2.2 kcal/mol. However, the TS has a barrier of 6.7 kcal/mol and the final product **DiastA-II** is less stable than **II**. Therefore, its formation it is not favorable from a kinetic perspective. On the other hand, the formation of diastereoisomer B (**DiastB-II**) has the same energy barrier as the formation of **II**. Nevertheless, the initial approximation **DiastB-I** is higher in energy than **I** by 5.8 kcal/mol, as well as the product **DiasA-II** is higher in energy than **II** by 3.4 kcal/mol. Thus, the preferred formation of **II** is the result of a thermodynamic control (Figure S3).

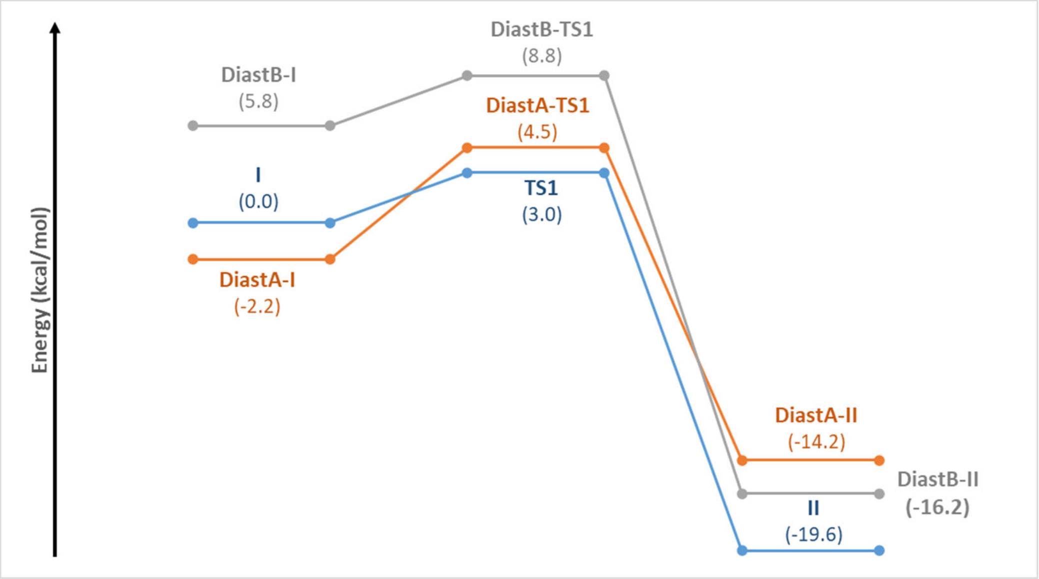

Figure S3.

11.5. Coordinates

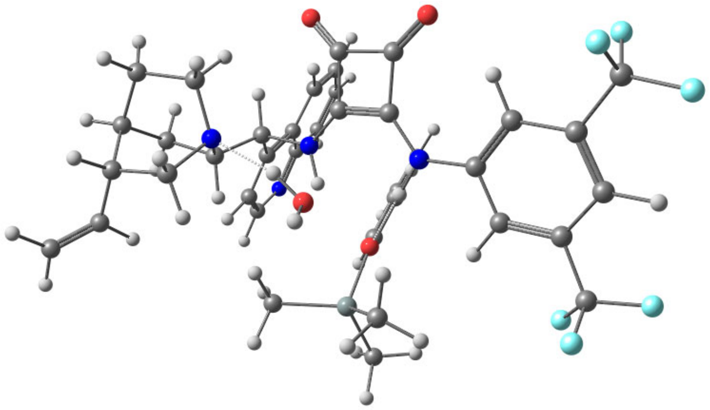

Hyd-1

Sum of electronic and thermal Free Energies= -2880.828675

(0.0 kcal/mol)

6	2.828441000	0.185897000	-0.748392000
1	3.004030000	0.469236000	-1.787644000
6	0.489646000	0.235075000	-1.520820000
6	0.534820000	0.508266000	-2.967806000
6	-0.979404000	0.381276000	-2.984897000
6	-0.904831000	0.135219000	-1.532316000
6	-3.103628000	-0.132627000	-0.421951000
6	-3.922318000	-0.104426000	-1.550905000
1	-3.493026000	-0.012341000	-2.544110000
6	-5.300987000	-0.187718000	-1.387736000
6	-5.894999000	-0.305262000	-0.139573000
1	-6.969535000	-0.373170000	-0.033256000
6	-5.060822000	-0.322489000	0.973943000
6	-3.686794000	-0.231118000	0.847434000
1	-3.054174000	-0.222495000	1.729078000
6	-5.638940000	-0.498562000	2.346904000
9	-6.919929000	-0.119889000	2.414071000
9	-4.965660000	0.203064000	3.271016000

9	-5.593015000	-1.783886000	2.741211000
7	1.385165000	0.160034000	-0.545610000
1	1.057785000	-0.120312000	0.375979000
7	-1.717746000	-0.071574000	-0.474545000
1	-1.236777000	-0.228871000	0.410100000
8	1.401555000	0.753444000	-3.774973000
8	-1.834856000	0.459307000	-3.834826000
6	3.442165000	-1.202686000	-0.513262000
1	3.272730000	-1.473652000	0.534365000
6	4.957382000	-1.222987000	-0.830374000
1	5.547477000	-1.121617000	0.082323000
1	5.224145000	-0.378243000	-1.475378000
6	5.272933000	-2.535186000	-1.561836000
1	6.352338000	-2.657779000	-1.671686000
6	4.588505000	-2.495541000	-2.934135000
1	4.714334000	-3.457125000	-3.439227000
1	5.054932000	-1.733184000	-3.562754000
6	3.086152000	-2.177450000	-2.718608000
1	2.830981000	-1.187431000	-3.098072000
1	2.448483000	-2.890080000	-3.244806000
6	3.144899000	-3.557554000	-0.763992000
1	2.664521000	-4.321426000	-1.379677000
1	2.751845000	-3.659150000	0.252019000
6	4.694144000	-3.732714000	-0.773397000
1	4.953195000	-4.647808000	-1.314120000
6	5.252365000	-3.832768000	0.617390000
1	4.957203000	-3.047097000	1.314515000
6	6.055357000	-4.802564000	1.041989000
1	6.365566000	-5.609289000	0.382594000
1	6.431841000	-4.827340000	2.059408000
7	2.732768000	-2.242931000	-1.287170000
6	3.402527000	1.263915000	0.163400000
6	3.294292000	2.642508000	-0.203534000
6	2.711717000	3.093525000	-1.417580000
1	2.312395000	2.382823000	-2.131798000

6	2.660819000	4.431196000	-1.715374000
1	2.214857000	4.756787000	-2.648407000
6	3.189219000	5.389571000	-0.820690000
1	3.146527000	6.442758000	-1.074962000
6	3.744916000	4.987784000	0.364157000
1	4.149046000	5.697805000	1.077064000
6	3.798843000	3.613534000	0.705131000
6	4.399040000	2.019123000	2.226622000
1	4.827504000	1.773325000	3.195036000
6	3.956409000	0.971139000	1.382343000
1	4.075203000	-0.046380000	1.732796000
7	4.336644000	3.286907000	1.917223000
6	-6.144555000	-0.154406000	-2.629213000
9	-5.849578000	-1.170001000	-3.455910000
9	-7.453755000	-0.233945000	-2.362627000
9	-5.953316000	0.973610000	-3.329946000
8	-0.217026000	0.106432000	2.044185000
6	0.231893000	1.367716000	2.313178000
6	0.003401000	2.392069000	1.487906000
1	-0.576426000	2.231374000	0.581089000
6	0.494962000	3.735596000	1.758471000
1	1.096652000	3.864423000	2.657125000
1	-0.340879000	4.689018000	0.065839000
6	0.249742000	4.786233000	0.972703000
1	0.631968000	5.772310000	1.210372000
1	0.804337000	1.488172000	3.231753000
1	1.021488000	-2.284419000	-0.561428000
8	0.271420000	-2.300558000	0.074444000
1	-0.004800000	-3.216740000	0.178893000
14	-0.124996000	-1.203449000	3.150171000
6	-1.482187000	-2.376005000	2.642799000
1	-2.421381000	-2.147545000	3.154918000
1	-1.663262000	-2.347395000	1.566188000
1	-1.212813000	-3.404351000	2.904198000
6	-0.452071000	-0.505315000	4.849874000

1	0.362814000	0.123068000	5.218750000
1	-1.370627000	0.089672000	4.863491000
1	-0.581996000	-1.321482000	5.567790000
6	1.593903000	-1.913710000	3.042993000
1	1.744530000	-2.691466000	3.798226000
1	1.758266000	-2.353402000	2.057486000
1	2.347615000	-1.138843000	3.218227000

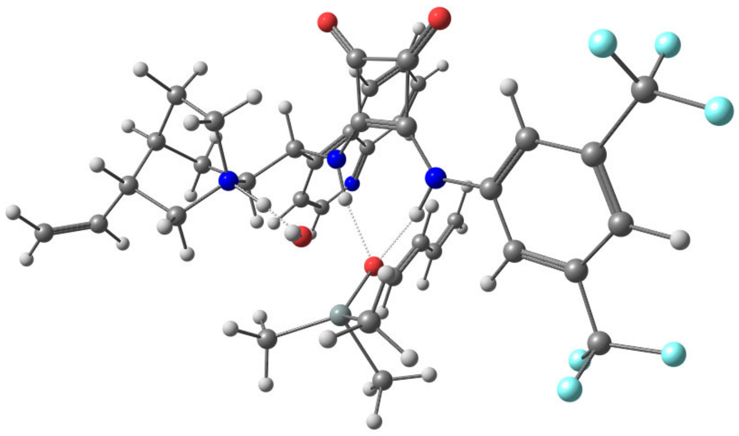

TS1$_{Hyd}$

Sum of electronic and thermal Free Energies= -2880.805921

(14.3 kcal/mol)

6	2.877117000	0.573117000	-0.557484000
1	3.119765000	0.910725000	-1.569189000
6	0.550880000	0.686830000	-1.423455000
6	0.594408000	1.159490000	-2.817504000
6	-0.919723000	1.037561000	-2.851770000
6	-0.840852000	0.557869000	-1.457436000
6	-3.029303000	0.056025000	-0.407692000
6	-3.856844000	0.350909000	-1.490200000
1	-3.436356000	0.682073000	-2.434772000
6	-5.232804000	0.209867000	-1.345861000
6	-5.813194000	-0.219030000	-0.161351000
1	-6.885840000	-0.327971000	-0.069880000
6	-4.969376000	-0.502542000	0.908167000
6	-3.597471000	-0.365530000	0.800628000
1	-2.956594000	-0.582697000	1.650558000
6	-5.536405000	-1.030809000	2.192749000
9	-6.818061000	-0.685410000	2.362607000
9	-4.858426000	-0.590377000	3.261799000
9	-5.488395000	-2.374126000	2.238367000
7	1.428711000	0.461037000	-0.452564000
1	1.069759000	-0.005143000	0.382446000
7	-1.642452000	0.143109000	-0.454674000

1	-1.147770000	-0.177730000	0.379273000
8	1.467090000	1.486983000	-3.588809000
8	-1.776369000	1.245283000	-3.677246000
6	3.519491000	-0.801691000	-0.305196000
1	3.352405000	-1.065892000	0.741864000
6	5.024860000	-0.828770000	-0.661916000
1	5.617049000	-1.148270000	0.197660000
1	5.363584000	0.176875000	-0.923772000
6	5.236141000	-1.766086000	-1.859617000
1	6.270771000	-1.709975000	-2.200840000
6	4.274252000	-1.346992000	-2.979305000
1	4.496930000	-1.889171000	-3.899656000
1	4.388893000	-0.280534000	-3.190357000
6	2.833737000	-1.657214000	-2.525388000
1	2.146296000	-0.851353000	-2.770702000
1	2.448278000	-2.568983000	-2.983414000
6	3.510019000	-3.174164000	-0.764385000
1	2.858150000	-3.982449000	-1.098308000
1	3.609331000	-3.248192000	0.319707000
6	4.887748000	-3.218317000	-1.465666000
1	4.805390000	-3.804365000	-2.386178000
6	5.918676000	-3.864858000	-0.587883000
1	6.095735000	-3.393402000	0.378479000
6	6.603173000	-4.954781000	-0.915523000
1	6.447843000	-5.458533000	-1.865899000
1	7.342863000	-5.382924000	-0.247240000
7	2.814802000	-1.886346000	-1.053817000
6	3.356685000	1.618032000	0.453922000
6	3.023375000	2.996843000	0.258858000
6	2.367823000	3.494180000	-0.897691000
1	2.141118000	2.833116000	-1.724162000
6	2.047624000	4.823090000	-1.007913000
1	1.551069000	5.182967000	-1.901937000
6	2.366325000	5.728856000	0.029325000
1	2.100237000	6.775150000	-0.069570000

6	3.020313000	5.285137000	1.147387000
1	3.291310000	5.954416000	1.956240000
6	3.369532000	3.918195000	1.285202000
6	4.340937000	2.291167000	2.553616000
1	4.870089000	2.009260000	3.460249000
6	4.026387000	1.287713000	1.602748000
1	4.328705000	0.274496000	1.832924000
7	4.026066000	3.550914000	2.423795000
6	-6.091767000	0.562811000	-2.525302000
9	-5.675803000	-0.049324000	-3.644193000
9	-7.374135000	0.225195000	-2.344223000
9	-6.066053000	1.880618000	-2.780798000
8	0.051708000	-0.742659000	1.713705000
6	0.308264000	0.124579000	2.723474000
6	0.237390000	1.449145000	2.538559000
1	-0.091547000	1.838871000	1.576300000
6	0.626579000	2.410239000	3.559547000
1	0.955218000	2.003962000	4.514864000
1	0.302671000	4.167294000	2.428722000
6	0.621416000	3.732443000	3.372566000
1	0.939482000	4.417897000	4.150223000
1	0.634717000	-0.306792000	3.666384000
1	1.698646000	-2.024499000	-0.617781000
8	0.515351000	-2.267345000	-0.104368000
1	0.053170000	-2.878637000	-0.686578000
14	-0.169651000	-2.492564000	1.809171000
6	-1.718512000	-3.215216000	0.982094000
1	-2.573070000	-3.260813000	1.661209000
1	-2.026875000	-2.668804000	0.086730000
1	-1.508939000	-4.244051000	0.663291000
6	-0.851325000	-2.470621000	3.618434000
1	-0.111128000	-2.243453000	4.393832000
1	-1.692034000	-1.778084000	3.757067000
1	-1.232229000	-3.474634000	3.845316000
6	1.365882000	-3.546588000	2.123479000

1	1.287614000	-4.113719000	3.054937000
1	1.581215000	-4.238445000	1.307057000
1	2.236166000	-2.885299000	2.225734000

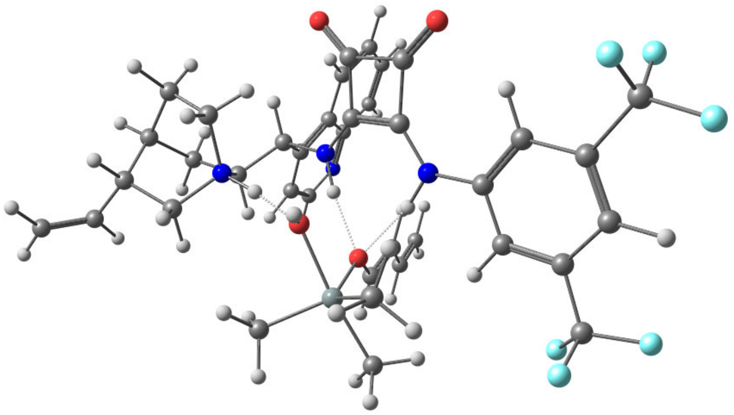

Hyd-2

Sum of electronic and thermal Free Energies= -2880.806023

(14.2 kcal/mol)

6	2.876787000	0.620812000	-0.415123000
1	3.138640000	1.061952000	-1.379607000
6	0.564205000	0.917443000	-1.268283000
6	0.631920000	1.568741000	-2.585316000
6	-0.886868000	1.530978000	-2.624154000
6	-0.830939000	0.855798000	-1.311190000
6	-3.034326000	0.269847000	-0.339811000
6	-3.853711000	0.766230000	-1.351398000
1	-3.424994000	1.252358000	-2.222571000
6	-5.232658000	0.623471000	-1.233291000
6	-5.821104000	-0.000433000	-0.143826000
1	-6.895575000	-0.105251000	-0.070512000
6	-4.983491000	-0.484499000	0.857233000
6	-3.609910000	-0.354164000	0.773707000
1	-2.972875000	-0.734567000	1.567346000
6	-5.563899000	-1.227880000	2.023828000
9	-6.827333000	-0.866075000	2.278079000
9	-4.859200000	-1.034879000	3.147324000
9	-5.578805000	-2.554893000	1.806287000
7	1.426846000	0.508289000	-0.342622000
1	1.048036000	-0.082269000	0.402038000

7	-1.646126000	0.339868000	-0.370448000
1	-1.162755000	-0.127187000	0.399085000
8	1.519500000	1.944634000	-3.317911000
8	-1.732517000	1.893533000	-3.405578000
6	3.500312000	-0.778561000	-0.315772000
1	3.268804000	-1.195284000	0.666585000
6	5.020286000	-0.787921000	-0.593149000
1	5.556789000	-1.235327000	0.245149000
1	5.383586000	0.237774000	-0.692961000
6	5.288605000	-1.552387000	-1.897449000
1	6.342869000	-1.474146000	-2.165343000
6	4.406992000	-0.952515000	-3.001047000
1	4.676738000	-1.364312000	-3.974367000
1	4.552329000	0.129572000	-3.049058000
6	2.936689000	-1.286074000	-2.689268000
1	2.275232000	-0.436445000	-2.843582000
1	2.563008000	-2.113266000	-3.293090000
6	3.479008000	-3.071650000	-1.124154000
1	2.831820000	-3.797374000	-1.616109000
1	3.501762000	-3.301654000	-0.058052000
6	4.895896000	-3.038473000	-1.738781000
1	4.864114000	-3.483548000	-2.737705000
6	5.861694000	-3.826845000	-0.902903000
1	5.974846000	-3.511286000	0.133703000
6	6.563528000	-4.861044000	-1.351168000
1	6.468957000	-5.209764000	-2.376173000
1	7.256994000	-5.396214000	-0.711181000
7	2.831203000	-1.729865000	-1.265086000
6	3.350010000	1.544714000	0.706537000
6	3.191317000	2.960845000	0.570865000
6	2.715877000	3.595608000	-0.606795000
1	2.477467000	3.013636000	-1.488071000
6	2.570965000	4.958254000	-0.660509000
1	2.207502000	5.423059000	-1.569995000
6	2.892719000	5.761753000	0.456559000

1	2.768549000	6.837162000	0.399256000
6	3.365077000	5.181563000	1.603192000
1	3.628234000	5.768686000	2.475784000
6	3.527167000	3.775855000	1.687714000
6	4.148286000	1.973317000	2.942618000
1	4.523839000	1.580167000	3.883539000
6	3.836513000	1.069914000	1.896135000
1	3.986186000	0.014941000	2.086389000
7	4.002035000	3.268215000	2.862389000
6	-6.082048000	1.186054000	-2.335795000
9	-5.728787000	0.696764000	-3.534307000
9	-7.382524000	0.917932000	-2.169120000
9	-5.963899000	2.520510000	-2.421241000
8	-0.017609000	-1.129031000	1.496305000
6	0.232493000	-0.644433000	2.730775000
6	0.361651000	0.669045000	2.969942000
1	0.220351000	1.383645000	2.160199000
6	0.721661000	1.183362000	4.282638000
1	0.830191000	0.446115000	5.077099000
1	0.839412000	3.237461000	3.787718000
6	0.934760000	2.473529000	4.554738000
1	1.212728000	2.805159000	5.548879000
1	0.375095000	-1.375257000	3.522625000
1	1.784712000	-1.862210000	-0.978134000
8	0.353132000	-2.198517000	-0.583846000
1	-0.077048000	-2.613813000	-1.337756000
14	-0.436712000	-2.816740000	1.037421000
6	-2.021959000	-3.190273000	0.033168000
1	-2.915110000	-3.286481000	0.654451000
1	-2.230775000	-2.451776000	-0.746185000
1	-1.888665000	-4.152583000	-0.479287000
6	-1.263626000	-3.176565000	2.769275000
1	-0.575903000	-3.278459000	3.617102000
1	-2.028545000	-2.443135000	3.059316000
1	-1.778328000	-4.142229000	2.681829000

6	0.916863000	-4.136857000	1.205821000
1	0.620259000	-4.948987000	1.875523000
1	1.226355000	-4.566256000	0.250406000
1	1.805865000	-3.672322000	1.653864000

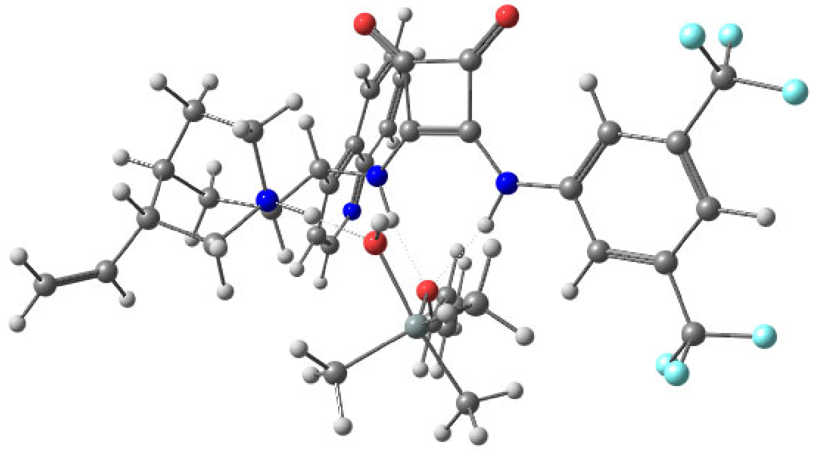

2

Electronic Energy= -2881.47030627

6	2.871310000	0.600548000	-0.421377000
1	3.127631000	1.037461000	-1.389140000
6	0.554812000	0.964522000	-1.230425000
6	0.618975000	1.614251000	-2.547681000
6	-0.899965000	1.614474000	-2.564737000
6	-0.842315000	0.936501000	-1.252726000
6	-3.045809000	0.373497000	-0.273323000
6	-3.861023000	0.862330000	-1.292164000
1	-3.429412000	1.345863000	-2.163276000
6	-5.240277000	0.716230000	-1.181733000
6	-5.834068000	0.096215000	-0.092884000
1	-6.908592000	-0.012228000	-0.026154000
6	-5.000930000	-0.379057000	0.915885000
6	-3.627282000	-0.244991000	0.839719000
1	-2.995072000	-0.618805000	1.639612000
6	-5.583728000	-1.119444000	2.083189000
9	-6.854002000	-0.771616000	2.322004000
9	-4.892412000	-0.907855000	3.211661000
9	-5.579301000	-2.448517000	1.877393000
7	1.420673000	0.529583000	-0.320272000
1	1.035172000	-0.059731000	0.425449000
7	-1.657032000	0.439478000	-0.301069000

1	-1.173922000	-0.026609000	0.470637000
8	1.505124000	1.965869000	-3.294363000
8	-1.746256000	1.997792000	-3.335746000
6	3.452429000	-0.817954000	-0.341258000
1	3.211427000	-1.239649000	0.636621000
6	4.969434000	-0.875883000	-0.628039000
1	5.494991000	-1.348990000	0.202924000
1	5.366561000	0.137621000	-0.721198000
6	5.206013000	-1.636401000	-1.940423000
1	6.261400000	-1.592062000	-2.211387000
6	4.342565000	-0.994630000	-3.034518000
1	4.593953000	-1.405068000	-4.013233000
1	4.525204000	0.082238000	-3.071045000
6	2.862793000	-1.279376000	-2.721203000
1	2.231204000	-0.405164000	-2.862409000
1	2.457646000	-2.086459000	-3.331751000
6	3.347514000	-3.102188000	-1.177181000
1	2.674276000	-3.797475000	-1.677569000
1	3.362622000	-3.345141000	-0.113823000
6	4.764026000	-3.109750000	-1.793027000
1	4.717300000	-3.546701000	-2.794854000
6	5.704781000	-3.933471000	-0.962300000
1	5.820338000	-3.633935000	0.078850000
6	6.382923000	-4.978831000	-1.420762000
1	6.284509000	-5.311827000	-2.450606000
1	7.059699000	-5.539371000	-0.784677000
7	2.747556000	-1.735616000	-1.300462000
6	3.398670000	1.504854000	0.691474000
6	3.306245000	2.926295000	0.550849000
6	2.838516000	3.578188000	-0.620662000
1	2.547362000	3.004556000	-1.491619000
6	2.764316000	4.946186000	-0.680845000
1	2.405097000	5.424079000	-1.585226000
6	3.153362000	5.738203000	0.422905000
1	3.086497000	6.818376000	0.360034000

6	3.617011000	5.140710000	1.564131000
1	3.928518000	5.718455000	2.426979000
6	3.705044000	3.728880000	1.655924000
6	4.255572000	1.903097000	2.911070000
1	4.626903000	1.496231000	3.847701000
6	3.879766000	1.011347000	1.875662000
1	3.977616000	-0.049276000	2.069862000
7	4.175445000	3.203515000	2.824604000
6	-6.083654000	1.266899000	-2.294850000
9	-5.729885000	0.757107000	-3.484812000
9	-7.386013000	1.008356000	-2.127870000
9	-5.957787000	2.599073000	-2.400789000
8	0.002415000	-1.111822000	1.479448000
6	0.338908000	-0.760639000	2.730015000
6	0.554606000	0.514663000	3.095412000
1	0.413347000	1.316188000	2.371303000
6	1.006154000	0.867330000	4.431802000
1	1.107649000	0.044310000	5.138522000
1	1.223345000	2.954975000	4.155344000
6	1.306613000	2.107207000	4.830173000
1	1.645376000	2.312375000	5.839562000
1	0.484501000	-1.569397000	3.443713000
1	1.708593000	-1.830017000	-1.020394000
8	0.222266000	-2.139182000	-0.647873000
1	-0.234513000	-2.462761000	-1.430658000
14	-0.574125000	-2.824669000	0.893142000
6	-2.177559000	-3.117155000	-0.115427000
1	-3.066661000	-3.233257000	0.507875000
1	-2.382612000	-2.328881000	-0.845868000
1	-2.065681000	-4.047772000	-0.687941000
6	-1.403979000	-3.141124000	2.624448000
1	-0.711784000	-3.308797000	3.456949000
1	-2.104248000	-2.356797000	2.940009000
1	-1.991263000	-4.063334000	2.529324000
6	0.688666000	-4.232773000	1.017951000

1	0.303113000	-5.069673000	1.607493000
1	1.016338000	-4.611373000	0.047179000
1	1.580423000	-3.868248000	1.544926000

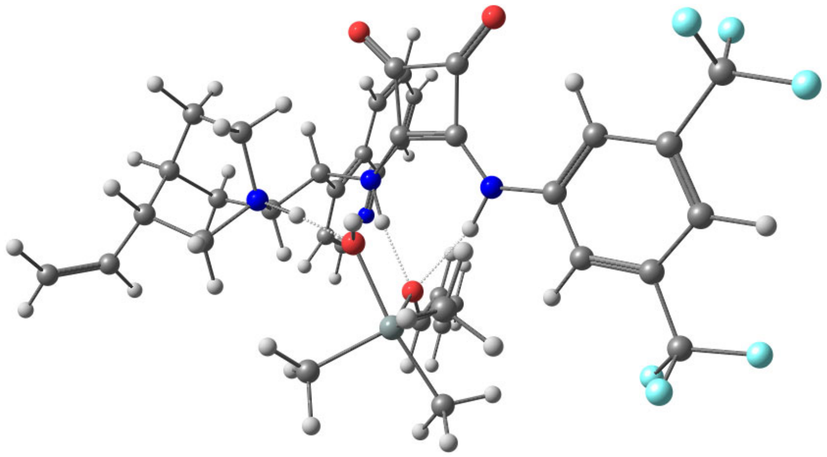

3

Electronic Energy= -2881.46974586

6	2.872097000	0.592771000	-0.426075000
1	3.127605000	1.023968000	-1.396660000
6	0.555221000	0.965619000	-1.229377000
6	0.618632000	1.608464000	-2.550013000
6	-0.900203000	1.616105000	-2.562759000
6	-0.842235000	0.943440000	-1.248006000
6	-3.045576000	0.387203000	-0.265372000
6	-3.860409000	0.870075000	-1.287375000
1	-3.428716000	1.350658000	-2.160057000
6	-5.239553000	0.721981000	-1.178096000
6	-5.833703000	0.105647000	-0.087384000
1	-6.908103000	-0.004682000	-0.021715000
6	-5.001071000	-0.363469000	0.924699000
6	-3.627599000	-0.226984000	0.849803000
1	-2.995667000	-0.595437000	1.652246000
6	-5.584536000	-1.099973000	2.094164000
9	-6.854567000	-0.750323000	2.331771000
9	-4.893196000	-0.885716000	3.222076000
9	-5.581425000	-2.429675000	1.892251000
7	1.421467000	0.531016000	-0.319465000
1	1.034229000	-0.052440000	0.430654000
7	-1.656816000	0.454069000	-0.292260000
1	-1.173360000	-0.009471000	0.481216000

8	1.504433000	1.951782000	-3.301064000
8	-1.746687000	2.000492000	-3.333088000
6	3.444265000	-0.829216000	-0.342287000
1	3.202578000	-1.245879000	0.637617000
6	4.959999000	-0.898926000	-0.632977000
1	5.484391000	-1.373673000	0.197794000
1	5.364023000	0.111430000	-0.729738000
6	5.187875000	-1.664217000	-1.944060000
1	6.242783000	-1.627648000	-2.217958000
6	4.325841000	-1.019129000	-3.037226000
1	4.571989000	-1.433046000	-4.015790000
1	4.515173000	0.056489000	-3.076238000
6	2.845151000	-1.293934000	-2.719685000
1	2.218626000	-0.416276000	-2.861734000
1	2.433684000	-2.100345000	-3.326830000
6	3.322015000	-3.115856000	-1.171635000
1	2.642564000	-3.807907000	-1.667998000
1	3.338940000	-3.355726000	-0.107657000
6	4.736455000	-3.134224000	-1.791863000
1	4.683797000	-3.573368000	-2.792432000
6	5.674215000	-3.961933000	-0.961638000
1	5.793479000	-3.661367000	0.078790000
6	6.345673000	-5.011694000	-1.419838000
1	6.243296000	-5.345781000	-2.448938000
1	7.020681000	-5.574919000	-0.784251000
7	2.730756000	-1.745401000	-1.297049000
6	3.410241000	1.498282000	0.680671000
6	3.323878000	2.919628000	0.535443000
6	2.851231000	3.569488000	-0.635211000
1	2.551396000	2.994007000	-1.501979000
6	2.783490000	4.937590000	-0.700137000
1	2.420371000	5.413852000	-1.603819000
6	3.184318000	5.731775000	0.397827000
1	3.122529000	6.812031000	0.331279000
6	3.652930000	5.136239000	1.538046000

1	3.973487000	5.715687000	2.396422000
6	3.734416000	3.724321000	1.634650000
6	4.284226000	1.900481000	2.892903000
1	4.659806000	1.495229000	3.828530000
6	3.896828000	1.006771000	1.863481000
1	3.990011000	-0.053628000	2.061592000
7	4.210327000	3.200972000	2.801983000
6	-6.082242000	1.265215000	-2.295382000
9	-5.728446000	0.746413000	-3.481486000
9	-7.384823000	1.008763000	-2.126975000
9	-5.955383000	2.596451000	-2.411098000
8	0.004204000	-1.091622000	1.487780000
6	0.352576000	-0.744330000	2.734644000
6	0.577545000	0.529360000	3.102082000
1	0.433719000	1.333306000	2.381134000
6	1.043428000	0.876394000	4.434772000
1	1.146255000	0.051004000	5.138625000
1	1.271951000	2.963410000	4.161877000
6	1.355732000	2.113314000	4.833719000
1	1.705004000	2.313566000	5.840522000
1	0.501528000	-1.554709000	3.446258000
1	1.694219000	-1.830773000	-1.014944000
8	0.197127000	-2.130968000	-0.637728000
1	-0.267922000	-2.442462000	-1.420528000
14	-0.592447000	-2.818443000	0.897443000
6	-2.198527000	-3.118424000	-0.105727000
1	-3.083743000	-3.244826000	0.521039000
1	-2.413172000	-2.325350000	-0.828358000
1	-2.083307000	-4.043080000	-0.687018000
6	-1.415167000	-3.112345000	2.634003000
1	-0.718212000	-3.274804000	3.463333000
1	-2.109966000	-2.322299000	2.946445000
1	-2.006495000	-4.033061000	2.550872000
6	0.665347000	-4.229760000	1.026135000
1	0.276603000	-5.063273000	1.618419000

1	0.990428000	-4.612788000	0.056218000
1	1.558747000	-3.867193000	1.551532000

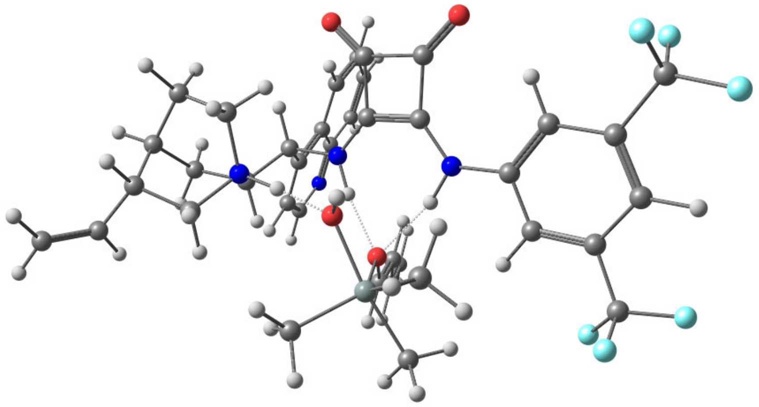

4

Electronic Energy= -2881.46914320

6	2.873284000	0.588241000	-0.426999000
1	3.130119000	1.016098000	-1.398695000
6	0.556938000	0.968077000	-1.228809000
6	0.620234000	1.607471000	-2.551081000
6	-0.898637000	1.620237000	-2.561144000
6	-0.840633000	0.949546000	-1.245390000
6	-3.043644000	0.395445000	-0.261295000
6	-3.858708000	0.878025000	-1.283290000
1	-3.427234000	1.359345000	-2.155697000
6	-5.237776000	0.728752000	-1.174284000
6	-5.831594000	0.111470000	-0.083926000
1	-6.905913000	0.000196000	-0.018465000
6	-4.998745000	-0.357210000	0.928196000
6	-3.625436000	-0.219407000	0.853619000
1	-2.993317000	-0.587231000	1.656053000
6	-5.582105000	-1.094642000	2.097121000
9	-6.851580000	-0.743828000	2.336074000
9	-4.889757000	-0.882628000	3.224823000
9	-5.580761000	-2.424087000	1.893380000
7	1.422599000	0.532233000	-0.319181000
1	1.033355000	-0.047571000	0.433437000
7	-1.654919000	0.462823000	-0.288031000

1	-1.170869000	-0.000160000	0.485893000
8	1.505928000	1.946158000	-3.304301000
8	-1.745261000	2.005259000	-3.331110000
6	3.439399000	-0.836056000	-0.340542000
1	3.195162000	-1.250235000	0.639751000
6	4.954823000	-0.913366000	-0.630745000
1	5.476539000	-1.390718000	0.200211000
1	5.363721000	0.095004000	-0.727210000
6	5.179529000	-1.679655000	-1.941782000
1	6.234590000	-1.647615000	-2.215615000
6	4.320305000	-1.030974000	-3.035066000
1	4.565014000	-1.445653000	-4.013652000
1	4.513737000	0.043897000	-3.073790000
6	2.838492000	-1.300171000	-2.718088000
1	2.215282000	-0.420235000	-2.860556000
1	2.424209000	-2.105502000	-3.324703000
6	3.307958000	-3.123314000	-1.168390000
1	2.625505000	-3.813060000	-1.663804000
1	3.324478000	-3.362008000	-0.104150000
6	4.721893000	-3.147661000	-1.789430000
1	4.666692000	-3.586584000	-2.789957000
6	5.656459000	-3.979403000	-0.959607000
1	5.777768000	-3.679080000	0.080649000
6	6.322794000	-5.032307000	-1.418032000
1	6.218202000	-5.366144000	-2.446989000
1	6.995677000	-5.598441000	-0.782782000
7	2.722186000	-1.750442000	-1.294955000
6	3.415684000	1.494219000	0.677233000
6	3.334847000	2.915457000	0.528459000
6	2.863575000	3.564145000	-0.643403000
1	2.560331000	2.987614000	-1.508325000
6	2.801295000	4.932331000	-0.711817000
1	2.439009000	5.407700000	-1.616305000
6	3.206568000	5.727733000	0.383648000
1	3.149260000	6.808057000	0.314266000

6	3.673787000	5.133274000	1.525000000
1	3.997522000	5.713666000	2.381545000
6	3.749450000	3.721278000	1.625302000
6	4.292476000	1.898381000	2.887905000
1	4.666688000	1.493990000	3.824438000
6	3.900993000	1.003668000	1.860899000
1	3.989651000	-0.056593000	2.061799000
7	4.223933000	3.198956000	2.793676000
6	-6.080870000	1.271943000	-2.291283000
9	-5.726057000	0.754821000	-3.477818000
9	-7.383152000	1.013558000	-2.123468000
9	-5.955759000	2.603448000	-2.405617000
8	0.003955000	-1.078174000	1.490819000
6	0.353245000	-0.733264000	2.736688000
6	0.589023000	0.538535000	3.105781000
1	0.454578000	1.344223000	2.384968000
6	1.055750000	0.880197000	4.439410000
1	1.148841000	0.053199000	5.142810000
1	1.307012000	2.965130000	4.169368000
6	1.380409000	2.113434000	4.840403000
1	1.729654000	2.308907000	5.848172000
1	0.493772000	-1.544473000	3.449373000
1	1.687410000	-1.830286000	-1.013394000
8	0.180761000	-2.124911000	-0.633603000
1	-0.289268000	-2.422665000	-1.418807000
14	-0.606050000	-2.819356000	0.891001000
6	-2.212448000	-3.124389000	-0.110432000
1	-3.095190000	-3.260502000	0.517698000
1	-2.434272000	-2.326491000	-0.825795000
1	-2.093166000	-4.043051000	-0.700239000
6	-1.423718000	-3.098616000	2.630704000
1	-0.722985000	-3.259113000	3.457060000
1	-2.114130000	-2.304876000	2.942624000
1	-2.018037000	-4.017890000	2.554300000
6	0.646086000	-4.234828000	1.019945000

1	0.253678000	-5.065849000	1.613255000
1	0.967406000	-4.620232000	0.049633000
1	1.542256000	-3.876590000	1.543393000

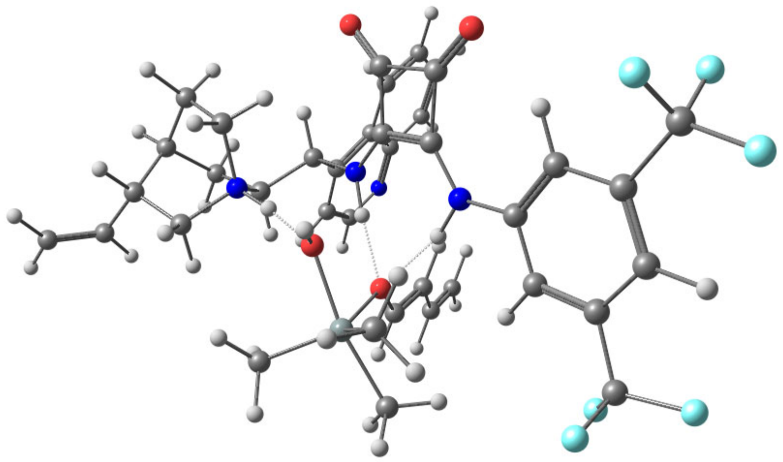

5

Electronic Energy= -2881.46901779

6	2.873337000	0.588135000	-0.427378000
1	3.130153000	1.015769000	-1.399178000
6	0.556969000	0.967963000	-1.229137000
6	0.620227000	1.607029000	-2.551567000
6	-0.898639000	1.619908000	-2.561538000
6	-0.840603000	0.949528000	-1.245625000
6	-3.043599000	0.395822000	-0.261280000
6	-3.858676000	0.878165000	-1.283377000
1	-3.427210000	1.359229000	-2.155928000
6	-5.237747000	0.728994000	-1.174279000
6	-5.831557000	0.112036000	-0.083733000
1	-6.905879000	0.000831000	-0.018205000
6	-4.998696000	-0.356411000	0.928487000
6	-3.625381000	-0.218697000	0.853823000
1	-2.993251000	-0.586336000	1.656333000
6	-5.582055000	-1.093505000	2.097626000
9	-6.851493000	-0.742540000	2.336552000
9	-4.889638000	-0.881263000	3.225242000
9	-5.580820000	-2.423001000	1.894218000
7	1.422655000	0.532302000	-0.319445000
1	1.033416000	-0.047327000	0.433307000
7	-1.654869000	0.463109000	-0.288094000

1	-1.170815000	0.000252000	0.485904000
8	1.505901000	1.945485000	-3.304918000
8	-1.745280000	2.004829000	-3.331534000
6	3.439246000	-0.836219000	-0.340669000
1	3.195060000	-1.250111000	0.639760000
6	4.954616000	-0.913851000	-0.631078000
1	5.476383000	-1.391072000	0.199921000
1	5.363660000	0.094429000	-0.727861000
6	5.179006000	-1.680499000	-1.941962000
1	6.234034000	-1.648710000	-2.215949000
6	4.319742000	-1.031931000	-3.035282000
1	4.564230000	-1.446896000	-4.013802000
1	4.513368000	0.042894000	-3.074300000
6	2.837926000	-1.300779000	-2.718021000
1	2.214856000	-0.420764000	-2.860613000
1	2.423410000	-2.106181000	-3.324383000
6	3.307301000	-3.123642000	-1.167959000
1	2.624652000	-3.813385000	-1.663107000
1	3.323939000	-3.362081000	-0.103664000
6	4.721139000	-3.148389000	-1.789197000
1	4.665720000	-3.587545000	-2.789610000
6	5.655685000	-3.980085000	-0.959305000
1	5.777146000	-3.679565000	0.080876000
6	6.321838000	-5.033167000	-1.417588000
1	6.217091000	-5.367202000	-2.446464000
1	6.994720000	-5.599254000	-0.782295000
7	2.721748000	-1.750696000	-1.294766000
6	3.415993000	1.494274000	0.676599000
6	3.335181000	2.915491000	0.527596000
6	2.863654000	3.563994000	-0.644267000
1	2.560204000	2.987323000	-1.509022000
6	2.801404000	4.932169000	-0.712907000
1	2.438917000	5.407391000	-1.617391000
6	3.206974000	5.727749000	0.382320000
1	3.149689000	6.808062000	0.312763000

6	3.674463000	5.133473000	1.523656000
1	3.998442000	5.714004000	2.380014000
6	3.750104000	3.721492000	1.624186000
6	4.293389000	1.898797000	2.886965000
1	4.667841000	1.494554000	3.823466000
6	3.901605000	1.003915000	1.860221000
1	3.990278000	-0.056311000	2.061312000
7	4.224877000	3.199358000	2.792526000
6	-6.080854000	1.271926000	-2.291395000
9	-5.726079000	0.754499000	-3.477808000
9	-7.383138000	1.013612000	-2.123486000
9	-5.955716000	2.603399000	-2.406065000
8	0.004780000	-1.076335000	1.492215000
6	0.354290000	-0.731724000	2.738095000
6	0.590339000	0.539961000	3.107418000
1	0.455954000	1.345814000	2.386777000
6	1.057280000	0.881282000	4.441057000
1	1.150263000	0.054140000	5.144302000
1	1.308972000	2.966207000	4.171358000
6	1.382270000	2.114374000	4.842229000
1	1.731681000	2.309589000	5.849991000
1	0.494698000	-1.543091000	3.450630000
1	1.687001000	-1.830300000	-1.013035000
8	0.180357000	-2.124658000	-0.633007000
1	-0.289856000	-2.422266000	-1.418157000
14	-0.606982000	-2.820886000	0.891028000
6	-2.213627000	-3.125405000	-0.110170000
1	-3.096299000	-3.261339000	0.518097000
1	-2.435355000	-2.327382000	-0.825424000
1	-2.094698000	-4.044046000	-0.700082000
6	-1.424401000	-3.100113000	2.630849000
1	-0.723535000	-3.260774000	3.457062000
1	-2.114606000	-2.306262000	2.942946000
1	-2.018900000	-4.019276000	2.554514000
6	0.644845000	-4.236669000	1.019616000

1	0.252343000	-5.067685000	1.612870000
1	0.965968000	-4.622009000	0.049213000
1	1.541151000	-3.878681000	1.543004000

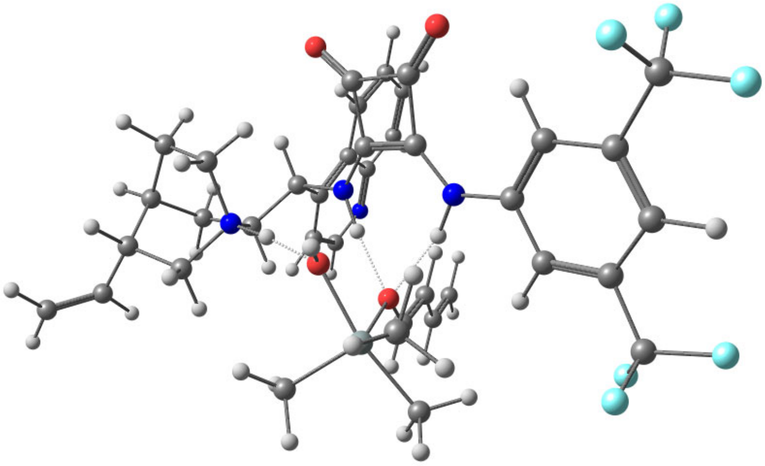

6

Electronic Energy= -2881.46894863

6	2.875687000	0.589380000	-0.464256000
1	3.122082000	0.996827000	-1.447954000
6	0.550833000	0.950737000	-1.250910000
6	0.602980000	1.555276000	-2.589953000
6	-0.915164000	1.561364000	-2.591542000
6	-0.847139000	0.929675000	-1.256729000
6	-3.044275000	0.397350000	-0.250545000
6	-3.862129000	0.851341000	-1.283317000
1	-3.434457000	1.315484000	-2.166616000
6	-5.240279000	0.698156000	-1.170665000
6	-5.831195000	0.103936000	-0.066120000
1	-6.905003000	-0.010222000	0.002271000
6	-4.996084000	-0.335832000	0.956940000
6	-3.623249000	-0.192909000	0.878959000
1	-2.989218000	-0.537455000	1.689991000
6	-5.575938000	-1.046966000	2.143940000
9	-6.849687000	-0.702718000	2.369431000
9	-4.889402000	-0.797200000	3.267438000
9	-5.560435000	-2.381328000	1.976742000
7	1.425692000	0.545295000	-0.335681000
1	1.043681000	-0.015002000	0.435281000
7	-1.655684000	0.471829000	-0.280351000

1	-1.167211000	0.034197000	0.505461000
8	1.482637000	1.879868000	-3.356468000
8	-1.766768000	1.922311000	-3.367635000
6	3.430672000	-0.837779000	-0.352001000
1	3.188446000	-1.224856000	0.640644000
6	4.943035000	-0.935821000	-0.651709000
1	5.466285000	-1.402036000	0.184684000
1	5.360950000	0.066337000	-0.770298000
6	5.151568000	-1.729301000	-1.948949000
1	6.204581000	-1.710349000	-2.231667000
6	4.288166000	-1.094730000	-3.046910000
1	4.522449000	-1.527656000	-4.020129000
1	4.487851000	-0.021855000	-3.105415000
6	2.807503000	-1.349419000	-2.713695000
1	2.188108000	-0.469414000	-2.870926000
1	2.385250000	-2.165639000	-3.299919000
6	3.277003000	-3.142150000	-1.129024000
1	2.584597000	-3.837360000	-1.602348000
1	3.303495000	-3.358274000	-0.060205000
6	4.684072000	-3.190366000	-1.764125000
1	4.615011000	-3.648442000	-2.755158000
6	5.619680000	-4.013636000	-0.927046000
1	5.758924000	-3.689793000	0.103811000
6	6.266074000	-5.086203000	-1.368120000
1	6.143589000	-5.443629000	-2.387109000
1	6.940043000	-5.645453000	-0.727936000
7	2.699407000	-1.767830000	-1.279970000
6	3.441471000	1.512947000	0.613898000
6	3.311419000	2.930850000	0.468155000
6	2.777024000	3.562584000	-0.685496000
1	2.466862000	2.974892000	-1.540155000
6	2.668346000	4.927935000	-0.751871000
1	2.259582000	5.390483000	-1.643091000
6	3.087202000	5.737173000	0.328301000
1	2.991029000	6.814920000	0.261761000

6	3.618452000	5.159571000	1.450131000
1	3.957285000	5.751242000	2.293024000
6	3.745697000	3.750976000	1.546346000
6	4.408111000	1.948831000	2.780971000
1	4.839109000	1.557839000	3.698648000
6	4.001030000	1.040705000	1.771902000
1	4.140726000	-0.015635000	1.964069000
7	4.288226000	3.245927000	2.692575000
6	-6.083403000	1.192878000	-2.309856000
9	-5.804443000	0.542132000	-3.450803000
9	-7.393299000	1.041139000	-2.082731000
9	-5.873478000	2.495176000	-2.555308000
8	0.031907000	-1.025246000	1.521696000
6	0.430791000	-0.670472000	2.748881000
6	0.660405000	0.606148000	3.105714000
1	0.475164000	1.407801000	2.391656000
6	1.182186000	0.957967000	4.415887000
1	1.327259000	0.132589000	5.112413000
1	1.371000000	3.048617000	4.140004000
6	1.496274000	2.197887000	4.804498000
1	1.888167000	2.399887000	5.795130000
1	0.621775000	-1.477201000	3.455586000
1	1.667281000	-1.834708000	-0.987438000
8	0.163667000	-2.119355000	-0.582832000
1	-0.321177000	-2.428619000	-1.354364000
14	-0.597319000	-2.781029000	0.966522000
6	-2.207836000	-3.134223000	-0.014620000
1	-3.079664000	-3.288652000	0.624414000
1	-2.457616000	-2.342758000	-0.728165000
1	-2.074223000	-4.050201000	-0.605318000
6	-1.403642000	-3.000857000	2.718770000
1	-0.696522000	-3.119506000	3.546490000
1	-2.100152000	-2.202295000	3.002925000
1	-1.989124000	-3.928236000	2.683728000
6	0.666074000	-4.181507000	1.131210000

1	0.283455000	-4.995930000	1.753297000
1	0.982827000	-4.595952000	0.171473000
1	1.563111000	-3.800542000	1.636856000

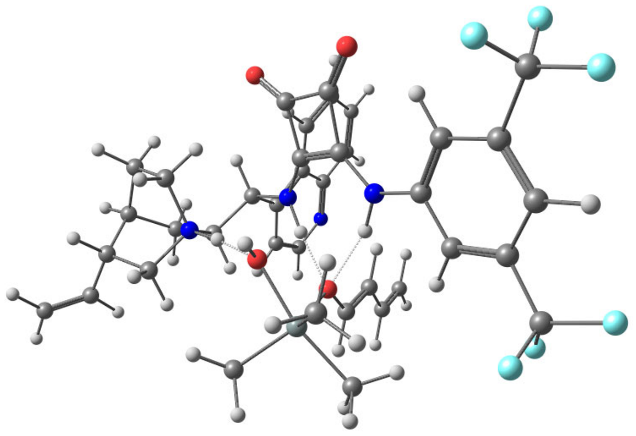

7

Electronic Energy= -2881.46888685

6	2.875659000	0.588012000	-0.465110000
1	3.121821000	0.994965000	-1.449073000
6	0.550806000	0.951195000	-1.250653000
6	0.602912000	1.555266000	-2.589917000
6	-0.915219000	1.562435000	-2.590981000
6	-0.847190000	0.931059000	-1.256017000
6	-3.044348000	0.399385000	-0.249682000
6	-3.862039000	0.852422000	-1.283006000
1	-3.434293000	1.316183000	-2.166466000
6	-5.240173000	0.698907000	-1.170668000
6	-5.831265000	0.105284000	-0.065900000
1	-6.905059000	-0.009158000	0.002227000
6	-4.996340000	-0.333478000	0.957742000
6	-3.623521000	-0.190197000	0.880070000
1	-2.989636000	-0.533910000	1.691548000
6	-5.576366000	-1.043804000	2.145149000
9	-6.850366000	-0.699923000	2.369792000
9	-4.890412000	-0.792677000	3.268701000
9	-5.560186000	-2.378305000	1.979191000
7	1.425705000	0.545426000	-0.335626000
1	1.043489000	-0.014174000	0.435826000
7	-1.655760000	0.474173000	-0.279191000
1	-1.167296000	0.036955000	0.506893000

8	1.482545000	1.878927000	-3.356863000
8	-1.766817000	1.923752000	-3.366913000
6	3.429261000	-0.839664000	-0.352819000
1	3.187241000	-1.226180000	0.640098000
6	4.941326000	-0.939290000	-0.653516000
1	5.464718000	-1.405643000	0.182713000
1	5.360075000	0.062442000	-0.772787000
6	5.148231000	-1.733476000	-1.950572000
1	6.201095000	-1.715772000	-2.233914000
6	4.284890000	-1.098308000	-3.048225000
1	4.518116000	-1.531756000	-4.021462000
1	4.485726000	-0.025663000	-3.107096000
6	2.804136000	-1.351246000	-2.714065000
1	2.185667000	-0.470559000	-2.871078000
1	2.380580000	-2.167110000	-3.299840000
6	3.272600000	-3.144154000	-1.129205000
1	2.579217000	-3.838646000	-1.602145000
1	3.299287000	-3.360180000	-0.060379000
6	4.679328000	-3.193995000	-1.764928000
1	4.609351000	-3.652415000	-2.755737000
6	5.614561000	-4.017801000	-0.927951000
1	5.754162000	-3.694010000	0.102870000
6	6.260304000	-5.090721000	-1.369125000
1	6.137428000	-5.448105000	-2.388081000
1	6.934101000	-5.650311000	-0.729058000
7	2.696464000	-1.769196000	-1.280150000
6	3.443111000	1.511242000	0.612446000
6	3.314664000	2.929244000	0.466294000
6	2.780296000	3.561193000	-0.687248000
1	2.468846000	2.973543000	-1.541470000
6	2.673215000	4.926647000	-0.754067000
1	2.264434000	5.389353000	-1.645197000
6	3.093735000	5.735782000	0.325538000
1	2.998831000	6.813619000	0.258652000
6	3.624979000	5.157954000	1.447254000

1	3.965049000	5.749528000	2.289717000
6	3.750577000	3.749239000	1.543921000
6	4.411476000	1.946740000	2.778822000
1	4.842484000	1.555548000	3.696408000
6	4.002701000	1.038735000	1.770325000
1	4.141105000	-0.017699000	1.962927000
7	4.293172000	3.243951000	2.690008000
6	-6.083054000	1.192697000	-2.310440000
9	-5.803374000	0.541462000	-3.450941000
9	-7.392978000	1.040560000	-2.083764000
9	-5.873535000	2.494946000	-2.556475000
8	0.032233000	-1.022492000	1.523211000
6	0.432778000	-0.668324000	2.749882000
6	0.663861000	0.608002000	3.107016000
1	0.478524000	1.410070000	2.393448000
6	1.187344000	0.958838000	4.416744000
1	1.332350000	0.133028000	5.112784000
1	1.377980000	3.049443000	4.141633000
6	1.503069000	2.198280000	4.805607000
1	1.896160000	2.399432000	5.795937000
1	0.623990000	-1.475371000	3.456208000
1	1.664702000	-1.834757000	-0.987004000
8	0.160298000	-2.118243000	-0.580856000
1	-0.325642000	-2.426465000	-1.352113000
14	-0.599742000	-2.779597000	0.968328000
6	-2.210175000	-3.134829000	-0.012415000
1	-3.081350000	-3.291818000	0.626880000
1	-2.461860000	-2.342461000	-0.724324000
1	-2.075328000	-4.049423000	-0.604954000
6	-1.405846000	-2.996021000	2.720873000
1	-0.698419000	-3.113173000	3.548515000
1	-2.102256000	-2.197032000	3.003928000
1	-1.991283000	-3.923485000	2.687594000
6	0.663764000	-4.179663000	1.134523000
1	0.281615000	-4.993144000	1.758130000

1	0.979764000	-4.595616000	0.175184000
1	1.561173000	-3.797849000	1.638863000

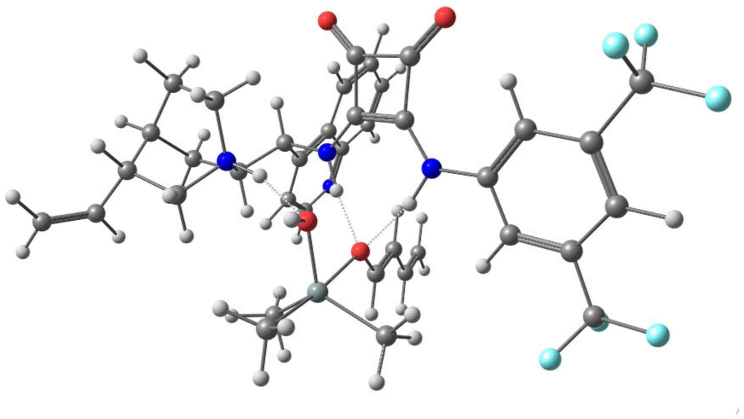

8

Electronic Energy= -2881.48376546

6	2.805784000	0.674113000	-0.564091000
1	3.054391000	1.037897000	-1.563804000
6	0.474370000	0.756733000	-1.424407000
6	0.498608000	1.261266000	-2.802942000
6	-1.014006000	1.136464000	-2.821087000
6	-0.917781000	0.619593000	-1.437465000
6	-3.085851000	0.071755000	-0.391172000
6	-3.912166000	0.309236000	-1.490466000
1	-3.492308000	0.613961000	-2.444068000
6	-5.286253000	0.155695000	-1.350455000
6	-5.870069000	-0.235798000	-0.153479000
1	-6.940958000	-0.361692000	-0.067114000
6	-5.030204000	-0.459513000	0.931314000
6	-3.658540000	-0.300065000	0.829330000
1	-3.022420000	-0.456693000	1.694989000
6	-5.589519000	-0.863684000	2.263640000
9	-6.888539000	-1.176918000	2.199533000
9	-5.466070000	0.114829000	3.174792000
9	-4.948871000	-1.931445000	2.766550000
7	1.365121000	0.488461000	-0.474453000
1	1.002615000	0.044447000	0.383215000
7	-1.699133000	0.173148000	-0.435919000
1	-1.189478000	-0.193617000	0.379365000

8	1.362012000	1.602966000	-3.581908000
8	-1.879007000	1.358101000	-3.633921000
6	3.485242000	-0.682575000	-0.338347000
1	3.247935000	-1.029814000	0.668836000
6	5.005214000	-0.683334000	-0.595632000
1	5.539363000	-1.006234000	0.299037000
1	5.340666000	0.332193000	-0.819437000
6	5.313831000	-1.595141000	-1.791061000
1	6.375166000	-1.541178000	-2.035178000
6	4.466712000	-1.130692000	-2.982938000
1	4.761809000	-1.655677000	-3.892087000
1	4.618507000	-0.062487000	-3.153477000
6	2.985806000	-1.418862000	-2.682428000
1	2.339274000	-0.579958000	-2.929497000
1	2.616021000	-2.298333000	-3.208505000
6	3.480131000	-3.051800000	-0.931770000
1	2.861324000	-3.814321000	-1.403459000
1	3.439403000	-3.190109000	0.149878000
6	4.928796000	-3.057562000	-1.471349000
1	4.956742000	-3.621466000	-2.408188000
6	5.860271000	-3.716558000	-0.495534000
1	5.914111000	-3.273154000	0.498132000
6	6.597834000	-4.783918000	-0.776904000
1	6.561029000	-5.257209000	-1.754443000
1	7.264653000	-5.222309000	-0.042134000
7	2.846807000	-1.724008000	-1.220472000
6	3.214611000	1.713531000	0.474239000
6	2.963640000	3.098813000	0.217659000
6	2.464989000	3.598976000	-1.014059000
1	2.276399000	2.930881000	-1.845439000
6	2.229301000	4.939546000	-1.182078000
1	1.849354000	5.302195000	-2.130445000
6	2.477467000	5.852159000	-0.132076000
1	2.282523000	6.908203000	-0.280465000
6	2.965058000	5.401967000	1.065577000

1	3.169857000	6.075434000	1.890130000
6	3.218530000	4.022245000	1.268866000
6	3.923902000	2.372985000	2.682476000
1	4.297506000	2.084156000	3.661177000
6	3.701693000	1.368524000	1.706906000
1	3.913329000	0.343387000	1.984432000
7	3.694620000	3.643813000	2.491278000
6	-6.135621000	0.411532000	-2.561434000
9	-5.829303000	-0.423393000	-3.567887000
9	-7.442052000	0.261650000	-2.313058000
9	-5.964422000	1.654900000	-3.035542000
8	0.055162000	-0.799617000	1.576686000
6	0.135265000	-0.138946000	2.728920000
6	0.259501000	1.199410000	2.830115000
1	0.293058000	1.807273000	1.926479000
6	0.385696000	1.868984000	4.112969000
1	0.331870000	1.230147000	4.994369000
1	0.636447000	3.856804000	3.426813000
6	0.574113000	3.182741000	4.277128000
1	0.671776000	3.622942000	5.263197000
1	0.108216000	-0.746682000	3.638184000
1	1.842259000	-1.798695000	-0.946694000
8	0.327416000	-2.341972000	-0.201966000
1	0.125860000	-3.117997000	-0.736641000
14	0.163217000	-2.744767000	1.491753000
6	0.238630000	-4.662592000	1.123693000
1	0.269903000	-5.228440000	2.063065000
1	-0.636373000	-5.033327000	0.572066000
1	1.128290000	-4.961784000	0.552780000
6	-1.547529000	-2.811961000	2.315004000
1	-1.793768000	-1.977937000	2.976182000
1	-2.302570000	-2.826762000	1.518162000
1	-1.679821000	-3.741958000	2.875890000
6	1.728246000	-2.706224000	2.574575000
1	1.464017000	-3.021314000	3.591995000

1	2.463142000	-3.435828000	2.221705000
1	2.218397000	-1.732803000	2.665357000

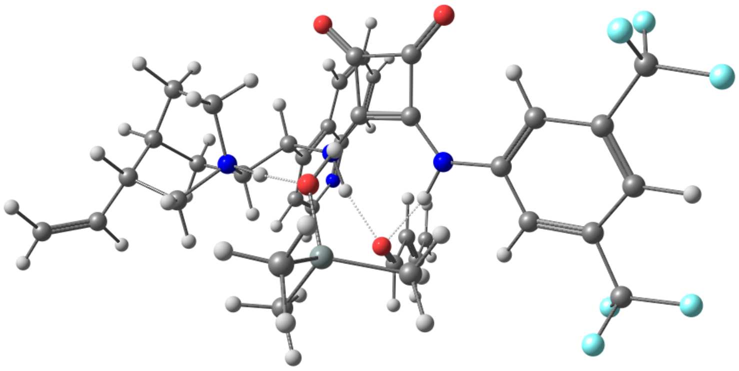

Hyd-3

Sum of electronic and thermal Free Energies= -2880.828186

(0.3 kcal/mol)

6	2.838102000	0.522864000	-0.464718000
1	3.071729000	0.578625000	-1.530126000
6	0.532783000	0.327832000	-1.315226000
6	0.595372000	0.372881000	-2.782100000
6	-0.920006000	0.314699000	-2.801433000
6	-0.867943000	0.255303000	-1.321764000
6	-3.068225000	0.129696000	-0.220715000
6	-3.859328000	0.029244000	-1.366400000
1	-3.407924000	0.025276000	-2.353790000
6	-5.240037000	-0.059356000	-1.232922000
6	-5.865249000	-0.061756000	0.005899000
1	-6.940398000	-0.143318000	0.091368000
6	-5.060707000	0.047653000	1.134267000
6	-3.683825000	0.153610000	1.035997000
1	-3.075868000	0.253303000	1.930484000
6	-5.660107000	0.018142000	2.508920000
9	-6.997505000	-0.007155000	2.486288000
9	-5.296128000	1.083167000	3.238295000
9	-5.258480000	-1.065692000	3.195617000
7	1.406040000	0.305899000	-0.315089000
1	1.007431000	0.202398000	0.642522000
7	-1.678943000	0.178577000	-0.249902000
1	-1.182480000	0.140101000	0.666417000

8	1.482296000	0.421063000	-3.607751000
8	-1.761667000	0.306137000	-3.667937000
6	3.589882000	-0.664508000	0.143897000
1	3.326630000	-0.741692000	1.200065000
6	5.118949000	-0.610653000	-0.060379000
1	5.628364000	-0.635364000	0.903887000
1	5.392226000	0.331479000	-0.541216000
6	5.557507000	-1.778413000	-0.954177000
1	6.623683000	-1.701119000	-1.169255000
6	4.743008000	-1.720068000	-2.252392000
1	5.124310000	-2.436948000	-2.980329000
1	4.819372000	-0.724320000	-2.695226000
6	3.277924000	-2.050610000	-1.926771000
1	2.576187000	-1.374908000	-2.412384000
1	3.009549000	-3.071461000	-2.197575000
6	3.798207000	-3.092916000	0.226139000
1	3.256038000	-4.007538000	-0.013143000
1	3.731885000	-2.915735000	1.300181000
6	5.262536000	-3.130625000	-0.265950000
1	5.365808000	-3.918060000	-1.018099000
6	6.198329000	-3.435738000	0.867924000
1	6.185241000	-2.742221000	1.707940000
6	7.019489000	-4.478616000	0.893887000
1	7.053558000	-5.195199000	0.077523000
1	7.687452000	-4.653510000	1.730521000
7	3.080066000	-1.956874000	-0.440743000
6	3.199772000	1.844892000	0.202000000
6	2.983933000	3.074521000	-0.496808000
6	2.548943000	3.157313000	-1.846290000
1	2.379954000	2.259919000	-2.428485000
6	2.346286000	4.374542000	-2.444775000
1	2.012547000	4.415919000	-3.475381000
6	2.567949000	5.574444000	-1.731976000
1	2.400321000	6.528256000	-2.219232000
6	2.993826000	5.528917000	-0.431637000

1	3.175008000	6.429809000	0.143700000
6	3.209656000	4.285837000	0.214635000
6	3.808730000	3.175366000	2.118008000
1	4.124120000	3.215938000	3.157116000
6	3.615308000	1.912598000	1.505259000
1	3.787348000	1.029856000	2.108462000
7	3.621225000	4.318183000	1.515201000
6	-6.050270000	-0.159996000	-2.492236000
9	-5.688545000	-1.220782000	-3.232530000
9	-7.361287000	-0.280280000	-2.251278000
9	-5.891318000	0.918991000	-3.273863000
8	-0.065614000	-0.044696000	1.952728000
6	0.055698000	0.950595000	2.780596000
6	0.229826000	2.267683000	2.472247000
1	0.273499000	2.574409000	1.426693000
6	0.377178000	3.275118000	3.499437000
1	0.314132000	2.909035000	4.525789000
1	0.659120000	5.013682000	2.314393000
6	0.584191000	4.587481000	3.311515000
1	0.685311000	5.270730000	4.147623000
1	0.021067000	0.692363000	3.851675000
1	2.061365000	-2.054414000	-0.253685000
8	0.398733000	-2.657458000	-0.265474000
1	-0.165823000	-2.386671000	-0.997978000
14	-0.307757000	-3.502906000	1.028228000
6	-0.486453000	-5.295915000	0.532433000
1	-0.878417000	-5.890181000	1.363788000
1	-1.176375000	-5.416241000	-0.308296000
1	0.474303000	-5.730464000	0.240531000
6	-1.961248000	-2.759027000	1.448567000
1	-1.803554000	-1.772303000	1.888623000
1	-2.615403000	-2.661165000	0.576736000
1	-2.487038000	-3.378878000	2.181957000
6	0.904049000	-3.299867000	2.425424000
1	0.471129000	-3.677641000	3.357249000

1	1.839860000	-3.840122000	2.262840000
1	1.109779000	-2.235035000	2.569062000

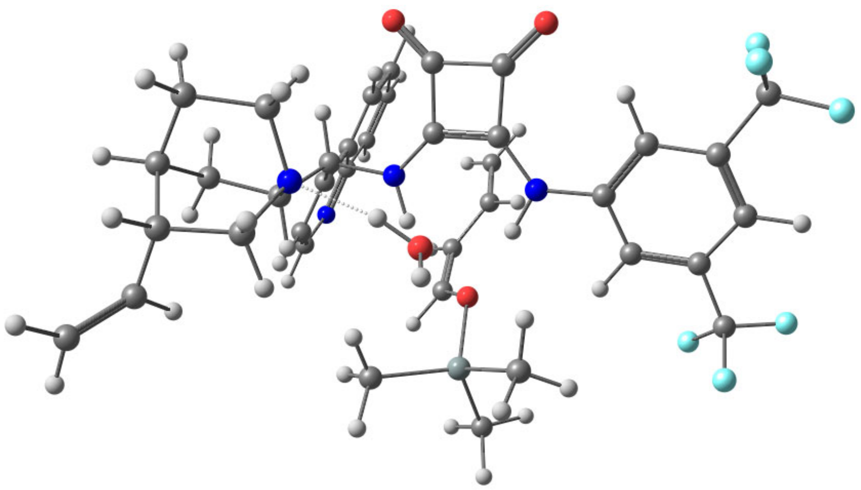

Z-hyd-1

Sum of electronic and thermal Free Energies= -2880.829677

(0.0 kcal/mol)

6	-2.863125000	0.574183000	0.512986000
1	-3.023759000	1.258993000	1.347487000
6	-0.520401000	0.902248000	1.199205000
6	-0.563544000	1.771391000	2.388008000
6	0.945400000	1.632548000	2.479560000
6	0.870260000	0.785096000	1.273731000
6	3.075910000	0.059000000	0.416043000
6	3.870961000	0.519385000	1.465962000
1	3.420345000	0.979365000	2.339769000
6	5.252218000	0.392023000	1.375021000
6	5.872639000	-0.185752000	0.276562000
1	6.949064000	-0.282261000	0.226486000
6	5.062960000	-0.628800000	-0.763619000
6	3.685539000	-0.505861000	-0.710017000
1	3.073971000	-0.834055000	-1.544173000
6	5.669777000	-1.302571000	-1.958652000
9	6.964598000	-1.004721000	-2.110495000
9	5.045914000	-0.965805000	-3.097111000
9	5.587643000	-2.642272000	-1.864921000
7	-1.419289000	0.433465000	0.343010000

1	-1.101518000	-0.221629000	-0.366898000
7	1.686901000	0.125078000	0.424478000
1	1.212533000	-0.401508000	-0.306766000
8	-1.425989000	2.355051000	3.003415000
8	1.796579000	2.049174000	3.228984000
6	-3.512011000	-0.773507000	0.861937000
1	-3.348282000	-1.456079000	0.020303000
6	-5.027518000	-0.620579000	1.145783000
1	-5.619756000	-0.921221000	0.279702000
1	-5.274136000	0.427749000	1.347465000
6	-5.372169000	-1.463315000	2.381285000
1	-6.453093000	-1.483653000	2.534483000
6	-4.665109000	-0.847673000	3.595127000
1	-4.834117000	-1.469373000	4.478407000
1	-5.081621000	0.140090000	3.806949000
6	-3.151217000	-0.742815000	3.275815000
1	-2.828129000	0.296731000	3.213727000
1	-2.546847000	-1.214260000	4.052937000
6	-3.296738000	-2.813565000	2.084174000
1	-2.813658000	-3.273162000	2.949404000
1	-2.946718000	-3.342825000	1.192910000
6	-4.849067000	-2.906881000	2.202001000
1	-5.112283000	-3.472948000	3.100466000
6	-5.457869000	-3.596377000	1.015209000
1	-5.175593000	-3.210211000	0.034681000
6	-6.292370000	-4.627948000	1.084993000
1	-6.592629000	-5.048917000	2.041237000
1	-6.706026000	-5.088727000	0.193897000
7	-2.830384000	-1.418328000	2.003572000
6	-3.415674000	1.202094000	-0.760619000
6	-3.226584000	2.599165000	-1.006134000
6	-2.658957000	3.498334000	-0.064616000
1	-2.340831000	3.145066000	0.909012000
6	-2.515008000	4.828530000	-0.363976000
1	-2.083895000	5.500335000	0.369641000

6	-2.920139000	5.332196000	-1.621156000
1	-2.797692000	6.386441000	-1.842922000
6	-3.465082000	4.490219000	-2.553603000
1	-3.788714000	4.844941000	-3.525768000
6	-3.634001000	3.111373000	-2.268769000
6	-4.368547000	1.065289000	-2.971437000
1	-4.821038000	0.458419000	-3.751632000
6	-4.001555000	0.454305000	-1.747572000
1	-4.184273000	-0.607054000	-1.634028000
7	-4.190715000	2.331302000	-3.242113000
6	6.071640000	0.913639000	2.520048000
9	5.685078000	0.382502000	3.690073000
9	7.375853000	0.647690000	2.379204000
9	5.954098000	2.244166000	2.649542000
1	-1.119179000	-1.767258000	1.467170000
8	-0.305257000	-2.042892000	0.984421000
1	-0.090515000	-2.939437000	1.262008000
8	0.172837000	-0.919033000	-2.023952000
6	-0.605152000	-0.171930000	-2.866951000
6	-0.585603000	1.163026000	-2.909919000
1	-1.289778000	1.640105000	-3.586373000
6	0.285765000	2.018194000	-2.115266000
1	1.061189000	1.533740000	-1.528838000
1	-0.584267000	3.867746000	-2.657180000
6	0.177983000	3.347974000	-2.082335000
1	0.843636000	3.955918000	-1.479230000
1	-1.288940000	-0.733781000	-3.500146000
14	0.221250000	-2.628415000	-2.212813000
6	-1.472071000	-3.273114000	-1.770495000
1	-1.704510000	-3.011439000	-0.736865000
1	-2.244995000	-2.858290000	-2.425335000
1	-1.516049000	-4.362395000	-1.867435000
6	1.599352000	-3.248636000	-1.122863000
1	1.695617000	-2.681833000	-0.195237000
1	1.404396000	-4.290890000	-0.848419000

1	2.560697000	-3.229670000	-1.643292000
6	0.617570000	-2.965241000	-4.005267000
1	0.791128000	-4.035371000	-4.157520000
1	-0.185726000	-2.666282000	-4.683820000
1	1.528085000	-2.440227000	-4.310214000

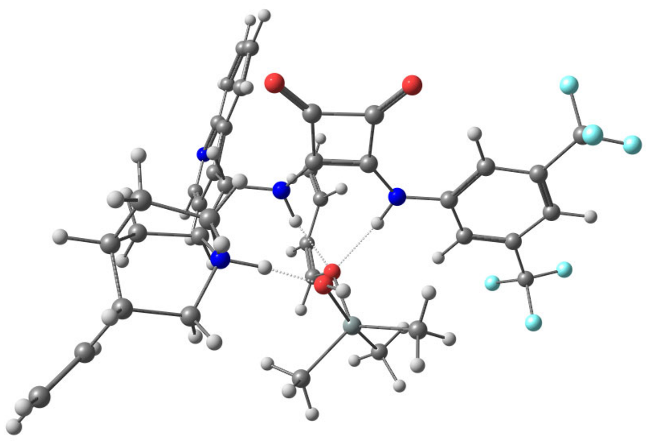

Z-Hyd-2

Sum of electronic and thermal Free Energies= -2880.804746

(15.6 kcal/mol)

6	2.895306000	0.631138000	-0.363949000
1	3.140435000	1.080538000	-1.329646000
6	0.571266000	0.929520000	-1.180828000
6	0.632131000	1.579707000	-2.499073000
6	-0.884586000	1.530315000	-2.537921000
6	-0.824428000	0.862113000	-1.220883000
6	-3.034334000	0.275493000	-0.264691000
6	-3.842014000	0.754398000	-1.293771000
1	-3.405914000	1.232829000	-2.165161000
6	-5.221922000	0.607407000	-1.193388000
6	-5.823693000	-0.004723000	-0.104781000
1	-6.898659000	-0.113261000	-0.045318000
6	-4.997896000	-0.470887000	0.914281000
6	-3.623823000	-0.335373000	0.848292000
1	-2.997764000	-0.703546000	1.655646000
6	-5.590244000	-1.202256000	2.082519000
9	-6.859756000	-0.845953000	2.313150000
9	-4.903520000	-0.989202000	3.213344000
9	-5.592537000	-2.532117000	1.882509000
7	1.445418000	0.531489000	-0.262545000

1	1.075602000	-0.048266000	0.494929000
7	-1.644688000	0.348981000	-0.280988000
1	-1.168955000	-0.102256000	0.501171000
8	1.516743000	1.962889000	-3.231337000
8	-1.730542000	1.883529000	-3.323477000
6	3.501798000	-0.777681000	-0.288728000
1	3.271796000	-1.198224000	0.693107000
6	5.018496000	-0.807178000	-0.583279000
1	5.558050000	-1.267661000	0.245938000
1	5.395654000	0.213541000	-0.681296000
6	5.262726000	-1.566907000	-1.894845000
1	6.315493000	-1.502153000	-2.172072000
6	4.379895000	-0.946355000	-2.985642000
1	4.633329000	-1.355384000	-3.964478000
1	4.541260000	0.133743000	-3.027120000
6	2.907905000	-1.258712000	-2.660953000
1	2.259638000	-0.397077000	-2.802176000
1	2.514609000	-2.075080000	-3.267015000
6	3.438163000	-3.064293000	-1.115025000
1	2.775569000	-3.775503000	-1.607517000
1	3.466256000	-3.304059000	-0.051363000
6	4.850386000	-3.047962000	-1.740854000
1	4.805140000	-3.488210000	-2.741343000
6	5.811928000	-3.852332000	-0.915144000
1	5.934441000	-3.544303000	0.122703000
6	6.499931000	-4.891374000	-1.373433000
1	6.395733000	-5.232920000	-2.399906000
1	7.191068000	-5.437900000	-0.740566000
7	2.809586000	-1.711375000	-1.238944000
6	3.407118000	1.536832000	0.756231000
6	3.202774000	2.950757000	0.671675000
6	2.634913000	3.601365000	-0.454090000
1	2.376796000	3.037797000	-1.341478000
6	2.428537000	4.957385000	-0.451997000
1	1.993657000	5.434813000	-1.322827000

6	2.781917000	5.737973000	0.671578000
1	2.608101000	6.807971000	0.659449000
6	3.349403000	5.142658000	1.766469000
1	3.639353000	5.712550000	2.642049000
6	3.573416000	3.743802000	1.792304000
6	4.319672000	1.927231000	2.954963000
1	4.763998000	1.519473000	3.859225000
6	3.977098000	1.044621000	1.900502000
1	4.171389000	-0.009738000	2.051440000
7	4.127289000	3.218149000	2.924036000
6	-6.054404000	1.129061000	-2.328413000
9	-5.772631000	0.497907000	-3.479572000
9	-7.366642000	0.981996000	-2.112203000
9	-5.834285000	2.434315000	-2.548078000
1	1.764650000	-1.829529000	-0.944079000
8	0.327644000	-2.146763000	-0.545725000
1	-0.122336000	-2.523523000	-1.308211000
8	0.053214000	-1.141678000	1.601124000
6	0.598244000	-0.899567000	2.813172000
6	0.785994000	0.326837000	3.325439000
1	1.305019000	0.385051000	4.277718000
6	0.368529000	1.580503000	2.709875000
1	-0.250873000	1.524126000	1.817762000
1	1.321131000	2.906129000	4.059773000
6	0.702417000	2.787871000	3.174124000
1	0.370388000	3.694400000	2.679455000
1	0.951580000	-1.772411000	3.356198000
14	-0.453116000	-2.790500000	1.063246000
6	0.886344000	-4.124795000	1.215452000
1	1.160106000	-4.568465000	0.255285000
1	1.796613000	-3.668454000	1.627471000
1	0.599687000	-4.926318000	1.901845000
6	-2.041443000	-3.142544000	0.048888000
1	-2.248592000	-2.403108000	-0.729747000
1	-1.906447000	-4.103889000	-0.465544000

1	-2.936542000	-3.240482000	0.666801000
6	-1.313060000	-3.143031000	2.780619000
1	-1.838301000	-4.103904000	2.701689000
1	-0.646437000	-3.229191000	3.646136000
1	-2.074215000	-2.394282000	3.039531000

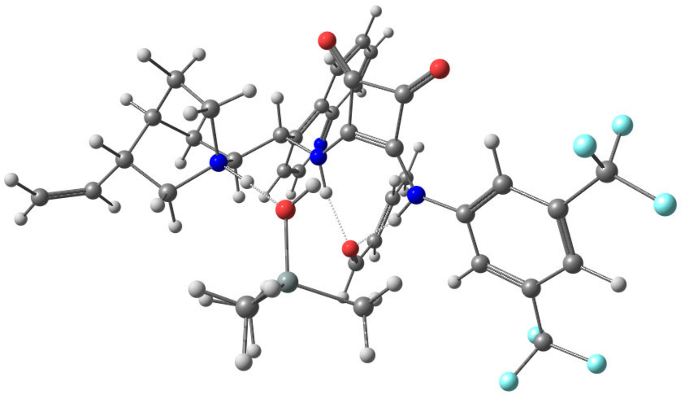

Z-Hyd-3

Sum of electronic and thermal Free Energies= -2880.829740

(0.0 kcal/mol)

6	-2.831547000	0.586318000	0.307234000
1	-3.065369000	0.751843000	1.360834000
6	-0.530431000	0.468535000	1.180482000
6	-0.604060000	0.676680000	2.632548000
6	0.910215000	0.613549000	2.671828000
6	0.871162000	0.395408000	1.206603000
6	3.084610000	0.159152000	0.148106000
6	3.866593000	0.188296000	1.304109000
1	3.406449000	0.283999000	2.282499000
6	5.248581000	0.097648000	1.192233000
6	5.885311000	-0.030819000	-0.034369000
1	6.962020000	-0.110468000	-0.101063000
6	5.089872000	-0.053064000	-1.174271000
6	3.710910000	0.048593000	-1.098568000
1	3.108981000	0.041861000	-2.002779000
6	5.702088000	-0.214479000	-2.534277000
9	7.038831000	-0.253822000	-2.494221000
9	5.360342000	0.785954000	-3.359819000
9	5.293096000	-1.349621000	-3.126620000
7	-1.401007000	0.344076000	0.185263000
1	-1.000113000	0.143611000	-0.756116000
7	1.695275000	0.205159000	0.159239000

1	1.210493000	0.072216000	-0.755156000
8	-1.497514000	0.821447000	3.439908000
8	1.744294000	0.694563000	3.542027000
6	-3.587603000	-0.656304000	-0.174278000
1	-3.316820000	-0.852099000	-1.213528000
6	-5.118280000	-0.573741000	0.008671000
1	-5.618922000	-0.708048000	-0.951338000
1	-5.391889000	0.418177000	0.375605000
6	-5.569106000	-1.629542000	1.027417000
1	-6.636236000	-1.523233000	1.224792000
6	-4.762287000	-1.426605000	2.316092000
1	-5.154265000	-2.050650000	3.119935000
1	-4.831705000	-0.385414000	2.640665000
6	-3.298695000	-1.806377000	2.041189000
1	-2.591990000	-1.091592000	2.458712000
1	-3.045002000	-2.795291000	2.421954000
6	-3.809637000	-3.076887000	0.014381000
1	-3.273422000	-3.962062000	0.355700000
1	-3.738329000	-3.019932000	-1.072227000
6	-5.276054000	-3.053301000	0.501667000
1	-5.384046000	-3.748283000	1.339476000
6	-6.206274000	-3.484753000	-0.594797000
1	-6.198076000	-2.879391000	-1.500357000
6	-7.013023000	-4.536211000	-0.516726000
1	-7.040613000	-5.165938000	0.368578000
1	-7.675050000	-4.804560000	-1.333075000
7	-3.089447000	-1.877486000	0.555032000
6	-3.197148000	1.833882000	-0.490759000
6	-3.054600000	3.130649000	0.096963000
6	-2.636873000	3.355320000	1.435111000
1	-2.423702000	2.522672000	2.094267000
6	-2.496254000	4.630208000	1.921822000
1	-2.170079000	4.780156000	2.944810000
6	-2.772494000	5.748517000	1.103664000
1	-2.655917000	6.749520000	1.503331000

6	-3.183366000	5.564958000	-0.189724000
1	-3.398216000	6.400616000	-0.846229000
6	-3.327111000	4.260531000	-0.723463000
6	-3.806612000	2.956807000	-2.535377000
1	-4.093668000	2.887253000	-3.581294000
6	-3.572526000	1.765269000	-1.806050000
1	-3.683850000	0.823639000	-2.329200000
7	-3.700780000	4.157014000	-2.032027000
6	6.049390000	0.161954000	2.459772000
9	5.597467000	-0.705864000	3.378902000
9	7.344642000	-0.112622000	2.263131000
9	5.988313000	1.377976000	3.025203000
1	-2.069896000	-2.001317000	0.392611000
8	-0.427461000	-2.644222000	0.559237000
1	0.123094000	-2.294543000	1.268427000
8	0.096345000	-0.277905000	-1.991614000
6	-0.066844000	0.419640000	-3.079542000
6	-0.177177000	1.774521000	-3.192120000
1	-0.337129000	2.193625000	-4.182174000
6	-0.099969000	2.684264000	-2.065475000
1	0.132066000	2.236576000	-1.099540000
1	-0.529851000	4.526937000	-3.029990000
6	-0.285192000	4.011506000	-2.104265000
1	-0.205872000	4.619340000	-1.208923000
1	-0.134208000	-0.162206000	-4.011370000
14	0.299610000	-3.622779000	-0.624521000
6	-0.898771000	-3.583090000	-2.048658000
1	-1.824603000	-4.125611000	-1.842001000
1	-1.126261000	-2.542389000	-2.299156000
1	-0.446838000	-4.040209000	-2.934733000
6	0.476949000	-5.349150000	0.068873000
1	1.154685000	-5.372423000	0.927603000
1	-0.486979000	-5.752170000	0.393810000
1	0.882487000	-6.030790000	-0.685360000
6	1.956558000	-2.924737000	-1.106678000

1	2.497868000	-3.628780000	-1.746946000
1	1.801845000	-2.001603000	-1.669058000
1	2.594744000	-2.713410000	-0.243114000

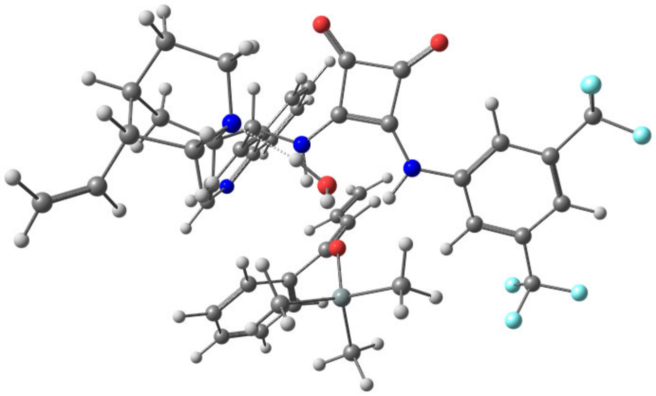

Hyd-K-1

Sum of electronic and thermal Free Energies= -3111.767629

(0.0 kcal/mol)

6	-2.539156000	-1.306977000	0.724445000
1	-2.566823000	-2.227015000	1.313040000
6	-0.142542000	-1.874029000	0.676771000
6	-0.007437000	-3.168936000	1.368404000
6	1.485510000	-3.094540000	1.105299000
6	1.233934000	-1.795688000	0.453837000
6	3.296621000	-0.584468000	-0.159757000
6	4.215612000	-1.618859000	0.027382000
1	3.874386000	-2.624535000	0.248741000
6	5.573729000	-1.343546000	-0.062419000
6	6.053739000	-0.070640000	-0.344098000
1	7.115063000	0.123073000	-0.422922000
6	5.123961000	0.945478000	-0.520434000
6	3.762603000	0.706914000	-0.419625000
1	3.056847000	1.525119000	-0.531329000
6	5.562987000	2.350517000	-0.807260000
9	6.883743000	2.450140000	-0.986849000
9	5.235067000	3.187771000	0.189760000
9	4.974347000	2.836156000	-1.914134000
7	-1.139263000	-1.040339000	0.410067000
1	-0.939795000	-0.244412000	-0.193924000
7	1.918428000	-0.778620000	-0.112836000
1	1.342704000	-0.038735000	-0.513206000

8	-0.758110000	-3.931354000	1.932990000
8	2.431959000	-3.806428000	1.346113000
6	-3.348305000	-1.524680000	-0.563836000
1	-3.338445000	-0.583529000	-1.127676000
6	-4.805546000	-1.961915000	-0.266601000
1	-5.496829000	-1.123374000	-0.367796000
1	-4.892958000	-2.319876000	0.765078000
6	-5.172039000	-3.100600000	-1.228148000
1	-6.231222000	-3.348024000	-1.131870000
6	-4.299235000	-4.315920000	-0.892581000
1	-4.489007000	-5.119304000	-1.609406000
1	-4.555921000	-4.695909000	0.099295000
6	-2.813623000	-3.874417000	-0.938316000
1	-2.354886000	-3.922780000	0.049512000
1	-2.224998000	-4.521358000	-1.591468000
6	-3.335425000	-2.429254000	-2.774645000
1	-2.857112000	-3.172016000	-3.417158000
1	-3.132150000	-1.443770000	-3.204563000
6	-4.871718000	-2.680708000	-2.685366000
1	-5.143256000	-3.516512000	-3.336979000
6	-5.662330000	-1.476828000	-3.110433000
1	-5.386507000	-0.528345000	-2.647162000
6	-6.652480000	-1.497070000	-3.996156000
1	-6.953292000	-2.419119000	-4.487186000
1	-7.197314000	-0.597672000	-4.264083000
7	-2.684991000	-2.498251000	-1.455366000
6	-3.059870000	-0.159149000	1.586512000
6	-2.616598000	-0.027384000	2.943378000
6	-1.763830000	-0.959482000	3.589089000
1	-1.426658000	-1.845532000	3.066040000
6	-1.380948000	-0.776490000	4.893782000
1	-0.732700000	-1.503785000	5.369474000
6	-1.833030000	0.343405000	5.626382000
1	-1.524101000	0.472224000	6.657683000
6	-2.658723000	1.260583000	5.032352000

1	-3.022305000	2.132610000	5.564578000
6	-3.062641000	1.101176000	3.684056000
6	-4.253277000	1.914836000	1.913956000
1	-4.888141000	2.693205000	1.497428000
6	-3.889764000	0.813358000	1.097429000
1	-4.278871000	0.787437000	0.088375000
7	-3.867193000	2.069356000	3.151209000
6	6.535297000	-2.470306000	0.183283000
9	6.162012000	-3.593703000	-0.446104000
9	7.775894000	-2.180246000	-0.228840000
9	6.619427000	-2.773694000	1.488373000
1	-1.031000000	-1.838859000	-1.936306000
8	-0.241877000	-1.332729000	-2.237437000
1	-0.161146000	-1.460805000	-3.188366000
8	-0.005756000	1.413887000	-0.951531000
6	-0.380886000	2.221505000	0.099586000
6	0.175336000	1.995857000	1.302875000
1	0.964742000	1.249200000	1.356606000
6	-0.218366000	2.600998000	2.567452000
1	-1.108732000	3.224453000	2.581195000
1	1.334432000	1.757937000	3.733374000
6	0.447460000	2.385191000	3.705557000
1	0.124282000	2.827195000	4.640984000
14	0.224634000	1.844387000	-2.603794000
6	1.742946000	0.918745000	-3.175279000
1	2.664732000	1.432140000	-2.890485000
1	1.783439000	-0.106010000	-2.800034000
1	1.736275000	0.867200000	-4.269267000
6	0.562196000	3.680423000	-2.683106000
1	-0.338289000	4.290779000	-2.583614000
1	1.270500000	3.990234000	-1.907304000
1	1.019576000	3.917900000	-3.649621000
6	-1.270554000	1.372787000	-3.623680000
1	-0.955177000	1.127916000	-4.643194000
1	-1.754292000	0.484744000	-3.211775000

1	-2.004966000	2.178390000	-3.691153000
6	-1.449692000	3.206251000	-0.185186000
6	-1.376241000	4.517926000	0.292275000
6	-2.541574000	2.832078000	-0.974029000
6	-2.384322000	5.429668000	-0.001336000
6	-3.546941000	3.744180000	-1.271054000
6	-3.470911000	5.046427000	-0.783171000
1	-0.514248000	4.829323000	0.872117000
1	-2.609899000	1.815113000	-1.344998000
1	-2.313779000	6.445743000	0.370118000
1	-4.391107000	3.438577000	-1.880033000
1	-4.252487000	5.760979000	-1.015609000

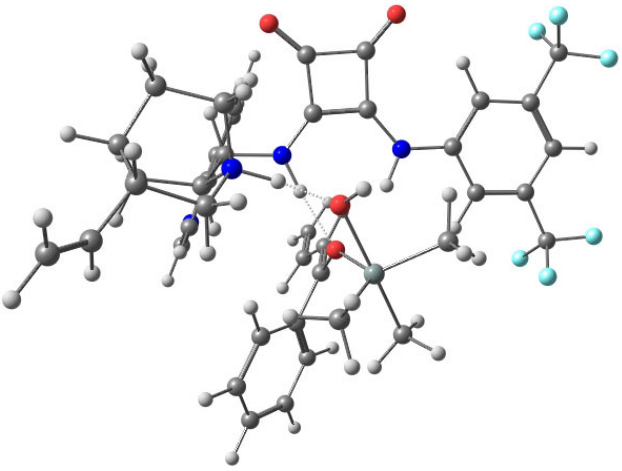

Hyd-K-2

Sum of electronic and thermal Free Energies= -3111.741750

(16.2 kcal/mol)

6	-2.490246000	-1.542322000	0.319056000
1	-2.591495000	-2.630219000	0.269284000
6	-0.092737000	-2.049998000	-0.072790000
6	0.065246000	-3.509560000	-0.172305000
6	1.556971000	-3.285305000	-0.346510000
6	1.277493000	-1.837449000	-0.244912000
6	3.295412000	-0.401782000	-0.278690000
6	4.246104000	-1.402134000	-0.482901000
1	3.943029000	-2.436043000	-0.614202000
6	5.592144000	-1.059256000	-0.519335000
6	6.027091000	0.250101000	-0.365863000
1	7.078779000	0.500112000	-0.406406000
6	5.063816000	1.230047000	-0.160938000
6	3.715016000	0.920333000	-0.108219000
1	2.982721000	1.703615000	0.064930000
6	5.453497000	2.667649000	0.018769000
9	6.768841000	2.864014000	-0.120441000
9	5.111888000	3.126027000	1.233356000
9	4.838043000	3.463757000	-0.871426000
7	-1.090995000	-1.197682000	0.114976000

1	-0.882672000	-0.201761000	-0.029016000
7	1.924837000	-0.651633000	-0.248606000
1	1.323829000	0.170779000	-0.224833000
8	-0.682378000	-4.460285000	-0.134898000
8	2.514790000	-4.003646000	-0.503762000
6	-3.333377000	-0.893847000	-0.788658000
1	-3.295387000	0.187585000	-0.652539000
6	-4.789413000	-1.406039000	-0.832856000
1	-5.489699000	-0.575445000	-0.728548000
1	-4.967593000	-2.080547000	0.008235000
6	-5.015808000	-2.161856000	-2.149662000
1	-6.006027000	-2.618874000	-2.151951000
6	-3.927428000	-3.234935000	-2.283599000
1	-4.138393000	-3.894066000	-3.126605000
1	-3.898722000	-3.850418000	-1.380760000
6	-2.571720000	-2.536445000	-2.498867000
1	-1.780606000	-2.976254000	-1.896359000
1	-2.253031000	-2.560114000	-3.540927000
6	-3.564970000	-0.412025000	-3.158068000
1	-2.983911000	-0.343595000	-4.077430000
1	-3.746070000	0.600550000	-2.793726000
6	-4.880804000	-1.198328000	-3.350079000
1	-4.807828000	-1.803646000	-4.258455000
6	-6.047181000	-0.265018000	-3.497860000
1	-6.224828000	0.422215000	-2.671206000
6	-6.850058000	-0.235611000	-4.555024000
1	-6.697885000	-0.900090000	-5.401414000
1	-7.687130000	0.452011000	-4.612965000
7	-2.707742000	-1.092493000	-2.137507000
6	-2.909323000	-1.051619000	1.707606000
6	-2.389151000	-1.702701000	2.871734000
6	-1.597169000	-2.879798000	2.830424000
1	-1.401155000	-3.374765000	1.887866000
6	-1.096490000	-3.431171000	3.981885000
1	-0.495604000	-4.331964000	3.928647000

6	-1.361594000	-2.839143000	5.237440000
1	-0.951322000	-3.281909000	6.137922000
6	-2.143850000	-1.717914000	5.313415000
1	-2.377274000	-1.245526000	6.261020000
6	-2.680109000	-1.130207000	4.140434000
6	-3.927843000	0.538320000	3.212474000
1	-4.539294000	1.427635000	3.343049000
6	-3.685882000	0.061013000	1.899478000
1	-4.125391000	0.620342000	1.083673000
7	-3.448968000	-0.013152000	4.294225000
6	6.586833000	-2.167910000	-0.705964000
9	6.243541000	-2.982324000	-1.714768000
9	7.817860000	-1.711961000	-0.967872000
9	6.681238000	-2.938787000	0.389594000
1	-1.746466000	-0.574075000	-2.167118000
8	-0.496470000	0.288769000	-2.343061000
1	-0.084492000	0.037151000	-3.176136000
8	-0.393320000	1.446446000	-0.299479000
6	-0.666241000	2.040778000	0.893840000
6	-0.172096000	1.475728000	2.016592000
1	0.525937000	0.649462000	1.892126000
6	-0.572615000	1.760607000	3.386346000
1	-1.365906000	2.485475000	3.545660000
1	0.729606000	0.383293000	4.333934000
6	-0.054659000	1.127828000	4.444102000
1	-0.409454000	1.327201000	5.449250000
14	0.027312000	2.087264000	-1.925096000
6	1.733913000	1.702076000	-2.707708000
1	2.539203000	2.328666000	-2.317647000
1	2.047690000	0.657484000	-2.638682000
1	1.658269000	1.934015000	-3.779119000
6	0.564734000	3.822532000	-1.229984000
1	-0.257638000	4.480465000	-0.932744000
1	1.261976000	3.766785000	-0.382223000
1	1.099429000	4.338659000	-2.038730000

6	-1.298657000	2.719296000	-3.136618000
1	-1.150950000	2.277768000	-4.128412000
1	-2.304300000	2.426184000	-2.821987000
1	-1.293391000	3.807678000	-3.245354000
6	-1.629181000	3.167852000	0.879293000
6	-1.437415000	4.297065000	1.680472000
6	-2.729971000	3.130578000	0.018655000
6	-2.336709000	5.356318000	1.632534000
6	-3.630080000	4.188514000	-0.029675000
6	-3.435704000	5.305406000	0.779143000
1	-0.559522000	4.359149000	2.314265000
1	-2.872493000	2.269646000	-0.625133000
1	-2.168736000	6.231251000	2.250591000
1	-4.479060000	4.146325000	-0.703389000
1	-4.131447000	6.135989000	0.737268000

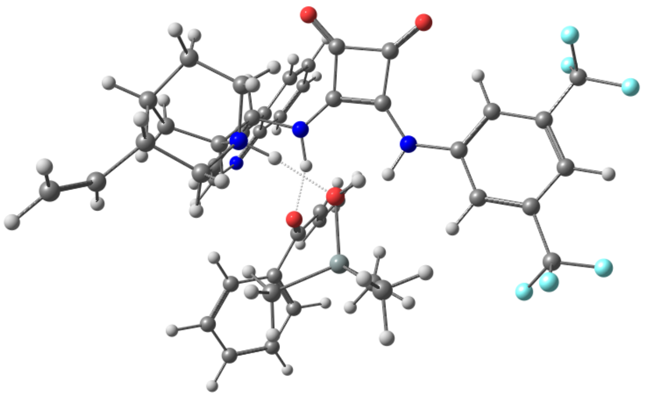

Hyd-K-3

Sum of electronic and thermal Free Energies= -3111.757037

(6.6 kcal/mol)

6	2.331955000	-1.699510000	0.548972000
1	2.307389000	-2.679065000	1.036534000
6	-0.118285000	-1.955122000	0.597175000
6	-0.388423000	-3.279753000	1.192052000
6	-1.869064000	-3.000847000	1.036791000
6	-1.489357000	-1.675238000	0.513543000
6	-3.431847000	-0.208070000	0.068575000
6	-4.434069000	-1.171548000	0.193739000
1	-4.183134000	-2.215385000	0.348712000
6	-5.765158000	-0.777275000	0.141220000
6	-6.140165000	0.547438000	-0.036454000
1	-7.182162000	0.835339000	-0.076120000
6	-5.128051000	1.490443000	-0.164458000
6	-3.791657000	1.130294000	-0.116983000
1	-3.021827000	1.886812000	-0.227374000
6	-5.455328000	2.948062000	-0.303351000
9	-6.737768000	3.157273000	-0.615719000
9	-4.710945000	3.541181000	-1.248458000
9	-5.216570000	3.617144000	0.839391000
7	0.962316000	-1.259947000	0.292172000
1	0.927826000	-0.276841000	-0.114266000

7	-2.074397000	-0.519897000	0.116770000
1	-1.432305000	0.255399000	-0.022336000
8	0.293035000	-4.168539000	1.650831000
8	-2.881309000	-3.620588000	1.267112000
6	3.003674000	-0.666570000	1.472152000
1	3.103258000	0.261771000	0.908525000
6	4.338260000	-1.137731000	2.086083000
1	5.136754000	-0.434820000	1.841851000
1	4.618774000	-2.102143000	1.656121000
6	4.179336000	-1.282875000	3.605857000
1	5.083308000	-1.714157000	4.036732000
6	2.970405000	-2.185333000	3.879893000
1	2.912713000	-2.442807000	4.938070000
1	3.062607000	-3.116327000	3.315678000
6	1.693400000	-1.435749000	3.466859000
1	1.003264000	-2.069408000	2.917499000
1	1.163219000	-1.010526000	4.318655000
6	2.768463000	0.761103000	3.435556000
1	2.014348000	1.224817000	4.070597000
1	3.155243000	1.510805000	2.745278000
6	3.896898000	0.092864000	4.250945000
1	3.546083000	-0.080943000	5.272408000
6	5.103089000	0.984690000	4.311540000
1	5.558778000	1.247544000	3.357468000
6	5.621975000	1.456985000	5.438547000
1	5.186482000	1.221721000	6.406065000
1	6.500257000	2.093664000	5.435513000
7	2.078212000	-0.275056000	2.596665000
6	3.069048000	-1.794572000	-0.789160000
6	2.625152000	-2.730739000	-1.776772000
6	1.675443000	-3.754349000	-1.523592000
1	1.302089000	-3.910224000	-0.520042000
6	1.257946000	-4.590848000	-2.526480000
1	0.538008000	-5.372082000	-2.310006000
6	1.763560000	-4.448107000	-3.838977000

1	1.413034000	-5.107037000	-4.625277000
6	2.703972000	-3.490437000	-4.108586000
1	3.124444000	-3.362303000	-5.099641000
6	3.167832000	-2.624644000	-3.086132000
6	4.560510000	-0.925883000	-2.476545000
1	5.332341000	-0.210288000	-2.748667000
6	4.066743000	-0.928560000	-1.148069000
1	4.481983000	-0.200197000	-0.465308000
7	4.124726000	-1.711853000	-3.423368000
6	-6.808859000	-1.851028000	0.255909000
9	-6.617864000	-2.615719000	1.341214000
9	-8.048449000	-1.353049000	0.337613000
9	-6.787214000	-2.677700000	-0.801213000
1	1.231267000	0.163678000	2.184542000
8	-0.223704000	1.210590000	1.715395000
1	-0.944309000	1.043063000	2.335408000
8	1.436125000	1.151388000	-0.547970000
6	1.640369000	1.282269000	-1.821720000
6	1.112883000	0.436593000	-2.770067000
1	0.442732000	-0.341818000	-2.403404000
6	1.454011000	0.378127000	-4.173436000
1	2.225909000	1.059241000	-4.526890000
1	0.168858000	-1.205106000	-4.769845000
6	0.930325000	-0.485100000	-5.060391000
1	1.257735000	-0.503326000	-6.094109000
14	-0.139064000	2.843071000	1.194302000
6	-1.342330000	3.732182000	2.331936000
1	-1.340798000	4.806337000	2.119182000
1	-2.372365000	3.384573000	2.194411000
1	-1.086117000	3.610251000	3.389167000
6	-0.727276000	3.110998000	-0.565556000
1	0.038490000	3.664090000	-1.118383000
1	-0.891054000	2.190104000	-1.129379000
1	-1.645059000	3.707263000	-0.593357000
6	1.578129000	3.490854000	1.508656000

1	1.783879000	3.591186000	2.578327000
1	2.319786000	2.838616000	1.048059000
1	1.688918000	4.482917000	1.057925000
6	2.518999000	2.441404000	-2.199512000
6	2.199358000	3.315914000	-3.242405000
6	3.654951000	2.710756000	-1.429487000
6	3.008825000	4.413848000	-3.523308000
6	4.473619000	3.797087000	-1.715970000
6	4.151945000	4.653797000	-2.766733000
1	1.298325000	3.146521000	-3.822388000
1	3.888693000	2.055447000	-0.597087000
1	2.740663000	5.087082000	-4.330423000
1	5.358624000	3.982993000	-1.116513000
1	4.782839000	5.507930000	-2.987362000

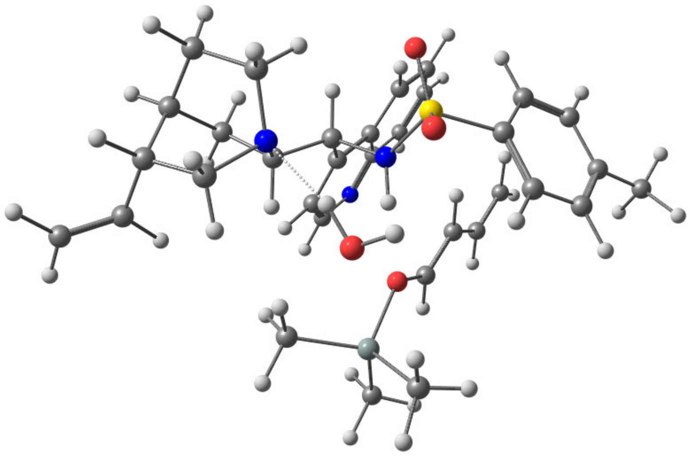

Hyd-S-1

Sum of electronic and thermal Free Energies= -2436.443658

(0.0 kcal/mol)

6	0.915315000	1.149729000	-0.069403000
1	0.862023000	2.149389000	-0.502720000
7	0.070059000	0.278226000	-0.888687000
1	0.141154000	-0.722688000	-0.691597000
6	2.389374000	0.717160000	-0.043117000
1	2.448436000	-0.268629000	0.429449000
6	3.251698000	1.739751000	0.742090000
1	3.466948000	1.378015000	1.749450000
1	2.712924000	2.687064000	0.857019000
6	4.540777000	1.990270000	-0.050914000
1	5.224607000	2.615505000	0.527280000
6	4.167709000	2.675107000	-1.372582000
1	5.061788000	2.802997000	-1.988998000
1	3.764899000	3.671475000	-1.173221000
6	3.115680000	1.796751000	-2.101495000
1	2.153908000	2.304232000	-2.175775000
1	3.427073000	1.565708000	-3.121799000
6	4.236779000	-0.148820000	-1.290601000
1	4.635930000	-0.254623000	-2.301493000
1	4.082020000	-1.157397000	-0.898638000
6	5.225124000	0.645637000	-0.384235000

1	6.139876000	0.864133000	-0.943562000
6	5.598704000	-0.134444000	0.843348000
1	4.772504000	-0.552990000	1.420331000
6	6.844402000	-0.352903000	1.251063000
1	7.696397000	0.034927000	0.698184000
1	7.059936000	-0.926432000	2.146728000
7	2.925149000	0.513322000	-1.401693000
6	0.286954000	1.193052000	1.320262000
6	-0.879002000	1.988003000	1.555166000
6	-1.490459000	2.802467000	0.564716000
1	-1.079984000	2.841798000	-0.437301000
6	-2.596882000	3.557159000	0.863177000
1	-3.047268000	4.177486000	0.095707000
6	-3.153607000	3.540279000	2.162943000
1	-4.024598000	4.147192000	2.383741000
6	-2.599348000	2.749206000	3.132716000
1	-3.010580000	2.698370000	4.134686000
6	-1.462491000	1.950074000	2.851020000
6	0.073281000	0.436705000	3.599684000
1	0.442189000	-0.191325000	4.407131000
6	0.751362000	0.428949000	2.356821000
1	1.620526000	-0.208224000	2.255280000
7	-0.983960000	1.161132000	3.856931000
16	-0.426265000	0.638726000	-2.410658000
8	0.210851000	-0.210306000	-3.410762000
8	-0.329649000	2.085303000	-2.558463000
6	-2.132695000	0.161000000	-2.334178000
6	-2.448066000	-1.193223000	-2.377711000
6	-3.101493000	1.126837000	-2.096158000
6	-3.761777000	-1.580867000	-2.154833000
6	-4.413476000	0.718443000	-1.888527000
6	-4.757821000	-0.635158000	-1.893255000
1	-1.680139000	-1.935527000	-2.568521000
1	-2.835931000	2.177249000	-2.080673000
1	-4.016641000	-2.635447000	-2.173966000

1	-5.180276000	1.464291000	-1.705330000
6	-6.161856000	-1.069762000	-1.579612000
1	-6.432394000	-1.972828000	-2.129348000
1	-6.250633000	-1.292546000	-0.511547000
1	-6.883870000	-0.287172000	-1.817072000
8	-0.374315000	-2.176313000	0.738120000
6	-1.511866000	-1.938873000	1.436569000
6	-2.386021000	-0.972459000	1.135372000
1	-2.207808000	-0.314306000	0.290038000
6	-3.582732000	-0.764884000	1.937705000
1	-3.731705000	-1.446883000	2.773974000
1	-4.351414000	0.906213000	0.897195000
6	-4.479940000	0.199393000	1.712275000
1	-5.352636000	0.322008000	2.344659000
1	-1.684436000	-2.586178000	2.295394000
14	0.582721000	-3.580955000	0.910936000
6	2.361465000	-3.057304000	1.061966000
1	2.696572000	-2.589787000	0.135892000
1	2.516671000	-2.372405000	1.900589000
1	2.988392000	-3.937601000	1.239585000
6	0.276987000	-4.678081000	-0.561487000
1	-0.791763000	-4.875244000	-0.689928000
1	0.659995000	-4.219163000	-1.474399000
1	0.777287000	-5.643033000	-0.432184000
6	0.054508000	-4.456493000	2.478632000
1	0.720232000	-5.310613000	2.641048000
1	0.130652000	-3.816858000	3.363163000
1	-0.964757000	-4.849117000	2.429800000
8	1.704741000	-2.018035000	-1.897068000
1	2.061678000	-1.105502000	-1.896440000
1	1.129082000	-2.031930000	-2.669336000

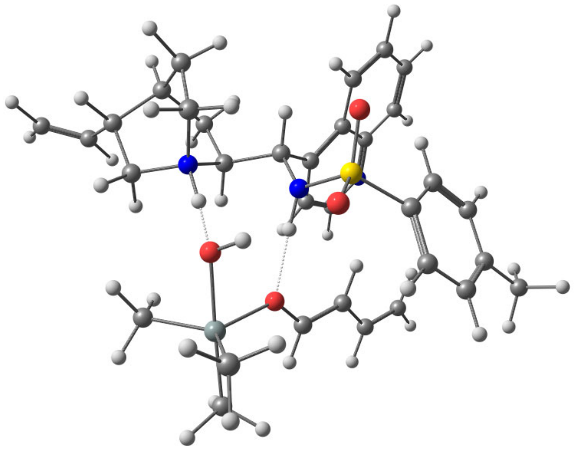

Hyd-S-2

Sum of electronic and thermal Free Energies= -2436.421744

(13.8 kcal/mol)

6	-0.581139000	-1.163868000	-0.293137000
1	-0.700096000	-1.854588000	-1.124774000
7	-0.184275000	0.116010000	-0.856385000
1	-0.249290000	0.969123000	-0.280277000
6	-1.951452000	-0.993108000	0.381422000
1	-1.843471000	-0.354742000	1.259857000
6	-2.586414000	-2.353247000	0.753977000
1	-2.835269000	-2.379714000	1.815710000
1	-1.868787000	-3.158793000	0.576172000
6	-3.832141000	-2.580948000	-0.114373000
1	-4.229432000	-3.582228000	0.057449000
6	-3.438972000	-2.403966000	-1.587578000
1	-4.262634000	-2.696317000	-2.240498000
1	-2.587564000	-3.046756000	-1.825529000
6	-3.087257000	-0.923203000	-1.826885000
1	-2.179854000	-0.791270000	-2.414538000
1	-3.889250000	-0.384222000	-2.331201000
6	-4.219105000	-0.142586000	0.181423000
1	-4.810075000	0.600013000	-0.353419000
1	-4.024807000	0.236670000	1.186184000
6	-4.910915000	-1.524527000	0.212726000

1	-5.668000000	-1.564282000	-0.576344000
6	-5.592872000	-1.754263000	1.529932000
1	-4.959715000	-1.721043000	2.415903000
6	-6.893573000	-1.984468000	1.666148000
1	-7.557610000	-2.014356000	0.806246000
1	-7.341853000	-2.150219000	2.640097000
7	-2.898576000	-0.244752000	-0.509583000
6	0.445440000	-1.716647000	0.689697000
6	1.265187000	-2.843662000	0.375265000
6	1.173087000	-3.592128000	-0.831210000
1	0.453031000	-3.319966000	-1.592189000
6	2.019266000	-4.643449000	-1.070398000
1	1.938486000	-5.197010000	-1.998919000
6	3.003618000	-5.008576000	-0.122689000
1	3.665558000	-5.841830000	-0.329541000
6	3.120869000	-4.308089000	1.046921000
1	3.866927000	-4.559328000	1.792336000
6	2.262183000	-3.213438000	1.324126000
6	1.670813000	-1.533680000	2.758074000
1	1.836439000	-1.012776000	3.697274000
6	0.654571000	-1.080976000	1.885877000
1	0.081730000	-0.206730000	2.172946000
7	2.450860000	-2.552816000	2.501675000
16	0.709482000	0.284959000	-2.211559000
8	0.218976000	1.485899000	-2.876593000
8	0.671195000	-0.998138000	-2.898923000
6	2.368585000	0.616681000	-1.679965000
6	2.782990000	1.935975000	-1.538347000
6	3.164168000	-0.445268000	-1.259483000
6	4.018263000	2.190256000	-0.955994000
6	4.391215000	-0.169340000	-0.672405000
6	4.829146000	1.148320000	-0.501774000
1	2.143318000	2.748675000	-1.862000000
1	2.835344000	-1.469849000	-1.394801000
1	4.344811000	3.217186000	-0.828063000

1	5.014817000	-0.990029000	-0.332226000
6	6.150649000	1.440088000	0.152358000
1	6.927012000	1.591977000	-0.603640000
1	6.095978000	2.346956000	0.757242000
1	6.467154000	0.614904000	0.792221000
8	-0.324611000	2.547527000	0.573040000
6	0.632040000	2.750812000	1.490891000
6	1.725785000	1.978387000	1.605249000
1	1.886955000	1.147257000	0.924212000
6	2.721590000	2.205083000	2.638808000
1	2.555555000	3.062328000	3.290781000
1	3.998522000	0.570332000	2.212108000
6	3.801683000	1.439314000	2.835025000
1	4.512392000	1.652765000	3.626022000
1	0.482256000	3.578440000	2.178392000
14	-1.741509000	3.586994000	0.170977000
6	-3.392049000	3.205056000	1.041015000
1	-4.152282000	2.798613000	0.372045000
1	-3.207335000	2.458945000	1.825628000
1	-3.804824000	4.082117000	1.546583000
6	-1.561354000	4.703620000	-1.363938000
1	-0.714393000	4.355299000	-1.968756000
1	-2.443048000	4.638719000	-2.007939000
1	-1.380432000	5.755138000	-1.128654000
6	-1.253246000	4.989529000	1.450061000
1	-1.993996000	5.787276000	1.308468000
1	-1.310074000	4.707494000	2.509443000
1	-0.271853000	5.450759000	1.281709000
8	-2.151067000	2.171835000	-0.994576000
1	-2.535988000	0.795264000	-0.688682000
1	-1.550895000	2.124358000	-1.750217000

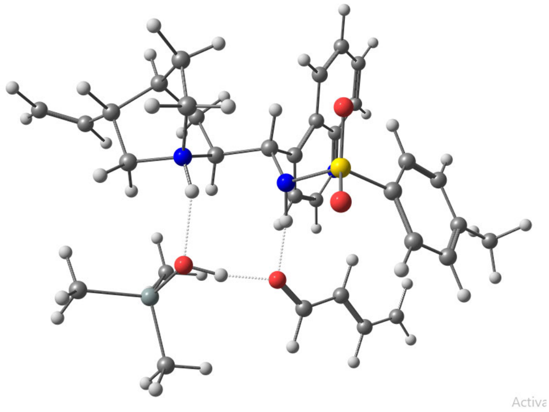

Hyd-S-3

Sum of electronic and thermal Free Energies= -2436.449716

(-3.8 kcal/mol)

6	-0.349676000	1.057745000	0.408279000
1	-0.334470000	1.951071000	1.028279000
7	-0.020756000	-0.068572000	1.282613000
1	0.109921000	-0.999976000	0.815152000
6	-1.780414000	0.859044000	-0.118724000
1	-1.794136000	0.002519000	-0.796529000
6	-2.364756000	2.124896000	-0.781772000
1	-2.747108000	1.888547000	-1.775777000
1	-1.577747000	2.870842000	-0.912378000
6	-3.472289000	2.703556000	0.112546000
1	-3.799017000	3.669034000	-0.274965000
6	-2.924606000	2.854012000	1.538958000
1	-3.632693000	3.398590000	2.164630000
1	-1.992853000	3.424539000	1.524407000
6	-2.692840000	1.451865000	2.130802000
1	-1.746321000	1.358448000	2.658766000
1	-3.492606000	1.148232000	2.805309000
6	-4.092673000	0.327913000	0.462525000
1	-4.678051000	-0.229491000	1.192853000
1	-4.012837000	-0.266596000	-0.446793000
6	-4.668903000	1.728371000	0.174652000
1	-5.309888000	2.032527000	1.007271000

6	-5.497622000	1.706347000	-1.077185000
1	-4.981978000	1.424788000	-1.994960000
6	-6.792767000	1.997064000	-1.116219000
1	-7.339346000	2.272944000	-0.218263000
1	-7.352231000	1.971352000	-2.045228000
7	-2.702291000	0.460805000	1.006053000
6	0.606733000	1.224536000	-0.766080000
6	1.310050000	2.440889000	-1.021219000
6	1.177993000	3.624395000	-0.244056000
1	0.498801000	3.652271000	0.599188000
6	1.914157000	4.743051000	-0.536519000
1	1.802780000	5.634200000	0.070602000
6	2.822486000	4.744967000	-1.620523000
1	3.397741000	5.637729000	-1.838028000
6	2.974878000	3.622047000	-2.387705000
1	3.664826000	3.589438000	-3.223399000
6	2.229879000	2.447440000	-2.108751000
6	1.776994000	0.270587000	-2.642725000
1	1.971480000	-0.591149000	-3.276206000
6	0.839295000	0.155394000	-1.590456000
1	0.349542000	-0.798760000	-1.421411000
7	2.451401000	1.361323000	-2.903162000
16	1.079737000	0.148595000	2.497446000
8	0.809142000	-0.874889000	3.490005000
8	1.011312000	1.557193000	2.870913000
6	2.688410000	-0.184197000	1.816738000
6	3.210654000	-1.466648000	1.923231000
6	3.365343000	0.820014000	1.127103000
6	4.436929000	-1.745227000	1.328902000
6	4.577343000	0.517352000	0.524859000
6	5.129536000	-0.766889000	0.616734000
1	2.665589000	-2.236902000	2.455620000
1	2.961753000	1.824987000	1.078799000
1	4.846096000	-2.747623000	1.398712000
1	5.109453000	1.291633000	-0.019379000

6	6.454691000	-1.068599000	-0.027029000
1	7.269333000	-0.614258000	0.544624000
1	6.635586000	-2.142946000	-0.077839000
1	6.501282000	-0.661247000	-1.039348000
8	0.060220000	-2.523635000	0.179213000
6	0.952397000	-3.255890000	-0.403403000
6	2.164807000	-2.842709000	-0.872954000
1	2.453590000	-1.801954000	-0.746642000
6	3.113915000	-3.738228000	-1.486244000
1	2.811531000	-4.784732000	-1.555328000
1	4.665807000	-2.361119000	-1.927451000
6	4.323260000	-3.392822000	-1.961403000
1	4.990357000	-4.121365000	-2.409186000
1	0.700734000	-4.320970000	-0.535158000
14	-3.299508000	-3.114185000	-0.573895000
6	-5.131325000	-3.016615000	-0.208879000
1	-5.331522000	-3.234712000	0.844390000
1	-5.574615000	-2.045181000	-0.442923000
1	-5.666349000	-3.764962000	-0.802355000
6	-2.741073000	-4.898354000	-0.570580000
1	-1.662844000	-4.974997000	-0.741038000
1	-2.960383000	-5.383489000	0.385111000
1	-3.239969000	-5.472507000	-1.357462000
6	-2.914731000	-2.364138000	-2.250264000
1	-3.389171000	-2.943404000	-3.049269000
1	-3.263188000	-1.331084000	-2.352200000
1	-1.836004000	-2.373860000	-2.439295000
8	-2.482939000	-2.284919000	0.631601000
1	-2.418258000	-0.480551000	1.317576000
1	-1.512091000	-2.490695000	0.594692000

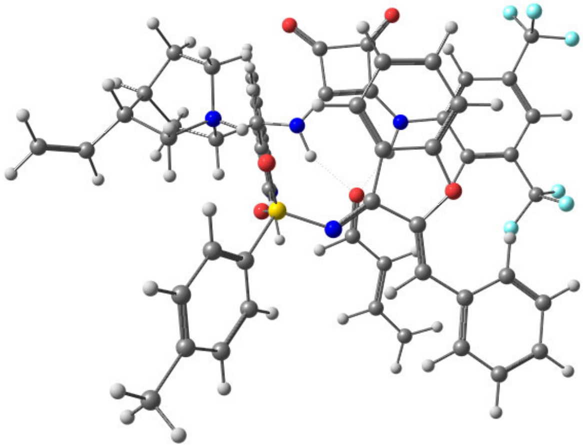

I

Sum of electronic and thermal Free Energies= -3922.529519

(0.0 kcal/mol)

6	-2.654429000	-2.112014000	0.550218000
1	-2.778643000	-3.090244000	0.089458000
6	-0.529362000	-2.395153000	-0.497629000
6	-0.664745000	-3.705117000	-1.131266000
6	0.838445000	-3.723322000	-1.307840000
6	0.867525000	-2.414010000	-0.593869000
6	3.152316000	-1.575426000	-0.239752000
6	3.836401000	-2.503069000	-1.022427000
1	3.299133000	-3.271529000	-1.566806000
6	5.221869000	-2.431842000	-1.111893000
6	5.955158000	-1.470977000	-0.435440000
1	7.034918000	-1.429510000	-0.508541000
6	5.255785000	-0.561194000	0.354405000
6	3.876445000	-0.593519000	0.451070000
1	3.347726000	0.129586000	1.062181000
6	6.042956000	0.484151000	1.086839000
9	7.092208000	-0.045478000	1.732324000
9	5.313366000	1.145970000	1.989781000
9	6.549105000	1.402151000	0.239422000
7	-1.453447000	-1.514774000	-0.052495000
1	-1.069416000	-0.661363000	0.386324000

7	1.765537000	-1.528755000	-0.136298000
1	1.365701000	-0.716567000	0.377657000
8	-1.584967000	-4.448312000	-1.404684000
8	1.624673000	-4.470707000	-1.834166000
6	-3.919234000	-1.296691000	0.269839000
1	-3.864716000	-0.347258000	0.795245000
6	-5.216781000	-2.058300000	0.617164000
1	-5.805705000	-1.484375000	1.333220000
1	-4.973203000	-3.010980000	1.094335000
6	-6.009858000	-2.327033000	-0.668350000
1	-6.889547000	-2.931451000	-0.444933000
6	-5.091466000	-3.062490000	-1.652941000
1	-5.653047000	-3.404835000	-2.522806000
1	-4.655798000	-3.942004000	-1.172017000
6	-3.982116000	-2.099490000	-2.105175000
1	-2.993955000	-2.554286000	-2.081728000
1	-4.154313000	-1.700767000	-3.104606000
6	-5.204509000	-0.084260000	-1.418666000
1	-5.101142000	0.381374000	-2.396955000
1	-5.202177000	0.697080000	-0.656667000
6	-6.448645000	-0.996478000	-1.323564000
1	-6.798860000	-1.223845000	-2.334663000
6	-7.558703000	-0.307351000	-0.583693000
1	-7.340166000	0.005811000	0.436442000
6	-8.758084000	-0.067531000	-1.100092000
1	-9.006199000	-0.357475000	-2.117665000
1	-9.534525000	0.426104000	-0.525429000
7	-3.975534000	-0.911945000	-1.185563000
6	-2.472431000	-2.260123000	2.053043000
6	-2.478425000	-3.525135000	2.710064000
6	-2.663981000	-4.771866000	2.050561000
1	-2.784471000	-4.812897000	0.974415000
6	-2.665436000	-5.943690000	2.760585000
1	-2.800571000	-6.886021000	2.242036000
6	-2.482921000	-5.938119000	4.163549000

1	-2.487702000	-6.875113000	4.708752000
6	-2.290922000	-4.756728000	4.826349000
1	-2.138107000	-4.720471000	5.899027000
6	-2.279040000	-3.525734000	4.121151000
6	-2.050336000	-1.250174000	4.196693000
1	-1.867713000	-0.355442000	4.785814000
6	-2.253826000	-1.133503000	2.804019000
1	-2.223613000	-0.149500000	2.345431000
7	-2.068524000	-2.388988000	4.842596000
6	5.900917000	-3.406864000	-2.027159000
9	5.577280000	-3.174397000	-3.312779000
9	7.234908000	-3.348115000	-1.945485000
9	5.542463000	-4.672796000	-1.770761000
8	0.299411000	0.447965000	0.931046000
6	0.177111000	1.343242000	1.842364000
6	1.188682000	1.959274000	2.534970000
1	2.221053000	1.673863000	2.339924000
6	0.934134000	2.973476000	3.526224000
1	-0.116133000	3.213240000	3.702506000
1	2.926581000	3.447051000	4.083651000
6	1.866678000	3.644735000	4.223825000
1	1.597516000	4.401596000	4.952565000
1	-0.849371000	1.678204000	2.063859000
1	-3.156421000	-0.331714000	-1.398752000
6	-0.036549000	-0.007825000	-2.816825000
6	0.665131000	0.961226000	-2.084162000
6	2.066932000	0.945306000	-2.126791000
6	2.801235000	0.039212000	-2.873959000
6	2.083683000	-0.918187000	-3.575734000
6	0.681672000	-0.942311000	-3.545631000
6	0.321246000	2.084958000	-1.213229000
6	1.606128000	2.615861000	-0.776044000
1	-1.116135000	-0.021120000	-2.821818000
1	3.884647000	0.068921000	-2.877410000
1	2.619802000	-1.663632000	-4.153248000

1	0.151520000	-1.703864000	-4.106248000
8	2.635950000	1.902892000	-1.349833000
6	1.767018000	3.612378000	0.114821000
1	0.834021000	4.063589000	0.435621000
6	2.983632000	4.193395000	0.658597000
6	2.850537000	5.339245000	1.459160000
6	4.266969000	3.661887000	0.448661000
6	3.964287000	5.950930000	2.018562000
6	5.375729000	4.269847000	1.020120000
6	5.230183000	5.416464000	1.800019000
1	1.860601000	5.744586000	1.639448000
1	4.393311000	2.772378000	-0.154794000
1	3.843197000	6.836952000	2.631551000
1	6.359861000	3.845345000	0.860303000
1	6.102688000	5.886310000	2.240315000
7	-0.761579000	2.701961000	-0.816510000
8	-2.560212000	1.450098000	-2.229635000
8	-2.637987000	1.385529000	0.231074000
16	-2.268256000	2.152033000	-0.967399000
6	-3.185227000	3.668162000	-0.963067000
6	-4.005679000	3.966125000	-2.039947000
6	-3.096457000	4.510473000	0.144364000
6	-4.754451000	5.139835000	-2.006470000
6	-3.848151000	5.672881000	0.156890000
6	-4.687183000	6.004604000	-0.915892000
1	-4.052845000	3.298502000	-2.891929000
1	-2.447726000	4.260416000	0.977007000
1	-5.397449000	5.383824000	-2.845400000
1	-3.787004000	6.339387000	1.011309000
6	-5.486492000	7.277488000	-0.877964000
1	-6.137513000	7.366202000	-1.748185000
1	-6.105981000	7.320451000	0.021094000
1	-4.824255000	8.146959000	-0.857512000

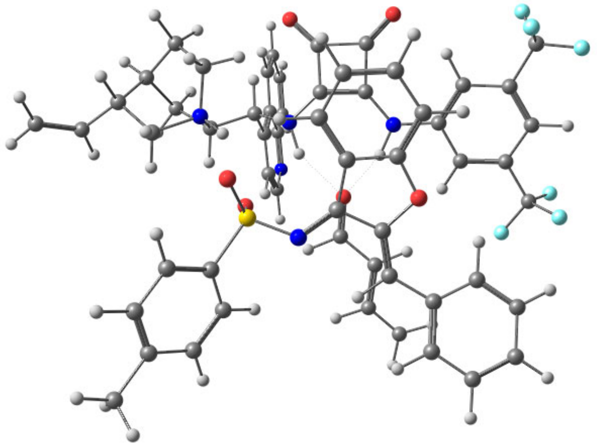

TS1

Sum of electronic and thermal Free Energies= -3922.491643

(3.0 kcal/mol)

6	-2.675824000	-2.023126000	0.582353000
1	-2.763298000	-3.026778000	0.171502000
6	-0.529339000	-2.324897000	-0.422450000
6	-0.659069000	-3.648690000	-1.034957000
6	0.846703000	-3.677947000	-1.182616000
6	0.868329000	-2.357496000	-0.491540000
6	3.163215000	-1.564746000	-0.097974000
6	3.834163000	-2.446234000	-0.941231000
1	3.285363000	-3.153305000	-1.552141000
6	5.223578000	-2.413403000	-0.998115000
6	5.970254000	-1.530714000	-0.237174000
1	7.051890000	-1.512939000	-0.292759000
6	5.282463000	-0.662707000	0.608852000
6	3.902258000	-0.664015000	0.681328000
1	3.384622000	0.014104000	1.349713000
6	6.089160000	0.278526000	1.452096000
9	6.928135000	-0.378381000	2.267453000
9	5.332791000	1.069603000	2.220482000
9	6.856580000	1.080263000	0.690819000
7	-1.463424000	-1.440521000	-0.011302000
1	-1.117401000	-0.561217000	0.391534000

7	1.775705000	-1.491065000	-0.011460000
1	1.392573000	-0.664899000	0.472880000
8	-1.575779000	-4.397253000	-1.301909000
8	1.638196000	-4.441228000	-1.676494000
6	-3.960666000	-1.263115000	0.229548000
1	-3.972660000	-0.305776000	0.743174000
6	-5.234034000	-2.084210000	0.534736000
1	-5.889835000	-1.522764000	1.201179000
1	-4.967728000	-3.008736000	1.053671000
6	-5.944488000	-2.432073000	-0.780146000
1	-6.792660000	-3.088857000	-0.584193000
6	-4.935198000	-3.121517000	-1.708213000
1	-5.435109000	-3.518536000	-2.592493000
1	-4.459007000	-3.959904000	-1.193736000
6	-3.878090000	-2.091467000	-2.136529000
1	-2.864154000	-2.479470000	-2.076754000
1	-4.044601000	-1.714937000	-3.145847000
6	-5.249988000	-0.154450000	-1.523888000
1	-5.129824000	0.317862000	-2.496644000
1	-5.335110000	0.626225000	-0.765954000
6	-6.434792000	-1.144951000	-1.483131000
1	-6.711611000	-1.409858000	-2.507933000
6	-7.626825000	-0.519813000	-0.818538000
1	-7.486920000	-0.178944000	0.206468000
6	-8.808197000	-0.364431000	-1.404172000
1	-8.980283000	-0.683462000	-2.428723000
1	-9.645247000	0.086339000	-0.881734000
7	-3.981144000	-0.899053000	-1.231073000
6	-2.537293000	-2.086164000	2.096659000
6	-2.542827000	-3.313528000	2.821121000
6	-2.680792000	-4.597010000	2.224037000
1	-2.764307000	-4.697540000	1.148162000
6	-2.684583000	-5.729498000	2.995149000
1	-2.783865000	-6.700422000	2.523420000
6	-2.551890000	-5.645944000	4.401285000

1	-2.558018000	-6.552709000	4.995416000
6	-2.407005000	-4.427251000	5.005895000
1	-2.293865000	-4.330757000	6.079777000
6	-2.394043000	-3.235424000	4.236327000
6	-2.212059000	-0.955235000	4.195631000
1	-2.072387000	-0.026920000	4.743088000
6	-2.363559000	-0.916030000	2.792045000
1	-2.331695000	0.041914000	2.280179000
7	-2.233062000	-2.057602000	4.902195000
6	5.888433000	-3.340200000	-1.971703000
9	5.527037000	-3.057549000	-3.236708000
9	7.223308000	-3.270301000	-1.921995000
9	5.549730000	-4.619893000	-1.759063000
8	0.313750000	0.579555000	0.993407000
6	0.256701000	1.628053000	1.685005000
6	1.365045000	2.367482000	2.115635000
1	2.349651000	1.917919000	2.021865000
6	1.211825000	3.494177000	3.005017000
1	0.192987000	3.850183000	3.161053000
1	3.252941000	3.814924000	3.478143000
6	2.222976000	4.138008000	3.607047000
1	2.050191000	4.993414000	4.250916000
1	-0.741857000	2.030475000	1.917754000
1	-3.199854000	-0.260200000	-1.420567000
6	-0.279405000	-0.090643000	-2.951322000
6	0.509578000	0.800582000	-2.204790000
6	1.904383000	0.643903000	-2.235759000
6	2.551145000	-0.333218000	-2.979208000
6	1.747811000	-1.215882000	-3.684722000
6	0.348052000	-1.094825000	-3.669844000
6	0.278141000	1.958172000	-1.347272000
6	1.566113000	2.365910000	-0.926326000
1	-1.354574000	0.019377000	-2.979031000
1	3.632375000	-0.403130000	-2.986852000
1	2.210862000	-2.008407000	-4.262529000

1	-0.249950000	-1.795257000	-4.242338000
8	2.544309000	1.549812000	-1.458239000
6	1.810204000	3.385232000	-0.030061000
1	0.941661000	4.010106000	0.139649000
6	3.094239000	3.983471000	0.318883000
6	3.086269000	5.304526000	0.790735000
6	4.322304000	3.307463000	0.229651000
6	4.269546000	5.950106000	1.130455000
6	5.499845000	3.952012000	0.582348000
6	5.479288000	5.274313000	1.026578000
1	2.140412000	5.828126000	0.876481000
1	4.351610000	2.278829000	-0.112464000
1	4.243065000	6.975100000	1.482967000
1	6.442223000	3.422927000	0.513203000
1	6.405868000	5.769914000	1.294505000
7	-0.779908000	2.687474000	-0.963473000
8	-2.734509000	1.527859000	-2.218076000
8	-2.575384000	1.332725000	0.218605000
16	-2.276525000	2.169020000	-0.964898000
6	-3.173061000	3.691384000	-0.784363000
6	-4.176685000	3.995215000	-1.690392000
6	-2.893193000	4.527067000	0.295580000
6	-4.912646000	5.165133000	-1.513668000
6	-3.634282000	5.685973000	0.454157000
6	-4.654316000	6.022282000	-0.446266000
1	-4.372199000	3.333752000	-2.525957000
1	-2.102380000	4.275422000	0.993924000
1	-5.696521000	5.412146000	-2.221886000
1	-3.421439000	6.346532000	1.288858000
6	-5.442372000	7.288128000	-0.250574000
1	-6.198235000	7.411814000	-1.026710000
1	-5.945672000	7.285570000	0.719604000
1	-4.784685000	8.160773000	-0.272192000

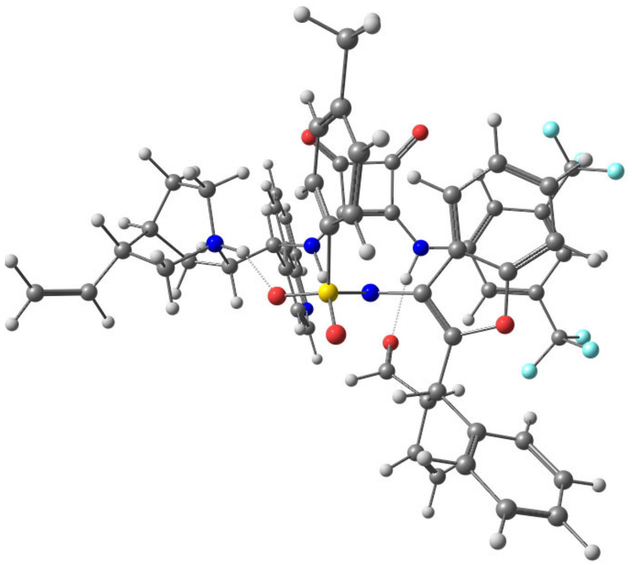

II

Sum of electronic and thermal Free Energies= -3922.527579

(-19.6 kcal/mol)

6	3.670051000	-0.560238000	-0.662859000
1	4.154373000	-1.270021000	0.006426000
6	1.703082000	-1.399695000	0.456553000
6	2.175941000	-2.371465000	1.438977000
6	0.758429000	-2.937228000	1.477063000
6	0.413585000	-1.927594000	0.429682000
6	-1.938102000	-2.034733000	-0.337961000
6	-2.429052000	-2.902775000	0.636117000
1	-1.774009000	-3.311536000	1.395242000
6	-3.781466000	-3.212733000	0.639120000
6	-4.660856000	-2.688650000	-0.298173000
1	-5.719155000	-2.922734000	-0.264956000
6	-4.146837000	-1.837849000	-1.269348000
6	-2.801899000	-1.512146000	-1.305211000
1	-2.407968000	-0.852956000	-2.069163000
6	-5.099404000	-1.230054000	-2.253763000
9	-5.791367000	-2.161186000	-2.927214000
9	-4.481027000	-0.465053000	-3.167148000
9	-6.008107000	-0.449889000	-1.645120000

7	2.321699000	-0.353349000	-0.136260000
1	1.711580000	0.321485000	-0.597188000
7	-0.612581000	-1.600337000	-0.369509000
1	-0.415453000	-0.870138000	-1.050826000
8	3.233216000	-2.612011000	1.977685000
8	0.190399000	-3.790631000	2.104773000
6	4.463602000	0.750287000	-0.650375000
1	3.960593000	1.471376000	-1.298400000
6	5.946782000	0.572522000	-1.045233000
1	6.192724000	1.223895000	-1.884639000
1	6.123493000	-0.453782000	-1.375059000
6	6.835922000	0.868454000	0.170766000
1	7.876961000	0.648882000	-0.068335000
6	6.359272000	-0.003751000	1.339561000
1	7.053980000	0.061701000	2.177685000
1	6.315496000	-1.050613000	1.028110000
6	4.969028000	0.482482000	1.784663000
1	4.270234000	-0.337055000	1.947175000
1	5.005797000	1.084958000	2.692199000
6	5.190553000	2.663655000	0.687063000
1	4.932644000	3.215879000	1.589410000
1	4.831372000	3.224453000	-0.176818000
6	6.699280000	2.348399000	0.594997000
1	7.147143000	2.454180000	1.587538000
6	7.389654000	3.299794000	-0.338940000
1	7.020908000	3.325455000	-1.363575000
6	8.398582000	4.087659000	0.013272000
1	8.785349000	4.095343000	1.028766000
1	8.873494000	4.753389000	-0.699520000
7	4.416983000	1.378748000	0.716807000
6	3.632723000	-1.129229000	-2.076844000
6	4.227808000	-2.380280000	-2.420790000
6	4.913956000	-3.226246000	-1.505571000
1	4.999845000	-2.954086000	-0.460549000
6	5.460446000	-4.411967000	-1.921220000

1	5.972957000	-5.046414000	-1.207199000
6	5.356118000	-4.821204000	-3.271029000
1	5.795192000	-5.762225000	-3.582205000
6	4.696910000	-4.035086000	-4.176019000
1	4.592812000	-4.323215000	-5.215904000
6	4.116039000	-2.803911000	-3.777239000
6	2.918904000	-0.947514000	-4.370048000
1	2.394780000	-0.388951000	-5.140791000
6	2.978179000	-0.429700000	-3.057234000
1	2.484048000	0.512557000	-2.844472000
7	3.465867000	-2.079937000	-4.731405000
6	-4.322108000	-4.136929000	1.690282000
9	-5.517066000	-3.727450000	2.146277000
9	-4.505189000	-5.381136000	1.215731000
9	-3.511011000	-4.243018000	2.749282000
8	-0.354059000	0.292133000	-2.641116000
6	-0.623412000	1.467074000	-2.539132000
6	-2.023797000	2.028170000	-2.636794000
1	-2.711848000	1.208185000	-2.851330000
6	-2.024746000	3.012831000	-3.778185000
1	-1.454007000	3.927442000	-3.623600000
1	-3.254858000	1.907720000	-5.086288000
6	-2.672474000	2.809978000	-4.918696000
1	-2.652956000	3.542387000	-5.718690000
1	0.177770000	2.216501000	-2.414387000
1	3.427362000	1.633946000	0.903002000
6	-1.648225000	-0.407788000	2.657382000
6	-2.087223000	0.388674000	1.593395000
6	-3.458868000	0.473581000	1.330096000
6	-4.425089000	-0.188104000	2.074871000
6	-3.969813000	-0.962107000	3.135308000
6	-2.598585000	-1.070958000	3.420750000
6	-1.450502000	1.208792000	0.577823000
6	-2.466293000	1.728120000	-0.163466000
1	-0.591289000	-0.501682000	2.881519000

1	-5.477759000	-0.104185000	1.829529000
1	-4.685908000	-1.499986000	3.746361000
1	-2.277774000	-1.695225000	4.247856000
8	-3.692334000	1.283490000	0.262004000
6	-2.399398000	2.716632000	-1.291054000
1	-1.578097000	3.395810000	-1.039076000
6	-3.667811000	3.539141000	-1.407687000
6	-3.678802000	4.863292000	-0.974794000
6	-4.835950000	2.992856000	-1.944415000
6	-4.835492000	5.633721000	-1.075682000
6	-5.990442000	3.758825000	-2.046359000
6	-5.993475000	5.083385000	-1.612595000
1	-2.776639000	5.293739000	-0.550890000
1	-4.845857000	1.962004000	-2.281989000
1	-4.828007000	6.663141000	-0.734494000
1	-6.890663000	3.321377000	-2.464221000
1	-6.894960000	5.680749000	-1.693427000
7	-0.079749000	1.377643000	0.310464000
8	2.029810000	2.596981000	0.616558000
8	-0.074304000	3.815448000	1.146287000
16	0.632527000	2.529864000	1.117975000
6	0.756358000	1.999596000	2.827131000
6	1.580818000	0.917261000	3.131533000
6	-0.051744000	2.564386000	3.801054000
6	1.609141000	0.415908000	4.422664000
6	-0.024441000	2.044172000	5.094611000
6	0.800924000	0.970738000	5.424583000
1	2.165159000	0.445414000	2.349346000
1	-0.697993000	3.397123000	3.549218000
1	2.248453000	-0.430685000	4.657067000
1	-0.662451000	2.480559000	5.856305000
6	0.833447000	0.407116000	6.819002000
1	1.819299000	0.545952000	7.270991000
1	0.095540000	0.889884000	7.460583000
1	0.629036000	-0.666303000	6.809968000

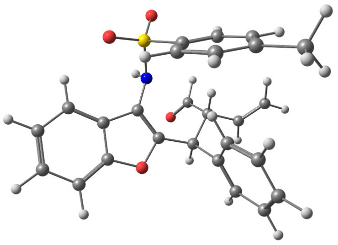

II-NC

Sum of electronic and thermal Free Energies= -1757.776401

(5.4 kcal/mol)

6	1.674281000	-0.215337000	0.514012000
6	2.854607000	-0.497853000	-0.283503000
6	4.055257000	0.156382000	-0.583354000
1	4.259032000	1.146041000	-0.196092000
6	4.965188000	-0.493449000	-1.403234000
1	5.901626000	-0.005562000	-1.648875000
6	4.700960000	-1.768889000	-1.928787000
1	5.434125000	-2.243168000	-2.570976000
6	3.518865000	-2.435063000	-1.638205000
1	3.297581000	-3.421814000	-2.026652000
6	2.627875000	-1.770448000	-0.809309000
6	0.870736000	-1.312687000	0.428338000
6	-0.431975000	-1.744242000	1.033042000
1	-0.222892000	-2.676048000	1.570895000
6	-1.454261000	-2.036530000	-0.053426000
6	-2.112551000	-3.262079000	-0.110300000
1	-1.863311000	-4.039009000	0.605873000
6	-3.085573000	-3.494081000	-1.080326000
1	-3.591370000	-4.452726000	-1.116456000
6	-3.405728000	-2.501978000	-2.001149000
1	-4.160997000	-2.684265000	-2.757567000
6	-2.748406000	-1.274031000	-1.949161000
1	-2.984980000	-0.495774000	-2.667911000
6	-1.780656000	-1.045435000	-0.978646000

1	-1.263001000	-0.092617000	-0.941100000
6	-0.998284000	-0.736304000	2.045128000
1	-1.171498000	0.228966000	1.565736000
6	-2.299784000	-1.218413000	2.638940000
1	-2.291915000	-2.229044000	3.044771000
6	-3.395775000	-0.473543000	2.714475000
1	-3.423400000	0.535732000	2.312371000
1	-4.305146000	-0.849328000	3.171202000
6	-0.064076000	-0.511296000	3.219104000
1	-0.262162000	0.414199000	3.793010000
6	-0.228082000	2.369677000	-0.247095000
6	-1.427369000	2.519617000	0.447297000
1	-1.425427000	2.670498000	1.521545000
6	-2.616572000	2.488550000	-0.263281000
1	-3.558450000	2.602677000	0.263725000
6	-2.623129000	2.321877000	-1.654547000
6	-1.405174000	2.191552000	-2.322323000
1	-1.394136000	2.065449000	-3.399697000
6	-0.199795000	2.213243000	-1.626773000
1	0.745712000	2.113672000	-2.146569000
6	-3.928898000	2.288749000	-2.397985000
1	-3.781773000	2.069951000	-3.456150000
1	-4.440561000	3.251540000	-2.317502000
1	-4.594228000	1.531513000	-1.975909000
7	1.415327000	0.910683000	1.343539000
8	1.430712000	-2.255211000	-0.378065000
8	0.787429000	-1.287328000	3.564370000
8	2.359827000	2.665280000	-0.319845000
8	1.172477000	3.331809000	1.785493000
16	1.298983000	2.438709000	0.646512000
1	2.032496000	0.980652000	2.154136000

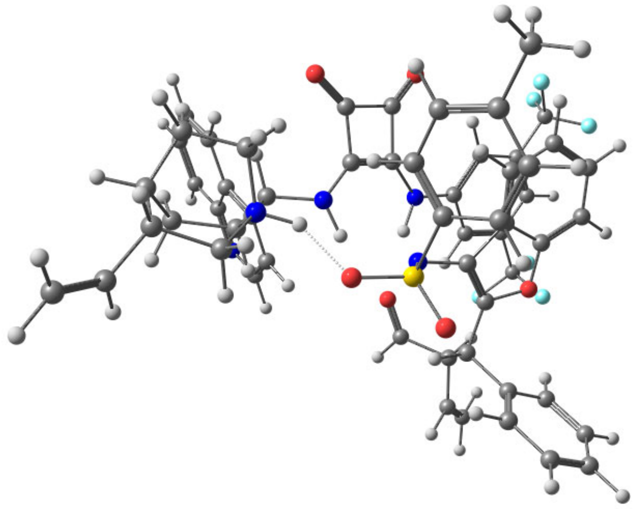

TS-Rot

Sum of electronic and thermal Free Energies= -3922.518795

(-14.1 kcal/mol)

6	3.655951000	-0.775597000	-0.466288000
1	4.142250000	-1.345996000	0.324547000
6	1.683256000	-1.317928000	0.806235000
6	2.144370000	-2.110278000	1.941601000
6	0.725816000	-2.656642000	2.065723000
6	0.383725000	-1.819684000	0.872325000
6	-1.972184000	-2.068096000	0.175583000
6	-2.464508000	-2.645792000	1.347480000
1	-1.815086000	-2.816342000	2.195681000
6	-3.806614000	-2.984154000	1.420301000
6	-4.684578000	-2.765761000	0.364732000
1	-5.734875000	-3.019020000	0.449388000
6	-4.173823000	-2.200762000	-0.794726000
6	-2.835467000	-1.857544000	-0.900907000
1	-2.453798000	-1.425814000	-1.815253000
6	-5.099690000	-1.868692000	-1.925661000
9	-6.100286000	-2.750836000	-2.040202000
9	-4.464289000	-1.831721000	-3.109097000
9	-5.670270000	-0.661224000	-1.765440000
7	2.331317000	-0.414125000	0.035987000

1	1.729027000	0.173242000	-0.542632000
7	-0.652670000	-1.628439000	0.040860000
1	-0.483929000	-0.997161000	-0.743220000
8	3.198279000	-2.260460000	2.517655000
8	0.157517000	-3.407672000	2.812769000
6	4.477446000	0.486397000	-0.750145000
1	3.976487000	1.057658000	-1.535138000
6	5.952368000	0.198914000	-1.107912000
1	6.203719000	0.659035000	-2.064166000
1	6.107817000	-0.875984000	-1.221075000
6	6.858543000	0.722765000	0.015166000
1	7.892090000	0.431582000	-0.174875000
6	6.371785000	0.131738000	1.345428000
1	7.075421000	0.357693000	2.147409000
1	6.301334000	-0.956200000	1.264903000
6	4.997336000	0.737159000	1.681361000
1	4.276784000	-0.007190000	2.018845000
1	5.058840000	1.519833000	2.437700000
6	5.259663000	2.626068000	0.139820000
1	5.022971000	3.364997000	0.903633000
1	4.903455000	2.995966000	-0.822158000
6	6.759878000	2.262298000	0.111920000
1	7.219833000	2.568075000	1.056148000
6	7.461047000	2.974582000	-1.008436000
1	7.079019000	2.791168000	-2.012018000
6	8.491510000	3.794595000	-0.839653000
1	8.890983000	4.008263000	0.148206000
1	8.972453000	4.281557000	-1.681291000
7	4.458433000	1.397008000	0.448182000
6	3.557616000	-1.624944000	-1.729508000
6	4.156766000	-2.915213000	-1.843427000
6	4.913298000	-3.545746000	-0.816869000
1	5.061123000	-3.055436000	0.137521000
6	5.456748000	-4.788613000	-1.008560000
1	6.024921000	-5.255203000	-0.212032000

6	5.278855000	-5.472922000	-2.233616000
1	5.715914000	-6.455858000	-2.367711000
6	4.553300000	-4.897605000	-3.240542000
1	4.394033000	-5.397715000	-4.189089000
6	3.974881000	-3.613085000	-3.073034000
6	2.715725000	-1.925960000	-3.965978000
1	2.140836000	-1.541350000	-4.804227000
6	2.837961000	-1.147200000	-2.794148000
1	2.339852000	-0.184204000	-2.751944000
7	3.259297000	-3.106892000	-4.116459000
6	-4.346810000	-3.594422000	2.680014000
9	-5.539499000	-3.073015000	3.012176000
9	-4.537037000	-4.918833000	2.553740000
9	-3.534589000	-3.417191000	3.728305000
8	-0.668548000	-0.289649000	-2.673027000
6	-1.340926000	0.238345000	-3.524523000
6	-2.553386000	1.121545000	-3.260483000
1	-3.363739000	0.447132000	-2.952195000
6	-2.950438000	1.788728000	-4.551076000
1	-2.336093000	2.630262000	-4.867780000
1	-4.586122000	0.541244000	-5.007368000
6	-3.956110000	1.374065000	-5.310566000
1	-4.193044000	1.855728000	-6.253208000
1	-1.089497000	0.082814000	-4.589915000
1	3.476323000	1.707881000	0.577715000
6	-1.715972000	0.353354000	2.640719000
6	-2.083479000	0.795155000	1.364932000
6	-3.434572000	0.765483000	1.002035000
6	-4.448359000	0.341738000	1.848800000
6	-4.064877000	-0.070989000	3.119471000
6	-2.715257000	-0.070155000	3.506946000
6	-1.375377000	1.274008000	0.187143000
6	-2.337694000	1.499675000	-0.747163000
1	-0.675778000	0.342508000	2.946863000
1	-5.482633000	0.329594000	1.524080000

1	-4.820502000	-0.410935000	3.818765000
1	-2.449730000	-0.414275000	4.500834000
8	-3.592923000	1.191056000	-0.279293000
6	-2.254419000	2.122846000	-2.102161000
1	-1.219679000	2.461960000	-2.201686000
6	-3.153714000	3.351136000	-2.135730000
6	-2.597241000	4.614014000	-1.938216000
6	-4.534542000	3.238802000	-2.315843000
6	-3.403531000	5.750137000	-1.938602000
6	-5.339939000	4.371637000	-2.314760000
6	-4.775794000	5.632253000	-2.130341000
1	-1.530647000	4.699989000	-1.756568000
1	-4.983207000	2.261032000	-2.457654000
1	-2.956836000	6.726564000	-1.784939000
1	-6.410264000	4.270644000	-2.458561000
1	-5.404874000	6.515754000	-2.132332000
7	0.010818000	1.365494000	-0.015913000
8	2.119007000	2.625806000	0.037648000
8	0.007478000	3.938395000	0.110442000
16	0.699075000	2.696013000	0.471104000
6	0.749989000	2.660365000	2.263954000
6	1.543635000	1.700144000	2.891973000
6	-0.082801000	3.481702000	3.006757000
6	1.512379000	1.582054000	4.271950000
6	-0.113513000	3.346255000	4.394428000
6	0.677961000	2.401913000	5.044825000
1	2.151933000	1.021782000	2.301324000
1	-0.704497000	4.213621000	2.504357000
1	2.127285000	0.831876000	4.761189000
1	-0.770453000	3.984397000	4.976621000
6	0.643185000	2.246504000	6.540862000
1	1.631018000	2.422961000	6.974444000
1	-0.058503000	2.945564000	6.997100000
1	0.343480000	1.232787000	6.818941000

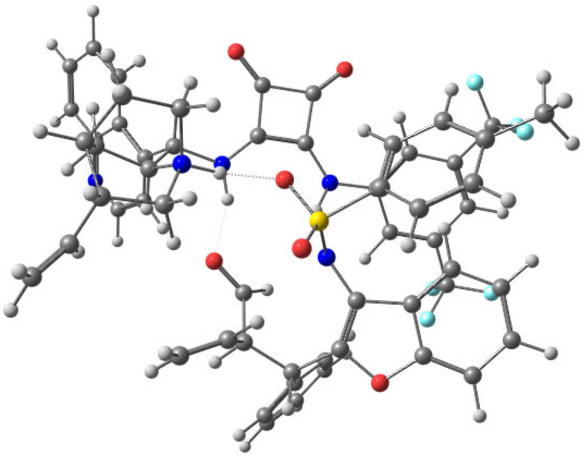

Rot-II

Sum of electronic and thermal Free Energies= -3922,527293

(-19.4 kcal/mol)

6	3.967628000	-0.923328000	-0.376823000
1	4.171341000	-1.959383000	-0.115836000
6	1.797851000	-1.933031000	-0.857561000
6	2.090605000	-3.373816000	-0.842591000
6	0.622179000	-3.616911000	-1.149457000
6	0.437200000	-2.150282000	-1.082759000
6	-1.921857000	-1.494637000	-1.424660000
6	-2.534826000	-2.724141000	-1.168581000
1	-1.950248000	-3.569948000	-0.829971000
6	-3.903705000	-2.847090000	-1.347781000
6	-4.687899000	-1.788769000	-1.795097000
1	-5.758540000	-1.903053000	-1.915584000
6	-4.061088000	-0.581304000	-2.059849000
6	-2.695028000	-0.422634000	-1.872568000
1	-2.219884000	0.533805000	-2.067110000
6	-4.857514000	0.582843000	-2.571701000
9	-6.168013000	0.445321000	-2.341822000
9	-4.709596000	0.738227000	-3.900601000
9	-4.474342000	1.741050000	-2.015173000
7	2.548660000	-0.845053000	-0.701467000
1	2.114640000	0.077347000	-0.687729000

7	-0.563642000	-1.249936000	-1.184478000
1	-0.395338000	-0.318340000	-0.790640000
8	3.070089000	-4.051975000	-0.633716000
8	-0.065948000	-4.591584000	-1.338436000
6	4.272123000	-0.015943000	0.824738000
1	4.067081000	1.014627000	0.530759000
6	5.702617000	-0.151083000	1.371011000
1	6.334142000	0.623070000	0.933634000
1	6.130725000	-1.117439000	1.086939000
6	5.651613000	-0.051221000	2.903778000
1	6.659198000	0.050976000	3.307792000
6	4.984536000	-1.325641000	3.440434000
1	4.811194000	-1.233730000	4.514515000
1	5.633115000	-2.188100000	3.281034000
6	3.651368000	-1.535546000	2.698581000
1	3.692641000	-2.347457000	1.973136000
1	2.815941000	-1.728291000	3.369571000
6	3.336375000	0.888334000	2.893965000
1	2.680394000	0.631648000	3.725220000
1	2.897499000	1.727821000	2.354695000
6	4.793081000	1.157031000	3.347112000
1	4.812392000	1.193045000	4.439104000
6	5.301482000	2.472668000	2.824401000
1	5.266241000	2.620078000	1.745072000
6	5.763324000	3.449084000	3.597212000
1	5.802608000	3.345147000	4.678413000
1	6.118285000	4.385062000	3.178851000
7	3.317157000	-0.279157000	1.955468000
6	4.853155000	-0.498096000	-1.539364000
6	5.944896000	-1.292182000	-1.998769000
6	6.275913000	-2.577165000	-1.485855000
1	5.661314000	-3.038203000	-0.722767000
6	7.351739000	-3.269198000	-1.975830000
1	7.584349000	-4.251143000	-1.579916000
6	8.156310000	-2.721491000	-3.002695000

1	9.005036000	-3.283012000	-3.376334000
6	7.855441000	-1.495529000	-3.529693000
1	8.444352000	-1.053995000	-4.325587000
6	6.744782000	-0.755182000	-3.048989000
6	5.468021000	1.136975000	-3.195584000
1	5.279851000	2.093055000	-3.676556000
6	4.620703000	0.707047000	-2.150564000
1	3.795145000	1.342743000	-1.845995000
7	6.495768000	0.452231000	-3.629553000
6	-4.596068000	-4.113544000	-0.941928000
9	-5.297308000	-3.937328000	0.195400000
9	-5.475287000	-4.529512000	-1.864568000
9	-3.745591000	-5.121280000	-0.718360000
8	1.945987000	1.865952000	0.007631000
6	0.927572000	2.480902000	-0.235976000
6	0.529738000	3.732312000	0.518104000
1	1.138756000	4.507406000	0.029842000
6	0.926972000	3.684561000	1.966638000
1	0.337328000	3.031638000	2.604456000
1	2.569388000	5.011054000	1.840250000
6	1.953416000	4.366475000	2.462911000
1	2.217090000	4.304144000	3.514228000
1	0.319434000	2.220499000	-1.115928000
1	2.340297000	-0.363522000	1.608006000
6	-4.222095000	0.321973000	0.994797000
6	-3.463066000	1.498738000	0.974292000
6	-4.128177000	2.725555000	0.920664000
6	-5.510038000	2.856954000	0.947220000
6	-6.242389000	1.678999000	0.991175000
6	-5.604616000	0.426925000	1.002691000
6	-2.042626000	1.821627000	0.871731000
6	-1.985308000	3.176683000	0.732639000
1	-3.747489000	-0.653721000	0.976975000
1	-5.982661000	3.831078000	0.906281000
1	-7.325574000	1.725783000	0.997440000

1	-6.204896000	-0.477164000	1.006218000
8	-3.240393000	3.740681000	0.782887000
6	-0.942010000	4.155867000	0.289964000
1	-1.084332000	5.064234000	0.885610000
6	-1.170115000	4.549064000	-1.173336000
6	-0.621159000	5.740938000	-1.651913000
6	-1.900571000	3.749789000	-2.051622000
6	-0.789875000	6.118951000	-2.979356000
6	-2.073796000	4.125631000	-3.381269000
6	-1.515996000	5.309875000	-3.850510000
1	-0.063241000	6.382529000	-0.974995000
1	-2.354709000	2.832681000	-1.692399000
1	-0.360847000	7.050293000	-3.332624000
1	-2.654724000	3.492762000	-4.044112000
1	-1.652616000	5.606507000	-4.884425000
7	-0.965371000	0.931429000	0.746452000
8	0.680418000	-0.664154000	1.570700000
8	-0.206388000	1.001441000	3.212369000
16	-0.464097000	0.157955000	2.037137000
6	-1.683433000	-1.033022000	2.576476000
6	-1.745211000	-2.267936000	1.943561000
6	-2.617754000	-0.679463000	3.544901000
6	-2.779756000	-3.143253000	2.254344000
6	-3.641661000	-1.566019000	3.850454000
6	-3.750787000	-2.798563000	3.197528000
1	-0.978385000	-2.543877000	1.228674000
1	-2.547900000	0.281249000	4.042020000
1	-2.832276000	-4.107685000	1.758425000
1	-4.379670000	-1.291736000	4.597962000
6	-4.898661000	-3.721638000	3.503119000
1	-5.835970000	-3.304469000	3.124217000
1	-4.757138000	-4.700450000	3.043648000
1	-5.016024000	-3.859493000	4.580435000

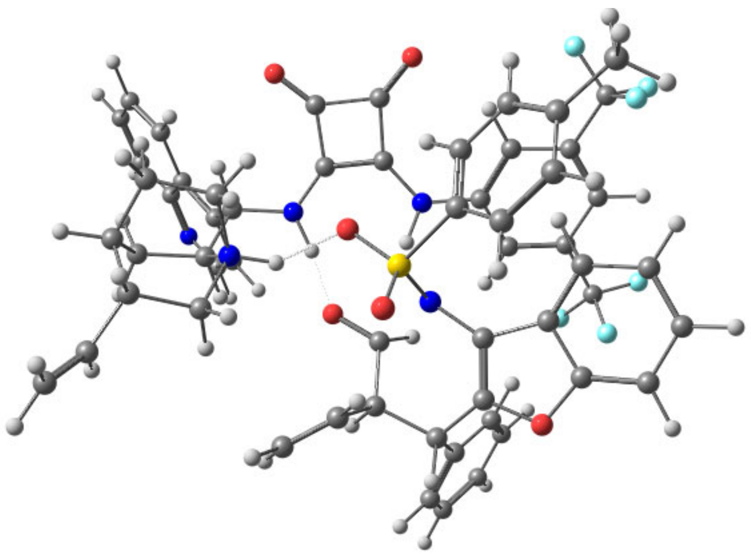

TS2

Sum of electronic and thermal Free Energies= -3922.515165

(-11.8 kcal/mol)

6	4.103940000	-0.605258000	-0.446119000
1	4.403730000	-1.526096000	0.051076000
6	2.017442000	-1.855514000	-0.682728000
6	2.358774000	-3.265788000	-0.429185000
6	0.877272000	-3.587846000	-0.589322000
6	0.664137000	-2.147364000	-0.833946000
6	-1.677531000	-1.515889000	-1.344832000
6	-2.289586000	-2.736838000	-1.053356000
1	-1.714946000	-3.552959000	-0.629964000
6	-3.642044000	-2.894351000	-1.316704000
6	-4.401475000	-1.888187000	-1.902107000
1	-5.455027000	-2.032578000	-2.108819000
6	-3.768486000	-0.692276000	-2.210381000
6	-2.429175000	-0.485881000	-1.915828000
1	-1.949113000	0.457908000	-2.158825000
6	-4.550155000	0.426481000	-2.831318000
9	-5.632686000	-0.011919000	-3.484283000
9	-3.808477000	1.119660000	-3.713030000
9	-4.979656000	1.313128000	-1.920161000

7	2.682409000	-0.709047000	-0.737257000
1	2.138280000	0.163617000	-0.801905000
7	-0.333233000	-1.267530000	-1.074263000
1	-0.092283000	-0.286480000	-0.962104000
8	3.366884000	-3.876098000	-0.166019000
8	0.200209000	-4.585284000	-0.500226000
6	4.320718000	0.587375000	0.498011000
1	4.016179000	1.495294000	-0.026346000
6	5.759920000	0.701840000	1.044730000
1	6.169082000	1.687719000	0.821072000
1	6.403775000	-0.031064000	0.553306000
6	5.750767000	0.429786000	2.555430000
1	6.772651000	0.402135000	2.935078000
6	5.050299000	-0.915177000	2.792477000
1	5.162920000	-1.231707000	3.830139000
1	5.501856000	-1.685827000	2.161811000
6	3.556548000	-0.752522000	2.468900000
1	3.134876000	-1.582592000	1.905883000
1	2.945430000	-0.623823000	3.360253000
6	3.602477000	1.690267000	2.552111000
1	2.751627000	1.756622000	3.229634000
1	3.594215000	2.567108000	1.904483000
6	4.945097000	1.521983000	3.295722000
1	4.749978000	1.165093000	4.311296000
6	5.662439000	2.837728000	3.385957000
1	5.925502000	3.310576000	2.440316000
6	5.972509000	3.441134000	4.527415000
1	5.715835000	3.003495000	5.488461000
1	6.494525000	4.391987000	4.542681000
7	3.375714000	0.503124000	1.667174000
6	4.928447000	-0.407851000	-1.711857000
6	6.026105000	-1.253722000	-2.051558000
6	6.450368000	-2.373127000	-1.281976000
1	5.913691000	-2.660568000	-0.386286000
6	7.521854000	-3.128907000	-1.678933000

1	7.825117000	-3.983551000	-1.085072000
6	8.231058000	-2.813350000	-2.861663000
1	9.076895000	-3.422858000	-3.158832000
6	7.843682000	-1.750049000	-3.629802000
1	8.360378000	-1.486764000	-4.545753000
6	6.736105000	-0.949042000	-3.249652000
6	5.378897000	0.828242000	-3.728349000
1	5.124455000	1.646924000	-4.395929000
6	4.612437000	0.621732000	-2.560494000
1	3.777541000	1.284079000	-2.355032000
7	6.403542000	0.088186000	-4.068069000
6	-4.336212000	-4.140310000	-0.854577000
9	-4.978530000	-3.928757000	0.312542000
9	-5.264028000	-4.560605000	-1.724651000
9	-3.491741000	-5.156553000	-0.646670000
8	1.314922000	1.574889000	-0.169652000
6	0.154354000	2.072860000	-0.170461000
6	-0.016055000	3.498026000	0.387762000
1	0.594442000	4.094123000	-0.300929000
6	0.534305000	3.692456000	1.768917000
1	-0.005960000	3.210853000	2.578973000
1	2.162590000	4.932848000	1.243663000
6	1.603185000	4.436444000	2.033333000
1	1.960222000	4.577451000	3.049177000
1	-0.477066000	1.919609000	-1.063716000
1	2.398561000	0.544138000	1.327896000
6	-4.234473000	-0.152251000	1.002363000
6	-3.648596000	1.119249000	0.975364000
6	-4.488481000	2.236909000	0.906496000
6	-5.873302000	2.169047000	0.951764000
6	-6.429374000	0.899749000	1.019099000
6	-5.617695000	-0.245980000	1.024344000
6	-2.296450000	1.663904000	0.896044000
6	-2.448305000	3.002100000	0.716197000
1	-3.628712000	-1.051122000	0.983855000

1	-6.479229000	3.066214000	0.908182000
1	-7.507737000	0.791216000	1.045942000
1	-6.080045000	-1.227422000	1.040005000
8	-3.763912000	3.374701000	0.740648000
6	-1.470406000	4.033160000	0.275876000
1	-1.552434000	4.907972000	0.929889000
6	-1.770429000	4.508397000	-1.144502000
6	-1.276867000	5.750639000	-1.550185000
6	-2.483857000	3.738153000	-2.063019000
6	-1.482606000	6.210416000	-2.845684000
6	-2.693584000	4.197031000	-3.361649000
6	-2.192083000	5.432190000	-3.757424000
1	-0.727501000	6.361762000	-0.839043000
1	-2.896540000	2.779861000	-1.762229000
1	-1.095066000	7.179023000	-3.142336000
1	-3.256704000	3.588165000	-4.059830000
1	-2.358539000	5.790259000	-4.767291000
7	-1.048210000	1.010301000	0.826250000
8	0.775261000	-0.344705000	1.811229000
8	-0.444710000	1.203021000	3.349742000
16	-0.486311000	0.308263000	2.190651000
6	-1.608667000	-0.998274000	2.643510000
6	-1.473811000	-2.240366000	2.038870000
6	-2.607205000	-0.758984000	3.582725000
6	-2.386392000	-3.244642000	2.339368000
6	-3.504579000	-1.774407000	3.879762000
6	-3.421709000	-3.021276000	3.249600000
1	-0.652965000	-2.421561000	1.355969000
1	-2.684880000	0.210896000	4.059918000
1	-2.289466000	-4.214773000	1.861637000
1	-4.295625000	-1.593339000	4.600782000
6	-4.433906000	-4.091294000	3.552099000
1	-5.438300000	-3.757128000	3.278938000
1	-4.220223000	-5.008983000	3.003375000
1	-4.447573000	-4.323412000	4.619991000

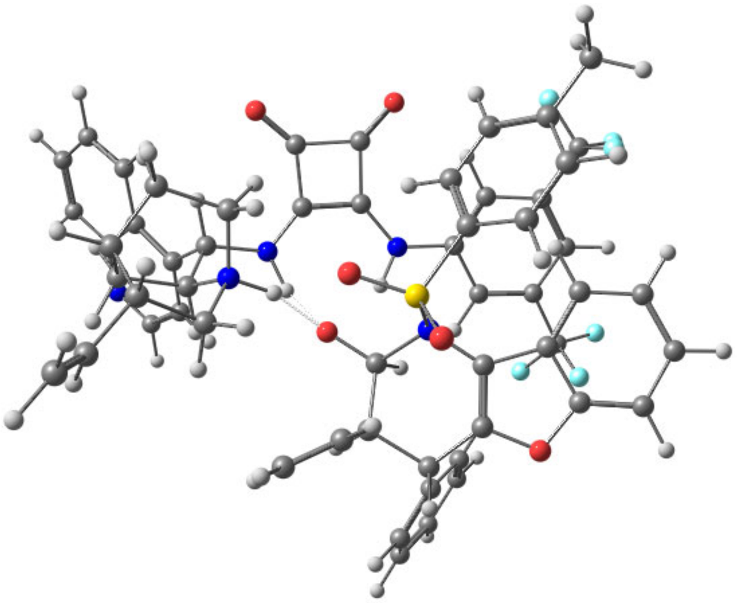

III

Sum of electronic and thermal Free Energies= -3922.516720

(-12.8 kcal/mol)

6	4.137452000	-0.457977000	-0.590553000
1	4.410747000	-1.440404000	-0.211778000
6	2.063206000	-1.665837000	-0.995806000
6	2.380978000	-3.082229000	-0.749493000
6	0.900108000	-3.385208000	-0.934191000
6	0.703882000	-1.936934000	-1.156699000
6	-1.635200000	-1.268886000	-1.612364000
6	-2.240187000	-2.498761000	-1.347292000
1	-1.653126000	-3.333683000	-0.984839000
6	-3.602141000	-2.644533000	-1.562797000
6	-4.382246000	-1.615195000	-2.075126000
1	-5.444538000	-1.747957000	-2.242532000
6	-3.757687000	-0.408438000	-2.359276000
6	-2.406099000	-0.216938000	-2.114222000
1	-1.933378000	0.732702000	-2.344135000
6	-4.567986000	0.736291000	-2.889661000
9	-5.625716000	0.323633000	-3.597630000
9	-3.841968000	1.536119000	-3.688963000
9	-5.041718000	1.515212000	-1.904129000

7	2.751648000	-0.530253000	-1.029526000
1	2.189824000	0.330535000	-0.975182000
7	-0.278755000	-1.040684000	-1.389493000
1	0.007124000	-0.072691000	-1.254484000
8	3.376107000	-3.703525000	-0.460784000
8	0.211050000	-4.376209000	-0.861502000
6	4.262267000	0.580687000	0.537751000
1	4.004638000	1.560887000	0.131954000
6	5.660750000	0.591124000	1.194532000
1	6.071586000	1.601760000	1.186858000
1	6.345706000	-0.035055000	0.618563000
6	5.553565000	0.043727000	2.624027000
1	6.548614000	-0.083509000	3.051916000
6	4.811850000	-1.298521000	2.570769000
1	4.853720000	-1.798833000	3.539149000
1	5.285019000	-1.960119000	1.840481000
6	3.341950000	-1.031479000	2.204063000
1	2.942620000	-1.748980000	1.489439000
1	2.688885000	-1.035757000	3.072552000
6	3.449137000	1.360085000	2.695999000
1	2.550382000	1.368463000	3.308349000
1	3.536165000	2.326202000	2.198079000
6	4.718129000	1.004795000	3.497211000
1	4.429554000	0.471364000	4.407932000
6	5.455486000	2.250044000	3.895441000
1	5.824899000	2.875103000	3.083314000
6	5.666562000	2.621103000	5.152884000
1	5.304097000	2.027188000	5.987724000
1	6.208386000	3.529740000	5.393349000
7	3.232466000	0.343567000	1.613077000
6	5.064128000	-0.074964000	-1.736698000
6	6.161835000	-0.894311000	-2.136144000
6	6.479392000	-2.153364000	-1.554403000
1	5.847575000	-2.578752000	-0.783969000
6	7.562903000	-2.870689000	-1.988883000

1	7.784603000	-3.832301000	-1.540278000
6	8.389371000	-2.375614000	-3.024707000
1	9.243897000	-2.956202000	-3.353244000
6	8.102606000	-1.175879000	-3.616234000
1	8.708797000	-0.774612000	-4.420459000
6	6.984828000	-0.410955000	-3.194500000
6	5.717174000	1.473569000	-3.460802000
1	5.540924000	2.404921000	-3.992162000
6	4.847088000	1.097081000	-2.413607000
1	4.013973000	1.746204000	-2.163719000
7	6.751852000	0.767074000	-3.838707000
6	-4.279240000	-3.905319000	-1.116660000
9	-4.892589000	-3.726186000	0.071169000
9	-5.226921000	-4.308813000	-1.973162000
9	-3.424634000	-4.922201000	-0.955348000
8	1.075973000	1.200526000	0.087367000
6	-0.108610000	1.768380000	0.214029000
6	-0.112270000	3.235146000	0.735361000
1	0.559407000	3.757204000	0.048078000
6	0.417347000	3.414665000	2.125640000
1	-0.182793000	3.013423000	2.939460000
1	2.142092000	4.523371000	1.621451000
6	1.530553000	4.084405000	2.406472000
1	1.872878000	4.218854000	3.428171000
1	-0.681535000	1.803069000	-0.735778000
1	2.272216000	0.525497000	1.220634000
6	-4.324058000	-0.319889000	0.993426000
6	-3.758550000	0.959971000	1.024746000
6	-4.605654000	2.074346000	0.959130000
6	-5.988992000	1.988579000	0.950263000
6	-6.529810000	0.710159000	0.962068000
6	-5.706758000	-0.427403000	0.963881000
6	-2.423152000	1.524587000	1.014434000
6	-2.569584000	2.864424000	0.883451000
1	-3.702537000	-1.208039000	0.965850000

1	-6.605875000	2.878246000	0.908595000
1	-7.606936000	0.588682000	0.947236000
1	-6.160418000	-1.412001000	0.934042000
8	-3.884363000	3.225808000	0.855149000
6	-1.528216000	3.886679000	0.592029000
1	-1.607859000	4.702022000	1.318603000
6	-1.712747000	4.495966000	-0.793117000
6	-1.153760000	5.750245000	-1.050319000
6	-2.376909000	3.832728000	-1.825234000
6	-1.249677000	6.327386000	-2.311004000
6	-2.476756000	4.410067000	-3.089319000
6	-1.912506000	5.656817000	-3.336423000
1	-0.639447000	6.275866000	-0.250355000
1	-2.834555000	2.865014000	-1.641783000
1	-0.813005000	7.303611000	-2.491864000
1	-3.004424000	3.885717000	-3.877753000
1	-1.994525000	6.106984000	-4.319445000
7	-1.149334000	0.901464000	1.044018000
8	0.668046000	0.006231000	2.579727000
8	-1.301056000	1.117514000	3.637399000
16	-0.761162000	0.293556000	2.562463000
6	-1.624265000	-1.257575000	2.628878000
6	-1.200367000	-2.294370000	1.806481000
6	-2.696850000	-1.413636000	3.497213000
6	-1.883069000	-3.502181000	1.836175000
6	-3.370114000	-2.629372000	3.514965000
6	-2.982882000	-3.682449000	2.682140000
1	-0.341719000	-2.153563000	1.158920000
1	-3.010456000	-0.590847000	4.128325000
1	-1.556779000	-4.315172000	1.194851000
1	-4.219805000	-2.756970000	4.177907000
6	-3.718848000	-4.992887000	2.711701000
1	-3.561250000	-5.556469000	1.791663000
1	-3.367157000	-5.607292000	3.545877000
1	-4.791028000	-4.838696000	2.845439000

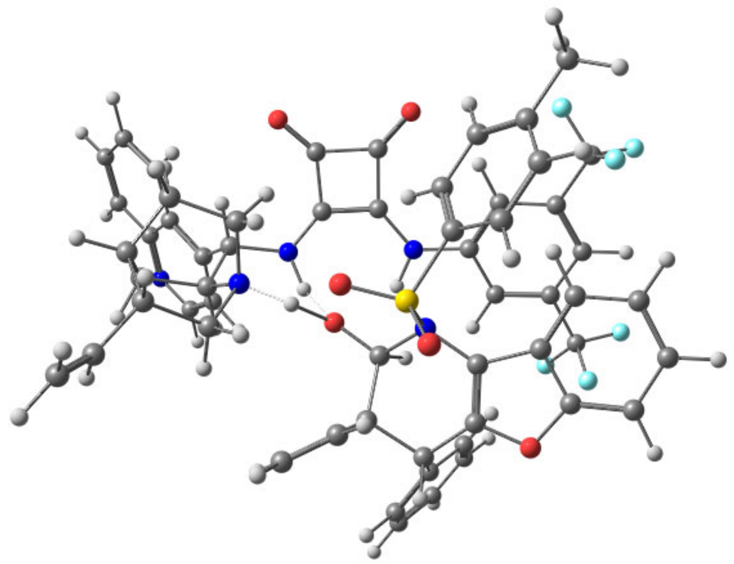

TS3

Sum of electronic and thermal Free Energies= -3922.513154

(-10.5 kcal/mol)

6	4.109207000	-0.420309000	-0.657170000
1	4.324081000	-1.430530000	-0.317425000
6	2.016894000	-1.537869000	-1.203948000
6	2.291173000	-2.967478000	-0.975666000
6	0.810628000	-3.227660000	-1.202783000
6	0.656343000	-1.771858000	-1.411997000
6	-1.681653000	-1.060207000	-1.828457000
6	-2.302188000	-2.271852000	-1.519323000
1	-1.716765000	-3.123918000	-1.198529000
6	-3.676693000	-2.384771000	-1.659656000
6	-4.460680000	-1.334449000	-2.121514000
1	-5.533470000	-1.440597000	-2.228783000
6	-3.822940000	-0.144094000	-2.443896000
6	-2.452835000	0.009733000	-2.289739000
1	-1.969566000	0.941813000	-2.564707000
6	-4.630983000	1.021745000	-2.930902000
9	-5.745935000	0.637106000	-3.562157000
9	-3.935201000	1.791022000	-3.784601000
9	-5.013152000	1.823442000	-1.923749000

7	2.750288000	-0.429938000	-1.189023000
1	2.221526000	0.437426000	-1.088760000
7	-0.309403000	-0.866948000	-1.689736000
1	0.001075000	0.097427000	-1.736402000
8	3.261296000	-3.623976000	-0.684959000
8	0.099569000	-4.205007000	-1.162920000
6	4.201364000	0.558888000	0.531417000
1	4.001237000	1.563009000	0.146658000
6	5.590338000	0.502674000	1.217268000
1	6.021027000	1.503514000	1.282886000
1	6.283357000	-0.100337000	0.626439000
6	5.435170000	-0.127068000	2.607642000
1	6.415881000	-0.312289000	3.049103000
6	4.650218000	-1.436049000	2.452525000
1	4.652089000	-1.998364000	3.388128000
1	5.124278000	-2.066401000	1.694274000
6	3.198811000	-1.086417000	2.063359000
1	2.796139000	-1.763183000	1.309668000
1	2.531162000	-1.131209000	2.919354000
6	3.372155000	1.242063000	2.689495000
1	2.467810000	1.262019000	3.293240000
1	3.512729000	2.237522000	2.261980000
6	4.606078000	0.807052000	3.512682000
1	4.272771000	0.231373000	4.382225000
6	5.366374000	2.001926000	4.006083000
1	5.794710000	2.652093000	3.243470000
6	5.529327000	2.310634000	5.287965000
1	5.110113000	1.692447000	6.077537000
1	6.086109000	3.189971000	5.594757000
7	3.130029000	0.311292000	1.551163000
6	5.106093000	-0.020322000	-1.736738000
6	6.228726000	-0.830924000	-2.082270000
6	6.524544000	-2.090461000	-1.489389000
1	5.864269000	-2.516961000	-0.743937000
6	7.632032000	-2.800879000	-1.871320000

1	7.837562000	-3.761483000	-1.412941000
6	8.506703000	-2.298276000	-2.863011000
1	9.379278000	-2.873735000	-3.150681000
6	8.246256000	-1.095608000	-3.460372000
1	8.892318000	-0.686096000	-4.228607000
6	7.106146000	-0.336404000	-3.090717000
6	5.855228000	1.551902000	-3.400898000
1	5.709029000	2.491026000	-3.927877000
6	4.928789000	1.161131000	-2.409301000
1	4.082756000	1.806957000	-2.197640000
7	6.907632000	0.848615000	-3.733593000
6	-4.349345000	-3.648482000	-1.211729000
9	-4.869640000	-3.514439000	0.024652000
9	-5.365152000	-3.995271000	-2.013135000
9	-3.507343000	-4.687528000	-1.159808000
8	1.088641000	1.022483000	0.307925000
6	-0.102863000	1.633492000	0.470349000
6	-0.071048000	3.084307000	1.024979000
1	0.682426000	3.596008000	0.420335000
6	0.309460000	3.226777000	2.468780000
1	-0.369713000	2.803371000	3.205476000
1	2.061472000	4.368910000	2.177223000
6	1.375205000	3.904225000	2.881128000
1	1.605162000	4.016179000	3.936046000
1	-0.587976000	1.721131000	-0.515391000
1	1.982789000	0.660600000	1.072577000
6	-4.369651000	-0.355078000	1.063686000
6	-3.772457000	0.910957000	1.082089000
6	-4.588870000	2.045707000	0.979439000
6	-5.972644000	1.994554000	0.932821000
6	-6.546240000	0.730656000	0.951922000
6	-5.753670000	-0.426988000	1.000228000
6	-2.426375000	1.444605000	1.106403000
6	-2.534063000	2.786949000	0.966992000
1	-3.772850000	-1.259325000	1.071386000

1	-6.564622000	2.898853000	0.858848000
1	-7.625332000	0.636328000	0.910384000
1	-6.232682000	-1.399440000	0.979309000
8	-3.836936000	3.179263000	0.887776000
6	-1.448017000	3.782413000	0.755549000
1	-1.562689000	4.596655000	1.478429000
6	-1.489984000	4.403181000	-0.635974000
6	-0.773144000	5.583252000	-0.854126000
6	-2.181290000	3.831014000	-1.703403000
6	-0.739083000	6.173604000	-2.111148000
6	-2.150066000	4.422562000	-2.965681000
6	-1.427698000	5.591694000	-3.173689000
1	-0.240335000	6.041257000	-0.025121000
1	-2.766017000	2.929020000	-1.550940000
1	-0.181562000	7.091699000	-2.261195000
1	-2.701372000	3.971353000	-3.782649000
1	-1.408045000	6.052898000	-4.154774000
7	-1.169264000	0.790225000	1.188569000
8	0.566674000	-0.045156000	2.833872000
8	-1.566712000	0.864359000	3.763203000
16	-0.872635000	0.128325000	2.716220000
6	-1.641501000	-1.465707000	2.609064000
6	-1.200780000	-2.363935000	1.644712000
6	-2.668706000	-1.785147000	3.487913000
6	-1.817627000	-3.603905000	1.550386000
6	-3.278409000	-3.028373000	3.375613000
6	-2.870009000	-3.947647000	2.405884000
1	-0.392954000	-2.084890000	0.975468000
1	-3.000491000	-1.062882000	4.223788000
1	-1.480514000	-4.311576000	0.799789000
1	-4.094504000	-3.283256000	4.043942000
6	-3.542452000	-5.287138000	2.294808000
1	-3.348984000	-5.749565000	1.326702000
1	-3.171727000	-5.963755000	3.070431000
1	-4.622047000	-5.195786000	2.427131000

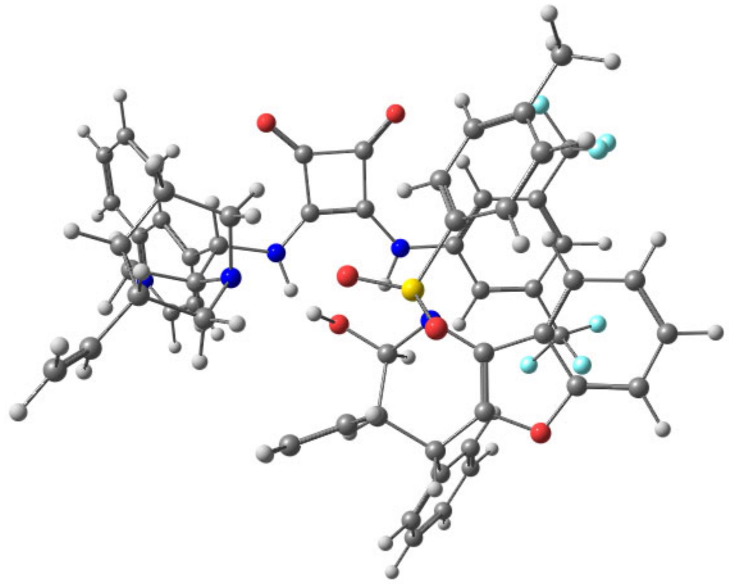

IV

Sum of electronic and thermal Free Energies= -3922.522090

(-16.2 kcal/mol)

6	4.258262000	-0.340191000	-0.562861000
1	4.477796000	-1.310563000	-0.123245000
6	2.162931000	-1.516013000	-0.956037000
6	2.485896000	-2.907054000	-0.578543000
6	1.023744000	-3.246562000	-0.790791000
6	0.817776000	-1.835233000	-1.171834000
6	-1.542994000	-1.297450000	-1.713391000
6	-2.101906000	-2.514366000	-1.317129000
1	-1.476687000	-3.306092000	-0.924259000
6	-3.466252000	-2.713998000	-1.459493000
6	-4.299808000	-1.748244000	-2.007586000
1	-5.364243000	-1.920547000	-2.115324000
6	-3.724708000	-0.550402000	-2.410986000
6	-2.367962000	-0.306693000	-2.256519000
1	-1.935561000	0.632296000	-2.589378000
6	-4.609142000	0.520961000	-2.974389000
9	-5.613885000	0.016047000	-3.699517000
9	-3.934812000	1.370754000	-3.765609000
9	-5.169299000	1.263991000	-2.005179000

7	2.877265000	-0.403222000	-1.040485000
1	2.373151000	0.475536000	-1.113184000
7	-0.184661000	-1.022007000	-1.582384000
1	0.083508000	-0.059985000	-1.751717000
8	3.476030000	-3.498878000	-0.224250000
8	0.352308000	-4.243525000	-0.647451000
6	4.396821000	0.738225000	0.525998000
1	4.222366000	1.710125000	0.053166000
6	5.813577000	0.706411000	1.167033000
1	6.307818000	1.674069000	1.056638000
1	6.451996000	-0.030454000	0.670043000
6	5.665940000	0.320610000	2.645186000
1	6.649939000	0.204688000	3.104697000
6	4.875180000	-0.993543000	2.709719000
1	4.869561000	-1.389558000	3.728016000
1	5.356125000	-1.743158000	2.072409000
6	3.430000000	-0.704120000	2.235010000
1	3.061095000	-1.490769000	1.574065000
1	2.737397000	-0.659641000	3.075053000
6	3.564096000	1.651950000	2.558796000
1	2.683292000	1.699175000	3.201379000
1	3.637850000	2.607283000	2.030871000
6	4.850534000	1.394830000	3.398618000
1	4.568170000	0.979135000	4.371850000
6	5.609056000	2.665145000	3.635829000
1	5.973905000	3.182079000	2.747911000
6	5.840029000	3.197825000	4.831696000
1	5.487170000	2.720126000	5.742170000
1	6.388568000	4.127283000	4.945469000
7	3.343578000	0.601560000	1.552232000
6	5.215844000	-0.070521000	-1.715324000
6	6.257378000	-0.978418000	-2.074464000
6	6.488988000	-2.227376000	-1.432882000
1	5.830153000	-2.569596000	-0.644634000
6	7.522063000	-3.036073000	-1.827704000

1	7.678288000	-3.987316000	-1.331744000
6	8.381993000	-2.648067000	-2.881706000
1	9.195959000	-3.299862000	-3.178374000
6	8.179062000	-1.460208000	-3.529117000
1	8.813574000	-1.137896000	-4.346956000
6	7.115462000	-0.601757000	-3.148657000
6	5.984904000	1.352894000	-3.502230000
1	5.876894000	2.271445000	-4.073016000
6	5.089116000	1.084543000	-2.444140000
1	4.309283000	1.806117000	-2.225024000
7	6.967354000	0.559443000	-3.846529000
6	-4.080106000	-3.972864000	-0.922315000
9	-4.661211000	-3.758332000	0.275432000
9	-5.038821000	-4.452607000	-1.725460000
9	-3.182289000	-4.948189000	-0.741987000
8	0.889375000	1.226724000	0.080959000
6	-0.374607000	1.766962000	0.233004000
6	-0.388151000	3.248385000	0.671036000
1	0.268174000	3.749830000	-0.044902000
6	0.152057000	3.500269000	2.048984000
1	-0.462455000	3.190445000	2.890725000
1	1.928926000	4.466215000	1.446828000
6	1.306324000	4.120152000	2.268106000
1	1.669553000	4.307878000	3.273218000
1	-0.828192000	1.737870000	-0.763328000
1	1.407687000	1.109608000	0.905816000
6	-4.417487000	-0.482757000	1.044275000
6	-3.921368000	0.824479000	0.997573000
6	-4.821849000	1.891361000	0.878836000
6	-6.198448000	1.734400000	0.867698000
6	-6.671662000	0.431715000	0.946100000
6	-5.792752000	-0.661023000	1.018901000
6	-2.621288000	1.456748000	0.959254000
6	-2.829799000	2.782677000	0.783331000
1	-3.749261000	-1.335581000	1.080482000

1	-6.860924000	2.587039000	0.779912000
1	-7.740968000	0.254506000	0.934802000
1	-6.197126000	-1.666586000	1.049147000
8	-4.157620000	3.074669000	0.732775000
6	-1.826342000	3.843387000	0.492142000
1	-1.946001000	4.660590000	1.210500000
6	-2.004219000	4.428830000	-0.903830000
6	-1.448287000	5.681979000	-1.174500000
6	-2.656713000	3.745799000	-1.929812000
6	-1.536424000	6.238425000	-2.444461000
6	-2.747664000	4.303140000	-3.204063000
6	-2.187131000	5.548178000	-3.465024000
1	-0.942967000	6.222121000	-0.378575000
1	-3.117390000	2.781693000	-1.736472000
1	-1.103290000	7.213865000	-2.636907000
1	-3.266968000	3.764682000	-3.988405000
1	-2.262877000	5.982722000	-4.455468000
7	-1.317196000	0.896669000	1.023521000
8	0.559753000	0.304333000	2.609368000
8	-1.544721000	1.225739000	3.608730000
16	-0.893256000	0.404088000	2.602722000
6	-1.604881000	-1.211761000	2.695442000
6	-1.115602000	-2.208671000	1.859156000
6	-2.644054000	-1.448426000	3.586589000
6	-1.697307000	-3.466991000	1.906139000
6	-3.215532000	-2.714188000	3.617878000
6	-2.760323000	-3.732894000	2.776904000
1	-0.291852000	-1.996350000	1.185696000
1	-3.011401000	-0.652568000	4.223047000
1	-1.320627000	-4.251445000	1.257767000
1	-4.038277000	-2.909164000	4.297902000
6	-3.388727000	-5.097051000	2.820668000
1	-3.208951000	-5.645957000	1.895961000
1	-2.967921000	-5.681042000	3.644608000
1	-4.465921000	-5.028458000	2.981894000

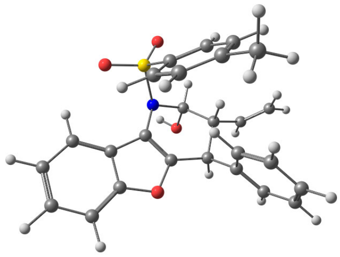

IV-NC

Sum of electronic and thermal Free Energies= -1757.785032

(0.0 kcal/mol)

6	-1.518277000	0.513479000	0.531068000
6	-2.636505000	0.879792000	-0.314153000
6	-3.915421000	0.384247000	-0.596706000
1	-4.275598000	-0.515425000	-0.116914000
6	-4.698840000	1.074796000	-1.509103000
1	-5.693243000	0.708629000	-1.738023000
6	-4.235355000	2.236959000	-2.145773000
1	-4.874275000	2.746452000	-2.857903000
6	-2.973912000	2.748435000	-1.875808000
1	-2.598815000	3.647618000	-2.349630000
6	-2.207906000	2.051347000	-0.954236000
6	-0.567230000	1.460391000	0.355644000
6	0.726092000	1.610382000	1.075753000
1	0.650646000	2.485308000	1.731780000
6	1.899037000	1.793544000	0.131685000
6	2.858674000	2.777416000	0.353371000
1	2.745020000	3.457820000	1.191650000
6	3.961404000	2.889277000	-0.491692000
1	4.701955000	3.660487000	-0.310164000
6	4.112679000	2.016551000	-1.563794000
1	4.969988000	2.105720000	-2.221799000
6	3.156409000	1.027472000	-1.789200000
1	3.262983000	0.343185000	-2.624951000
6	2.058947000	0.918514000	-0.944772000

1	1.313887000	0.147042000	-1.121146000
6	0.944736000	0.340378000	1.944955000
1	1.317439000	-0.447164000	1.285393000
6	1.966891000	0.595070000	3.014374000
1	1.661285000	1.261787000	3.817629000
6	3.189164000	0.076947000	2.997388000
1	3.513361000	-0.582390000	2.195679000
1	3.909950000	0.295697000	3.778248000
6	-0.367094000	-0.168712000	2.550367000
1	-0.185197000	-1.086624000	3.111489000
6	-0.184262000	-2.270661000	-0.282802000
6	1.084094000	-2.709320000	0.088650000
1	1.291101000	-2.986347000	1.116575000
6	2.073529000	-2.792834000	-0.880683000
1	3.065646000	-3.132229000	-0.601255000
6	1.811127000	-2.451060000	-2.211830000
6	0.527184000	-2.020691000	-2.555035000
1	0.308382000	-1.753024000	-3.583353000
6	-0.476518000	-1.923251000	-1.598024000
1	-1.473199000	-1.594578000	-1.870822000
6	2.889497000	-2.570718000	-3.252386000
1	2.676191000	-1.946333000	-4.120971000
1	2.967839000	-3.605615000	-3.598500000
1	3.862414000	-2.287730000	-2.846568000
7	-1.376482000	-0.504271000	1.508113000
8	-0.960864000	2.408666000	-0.535905000
8	-0.882687000	0.842252000	3.360245000
8	-2.728396000	-2.230388000	0.291205000
8	-1.117419000	-2.932234000	2.092566000
16	-1.447109000	-2.096291000	0.953549000
1	-1.682584000	0.515400000	3.791526000

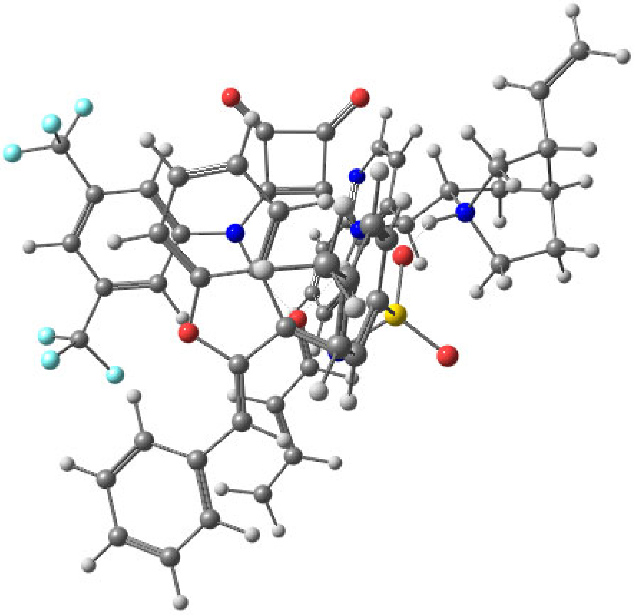

I_{enan}

Sum of electronic and thermal Free Energies= -3922.495507

(0.5 kcal/mol)

6	-3.095401000	-1.692862000	-1.111292000
1	-3.055420000	-1.317479000	-2.135882000
6	-1.521058000	-1.564150000	0.758421000
6	-2.156427000	-2.092698000	1.968179000
6	-0.765406000	-2.253874000	2.561889000
6	-0.231717000	-1.739430000	1.268730000
6	2.229762000	-1.748343000	1.133637000
6	2.506782000	-2.246512000	2.405002000
1	1.702702000	-2.491050000	3.090776000
6	3.830277000	-2.393335000	2.803547000
6	4.891318000	-2.074720000	1.970146000
1	5.917399000	-2.198077000	2.294038000
6	4.593722000	-1.600983000	0.694602000
6	3.287901000	-1.427958000	0.272519000
1	3.072178000	-1.045106000	-0.719454000
6	5.729928000	-1.234076000	-0.213304000
9	6.293487000	-0.064687000	0.150133000
9	5.349914000	-1.099527000	-1.488246000
9	6.709605000	-2.147863000	-0.182849000

7	-1.973333000	-1.053790000	-0.411764000
1	-1.232419000	-0.639646000	-1.001303000
7	0.944240000	-1.501758000	0.665617000
1	0.881780000	-1.057586000	-0.269982000
8	-3.285727000	-2.305019000	2.350454000
8	-0.329694000	-2.616111000	3.624904000
6	-4.433546000	-1.260340000	-0.506991000
1	-4.459962000	-1.567762000	0.541096000
6	-5.683784000	-1.738087000	-1.265628000
1	-6.092339000	-2.626428000	-0.784526000
1	-5.423393000	-2.016195000	-2.291792000
6	-6.715109000	-0.599412000	-1.287694000
1	-7.679009000	-0.972583000	-1.634719000
6	-6.198796000	0.505074000	-2.216431000
1	-6.842569000	1.384293000	-2.149420000
1	-6.208283000	0.164250000	-3.252464000
6	-4.760796000	0.859261000	-1.802189000
1	-4.016939000	0.477896000	-2.499172000
1	-4.595857000	1.929498000	-1.689305000
6	-5.537218000	0.672286000	0.512920000
1	-5.584769000	1.759278000	0.464945000
1	-5.194499000	0.377301000	1.505077000
6	-6.882269000	0.005868000	0.126381000
1	-7.644455000	0.786159000	0.062266000
6	-7.329334000	-1.007610000	1.144563000
1	-6.601244000	-1.760861000	1.445167000
6	-8.543671000	-1.027510000	1.680974000
1	-9.292743000	-0.286052000	1.415220000
1	-8.832906000	-1.783012000	2.403608000
7	-4.482454000	0.244529000	-0.460970000
6	-2.912523000	-3.203106000	-1.164274000
6	-1.894559000	-3.749213000	-2.009704000
6	-1.046976000	-2.964080000	-2.835536000
1	-1.140986000	-1.886338000	-2.852698000
6	-0.080474000	-3.551761000	-3.610304000

1	0.562120000	-2.932100000	-4.226076000
6	0.087038000	-4.955467000	-3.606918000
1	0.854692000	-5.405975000	-4.225747000
6	-0.713750000	-5.740342000	-2.820922000
1	-0.606597000	-6.818783000	-2.791989000
6	-1.713302000	-5.158837000	-2.001034000
6	-3.363020000	-5.458932000	-0.448601000
1	-3.939755000	-6.136904000	0.174917000
6	-3.625036000	-4.067328000	-0.377720000
1	-4.376955000	-3.725539000	0.322092000
7	-2.459662000	-5.997102000	-1.223067000
6	4.084727000	-2.836876000	4.213617000
9	3.822992000	-1.848089000	5.089507000
9	3.308748000	-3.870126000	4.566571000
9	5.355220000	-3.204883000	4.415693000
8	0.332441000	-0.095984000	-1.549044000
6	0.553833000	0.197417000	-2.780412000
6	1.762819000	0.191175000	-3.422606000
1	2.653975000	-0.104105000	-2.870977000
6	1.900710000	0.578743000	-4.804475000
1	0.976335000	0.846813000	-5.318697000
1	4.005002000	0.410705000	-5.017258000
6	3.055538000	0.658338000	-5.485751000
1	3.083695000	0.969553000	-6.524298000
1	-0.318354000	0.519696000	-3.375948000
1	-3.572346000	0.608962000	-0.143132000
6	-0.503163000	1.581978000	2.114352000
6	0.435501000	1.830718000	1.098951000
6	1.777547000	1.502786000	1.336706000
6	2.240505000	0.982953000	2.534925000
6	1.292296000	0.742506000	3.517261000
6	-0.064341000	1.032514000	3.307295000
6	0.406512000	2.382137000	-0.255301000
6	1.788769000	2.289871000	-0.717241000
1	-1.552736000	1.782032000	1.961848000

1	3.290372000	0.750075000	2.672449000
1	1.606003000	0.308408000	4.460399000
1	-0.780397000	0.817605000	4.091828000
8	2.578429000	1.735542000	0.268824000
6	2.260924000	2.703409000	-1.907493000
1	1.510416000	3.152090000	-2.550696000
6	3.627512000	2.691056000	-2.411556000
6	3.885239000	3.373853000	-3.609928000
6	4.687155000	2.017213000	-1.780773000
6	5.164010000	3.407973000	-4.151450000
6	5.959812000	2.041559000	-2.332802000
6	6.205293000	2.741711000	-3.513694000
1	3.068556000	3.878220000	-4.115780000
1	4.512669000	1.472751000	-0.862612000
1	5.343767000	3.944697000	-5.076235000
1	6.765655000	1.510860000	-1.838962000
1	7.203827000	2.759617000	-3.936131000
7	-0.481339000	2.956630000	-1.025083000
8	-2.684789000	3.855686000	-1.688741000
8	-2.666161000	2.188291000	0.145066000
16	-1.993174000	3.310583000	-0.530598000
6	-1.778217000	4.591441000	0.688023000
6	-0.960510000	5.678066000	0.380558000
6	-2.455332000	4.516455000	1.896897000
6	-0.818223000	6.692535000	1.312958000
6	-2.302220000	5.547754000	2.818854000
6	-1.484663000	6.643035000	2.543703000
1	-0.437054000	5.723600000	-0.568225000
1	-3.090467000	3.666952000	2.118469000
1	-0.179662000	7.540110000	1.085486000
1	-2.826278000	5.494978000	3.767231000
6	-1.300591000	7.749521000	3.544654000
1	-1.980076000	7.639295000	4.390258000
1	-0.277220000	7.751562000	3.929548000
1	-1.476510000	8.724606000	3.084762000

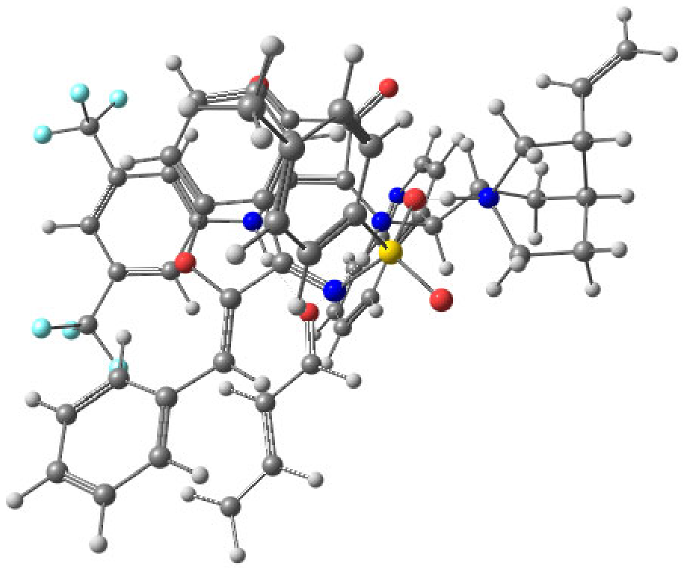

TS1$_{enan}$

Sum of electronic and thermal Free Energies= -3922.490310

(3.8 kcal/mol)

6	-3.142105000	-1.697031000	-1.114963000
1	-3.118471000	-1.427276000	-2.173381000
6	-1.482224000	-1.461874000	0.660857000
6	-2.074997000	-1.893404000	1.927002000
6	-0.662397000	-2.156392000	2.434519000
6	-0.177889000	-1.689661000	1.098531000
6	2.272467000	-1.806514000	0.840989000
6	2.590892000	-2.252465000	2.120476000
1	1.813267000	-2.429942000	2.855480000
6	3.926251000	-2.447078000	2.460045000
6	4.954934000	-2.221983000	1.561708000
1	5.990467000	-2.375024000	1.840621000
6	4.614398000	-1.791573000	0.280106000
6	3.299530000	-1.576992000	-0.085568000
1	3.052754000	-1.236214000	-1.085261000
6	5.724298000	-1.552362000	-0.699038000
9	6.563443000	-0.594784000	-0.262542000
9	5.283551000	-1.168667000	-1.902282000
9	6.472405000	-2.650366000	-0.883704000

7	-2.003766000	-0.991912000	-0.502758000
1	-1.302437000	-0.635747000	-1.161609000
7	0.974253000	-1.535455000	0.422208000
1	0.886806000	-1.131440000	-0.520667000
8	-3.189878000	-1.997639000	2.383767000
8	-0.189091000	-2.562139000	3.462835000
6	-4.475165000	-1.211772000	-0.539683000
1	-4.518714000	-1.468553000	0.521329000
6	-5.715353000	-1.715596000	-1.301155000
1	-6.151260000	-2.568836000	-0.782290000
1	-5.430901000	-2.057213000	-2.301555000
6	-6.726609000	-0.566487000	-1.420825000
1	-7.682257000	-0.944398000	-1.785401000
6	-6.160697000	0.483621000	-2.384480000
1	-6.797681000	1.370432000	-2.388653000
1	-6.134800000	0.088548000	-3.400893000
6	-4.735601000	0.849182000	-1.933656000
1	-3.968393000	0.434602000	-2.585614000
1	-4.567484000	1.922777000	-1.859063000
6	-5.595294000	0.771439000	0.357147000
1	-5.622028000	1.855335000	0.262079000
1	-5.288982000	0.516974000	1.371992000
6	-6.934996000	0.106480000	-0.044187000
1	-7.684623000	0.891549000	-0.168843000
6	-7.426619000	-0.855029000	1.003417000
1	-6.714525000	-1.597205000	1.364529000
6	-8.659013000	-0.843006000	1.497240000
1	-9.392687000	-0.110525000	1.170592000
1	-8.978919000	-1.561311000	2.244504000
7	-4.511890000	0.290556000	-0.559521000
6	-2.969333000	-3.208056000	-1.016501000
6	-1.931389000	-3.844993000	-1.770112000
6	-1.052118000	-3.155812000	-2.646709000
1	-1.133789000	-2.085507000	-2.782380000
6	-0.065051000	-3.827579000	-3.321400000

1	0.601215000	-3.281115000	-3.979786000
6	0.092457000	-5.223371000	-3.165188000
1	0.876770000	-5.740004000	-3.706601000
6	-0.741208000	-5.917075000	-2.329365000
1	-0.645345000	-6.987097000	-2.183864000
6	-1.762693000	-5.247558000	-1.610094000
6	-3.468430000	-5.379794000	-0.096454000
1	-4.074214000	-5.987197000	0.570635000
6	-3.717591000	-3.986397000	-0.175181000
1	-4.492481000	-3.573972000	0.457984000
7	-2.542697000	-5.998575000	-0.777980000
6	4.223873000	-2.837502000	3.876835000
9	3.951295000	-1.828737000	4.725704000
9	3.484186000	-3.880087000	4.279028000
9	5.507368000	-3.165487000	4.062657000
8	0.286778000	-0.231621000	-1.892755000
6	0.565577000	0.434955000	-2.924928000
6	1.854091000	0.711914000	-3.378659000
1	2.686652000	0.201446000	-2.901561000
6	2.078858000	1.375388000	-4.639931000
1	1.196096000	1.782145000	-5.133685000
1	4.186035000	1.170460000	-4.744415000
6	3.280600000	1.554804000	-5.207676000
1	3.392262000	2.080996000	-6.149336000
1	-0.268622000	0.886325000	-3.485785000
1	-3.617046000	0.672903000	-0.214508000
6	-0.750528000	1.516806000	2.238906000
6	0.212426000	1.679910000	1.225194000
6	1.534768000	1.296868000	1.496452000
6	1.953807000	0.784330000	2.717500000
6	0.983481000	0.629364000	3.693792000
6	-0.353951000	0.989837000	3.455091000
6	0.228271000	2.163803000	-0.153073000
6	1.581143000	2.034683000	-0.565990000
1	-1.785827000	1.772177000	2.064687000

1	2.990453000	0.515579000	2.882350000
1	1.261474000	0.214514000	4.656306000
1	-1.089512000	0.842459000	4.237611000
8	2.350313000	1.467258000	0.432498000
6	2.113678000	2.498146000	-1.749630000
1	1.412648000	3.088751000	-2.328772000
6	3.528434000	2.702525000	-2.050269000
6	3.862784000	3.701890000	-2.974636000
6	4.561037000	1.934207000	-1.487007000
6	5.190225000	3.959024000	-3.300583000
6	5.882475000	2.180260000	-1.830287000
6	6.202794000	3.197786000	-2.730162000
1	3.070939000	4.286174000	-3.430695000
1	4.325138000	1.141381000	-0.786701000
1	5.428621000	4.744777000	-4.008672000
1	6.670387000	1.576321000	-1.396349000
1	7.239083000	3.387225000	-2.987257000
7	-0.671148000	2.696019000	-0.989527000
8	-2.744788000	3.871303000	-1.624547000
8	-2.898729000	2.279304000	0.260370000
16	-2.077015000	3.266486000	-0.475708000
6	-1.681186000	4.543324000	0.710512000
6	-0.656739000	5.440561000	0.414019000
6	-2.407779000	4.648189000	1.887962000
6	-0.363210000	6.448958000	1.318306000
6	-2.100861000	5.668742000	2.783586000
6	-1.079414000	6.578791000	2.514279000
1	-0.090069000	5.342665000	-0.505845000
1	-3.195694000	3.937094000	2.106565000
1	0.438052000	7.147391000	1.098174000
1	-2.662778000	5.751886000	3.708027000
6	-0.734975000	7.677213000	3.482124000
1	-1.392767000	7.664352000	4.351762000
1	0.295266000	7.574454000	3.832451000
1	-0.818983000	8.656259000	3.003827000

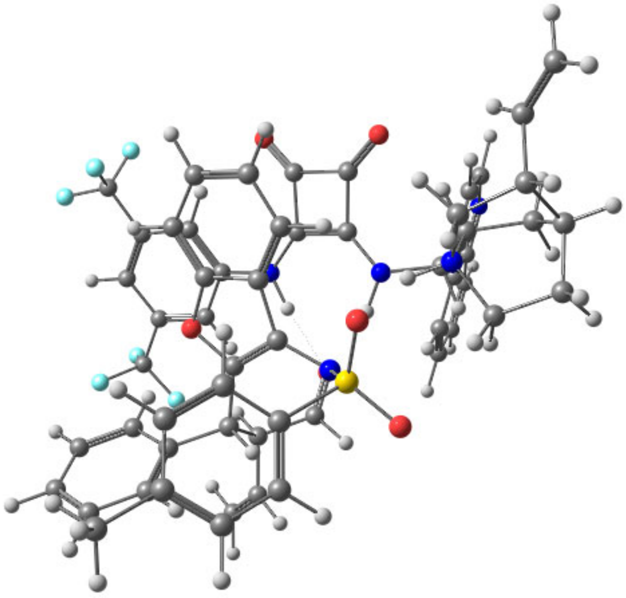

II$_{enan}$

Sum of electronic and thermal Free Energies= -3922.518353

(-13.8 kcal/mol)

6	-3.637546000	-0.574654000	-0.910924000
1	-3.620543000	0.025254000	-1.821834000
6	-1.835812000	-1.484682000	0.500331000
6	-2.444818000	-2.060294000	1.710893000
6	-1.125804000	-2.768912000	1.970707000
6	-0.612725000	-2.107512000	0.741303000
6	1.773551000	-2.578131000	0.338996000
6	2.085826000	-3.094462000	1.593293000
1	1.329777000	-3.177418000	2.365347000
6	3.388779000	-3.512863000	1.850492000
6	4.393241000	-3.426014000	0.902143000
1	5.406901000	-3.736160000	1.124475000
6	4.055375000	-2.925398000	-0.353782000
6	2.768097000	-2.518142000	-0.645345000
1	2.518404000	-2.160790000	-1.637132000
6	5.131292000	-2.817721000	-1.392806000
9	6.155011000	-2.059971000	-0.963157000
9	4.689054000	-2.265985000	-2.531386000
9	5.650930000	-4.012179000	-1.708283000

7	-2.248923000	-0.599134000	-0.431169000
1	-1.553068000	-0.248690000	-1.083870000
7	0.520137000	-2.067053000	0.011135000
1	0.467189000	-1.569946000	-0.878554000
8	-3.509596000	-1.995212000	2.278333000
8	-0.717316000	-3.516954000	2.819918000
6	-4.555103000	0.127235000	0.094949000
1	-4.439229000	-0.357132000	1.066185000
6	-6.039208000	0.200000000	-0.309075000
1	-6.585940000	-0.618135000	0.157848000
1	-6.146240000	0.083056000	-1.392264000
6	-6.609979000	1.559363000	0.118187000
1	-7.697693000	1.549301000	0.042938000
6	-6.012756000	2.634531000	-0.792299000
1	-6.284973000	3.628441000	-0.431653000
1	-6.399627000	2.530499000	-1.806918000
6	-4.485200000	2.470994000	-0.805304000
1	-4.114160000	2.040194000	-1.733336000
1	-3.953331000	3.402792000	-0.637221000
6	-4.661385000	2.051584000	1.606243000
1	-4.343134000	3.088028000	1.696252000
1	-4.204600000	1.472551000	2.408927000
6	-6.201780000	1.889950000	1.572331000
1	-6.648751000	2.854046000	1.826247000
6	-6.692043000	0.870267000	2.564447000
1	-6.211342000	-0.107725000	2.550746000
6	-7.663198000	1.102144000	3.439713000
1	-8.157710000	2.068567000	3.494652000
1	-7.997345000	0.337450000	4.132758000
7	-4.083835000	1.546110000	0.314337000
6	-4.099017000	-1.978187000	-1.280829000
6	-3.632109000	-2.571355000	-2.496646000
6	-2.755291000	-1.926244000	-3.408711000
1	-2.379739000	-0.931661000	-3.202329000
6	-2.343846000	-2.560916000	-4.552382000

1	-1.667097000	-2.056411000	-5.232824000
6	-2.788505000	-3.868894000	-4.850821000
1	-2.456651000	-4.354925000	-5.761243000
6	-3.627578000	-4.520702000	-3.987484000
1	-3.979516000	-5.527576000	-4.181406000
6	-4.059233000	-3.895346000	-2.790970000
6	-5.251106000	-4.049315000	-0.847785000
1	-5.884793000	-4.638862000	-0.190749000
6	-4.889506000	-2.735152000	-0.459090000
1	-5.237635000	-2.369553000	0.498187000
7	-4.871808000	-4.614332000	-1.962442000
6	3.678023000	-4.038402000	3.225870000
9	3.376738000	-3.132049000	4.172490000
9	2.952645000	-5.131275000	3.505198000
9	4.964255000	-4.365345000	3.391929000
8	0.102859000	-0.429946000	-2.389281000
6	0.689665000	0.550566000	-2.797029000
6	2.157235000	0.793247000	-2.568128000
1	2.635494000	-0.158869000	-2.329107000
6	2.780396000	1.401873000	-3.792584000
1	2.336228000	2.334635000	-4.138086000
1	4.293300000	-0.037543000	-4.087964000
6	3.826145000	0.884068000	-4.424073000
1	4.258351000	1.368991000	-5.292801000
1	0.143042000	1.324537000	-3.358201000
1	-3.057167000	1.567361000	0.388743000
6	-0.959634000	0.871017000	2.456103000
6	0.077713000	0.956444000	1.519255000
6	1.307870000	0.362946000	1.829938000
6	1.555190000	-0.331274000	3.006419000
6	0.501175000	-0.428797000	3.905008000
6	-0.739524000	0.171591000	3.634071000
6	0.258228000	1.536263000	0.194954000
6	1.555086000	1.282330000	-0.134584000
1	-1.902422000	1.371345000	2.276510000

1	2.525706000	-0.768989000	3.206337000
1	0.642442000	-0.972675000	4.832198000
1	-1.538596000	0.085572000	4.362389000
8	2.204211000	0.549876000	0.826239000
6	2.299314000	1.760339000	-1.339795000
1	1.806802000	2.695479000	-1.625895000
6	3.755815000	2.059795000	-1.049783000
6	4.200116000	3.380107000	-1.052806000
6	4.671514000	1.037363000	-0.791412000
6	5.536636000	3.679985000	-0.797708000
6	6.005958000	1.332858000	-0.540222000
6	6.442539000	2.656391000	-0.542506000
1	3.493276000	4.178731000	-1.256017000
1	4.337730000	0.004883000	-0.774788000
1	5.867931000	4.712971000	-0.805571000
1	6.706197000	0.528732000	-0.343090000
1	7.484379000	2.885380000	-0.347293000
7	-0.629536000	2.175413000	-0.683217000
8	-1.854490000	4.229688000	-1.340428000
8	-1.979419000	3.497886000	1.029984000
16	-1.170608000	3.588562000	-0.210484000
6	0.240848000	4.616705000	0.194643000
6	0.712520000	5.521387000	-0.749354000
6	0.912088000	4.433976000	1.401451000
6	1.862485000	6.255495000	-0.476074000
6	2.069950000	5.159822000	1.650947000
6	2.562564000	6.077553000	0.718689000
1	0.175475000	5.654016000	-1.681662000
1	0.529723000	3.738057000	2.140443000
1	2.227910000	6.968308000	-1.208906000
1	2.602912000	5.009070000	2.584618000
6	3.818189000	6.854936000	1.006826000
1	4.570660000	6.221627000	1.481414000
1	4.244942000	7.274750000	0.094412000
1	3.612007000	7.684404000	1.689733000

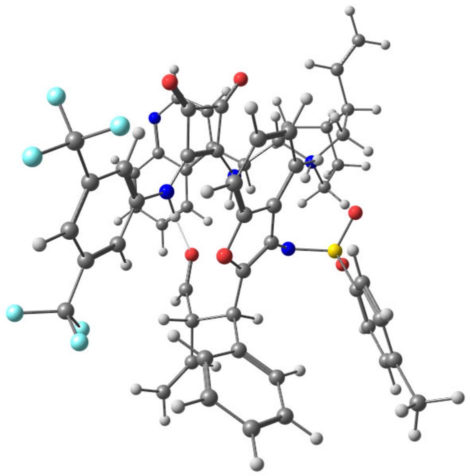

TS-Rot$_{enan}$

Sum of electronic and thermal Free Energies= -3922.510998

(-9.2 kcal/mol)

6	3.548081000	-0.539030000	0.898054000
1	3.504776000	0.147012000	1.744376000
6	1.796384000	-1.543721000	-0.519631000
6	2.425967000	-2.153385000	-1.705253000
6	1.121517000	-2.891405000	-1.951472000
6	0.589398000	-2.202910000	-0.747293000
6	-1.795765000	-2.685240000	-0.327658000
6	-2.076260000	-3.332276000	-1.526533000
1	-1.297523000	-3.506360000	-2.261007000
6	-3.380450000	-3.746732000	-1.785367000
6	-4.414658000	-3.532207000	-0.891433000
1	-5.428325000	-3.840727000	-1.116507000
6	-4.108378000	-2.903722000	0.314862000
6	-2.821826000	-2.495887000	0.607105000
1	-2.596990000	-2.031306000	1.559349000
6	-5.223786000	-2.642300000	1.282808000
9	-6.133858000	-1.797009000	0.768588000
9	-4.792118000	-2.096330000	2.429049000
9	-5.885645000	-3.762073000	1.603022000
7	2.172001000	-0.618841000	0.389131000
1	1.453885000	-0.251654000	1.006888000

7	-0.544012000	-2.158260000	-0.017678000
1	-0.507626000	-1.569858000	0.812712000
8	3.494380000	-2.098687000	-2.267498000
8	0.733466000	-3.671479000	-2.781491000
6	4.482263000	0.071197000	-0.150277000
1	4.351037000	-0.468529000	-1.089344000
6	5.969772000	0.130825000	0.237085000
1	6.486268000	-0.738656000	-0.168308000
1	6.085650000	0.098821000	1.324979000
6	6.573443000	1.434165000	-0.305495000
1	7.661741000	1.395063000	-0.251639000
6	6.027690000	2.592738000	0.531198000
1	6.315215000	3.548216000	0.087785000
1	6.436763000	2.557846000	1.541861000
6	4.497799000	2.469963000	0.592230000
1	4.139963000	2.111263000	1.555250000
1	3.985773000	3.402719000	0.376781000
6	4.608600000	1.885250000	-1.787489000
1	4.328010000	2.925676000	-1.938220000
1	4.111966000	1.273166000	-2.541034000
6	6.142751000	1.665331000	-1.772879000
1	6.619724000	2.587645000	-2.112974000
6	6.571591000	0.550137000	-2.687821000
1	6.057675000	-0.405274000	-2.583244000
6	7.528882000	0.673088000	-3.599681000
1	8.055697000	1.612639000	-3.745395000
1	7.818512000	-0.158015000	-4.233858000
7	4.044596000	1.485150000	-0.453949000
6	4.002985000	-1.899409000	1.406272000
6	3.583319000	-2.349724000	2.697721000
6	2.769396000	-1.589647000	3.579600000
1	2.408246000	-0.610688000	3.290545000
6	2.406682000	-2.089858000	4.803439000
1	1.782966000	-1.496519000	5.462659000
6	2.836550000	-3.372022000	5.214797000

1	2.543069000	-3.750840000	6.187225000
6	3.614340000	-4.132989000	4.384090000
1	3.954087000	-5.123254000	4.665582000
6	3.997834000	-3.646905000	3.108998000
6	5.091310000	-4.031646000	1.139137000
1	5.681180000	-4.701984000	0.519853000
6	4.739118000	-2.756145000	0.632815000
1	5.053705000	-2.494707000	-0.369978000
7	4.753296000	-4.468400000	2.323042000
6	-3.640556000	-4.388378000	-3.116251000
9	-3.400484000	-3.533317000	-4.126045000
9	-2.848869000	-5.450511000	-3.322824000
9	-4.904658000	-4.805086000	-3.248433000
8	-0.196799000	-0.467869000	2.378483000
6	-1.034165000	-0.023387000	3.130755000
6	-2.321077000	0.653728000	2.704288000
1	-3.013141000	-0.158978000	2.448178000
6	-2.913450000	1.380515000	3.886005000
1	-2.334290000	2.223039000	4.262295000
1	-4.669934000	0.230989000	4.087661000
6	-4.067062000	1.057397000	4.455293000
1	-4.455667000	1.612764000	5.302273000
1	-0.874344000	-0.107520000	4.220417000
1	3.016858000	1.531158000	-0.507490000
6	0.864291000	0.844106000	-2.500045000
6	-0.103604000	0.904053000	-1.488795000
6	-1.319377000	0.239252000	-1.691134000
6	-1.623307000	-0.487548000	-2.834009000
6	-0.636679000	-0.555558000	-3.809219000
6	0.591488000	0.104896000	-3.642839000
6	-0.205990000	1.498963000	-0.159849000
6	-1.444245000	1.153953000	0.292160000
1	1.790889000	1.394941000	-2.403714000
1	-2.584899000	-0.972414000	-2.953156000
1	-0.822945000	-1.122781000	-4.714290000

1	1.337290000	0.039670000	-4.427655000
8	-2.131061000	0.378715000	-0.610904000
6	-2.158403000	1.637831000	1.509987000
1	-1.533667000	2.459267000	1.874567000
6	-3.512707000	2.209718000	1.120169000
6	-3.693328000	3.588673000	1.078250000
6	-4.583403000	1.374378000	0.792363000
6	-4.925794000	4.132821000	0.722327000
6	-5.814205000	1.913188000	0.438683000
6	-5.990052000	3.296118000	0.404945000
1	-2.861896000	4.241966000	1.324681000
1	-4.454099000	0.296114000	0.804224000
1	-5.051658000	5.210519000	0.700692000
1	-6.637943000	1.252883000	0.189293000
1	-6.952236000	3.715493000	0.132145000
7	0.719573000	2.207344000	0.617860000
8	1.946638000	4.302235000	1.112040000
8	1.971162000	3.457962000	-1.220984000
16	1.216137000	3.599888000	0.049141000
6	-0.207431000	4.618865000	-0.347391000
6	-0.546981000	5.665150000	0.501736000
6	-0.977152000	4.342772000	-1.476323000
6	-1.661052000	6.448229000	0.211171000
6	-2.095416000	5.121645000	-1.742303000
6	-2.451742000	6.186171000	-0.908213000
1	0.066818000	5.870631000	1.371021000
1	-0.697882000	3.539501000	-2.149569000
1	-1.919629000	7.273030000	0.868158000
1	-2.701969000	4.901777000	-2.615448000
6	-3.650309000	7.036537000	-1.231839000
1	-4.473551000	6.428166000	-1.612043000
1	-3.999394000	7.584807000	-0.355222000
1	-3.403189000	7.770284000	-2.004925000

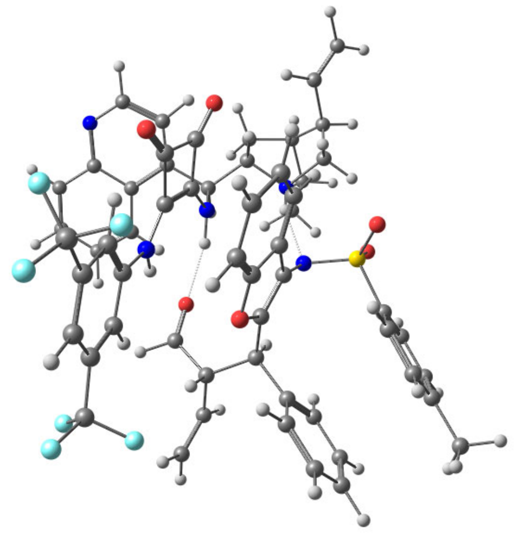

Rot-II$_{enan}$

Sum of electronic and thermal Free Energies= -3922.518784

(-14.1 kcal/mol)

6	3.466499000	-0.599056000	1.027415000
1	3.399530000	-0.064998000	1.976361000
6	1.744911000	-1.589935000	-0.424111000
6	2.388437000	-2.080590000	-1.656104000
6	1.143553000	-2.924656000	-1.901204000
6	0.589614000	-2.328813000	-0.655444000
6	-1.758623000	-2.968317000	-0.204594000
6	-1.974885000	-3.725725000	-1.355745000
1	-1.160465000	-3.939855000	-2.039533000
6	-3.256972000	-4.178137000	-1.636486000
6	-4.338081000	-3.908544000	-0.806581000
1	-5.332833000	-4.258454000	-1.049548000
6	-4.095441000	-3.174901000	0.347147000
6	-2.826660000	-2.711115000	0.654490000
1	-2.667560000	-2.134883000	1.559007000
6	-5.213650000	-2.790428000	1.269554000
9	-5.490533000	-1.475479000	1.180150000
9	-4.902109000	-3.016883000	2.556068000
9	-6.346587000	-3.447058000	1.010781000
7	2.086740000	-0.702734000	0.533782000

1	1.359029000	-0.396861000	1.171899000
7	-0.523777000	-2.403231000	0.107575000
1	-0.471137000	-1.943742000	1.009676000
8	3.428993000	-1.897631000	-2.237518000
8	0.805422000	-3.716539000	-2.739975000
6	4.323919000	0.239298000	0.077493000
1	4.249727000	-0.189947000	-0.923744000
6	5.796532000	0.402261000	0.501169000
1	6.409649000	-0.342778000	-0.004623000
1	5.905786000	0.234104000	1.577606000
6	6.262316000	1.822288000	0.149928000
1	7.347793000	1.891967000	0.227735000
6	5.581659000	2.804459000	1.108060000
1	5.749407000	3.831554000	0.776307000
1	5.996050000	2.707180000	2.112733000
6	4.078919000	2.487549000	1.138199000
1	3.789481000	1.940328000	2.033375000
1	3.449885000	3.370367000	1.051816000
6	4.278820000	2.254771000	-1.299870000
1	3.914920000	3.279568000	-1.312585000
1	3.839412000	1.715866000	-2.139788000
6	5.825807000	2.183324000	-1.288069000
1	6.211040000	3.181816000	-1.509006000
6	6.370635000	1.232781000	-2.319364000
1	5.938756000	0.232904000	-2.352272000
6	7.334318000	1.548673000	-3.176460000
1	7.780191000	2.539976000	-3.186644000
1	7.708939000	0.832213000	-3.899762000
7	3.740316000	1.624191000	-0.045660000
6	4.030487000	-1.984620000	1.312098000
6	3.588629000	-2.694750000	2.473656000
6	2.654505000	-2.178021000	3.410259000
1	2.219546000	-1.196573000	3.270700000
6	2.271694000	-2.918617000	4.499193000
1	1.555651000	-2.508537000	5.202773000

6	2.801834000	-4.211221000	4.714318000
1	2.492283000	-4.781963000	5.582418000
6	3.699794000	-4.739370000	3.825949000
1	4.120677000	-5.729654000	3.958069000
6	4.106255000	-4.001822000	2.685618000
6	5.344357000	-3.929332000	0.766808000
1	6.033671000	-4.422079000	0.086540000
6	4.892512000	-2.621010000	0.461056000
1	5.230966000	-2.164262000	-0.459533000
7	4.985578000	-4.599157000	1.829041000
6	-3.467860000	-4.891878000	-2.939266000
9	-3.455124000	-4.027555000	-3.970754000
9	-2.506806000	-5.791440000	-3.184634000
9	-4.639612000	-5.535817000	-2.988134000
8	0.005376000	-0.215123000	2.596366000
6	-1.157909000	-0.274235000	2.921641000
6	-2.231857000	0.701747000	2.513084000
1	-3.020416000	0.104311000	2.035531000
6	-2.798206000	1.266781000	3.796487000
1	-2.166646000	1.984559000	4.316847000
1	-4.622191000	0.207799000	3.787801000
6	-3.974433000	0.914884000	4.299579000
1	-4.336965000	1.331825000	5.233031000
1	-1.501267000	-1.086162000	3.589419000
1	2.693369000	1.605050000	-0.112502000
6	0.555996000	0.491766000	-2.816614000
6	-0.251065000	0.717600000	-1.695282000
6	-1.466796000	0.039718000	-1.586337000
6	-1.952095000	-0.829062000	-2.554711000
6	-1.133696000	-1.046453000	-3.655764000
6	0.107994000	-0.399429000	-3.780316000
6	-0.133020000	1.514100000	-0.482465000
6	-1.255955000	1.243900000	0.240303000
1	1.491739000	1.025468000	-2.932017000
1	-2.916728000	-1.312415000	-2.450678000

1	-1.459443000	-1.731213000	-4.430862000
1	0.722858000	-0.602148000	-4.650271000
8	-2.074338000	0.339166000	-0.404487000
6	-1.744829000	1.798916000	1.539017000
1	-0.885850000	2.310016000	1.983970000
6	-2.871381000	2.810460000	1.367059000
6	-2.785357000	4.055152000	1.987371000
6	-4.046743000	2.473400000	0.689630000
6	-3.856827000	4.943756000	1.949095000
6	-5.115010000	3.362988000	0.645152000
6	-5.027189000	4.598763000	1.282655000
1	-1.874358000	4.329480000	2.511048000
1	-4.133535000	1.507199000	0.203876000
1	-3.771876000	5.908730000	2.438154000
1	-6.021905000	3.086424000	0.118084000
1	-5.863906000	5.288433000	1.254944000
7	0.918773000	2.358459000	-0.087965000
8	2.141372000	4.488783000	-0.332943000
8	1.236847000	3.435880000	-2.397223000
16	1.071780000	3.692845000	-0.957641000
6	-0.423905000	4.672183000	-0.834416000
6	-0.413831000	5.839984000	-0.078918000
6	-1.556620000	4.299437000	-1.552218000
6	-1.547496000	6.642371000	-0.051909000
6	-2.689553000	5.103312000	-1.498964000
6	-2.698711000	6.287337000	-0.759437000
1	0.483727000	6.122178000	0.458698000
1	-1.550236000	3.402881000	-2.163034000
1	-1.539512000	7.560724000	0.528030000
1	-3.577751000	4.809568000	-2.049604000
6	-3.913140000	7.174632000	-0.733303000
1	-4.798793000	6.646235000	-1.089314000
1	-4.113754000	7.541356000	0.275980000
1	-3.764487000	8.048707000	-1.374527000

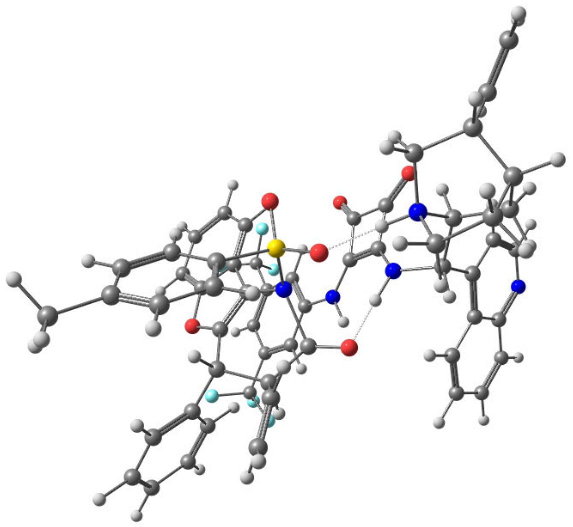

TS2$_{enan}$

Sum of electronic and thermal Free Energies= -3922.497824

(-0.9 kcal/mol)

6	3.885718000	-0.412765000	1.000106000
1	3.851168000	0.221219000	1.889309000
6	2.115989000	-1.286564000	-0.458854000
6	2.731039000	-1.919478000	-1.641350000
6	1.385934000	-2.579961000	-1.919497000
6	0.899661000	-1.939478000	-0.674392000
6	-1.460276000	-2.475861000	-0.098787000
6	-1.792202000	-3.170494000	-1.263014000
1	-1.048197000	-3.351066000	-2.029743000
6	-3.093908000	-3.618104000	-1.430656000
6	-4.076468000	-3.406244000	-0.472430000
1	-5.089073000	-3.763284000	-0.621876000
6	-3.723154000	-2.726258000	0.685849000
6	-2.433330000	-2.256086000	0.882162000
1	-2.169623000	-1.727105000	1.792317000
6	-4.788209000	-2.483626000	1.714253000
9	-5.687076000	-1.577086000	1.295892000
9	-4.291456000	-2.030426000	2.874508000
9	-5.478084000	-3.599635000	1.991149000
7	2.522262000	-0.405484000	0.470175000

1	1.829446000	0.118527000	1.052611000
7	-0.183914000	-1.962104000	0.129031000
1	-0.040283000	-1.554746000	1.046107000
8	3.820903000	-1.940156000	-2.162546000
8	0.945570000	-3.288447000	-2.788518000
6	4.879546000	0.209913000	0.016576000
1	4.832692000	-0.348966000	-0.919836000
6	6.327772000	0.335074000	0.519863000
1	6.918041000	-0.507820000	0.162527000
1	6.355068000	0.308523000	1.614067000
6	6.911455000	1.667645000	0.024311000
1	7.991326000	1.684034000	0.173927000
6	6.240228000	2.804679000	0.801795000
1	6.521559000	3.770003000	0.376066000
1	6.561682000	2.794091000	1.844079000
6	4.718082000	2.606010000	0.729923000
1	4.296446000	2.232575000	1.660713000
1	4.176840000	3.509304000	0.456886000
6	5.064274000	2.013606000	-1.624108000
1	4.761419000	3.043193000	-1.811330000
1	4.655962000	1.380305000	-2.411918000
6	6.601484000	1.872361000	-1.477803000
1	7.059781000	2.816646000	-1.781314000
6	7.166105000	0.779932000	-2.344977000
1	6.697841000	-0.201925000	-2.279360000
6	8.192491000	0.952371000	-3.169408000
1	8.680008000	1.918273000	-3.273986000
1	8.580363000	0.137117000	-3.770783000
7	4.411663000	1.597421000	-0.340570000
6	4.260111000	-1.815034000	1.452094000
6	3.633468000	-2.350647000	2.622573000
6	2.675455000	-1.644548000	3.400971000
1	2.357091000	-0.646445000	3.118458000
6	2.118809000	-2.225069000	4.512383000
1	1.387707000	-1.675817000	5.095011000

6	2.484967000	-3.532865000	4.905519000
1	2.036760000	-3.973746000	5.788691000
6	3.395390000	-4.240882000	4.167793000
1	3.690058000	-5.249050000	4.436409000
6	3.981116000	-3.673272000	3.008264000
6	5.381522000	-3.933174000	1.220416000
1	6.065238000	-4.565546000	0.660403000
6	5.111655000	-2.623342000	0.749582000
1	5.579296000	-2.302191000	-0.172600000
7	4.859981000	-4.445121000	2.302833000
6	-3.457243000	-4.405164000	-2.656011000
9	-4.658600000	-4.050628000	-3.139722000
9	-2.571227000	-4.262714000	-3.646832000
9	-3.533476000	-5.721248000	-2.389714000
8	0.935368000	1.187657000	2.000290000
6	-0.214629000	1.134482000	1.461242000
6	-1.293337000	2.124287000	1.955777000
1	-1.693565000	1.615374000	2.840147000
6	-0.686513000	3.423125000	2.406704000
1	0.195067000	3.747959000	1.862762000
1	-2.017473000	3.844421000	3.992326000
6	-1.158067000	4.160757000	3.406214000
1	-0.697931000	5.104981000	3.680666000
1	-0.708933000	0.149453000	1.385366000
1	3.386388000	1.608709000	-0.467163000
6	-1.275997000	-0.504334000	-2.996017000
6	-1.870335000	0.152327000	-1.914255000
6	-3.267008000	0.154213000	-1.787506000
6	-4.109060000	-0.502837000	-2.672356000
6	-3.500069000	-1.157999000	-3.736930000
6	-2.105233000	-1.152386000	-3.899485000
6	-1.412574000	0.936011000	-0.794383000
6	-2.513090000	1.360316000	-0.130752000
1	-0.200798000	-0.481151000	-3.127821000
1	-5.184341000	-0.496229000	-2.536808000

1	-4.116493000	-1.682561000	-4.457816000
1	-1.669554000	-1.675518000	-4.743295000
8	-3.658160000	0.890978000	-0.703661000
6	-2.518026000	2.354001000	0.991015000
1	-2.378741000	3.336516000	0.521966000
6	-3.819594000	2.391358000	1.757242000
6	-4.471988000	3.597109000	2.002857000
6	-4.361458000	1.210716000	2.269310000
6	-5.646219000	3.623714000	2.753964000
6	-5.534918000	1.230953000	3.010714000
6	-6.179493000	2.442558000	3.258139000
1	-4.055550000	4.519006000	1.606872000
1	-3.857473000	0.269151000	2.072381000
1	-6.144729000	4.568610000	2.941185000
1	-5.947912000	0.303704000	3.392657000
1	-7.095257000	2.462452000	3.838590000
7	-0.132994000	1.236197000	-0.296934000
8	1.902279000	2.687617000	-0.438460000
8	0.864225000	1.818531000	-2.535784000
16	0.687786000	2.329754000	-1.179873000
6	-0.315048000	3.798911000	-1.304903000
6	-0.027295000	4.906029000	-0.518808000
6	-1.437830000	3.777964000	-2.134135000
6	-0.896060000	5.993386000	-0.533276000
6	-2.297923000	4.865267000	-2.127406000
6	-2.048921000	5.980956000	-1.318123000
1	0.869322000	4.921573000	0.088642000
1	-1.632009000	2.928223000	-2.779094000
1	-0.673431000	6.860948000	0.079293000
1	-3.178107000	4.852547000	-2.762603000
6	-3.008616000	7.138793000	-1.316798000
1	-3.992862000	6.822624000	-0.961172000
1	-2.657948000	7.947280000	-0.674594000
1	-3.141171000	7.535458000	-2.326352000

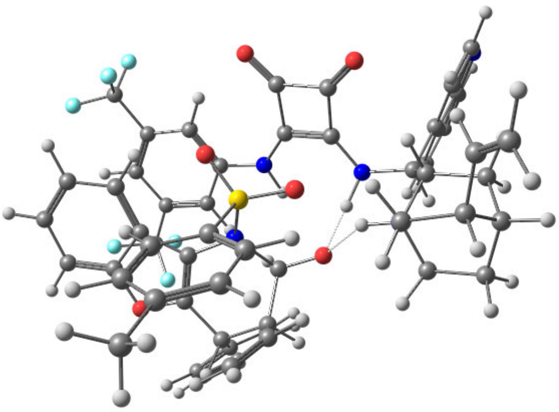

III_{enan}

Sum of electronic and thermal Free Energies= -3922.507348

(-6.9 kcal/mol)

6	3.855787000	-0.557779000	0.985911000
1	3.840424000	0.062759000	1.885374000
6	2.078166000	-1.316779000	-0.520490000
6	2.680636000	-1.922259000	-1.725997000
6	1.308700000	-2.484383000	-2.065564000
6	0.836442000	-1.901605000	-0.790851000
6	-1.541603000	-2.391798000	-0.266243000
6	-1.892327000	-2.957819000	-1.494096000
1	-1.154455000	-3.075740000	-2.278661000
6	-3.202717000	-3.361717000	-1.700779000
6	-4.179275000	-3.231418000	-0.721530000
1	-5.198907000	-3.552057000	-0.901554000
6	-3.806530000	-2.686599000	0.499860000
6	-2.507060000	-2.264986000	0.737702000
1	-2.232157000	-1.853185000	1.702624000
6	-4.858173000	-2.529615000	1.557273000
9	-5.704125000	-1.526939000	1.273571000
9	-4.336587000	-2.269930000	2.765923000
9	-5.608741000	-3.634032000	1.683376000
7	2.492603000	-0.503362000	0.459292000
1	1.788633000	-0.004348000	1.084899000
7	-0.258068000	-1.918351000	0.000027000

1	-0.111059000	-1.549406000	0.933928000
8	3.778858000	-1.984533000	-2.227770000
8	0.848722000	-3.106798000	-2.990759000
6	4.871740000	0.047689000	0.013067000
1	4.809211000	-0.493278000	-0.932698000
6	6.323318000	0.117286000	0.517480000
1	6.885112000	-0.737913000	0.143713000
1	6.350300000	0.070006000	1.610859000
6	6.950002000	1.438155000	0.044534000
1	8.029771000	1.417175000	0.194400000
6	6.315477000	2.582268000	0.841592000
1	6.631383000	3.545471000	0.435758000
1	6.633000000	2.540176000	1.884319000
6	4.787724000	2.438118000	0.761600000
1	4.348250000	2.066316000	1.684717000
1	4.280388000	3.363679000	0.499050000
6	5.116679000	1.868306000	-1.601786000
1	4.845085000	2.908832000	-1.777126000
1	4.692756000	1.255466000	-2.397432000
6	6.648774000	1.678554000	-1.454301000
1	7.136454000	2.613654000	-1.740056000
6	7.182266000	0.584460000	-2.339215000
1	6.677356000	-0.380326000	-2.299092000
6	8.223596000	0.736006000	-3.148842000
1	8.747139000	1.685356000	-3.227870000
1	8.588588000	-0.080489000	-3.762717000
7	4.450519000	1.455798000	-0.324086000
6	4.190152000	-1.978764000	1.413389000
6	3.551227000	-2.518732000	2.575376000
6	2.608697000	-1.803784000	3.364585000
1	2.303668000	-0.798163000	3.094383000
6	2.040251000	-2.389773000	4.467280000
1	1.318981000	-1.834575000	5.056604000
6	2.378027000	-3.710843000	4.840789000
1	1.920681000	-4.155331000	5.717515000

6	3.271152000	-4.427843000	4.090655000
1	3.542592000	-5.446850000	4.342391000
6	3.867690000	-3.855716000	2.938898000
6	5.255125000	-4.121899000	1.142312000
1	5.920414000	-4.762205000	0.569156000
6	5.018055000	-2.797887000	0.694772000
1	5.493440000	-2.473157000	-0.222081000
7	4.725026000	-4.638085000	2.218562000
6	-3.583707000	-4.017990000	-2.995811000
9	-4.784071000	-3.603557000	-3.432200000
9	-2.703161000	-3.788600000	-3.975103000
9	-3.674067000	-5.353196000	-2.861281000
8	0.893427000	0.892109000	2.047917000
6	-0.268724000	1.021395000	1.481703000
6	-1.231706000	2.078623000	2.097757000
1	-1.540785000	1.566772000	3.018830000
6	-0.499365000	3.310731000	2.547462000
1	0.544076000	3.118036000	2.786021000
1	-2.045662000	4.768424000	2.508814000
6	-1.008197000	4.525541000	2.721258000
1	-0.391132000	5.340140000	3.088217000
1	-0.889648000	0.104196000	1.435136000
1	3.426496000	1.494258000	-0.440481000
6	-1.403072000	-0.184713000	-2.985706000
6	-1.966599000	0.404595000	-1.849542000
6	-3.361609000	0.467463000	-1.721276000
6	-4.233542000	-0.065722000	-2.658507000
6	-3.656106000	-0.655137000	-3.777732000
6	-2.262481000	-0.705205000	-3.941943000
6	-1.477814000	1.059805000	-0.663409000
6	-2.555656000	1.478939000	0.038723000
1	-0.327336000	-0.213599000	-3.112582000
1	-5.307266000	-0.016031000	-2.520742000
1	-4.296832000	-1.082726000	-4.540248000
1	-1.851440000	-1.174538000	-4.828546000

8	-3.718332000	1.123000000	-0.574815000
6	-2.536653000	2.340507000	1.267325000
1	-2.497897000	3.373089000	0.897034000
6	-3.796374000	2.192739000	2.096756000
6	-4.620402000	3.285247000	2.356168000
6	-4.140948000	0.946053000	2.622406000
6	-5.762397000	3.139814000	3.141374000
6	-5.280456000	0.795132000	3.402144000
6	-6.092888000	1.896208000	3.667689000
1	-4.368613000	4.256062000	1.939716000
1	-3.507072000	0.088200000	2.410205000
1	-6.393262000	3.999571000	3.339222000
1	-5.538703000	-0.180281000	3.798509000
1	-6.981830000	1.781216000	4.277970000
7	-0.185213000	1.214649000	-0.123696000
8	1.942567000	2.577034000	-0.124612000
8	0.937301000	1.816261000	-2.277765000
16	0.744262000	2.315420000	-0.921553000
6	-0.155243000	3.847140000	-1.061012000
6	0.215356000	4.932585000	-0.282026000
6	-1.220733000	3.931126000	-1.959927000
6	-0.503437000	6.119668000	-0.391432000
6	-1.935171000	5.115364000	-2.041706000
6	-1.590277000	6.224407000	-1.257545000
1	1.049185000	4.849582000	0.403351000
1	-1.475726000	3.090894000	-2.596062000
1	-0.216134000	6.972646000	0.214883000
1	-2.768501000	5.188388000	-2.733357000
6	-2.376769000	7.500812000	-1.374430000
1	-3.435290000	7.324436000	-1.167863000
1	-2.013806000	8.258744000	-0.679637000
1	-2.307855000	7.905264000	-2.387608000

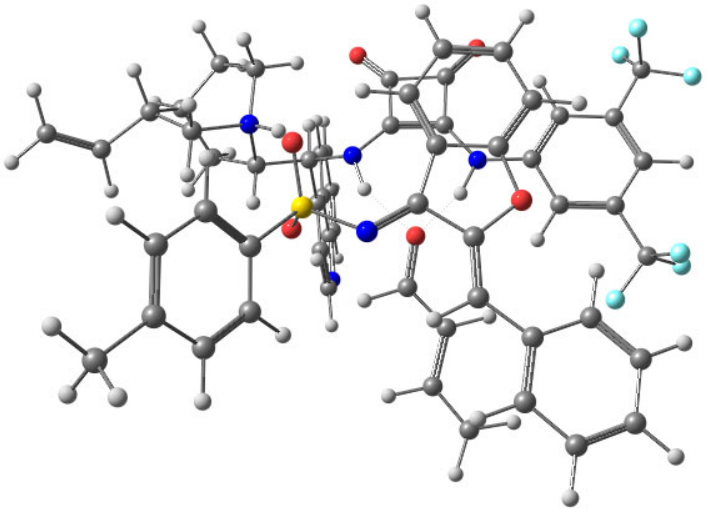

Regio-I

Sum of electronic and thermal Free Energies= -3922.496352

(2.6 kcal/mol)

6	-2.654429000	-2.112014000	0.550218000
1	-2.778643000	-3.090244000	0.089458000
6	-0.529362000	-2.395153000	-0.497629000
6	-0.664745000	-3.705117000	-1.131266000
6	0.838445000	-3.723322000	-1.307840000
6	0.867525000	-2.414010000	-0.593869000
6	3.152316000	-1.575426000	-0.239752000
6	3.836401000	-2.503069000	-1.022427000
1	3.299133000	-3.271529000	-1.566806000
6	5.221869000	-2.431842000	-1.111893000
6	5.955158000	-1.470977000	-0.435440000
1	7.034918000	-1.429510000	-0.508541000
6	5.255785000	-0.561194000	0.354405000
6	3.876445000	-0.593519000	0.451070000
1	3.347726000	0.129586000	1.062181000
6	6.042956000	0.484151000	1.086839000
9	7.092208000	-0.045478000	1.732324000
9	5.313366000	1.145970000	1.989781000
9	6.549105000	1.402151000	0.239422000
7	-1.453447000	-1.514774000	-0.052495000

1	-1.069416000	-0.661363000	0.386324000
7	1.765537000	-1.528755000	-0.136298000
1	1.365701000	-0.716567000	0.377657000
8	-1.584967000	-4.448312000	-1.404684000
8	1.624673000	-4.470707000	-1.834166000
6	-3.919234000	-1.296691000	0.269839000
1	-3.864716000	-0.347258000	0.795245000
6	-5.216781000	-2.058300000	0.617164000
1	-5.805705000	-1.484375000	1.333220000
1	-4.973203000	-3.010980000	1.094335000
6	-6.009858000	-2.327033000	-0.668350000
1	-6.889547000	-2.931451000	-0.444933000
6	-5.091466000	-3.062490000	-1.652941000
1	-5.653047000	-3.404835000	-2.522806000
1	-4.655798000	-3.942004000	-1.172017000
6	-3.982116000	-2.099490000	-2.105175000
1	-2.993955000	-2.554286000	-2.081728000
1	-4.154313000	-1.700767000	-3.104606000
6	-5.204509000	-0.084260000	-1.418666000
1	-5.101142000	0.381374000	-2.396955000
1	-5.202177000	0.697080000	-0.656667000
6	-6.448645000	-0.996478000	-1.323564000
1	-6.798860000	-1.223845000	-2.334663000
6	-7.558703000	-0.307351000	-0.583693000
1	-7.340166000	0.005811000	0.436442000
6	-8.758084000	-0.067531000	-1.100092000
1	-9.006199000	-0.357475000	-2.117665000
1	-9.534525000	0.426104000	-0.525429000
7	-3.975534000	-0.911945000	-1.185563000
6	-2.472431000	-2.260123000	2.053043000
6	-2.478425000	-3.525135000	2.710064000
6	-2.663981000	-4.771866000	2.050561000
1	-2.784471000	-4.812897000	0.974415000
6	-2.665436000	-5.943690000	2.760585000
1	-2.800571000	-6.886021000	2.242036000

6	-2.482921000	-5.938119000	4.163549000
1	-2.487702000	-6.875113000	4.708752000
6	-2.290922000	-4.756728000	4.826349000
1	-2.138107000	-4.720471000	5.899027000
6	-2.279040000	-3.525734000	4.121151000
6	-2.050336000	-1.250174000	4.196693000
1	-1.867713000	-0.355442000	4.785814000
6	-2.253826000	-1.133503000	2.804019000
1	-2.223613000	-0.149500000	2.345431000
7	-2.068524000	-2.388988000	4.842596000
6	5.900917000	-3.406864000	-2.027159000
9	5.577280000	-3.174397000	-3.312779000
9	7.234908000	-3.348115000	-1.945485000
9	5.542463000	-4.672796000	-1.770761000
8	0.299411000	0.447965000	0.931046000
6	0.177111000	1.343242000	1.842364000
6	1.188682000	1.959274000	2.534970000
1	2.221053000	1.673863000	2.339924000
6	0.934134000	2.973476000	3.526224000
1	-0.116133000	3.213240000	3.702506000
1	2.926581000	3.447051000	4.083651000
6	1.866678000	3.644735000	4.223825000
1	1.597516000	4.401596000	4.952565000
1	-0.849371000	1.678204000	2.063859000
1	-3.156421000	-0.331714000	-1.398752000
6	-0.036549000	-0.007825000	-2.816825000
6	0.665131000	0.961226000	-2.084162000
6	2.066932000	0.945306000	-2.126791000
6	2.801235000	0.039212000	-2.873959000
6	2.083683000	-0.918187000	-3.575734000
6	0.681672000	-0.942311000	-3.545631000
6	0.321246000	2.084958000	-1.213229000
6	1.606128000	2.615861000	-0.776044000
1	-1.116135000	-0.021120000	-2.821818000
1	3.884647000	0.068921000	-2.877410000

1	2.619802000	-1.663632000	-4.153248000
1	0.151520000	-1.703864000	-4.106248000
8	2.635950000	1.902892000	-1.349833000
6	1.767018000	3.612378000	0.114821000
1	0.834021000	4.063589000	0.435621000
6	2.983632000	4.193395000	0.658597000
6	2.850537000	5.339245000	1.459160000
6	4.266969000	3.661887000	0.448661000
6	3.964287000	5.950930000	2.018562000
6	5.375729000	4.269847000	1.020120000
6	5.230183000	5.416464000	1.800019000
1	1.860601000	5.744586000	1.639448000
1	4.393311000	2.772378000	-0.154794000
1	3.843197000	6.836952000	2.631551000
1	6.359861000	3.845345000	0.860303000
1	6.102688000	5.886310000	2.240315000
7	-0.761579000	2.701961000	-0.816510000
8	-2.560212000	1.450098000	-2.229635000
8	-2.637987000	1.385529000	0.231074000
16	-2.268256000	2.152033000	-0.967399000
6	-3.185227000	3.668162000	-0.963067000
6	-4.005679000	3.966125000	-2.039947000
6	-3.096457000	4.510473000	0.144364000
6	-4.754451000	5.139835000	-2.006470000
6	-3.848151000	5.672881000	0.156890000
6	-4.687183000	6.004604000	-0.915892000
1	-4.052845000	3.298502000	-2.891929000
1	-2.447726000	4.260416000	0.977007000
1	-5.397449000	5.383824000	-2.845400000
1	-3.787004000	6.339387000	1.011309000
6	-5.486492000	7.277488000	-0.877964000
1	-6.137513000	7.366202000	-1.748185000
1	-6.105981000	7.320451000	0.021094000
1	-4.824255000	8.146959000	-0.857512000

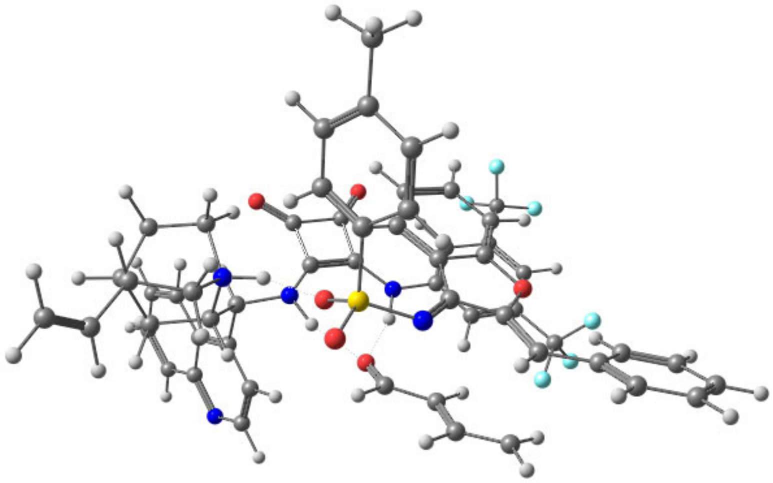

Regio-TS1

Sum of electronic and thermal Free Energies= -3922.482943

(8.4 kcal/mol)

6	3.793596000	-0.844318000	-0.466984000
1	4.287105000	-1.150093000	0.453909000
6	1.869472000	-1.721781000	0.678058000
6	2.430325000	-2.313231000	1.891781000
6	1.096315000	-3.008864000	2.105864000
6	0.643440000	-2.374228000	0.836633000
6	-1.700999000	-2.888305000	0.263458000
6	-2.010616000	-3.711874000	1.342109000
1	-1.250179000	-3.991860000	2.063120000
6	-3.324668000	-4.136663000	1.515516000
6	-4.339281000	-3.777464000	0.643689000
1	-5.359927000	-4.104799000	0.800811000
6	-4.001922000	-2.979322000	-0.448443000
6	-2.707235000	-2.544226000	-0.649323000
1	-2.463296000	-1.912970000	-1.494840000
6	-5.095932000	-2.550816000	-1.380869000
9	-5.728410000	-3.597316000	-1.931220000
9	-4.651103000	-1.784946000	-2.383677000
9	-6.037463000	-1.839171000	-0.732855000
7	2.358059000	-0.817112000	-0.191149000

1	1.725038000	-0.558478000	-0.962907000
7	-0.447591000	-2.323782000	0.052659000
1	-0.354945000	-1.728975000	-0.785371000
8	3.486266000	-2.235999000	2.480969000
8	0.630402000	-3.717950000	2.962399000
6	4.275342000	0.558094000	-0.851352000
1	3.753320000	0.868480000	-1.759726000
6	5.807227000	0.669308000	-1.012348000
1	6.051808000	1.106401000	-1.981105000
1	6.257781000	-0.324835000	-0.988641000
6	6.378066000	1.511048000	0.136739000
1	7.467820000	1.492679000	0.106923000
6	5.867468000	0.927030000	1.461126000
1	6.374341000	1.390850000	2.307993000
1	6.074505000	-0.145370000	1.501020000
6	4.357089000	1.195596000	1.564022000
1	3.791537000	0.340479000	1.931345000
1	4.135712000	2.051626000	2.201476000
6	4.348971000	2.915088000	-0.187238000
1	3.817124000	3.644265000	0.423493000
1	4.070703000	3.069014000	-1.230899000
6	5.875940000	2.969231000	0.032304000
1	6.082077000	3.464914000	0.985650000
6	6.544187000	3.752765000	-1.059816000
1	6.409803000	3.381479000	-2.074803000
6	7.268960000	4.845427000	-0.851957000
1	7.414590000	5.248683000	0.146622000
1	7.741741000	5.376324000	-1.671282000
7	3.846775000	1.553335000	0.197753000
6	4.131958000	-1.826179000	-1.582772000
6	5.110916000	-2.853139000	-1.429559000
6	5.844147000	-3.094283000	-0.234343000
1	5.658654000	-2.502292000	0.653636000
6	6.772506000	-4.100049000	-0.173286000
1	7.314441000	-4.275538000	0.748997000

6	7.025135000	-4.918912000	-1.298692000
1	7.764453000	-5.709033000	-1.232405000
6	6.331336000	-4.719562000	-2.460715000
1	6.494787000	-5.335057000	-3.338044000
6	5.358680000	-3.691209000	-2.555170000
6	3.790809000	-2.620465000	-3.830049000
1	3.266386000	-2.536396000	-4.777893000
6	3.472382000	-1.729299000	-2.781021000
1	2.690806000	-0.992585000	-2.937621000
7	4.698455000	-3.559008000	-3.739858000
6	-3.631204000	-4.946766000	2.740209000
9	-4.866325000	-5.460978000	2.722001000
9	-2.777566000	-5.967937000	2.896497000
9	-3.537078000	-4.198677000	3.855133000
8	0.251108000	-0.583517000	-1.954440000
6	-0.264237000	0.438113000	-2.485367000
6	-1.594727000	0.604577000	-2.861556000
1	-2.312110000	-0.191667000	-2.682395000
6	-2.026992000	1.791728000	-3.480833000
1	-1.275185000	2.557106000	-3.669349000
1	-4.097530000	1.314036000	-3.647734000
6	-3.338126000	2.085586000	-3.729489000
1	-3.629646000	3.006991000	-4.220853000
1	0.401926000	1.292973000	-2.684882000
1	2.815923000	1.601230000	0.195138000
6	-0.505396000	0.822282000	1.628437000
6	-1.567092000	1.178967000	0.778181000
6	-2.792567000	0.511186000	0.933839000
6	-3.023830000	-0.457379000	1.899835000
6	-1.967559000	-0.767427000	2.742226000
6	-0.721644000	-0.136249000	2.604554000
6	-1.810484000	2.192506000	-0.250224000
6	-3.181198000	2.016557000	-0.615371000
1	0.468790000	1.274413000	1.522977000
1	-3.990015000	-0.942880000	1.979231000

1	-2.105188000	-1.520031000	3.511187000
1	0.086351000	-0.406589000	3.275662000
8	-3.736348000	0.952545000	0.071334000
6	-3.899874000	2.810985000	-1.471909000
1	-3.390946000	3.725984000	-1.748904000
6	-5.348325000	2.770752000	-1.690641000
6	-5.982082000	3.934336000	-2.148254000
6	-6.122126000	1.617468000	-1.492170000
6	-7.351954000	3.956139000	-2.375711000
6	-7.490483000	1.641322000	-1.726382000
6	-8.112146000	2.808965000	-2.163651000
1	-5.388495000	4.826416000	-2.322660000
1	-5.651484000	0.700653000	-1.164937000
1	-7.826166000	4.867407000	-2.722848000
1	-8.072753000	0.739401000	-1.571731000
1	-9.181066000	2.822218000	-2.345046000
7	-1.123999000	3.210998000	-0.769779000
8	1.245677000	2.321979000	-0.316770000
8	0.865934000	4.611957000	-1.196296000
16	0.389120000	3.529040000	-0.345387000
6	0.308286000	4.140060000	1.334774000
6	1.278976000	3.781003000	2.258733000
6	-0.705397000	5.032808000	1.676358000
6	1.231190000	4.313569000	3.543121000
6	-0.741296000	5.553922000	2.960690000
6	0.224073000	5.204730000	3.912021000
1	2.057180000	3.076561000	1.989828000
1	-1.462711000	5.305240000	0.949494000
1	1.985380000	4.026347000	4.268456000
1	-1.533624000	6.243548000	3.234144000
6	0.154367000	5.773668000	5.302213000
1	0.102378000	6.864611000	5.274260000
1	-0.741682000	5.419162000	5.818509000
1	1.023273000	5.486586000	5.895356000

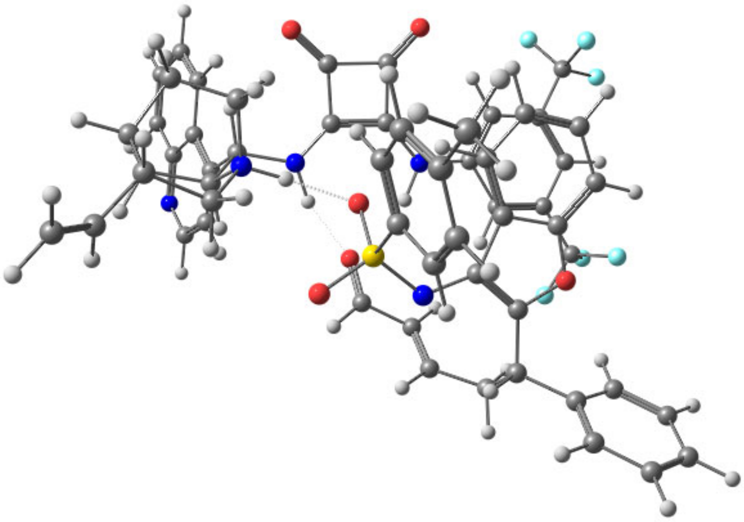

Regio-II

Sum of electronic and thermal Free Energies= -3922.529519

(-20.8 kcal/mol)

6	-4.164213000	0.464565000	-0.528570000
1	-4.627439000	0.788126000	0.401545000
6	-2.227778000	1.503350000	0.550912000
6	-2.777681000	2.041777000	1.802272000
6	-1.400093000	2.576129000	2.124633000
6	-0.948965000	1.999838000	0.836375000
6	1.395306000	2.563308000	0.313512000
6	1.577847000	3.541026000	1.290785000
1	0.779374000	3.798058000	1.976427000
6	2.812734000	4.170808000	1.401951000
6	3.874686000	3.868527000	0.564488000
1	4.829117000	4.372183000	0.660855000
6	3.674716000	2.888184000	-0.402471000
6	2.466026000	2.229750000	-0.524431000
1	2.349484000	1.437165000	-1.253412000
6	4.825321000	2.510568000	-1.286128000
9	5.353666000	3.580571000	-1.901253000
9	4.471617000	1.641683000	-2.245042000
9	5.825757000	1.943349000	-0.593981000

7	-2.751503000	0.810164000	-0.454078000
1	-2.148466000	0.539163000	-1.231933000
7	0.186692000	1.896685000	0.115270000
1	0.131352000	1.309935000	-0.715114000
8	-3.861774000	2.028584000	2.344879000
8	-0.923978000	3.192674000	3.047695000
6	-4.336885000	-1.055265000	-0.654571000
1	-3.814399000	-1.398948000	-1.548797000
6	-5.813877000	-1.505765000	-0.653694000
1	-6.026839000	-2.095651000	-1.545953000
1	-6.466656000	-0.630094000	-0.685491000
6	-6.101595000	-2.301228000	0.625788000
1	-7.164535000	-2.536627000	0.687044000
6	-5.669173000	-1.446851000	1.824275000
1	-6.006799000	-1.892337000	2.760652000
1	-6.112955000	-0.449946000	1.753215000
6	-4.135271000	-1.357545000	1.827996000
1	-3.768738000	-0.360485000	2.060815000
1	-3.685735000	-2.057246000	2.531842000
6	-3.812862000	-3.240924000	0.303641000
1	-3.094389000	-3.737121000	0.955533000
1	-3.549188000	-3.476083000	-0.726569000
6	-5.273858000	-3.606452000	0.647042000
1	-5.313242000	-4.010278000	1.663164000
6	-5.793508000	-4.651749000	-0.296825000
1	-5.796958000	-4.390056000	-1.354375000
6	-6.234733000	-5.844962000	0.082834000
1	-6.236012000	-6.143868000	1.127643000
1	-6.609053000	-6.566062000	-0.636004000
7	-3.625687000	-1.758943000	0.473032000
6	-4.820212000	1.180790000	-1.701936000
6	-5.687915000	2.297877000	-1.505289000
6	-6.016537000	2.844545000	-0.234147000
1	-5.580151000	2.441058000	0.671914000
6	-6.866977000	3.914714000	-0.132968000

1	-7.099603000	4.323582000	0.843697000
6	-7.437288000	4.497360000	-1.288187000
1	-8.110052000	5.341505000	-1.187963000
6	-7.132346000	4.002354000	-2.526902000
1	-7.544706000	4.431785000	-3.432828000
6	-6.250069000	2.901227000	-2.667287000
6	-5.157402000	1.464473000	-4.070225000
1	-4.944403000	1.141929000	-5.085762000
6	-4.555519000	0.783499000	-2.987013000
1	-3.887877000	-0.042888000	-3.204297000
7	-5.978804000	2.472514000	-3.932907000
6	3.001430000	5.152179000	2.520145000
9	4.046883000	5.964131000	2.315712000
9	1.924661000	5.929756000	2.699964000
9	3.220591000	4.528273000	3.691105000
8	-0.701811000	0.579951000	-2.466060000
6	-0.047584000	-0.274026000	-3.045640000
6	1.366120000	-0.130725000	-3.354376000
1	1.831508000	0.840737000	-3.217613000
6	2.076020000	-1.242779000	-3.571901000
1	1.538850000	-2.183980000	-3.676917000
1	4.023494000	-0.356099000	-3.487421000
6	3.558240000	-1.343237000	-3.490015000
1	3.951833000	-1.914323000	-4.336253000
1	-0.513764000	-1.234890000	-3.318130000
1	-2.609373000	-1.533015000	0.403882000
6	1.542031000	-0.344683000	1.975212000
6	2.366874000	-0.731276000	0.904512000
6	3.689144000	-0.265369000	0.892612000
6	4.239073000	0.541656000	1.881159000
6	3.405031000	0.904365000	2.926298000
6	2.070002000	0.465341000	2.967559000
6	2.233643000	-1.522949000	-0.318792000
6	3.451303000	-1.443777000	-0.936053000
1	0.500915000	-0.633325000	2.006798000

1	5.266903000	0.878075000	1.815683000
1	3.783281000	1.543808000	3.716066000
1	1.436278000	0.781371000	3.789003000
8	4.340887000	-0.676106000	-0.219457000
6	3.927214000	-2.122112000	-2.187539000
1	3.377714000	-3.068178000	-2.229696000
6	5.409194000	-2.443209000	-2.157594000
6	5.834228000	-3.770034000	-2.103880000
6	6.371307000	-1.430254000	-2.207973000
6	7.191219000	-4.085041000	-2.102320000
6	7.725867000	-1.742093000	-2.206764000
6	8.140862000	-3.071174000	-2.155458000
1	5.096047000	-4.565226000	-2.061703000
1	6.059418000	-0.391848000	-2.231981000
1	7.503253000	-5.122949000	-2.060336000
1	8.460551000	-0.944884000	-2.242615000
1	9.198133000	-3.312523000	-2.154892000
7	1.198805000	-2.265092000	-0.881505000
8	-0.878729000	-1.230459000	0.244953000
8	-1.017677000	-3.332431000	-1.022266000
16	-0.167745000	-2.480252000	-0.176237000
6	0.097284000	-3.376867000	1.359571000
6	-0.645237000	-3.074976000	2.497967000
6	1.027434000	-4.408535000	1.377639000
6	-0.456270000	-3.818948000	3.654810000
6	1.207261000	-5.146473000	2.543099000
6	0.468827000	-4.867142000	3.693733000
1	-1.341079000	-2.242934000	2.486658000
1	1.614923000	-4.625483000	0.491624000
1	-1.025781000	-3.577129000	4.547309000
1	1.938564000	-5.948085000	2.559333000
6	0.659384000	-5.661666000	4.957022000
1	-0.255589000	-6.199570000	5.219448000
1	1.462476000	-6.391584000	4.850490000
1	0.903128000	-5.006301000	5.796671000

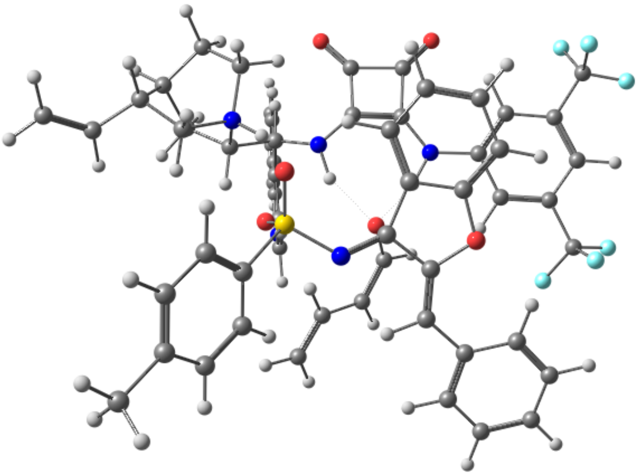

Z-I

Sum of electronic and thermal Free Energies= -3922.496569

(-0.1 kcal/mol)

6	-2.405635000	-2.240711000	0.579852000
1	-2.427230000	-3.277699000	0.249971000
6	-0.242861000	-2.489705000	-0.412760000
6	-0.286315000	-3.865239000	-0.907948000
6	1.217802000	-3.803194000	-1.073654000
6	1.153632000	-2.424718000	-0.507671000
6	3.361269000	-1.383154000	-0.241969000
6	4.110014000	-2.305797000	-0.972826000
1	3.630455000	-3.154741000	-1.447110000
6	5.481550000	-2.125839000	-1.100698000
6	6.143195000	-1.058090000	-0.513890000
1	7.213117000	-0.931868000	-0.620864000
6	5.384742000	-0.158999000	0.230417000
6	4.014733000	-0.302814000	0.367562000
1	3.442867000	0.425179000	0.933789000
6	6.069799000	1.027285000	0.841816000
9	7.307490000	0.730895000	1.262735000
9	5.401664000	1.522650000	1.887668000
9	6.204409000	2.032099000	-0.046935000
7	-1.221689000	-1.630509000	-0.046638000
1	-0.893693000	-0.740976000	0.355197000

7	1.976888000	-1.436021000	-0.133971000
1	1.473928000	-0.613309000	0.308626000
8	-1.151411000	-4.695370000	-1.097661000
8	2.060498000	-4.552946000	-1.499156000
6	-3.721217000	-1.582458000	0.144033000
1	-3.810711000	-0.604178000	0.606990000
6	-4.959081000	-2.455464000	0.443812000
1	-5.654124000	-1.910182000	1.083046000
1	-4.657945000	-3.353411000	0.989845000
6	-5.623012000	-2.867712000	-0.876464000
1	-6.457121000	-3.542148000	-0.680341000
6	-4.568536000	-3.560283000	-1.748949000
1	-5.029417000	-3.998136000	-2.635125000
1	-4.088899000	-4.367512000	-1.190180000
6	-3.520881000	-2.520395000	-2.175668000
1	-2.501793000	-2.876145000	-2.045848000
1	-3.645187000	-2.196027000	-3.208476000
6	-4.976969000	-0.599929000	-1.707055000
1	-4.843153000	-0.184215000	-2.704335000
1	-5.106673000	0.218460000	-0.997771000
6	-6.133647000	-1.623432000	-1.640886000
1	-6.385782000	-1.940101000	-2.657052000
6	-7.353665000	-1.005661000	-1.020899000
1	-7.233820000	-0.602772000	-0.015951000
6	-8.535367000	-0.930104000	-1.621376000
1	-8.686468000	-1.313339000	-2.627072000
1	-9.393476000	-0.483854000	-1.130206000
7	-3.698399000	-1.289483000	-1.333797000
6	-2.291644000	-2.162723000	2.095324000
6	-2.272996000	-3.317409000	2.931845000
6	-2.358712000	-4.655198000	2.456941000
1	-2.419347000	-4.858018000	1.394008000
6	-2.340188000	-5.710197000	3.331260000
1	-2.399268000	-6.723999000	2.951981000
6	-2.236166000	-5.490199000	4.724662000

1	-2.224255000	-6.336676000	5.401965000
6	-2.141751000	-4.215378000	5.213160000
1	-2.051801000	-4.014408000	6.274720000
6	-2.152320000	-3.101250000	4.335161000
6	-2.039295000	-0.829042000	4.084195000
1	-1.938456000	0.153701000	4.538076000
6	-2.166921000	-0.928362000	2.680529000
1	-2.150417000	-0.017875000	2.087149000
7	-2.040309000	-1.860989000	4.890296000
6	6.229427000	-3.098831000	-1.963255000
9	5.935647000	-2.923803000	-3.265301000
9	7.556663000	-2.975250000	-1.846506000
9	5.920630000	-4.370166000	-1.673971000
1	1.742516000	0.335467000	2.500193000
6	0.826423000	0.807374000	2.106514000
6	0.237801000	1.740633000	2.914212000
1	0.705007000	1.950176000	3.872778000
6	-0.986546000	2.423068000	2.567469000
1	-1.414219000	2.143612000	1.606243000
1	-1.236136000	3.653555000	4.284428000
6	-1.624400000	3.338810000	3.318480000
1	-2.559412000	3.785909000	2.996578000
1	-2.917963000	-0.648589000	-1.505334000
6	0.255755000	0.055077000	-2.784606000
6	0.795576000	1.095854000	-2.016748000
6	2.187588000	1.246900000	-1.969536000
6	3.065028000	0.459154000	-2.694969000
6	2.507068000	-0.563932000	-3.448270000
6	1.121283000	-0.773349000	-3.482176000
6	0.266447000	2.159052000	-1.163257000
6	1.456543000	2.789541000	-0.577039000
1	-0.812365000	-0.094405000	-2.845117000
1	4.135612000	0.618764000	-2.636102000
1	3.161504000	-1.221999000	-4.009690000
1	0.720963000	-1.588603000	-4.073499000

8	2.593079000	2.234511000	-1.125964000
6	1.446343000	3.686412000	0.419370000
1	0.450360000	3.976681000	0.740948000
6	2.561682000	4.285305000	1.138896000
6	2.258483000	5.080094000	2.256160000
6	3.906819000	4.101632000	0.785065000
6	3.268043000	5.672581000	3.000014000
6	4.914086000	4.696065000	1.533830000
6	4.600617000	5.482521000	2.639289000
1	1.219008000	5.216463000	2.539092000
1	4.165003000	3.494319000	-0.071229000
1	3.018330000	6.280266000	3.862460000
1	5.949741000	4.539564000	1.253452000
1	5.391910000	5.943556000	3.219870000
7	-0.896122000	2.661598000	-0.885709000
8	-2.423706000	1.341517000	-2.557896000
8	-2.734746000	1.144440000	-0.122140000
16	-2.330485000	1.998045000	-1.248405000
6	-3.362548000	3.436076000	-1.268609000
6	-4.246255000	3.612890000	-2.322323000
6	-3.322042000	4.316502000	-0.188374000
6	-5.109771000	4.704838000	-2.293341000
6	-4.188782000	5.395822000	-0.180514000
6	-5.094830000	5.604913000	-1.229200000
1	-4.252831000	2.918558000	-3.154154000
1	-2.616330000	4.164528000	0.621745000
1	-5.802575000	4.856940000	-3.114050000
1	-4.165172000	6.092954000	0.651109000
6	-6.019399000	6.789683000	-1.196998000
1	-6.714317000	6.776210000	-2.036929000
1	-6.598595000	6.805090000	-0.270698000
1	-5.450552000	7.722210000	-1.239467000
8	0.439518000	0.420751000	0.933906000

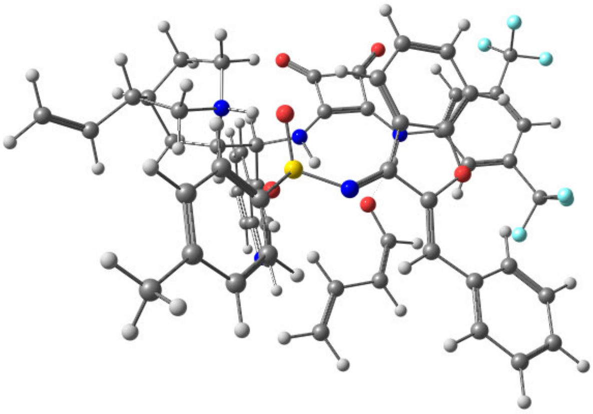

Z-TS1

Sum of electronic and thermal Free Energies= -3922.482323

(8.8 kcal/mol)

6	-2.315587000	-2.259423000	0.623682000
1	-2.307243000	-3.291126000	0.277979000
6	-0.164444000	-2.437330000	-0.404404000
6	-0.196084000	-3.812788000	-0.910791000
6	1.303946000	-3.730025000	-1.095839000
6	1.228461000	-2.363132000	-0.506498000
6	3.450734000	-1.345816000	-0.219576000
6	4.184170000	-2.232041000	-1.007137000
1	3.694632000	-3.048889000	-1.524489000
6	5.556255000	-2.056440000	-1.134833000
6	6.232419000	-1.025363000	-0.499429000
1	7.301837000	-0.900048000	-0.611854000
6	5.487513000	-0.162422000	0.297633000
6	4.118608000	-0.308483000	0.440285000
1	3.560068000	0.388310000	1.053701000
6	6.172003000	0.994750000	0.962550000
9	7.428519000	0.703385000	1.319099000
9	5.528230000	1.403742000	2.064293000
9	6.243673000	2.062335000	0.144293000
7	-1.160280000	-1.608351000	-0.018635000
1	-0.893340000	-0.689448000	0.343035000
7	2.064235000	-1.392354000	-0.102817000

1	1.607024000	-0.604904000	0.387940000
8	-1.047813000	-4.657731000	-1.082870000
8	2.148636000	-4.464526000	-1.540390000
6	-3.664746000	-1.648971000	0.225878000
1	-3.776942000	-0.675333000	0.692999000
6	-4.854779000	-2.574809000	0.565003000
1	-5.540886000	-2.067061000	1.243621000
1	-4.498036000	-3.470360000	1.080896000
6	-5.559351000	-2.992970000	-0.731744000
1	-6.364966000	-3.693629000	-0.509584000
6	-4.519959000	-3.647551000	-1.651332000
1	-5.002056000	-4.086237000	-2.525800000
1	-4.001845000	-4.449898000	-1.120189000
6	-3.512491000	-2.577383000	-2.100541000
1	-2.480407000	-2.904805000	-2.003061000
1	-3.673484000	-2.252591000	-3.128218000
6	-5.008858000	-0.707146000	-1.580257000
1	-4.920483000	-0.301815000	-2.586477000
1	-5.130038000	0.120299000	-0.878917000
6	-6.137372000	-1.756357000	-1.459391000
1	-6.436963000	-2.072919000	-2.462737000
6	-7.336649000	-1.172342000	-0.769631000
1	-7.169323000	-0.758417000	0.223985000
6	-8.554407000	-1.137282000	-1.297427000
1	-8.754193000	-1.531535000	-2.290290000
1	-9.394682000	-0.714368000	-0.757170000
7	-3.698913000	-1.356008000	-1.250148000
6	-2.168387000	-2.205582000	2.137445000
6	-2.085564000	-3.373880000	2.950280000
6	-2.144241000	-4.705690000	2.453494000
1	-2.240066000	-4.892476000	1.390216000
6	-2.061312000	-5.774386000	3.306713000
1	-2.101765000	-6.782667000	2.910724000
6	-1.916431000	-5.576253000	4.700105000
1	-1.853931000	-6.433930000	5.360237000

6	-1.849115000	-4.308357000	5.209548000
1	-1.731638000	-4.123321000	6.271297000
6	-1.927362000	-3.179583000	4.353459000
6	-1.907790000	-0.901079000	4.143644000
1	-1.829990000	0.073937000	4.617473000
6	-2.075178000	-0.977919000	2.743526000
1	-2.114346000	-0.059200000	2.164652000
7	-1.841748000	-1.947218000	4.928676000
6	6.288817000	-2.993490000	-2.049756000
9	5.973773000	-2.765629000	-3.337739000
9	7.617643000	-2.875550000	-1.948546000
9	5.982953000	-4.275147000	-1.806226000
1	2.083627000	1.255685000	2.199132000
6	1.030516000	1.391003000	1.899926000
6	0.426330000	2.563431000	2.377628000
1	0.997169000	3.140286000	3.098938000
6	-1.002536000	2.792948000	2.303972000
1	-1.562361000	2.130175000	1.648961000
1	-1.101954000	4.447953000	3.630289000
6	-1.637355000	3.766411000	2.973623000
1	-2.712103000	3.895848000	2.900205000
1	-2.956224000	-0.679444000	-1.465622000
6	0.285288000	0.185268000	-2.961122000
6	0.769213000	1.159501000	-2.073459000
6	2.156108000	1.270727000	-1.893621000
6	3.084847000	0.515443000	-2.594093000
6	2.582591000	-0.445878000	-3.457502000
6	1.198992000	-0.615969000	-3.628732000
6	0.192782000	2.193627000	-1.218837000
6	1.288362000	2.787235000	-0.566065000
1	-0.776993000	0.075491000	-3.129337000
1	4.148770000	0.657793000	-2.439233000
1	3.272938000	-1.079345000	-4.004158000
1	0.840194000	-1.377010000	-4.312456000
8	2.478026000	2.208425000	-0.972022000

6	1.219760000	3.681662000	0.496490000
1	0.261677000	4.184853000	0.563079000
6	2.353420000	4.393188000	1.102421000
6	2.093894000	5.616673000	1.736456000
6	3.664100000	3.896091000	1.110140000
6	3.115220000	6.332698000	2.345994000
6	4.683417000	4.613548000	1.724152000
6	4.415185000	5.832622000	2.341422000
1	1.078874000	6.001999000	1.744897000
1	3.888113000	2.952882000	0.630576000
1	2.897486000	7.280212000	2.826208000
1	5.692293000	4.216460000	1.719728000
1	5.214570000	6.388664000	2.818287000
7	-1.044173000	2.688462000	-1.032578000
8	-2.545766000	1.071457000	-2.425926000
8	-2.670737000	1.054549000	0.009873000
16	-2.382919000	1.875184000	-1.192094000
6	-3.586465000	3.181329000	-1.250906000
6	-4.605270000	3.109590000	-2.189182000
6	-3.538013000	4.209420000	-0.311891000
6	-5.594537000	4.089986000	-2.186957000
6	-4.530786000	5.175834000	-0.325854000
6	-5.573226000	5.130963000	-1.260533000
1	-4.617145000	2.308798000	-2.919038000
1	-2.727919000	4.254922000	0.407883000
1	-6.391246000	4.043372000	-2.922066000
1	-4.498680000	5.984049000	0.398377000
6	-6.629653000	6.201388000	-1.265189000
1	-7.444869000	5.953413000	-1.945755000
1	-7.046591000	6.344103000	-0.265637000
1	-6.205533000	7.158755000	-1.580135000
8	0.523738000	0.567134000	1.101925000

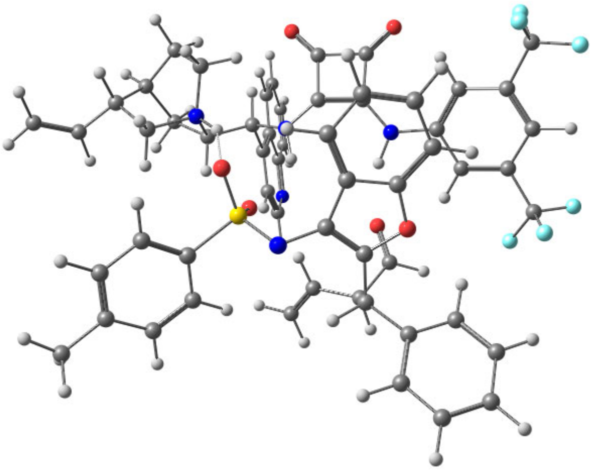

Z-II

Sum of electronic and thermal Free Energies= -3922.515439

(-12.0 kcal/mol)

6	-2.636618000	-2.064711000	0.470645000
1	-2.744042000	-3.088467000	0.118018000
6	-0.425235000	-2.500114000	-0.352533000
6	-0.539675000	-3.896236000	-0.797202000
6	0.978932000	-3.963045000	-0.807705000
6	0.968350000	-2.551672000	-0.336198000
6	3.264849000	-1.720322000	-0.040636000
6	3.897479000	-2.561198000	-0.952886000
1	3.326486000	-3.252479000	-1.559755000
6	5.279416000	-2.496903000	-1.092496000
6	6.052555000	-1.601485000	-0.369307000
1	7.125401000	-1.547324000	-0.505246000
6	5.401831000	-0.775858000	0.542755000
6	4.032408000	-0.834933000	0.723122000
1	3.543554000	-0.197762000	1.447456000
6	6.194450000	0.260455000	1.282045000
9	7.387648000	-0.198372000	1.678080000
9	5.557690000	0.712051000	2.372135000
9	6.434697000	1.335631000	0.507927000
7	-1.373497000	-1.579961000	-0.097891000
1	-1.108653000	-0.623535000	0.133361000

7	1.875654000	-1.651233000	0.088593000
1	1.522013000	-0.859020000	0.620878000
8	-1.452072000	-4.658345000	-1.021461000
8	1.800775000	-4.799100000	-1.076269000
6	-3.852942000	-1.264088000	-0.008439000
1	-3.828363000	-0.270354000	0.432668000
6	-5.183872000	-1.990735000	0.291427000
1	-5.846731000	-1.337726000	0.860261000
1	-4.994970000	-2.871070000	0.910487000
6	-5.835498000	-2.429025000	-1.027080000
1	-6.719928000	-3.033720000	-0.823926000
6	-4.807571000	-3.234957000	-1.832820000
1	-5.275622000	-3.688195000	-2.707478000
1	-4.400010000	-4.043993000	-1.221328000
6	-3.684080000	-2.287962000	-2.286291000
1	-2.691108000	-2.706936000	-2.139166000
1	-3.779825000	-1.995259000	-3.331396000
6	-5.003592000	-0.255020000	-1.909578000
1	-4.812062000	0.141585000	-2.904929000
1	-5.098413000	0.582148000	-1.215597000
6	-6.229342000	-1.191762000	-1.865547000
1	-6.454391000	-1.532341000	-2.880476000
6	-7.431434000	-0.466120000	-1.334097000
1	-7.341262000	-0.050105000	-0.331327000
6	-8.563590000	-0.308307000	-2.009587000
1	-8.683815000	-0.701710000	-3.015527000
1	-9.410400000	0.217716000	-1.581786000
7	-3.780508000	-1.018209000	-1.492664000
6	-2.580136000	-2.042459000	1.993555000
6	-2.703188000	-3.223056000	2.784616000
6	-2.907216000	-4.528675000	2.256823000
1	-2.959380000	-4.688241000	1.186360000
6	-3.019949000	-5.610223000	3.090329000
1	-3.168531000	-6.598587000	2.670612000
6	-2.936885000	-5.452481000	4.493736000

1	-3.028253000	-6.319923000	5.137485000
6	-2.734297000	-4.211950000	5.033495000
1	-2.659330000	-4.058296000	6.104043000
6	-2.609384000	-3.071501000	4.198922000
6	-2.275623000	-0.814164000	4.041423000
1	-2.095812000	0.134407000	4.540669000
6	-2.363874000	-0.847047000	2.632152000
1	-2.247922000	0.077735000	2.073790000
7	-2.397719000	-1.870015000	4.805856000
6	5.912578000	-3.400923000	-2.109404000
9	5.495465000	-3.109661000	-3.354290000
9	7.247292000	-3.312469000	-2.114811000
9	5.600653000	-4.687299000	-1.897805000
1	2.977554000	1.734112000	2.687582000
6	1.936217000	1.648102000	2.330425000
6	1.153672000	2.927393000	2.204224000
1	1.471382000	3.609821000	2.997710000
6	-0.320524000	2.662236000	2.285053000
1	-0.738701000	2.026586000	1.509592000
1	-0.723940000	3.810322000	4.016839000
6	-1.108498000	3.170685000	3.226434000
1	-2.174443000	2.962605000	3.237081000
1	-2.973101000	-0.408347000	-1.703758000
6	0.607511000	0.576926000	-3.172203000
6	1.067627000	1.314579000	-2.074154000
6	2.398306000	1.166422000	-1.668781000
6	3.313400000	0.350803000	-2.316430000
6	2.836354000	-0.380182000	-3.396423000
6	1.499637000	-0.272110000	-3.813220000
6	0.498831000	2.314408000	-1.177751000
6	1.500903000	2.647311000	-0.318082000
1	-0.418299000	0.676241000	-3.505109000
1	4.344122000	0.286500000	-1.983934000
1	3.510740000	-1.044665000	-3.925186000
1	1.164555000	-0.854445000	-4.664474000

8	2.661338000	1.954555000	-0.593353000
6	1.517962000	3.613290000	0.821555000
1	0.686620000	4.296199000	0.630631000
6	2.787228000	4.443634000	0.917344000
6	2.683338000	5.835378000	0.884678000
6	4.054659000	3.873916000	1.079974000
6	3.811336000	6.641355000	1.011482000
6	5.182662000	4.677180000	1.206588000
6	5.065677000	6.064146000	1.174777000
1	1.707710000	6.293619000	0.755408000
1	4.175094000	2.797792000	1.085547000
1	3.706862000	7.720380000	0.980320000
1	6.155870000	4.213351000	1.326036000
1	5.946700000	6.688639000	1.272642000
7	-0.748595000	2.944750000	-1.216490000
8	-2.363495000	1.257163000	-2.365579000
8	-2.116197000	1.174150000	0.048673000
16	-2.029352000	2.062321000	-1.154700000
6	-3.310139000	3.287616000	-0.993032000
6	-4.338196000	3.337128000	-1.921128000
6	-3.283984000	4.151059000	0.100202000
6	-5.362283000	4.267103000	-1.749339000
6	-4.309223000	5.069701000	0.256160000
6	-5.363791000	5.140643000	-0.663850000
1	-4.331132000	2.663928000	-2.770127000
1	-2.465932000	4.105290000	0.811587000
1	-6.167311000	4.313278000	-2.475582000
1	-4.295591000	5.748526000	1.103512000
6	-6.462722000	6.150020000	-0.473491000
1	-7.220349000	6.065746000	-1.253226000
1	-6.951790000	6.015581000	0.494622000
1	-6.062605000	7.166939000	-0.496068000
8	1.488375000	0.563638000	2.033859000

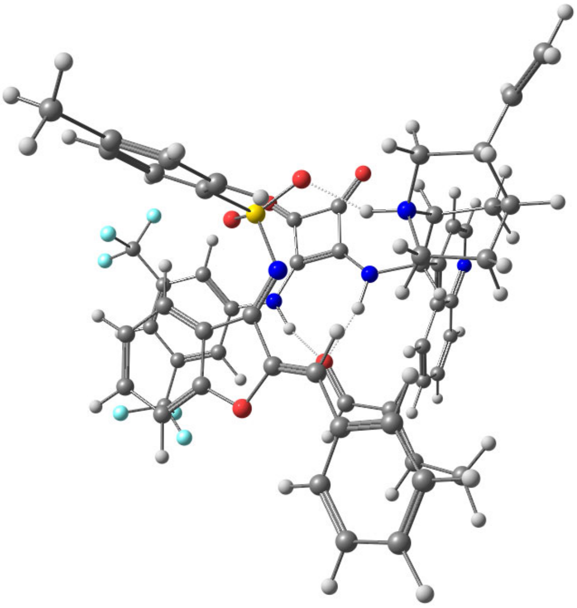

DiastA-I

Sum of electronic and thermal Free Energies= -3922.499840

(-2.2 kcal/mol)

6	3.181547000	-1.273550000	-0.895193000
1	3.613445000	-1.221361000	0.110019000
6	0.863648000	-1.279084000	-1.705708000
6	0.894360000	-1.282567000	-3.175384000
6	-0.605125000	-1.539624000	-3.145797000
6	-0.514228000	-1.489151000	-1.666809000
6	-2.709121000	-1.684305000	-0.600452000
6	-3.485464000	-1.255668000	-1.678240000
1	-3.013944000	-0.864780000	-2.570581000
6	-4.869188000	-1.322186000	-1.585325000
6	-5.510691000	-1.788799000	-0.446884000
1	-6.591449000	-1.816393000	-0.383350000
6	-4.719825000	-2.193952000	0.623479000
6	-3.337940000	-2.147569000	0.561551000
1	-2.735937000	-2.461295000	1.407747000
6	-5.389271000	-2.710405000	1.862115000
9	-6.532256000	-2.053992000	2.122430000
9	-4.614878000	-2.594650000	2.949315000
9	-5.714324000	-4.008920000	1.755507000

7	1.751276000	-1.046520000	-0.722597000
1	1.358777000	-1.065836000	0.235867000
7	-1.321915000	-1.613044000	-0.598542000
1	-0.860182000	-1.637692000	0.324598000
8	1.730856000	-1.123821000	-4.034981000
8	-1.464136000	-1.692939000	-3.976634000
6	3.818182000	-0.164527000	-1.744834000
1	3.454039000	-0.249489000	-2.769685000
6	5.360594000	-0.138414000	-1.670227000
1	5.788249000	-0.208520000	-2.671419000
1	5.715299000	-1.006441000	-1.109541000
6	5.817431000	1.143369000	-0.961880000
1	6.898821000	1.127111000	-0.822429000
6	5.105757000	1.225632000	0.395900000
1	5.509462000	2.044866000	0.992370000
1	5.265453000	0.299471000	0.953537000
6	3.605215000	1.457193000	0.151679000
1	2.961822000	0.816733000	0.753215000
1	3.307009000	2.492296000	0.321856000
6	3.922114000	2.248446000	-2.145529000
1	3.362170000	3.164436000	-1.965173000
1	3.765730000	1.946551000	-3.182002000
6	5.419035000	2.386624000	-1.790052000
1	5.555328000	3.267194000	-1.155378000
6	6.248994000	2.564658000	-3.028705000
1	6.165329000	1.784293000	-3.784113000
6	7.054503000	3.598544000	-3.240584000
1	7.152482000	4.400138000	-2.513121000
1	7.644011000	3.681534000	-4.147380000
7	3.309231000	1.179765000	-1.290552000
6	3.475216000	-2.667309000	-1.445207000
6	3.170795000	-3.812777000	-0.642438000
6	2.616416000	-3.735706000	0.661499000
1	2.373578000	-2.778754000	1.103393000
6	2.360090000	-4.871854000	1.384251000

1	1.940404000	-4.785471000	2.380850000
6	2.637528000	-6.147359000	0.841712000
1	2.427165000	-7.037539000	1.423752000
6	3.167665000	-6.255553000	-0.416403000
1	3.390057000	-7.218192000	-0.862916000
6	3.445441000	-5.097343000	-1.185377000
6	4.231247000	-4.209078000	-3.136615000
1	4.646554000	-4.365885000	-4.128639000
6	4.001757000	-2.883509000	-2.690020000
1	4.241706000	-2.074786000	-3.367282000
7	3.974775000	-5.277677000	-2.431125000
6	-5.675763000	-0.732379000	-2.702143000
9	-6.943825000	-1.162632000	-2.705317000
9	-5.727745000	0.612813000	-2.600235000
9	-5.160566000	-0.999614000	-3.907203000
8	0.258278000	-1.297483000	1.579265000
6	0.759526000	-1.508830000	2.743107000
6	2.039591000	-1.223928000	3.135600000
1	2.719360000	-0.767995000	2.413098000
6	2.504325000	-1.424872000	4.484593000
1	1.779776000	-1.864815000	5.171921000
1	4.476295000	-0.649882000	4.333600000
6	3.713596000	-1.096009000	4.967725000
1	3.974217000	-1.260656000	6.007341000
1	0.090650000	-1.945869000	3.503112000
1	2.289704000	1.222483000	-1.440215000
6	-3.324540000	1.332360000	0.341167000
6	-2.196143000	1.240509000	1.175783000
6	-2.384227000	0.910450000	2.524659000
6	-3.623786000	0.627327000	3.076207000
6	-4.719626000	0.736164000	2.233161000
6	-4.574127000	1.098634000	0.885863000
6	-0.762098000	1.481858000	1.036082000
6	-0.243074000	1.388765000	2.408858000
1	-3.229183000	1.569414000	-0.707380000

1	-3.720875000	0.360897000	4.121297000
1	-5.710329000	0.539889000	2.627624000
1	-5.451990000	1.176346000	0.255998000
8	-1.237887000	0.952042000	3.250582000
6	0.971564000	1.773129000	2.829046000
1	1.615223000	2.135241000	2.033414000
6	1.516224000	1.837458000	4.179683000
6	2.798195000	2.384095000	4.334140000
6	0.844216000	1.372801000	5.322120000
6	3.386407000	2.489254000	5.588852000
6	1.435360000	1.478403000	6.572536000
6	2.702775000	2.042105000	6.714271000
1	3.334020000	2.733331000	3.456384000
1	-0.133342000	0.919633000	5.229799000
1	4.377828000	2.917385000	5.686275000
1	0.905134000	1.114001000	7.445483000
1	3.157466000	2.119765000	7.695485000
7	0.056934000	1.778224000	0.068554000
8	0.836737000	2.062818000	-2.233802000
8	-1.476135000	1.180177000	-1.966491000
16	-0.422985000	2.063672000	-1.481336000
6	-1.060633000	3.720753000	-1.438633000
6	-0.289035000	4.729491000	-0.862443000
6	-2.299123000	3.987197000	-2.004701000
6	-0.784117000	6.022220000	-0.850248000
6	-2.776105000	5.294783000	-1.985916000
6	-2.032821000	6.323739000	-1.410390000
1	0.677311000	4.504521000	-0.424044000
1	-2.878964000	3.191922000	-2.458599000
1	-0.196371000	6.815890000	-0.400257000
1	-3.742428000	5.512839000	-2.427721000
6	-2.549681000	7.734750000	-1.377173000
1	-1.832070000	8.421821000	-1.831840000
1	-3.496629000	7.824971000	-1.909669000
1	-2.705278000	8.062864000	-0.346073000

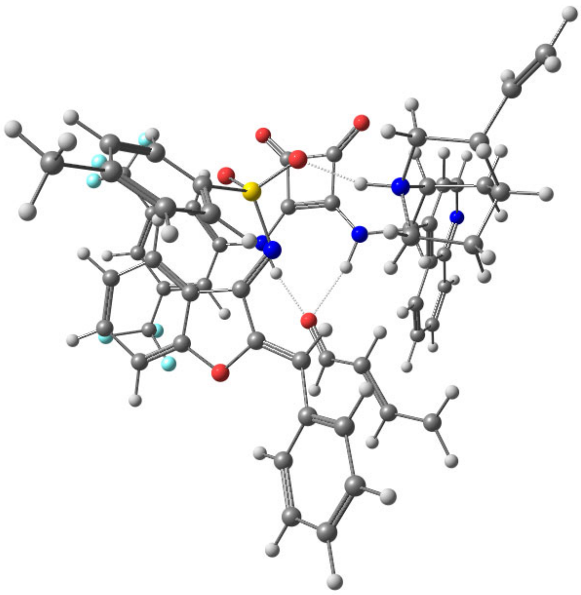

DiastA-TS1

Sum of electronic and thermal Free Energies= -3922.489179

(4.5 kcal/mol)

6	3.370554000	-1.276332000	-0.239256000
1	3.715281000	-0.715404000	0.633183000
6	1.114977000	-1.795819000	-1.101041000
6	1.251166000	-2.337181000	-2.461813000
6	-0.232599000	-2.657672000	-2.421118000
6	-0.253641000	-2.062157000	-1.064245000
6	-2.515369000	-2.010635000	-0.101012000
6	-3.240275000	-2.156302000	-1.282247000
1	-2.735912000	-2.152312000	-2.240117000
6	-4.621413000	-2.295615000	-1.212902000
6	-5.306137000	-2.276502000	-0.007331000
1	-6.384558000	-2.370614000	0.026731000
6	-4.565086000	-2.110165000	1.159074000
6	-3.189059000	-1.977301000	1.126600000
1	-2.625228000	-1.839156000	2.043130000
6	-5.282907000	-2.109272000	2.475796000
9	-6.455150000	-1.458000000	2.407656000
9	-4.568376000	-1.534089000	3.451798000
9	-5.568207000	-3.355715000	2.890308000

7	1.916797000	-1.159983000	-0.225973000
1	1.467705000	-0.852760000	0.638997000
7	-1.135218000	-1.836590000	-0.075005000
1	-0.749936000	-1.490783000	0.805309000
8	2.136577000	-2.460482000	-3.278446000
8	-1.016405000	-3.162177000	-3.183079000
6	3.981281000	-0.626732000	-1.488242000
1	3.692027000	-1.201174000	-2.367169000
6	5.514247000	-0.453721000	-1.395000000
1	6.001821000	-0.984211000	-2.213899000
1	5.881683000	-0.896176000	-0.465896000
6	5.863592000	1.039764000	-1.414573000
1	6.937689000	1.172518000	-1.280053000
6	5.095502000	1.727746000	-0.277631000
1	5.417799000	2.763963000	-0.167361000
1	5.298970000	1.213393000	0.664771000
6	3.592266000	1.684206000	-0.600753000
1	2.977235000	1.352696000	0.232697000
1	3.204986000	2.645014000	-0.940882000
6	3.948048000	1.298009000	-2.991750000
1	3.332057000	2.152607000	-3.264782000
1	3.833875000	0.530850000	-3.759236000
6	5.425937000	1.679653000	-2.751976000
1	5.501046000	2.765510000	-2.642033000
6	6.282413000	1.261821000	-3.912766000
1	6.258822000	0.205189000	-4.176506000
6	7.042340000	2.094422000	-4.614013000
1	7.079924000	3.156589000	-4.387088000
1	7.652285000	1.745554000	-5.440491000
7	3.371049000	0.728115000	-1.730267000
6	3.819610000	-2.725563000	-0.066052000
6	3.690718000	-3.350368000	1.215284000
6	3.188293000	-2.692962000	2.367986000
1	2.864429000	-1.662188000	2.315635000
6	3.092795000	-3.348237000	3.568028000

1	2.704532000	-2.824082000	4.434839000
6	3.493732000	-4.698861000	3.682160000
1	3.411910000	-5.205837000	4.636858000
6	3.980872000	-5.363006000	2.588082000
1	4.294620000	-6.399387000	2.641200000
6	4.088090000	-4.709928000	1.334363000
6	4.670638000	-4.831765000	-0.870630000
1	5.054582000	-5.421076000	-1.699075000
6	4.306756000	-3.480582000	-1.098920000
1	4.414155000	-3.090225000	-2.102553000
7	4.576267000	-5.433558000	0.284526000
6	-5.387663000	-2.340683000	-2.500382000
9	-6.623068000	-2.835749000	-2.346630000
9	-5.531363000	-1.106668000	-3.023886000
9	-4.777153000	-3.079850000	-3.434427000
8	0.211861000	-0.798671000	2.132005000
6	0.719923000	-0.240339000	3.123996000
6	1.843162000	0.606479000	3.081133000
1	2.434619000	0.612120000	2.167508000
6	2.488560000	1.034003000	4.303238000
1	1.929550000	0.868898000	5.223745000
1	4.270452000	1.841380000	3.483105000
6	3.680052000	1.642293000	4.374199000
1	4.099825000	1.958527000	5.322346000
1	0.221944000	-0.350792000	4.102350000
1	2.349943000	0.644986000	-1.888477000
6	-3.457153000	1.052194000	-0.422976000
6	-2.440851000	1.341531000	0.507296000
6	-2.807813000	1.588427000	1.837453000
6	-4.117312000	1.537371000	2.297796000
6	-5.097234000	1.258915000	1.359222000
6	-4.769458000	1.027046000	0.012750000
6	-0.986650000	1.497306000	0.473807000
6	-0.642028000	1.917655000	1.777886000
1	-3.224911000	0.830953000	-1.454307000

1	-4.348871000	1.724212000	3.339354000
1	-6.134872000	1.219823000	1.670232000
1	-5.559044000	0.805561000	-0.696085000
8	-1.745052000	1.908770000	2.608525000
6	0.589709000	2.370146000	2.236129000
1	1.317383000	2.452602000	1.435226000
6	0.791723000	3.296362000	3.355697000
6	1.844909000	4.213651000	3.263106000
6	0.020397000	3.276123000	4.526542000
6	2.103087000	5.113399000	4.292085000
6	0.285367000	4.168767000	5.555603000
6	1.321881000	5.094896000	5.441554000
1	2.462114000	4.222581000	2.369656000
1	-0.776882000	2.552237000	4.635513000
1	2.918609000	5.821764000	4.197145000
1	-0.318857000	4.141578000	6.455726000
1	1.521926000	5.790253000	6.249058000
7	-0.046596000	1.361409000	-0.478699000
8	0.882426000	1.176599000	-2.731987000
8	-1.359750000	0.179469000	-2.393910000
16	-0.417884000	1.242965000	-2.033146000
6	-1.196155000	2.786885000	-2.486772000
6	-0.707327000	3.980006000	-1.958652000
6	-2.255625000	2.779581000	-3.383382000
6	-1.296534000	5.175755000	-2.339217000
6	-2.833679000	3.989508000	-3.755890000
6	-2.365607000	5.198778000	-3.242833000
1	0.113561000	3.969371000	-1.249165000
1	-2.627890000	1.841407000	-3.778490000
1	-0.926921000	6.109664000	-1.927363000
1	-3.664195000	3.989146000	-4.454068000
6	-2.994132000	6.508007000	-3.633636000
1	-2.246605000	7.192099000	-4.042769000
1	-3.774884000	6.367538000	-4.381804000
1	-3.440484000	6.996952000	-2.763766000

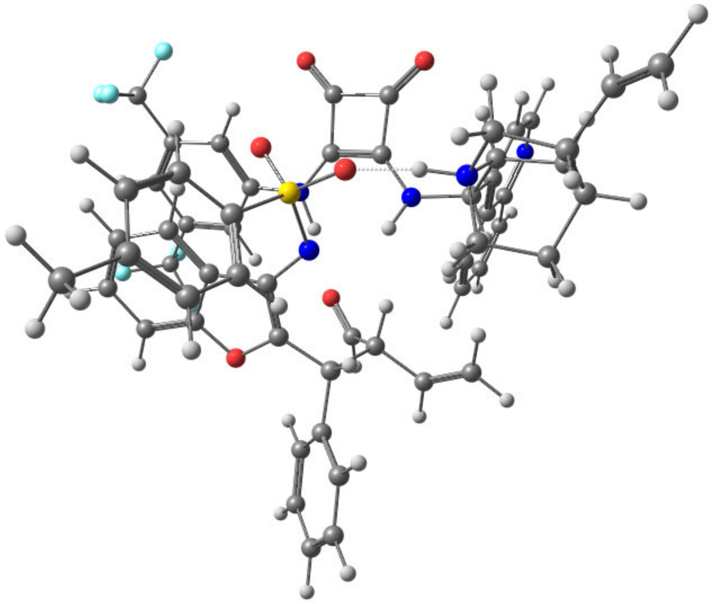

DiastA-II

Sum of electronic and thermal Free Energies= -3922.519046

(-14.2 kcal/mol)

6	3.617089000	-0.838196000	0.245577000
1	3.810377000	-0.030264000	0.958160000
6	1.542636000	-1.859545000	-0.623425000
6	1.914008000	-2.866627000	-1.625199000
6	0.452084000	-3.298295000	-1.639983000
6	0.204219000	-2.241936000	-0.621520000
6	-2.164177000	-2.135143000	-0.014696000
6	-2.680366000	-2.634918000	-1.206626000
1	-2.037389000	-2.790139000	-2.062585000
6	-4.039981000	-2.912570000	-1.291306000
6	-4.904993000	-2.678223000	-0.234387000
1	-5.965868000	-2.877813000	-0.323582000
6	-4.372454000	-2.150185000	0.938702000
6	-3.021364000	-1.882014000	1.062267000
1	-2.619582000	-1.471691000	1.982074000
6	-5.286462000	-1.907565000	2.102437000
9	-6.470595000	-1.405568000	1.719883000
9	-4.766302000	-1.056448000	2.996540000
9	-5.558160000	-3.045009000	2.767406000

7	2.174850000	-0.861422000	0.037755000
1	1.621314000	-0.014034000	0.170533000
7	-0.815030000	-1.809248000	0.133450000
1	-0.589297000	-1.176300000	0.901160000
8	2.918982000	-3.206924000	-2.205002000
8	-0.192441000	-4.114841000	-2.242013000
6	4.401757000	-0.526804000	-1.036759000
1	4.227726000	-1.326790000	-1.758085000
6	5.909164000	-0.277031000	-0.801875000
1	6.502039000	-0.964694000	-1.406705000
1	6.158554000	-0.476397000	0.242974000
6	6.245507000	1.183587000	-1.129221000
1	7.289005000	1.388426000	-0.887608000
6	5.318328000	2.084467000	-0.301813000
1	5.621595000	3.129196000	-0.379804000
1	5.373462000	1.800640000	0.752807000
6	3.886118000	1.924421000	-0.833847000
1	3.133232000	1.834667000	-0.050309000
1	3.586329000	2.750074000	-1.478415000
6	4.575715000	0.957581000	-2.967033000
1	3.994051000	1.677044000	-3.541135000
1	4.602139000	0.013829000	-3.512873000
6	5.991253000	1.475854000	-2.626145000
1	6.017189000	2.560739000	-2.764252000
6	7.018344000	0.859748000	-3.531431000
1	7.074244000	-0.228089000	-3.530000000
6	7.835461000	1.556439000	-4.312197000
1	7.799028000	2.642076000	-4.346999000
1	8.568767000	1.067855000	-4.944920000
7	3.828071000	0.697770000	-1.694746000
6	4.068959000	-2.134997000	0.908620000
6	3.711067000	-2.379607000	2.271499000
6	2.970101000	-1.465685000	3.062341000
1	2.636802000	-0.532739000	2.629305000
6	2.670196000	-1.746550000	4.369530000

1	2.108898000	-1.028866000	4.959612000
6	3.087654000	-2.964476000	4.954100000
1	2.840695000	-3.177512000	5.987904000
6	3.801553000	-3.870229000	4.215253000
1	4.136029000	-4.812400000	4.634535000
6	4.130226000	-3.601039000	2.862461000
6	5.152056000	-4.286144000	0.938296000
1	5.718703000	-5.046175000	0.407144000
6	4.788872000	-3.097418000	0.255237000
1	5.077302000	-2.997892000	-0.782399000
7	4.848445000	-4.542182000	2.182650000
6	-4.584370000	-3.350318000	-2.616796000
9	-5.799264000	-3.905958000	-2.515070000
9	-4.708447000	-2.308425000	-3.462439000
9	-3.790611000	-4.239263000	-3.227332000
8	-0.623602000	-0.537499000	2.873221000
6	-0.123272000	0.399358000	3.450341000
6	0.735908000	1.419507000	2.750923000
1	1.188496000	0.912023000	1.896976000
6	1.799185000	1.985794000	3.646559000
1	1.485257000	2.301081000	4.639779000
1	3.394593000	1.848842000	2.275104000
6	3.061703000	2.148267000	3.267667000
1	3.804561000	2.588545000	3.923685000
1	-0.305115000	0.540916000	4.530696000
1	2.827993000	0.549763000	-1.944569000
6	-3.381392000	0.591540000	-1.309354000
6	-2.651370000	1.142518000	-0.240724000
6	-3.369803000	1.587840000	0.876514000
6	-4.751863000	1.521016000	0.997614000
6	-5.445665000	0.986139000	-0.075329000
6	-4.762102000	0.528422000	-1.213624000
6	-1.245962000	1.419192000	0.076284000
6	-1.271448000	2.021763000	1.301125000
1	-2.880613000	0.194193000	-2.180843000

1	-5.249347000	1.875660000	1.892742000
1	-6.526142000	0.910912000	-0.030926000
1	-5.327262000	0.097053000	-2.032590000
8	-2.541632000	2.101210000	1.815412000
6	-0.147125000	2.568148000	2.118396000
1	0.541287000	3.048265000	1.415718000
6	-0.564014000	3.608710000	3.140503000
6	-0.008268000	4.886239000	3.076778000
6	-1.444398000	3.313721000	4.186079000
6	-0.321991000	5.852094000	4.030032000
6	-1.757130000	4.274710000	5.140593000
6	-1.196171000	5.547677000	5.066610000
1	0.680170000	5.127500000	2.272846000
1	-1.907139000	2.335367000	4.250373000
1	0.119433000	6.840226000	3.960797000
1	-2.444329000	4.029642000	5.942924000
1	-1.442148000	6.295879000	5.811850000
7	-0.037297000	1.173799000	-0.586628000
8	1.352916000	0.980979000	-2.608963000
8	-0.754250000	-0.305852000	-2.591238000
16	-0.056972000	0.910003000	-2.146214000
6	-0.905660000	2.281218000	-2.929320000
6	-0.738441000	3.565758000	-2.418540000
6	-1.691676000	2.056585000	-4.050996000
6	-1.370554000	4.630446000	-3.044071000
6	-2.316453000	3.135300000	-4.669598000
6	-2.167256000	4.431838000	-4.177182000
1	-0.131036000	3.725345000	-1.533797000
1	-1.821195000	1.048592000	-4.428052000
1	-1.251902000	5.633381000	-2.645458000
1	-2.933894000	2.962629000	-5.545082000
6	-2.856481000	5.599258000	-4.829504000
1	-2.141730000	6.388563000	-5.074708000
1	-3.364661000	5.300564000	-5.747039000
1	-3.600743000	6.032858000	-4.156186000

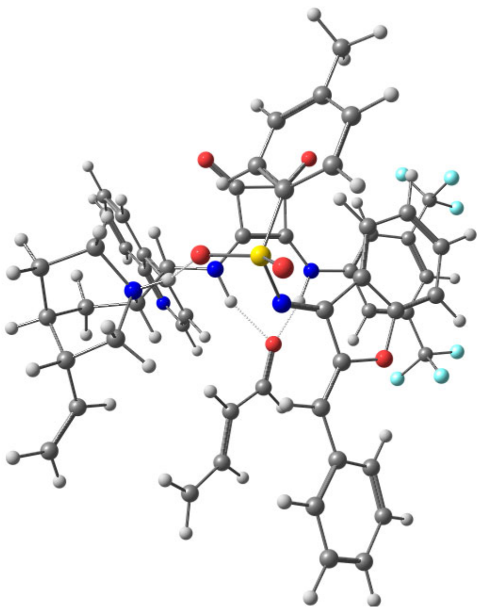

DiastB-I

Sum of electronic and thermal Free Energies= -3922.487159

(5.8 kcal/mol)

6	2.873043000	-1.382955000	-0.694257000
1	3.132824000	-2.308986000	-0.182340000
6	0.599968000	-1.839947000	-0.099170000
6	0.569986000	-3.244809000	0.295323000
6	-0.939806000	-3.212110000	0.084929000
6	-0.762147000	-1.811294000	-0.406972000
6	-2.821962000	-0.770757000	-1.278971000
6	-3.710713000	-1.822002000	-1.050550000
1	-3.364431000	-2.754806000	-0.618380000
6	-5.054146000	-1.648795000	-1.352821000
6	-5.548463000	-0.462862000	-1.882267000
1	-6.600675000	-0.345424000	-2.105852000
6	-4.642830000	0.561947000	-2.126462000
6	-3.296486000	0.422229000	-1.833130000
1	-2.606551000	1.241973000	-2.013608000
6	-5.105795000	1.884812000	-2.658474000
9	-6.387389000	1.867303000	-3.038706000
9	-4.381619000	2.283224000	-3.714267000
9	-4.986536000	2.856871000	-1.733015000

7	1.597017000	-0.923000000	-0.139158000
1	1.278077000	0.014283000	-0.440528000
7	-1.470882000	-0.817852000	-0.960660000
1	-0.936959000	0.051493000	-1.148642000
8	1.389504000	-4.062655000	0.649618000
8	-1.851245000	-3.974392000	0.280912000
6	3.933150000	-0.318135000	-0.418285000
1	3.547161000	0.634666000	-0.792777000
6	5.321543000	-0.605563000	-1.010641000
1	5.404563000	-0.120844000	-1.983870000
1	5.457891000	-1.679736000	-1.169330000
6	6.387114000	-0.100782000	-0.026821000
1	7.367216000	-0.090972000	-0.503829000
6	6.387472000	-1.032649000	1.192514000
1	7.013963000	-0.616907000	1.984221000
1	6.793579000	-2.009007000	0.924953000
6	4.938681000	-1.196651000	1.683774000
1	4.503563000	-2.152840000	1.396319000
1	4.833503000	-1.083668000	2.760879000
6	4.750365000	1.216012000	1.315249000
1	4.951364000	1.260506000	2.384351000
1	4.015741000	1.982580000	1.069628000
6	6.039651000	1.323831000	0.464748000
1	6.849873000	1.655401000	1.118592000
6	5.902058000	2.319351000	-0.655900000
1	5.036017000	2.219705000	-1.310913000
6	6.763266000	3.305731000	-0.877014000
1	7.634377000	3.452627000	-0.243589000
1	6.624625000	4.002514000	-1.697113000
7	4.103522000	-0.114812000	1.062274000
6	2.788174000	-1.622994000	-2.198468000
6	3.255991000	-2.822309000	-2.813978000
6	3.795842000	-3.932181000	-2.106398000
1	3.853244000	-3.916524000	-1.025353000
6	4.225695000	-5.047881000	-2.775014000

1	4.626339000	-5.887336000	-2.218327000
6	4.143294000	-5.120333000	-4.185438000
1	4.488975000	-6.010681000	-4.698307000
6	3.620210000	-4.074974000	-4.895958000
1	3.535001000	-4.102757000	-5.976343000
6	3.161394000	-2.907303000	-4.233393000
6	2.205014000	-0.836884000	-4.398123000
1	1.788475000	-0.055220000	-5.027582000
6	2.252913000	-0.646028000	-2.999163000
1	1.854714000	0.272617000	-2.579197000
7	2.643108000	-1.910828000	-5.004818000
6	-5.995512000	-2.761717000	-1.001504000
9	-7.184669000	-2.637831000	-1.602936000
9	-5.505192000	-3.963745000	-1.330752000
9	-6.234708000	-2.799339000	0.324050000
8	0.169987000	1.280973000	-1.017947000
6	0.623984000	2.391148000	-1.475868000
6	1.781233000	3.011615000	-1.084761000
1	2.368528000	2.553887000	-0.289769000
6	2.202236000	4.278498000	-1.627219000
1	1.564091000	4.689795000	-2.411385000
1	3.938896000	4.648447000	-0.457533000
6	3.273671000	4.994796000	-1.244442000
1	3.506377000	5.951158000	-1.699750000
1	0.020127000	2.901562000	-2.244933000
1	3.188325000	-0.107432000	1.537108000
6	-2.263239000	-0.623390000	2.245201000
6	-1.852442000	0.681453000	1.916486000
6	-2.790949000	1.536799000	1.326164000
6	-4.135174000	1.216792000	1.175675000
6	-4.524675000	-0.047141000	1.581068000
6	-3.589250000	-0.972563000	2.076962000
6	-0.602742000	1.442298000	1.957019000
6	-0.935616000	2.713781000	1.304498000
1	-1.561285000	-1.365616000	2.589120000

1	-4.827510000	1.926853000	0.741213000
1	-5.562779000	-0.342575000	1.477963000
1	-3.909972000	-1.978431000	2.323774000
8	-2.258571000	2.712147000	0.916411000
6	-0.130368000	3.782549000	1.180526000
1	0.855891000	3.653214000	1.613893000
6	-0.421959000	5.079950000	0.584305000
6	0.493555000	6.120119000	0.805423000
6	-1.539940000	5.330195000	-0.229787000
6	0.292364000	7.377994000	0.252903000
6	-1.731644000	6.586444000	-0.787306000
6	-0.822219000	7.615104000	-0.545462000
1	1.371470000	5.929617000	1.413953000
1	-2.250729000	4.541331000	-0.436615000
1	1.010482000	8.169006000	0.437384000
1	-2.595484000	6.764713000	-1.418020000
1	-0.980265000	8.594267000	-0.983733000
7	0.607286000	1.266318000	2.401860000
8	2.562009000	0.056678000	3.261828000
8	1.073366000	1.288714000	4.841591000
16	1.204434000	0.439690000	3.667745000
6	0.348933000	-1.072699000	4.014010000
6	0.674824000	-2.213955000	3.285173000
6	-0.651535000	-1.085510000	4.977264000
6	-0.050890000	-3.375790000	3.501267000
6	-1.363670000	-2.261876000	5.185884000
6	-1.088057000	-3.411575000	4.442925000
1	1.484260000	-2.191305000	2.563673000
1	-0.878079000	-0.189851000	5.543562000
1	0.185102000	-4.266967000	2.928448000
1	-2.154524000	-2.280258000	5.928144000
6	-1.872870000	-4.677888000	4.639569000
1	-1.226216000	-5.478391000	5.008155000
1	-2.685206000	-4.538605000	5.353021000
1	-2.297791000	-5.015847000	3.691113000

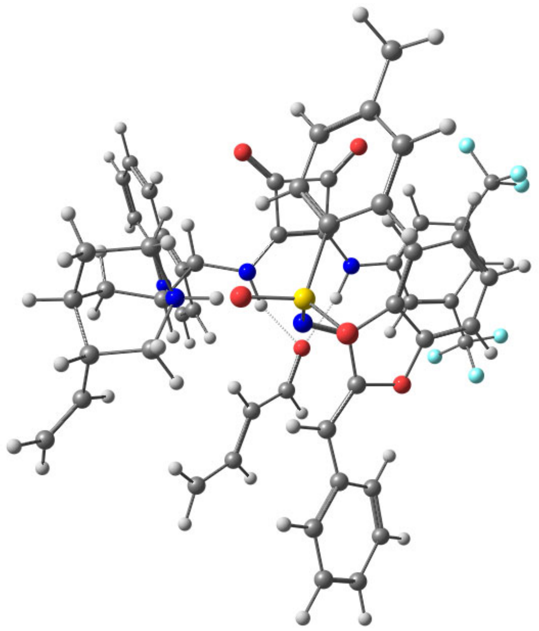

DiastB-TS1

Sum of electronic and thermal Free Energies= -3922.482393

(8.8 kcal/mol)

6	2.758859000	-1.432685000	-0.913745000
1	2.849790000	-2.409594000	-0.442683000
6	0.429522000	-1.790349000	-0.375668000
6	0.374940000	-3.146194000	0.170537000
6	-1.141405000	-3.061793000	0.081778000
6	-0.955937000	-1.725309000	-0.547842000
6	-3.044472000	-0.675877000	-1.345798000
6	-3.939603000	-1.669353000	-0.946717000
1	-3.585490000	-2.564204000	-0.446092000
6	-5.297033000	-1.482221000	-1.165003000
6	-5.799465000	-0.340957000	-1.778708000
1	-6.862474000	-0.212072000	-1.935239000
6	-4.889422000	0.622661000	-2.193338000
6	-3.528369000	0.469665000	-1.983820000
1	-2.834287000	1.243151000	-2.299510000
6	-5.357621000	1.894873000	-2.833895000
9	-6.666858000	1.883800000	-3.104896000
9	-4.714899000	2.143961000	-3.984668000
9	-5.131502000	2.958688000	-2.040482000

7	1.430317000	-0.926806000	-0.594260000
1	1.146090000	-0.005361000	-0.960634000
7	-1.674650000	-0.742538000	-1.119367000
1	-1.137040000	0.075669000	-1.426921000
8	1.192734000	-3.960167000	0.541983000
8	-2.059482000	-3.769018000	0.416134000
6	3.828836000	-0.496287000	-0.353853000
1	3.691063000	0.489915000	-0.802966000
6	5.260551000	-1.005539000	-0.590269000
1	5.664974000	-0.540552000	-1.489775000
1	5.252979000	-2.086611000	-0.760628000
6	6.110043000	-0.691164000	0.646664000
1	7.165205000	-0.865783000	0.434906000
6	5.632837000	-1.589873000	1.793499000
1	6.110576000	-1.290007000	2.728360000
1	5.904912000	-2.628225000	1.599127000
6	4.103192000	-1.476009000	1.916450000
1	3.582325000	-2.344894000	1.515043000
1	3.767345000	-1.313678000	2.936642000
6	4.439999000	0.933141000	1.542424000
1	4.347003000	0.998376000	2.623294000
1	3.946678000	1.798806000	1.098922000
6	5.907178000	0.779419000	1.073392000
1	6.556638000	0.971066000	1.930969000
6	6.260395000	1.756486000	-0.014167000
1	5.622048000	1.765849000	-0.897605000
6	7.285922000	2.597703000	0.048138000
1	7.941922000	2.629628000	0.914212000
1	7.504916000	3.284205000	-0.762970000
7	3.647716000	-0.277074000	1.127263000
6	2.963803000	-1.552977000	-2.419570000
6	3.354483000	-2.770752000	-3.050437000
6	3.561236000	-4.001567000	-2.367449000
1	3.396668000	-4.072717000	-1.299202000
6	3.945208000	-5.124827000	-3.051274000

1	4.091074000	-6.056049000	-2.515961000
6	4.145286000	-5.085340000	-4.451206000
1	4.449906000	-5.984086000	-4.975332000
6	3.946214000	-3.920268000	-5.139796000
1	4.084081000	-3.858752000	-6.213321000
6	3.545004000	-2.739888000	-4.462757000
6	2.985876000	-0.525945000	-4.596926000
1	2.838382000	0.357649000	-5.211821000
6	2.777552000	-0.442187000	-3.202519000
1	2.472274000	0.508729000	-2.774450000
7	3.359921000	-1.618090000	-5.213850000
6	-6.241531000	-2.510683000	-0.618891000
9	-7.456070000	-2.440631000	-1.177180000
9	-5.791961000	-3.759613000	-0.796483000
9	-6.417465000	-2.354499000	0.707942000
8	0.095211000	1.318561000	-1.523293000
6	0.700440000	2.410536000	-1.741584000
6	1.882685000	2.803770000	-1.135442000
1	2.342499000	2.122477000	-0.421414000
6	2.560812000	4.026701000	-1.472414000
1	2.071729000	4.652865000	-2.219628000
1	4.225949000	3.894435000	-0.156090000
6	3.704010000	4.465976000	-0.919896000
1	4.146296000	5.412010000	-1.211569000
1	0.236178000	3.126330000	-2.439943000
1	2.652703000	-0.081370000	1.328234000
6	-2.338711000	-0.400199000	2.146061000
6	-1.756502000	0.808538000	1.713308000
6	-2.582056000	1.763983000	1.103647000
6	-3.963741000	1.635358000	1.002391000
6	-4.514727000	0.469495000	1.501888000
6	-3.706139000	-0.551270000	2.041042000
6	-0.417421000	1.363633000	1.663006000
6	-0.570886000	2.619557000	1.008031000
1	-1.737008000	-1.210707000	2.530459000

1	-4.563773000	2.411717000	0.544193000
1	-5.587579000	0.321556000	1.447284000
1	-4.166389000	-1.476896000	2.367616000
8	-1.882851000	2.823334000	0.639805000
6	0.386182000	3.590597000	0.880984000
1	1.323643000	3.350857000	1.371318000
6	0.206354000	4.964552000	0.443536000
6	1.166466000	5.907965000	0.841428000
6	-0.852632000	5.383021000	-0.381514000
6	1.061203000	7.237310000	0.450875000
6	-0.944928000	6.707532000	-0.779027000
6	0.006797000	7.639336000	-0.361277000
1	1.990868000	5.590117000	1.470164000
1	-1.591585000	4.668538000	-0.719557000
1	1.805732000	7.955269000	0.775472000
1	-1.762475000	7.019013000	-1.419374000
1	-0.075591000	8.674627000	-0.673028000
7	0.782299000	0.930638000	2.095797000
8	2.450307000	0.284459000	3.761237000
8	0.424156000	1.528335000	4.542277000
16	1.010309000	0.536993000	3.644437000
6	0.212926000	-1.016221000	3.999258000
6	0.547316000	-2.127714000	3.228608000
6	-0.761282000	-1.091441000	4.982105000
6	-0.126909000	-3.321108000	3.432779000
6	-1.425355000	-2.299982000	5.182575000
6	-1.127995000	-3.421635000	4.409598000
1	1.312355000	-2.055159000	2.460998000
1	-1.013141000	-0.212052000	5.562636000
1	0.114299000	-4.183758000	2.820153000
1	-2.196466000	-2.363720000	5.943334000
6	-1.854565000	-4.723351000	4.604332000
1	-1.163481000	-5.507428000	4.924678000
1	-2.639749000	-4.633154000	5.355522000
1	-2.309049000	-5.055245000	3.667679000

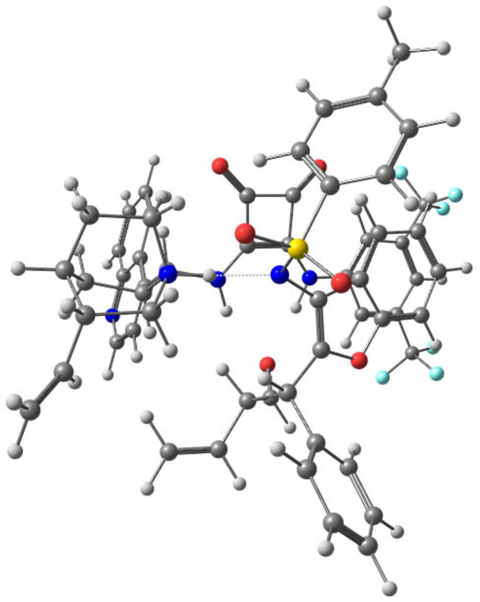

DiastB-II

Sum of electronic and thermal Free Energies= -3922.522168

(-16.2 kcal/mol)

6	2.656396000	-2.020083000	-0.423455000
1	2.612370000	-2.661305000	0.455075000
6	0.325575000	-1.716524000	0.231234000
6	0.211319000	-2.400773000	1.521100000
6	-1.297277000	-2.264969000	1.400953000
6	-1.056030000	-1.601363000	0.089428000
6	-3.125173000	-0.969777000	-1.111592000
6	-4.041783000	-1.513323000	-0.212605000
1	-3.705359000	-2.053229000	0.665050000
6	-5.397221000	-1.317754000	-0.433124000
6	-5.874185000	-0.605526000	-1.526237000
1	-6.936368000	-0.461115000	-1.679338000
6	-4.944557000	-0.094157000	-2.422147000
6	-3.583386000	-0.265336000	-2.226416000
1	-2.872219000	0.157930000	-2.929100000
6	-5.402742000	0.737653000	-3.582120000
9	-6.665505000	0.470611000	-3.934288000
9	-4.638305000	0.555404000	-4.668142000
9	-5.351102000	2.051923000	-3.297379000

7	1.365718000	-1.337500000	-0.525926000
1	1.154174000	-0.902778000	-1.421719000
7	-1.744583000	-1.077361000	-0.942987000
1	-1.187388000	-0.684259000	-1.696137000
8	0.984650000	-2.891921000	2.313837000
8	-2.238602000	-2.580056000	2.082512000
6	3.802237000	-1.032139000	-0.213689000
1	3.823036000	-0.312207000	-1.035074000
6	5.166563000	-1.734839000	-0.073844000
1	5.683394000	-1.729032000	-1.033377000
1	5.019067000	-2.784491000	0.201137000
6	5.983484000	-1.037140000	1.021308000
1	7.012069000	-1.399581000	1.010984000
6	5.319503000	-1.335647000	2.369100000
1	5.799534000	-0.754610000	3.159278000
1	5.432281000	-2.391868000	2.618588000
6	3.824885000	-0.980611000	2.277935000
1	3.178961000	-1.856429000	2.249454000
1	3.494593000	-0.339204000	3.090431000
6	4.509230000	0.980455000	0.961462000
1	4.347027000	1.535983000	1.882343000
1	4.185119000	1.598485000	0.120791000
6	5.973614000	0.492324000	0.809006000
1	6.562747000	0.945810000	1.609840000
6	6.580971000	0.897721000	-0.504694000
1	6.132483000	0.478464000	-1.404597000
6	7.616699000	1.720160000	-0.624227000
1	8.093479000	2.160745000	0.247369000
1	8.024588000	1.983100000	-1.594630000
7	3.578844000	-0.196612000	1.016858000
6	2.863987000	-2.881454000	-1.661028000
6	2.476247000	-4.256965000	-1.670556000
6	1.926212000	-4.941945000	-0.552903000
1	1.768276000	-4.428664000	0.388644000
6	1.579009000	-6.264721000	-0.643973000

1	1.157891000	-6.768956000	0.218174000
6	1.762554000	-6.977659000	-1.850886000
1	1.484381000	-8.024018000	-1.903984000
6	2.286087000	-6.346916000	-2.946778000
1	2.435761000	-6.863626000	-3.887901000
6	2.648931000	-4.977774000	-2.886752000
6	3.476724000	-3.146421000	-3.977238000
1	3.868256000	-2.708998000	-4.891550000
6	3.352346000	-2.339042000	-2.822064000
1	3.647281000	-1.297683000	-2.890911000
7	3.150133000	-4.411051000	-4.021831000
6	-6.367011000	-1.804482000	0.601708000
9	-7.571385000	-2.076717000	0.082029000
9	-5.940475000	-2.911453000	1.221288000
9	-6.563629000	-0.879179000	1.559856000
8	0.152163000	0.322937000	-2.788663000
6	0.614413000	1.433066000	-2.948517000
6	1.788177000	1.943925000	-2.158365000
1	2.214336000	1.083888000	-1.634415000
6	2.821583000	2.549249000	-3.065893000
1	2.505204000	3.390364000	-3.678173000
1	4.408102000	1.273845000	-2.523361000
6	4.073486000	2.111932000	-3.127651000
1	4.808934000	2.575555000	-3.776172000
1	0.170438000	2.106448000	-3.700107000
1	2.599068000	0.197582000	1.064438000
6	-2.181418000	0.796560000	1.993355000
6	-1.384771000	1.461765000	1.050657000
6	-2.024560000	2.126925000	0.000177000
6	-3.402736000	2.234287000	-0.130964000
6	-4.168596000	1.609529000	0.842838000
6	-3.561390000	0.882624000	1.879421000
6	0.046896000	1.662101000	0.824711000
6	0.125601000	2.407040000	-0.310356000
1	-1.740281000	0.220882000	2.796725000

1	-3.847868000	2.776633000	-0.957117000
1	-5.250403000	1.662280000	0.788159000
1	-4.184313000	0.370877000	2.605470000
8	-1.110364000	2.668807000	-0.850801000
6	1.321831000	2.936699000	-1.024434000
1	2.130556000	2.896424000	-0.290617000
6	1.196520000	4.380292000	-1.479705000
6	2.136992000	5.306160000	-1.025554000
6	0.209963000	4.812784000	-2.371110000
6	2.096763000	6.632780000	-1.445186000
6	0.169338000	6.137431000	-2.793174000
6	1.112565000	7.052167000	-2.332963000
1	2.907844000	4.985225000	-0.331738000
1	-0.542686000	4.121798000	-2.730629000
1	2.834561000	7.336262000	-1.075462000
1	-0.605412000	6.456155000	-3.481866000
1	1.077108000	8.084589000	-2.662402000
7	1.157324000	1.223528000	1.568085000
8	2.721930000	1.638853000	3.435546000
8	1.009736000	3.391260000	2.951739000
16	1.364026000	1.969080000	2.970711000
6	0.253326000	1.187699000	4.137579000
6	0.339964000	-0.194548000	4.300777000
6	-0.735574000	1.918660000	4.774113000
6	-0.590210000	-0.844117000	5.095228000
6	-1.662098000	1.253347000	5.576967000
6	-1.611514000	-0.129744000	5.738655000
1	1.109491000	-0.761248000	3.786456000
1	-0.796321000	2.989695000	4.620679000
1	-0.541010000	-1.923223000	5.205817000
1	-2.446392000	1.820846000	6.067674000
6	-2.625678000	-0.859716000	6.576503000
1	-2.145858000	-1.356059000	7.424248000
1	-3.384566000	-0.180031000	6.965743000
1	-3.127407000	-1.633117000	5.989305000
